# Comparative RNA-Seq and Microarray Analysis of Gene Expression Changes in B-Cell Lymphomas of *Canis familiaris*

**DOI:** 10.1371/journal.pone.0061088

**Published:** 2013-04-04

**Authors:** Marie Mooney, Jeffrey Bond, Noel Monks, Emily Eugster, David Cherba, Pamela Berlinski, Steve Kamerling, Keith Marotti, Heather Simpson, Tony Rusk, Waibhav Tembe, Christophe Legendre, Hollie Benson, Winnie Liang, Craig Paul Webb

**Affiliations:** 1 Laboratory of Translational Medicine, Van Andel Research Institute, Grand Rapids, Michigan, United States of America; 2 Department of Microbiology and Molecular Genetics, University of Vermont, Burlington, Vermont, United States of America; 3 Pfizer Animal Health, Pfizer Inc, Kalamazoo, Michigan, United States of America; 4 Animal Clinical Investigation, Washington, D.C., United States of America; 5 Collaborative Bioinformatics Center, Translational Genomics Research Institute, Phoenix, Arizona, United States of America; 6 Collaborative Sequencing Center, Translational Genomics Research Institute, Phoenix, Arizona, United States of America; Auburn University, United States of America

## Abstract

Comparative oncology is a developing research discipline that is being used to assist our understanding of human neoplastic diseases. Companion canines are a preferred animal oncology model due to spontaneous tumor development and similarity to human disease at the pathophysiological level. We use a paired RNA sequencing (RNA-Seq)/microarray analysis of a set of four normal canine lymph nodes and ten canine lymphoma fine needle aspirates to identify technical biases and variation between the technologies and convergence on biological disease pathways. Surrogate Variable Analysis (SVA) provides a formal multivariate analysis of the combined RNA-Seq/microarray data set. Applying SVA to the data allows us to decompose variation into contributions associated with transcript abundance, differences between the technology, and latent variation within each technology. A substantial and highly statistically significant component of the variation reflects transcript abundance, and RNA-Seq appeared more sensitive for detection of transcripts expressed at low levels. Latent random variation among RNA-Seq samples is also distinct in character from that impacting microarray samples. In particular, we observed variation between RNA-Seq samples that reflects transcript GC content. Platform-independent variable decomposition without *a priori* knowledge of the sources of variation using SVA represents a generalizable method for accomplishing cross-platform data analysis. We identified genes differentially expressed between normal lymph nodes of disease free dogs and a subset of the diseased dogs diagnosed with B-cell lymphoma using each technology. There is statistically significant overlap between the RNA-Seq and microarray sets of differentially expressed genes. Analysis of overlapping genes in the context of biological systems suggests elevated expression and activity of PI3K signaling in B-cell lymphoma biopsies compared with normal biopsies, consistent with literature describing successful use of drugs targeting this pathway in lymphomas.

## Introduction

Since domestic dogs, *Canis familiaris*, form spontaneous tumors and have a strong breed predilection towards specific types of cancer, the dog is an excellent model for identifying the genetic underpinnings associated with cancer (reviewed in Shearin and Ostrander [1] and Rowell et al [2]). Moreover, the disciplines of veterinary and human medicine use the same diagnostic and therapeutic tools, and the canine response to chemotherapies is more similar to the human response than other model systems [2]. The similarities in cancer treatment and response between species coupled with a shorter canine lifespan and higher frequencies of cancer occurrence in specific breeds afford an opportunity to accelerate the application of advanced diagnostic and interventional strategies for the benefit of both dogs and humans.

The analogous nature between human and canine disease counterparts is typified by canine lymphoma, which shares many of the epidemiological, biological, and clinical features of Non-Hodgkin's Lymphoma (NHL) in humans [2]–[4]. NHL is the fifth most common cancer in the United States and a cancer that has nearly doubled in worldwide incidence in the past 35 years to a lifetime incidence of 1 in 47 people [5], [6]. In the canine population, lymphoma represents approximately 24% of all canine cancer diagnoses and has a similar lifetime incidence to humans [2]. However, breeds such as the golden retriever and boxer have an even stronger predilection for B- and T- cell lymphomas, with lifetime incidences of 1 in 4 and 1 in 8 dogs, respectively [2].

Canine lymphoma most commonly manifests as multicentric lymphadenopathy with or without other organ involvement [7]. This multicentric presentation dictates systemic chemotherapy as the treatment of choice. Treatment is a multi-agent protocol that is based on the CHOP regime (adriamycin, vincristine, cyclophosphamide, and prednisone) used as standard-of-care in human NHL patients [7], [8]. However, many variations on this protocol exist in both veterinary and human medicine [7], [9], [10]. A detailed investigation of the molecular mechanisms of the etiology and progression of canine lymphomas is warranted for improving diagnostic and therapeutic strategies that may have direct translational relevance to human NHL patients.

Next Generation Sequencing (NGS) is the next frontier for scrutinizing molecular pathology. As sequencing cost decreases and access to instrumentation increases, NGS will likely replace microarrays for gene expression analysis due to added benefits such as quantitation of transcripts, improved dynamic range, and additional capabilities for detecting expressed single nucleotide variants (SNVs), translocations, and transcript isoform switches [11], [12]. However, the NGS field is still in flux, the internal standards for data quality, reliability, and reproducibility are still being established, and disparities between the technologies are still being investigated [13]–[17]. Generally, hybridization and sequencing technologies are viewed as complementary rather than competing approaches [14], [18], and microarrays continue to be used frequently for burgeoning model organisms like the dog [19], [20]. Examining methods for comparing expression data between the two technologies remains a critical task for the continued and comparative use of microarray data in the NGS era.

Our data include gene expression profiles for each of ten canine lymphoma samples and four lymph nodes samples from disease free subjects using both Illumina NGS and Affymetrix microarray technology. This design is a member of an important class: multivariate observations made in batches that exhibit *latent* variation with very different covariance. Our objective is to decompose variation among the gene expression profiles so that we can directly inspect 1) variation in transcript abundance among samples 2) differences in sensitivities of the two technologies and 3) latent variation due to each technology. Many studies have address the first two issues by gene or tag counts and assessments against PCR data, respectively [21], [22], or even using proteomic shot gun mass spectroscopy as a metric for sensitivity [23]. It is the latent variation due to technology that presents the challenge since numerous sources of technical variation can contribute to differing extents within and between technologies (as an example, 57% of the total expression variation between microarray and RNA-Seq data in [23] was unexplained). Variation among samples with respect to fragment length, coverage, GC content, amplification technology, proportions of cell types, proliferation rate, RNA degradation, preparative processes, or instrumentation may impact estimates of genome-wide expression profiles [24]–[29]. Numerous investigators have described methods for capturing latent variation [30]–[36]. While typically the goal of capturing latent variation is to improve inference about experimental factors impinging on biology, we are also interested in the direction and magnitude of latent technical variation for the purpose of comparing RNA-Seq with microarray technology. However, our design does not include replicated observations on RNA samples within each technology. Instead, statistical analysis of the *combined* RNA-Seq and microarray data provides for capturing latent variables within each technology. Understanding distinct technological variation is a prerequisite to examining biologically pertinent transcriptional pathways.

## Methods

### Ethics Statement

All studies were approved by the Institutional Animal Care and Use Committee (IACUC) at Animal Clinical Investigation (ACI) concomitant with owner consent forms.

### Samples

Fine needle aspirates (FNAs) were collected longitudinally from 30 dogs with lymphosarcoma as part of a study conducted by Pfizer Animal Health for the purpose of finding the maximum tolerated dose of an investigational phosphatidylinositol 3-kinase (PI3-K) inhibitor. Inclusion criteria required that at least one lymph node tumor measure >20 mm in diameter so that FNAs could be collected at 0 hr, 6 hr, and 24 hr after treatment from a single node. Samples were collected at three clinical sites under the coordination of ACI, Washington, DC. These sites were Friendship Animal Hospital, Washington, DC (sample id FS); Red Bank Veterinary Hospital, Tinton Falls, NJ (sample id RB); and New England Veterinary Oncology Group, Waltham, MA (sample id NE). After FNA collection, the 30 samples were shipped to the CLIA (Clinical Laboratory Improvement Amendments) accredited Clinical Reference Laboratory (CRL) for RNA isolation and genomic profiling using the GeneChip Canine Genome V2.0 Array (Affymetrix). Seven days after treatment, the change in lymph node tumor volume was assessed and used to classify responders and non-responders. Five responder and five non-responder 0 hr (pre-treatment) RNA samples previously used for GeneChip analysis were chosen to undergo additional total-RNA transcriptome sequencing using the Illumina HiSeq 2000 platform at the Translational Genomics Research Institute (TGen). In addition, a control set of four non-diseased lymph node FNAs were collected from three dogs in the Pfizer Beagle animal colony for RNA isolation and genomic profiling on both the GeneChip and HiSeq 2000. The ten lymphoma and four non-diseased lymph node samples with both GeneChip and HiSeq data are the focus of this report. Four of the lymphoma dogs had been given previous treatment, as summarized in Table S1 that provides clinical details for each dog.

### Sample sets for analysis

Genome-wide expression profiles were obtained from fourteen RNA samples using both RNA-Seq and microarray. The set of samples chosen for each analysis depended on the stated goal of the analysis (Table 1). For comparing RNA-Seq with microarray, all samples were used. For comparing B-cell lymphoma with normal biopsies we required that the cytological analysis confirmed >70% lymphoblasts, that immunophenotyping for CD79a staining was present among the majority of lymphoblasts, and that genome-wide expression profiling did not identify a gene expression signature indicative of a substantial proportion of T-cells involvement.

**Table 1:** Sample Sets for Comparative Analyses.

Id	Sample set	Goal of analysis
All	10 cancer, 4 normal	Comparison of RNA-Seq with microarray. All samples.
BvN	4 B-cell lymphoma, 3 normal	Comparison of B-cell lymphoma with normal biopsies. Samples NE42, FS21, RB03, RB16.

### Cytology

Cytology slides were prepared and Immunohistochemistry (IHC) was performed for each FNA through ACI. The cytology report was used to determine the percentage of lymphoblasts in the sample, and IHC staining for CD3, CD79a, and MDR was used to designate a T-cell, B-cell, and Multi-Drug Resistant immunophenotype, respectively (Table S1).

### Microarray

RNA from FNA was extracted and purified with the RNeasy Micro kit (Qiagen) at Clinical Research Laboratory (CRL) and in order to proceed to genome profiling a sample had to exceed the quality control (QC) requirements: RNA yield >20 ng, A260/280≥1.8, and RNA Integrity Number (RIN)≥6.0. RNA that passed QC was amplified using the WT-Ovation™ Pico RNA Amplification System (NuGen Technologies, Inc). CRL carried out genomic profiling in 8 batches of 12 samples (all three time points for four subjects in each batch) using the GeneChip Canine Genome V2.0 Array (Affymetrix). GeneChip data was normalized to a 95% trimmed mean intensity of 500 using the MAS 5.0 algorithm within the Affymetrix Gene Console. Distributional analysis was performed using the R Affy package and image plots were generated with the AffyPLM package. Arrays with greater than 3-fold change from mean or lower than 30% present probes compared to the total number of probes on the array failed QC. In addition, visual inspection of the array images was performed to identify arrays that passed QC but had clear visual anomalies (e.g. air bubbles on the array). Re-processing occurred in instances of failure where sufficient RNA from the sample remained available. The distributional analysis and visual inspection was done for the longitudinal samples of all 30 subjects; none of the pre-treatment samples from the ten subjects used for additional RNA-Seq profiling or samples from the normal lymph nodes failed QC or required re-processing.

Microarray files for the 14 samples analyzed in this report are available on GEO, accession GSE41917.

### RNA-Seq

NGS was performed on the same RNA that was isolated at CRL and used for microarray analysis for ten of the 30 dogs (individually listed in Table S1) and on RNA from the additional four non-disease samples. 2 µg total RNA from each of these 14 samples was shipped from VARI to TGen for transcriptome sequencing using the Illumina HiSeq 2000. Upon receipt at TGen, the RNA QC was repeated. A more stringent QC was applied to the selection of these samples, which needed to exceed a minimum RIN of 9. All samples passed QC. Then, 10 ng of total RNA from each sample was processed with the Ovation RNA-Seq System (Nugen) for transcriptome amplification. Amplified samples were fragmented using Adaptive Focused Acoustic (AFA) Technology (Covaris - Model E210) to an approximate base pair target of 400 bp. Samples were end repaired using New England Biolab's NebNext DNA Sample Prep Master Mix Set with the addition of NebNext End Repair Reaction buffer and 5 µL NebNext End Repair Enzyme Mix (New England Biolabs) and purified using Ampure XP beads (Beckman Coulter). ‘A’ bases were added using NebNext's dA-tailing Reaction Buffer and Klenow enzyme (New England Biolabs). Samples were purified again using Ampure XP beads. Resulting samples were quantitated using Picogreen DNA quantification assay system (Invitrogen). A 10∶1 adaptor to DNA molar ratio was used to determine the appropriate amount of sample for ligation to Illumina's paired end annealed adaptors. Ligation was performed with the NebNext Quick Ligation buffer and NebNext DNA ligase (New England Biolabs). Ligation products were run on a 3% TAE gel for 2.5 hours at 120V to separate products. Cuts were made in the gel at 350 bp and 400 bp to extract the products with the appropriate insert size. USA Scientific's x-tracta™ gel extractors (USA Scientific) were used for making cuts. Cuts were inserted into Bio-Rad's Freeze ‘N Squeeze DNA Gel Extraction spin columns (Bio-Rad). Samples were purified using Ampure XP beads and enriched using PCR with Finnzymes 2X Phusion® High Fidelity PCR 2x Master Mix (Thermo Scientific). PCR products were run on a 2% TAE gel for 2 hours at 120 V and final products were punched using x-tracta gel extractors. DNA was extracted from the cuts using Freeze ‘N Squeeze columns, cleaned using Ampure XP beads, and run on an Agilent High Sensitivity Bioanalyzer chip DNA Kit (Agilent Technologies) to verify libraries. Average final library size was 260 bp.

Total RNA was sequenced on two flow cells (A and C) on the Illumina HiSeq 2000. The final sample concentrations were 11.0 pM on flow cell A and 12.0 pM on flow cell C. Standard paired-end sequencing using Illumina SBS sequencing Kit reagents (Illumina, Inc.) occurred over 9 days with turnaround chemistry at four days post-initiation. Binary data from the sequencer was converted into plain text format using BCL Converter software (version 1.7.1 - Illumina, Inc.) software using the default quality thresholds to discard low quality reads. This data was preprocessed and converted to standard FASTQ format containing 100-mer paired-end reads. These FASTQ files were used as inputs for the data analysis. The workflow is presented in Figure S1. Sequencing coverage statistics are listed in Table 2.

**Table 2:** Summary Statistics of RNA-Seq Alignment.

	# Reads Total (millions)	# Mapped Reads (millions)	Mapped Reads (% of Total)	# Annotated Mapped Reads (millions)	Annotated Mapped Reads (% of Total)	# Annotated Uniquely Mapped Reads (millions)	# Annotated Exactly Mapped Reads (millions)
155-1 (NE-45)	173	167	96.5	113	65.3	37.7	20.5
155-2 (RB-07)	79.6	76.4	96.0	65	81.7	23.8	9.9
155-3 (FS-25)	54.8	52.2	95.3	47.2	86.1	22.4	13.1
155-4 (FS-22)	76.6	73.6	96.1	63.6	83.0	25.1	9.9
155-6 (3024l)	61.6	58.0	94.2	51.4	83.4	20.8	9.7
155-7 (0063)	73.6	69.0	93.8	61.8	84.0	26.2	13.5
157-1 (RB-10)	106	99.4	93.7	81.2	76.6	32.5	13.1
157-2 (NE-42)	114	108	95.0	91.2	80.0	34.0	12.3
157-3 (NE-41)	99.8	94.2	94.4	78.6	78.8	39.4	16.4
157-4 (RB-03)	108	102	94.4	92.2	85.4	42.5	20.6
157-5 (FS-21)	112	105	93.6	95.8	85.5	30.4	12.5
157-6 (RB-16)	148	141	95.2	126.2	85.3	57.2	29.3
157-7 (3024r)	134	126	93.7	102.8	76.7	34.6	14.2
157-8 (2970)	94.0	87.6	93.2	78.8	83.8	31.8	16.0

Code 155 designates flow cell A, code 157 designated flow cell C, followed by the lane number. The sample ID is in parentheses. Normal samples are bolded. Annotated mappings result from Bowtie alignment with the Ensembl 63 GTF file. Uniquely mapped reads are mapped to only one region of the genome by the Bowtie aligner, and exactly mapped reads map to only one location and have no mismatches to the reference.

For differential expression analysis, sequencing data from each of the 14 lanes was analyzed in parallel, using 8 processing cores per lane and 112 processing cores simultaneously on the Saguaro2 high performance computing cluster resource (jointly provided by TGen and Arizona State University). The data was aligned to the canFam2 reference genome using Bowtie [37] both with and without an Ensembl annotation file (Canis_familiaris.BROADD2.63.gtf). Alignments were processed within Tophat [38] (version 1.2.0) to identify loci and splice junctions. Next, Cufflinks [39](version 1.0.2) was run with the Ensembl annotation file to estimate the relative abundance of the transcripts in the data. The fragments per million mapped read (fpkm) metrics at the gene and transcript level were used for subsequent statistical analysis of differential expression and sample variation.

Compressed raw sequencing files are provided in the Short Read Archive, accession SRA059558.

### Statistical Analysis

Calculations were performed using the R language and environment for statistical computing and graphics [40]. Extensive use was made of Bioconductor packages [41]. Surrogate Variable Analysis [33], [34] was performed using the sva package [42]. Sequences associated with Ensembl gene identifiers (by Ensembl version 63) were obtained using Biomart [43]. GC content was calculated using the seqinR package [44]. Distance-based multivariate analysis was performed using the vegan package [45].

#### Microarray expression statistics

Probe intensities were summarized for each probe set in each sample, and a detection call was made, using MAS5. Probe sets, Ensembl genes, and (Probe Set, Ensembl gene) pairs obtained from Biomart [43] define a bipartite graph. Connected subgraphs containing one Ensembl gene and at least one probe set define the set of Ensembl genes queried using microarray. An Ensembl gene was called present in a sample if at least one of the associated probe sets was called present. Genes that were not called present in at least three of thirteen samples were removed. Summary expression intensities for a gene were the average of the associated probe set intensities.

#### RNA-Seq expression statistics

Genes that were not called present in at least three of thirteen samples were removed. The value three was chosen because the smaller, non-disease group has three samples from different dogs. We do not require presence in four samples because we consider that the two samples taken from the same dog artificially reduce the variance and we do not want to discard genes that may be present in only normal but not disease dogs. Log-transformed expression statistics,, were obtained from FPKM using
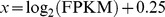



The addition of 0.25 eliminates the singularity at FPKM = 0. We chose 0.25 because it is approximately the 5% quantile of the non-zero FPKM and because in practice it does not substantially impact the results.

#### Univariate analysis

Genes differentially expressed between two sample groups (Table 1: B-cell vs Normal (BvN); All) were identified using the Welch two sample t-test. Normality was assessed for each technology and sample group by inspecting the distribution of gene p-values obtained using the Shapiro-Wilk test. We found no evidence for rejecting the normality assumption for oligonucleotide arrays or for normal RNA-Seq samples, that is, the gene p-value distributions are flat. A stronger statement about the normality would require additional samples. For RNA-Seq cancer profiles, there was some evidence for rejecting the normality assumption but it impacted fewer than 20% of the genes. The false discovery rate was limited using the method of Benjamini and Hochberg [25]. Permutation analysis based on the t-statistic provided clear evidence for differential expression: for 17% (RNA-Seq) or 22% (oligonucleotide arrays) of the 7,296 genes the maximum absolute value of the t-statistic was associated with the natural assignment to sample groups, compared with 1/35 under the null hypothesis.

#### Multivariate analysis on combined data

We used Surrogate Variable Analysis (SVA) [33], [42] to model expression data. As described above, multivariate observations were made in batches defined by the genomic technology; therefore batches may exhibit latent variation having different covariance. This suggests modeling genome-wide expression using
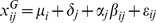
(1)where 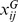 is the expression profile (that is, a vector of G expression levels) associated with RNA sample i and measurement type j, is the expression profile for sample i, is the difference in location of measurement type j from due to the technology (that is, a technical batch effect), is a G×L_j_ matrix describing latent variation associated with measurement type j, is the L_j_- vector of coefficients that combine the columns of , and is error for which the covariance matrix is diagonal. Use of Eq. 1 requires choice of L_j_. Additional constraints would be required for identifiability.

Surrogate Variable Analysis (SVA) [33], [42] provides for decomposition based on a similar bilinear model,
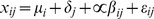
(2)where is a G×L matrix of surrogate variable loadings that must describe latent variation among all samples. Like Eq 1, Eq 2 accommodates both batch and latent variation. The rows of {} are not constrained as by Eq 1 and SVA chooses rows of {} to be orthogonal. We found, however, that SVA provided surrogate variables that satisfactorily capture the important features of the latent variables described in Eq 1 (see RESULTS). Therefore we used the results of SVA rather than develop and solve a probability model associated with Eq 1.

### Pathway Analysis

The set of canine genes (Ensembl identifiers) called present in at least three BvN samples by RNA-Seq and microarray (as per the *RNA expression statistics* section) was converted to a human gene space (Entrez identifiers) via BioMart and the R package Homovert for use with Gene Set Enrichment Analysis (GSEA) and GeneGO pathway analysis. This set is directly input into GSEA (v2.07) and run by setting the ‘collapse to gene symbol’ option to false, using ‘on-the-fly’ phenotypes corresponding to the labels for lymphoma samples versus normal samples, and permuting 1000 times on the gene set rather than the phenotype due to the small sample sizes. This is done separately for the microarray and RNA-Seq data since the intensity values are not equivalently derived. The gene set database versions 2.5 and 3.0 for all curated sets was used to show results in the expanded gene set space and persistent lymphoma hits.

Additionally, the positively expressed set of these genes was used to generate a topology map of ‘transcriptional activators’ by the Hidden Nodes Algorithm [46]. Then the positive expression data from the gene list and the topology map were overlaid on the pathway maps in GeneGo using the ‘Compare Experiments’ workflow.

Each individual canine sample was also processed within an internal personalized medicine engine, which required that the canine Affymetrix IDs be converted to human Affymetrix IDs for microarray data and that RNA-Seq canine Ensembl IDs be converted to human Affymetrix IDs, since the system was designed for use with human U133 2.0 Plus Affymetrix GeneChip microarray technology.

The canine microarray conversion occurs through the use of an in-house canine conversion tool that we routinely use. In this tool, each probeset's intensity value is converted to a Z-Score depicting its expression in terms of the number of standard-deviations from the mean expression of a normal canine reference set based on the 39 samples in GEO data set GSE20113. In cases where multiple probesets represent the same gene, they are aggregated to a single mean value for the appropriate Entrez gene identifier. The canine Entrez gene identifiers are then converted to human Entrez IDs using the homolog data from the NCI HomoloGene database 11/15/2010. Any ambiguously mapped canine IDs are removed. Finally, human Entrez gene identifiers are converted to human Affymetrix U133 2.0 Plus probesets based on the Affymetrix version 31 annotation file. The canine RNA-Seq conversion relies on a mean-based aggregation process using the Biomart mapping between canine Ensembl gene IDs and canine Affymetrix probeset IDs prior to the use of the canine conversion tool with a normal canine reference derived from the mean expression of the four normal RNA-Seq samples.

## Results

### Overview of RNA Sequencing Data

We sequenced 14 lymph node samples from 13 dogs, with each sample occupying one lane on an Illumina Hi-Seq 2000 flowcell. This produced between 55 and 175 million 100 bp paired-end reads per sample (Table 2). For comparative analysis with microarrays, we focused only on those reads which aligned to annotated regions of the canine genome, as microarray probes are designed primarily against annotated regions. We did not restrict the sequencing data to uniquely or exactly mapped reads, which is too stringent for the expected base-calling error of the sequencer [47] and similar to microarrays that allow for some measure of cross-hybridization. Instead, we allow for three mismatches (inclusive) within a read and a maximum read depth of 500 per bp.

### RNA-Seq appears more sensitive for detecting gene expression

Both RNA-Seq and microarray observations provide present detection calls for 15,092 genes in each of the 14 samples. The percent present detection calls provided by the two technologies agreed with high frequency (73%) and were statistically associated (Table 3; p<10^−15^, odds ratio >40). Among genes probed by both methods, percent present detection frequencies of 69% and 44% were obtained by RNA-Seq and microarray, respectively. Among genes called present using microarray over 97% were detected using RNA-Seq.

**Table 3:** Detection calls by Technology.

		RNA-Seq	
		Absent	Present
Microarray	**Absent**	30%	26%
	**Present**	1%	43%

Observations (15092 genes X 14 samples) were partitioned based on detection calls obtained from microarray and RNA-Seq.

### Decomposition of variation in all samples

**Figure 1:**
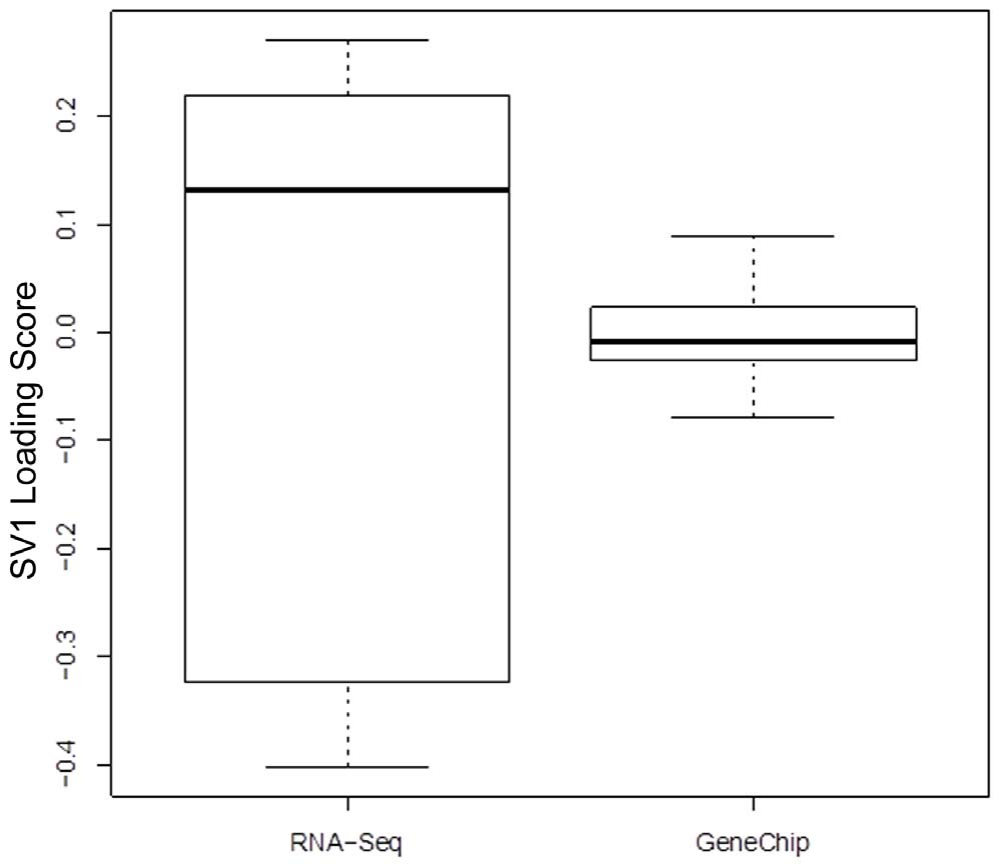
SVA Loadings by Technology. Scores from the first surrogate variable obtained from analysis of the combined RNA-Seq and microarray expression profiles reflect variation among RNA-Seq samples. Unexplained variation in the combined data set was captured using Surrogate Variable Analysis [42], which returns scores and loadings. The distribution of scores from the first surrogate variable is represented in box-and-whisker plots.

One of our objectives is to compare the covariance of the latent variation associated with each technology. The dispersion of the RNA-Seq profiles is larger than that of the microarray profiles (p<5×^10−5^; Figure 1) based on a permutation test for heterogeneity of multivariate dispersions [48]. This larger dispersion for RNA-Seq transcripts might be due to either a larger dispersion associated with latent variation or to a larger dynamic range.

Surrogate Variable Analysis (SVA) [33], [34] provided for decomposition of the variation in a form very similar to that suggested by Eq 1 (see above). We found that the variation associated with transcript levels is substantial and highly statistically significant (Table 4), which justifies extracting the shared variation as we have done.

**Table 4:** SVA ANOVA.

	DF	SSE x 10-3	MSE x 10-3	F	Cum	p
Transcript	7	92	13.0	5.8	0.70	0.001
SV	1	24	24.0	11.0	0.18	0.001
Residuals	7	16	2.3		0.12	
Total	15	130			1.00	

Analysis of variance based on distance matrices [48].

**Figure 2:**
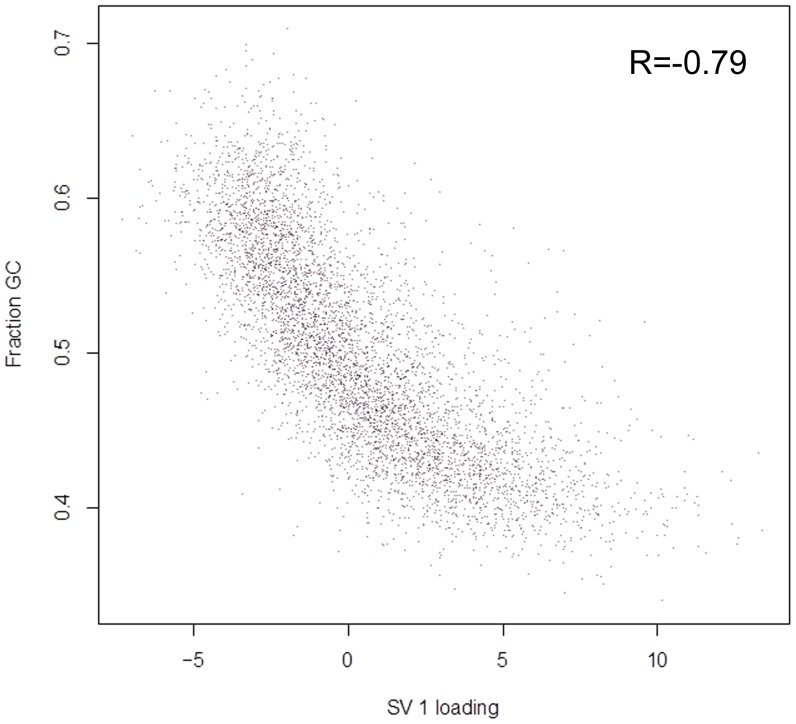
RNA-Seq SVA Loading Reflects GC Content in Transcripts. Loadings from the first surrogate variable, obtained from analysis of the combined RNA-Seq and microarray expression profiles, reflect transcript GC content. Unexplained variation in the combined data set was captured using Surrogate Variable Analysis [42], which returns scores and loadings. Each point represents a gene, located based on its first surrogate variable (SV 1) loading and GC content. Spearman correlation (R) is–0.79.

SVA identified two surrogate variables. The variation associated with the surrogate variables is much larger for the RNA-Seq samples than for the GeneChip samples (Table 5). The greater variation for RNA-Seq is captured by the first surrogate variable (SV1; Table 5), which allows us to evaluate the origin of the larger dispersion. The loadings of SV1 are strongly associated with GC-content (Figure 2), suggesting that the well-known impact of GC content on the number of observed reads within samples [26]–[28] is reflected in the variation in the number of instances of the same read between samples. The second surrogate variable appears to be dominated by one GeneChip sample.

**Table 5:** Surrogate Variable Analysis.

	SV 1	SV 2
Microarray	1.3	7.0
RNA-Seq	56.0	2.1

Surrogate Variable[42] loadings by technology.

It is reasonable to expect that the larger dynamic range of RNA-Seq will result in larger variation. SV1 loadings are also associated with expression level and it is possible to identify genes for which RNA-Seq exhibits, with high statistical significance, an enhanced dynamic range. However, we found no clear evidence of an association of SV1 loadings with nonlinear response in the microarrays. Thus, the general impression is that this enhanced dynamic range makes a smaller contribution to the enhanced dispersion.

**Figure 3:**
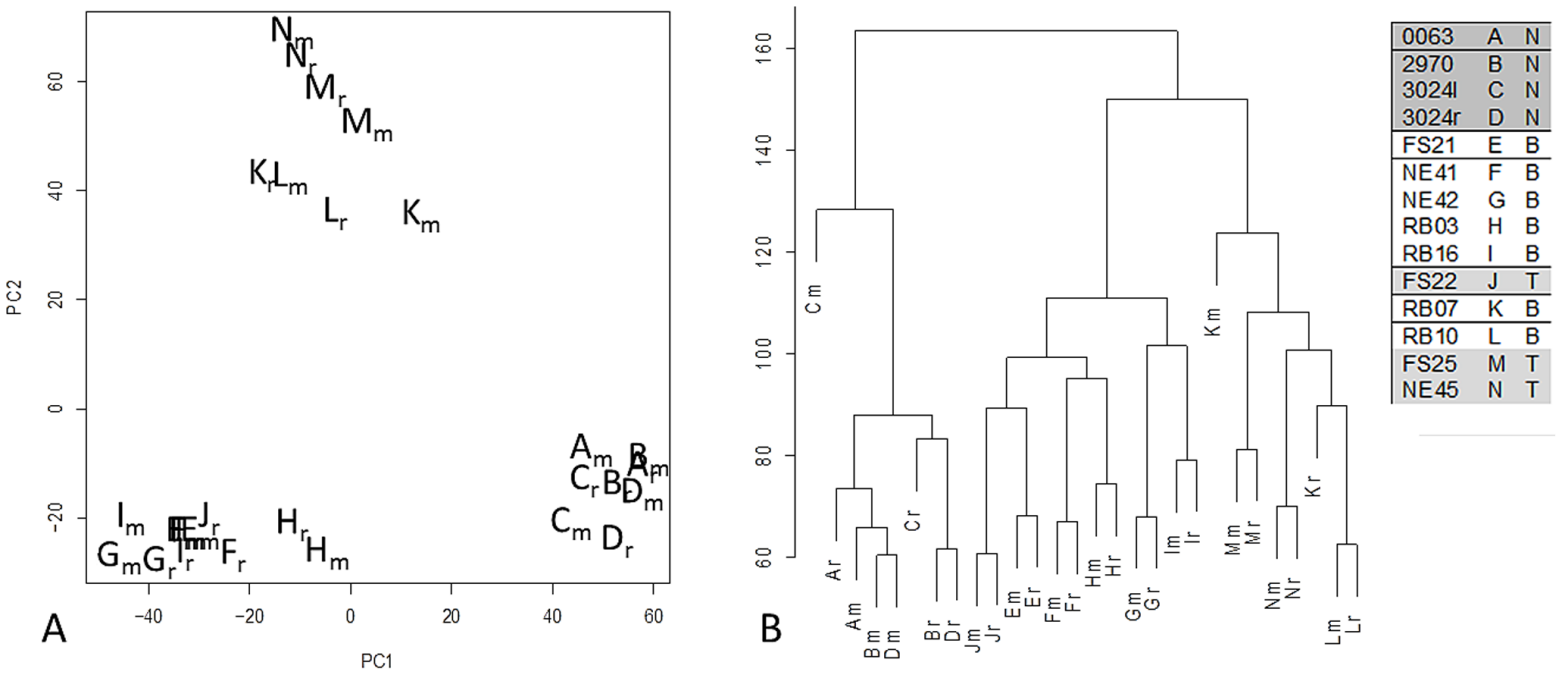
Expression Profiles Cluster by Sample Post-SVA Correction of RNA-Seq. Variation among expression profiles obtained using RNA-Seq is similar to that obtained using microarray after removing contributions of the first surrogate variable [42]. Each letter denotes a sample from a dog having a normal (N), B-cell (B), or T-cell (T) diagnosis as in the legend, with subscript ‘m’ run on the microarray platform and subscript ‘r’ run on the NGS platform. a) Principal component scores b) Hierarchical clustering

Filtration based on the first surrogate variable allowed us to analyze the RNA-Seq and microarray expression profiles simultaneously. More precisely, by row-centering the expression matrices obtained from RNA-Seq and microarray for each sample and by removing variation associated with the first surrogate variable from RNA-Seq data only, the variation associated with the difference between technologies is substantially less than the variation associated with the differences between samples. For example, both principal component analysis (Figure 3a) and hierarchical clustering (Figure 3b) identify three sets of expression profiles, each of which contains both RNA-Seq and microarray profiles for a disjoint set of samples: A–D (normal), E–J, and K–N.

### RNA-Seq and microarray identified similar sets of differentially expressed genes in canine B-cell lymphoma

**Figure 4:**
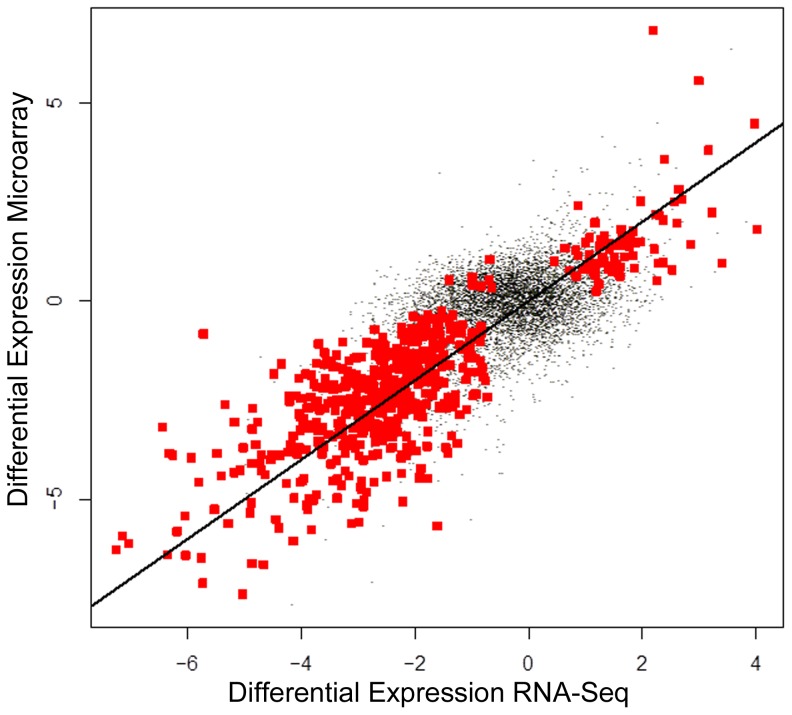
Differential Expression is Concordant Between Technologies. Estimates of differential expression obtained using RNA-Seq and Microarray are correlated. Points represent genes, located based on estimates of differential expression using RNA-Seq and Microarray. Red points represent genes for which FDR<0.2. The line corresponds to perfect agreement between the technologies.

RNA-Seq and microarray observations were each used to compare samples that reflect B-cell lymphoma with normal samples (BvN). Genes (7,296) detected in at least three samples using both methods were used to evaluate differential expression. Both methods detected differences between normal and B-cell lymphoma samples at the limit of the permutation test (p = 1/35) [48]. The F-statistic for the comparison obtained using microarray (5.9) was larger than that obtained using RNA-Seq (3.2) and the q-value method [49] suggests differential expression of 55% and 63% of the genome for RNA-Seq and microarray, respectively. The magnitude of differential expression was highly correlated (Figure 4; ρ = 0.6, p<^10−15^). Identification of differentially expressed genes based on a false discovery rate threshold (0.2) by the two methods were highly associated (Table 6; p<^10−15^, odds ratio = 4.6). The univariate analyses were repeated after filtering latent variation associated with the first surrogate variable, which impacts RNA-Seq variation. The results (not shown) are qualitatively consistent but the p-values obtained after filtering are in general more significant.

**Table 6:** Differentially Expressed Genes by Technology.

		RNA-Seq	
		FALSE	TRUE
Microarray	**FALSE**	4342	269
	**TRUE**	2087	598

Gene counts (out of 7296) were partitioned based on detection of differential expression using RNA-Seq and microarray on sample set BvN.

### Pathway Analysis

Pathway analysis was limited to the B-cell lymphoma sample set and requires a lossy conversion of canine Ensembl gene IDs to human Entrez gene IDs that results in a set of 5,733 input genes (Data File S1). This gene set was used as input into GeneGo and GSEA for each technology. GSEA was performed against two gene set database versions producing four result sets (Data File S2). Enrichment in the RNA-Seq lymphoma phenotype was seen in 21 gene sets at nominal p-value<1% in version 2.5 and 55 gene sets were significantly enriched at nominal p-value<1% in version 3.0. The top 20 gene sets are provided in Table S2. Enrichment in the Microarray lymphoma phenotype was seen in 37 gene sets at nominal p-value<1% in version 2.5 and 128 gene sets at nominal p-value<1% in version 3.0. GeneGo reports were generated for each technology and for the combined analysis using the gene set values from the SVA model producing three result sets (Data File S3).

**Figure 5:**
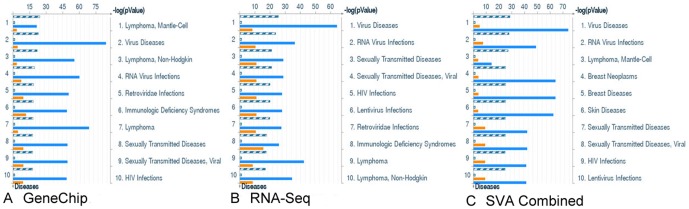
GeneGo Disease Categories. GeneGo Disease categories expression (orange) and topology overlay (blue) show broad support for lymphoma and infectious disease. Similar genes are in grey and common genes (in all samples) are in stripes. a) Disease categories called by microarray dataset b) Disease categories called by RNA-Seq dataset c) Disease categories called by combining the microarray data and RNA-Seq data using SVA.

**Figure 6:**
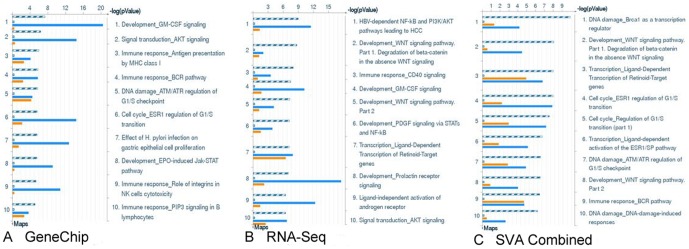
GeneGo Pathway Maps. GeneGO Pathway Maps by expression (orange) and topology overlay (blue). Similar genes are in grey and common genes (in all samples) are in stripes. A) Top ten maps based on microarray platform B) Top ten maps based on RNA-Seq platform C) Top ten maps based on combining the microarray data and RNA-Seq data using SVA.

**Figure 7:**
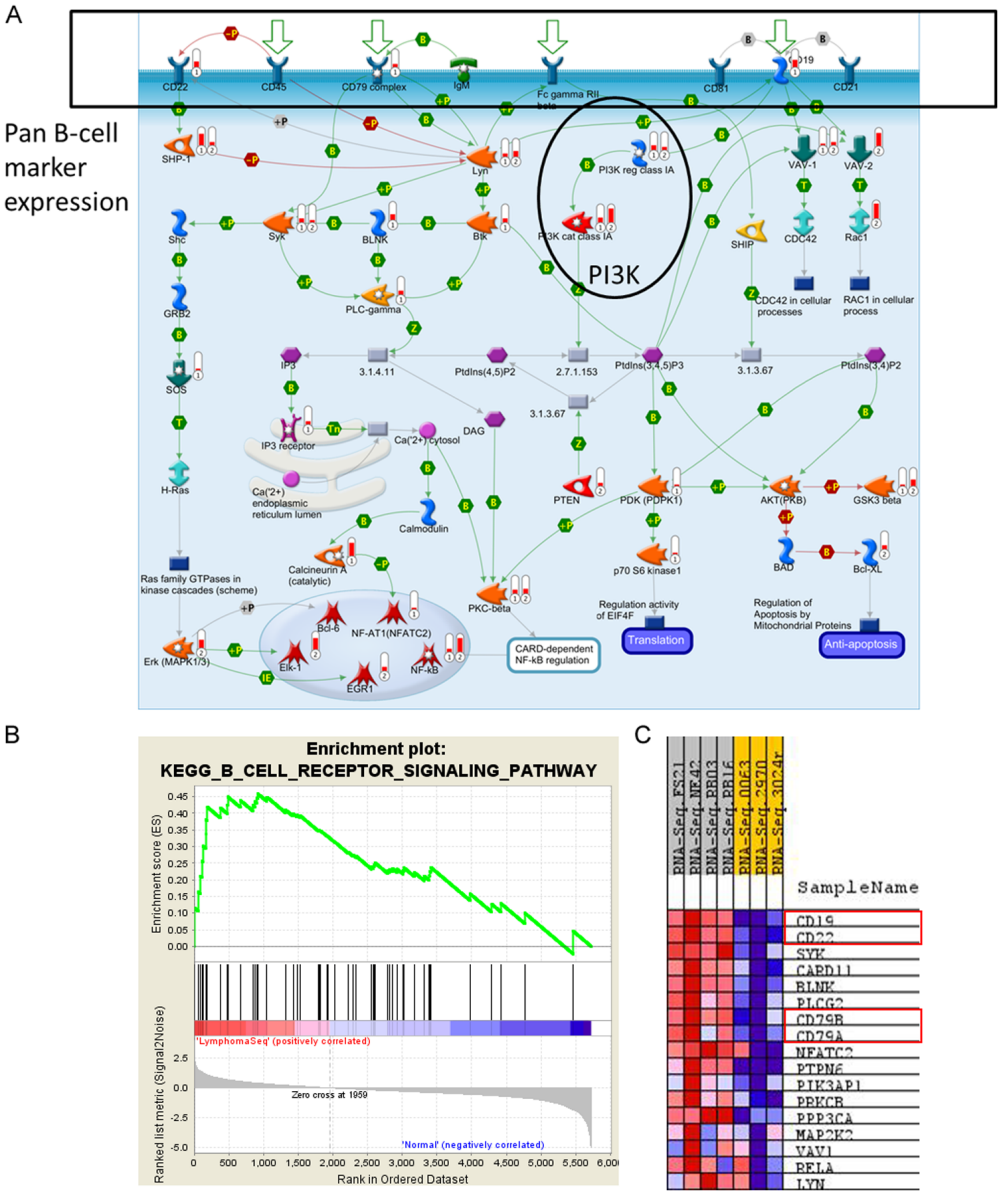
Canine B-Cell Lymphoma Pathway Analysis. a) ‘Immune Response BCR Pathway’ map generated from the combined SVA GeneGo dataset shows pan B-cell markers (CD19, CD22, CD79). The level of indicator 1 at each node denotes upregulated transcriptional expression BvN. The level of indicator 2 at each node denotes topologically discovered transcriptional activators based of the Hidden Nodes Algorithm. b) Supporting gene set enrichments for B cell biology from the top 20 sets by GSEA using the version 2.5 and 3.0 gene set databases (highlighted). The three B-cell maps shown in version 2.5 but not version 3.0 are still represented in the 3.0 enrichment list, but at a lower rank. Namely, BIOCARTA_BCR_PATHWAY is rank 22, ST_B_CELL_ANTIGEN_RECEPTOR is rank 27, and SIG_PIP3_SIGNALING_IN_B_LYMPHOCYTES is rank 33. c) Pan B-cell markers (Gene identifiers boxed in red) enriched in lymphoma in GSEA set ‘KEGG_B_CELL_RECEPTOR_SIGNALING_PATHWAY’, which was chosen because it represents a similar signaling space to the GeneGo map in part a.

GeneGo disease sets support the lymphoma diagnosis and identify immune response activation congruent with B-cell function (Figure 5). Additionally, PI3K, NFkB, MYC, and CBP are pathway elements with maintained expression and high topological significance in the GeneGo pathway maps that appear alongside broader cell cycle and DNA damage repair responses including p53 (Figure 6, Data File S3). Within these pathway maps, the expression of pan B-cell markers appears as expected and is connected topologically to PI3K activity (Figure 7a). GSEA was used as a second method to verify the enriched pathway maps (Figure 7b) and pan B-cell marker enrichment (Figure 7c). GSEA also identifies a Doxorubicin resistance signature in this cohort of samples (Figure 7b). Each of the samples in our study has also been processed through an internal prediction engine that generates a drug treatment report for individuals, and in 3 of 4 individual cases in the B-cell lymphoma cohort a ‘Doxorubicin insensitive’ determination was predicted as well. The basis of this prediction is a curated rules-based determination in medical literature that states the effects of over- or under- expression of a biomarker on drug resistance or sensitivity. The relevant rule in this case states that IF ABCC1>2-fold, THEN Doxorubicin RESISTANT, where ABCC1 is a well-known multi-drug resistance marker [50].

## Discussion

The data model presented here represents both technical and biological sources of variation. Variation shared between RNA-Seq and microarray expression profiles reflects variation present in the RNA samples, while the remaining variation is associated with the technologies. The statistical treatment of the data allowed us to distinguish variation arising from these different sources. Indeed, applying SVA to our data revealed a strong correlation with known technical biases associated with RNA-Seq [26]–[28], [51] and removing the associated variation from the RNA-Seq samples resulted in the two technologies pairing by individual sample.

We used two strategies for evaluating the extent to which RNA-Seq and microarray expression profiles share variation. Perhaps the most common approach to comparing differential expression profiles across platforms is to compare the results of a collection of univariate statistical analyses, one analysis for each gene. Figure 4 gives the general impression that variation is shared genome-wide, that is, the agreement between the two technologies is not limited to a small proportion of highly differentially expression genes. Evaluating the shared variation using univariate analysis is limited in that it does not provide for capturing latent variation associated with each technology. Multivariate analysis attributes 70% of the variation to that present in the RNA samples (Table 4) and rejects (p<0.001) the null hypothesis that variation is shared only by chance.

While SVA provides the basis for a satisfactory interpretation of the combined data set, it is important to address three limitations of our use of SVA. First, we have not attempted to capture differences in dynamic range between RNA-Seq and microarray. It is reasonable to expect that the larger dynamic range of RNA-Seq will result in larger variation. We found latent variation to be highly associated with GC content but, in contrast, found no clear evidence of an association pointing to nonlinear response in the microarrays. Second, the model underlying SVA does not explicitly capture different latent variables for the different technologies. However, Figure 1 and Table 5 give the general impression that we have, in effect, attained such a separation. Third, SVA may capture latent variables that confound design variables [42]. While removing confounding variation is desirable and an important stated goal of SVA, the possibility remains that estimates of differential expression associated with design and latent variables co-vary. We note, however, that pairs of coefficients of surrogate variables associated with the same RNA are uncorrelated so that, in our case, SVA captures latent but not confounding variation. Also, while removing true biological variation is a concern when using an inter-sample correlation matrix-based method to correct confounding variation [52], our approach for applying this procedure only to RNA-Seq data but not microarray data and the resulting concordance between the technologies suggests that we have targeted technical rather than biological variation. This approach should prove valuable for enhancing comparative cross-platform data analysis with NGS and chip-based platforms.

Our statistical procedure produced three latent sample classes. The normal samples are readily identifiable along with two distinct cancer classes. These latter two classes do not correspond to an obvious distinction in breed, age, prior chemotherapy, drug response to the PI3K inhibitor, treatment center, flow cell, lane effects, % tumor cellularity, or B- vs. T- cell lymphoma. An interesting possibility, at least in dogs, is that we have identified a therapeutically relevant lymphoma subtype that is more robust than a B- or T-cell lymphoma diagnosis. The differentially expressed gene list between the latent classes represented a general shift in the importance of cell cycle regulation. Although it is unclear what other characteristics might separate these latent groups, we note that the candidate doxorubicin resistance phenotype is only found within the E-J latent class. A larger sample set would be needed to explore the robustness of these classes and possible distinguishing features.

Additionally, the sequencing depth of these genomes is sufficient to detect genomic features such as differential splicing and alternative transcripts. In general, there are two ways to approach this for an organism with a reference genome and genome annotation: either genome-reference based transcript assembly or reference-annotation based transcript assembly (summarized in [39]). While we have used both methods to generate transcript sets for our data, there are a few issues that complicate the interpretation of the results. First, the canine genome annotation is not very advanced and suffers from numerous challenges (summarized in [53]). Thus, reference-annotation based transcript assembly is currently a less reliable method for novel transcript discovery. Genome-reference based transcript assembly is derived from earlier methods based on Expressed Sequence Tag (EST) libraries that may be more preferable for canines. Transcripts mapped in intergenic or intronic regions can evaluated against existing EST libraries (as in [54]), but here too the canine resources exist but remain sparse [55]. Since we have few samples, from mixed breeds, that are not only mixed (B-cells and T-cells), but also cancerous (which alters the proportion of cell types in the tissue), a proper treatment of how these issues impinge on novel transcript detection and isoform switching is still under investigation.

The two technologies agreed frequently by both detection and differential expression, with slight advantages for both. Detection calls for either RNA-Seq or microarray may reflect Type I or Type II errors so definitive conclusions might be based, for example, on a gold standard and ROC analysis as was done for simulated data in [56]. Instead, we used established methods for calling the presence of expression and found that the results are consistent with published observations that RNA-Seq may provide for more sensitive detection of transcripts than microarrays [54]. This sensitive transcript detection may contribute to the better resolution of cell type specific distinctions observed with RNA-Seq data during GSEA and GeneGo analysis. On the other hand, the higher percentage of differentially expressed genes identified by microarray raises the possibility that the microarray observations have greater statistical power for identifying genome-wide differential expression. We note that this high percentage is not based on filtration with respect to the magnitude of the differential expression, nor does it reflect an effort to limit the false discovery rate. It is reported simply as one summery measure of sensitivity. This might be reflected in GSEA, where microarray data enriches more gene sets at higher NES scores than RNA-Seq. Alternatively, the RNA-Seq platform may capture a wider range of biological variation, thus limiting global differential expression calls. The use of read count data may be more appropriate for assessing differential expression calls unlikely to be contributed due to biological variation [57]. However, normalization methods for read counts (such as in [58]) operate under the assumption that the majority of genes will not be differentially expressed. That assumption does not necessarily hold for our samples, given that even for normal lymphoid samples “at least 65% of transcripts are subtly but significantly different in B and T cells. [59]” The impact of alternative normalization schemes that could impact differential expression calls were not explored in this analysis. Original FPKM values are provided in Data File S4.

All of the downstream analysis tools (GSEA, GeneGo, and our prediction engine) require human information and were designed to operate from microarray platforms. While GeneGo does provide some functionality for canine IDs directly, the topology tool does not and thus we experienced the same lossy conversion between canine and human gene spaces when using GeneGo as with the other tools. However, it is promising that the converted canine data align with many human pathways known to be involved in human lymphoma, discussed briefly below. Although we are limited to seeing transcriptional changes in the current study, the GSEA and GeneGo expression analysis make the most of identifying context for transcriptional expression changes, while the addition of the topology tool helps identify additional areas of activity that likely direct transcriptional changes without necessarily being modified at the transcriptional level.

Numerous signaling pathways implicated in human lymphoma were identified in canine lymphoma by GSEA and GeneGo pathway analysis with topologic discovery. We observed PI3K pathway activity, which was particularly relevant since the origin of these samples was a phase I trial of a novel PI3-K inhibitor in dogs with B and T-cell lymphomas. During the originating dose escalation study, the anti-tumor effects of the drug were found to be largely dose dependent, and thus statistical association between drug response and predicted levels of pathway activation at analytical baseline (prior to onset of therapy) could not be assessed. However, there is anecdotal evidence for effect in subject NE42 with B-cell lymphoma and a high score for inferred PI3K pathway activation (via topological analysis), who achieved a partial response per RECIST criteria (data not shown). The PI3K/Akt/mTOR pathway is known to be deregulated especially in NHL (a primarily B-cell lymphoma in humans) [60], [61], and the response of both this dog and two of our T-cell lymphoma dogs (a much more rare condition in humans) to the high dose of the inhibitor is promising. While we observed little to no topological enrichment at the Akt and mTOR hubs in the canine samples, this could possibly be due to the known loss in data conversion between species or a slightly different biology in the canine disease. However, a larger signaling system has recently been implicated in B-lymphomagenesis that ties together many of the active pathways observed in our canine samples [62]. This includes CD19, PI3K, MYC, and GSK3β. In addition, we observed indications of pathway activity through LYN and SYK, tyrosine kinases known to be involved in B-cell signaling and cross-talk with the PI3K/Akt signaling pathway [61], [63]. We also observed indications of hematopoietic inflammatory response by the immunoproteasome (including the 20S core, 11S regulatory subunit, and PSME1/2 proteasome activators) and NFkB activity. This observation has been made in human lymphoma before, leading to rationale trials of the protease inhibitor Bortezomib with the expectation that it would prevent the degradation of IKBα and thereby block NFkB activity [64]. Altogether, our approach for reducing sample variability and applying human-compatible systems biology tools to canine data supports a thorough sampling of the disease space for B-cell lymphoma.

The identification of a viral disease state in the lymphoma samples (Figure 6) could signal either an infectious component to lymphoma pathogenesis or a more general mobilization of B-cell immune signaling pathways in disease. Numerous infectious organisms have been associated with human lymphoma, including Epstein-Barr virus (EBV), hepatitis B/C (HBV, HCV), HIV, and *Heliobactor pylori* [65], [66], although the role of these infections in making individuals susceptible to or outright causing lymphoma has not been established. Likewise, some correlative evidence for EBV infections in canine lymphoma cases has been surfacing by serologic, PCR, and ISH detection methods [67], [68]. Given the suggestion of viral etiology in our pathway analysis, we aligned our sequencing reads against a series of viral genomes (EBV, HBV, CHV, CAV1, CAV2) with Bowtie and BLAST looking for evidence of viral transcripts within our lymph node aspirates, but were unable to detect any. This is not especially surprising considering that while 64% of U.S. dog's sera samples responded to EBV antibody, no signs of viral transcripts were detected by PCR analysis on blood samples from these dogs [68]. Although persistent infection does not seem to play a role in maintaining lymphoma, the outcomes of the initial infection on lymphoma risk and development remain open for investigation.

GSEA analysis of BvN dogs picks up a highly ranked and robust signature for doxorubicin resistance. We were also able to predict this resistance on an individual level for each dog using an internal prediction engine operating a rules-based method for assigning drug resistance phenotypes. In all but one case (RB03), this engine predicts that the dogs are insensitive to doxorubicin based on extreme expression of the ABCC1 multi-drug resistance transporter (data not shown). The engine also predicts 5-fluorouracil insensitivity (data not shown) in all but one case (NE42), though at a lower confidence than for doxorubicin. Although this study could not address the predictive power of each method, distinguishing the value of expression signature versus biomarker methods on either a cohort or individual level is an important long-term goal for the research in our lab. For human patients, identifying these resistant phenotypes offers a way to avoid undesirable side-effects by reducing the number of agents used in combination regimens for lymphoma treatment. Doxorubicin and 5-fluorouracil had been used together in the F-MACHOP regimen until the late 90s, and doxorubicin continues to be used today in standard therapies for B-cell lymphoma (either CHOP or R-CHOP) [9], [69]. Using data-driven approaches to eliminate agents from therapy when the tumor is insensitive would further reduce treatment risk, such as the cardio-toxicity that can accompany doxorubicin therapy [70].

In summary, we have identified genes differentially expressed between the normal lymph nodes of disease free dogs and dogs diagnosed with B-cell lymphoma using two methods: RNA microarray and RNA-Seq. We report statistical methods for treating the combined data from these technologies as a means for collaboratively supporting biological discovery from the different platforms. Together, the data suggest the elevated expression of genes in the PI3K signaling pathway in B-cell lymphoma, a finding that has also been noted in human lymphoma. Since dogs form tumors spontaneously and have a strong breed predilection towards specific types of cancer, the dog is an excellent translational model for identifying the genetic basis associated with lymphoma.

## Supporting Information

Figure S1 **Sequencing Workflow.** A sequencing workflow based on the Formalwear Suite of Bowtie-Tophat-Cufflinks was employed to examine differential expression from the sequencing data.
(PNG)

Table S1 **Summary of canine subjects.** Age is given in years. Gender is provided as male (M), male-castrated (M/C), female (F), female-spade (F/S). Relapsed dogs received chemotherapy treatment prior to FNA collection for this study. The immunophenotype includes the multi-drug resistant (MDR) status as positive (pos) or negative (neg). Best RESICT (Response Evaluation Criteria in Solid Tumors) score is given of: complete response (CR), partial response (PR), stable disease (SD), or progressive disease (PD).
(PDF)

Table S2 **Top 20 GSEA gene sets.** Supporting gene set enrichments from the top 20 sets by GSEA using the version 2.5 and 3.0 gene set databases (Sets referring to B-cell biology highlighted). The three B-cell maps shown in version 2.5 but not version 3.0 are still represented in the 3.0 enrichment list, but at a lower rank. Namely, BIOCARTA_BCR_PATHWAY is rank 22, ST_B_CELL_ANTIGEN_RECEPTOR is rank 27, and SIG_PIP3_SIGNALING_IN_B_LYMPHOCYTES is rank 33.
(PDF)

Data File S1 **Overlapping Gene Set Used for Pathway Analysis.**
(XLSX)

Data File S2 **GSEA Results Files.**
(ZIP)

Data File S3 **GeneGo Reports.**
(ZIP)

Data File S4 **Pivot Table of Original FPKM Values.**
(ZIP)
