# Supplementary figures and images for: Comparative RNA-Seq and Microarray Analysis of Gene Expression Changes in B-Cell Lymphomas of Canis familiaris
Source: PLoS One. 2013 Apr 4;8(4):e61088. doi: 10.1371/journal.pone.0061088 (PMC3617154; doi:10.1371/journal.pone.0061088)

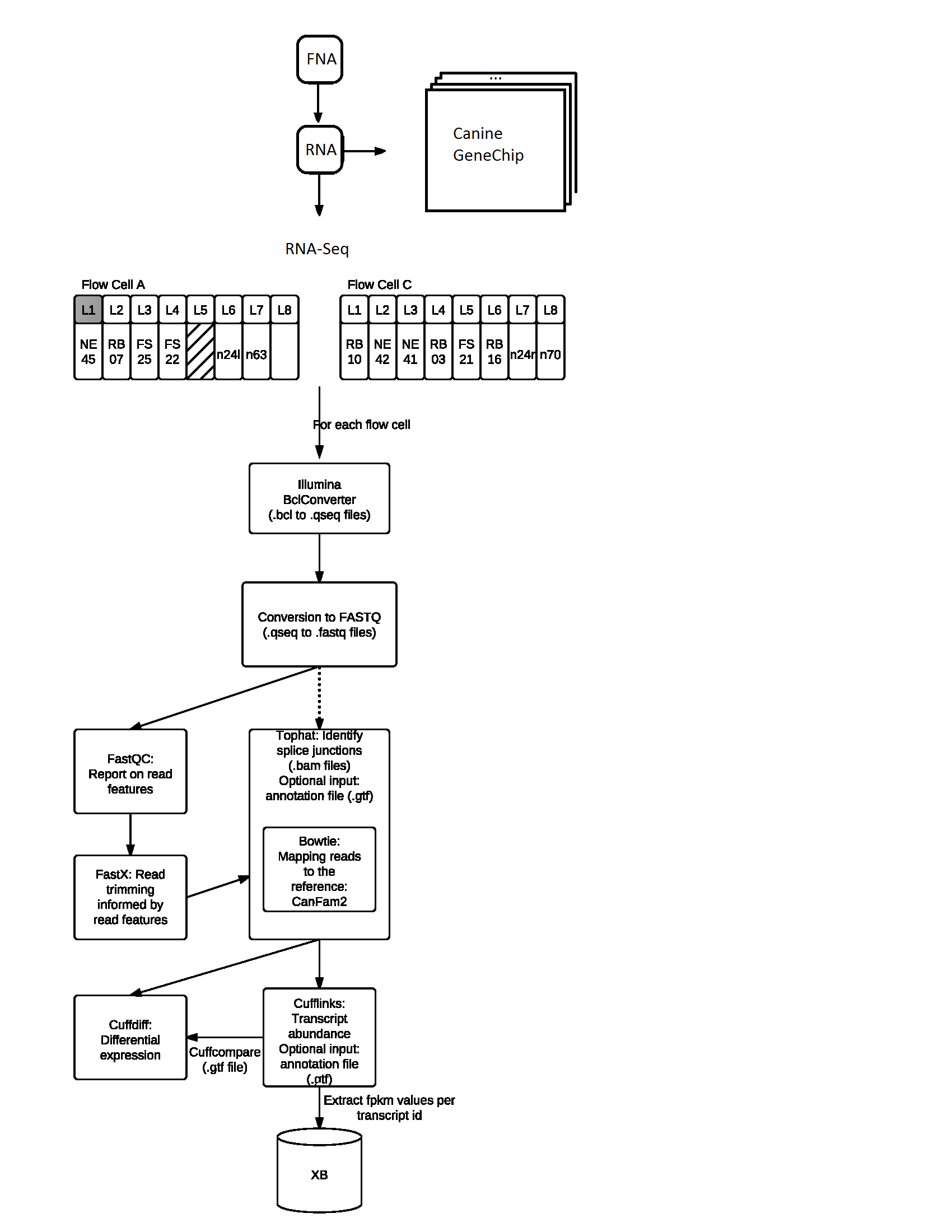

Supplement: Figure S1 — Sequencing Workflow. A sequencing workflow based on the Formalwear Suite of Bowtie-Tophat-Cufflinks was employed to examine differential expression from the sequencing data. (PNG) [file pone.0061088.s001.png]
