# Supplementary material for: Comparative RNA-Seq and Microarray Analysis of Gene Expression Changes in B-Cell Lymphomas of Canis familiaris
Source: PLoS One. 2013 Apr 4;8(4):e61088. doi: 10.1371/journal.pone.0061088 (PMC3617154; doi:10.1371/journal.pone.0061088)
Supplement: Table S1 — Summary of canine subjects. Age is given in years. Gender is provided as male (M), male-castrated (M/C), female (F), female-spade (F/S). Relapsed dogs received chemotherapy treatment prior to FNA collection for this study. The immunophenotype includes the multi-drug resistant (MDR) status as positive (pos) or negative (neg). Best RESICT (Response Evaluation Criteria in Solid Tumors) score is given of: complete response (CR), partial response (PR), stable disease (SD), or progressive disease (PD). (PDF) [file pone.0061088.s002.pdf]

Supplementary Table 1. Summary of canine subjects

Responders	Age	Gender	Breed	Stage	Recurrence	Immuno-phenotype	Best RECIST Response
RB-07	5.75	F/S	Boxer	IIIa	Relapsed	B-cell, MDR Neg	PR
NE-42	9	M/C	Labrador Retriever	Iva	Naïve	B-cell, MDR Neg	PR
NE-41	9	M/C	Boxer/Pitt Bull Terr. Mix	IVa	Naïve	B-cell, MDR Neg	PR
NE-45	9	M/C	Golden Retriever	IIIa	Naïve	T-cell, MDR Pos	PR
FS-25	7	F/S	Boxer	IIIa	Relapsed	T-cell, MDR Neg	SD

Non-responders	Age	Gender	Breed	Stage	Recurrence	Immuno-phenotype	Best RECIST response
RB-10	5	M	Boxer	Va	Naïve	B-cell, MDR Neg	SD
RB-03	12.5	M/C	Pointer	IIIa	Naïve	B-cell, MDR Neg	SD
FS-21	4	F/S	Pitt Bull Terrier/Lab Retriever Mix	IIIa	Relapsed	B-cell, MDR Neg	SD
RB-16	5	F/S	Chesapeake Bay Retriever	IVa	Naïve	B-cell, MDR Neg	SD
FS-22	11	F/S	Beagle	IIIa	Relapsed	T-cell, MDR Neg	SD
