# Supplementary material for: Comparative RNA-Seq and Microarray Analysis of Gene Expression Changes in B-Cell Lymphomas of Canis familiaris
Source: PLoS One. 2013 Apr 4;8(4):e61088. doi: 10.1371/journal.pone.0061088 (PMC3617154; doi:10.1371/journal.pone.0061088)
Supplement: Data File S2 — GSEA Results Files. (ZIP) [file pone.0061088.s005.zip › RNA-Seq/gsea_report_for_LymphomaSeq_v2.html]

Report for LymphomaSeq 1334779864086 [GSEA]

| GS  follow link to MSigDB | GS DETAILS | SIZE | ES | NES | NOM p-val | FDR q-val | FWER p-val | RANK AT MAX | LEADING EDGE || 1 | SIG\_BCR\_SIGNALING\_PATHWAY | Details ... | 30 | 0.59 | 2.23 | 0.000 | 0.010 | 0.009 | 923 | tags=37%, list=16%, signal=43% |
| 2 | HSA04662\_B\_CELL\_RECEPTOR\_SIGNALING\_PATHWAY | Details ... | 39 | 0.53 | 2.17 | 0.000 | 0.011 | 0.020 | 480 | tags=26%, list=8%, signal=28% |
| 3 | SHEPARD\_GENES\_COMMON\_BW\_CB\_MO | Details ... | 21 | 0.64 | 2.17 | 0.000 | 0.007 | 0.020 | 858 | tags=43%, list=15%, signal=50% |
| 4 | MANALO\_HYPOXIA\_DN | Details ... | 45 | 0.51 | 2.08 | 0.000 | 0.019 | 0.069 | 804 | tags=31%, list=14%, signal=36% |
| 5 | CELL\_CYCLE | Details ... | 40 | 0.50 | 2.07 | 0.000 | 0.015 | 0.070 | 1280 | tags=48%, list=22%, signal=61% |
| 6 | CROONQUIST\_IL6\_STARVE\_UP | Details ... | 15 | 0.65 | 2.05 | 0.000 | 0.015 | 0.083 | 931 | tags=53%, list=16%, signal=64% |
| 7 | ZHAN\_MM\_CD138\_PR\_VS\_REST | Details ... | 16 | 0.62 | 2.05 | 0.013 | 0.013 | 0.085 | 1600 | tags=88%, list=28%, signal=121% |
| 8 | BRENTANI\_REPAIR | Details ... | 24 | 0.57 | 2.03 | 0.000 | 0.014 | 0.103 | 1259 | tags=54%, list=22%, signal=69% |
| 9 | BRCA1\_OVEREXP\_DN | Details ... | 50 | 0.47 | 2.02 | 0.000 | 0.014 | 0.111 | 1007 | tags=42%, list=18%, signal=51% |
| 10 | P21\_P53\_MIDDLE\_DN | Details ... | 15 | 0.62 | 1.95 | 0.000 | 0.022 | 0.185 | 1194 | tags=53%, list=21%, signal=67% |
| 11 | DOX\_RESIST\_GASTRIC\_UP | Details ... | 19 | 0.58 | 1.94 | 0.000 | 0.023 | 0.209 | 1281 | tags=58%, list=22%, signal=74% |
| 12 | P21\_P53\_ANY\_DN | Details ... | 22 | 0.51 | 1.82 | 0.000 | 0.054 | 0.459 | 1194 | tags=50%, list=21%, signal=63% |
| 13 | DNA\_REPLICATION\_REACTOME | Details ... | 24 | 0.50 | 1.82 | 0.006 | 0.051 | 0.474 | 1049 | tags=42%, list=18%, signal=51% |
| 14 | BCRPATHWAY | Details ... | 23 | 0.49 | 1.77 | 0.007 | 0.065 | 0.582 | 380 | tags=22%, list=7%, signal=23% |
| 15 | ADIP\_DIFF\_CLUSTER5 | Details ... | 16 | 0.54 | 1.76 | 0.009 | 0.064 | 0.602 | 982 | tags=56%, list=17%, signal=68% |
| 16 | LI\_FETAL\_VS\_WT\_KIDNEY\_DN | Details ... | 69 | 0.39 | 1.75 | 0.018 | 0.068 | 0.635 | 1052 | tags=35%, list=18%, signal=42% |
| 17 | CANTHARIDIN\_DN | Details ... | 21 | 0.50 | 1.75 | 0.006 | 0.064 | 0.638 | 1302 | tags=43%, list=23%, signal=55% |
| 18 | RIBOSOMAL\_PROTEINS | Details ... | 22 | 0.48 | 1.73 | 0.011 | 0.069 | 0.683 | 2988 | tags=100%, list=52%, signal=208% |
| 19 | SIG\_PIP3\_SIGNALING\_IN\_B\_LYMPHOCYTES | Details ... | 19 | 0.51 | 1.73 | 0.010 | 0.066 | 0.683 | 1050 | tags=37%, list=18%, signal=45% |
| 20 | ST\_B\_CELL\_ANTIGEN\_RECEPTOR | Details ... | 27 | 0.47 | 1.71 | 0.006 | 0.072 | 0.730 | 167 | tags=15%, list=3%, signal=15% |
| 21 | SHEPARD\_CRASH\_AND\_BURN\_MUT\_VS\_WT\_DN |  | 47 | 0.39 | 1.68 | 0.000 | 0.080 | 0.784 | 1213 | tags=38%, list=21%, signal=48% |
| 22 | P21\_ANY\_DN |  | 19 | 0.48 | 1.67 | 0.014 | 0.083 | 0.811 | 1321 | tags=53%, list=23%, signal=68% |
| 23 | LEE\_TCELLS3\_UP |  | 35 | 0.41 | 1.66 | 0.008 | 0.081 | 0.816 | 1049 | tags=46%, list=18%, signal=56% |
| 24 | IDX\_TSA\_UP\_CLUSTER3 |  | 43 | 0.39 | 1.66 | 0.000 | 0.079 | 0.823 | 1321 | tags=49%, list=23%, signal=63% |
| 25 | ADIP\_DIFF\_CLUSTER4 |  | 17 | 0.49 | 1.60 | 0.036 | 0.111 | 0.915 | 1191 | tags=41%, list=21%, signal=52% |
| 26 | HSA04110\_CELL\_CYCLE |  | 59 | 0.36 | 1.59 | 0.000 | 0.117 | 0.933 | 1280 | tags=41%, list=22%, signal=52% |
| 27 | REN\_E2F1\_TARGETS |  | 25 | 0.42 | 1.58 | 0.024 | 0.116 | 0.937 | 506 | tags=24%, list=9%, signal=26% |
| 28 | REOVIRUS\_HEK293\_DN |  | 94 | 0.31 | 1.51 | 0.000 | 0.170 | 0.986 | 683 | tags=27%, list=12%, signal=30% |
| 29 | HSA00790\_FOLATE\_BIOSYNTHESIS |  | 18 | 0.44 | 1.50 | 0.064 | 0.178 | 0.989 | 796 | tags=33%, list=14%, signal=39% |
| 30 | HSA00240\_PYRIMIDINE\_METABOLISM |  | 45 | 0.36 | 1.48 | 0.022 | 0.192 | 0.996 | 243 | tags=16%, list=4%, signal=16% |
| 31 | HDACI\_COLON\_CLUSTER6 |  | 18 | 0.44 | 1.47 | 0.050 | 0.193 | 0.997 | 708 | tags=28%, list=12%, signal=32% |
| 32 | BRENTANI\_TRANSCRIPTION\_FACTORS |  | 23 | 0.41 | 1.47 | 0.048 | 0.193 | 0.997 | 919 | tags=48%, list=16%, signal=57% |
| 33 | H2O2\_CSBDIFF\_C2 |  | 17 | 0.43 | 1.46 | 0.066 | 0.195 | 0.997 | 875 | tags=35%, list=15%, signal=42% |
| 34 | CMV\_IE86\_UP |  | 29 | 0.38 | 1.45 | 0.051 | 0.204 | 0.999 | 758 | tags=28%, list=13%, signal=32% |
| 35 | DNMT1\_KO\_UP |  | 25 | 0.37 | 1.39 | 0.082 | 0.270 | 1.000 | 923 | tags=40%, list=16%, signal=47% |
| 36 | SA\_B\_CELL\_RECEPTOR\_COMPLEXES |  | 18 | 0.41 | 1.39 | 0.084 | 0.263 | 1.000 | 1050 | tags=28%, list=18%, signal=34% |
| 37 | PYRIMIDINE\_METABOLISM |  | 24 | 0.39 | 1.39 | 0.062 | 0.257 | 1.000 | 243 | tags=21%, list=4%, signal=22% |
| 38 | DNA\_DAMAGE\_SIGNALING |  | 51 | 0.32 | 1.38 | 0.022 | 0.259 | 1.000 | 858 | tags=25%, list=15%, signal=30% |
| 39 | BCNU\_GLIOMA\_MGMT\_48HRS\_DN |  | 32 | 0.34 | 1.37 | 0.081 | 0.270 | 1.000 | 723 | tags=34%, list=13%, signal=39% |
| 40 | AGUIRRE\_PANCREAS\_CHR22 |  | 21 | 0.40 | 1.34 | 0.074 | 0.301 | 1.000 | 1287 | tags=57%, list=22%, signal=73% |
| 41 | G1\_TO\_S\_CELL\_CYCLE\_REACTOME |  | 32 | 0.32 | 1.34 | 0.072 | 0.296 | 1.000 | 1280 | tags=44%, list=22%, signal=56% |
| 42 | FALT\_BCLL\_UP |  | 19 | 0.39 | 1.32 | 0.128 | 0.326 | 1.000 | 1500 | tags=74%, list=26%, signal=100% |
| 43 | WNT\_SIGNALING |  | 26 | 0.36 | 1.32 | 0.096 | 0.319 | 1.000 | 520 | tags=27%, list=9%, signal=29% |
| 44 | IL2RBPATHWAY |  | 19 | 0.38 | 1.30 | 0.126 | 0.337 | 1.000 | 480 | tags=21%, list=8%, signal=23% |
| 45 | UVB\_NHEK2\_DN |  | 39 | 0.32 | 1.29 | 0.088 | 0.348 | 1.000 | 754 | tags=26%, list=13%, signal=29% |
| 46 | UVC\_TTD\_ALL\_UP |  | 24 | 0.35 | 1.29 | 0.119 | 0.346 | 1.000 | 702 | tags=29%, list=12%, signal=33% |
| 47 | BRENTANI\_CELL\_CYCLE |  | 40 | 0.32 | 1.28 | 0.104 | 0.361 | 1.000 | 1212 | tags=33%, list=21%, signal=41% |
| 48 | MRNA\_PROCESSING\_REACTOME |  | 50 | 0.29 | 1.26 | 0.075 | 0.380 | 1.000 | 1392 | tags=46%, list=24%, signal=60% |
| 49 | SERUM\_FIBROBLAST\_CELLCYCLE |  | 58 | 0.28 | 1.25 | 0.070 | 0.406 | 1.000 | 1338 | tags=38%, list=23%, signal=49% |
| 50 | HSA03010\_RIBOSOME |  | 18 | 0.36 | 1.23 | 0.220 | 0.427 | 1.000 | 3654 | tags=100%, list=64%, signal=276% |
| 51 | PEART\_HISTONE\_DN |  | 39 | 0.30 | 1.20 | 0.164 | 0.475 | 1.000 | 1132 | tags=41%, list=20%, signal=51% |
| 52 | HSA00562\_INOSITOL\_PHOSPHATE\_METABOLISM |  | 22 | 0.34 | 1.19 | 0.224 | 0.494 | 1.000 | 246 | tags=18%, list=4%, signal=19% |
| 53 | GALE\_FLT3ANDAPL\_UP |  | 23 | 0.32 | 1.18 | 0.202 | 0.514 | 1.000 | 1007 | tags=30%, list=18%, signal=37% |
| 54 | GLYCINE\_SERINE\_AND\_THREONINE\_METABOLISM |  | 15 | 0.37 | 1.18 | 0.247 | 0.506 | 1.000 | 623 | tags=33%, list=11%, signal=37% |
| 55 | UVB\_SCC\_DN |  | 50 | 0.26 | 1.16 | 0.172 | 0.544 | 1.000 | 666 | tags=24%, list=12%, signal=27% |
| 56 | BRCA2\_BRCA1\_UP |  | 24 | 0.32 | 1.16 | 0.228 | 0.541 | 1.000 | 1323 | tags=42%, list=23%, signal=54% |
| 57 | HSA04310\_WNT\_SIGNALING\_PATHWAY |  | 50 | 0.27 | 1.16 | 0.102 | 0.535 | 1.000 | 655 | tags=22%, list=11%, signal=25% |
| 58 | CELL\_CYCLE\_KEGG |  | 43 | 0.27 | 1.15 | 0.192 | 0.539 | 1.000 | 1212 | tags=42%, list=21%, signal=53% |
| 59 | UVC\_TTD\_4HR\_UP |  | 22 | 0.34 | 1.14 | 0.235 | 0.544 | 1.000 | 702 | tags=27%, list=12%, signal=31% |
| 60 | HSA00970\_AMINOACYL\_TRNA\_BIOSYNTHESIS |  | 27 | 0.31 | 1.13 | 0.280 | 0.576 | 1.000 | 1189 | tags=37%, list=21%, signal=47% |
| 61 | MYC\_ONCOGENIC\_SIGNATURE |  | 71 | 0.24 | 1.13 | 0.181 | 0.573 | 1.000 | 949 | tags=34%, list=17%, signal=40% |
| 62 | ROTH\_HTERT\_DIFF |  | 16 | 0.33 | 1.10 | 0.314 | 0.620 | 1.000 | 630 | tags=19%, list=11%, signal=21% |
| 63 | HSA04930\_TYPE\_II\_DIABETES\_MELLITUS |  | 15 | 0.35 | 1.10 | 0.342 | 0.623 | 1.000 | 297 | tags=20%, list=5%, signal=21% |
| 64 | HSA04070\_PHOSPHATIDYLINOSITOL\_SIGNALING\_SYSTEM |  | 32 | 0.29 | 1.10 | 0.281 | 0.621 | 1.000 | 246 | tags=16%, list=4%, signal=16% |
| 65 | FETAL\_LIVER\_ENRICHED\_TRANSCRIPTION\_FACTORS |  | 36 | 0.27 | 1.10 | 0.312 | 0.613 | 1.000 | 900 | tags=36%, list=16%, signal=43% |
| 66 | COCAINE\_BRAIN\_5D\_UP |  | 25 | 0.30 | 1.09 | 0.333 | 0.626 | 1.000 | 507 | tags=20%, list=9%, signal=22% |
| 67 | FMLPPATHWAY |  | 19 | 0.32 | 1.08 | 0.327 | 0.633 | 1.000 | 919 | tags=26%, list=16%, signal=31% |
| 68 | ZHANG\_EFT\_EWSFLI1\_UP |  | 30 | 0.28 | 1.08 | 0.327 | 0.625 | 1.000 | 928 | tags=33%, list=16%, signal=40% |
| 69 | PENG\_RAPAMYCIN\_UP |  | 65 | 0.24 | 1.08 | 0.317 | 0.625 | 1.000 | 811 | tags=31%, list=14%, signal=35% |
| 70 | PASSERINI\_APOPTOSIS |  | 18 | 0.32 | 1.07 | 0.343 | 0.623 | 1.000 | 497 | tags=22%, list=9%, signal=24% |
| 71 | HSA04910\_INSULIN\_SIGNALING\_PATHWAY |  | 55 | 0.24 | 1.07 | 0.352 | 0.623 | 1.000 | 877 | tags=24%, list=15%, signal=28% |
| 72 | UVB\_NHEK1\_DN |  | 129 | 0.20 | 1.06 | 0.227 | 0.656 | 1.000 | 661 | tags=18%, list=12%, signal=20% |
| 73 | TCELL\_ANERGIC\_UP |  | 28 | 0.28 | 1.05 | 0.368 | 0.661 | 1.000 | 911 | tags=36%, list=16%, signal=42% |
| 74 | UVC\_XPCS\_ALL\_UP |  | 25 | 0.28 | 1.04 | 0.347 | 0.672 | 1.000 | 1080 | tags=40%, list=19%, signal=49% |
| 75 | AGUIRRE\_PANCREAS\_CHR7 |  | 17 | 0.32 | 1.02 | 0.377 | 0.723 | 1.000 | 117 | tags=18%, list=2%, signal=18% |
| 76 | JAIN\_NEMO\_DIFF |  | 36 | 0.26 | 1.01 | 0.427 | 0.733 | 1.000 | 836 | tags=22%, list=15%, signal=26% |
| 77 | CANCER\_UNDIFFERENTIATED\_META\_UP |  | 37 | 0.25 | 1.01 | 0.496 | 0.730 | 1.000 | 179 | tags=11%, list=3%, signal=11% |
| 78 | ZHAN\_MMPC\_EARLYVS |  | 23 | 0.28 | 0.99 | 0.444 | 0.784 | 1.000 | 1299 | tags=39%, list=23%, signal=50% |
| 79 | G13\_SIGNALING\_PATHWAY |  | 18 | 0.29 | 0.99 | 0.452 | 0.777 | 1.000 | 376 | tags=11%, list=7%, signal=12% |
| 80 | BYSTROM\_IL5\_DN |  | 39 | 0.24 | 0.98 | 0.480 | 0.791 | 1.000 | 430 | tags=13%, list=8%, signal=14% |
| 81 | HSA00010\_GLYCOLYSIS\_AND\_GLUCONEOGENESIS |  | 16 | 0.30 | 0.98 | 0.498 | 0.791 | 1.000 | 429 | tags=25%, list=7%, signal=27% |
| 82 | ZHAN\_MULTIPLE\_MYELOMA\_SUBCLASSES\_DIFF |  | 16 | 0.30 | 0.97 | 0.476 | 0.800 | 1.000 | 789 | tags=31%, list=14%, signal=36% |
| 83 | BREASTCA\_THREE\_CLASSES |  | 16 | 0.30 | 0.96 | 0.520 | 0.801 | 1.000 | 1340 | tags=44%, list=23%, signal=57% |
| 84 | TOLLPATHWAY |  | 18 | 0.30 | 0.96 | 0.512 | 0.798 | 1.000 | 70 | tags=11%, list=1%, signal=11% |
| 85 | HUMAN\_TISSUE\_TESTIS |  | 19 | 0.29 | 0.96 | 0.557 | 0.795 | 1.000 | 665 | tags=26%, list=12%, signal=30% |
| 86 | UVC\_XPCS\_8HR\_UP |  | 23 | 0.27 | 0.95 | 0.595 | 0.813 | 1.000 | 609 | tags=26%, list=11%, signal=29% |
| 87 | PENG\_LEUCINE\_DN |  | 79 | 0.19 | 0.94 | 0.605 | 0.819 | 1.000 | 272 | tags=6%, list=5%, signal=7% |
| 88 | KERATINOCYTEPATHWAY |  | 27 | 0.26 | 0.94 | 0.592 | 0.820 | 1.000 | 181 | tags=11%, list=3%, signal=11% |
| 89 | ZHAN\_TONSIL\_PCBC |  | 21 | 0.27 | 0.94 | 0.519 | 0.813 | 1.000 | 1281 | tags=38%, list=22%, signal=49% |
| 90 | WANG\_MLL\_CBP\_VS\_GMP\_DN |  | 23 | 0.26 | 0.90 | 0.600 | 0.892 | 1.000 | 984 | tags=39%, list=17%, signal=47% |
| 91 | PENG\_GLUTAMINE\_DN |  | 131 | 0.19 | 0.90 | 0.800 | 0.904 | 1.000 | 1443 | tags=34%, list=25%, signal=45% |
| 92 | SCHUMACHER\_MYC\_UP |  | 22 | 0.25 | 0.88 | 0.632 | 0.925 | 1.000 | 1366 | tags=45%, list=24%, signal=59% |
| 93 | RNA\_TRANSCRIPTION\_REACTOME |  | 19 | 0.27 | 0.88 | 0.628 | 0.923 | 1.000 | 602 | tags=16%, list=11%, signal=18% |
| 94 | AGUIRRE\_PANCREAS\_CHR8 |  | 16 | 0.27 | 0.88 | 0.626 | 0.915 | 1.000 | 1141 | tags=31%, list=20%, signal=39% |
| 95 | AMINOACYL\_TRNA\_BIOSYNTHESIS |  | 16 | 0.28 | 0.87 | 0.615 | 0.919 | 1.000 | 15 | tags=6%, list=0%, signal=6% |
| 96 | FETAL\_LIVER\_VS\_ADULT\_LIVER\_GNF2 |  | 16 | 0.26 | 0.87 | 0.671 | 0.916 | 1.000 | 670 | tags=25%, list=12%, signal=28% |
| 97 | HSA04720\_LONG\_TERM\_POTENTIATION |  | 26 | 0.23 | 0.87 | 0.695 | 0.909 | 1.000 | 877 | tags=31%, list=15%, signal=36% |
| 98 | HSA00251\_GLUTAMATE\_METABOLISM |  | 15 | 0.28 | 0.87 | 0.670 | 0.902 | 1.000 | 148 | tags=13%, list=3%, signal=14% |
| 99 | BRENTANI\_DEATH |  | 28 | 0.23 | 0.86 | 0.706 | 0.922 | 1.000 | 497 | tags=18%, list=9%, signal=19% |
| 100 | ST\_G\_ALPHA\_I\_PATHWAY |  | 16 | 0.26 | 0.85 | 0.713 | 0.935 | 1.000 | 25 | tags=6%, list=0%, signal=6% |
| 101 | IL2PATHWAY |  | 15 | 0.26 | 0.84 | 0.707 | 0.943 | 1.000 | 425 | tags=13%, list=7%, signal=14% |
| 102 | SIG\_PIP3\_SIGNALING\_IN\_CARDIAC\_MYOCTES |  | 30 | 0.21 | 0.84 | 0.735 | 0.936 | 1.000 | 802 | tags=20%, list=14%, signal=23% |
| 103 | HSA04012\_ERBB\_SIGNALING\_PATHWAY |  | 40 | 0.20 | 0.83 | 0.803 | 0.936 | 1.000 | 517 | tags=15%, list=9%, signal=16% |
| 104 | CELL\_CYCLE\_ARREST |  | 15 | 0.26 | 0.82 | 0.726 | 0.944 | 1.000 | 565 | tags=20%, list=10%, signal=22% |
| 105 | HSA04150\_MTOR\_SIGNALING\_PATHWAY |  | 23 | 0.23 | 0.82 | 0.729 | 0.939 | 1.000 | 785 | tags=22%, list=14%, signal=25% |
| 106 | UVC\_HIGH\_D3\_DN |  | 27 | 0.21 | 0.81 | 0.824 | 0.947 | 1.000 | 860 | tags=22%, list=15%, signal=26% |
| 107 | YU\_CMYC\_DN |  | 22 | 0.23 | 0.80 | 0.794 | 0.960 | 1.000 | 459 | tags=18%, list=8%, signal=20% |
| 108 | ST\_PHOSPHOINOSITIDE\_3\_KINASE\_PATHWAY |  | 17 | 0.25 | 0.79 | 0.749 | 0.964 | 1.000 | 4292 | tags=100%, list=75%, signal=399% |
| 109 | HSC\_STHSC\_FETAL |  | 15 | 0.24 | 0.78 | 0.784 | 0.976 | 1.000 | 942 | tags=27%, list=16%, signal=32% |
| 110 | NFATPATHWAY |  | 18 | 0.23 | 0.77 | 0.822 | 0.975 | 1.000 | 4420 | tags=100%, list=77%, signal=438% |
| 111 | HSC\_STHSC\_SHARED |  | 15 | 0.24 | 0.76 | 0.832 | 0.992 | 1.000 | 942 | tags=27%, list=16%, signal=32% |
| 112 | BLEO\_MOUSE\_LYMPH\_HIGH\_24HRS\_DN |  | 18 | 0.23 | 0.75 | 0.806 | 0.996 | 1.000 | 315 | tags=11%, list=6%, signal=12% |
| 113 | OXSTRESS\_RPETHREE\_DN |  | 15 | 0.24 | 0.74 | 0.819 | 0.998 | 1.000 | 452 | tags=13%, list=8%, signal=14% |
| 114 | TPOPATHWAY |  | 15 | 0.23 | 0.73 | 0.841 | 1.000 | 1.000 | 4420 | tags=100%, list=77%, signal=438% |
| 115 | HSA05223\_NON\_SMALL\_CELL\_LUNG\_CANCER |  | 28 | 0.19 | 0.72 | 0.896 | 1.000 | 1.000 | 877 | tags=21%, list=15%, signal=25% |
| 116 | HSA00650\_BUTANOATE\_METABOLISM |  | 24 | 0.19 | 0.68 | 0.933 | 1.000 | 1.000 | 588 | tags=13%, list=10%, signal=14% |
| 117 | HSA04115\_P53\_SIGNALING\_PATHWAY |  | 37 | 0.17 | 0.68 | 0.962 | 1.000 | 1.000 | 646 | tags=14%, list=11%, signal=15% |
| 118 | MAPK\_CASCADE |  | 17 | 0.20 | 0.67 | 0.896 | 1.000 | 1.000 | 910 | tags=24%, list=16%, signal=28% |
| 119 | YU\_CMYC\_UP |  | 20 | 0.20 | 0.65 | 0.917 | 1.000 | 1.000 | 1194 | tags=35%, list=21%, signal=44% |
| 120 | CELL\_CYCLE\_CHECKPOINT |  | 15 | 0.21 | 0.65 | 0.916 | 1.000 | 1.000 | 1206 | tags=40%, list=21%, signal=51% |
| 121 | MAPKPATHWAY |  | 46 | 0.15 | 0.65 | 1.000 | 1.000 | 1.000 | 919 | tags=17%, list=16%, signal=21% |
| 122 | STRESS\_GENOTOXIC\_SPECIFIC\_DN |  | 17 | 0.20 | 0.64 | 0.934 | 1.000 | 1.000 | 466 | tags=18%, list=8%, signal=19% |
| 123 | IFN\_ALPHA\_UP |  | 16 | 0.20 | 0.64 | 0.913 | 1.000 | 1.000 | 847 | tags=25%, list=15%, signal=29% |
| 124 | SRC\_ONCOGENIC\_SIGNATURE |  | 28 | 0.16 | 0.58 | 0.967 | 1.000 | 1.000 | 656 | tags=14%, list=11%, signal=16% |
| 125 | HSA00310\_LYSINE\_DEGRADATION |  | 27 | 0.15 | 0.57 | 0.994 | 1.000 | 1.000 | 266 | tags=7%, list=5%, signal=8% |
| 126 | AGUIRRE\_PANCREAS\_CHR17 |  | 29 | 0.14 | 0.54 | 1.000 | 1.000 | 1.000 | 1443 | tags=38%, list=25%, signal=50% |
| 127 | MRNA\_SPLICING |  | 17 | 0.16 | 0.53 | 0.991 | 1.000 | 1.000 | 4786 | tags=100%, list=84%, signal=608% |
| 128 | SHIPP\_DLBCL\_CURED\_DN |  | 18 | 0.15 | 0.51 | 1.000 | 1.000 | 1.000 | 525 | tags=11%, list=9%, signal=12% |
| 129 | APOPTOSIS |  | 27 | 0.11 | 0.43 | 1.000 | 1.000 | 1.000 | 636 | tags=11%, list=11%, signal=12% |
| 130 | PARK\_MSCS\_BOTH |  | 17 | 0.13 | 0.42 | 0.995 | 1.000 | 1.000 | 984 | tags=18%, list=17%, signal=21% |
Table: Gene sets enriched in phenotype **LymphomaSeq (4 samples)**[plain text format]****

  
