# Supplementary material for: Comparative RNA-Seq and Microarray Analysis of Gene Expression Changes in B-Cell Lymphomas of Canis familiaris
Source: PLoS One. 2013 Apr 4;8(4):e61088. doi: 10.1371/journal.pone.0061088 (PMC3617154; doi:10.1371/journal.pone.0061088)
Supplement: Data File S2 — GSEA Results Files. (ZIP) [file pone.0061088.s005.zip › RNA-Seq/gsea_report_for_LymphomaSeq_v3.html]

Report for LymphomaSeq 1334928352252 [GSEA]

| GS  follow link to MSigDB | GS DETAILS | SIZE | ES | NES | NOM p-val | FDR q-val | FWER p-val | RANK AT MAX | LEADING EDGE || 1 | GINESTIER\_BREAST\_CANCER\_ZNF217\_AMPLIFIED\_DN | Details ... | 102 | 0.58 | 2.78 | 0.000 | 0.000 | 0.000 | 1034 | tags=52%, list=18%, signal=62% |
| 2 | GINESTIER\_BREAST\_CANCER\_20Q13\_AMPLIFICATION\_DN | Details ... | 75 | 0.57 | 2.66 | 0.000 | 0.000 | 0.000 | 1021 | tags=47%, list=18%, signal=56% |
| 3 | CROONQUIST\_IL6\_DEPRIVATION\_DN | Details ... | 43 | 0.57 | 2.40 | 0.000 | 0.001 | 0.003 | 1131 | tags=53%, list=20%, signal=66% |
| 4 | WELCSH\_BRCA1\_TARGETS\_1\_DN | Details ... | 71 | 0.49 | 2.28 | 0.000 | 0.004 | 0.012 | 1006 | tags=44%, list=18%, signal=52% |
| 5 | NADERI\_BREAST\_CANCER\_PROGNOSIS\_UP | Details ... | 15 | 0.71 | 2.26 | 0.005 | 0.005 | 0.017 | 1035 | tags=67%, list=18%, signal=81% |
| 6 | SHEPARD\_BMYB\_TARGETS | Details ... | 21 | 0.64 | 2.24 | 0.000 | 0.005 | 0.019 | 857 | tags=43%, list=15%, signal=50% |
| 7 | SIG\_BCR\_SIGNALING\_PATHWAY | Details ... | 30 | 0.59 | 2.23 | 0.000 | 0.004 | 0.022 | 922 | tags=37%, list=16%, signal=43% |
| 8 | SOTIRIOU\_BREAST\_CANCER\_GRADE\_1\_VS\_3\_UP | Details ... | 72 | 0.45 | 2.17 | 0.000 | 0.007 | 0.040 | 1193 | tags=46%, list=21%, signal=57% |
| 9 | REN\_BOUND\_BY\_E2F | Details ... | 30 | 0.56 | 2.16 | 0.000 | 0.007 | 0.046 | 505 | tags=23%, list=9%, signal=25% |
| 10 | KANG\_DOXORUBICIN\_RESISTANCE\_UP | Details ... | 25 | 0.60 | 2.16 | 0.006 | 0.007 | 0.047 | 1280 | tags=56%, list=22%, signal=72% |
| 11 | ROSTY\_CERVICAL\_CANCER\_PROLIFERATION\_CLUSTER | Details ... | 65 | 0.48 | 2.15 | 0.000 | 0.007 | 0.051 | 1131 | tags=43%, list=20%, signal=53% |
| 12 | SCHLOSSER\_MYC\_TARGETS\_AND\_SERUM\_RESPONSE\_UP | Details ... | 26 | 0.56 | 2.07 | 0.000 | 0.013 | 0.111 | 478 | tags=35%, list=8%, signal=38% |
| 13 | FRASOR\_RESPONSE\_TO\_SERM\_OR\_FULVESTRANT\_DN | Details ... | 21 | 0.60 | 2.05 | 0.000 | 0.014 | 0.124 | 991 | tags=52%, list=17%, signal=63% |
| 14 | CROONQUIST\_NRAS\_SIGNALING\_DN | Details ... | 40 | 0.49 | 2.02 | 0.000 | 0.017 | 0.152 | 1103 | tags=43%, list=19%, signal=52% |
| 15 | KEGG\_B\_CELL\_RECEPTOR\_SIGNALING\_PATHWAY | Details ... | 50 | 0.46 | 2.00 | 0.000 | 0.020 | 0.195 | 918 | tags=32%, list=16%, signal=38% |
| 16 | PUJANA\_BREAST\_CANCER\_LIT\_INT\_NETWORK | Details ... | 56 | 0.45 | 2.00 | 0.000 | 0.019 | 0.195 | 1258 | tags=48%, list=22%, signal=61% |
| 17 | REACTOME\_DNA\_STRAND\_ELONGATION | Details ... | 22 | 0.55 | 1.96 | 0.000 | 0.026 | 0.270 | 1048 | tags=45%, list=18%, signal=55% |
| 18 | REACTOME\_DNA\_REPAIR | Details ... | 62 | 0.42 | 1.93 | 0.000 | 0.031 | 0.327 | 805 | tags=29%, list=14%, signal=33% |
| 19 | PASQUALUCCI\_LYMPHOMA\_BY\_GC\_STAGE\_DN | Details ... | 72 | 0.42 | 1.92 | 0.000 | 0.031 | 0.344 | 812 | tags=31%, list=14%, signal=35% |
| 20 | KAUFFMANN\_DNA\_REPAIR\_GENES | Details ... | 101 | 0.40 | 1.88 | 0.000 | 0.039 | 0.426 | 1258 | tags=40%, list=22%, signal=50% |
| 21 | RIZ\_ERYTHROID\_DIFFERENTIATION |  | 29 | 0.49 | 1.85 | 0.000 | 0.047 | 0.502 | 1048 | tags=55%, list=18%, signal=67% |
| 22 | BIOCARTA\_BCR\_PATHWAY |  | 24 | 0.51 | 1.85 | 0.000 | 0.047 | 0.516 | 1049 | tags=38%, list=18%, signal=46% |
| 23 | RASHI\_RESPONSE\_TO\_IONIZING\_RADIATION\_3 |  | 19 | 0.53 | 1.85 | 0.000 | 0.045 | 0.519 | 713 | tags=42%, list=12%, signal=48% |
| 24 | MUELLER\_PLURINET |  | 141 | 0.35 | 1.84 | 0.000 | 0.046 | 0.541 | 1344 | tags=43%, list=23%, signal=55% |
| 25 | WAKASUGI\_HAVE\_ZNF143\_BINDING\_SITES |  | 32 | 0.46 | 1.80 | 0.007 | 0.058 | 0.650 | 1258 | tags=47%, list=22%, signal=60% |
| 26 | LAIHO\_COLORECTAL\_CANCER\_SERRATED\_DN |  | 29 | 0.47 | 1.80 | 0.000 | 0.058 | 0.670 | 911 | tags=34%, list=16%, signal=41% |
| 27 | ST\_B\_CELL\_ANTIGEN\_RECEPTOR |  | 27 | 0.47 | 1.76 | 0.006 | 0.074 | 0.749 | 167 | tags=15%, list=3%, signal=15% |
| 28 | MANALO\_HYPOXIA\_DN |  | 165 | 0.34 | 1.75 | 0.000 | 0.074 | 0.767 | 1392 | tags=46%, list=24%, signal=59% |
| 29 | KEGG\_HOMOLOGOUS\_RECOMBINATION |  | 16 | 0.55 | 1.75 | 0.004 | 0.074 | 0.783 | 1205 | tags=56%, list=21%, signal=71% |
| 30 | ZHANG\_RESPONSE\_TO\_CANTHARIDIN\_DN |  | 30 | 0.47 | 1.74 | 0.007 | 0.076 | 0.803 | 1377 | tags=47%, list=24%, signal=61% |
| 31 | LI\_WILMS\_TUMOR\_VS\_FETAL\_KIDNEY\_1\_DN |  | 76 | 0.37 | 1.73 | 0.000 | 0.075 | 0.814 | 1051 | tags=33%, list=18%, signal=40% |
| 32 | KAUFFMANN\_MELANOMA\_RELAPSE\_UP |  | 28 | 0.45 | 1.73 | 0.012 | 0.077 | 0.832 | 1258 | tags=39%, list=22%, signal=50% |
| 33 | SIG\_PIP3\_SIGNALING\_IN\_B\_LYMPHOCYTES |  | 19 | 0.51 | 1.72 | 0.005 | 0.076 | 0.835 | 1049 | tags=37%, list=18%, signal=45% |
| 34 | WHITEFORD\_PEDIATRIC\_CANCER\_MARKERS |  | 37 | 0.41 | 1.72 | 0.000 | 0.076 | 0.845 | 1048 | tags=30%, list=18%, signal=36% |
| 35 | LIN\_MELANOMA\_COPY\_NUMBER\_UP |  | 24 | 0.47 | 1.69 | 0.011 | 0.090 | 0.892 | 831 | tags=46%, list=15%, signal=53% |
| 36 | ELVIDGE\_HIF1A\_AND\_HIF2A\_TARGETS\_UP |  | 22 | 0.47 | 1.66 | 0.006 | 0.104 | 0.928 | 622 | tags=36%, list=11%, signal=41% |
| 37 | POMEROY\_MEDULLOBLASTOMA\_PROGNOSIS\_DN |  | 17 | 0.50 | 1.66 | 0.015 | 0.105 | 0.936 | 753 | tags=35%, list=13%, signal=41% |
| 38 | REACTOME\_GENERIC\_TRANSCRIPTION\_PATHWAY |  | 16 | 0.49 | 1.65 | 0.017 | 0.103 | 0.936 | 956 | tags=50%, list=17%, signal=60% |
| 39 | KEGG\_DNA\_REPLICATION |  | 24 | 0.46 | 1.65 | 0.012 | 0.101 | 0.939 | 1048 | tags=38%, list=18%, signal=46% |
| 40 | SHEPARD\_CRUSH\_AND\_BURN\_MUTANT\_DN |  | 47 | 0.39 | 1.64 | 0.000 | 0.109 | 0.952 | 1212 | tags=38%, list=21%, signal=48% |
| 41 | KLEIN\_TARGETS\_OF\_BCR\_ABL1\_FUSION |  | 15 | 0.51 | 1.63 | 0.034 | 0.108 | 0.957 | 229 | tags=27%, list=4%, signal=28% |
| 42 | DAZARD\_RESPONSE\_TO\_UV\_SCC\_DN |  | 47 | 0.37 | 1.62 | 0.010 | 0.112 | 0.963 | 665 | tags=32%, list=12%, signal=36% |
| 43 | REACTOME\_PEPTIDE\_CHAIN\_ELONGATION |  | 18 | 0.49 | 1.61 | 0.024 | 0.119 | 0.970 | 2920 | tags=100%, list=51%, signal=204% |
| 44 | KEGG\_TYPE\_II\_DIABETES\_MELLITUS |  | 18 | 0.48 | 1.61 | 0.037 | 0.120 | 0.977 | 348 | tags=33%, list=6%, signal=35% |
| 45 | REACTOME\_EXTENSION\_OF\_TELOMERES |  | 18 | 0.49 | 1.60 | 0.033 | 0.122 | 0.979 | 1205 | tags=44%, list=21%, signal=56% |
| 46 | GRABARCZYK\_BCL11B\_TARGETS\_DN |  | 22 | 0.46 | 1.60 | 0.035 | 0.120 | 0.980 | 727 | tags=32%, list=13%, signal=36% |
| 47 | FAELT\_B\_CLL\_WITH\_VH3\_21\_UP |  | 22 | 0.45 | 1.60 | 0.016 | 0.120 | 0.983 | 1499 | tags=77%, list=26%, signal=104% |
| 48 | KEGG\_MISMATCH\_REPAIR |  | 18 | 0.47 | 1.59 | 0.031 | 0.125 | 0.991 | 505 | tags=22%, list=9%, signal=24% |
| 49 | REACTOME\_ACTIVATION\_OF\_THE\_PRE\_REPLICATIVE\_COMPLEX |  | 17 | 0.50 | 1.59 | 0.035 | 0.123 | 0.992 | 1205 | tags=53%, list=21%, signal=67% |
| 50 | REACTOME\_NUCLEOTIDE\_EXCISION\_REPAIR |  | 33 | 0.41 | 1.58 | 0.013 | 0.122 | 0.993 | 774 | tags=27%, list=14%, signal=31% |
| 51 | GARGALOVIC\_RESPONSE\_TO\_OXIDIZED\_PHOSPHOLIPIDS\_TURQUOISE\_DN |  | 28 | 0.41 | 1.58 | 0.034 | 0.123 | 0.995 | 1103 | tags=54%, list=19%, signal=66% |
| 52 | KOBAYASHI\_EGFR\_SIGNALING\_24HR\_DN |  | 123 | 0.31 | 1.58 | 0.000 | 0.121 | 0.995 | 1320 | tags=40%, list=23%, signal=51% |
| 53 | FURUKAWA\_DUSP6\_TARGETS\_PCI35\_DN |  | 29 | 0.42 | 1.57 | 0.019 | 0.122 | 0.996 | 1048 | tags=45%, list=18%, signal=55% |
| 54 | DEBIASI\_APOPTOSIS\_BY\_REOVIRUS\_INFECTION\_DN |  | 99 | 0.32 | 1.57 | 0.000 | 0.122 | 0.996 | 645 | tags=25%, list=11%, signal=28% |
| 55 | PUJANA\_BREAST\_CANCER\_WITH\_BRCA1\_MUTATED\_UP |  | 41 | 0.37 | 1.57 | 0.009 | 0.120 | 0.996 | 1048 | tags=34%, list=18%, signal=42% |
| 56 | ZHAN\_MULTIPLE\_MYELOMA\_PR\_UP |  | 22 | 0.44 | 1.57 | 0.031 | 0.119 | 0.996 | 1337 | tags=50%, list=23%, signal=65% |
| 57 | REACTOME\_GLOBAL\_GENOMIC\_NER |  | 23 | 0.43 | 1.57 | 0.053 | 0.119 | 0.997 | 774 | tags=30%, list=14%, signal=35% |
| 58 | REACTOME\_MRNA\_SPLICING |  | 61 | 0.35 | 1.56 | 0.011 | 0.118 | 0.997 | 1687 | tags=59%, list=29%, signal=83% |
| 59 | REACTOME\_ELONGATION\_AND\_PROCESSING\_OF\_CAPPED\_TRANSCRIPTS |  | 76 | 0.32 | 1.56 | 0.000 | 0.122 | 0.998 | 1641 | tags=53%, list=29%, signal=73% |
| 60 | NIKOLSKY\_BREAST\_CANCER\_20Q12\_Q13\_AMPLICON |  | 35 | 0.39 | 1.54 | 0.029 | 0.129 | 0.999 | 1214 | tags=40%, list=21%, signal=50% |
| 61 | REACTOME\_CENTROSOME\_MATURATION |  | 39 | 0.38 | 1.54 | 0.026 | 0.128 | 0.999 | 1196 | tags=44%, list=21%, signal=55% |
| 62 | REACTOME\_MRNA\_3\_END\_PROCESSING |  | 19 | 0.45 | 1.54 | 0.033 | 0.133 | 0.999 | 1598 | tags=68%, list=28%, signal=95% |
| 63 | KEGG\_CELL\_CYCLE |  | 68 | 0.34 | 1.53 | 0.000 | 0.135 | 0.999 | 1279 | tags=40%, list=22%, signal=51% |
| 64 | SCHLOSSER\_SERUM\_RESPONSE\_UP |  | 48 | 0.35 | 1.51 | 0.009 | 0.150 | 1.000 | 978 | tags=38%, list=17%, signal=45% |
| 65 | REACTOME\_LOSS\_OF\_NLP\_FROM\_MITOTIC\_CENTROSOMES |  | 36 | 0.36 | 1.51 | 0.022 | 0.149 | 1.000 | 1116 | tags=42%, list=20%, signal=51% |
| 66 | REACTOME\_TRANSCRIPTION\_COUPLED\_NER |  | 28 | 0.39 | 1.49 | 0.036 | 0.158 | 1.000 | 774 | tags=25%, list=14%, signal=29% |
| 67 | REACTOME\_G2\_M\_TRANSITION |  | 45 | 0.35 | 1.48 | 0.022 | 0.171 | 1.000 | 1196 | tags=40%, list=21%, signal=50% |
| 68 | MITSIADES\_RESPONSE\_TO\_APLIDIN\_DN |  | 135 | 0.31 | 1.47 | 0.000 | 0.175 | 1.000 | 1048 | tags=31%, list=18%, signal=37% |
| 69 | VECCHI\_GASTRIC\_CANCER\_EARLY\_UP |  | 163 | 0.27 | 1.47 | 0.000 | 0.176 | 1.000 | 1064 | tags=35%, list=19%, signal=42% |
| 70 | MORI\_EMU\_MYC\_LYMPHOMA\_BY\_ONSET\_TIME\_UP |  | 48 | 0.34 | 1.46 | 0.038 | 0.183 | 1.000 | 752 | tags=33%, list=13%, signal=38% |
| 71 | KEGG\_NUCLEOTIDE\_EXCISION\_REPAIR |  | 31 | 0.36 | 1.46 | 0.020 | 0.181 | 1.000 | 774 | tags=26%, list=14%, signal=30% |
| 72 | PUJANA\_XPRSS\_INT\_NETWORK |  | 112 | 0.28 | 1.46 | 0.000 | 0.182 | 1.000 | 976 | tags=25%, list=17%, signal=30% |
| 73 | BENPORATH\_ES\_CORE\_NINE\_CORRELATED |  | 43 | 0.34 | 1.45 | 0.030 | 0.190 | 1.000 | 744 | tags=33%, list=13%, signal=37% |
| 74 | ELVIDGE\_HYPOXIA\_BY\_DMOG\_DN |  | 26 | 0.39 | 1.43 | 0.073 | 0.204 | 1.000 | 615 | tags=23%, list=11%, signal=26% |
| 75 | PUJANA\_BRCA\_CENTERED\_NETWORK |  | 64 | 0.32 | 1.43 | 0.014 | 0.202 | 1.000 | 566 | tags=20%, list=10%, signal=22% |
| 76 | REACTOME\_FORMATION\_OF\_A\_POOL\_OF\_FREE\_40S\_SUBUNITS |  | 26 | 0.38 | 1.43 | 0.060 | 0.199 | 1.000 | 3532 | tags=100%, list=62%, signal=260% |
| 77 | HUMMEL\_BURKITTS\_LYMPHOMA\_UP |  | 17 | 0.43 | 1.43 | 0.076 | 0.200 | 1.000 | 470 | tags=29%, list=8%, signal=32% |
| 78 | FARMER\_BREAST\_CANCER\_CLUSTER\_2 |  | 19 | 0.42 | 1.43 | 0.099 | 0.198 | 1.000 | 1193 | tags=47%, list=21%, signal=60% |
| 79 | CHEMNITZ\_RESPONSE\_TO\_PROSTAGLANDIN\_E2\_UP |  | 61 | 0.31 | 1.40 | 0.011 | 0.227 | 1.000 | 659 | tags=23%, list=12%, signal=26% |
| 80 | KEGG\_RNA\_POLYMERASE |  | 16 | 0.44 | 1.40 | 0.091 | 0.225 | 1.000 | 212 | tags=19%, list=4%, signal=19% |
| 81 | VANTVEER\_BREAST\_CANCER\_BRCA1\_UP |  | 21 | 0.40 | 1.40 | 0.089 | 0.222 | 1.000 | 486 | tags=24%, list=8%, signal=26% |
| 82 | REACTOME\_TELOMERE\_MAINTENANCE |  | 22 | 0.40 | 1.40 | 0.064 | 0.222 | 1.000 | 1243 | tags=45%, list=22%, signal=58% |
| 83 | BARRIER\_CANCER\_RELAPSE\_NORMAL\_SAMPLE\_DN |  | 15 | 0.44 | 1.40 | 0.084 | 0.220 | 1.000 | 1219 | tags=53%, list=21%, signal=68% |
| 84 | REACTOME\_CELL\_CYCLE\_MITOTIC |  | 176 | 0.27 | 1.39 | 0.000 | 0.226 | 1.000 | 1231 | tags=33%, list=22%, signal=41% |
| 85 | REACTOME\_REGULATION\_OF\_GENE\_EXPRESSION\_IN\_BETA\_CELLS |  | 18 | 0.41 | 1.39 | 0.074 | 0.225 | 1.000 | 3394 | tags=100%, list=59%, signal=245% |
| 86 | REACTOME\_DOUBLE\_STRAND\_BREAK\_REPAIR |  | 15 | 0.44 | 1.39 | 0.091 | 0.226 | 1.000 | 1205 | tags=40%, list=21%, signal=51% |
| 87 | MORI\_LARGE\_PRE\_BII\_LYMPHOCYTE\_UP |  | 23 | 0.38 | 1.39 | 0.089 | 0.223 | 1.000 | 1280 | tags=48%, list=22%, signal=61% |
| 88 | NIKOLSKY\_BREAST\_CANCER\_16P13\_AMPLICON |  | 18 | 0.42 | 1.39 | 0.092 | 0.221 | 1.000 | 671 | tags=33%, list=12%, signal=38% |
| 89 | DAZARD\_RESPONSE\_TO\_UV\_NHEK\_DN |  | 140 | 0.28 | 1.38 | 0.000 | 0.223 | 1.000 | 660 | tags=21%, list=12%, signal=24% |
| 90 | AMUNDSON\_GAMMA\_RADIATION\_RESPONSE |  | 15 | 0.45 | 1.38 | 0.093 | 0.229 | 1.000 | 1279 | tags=60%, list=22%, signal=77% |
| 91 | REACTOME\_ACTIVATION\_OF\_ATR\_IN\_RESPONSE\_TO\_REPLICATION\_STRESS |  | 23 | 0.39 | 1.37 | 0.112 | 0.233 | 1.000 | 505 | tags=22%, list=9%, signal=24% |
| 92 | NIKOLSKY\_BREAST\_CANCER\_17Q11\_Q21\_AMPLICON |  | 39 | 0.34 | 1.37 | 0.096 | 0.231 | 1.000 | 1088 | tags=44%, list=19%, signal=53% |
| 93 | REACTOME\_MTOR\_SIGNALLING |  | 15 | 0.44 | 1.37 | 0.079 | 0.228 | 1.000 | 434 | tags=33%, list=8%, signal=36% |
| 94 | JISON\_SICKLE\_CELL\_DISEASE\_DN |  | 64 | 0.30 | 1.36 | 0.037 | 0.243 | 1.000 | 934 | tags=28%, list=16%, signal=33% |
| 95 | BANDRES\_RESPONSE\_TO\_CARMUSTIN\_MGMT\_48HR\_DN |  | 29 | 0.34 | 1.35 | 0.059 | 0.246 | 1.000 | 722 | tags=34%, list=13%, signal=39% |
| 96 | SA\_B\_CELL\_RECEPTOR\_COMPLEXES |  | 18 | 0.41 | 1.35 | 0.097 | 0.243 | 1.000 | 1049 | tags=28%, list=18%, signal=34% |
| 97 | ZHAN\_EARLY\_DIFFERENTIATION\_GENES\_DN |  | 21 | 0.40 | 1.35 | 0.119 | 0.244 | 1.000 | 1298 | tags=43%, list=23%, signal=55% |
| 98 | REACTOME\_FORMATION\_OF\_THE\_TERNARY\_COMPLEX\_AND\_SUBSEQUENTLY\_THE\_43S\_COMPLEX |  | 21 | 0.38 | 1.35 | 0.079 | 0.245 | 1.000 | 3532 | tags=100%, list=62%, signal=260% |
| 99 | REACTOME\_G2\_M\_CHECKPOINTS |  | 27 | 0.36 | 1.34 | 0.094 | 0.248 | 1.000 | 1279 | tags=37%, list=22%, signal=47% |
| 100 | KEGG\_PYRIMIDINE\_METABOLISM |  | 47 | 0.33 | 1.34 | 0.049 | 0.250 | 1.000 | 243 | tags=15%, list=4%, signal=15% |
| 101 | KEGG\_PHOSPHATIDYLINOSITOL\_SIGNALING\_SYSTEM |  | 34 | 0.34 | 1.34 | 0.070 | 0.252 | 1.000 | 707 | tags=26%, list=12%, signal=30% |
| 102 | SCIBETTA\_KDM5B\_TARGETS\_DN |  | 34 | 0.34 | 1.32 | 0.099 | 0.266 | 1.000 | 408 | tags=24%, list=7%, signal=25% |
| 103 | REACTOME\_FORMATION\_AND\_MATURATION\_OF\_MRNA\_TRANSCRIPT |  | 83 | 0.27 | 1.32 | 0.050 | 0.265 | 1.000 | 1416 | tags=40%, list=25%, signal=52% |
| 104 | MORI\_LARGE\_PRE\_BII\_LYMPHOCYTE\_DN |  | 31 | 0.35 | 1.32 | 0.119 | 0.265 | 1.000 | 909 | tags=35%, list=16%, signal=42% |
| 105 | CAIRO\_HEPATOBLASTOMA\_CLASSES\_UP |  | 310 | 0.23 | 1.32 | 0.000 | 0.264 | 1.000 | 1072 | tags=27%, list=19%, signal=32% |
| 106 | PUJANA\_CHEK2\_PCC\_NETWORK |  | 407 | 0.22 | 1.32 | 0.000 | 0.267 | 1.000 | 684 | tags=15%, list=12%, signal=16% |
| 107 | SONG\_TARGETS\_OF\_IE86\_CMV\_PROTEIN |  | 30 | 0.35 | 1.31 | 0.085 | 0.276 | 1.000 | 757 | tags=27%, list=13%, signal=31% |
| 108 | SENGUPTA\_EBNA1\_ANTICORRELATED |  | 36 | 0.32 | 1.30 | 0.116 | 0.279 | 1.000 | 975 | tags=50%, list=17%, signal=60% |
| 109 | KEGG\_INOSITOL\_PHOSPHATE\_METABOLISM |  | 26 | 0.36 | 1.30 | 0.116 | 0.284 | 1.000 | 246 | tags=19%, list=4%, signal=20% |
| 110 | CHANG\_CYCLING\_GENES |  | 22 | 0.37 | 1.30 | 0.096 | 0.282 | 1.000 | 930 | tags=32%, list=16%, signal=38% |
| 111 | DANG\_REGULATED\_BY\_MYC\_UP |  | 30 | 0.34 | 1.29 | 0.102 | 0.290 | 1.000 | 1380 | tags=47%, list=24%, signal=61% |
| 112 | SU\_TESTIS |  | 28 | 0.34 | 1.28 | 0.147 | 0.310 | 1.000 | 664 | tags=25%, list=12%, signal=28% |
| 113 | SCHUHMACHER\_MYC\_TARGETS\_UP |  | 34 | 0.33 | 1.27 | 0.117 | 0.320 | 1.000 | 1073 | tags=41%, list=19%, signal=50% |
| 114 | TOYOTA\_TARGETS\_OF\_MIR34B\_AND\_MIR34C |  | 192 | 0.23 | 1.27 | 0.000 | 0.320 | 1.000 | 1131 | tags=29%, list=20%, signal=35% |
| 115 | KEGG\_AMINOACYL\_TRNA\_BIOSYNTHESIS |  | 28 | 0.32 | 1.27 | 0.148 | 0.319 | 1.000 | 1188 | tags=39%, list=21%, signal=49% |
| 116 | PUJANA\_BRCA2\_PCC\_NETWORK |  | 248 | 0.27 | 1.26 | 0.000 | 0.326 | 1.000 | 803 | tags=21%, list=14%, signal=24% |
| 117 | BROWNE\_HCMV\_INFECTION\_6HR\_UP |  | 18 | 0.37 | 1.26 | 0.188 | 0.328 | 1.000 | 687 | tags=28%, list=12%, signal=31% |
| 118 | MISSIAGLIA\_REGULATED\_BY\_METHYLATION\_DN |  | 45 | 0.28 | 1.26 | 0.110 | 0.327 | 1.000 | 810 | tags=27%, list=14%, signal=31% |
| 119 | BILD\_MYC\_ONCOGENIC\_SIGNATURE |  | 81 | 0.26 | 1.25 | 0.071 | 0.342 | 1.000 | 1107 | tags=43%, list=19%, signal=53% |
| 120 | LEE\_EARLY\_T\_LYMPHOCYTE\_UP |  | 30 | 0.33 | 1.24 | 0.149 | 0.345 | 1.000 | 1048 | tags=40%, list=18%, signal=49% |
| 121 | GALE\_APL\_WITH\_FLT3\_MUTATED\_UP |  | 32 | 0.31 | 1.24 | 0.177 | 0.344 | 1.000 | 1006 | tags=28%, list=18%, signal=34% |
| 122 | REACTOME\_PROCESSING\_OF\_CAPPED\_INTRON\_CONTAINING\_PRE\_MRNA |  | 80 | 0.27 | 1.24 | 0.119 | 0.343 | 1.000 | 1641 | tags=51%, list=29%, signal=71% |
| 123 | KLEIN\_PRIMARY\_EFFUSION\_LYMPHOMA\_DN |  | 40 | 0.30 | 1.24 | 0.107 | 0.341 | 1.000 | 344 | tags=25%, list=6%, signal=26% |
| 124 | REACTOME\_TRANSCRIPTION |  | 75 | 0.27 | 1.24 | 0.073 | 0.338 | 1.000 | 601 | tags=19%, list=11%, signal=21% |
| 125 | REACTOME\_S\_PHASE |  | 62 | 0.27 | 1.22 | 0.125 | 0.370 | 1.000 | 1309 | tags=34%, list=23%, signal=43% |
| 126 | DOANE\_BREAST\_CANCER\_CLASSES\_UP |  | 16 | 0.37 | 1.22 | 0.194 | 0.373 | 1.000 | 444 | tags=31%, list=8%, signal=34% |
| 127 | BIOCARTA\_G1\_PATHWAY |  | 15 | 0.39 | 1.20 | 0.199 | 0.392 | 1.000 | 869 | tags=27%, list=15%, signal=31% |
| 128 | WONG\_EMBRYONIC\_STEM\_CELL\_CORE |  | 186 | 0.21 | 1.20 | 0.000 | 0.396 | 1.000 | 563 | tags=13%, list=10%, signal=14% |
| 129 | KEGG\_INSULIN\_SIGNALING\_PATHWAY |  | 58 | 0.26 | 1.20 | 0.141 | 0.400 | 1.000 | 876 | tags=24%, list=15%, signal=28% |
| 130 | JAIN\_NFKB\_SIGNALING |  | 39 | 0.30 | 1.19 | 0.202 | 0.408 | 1.000 | 835 | tags=26%, list=15%, signal=30% |
| 131 | TAKAO\_RESPONSE\_TO\_UVB\_RADIATION\_DN |  | 47 | 0.29 | 1.18 | 0.157 | 0.421 | 1.000 | 495 | tags=19%, list=9%, signal=21% |
| 132 | REACTOME\_RNA\_POLYMERASE\_I\_III\_AND\_MITOCHONDRIAL\_TRANSCRIPTION |  | 33 | 0.31 | 1.18 | 0.204 | 0.418 | 1.000 | 601 | tags=21%, list=11%, signal=24% |
| 133 | REACTOME\_RNA\_POLYMERASE\_III\_TRANSCRIPTION\_INITIATION |  | 15 | 0.37 | 1.17 | 0.274 | 0.436 | 1.000 | 418 | tags=20%, list=7%, signal=22% |
| 134 | KEGG\_RIBOSOME |  | 20 | 0.34 | 1.17 | 0.237 | 0.433 | 1.000 | 3779 | tags=100%, list=66%, signal=293% |
| 135 | YAO\_TEMPORAL\_RESPONSE\_TO\_PROGESTERONE\_CLUSTER\_7 |  | 32 | 0.30 | 1.17 | 0.199 | 0.438 | 1.000 | 364 | tags=16%, list=6%, signal=17% |
| 136 | REACTOME\_SYNTHESIS\_OF\_DNA |  | 53 | 0.27 | 1.16 | 0.155 | 0.448 | 1.000 | 1206 | tags=28%, list=21%, signal=36% |
| 137 | NIKOLSKY\_BREAST\_CANCER\_16Q24\_AMPLICON |  | 19 | 0.36 | 1.16 | 0.246 | 0.448 | 1.000 | 1309 | tags=47%, list=23%, signal=61% |
| 138 | BIOCARTA\_CREB\_PATHWAY |  | 15 | 0.36 | 1.16 | 0.244 | 0.448 | 1.000 | 3645 | tags=100%, list=64%, signal=275% |
| 139 | BLUM\_RESPONSE\_TO\_SALIRASIB\_DN |  | 162 | 0.22 | 1.15 | 0.111 | 0.465 | 1.000 | 1033 | tags=27%, list=18%, signal=32% |
| 140 | DAIRKEE\_CANCER\_PRONE\_RESPONSE\_BPA |  | 20 | 0.33 | 1.14 | 0.284 | 0.476 | 1.000 | 920 | tags=30%, list=16%, signal=36% |
| 141 | WU\_APOPTOSIS\_BY\_CDKN1A\_VIA\_TP53 |  | 17 | 0.35 | 1.14 | 0.290 | 0.476 | 1.000 | 1193 | tags=47%, list=21%, signal=59% |
| 142 | ODONNELL\_TARGETS\_OF\_MYC\_AND\_TFRC\_DN |  | 25 | 0.32 | 1.14 | 0.260 | 0.484 | 1.000 | 857 | tags=28%, list=15%, signal=33% |
| 143 | KAUFFMANN\_DNA\_REPLICATION\_GENES |  | 62 | 0.25 | 1.14 | 0.162 | 0.485 | 1.000 | 529 | tags=16%, list=9%, signal=18% |
| 144 | KEGG\_GLYCOLYSIS\_GLUCONEOGENESIS |  | 15 | 0.36 | 1.14 | 0.285 | 0.482 | 1.000 | 428 | tags=27%, list=7%, signal=29% |
| 145 | KIM\_WT1\_TARGETS\_8HR\_DN |  | 37 | 0.29 | 1.13 | 0.286 | 0.485 | 1.000 | 891 | tags=30%, list=16%, signal=35% |
| 146 | HOFFMANN\_LARGE\_TO\_SMALL\_PRE\_BII\_LYMPHOCYTE\_UP |  | 53 | 0.26 | 1.13 | 0.208 | 0.493 | 1.000 | 999 | tags=28%, list=17%, signal=34% |
| 147 | TOOKER\_GEMCITABINE\_RESISTANCE\_UP |  | 43 | 0.27 | 1.12 | 0.263 | 0.506 | 1.000 | 1141 | tags=33%, list=20%, signal=40% |
| 148 | JAZAERI\_BREAST\_CANCER\_BRCA1\_VS\_BRCA2\_UP |  | 24 | 0.32 | 1.12 | 0.256 | 0.507 | 1.000 | 1322 | tags=42%, list=23%, signal=54% |
| 149 | ELVIDGE\_HIF1A\_TARGETS\_UP |  | 38 | 0.27 | 1.11 | 0.280 | 0.523 | 1.000 | 622 | tags=24%, list=11%, signal=26% |
| 150 | KEGG\_WNT\_SIGNALING\_PATHWAY |  | 55 | 0.25 | 1.11 | 0.235 | 0.521 | 1.000 | 667 | tags=22%, list=12%, signal=24% |
| 151 | MORI\_PRE\_BI\_LYMPHOCYTE\_DN |  | 48 | 0.26 | 1.10 | 0.220 | 0.529 | 1.000 | 699 | tags=21%, list=12%, signal=24% |
| 152 | REACTOME\_MITOTIC\_PROMETAPHASE |  | 56 | 0.24 | 1.10 | 0.237 | 0.531 | 1.000 | 1211 | tags=32%, list=21%, signal=40% |
| 153 | TOOKER\_RESPONSE\_TO\_BEXAROTENE\_DN |  | 43 | 0.27 | 1.10 | 0.310 | 0.535 | 1.000 | 1141 | tags=33%, list=20%, signal=40% |
| 154 | BIOCARTA\_FMLP\_PATHWAY |  | 18 | 0.33 | 1.09 | 0.318 | 0.553 | 1.000 | 918 | tags=28%, list=16%, signal=33% |
| 155 | SPIELMAN\_LYMPHOBLAST\_EUROPEAN\_VS\_ASIAN\_UP |  | 219 | 0.18 | 1.09 | 0.000 | 0.554 | 1.000 | 560 | tags=14%, list=10%, signal=15% |
| 156 | REACTOME\_MITOTIC\_M\_M\_G1\_PHASES |  | 93 | 0.22 | 1.07 | 0.227 | 0.599 | 1.000 | 1211 | tags=29%, list=21%, signal=36% |
| 157 | MORI\_IMMATURE\_B\_LYMPHOCYTE\_DN |  | 24 | 0.31 | 1.07 | 0.343 | 0.601 | 1.000 | 990 | tags=38%, list=17%, signal=45% |
| 158 | PYEON\_HPV\_POSITIVE\_TUMORS\_UP |  | 38 | 0.26 | 1.06 | 0.358 | 0.606 | 1.000 | 161 | tags=11%, list=3%, signal=11% |
| 159 | ZHAN\_MULTIPLE\_MYELOMA\_LB\_DN |  | 20 | 0.32 | 1.05 | 0.377 | 0.623 | 1.000 | 547 | tags=35%, list=10%, signal=39% |
| 160 | REACTOME\_RNA\_POLYMERASE\_III\_TRANSCRIPTION |  | 19 | 0.31 | 1.05 | 0.381 | 0.620 | 1.000 | 418 | tags=16%, list=7%, signal=17% |
| 161 | LIN\_APC\_TARGETS |  | 24 | 0.29 | 1.05 | 0.367 | 0.621 | 1.000 | 212 | tags=13%, list=4%, signal=13% |
| 162 | DACOSTA\_UV\_RESPONSE\_VIA\_ERCC3\_TTD\_UP |  | 16 | 0.33 | 1.05 | 0.361 | 0.622 | 1.000 | 238 | tags=19%, list=4%, signal=20% |
| 163 | ROVERSI\_GLIOMA\_COPY\_NUMBER\_UP |  | 18 | 0.32 | 1.05 | 0.400 | 0.630 | 1.000 | 659 | tags=28%, list=12%, signal=31% |
| 164 | GARCIA\_TARGETS\_OF\_FLI1\_AND\_DAX1\_UP |  | 16 | 0.33 | 1.04 | 0.377 | 0.630 | 1.000 | 652 | tags=38%, list=11%, signal=42% |
| 165 | REACTOME\_TRNA\_AMINOACYLATION |  | 29 | 0.28 | 1.04 | 0.417 | 0.635 | 1.000 | 1188 | tags=38%, list=21%, signal=48% |
| 166 | JAZAG\_TGFB1\_SIGNALING\_UP |  | 43 | 0.25 | 1.03 | 0.385 | 0.660 | 1.000 | 834 | tags=30%, list=15%, signal=35% |
| 167 | BIOCARTA\_CARM\_ER\_PATHWAY |  | 15 | 0.33 | 1.02 | 0.440 | 0.684 | 1.000 | 804 | tags=33%, list=14%, signal=39% |
| 168 | BIOCARTA\_IL2RB\_PATHWAY |  | 20 | 0.29 | 1.02 | 0.422 | 0.680 | 1.000 | 479 | tags=20%, list=8%, signal=22% |
| 169 | STEIN\_ESRRA\_TARGETS\_RESPONSIVE\_TO\_ESTROGEN\_DN |  | 24 | 0.28 | 1.01 | 0.474 | 0.694 | 1.000 | 1131 | tags=33%, list=20%, signal=41% |
| 170 | REACTOME\_GTP\_HYDROLYSIS\_AND\_JOINING\_OF\_THE\_60S\_RIBOSOMAL\_SUBUNIT |  | 33 | 0.25 | 1.01 | 0.392 | 0.696 | 1.000 | 3743 | tags=97%, list=65%, signal=279% |
| 171 | ZHAN\_MULTIPLE\_MYELOMA\_SUBGROUPS |  | 16 | 0.30 | 1.00 | 0.421 | 0.714 | 1.000 | 788 | tags=31%, list=14%, signal=36% |
| 172 | RICKMAN\_TUMOR\_DIFFERENTIATED\_WELL\_VS\_POORLY\_UP |  | 101 | 0.21 | 1.00 | 0.439 | 0.713 | 1.000 | 611 | tags=18%, list=11%, signal=20% |
| 173 | REACTOME\_MRNA\_SPLICING\_MINOR\_PATHWAY |  | 23 | 0.29 | 1.00 | 0.487 | 0.713 | 1.000 | 1623 | tags=52%, list=28%, signal=73% |
| 174 | TARTE\_PLASMA\_CELL\_VS\_B\_LYMPHOCYTE\_DN |  | 22 | 0.28 | 1.00 | 0.467 | 0.712 | 1.000 | 693 | tags=27%, list=12%, signal=31% |
| 175 | FERREIRA\_EWINGS\_SARCOMA\_UNSTABLE\_VS\_STABLE\_UP |  | 68 | 0.22 | 1.00 | 0.434 | 0.710 | 1.000 | 898 | tags=19%, list=16%, signal=22% |
| 176 | DAZARD\_UV\_RESPONSE\_CLUSTER\_G6 |  | 64 | 0.22 | 1.00 | 0.608 | 0.707 | 1.000 | 774 | tags=20%, list=14%, signal=23% |
| 177 | REACTOME\_RNA\_POLYMERASE\_II\_TRANSCRIPTION |  | 49 | 0.23 | 1.00 | 0.463 | 0.705 | 1.000 | 1623 | tags=47%, list=28%, signal=65% |
| 178 | BIOCARTA\_KERATINOCYTE\_PATHWAY |  | 28 | 0.25 | 0.99 | 0.484 | 0.728 | 1.000 | 181 | tags=11%, list=3%, signal=11% |
| 179 | WATANABE\_COLON\_CANCER\_MSI\_VS\_MSS\_DN |  | 15 | 0.31 | 0.99 | 0.480 | 0.731 | 1.000 | 115 | tags=20%, list=2%, signal=20% |
| 180 | CROMER\_METASTASIS\_DN |  | 26 | 0.27 | 0.98 | 0.456 | 0.728 | 1.000 | 668 | tags=27%, list=12%, signal=30% |
| 181 | WOOD\_EBV\_EBNA1\_TARGETS\_UP |  | 35 | 0.24 | 0.98 | 0.407 | 0.728 | 1.000 | 642 | tags=23%, list=11%, signal=26% |
| 182 | GARY\_CD5\_TARGETS\_UP |  | 222 | 0.18 | 0.98 | 0.750 | 0.745 | 1.000 | 658 | tags=18%, list=11%, signal=19% |
| 183 | REACTOME\_HIV1\_TRANSCRIPTION\_ELONGATION |  | 24 | 0.26 | 0.96 | 0.521 | 0.770 | 1.000 | 4243 | tags=100%, list=74%, signal=385% |
| 184 | REACTOME\_DUAL\_INCISION\_REACTION\_IN\_TC\_NER |  | 15 | 0.30 | 0.96 | 0.490 | 0.772 | 1.000 | 601 | tags=20%, list=11%, signal=22% |
| 185 | STARK\_PREFRONTAL\_CORTEX\_22Q11\_DELETION\_UP |  | 75 | 0.21 | 0.96 | 0.603 | 0.778 | 1.000 | 1015 | tags=25%, list=18%, signal=30% |
| 186 | REACTOME\_CELL\_CYCLE\_CHECKPOINTS |  | 71 | 0.20 | 0.96 | 0.585 | 0.776 | 1.000 | 1279 | tags=30%, list=22%, signal=38% |
| 187 | BIOCARTA\_TOLL\_PATHWAY |  | 19 | 0.28 | 0.96 | 0.495 | 0.775 | 1.000 | 70 | tags=11%, list=1%, signal=11% |
| 188 | REACTOME\_METABOLISM\_OF\_MRNA |  | 28 | 0.25 | 0.95 | 0.584 | 0.787 | 1.000 | 3953 | tags=96%, list=69%, signal=310% |
| 189 | GARCIA\_TARGETS\_OF\_FLI1\_AND\_DAX1\_DN |  | 64 | 0.22 | 0.95 | 0.643 | 0.792 | 1.000 | 1064 | tags=25%, list=19%, signal=30% |
| 190 | BOYAULT\_LIVER\_CANCER\_SUBCLASS\_G123\_UP |  | 20 | 0.29 | 0.94 | 0.534 | 0.794 | 1.000 | 449 | tags=25%, list=8%, signal=27% |
| 191 | REACTOME\_TAT\_MEDIATED\_HIV1\_ELONGATION\_ARREST\_AND\_RECOVERY |  | 18 | 0.29 | 0.94 | 0.512 | 0.795 | 1.000 | 4091 | tags=100%, list=71%, signal=350% |
| 192 | WINTER\_HYPOXIA\_UP |  | 34 | 0.24 | 0.94 | 0.611 | 0.807 | 1.000 | 132 | tags=9%, list=2%, signal=9% |
| 193 | KEGG\_AMYOTROPHIC\_LATERAL\_SCLEROSIS\_ALS |  | 18 | 0.28 | 0.94 | 0.520 | 0.804 | 1.000 | 226 | tags=11%, list=4%, signal=12% |
| 194 | KEGG\_LONG\_TERM\_POTENTIATION |  | 27 | 0.25 | 0.93 | 0.575 | 0.803 | 1.000 | 876 | tags=33%, list=15%, signal=39% |
| 195 | KEGG\_ERBB\_SIGNALING\_PATHWAY |  | 42 | 0.23 | 0.93 | 0.537 | 0.801 | 1.000 | 667 | tags=19%, list=12%, signal=21% |
| 196 | REACTOME\_G\_ALPHA\_12\_13\_SIGNALLING\_EVENTS |  | 26 | 0.25 | 0.93 | 0.605 | 0.809 | 1.000 | 149 | tags=8%, list=3%, signal=8% |
| 197 | LIU\_COMMON\_CANCER\_GENES |  | 20 | 0.28 | 0.92 | 0.598 | 0.817 | 1.000 | 757 | tags=25%, list=13%, signal=29% |
| 198 | REACTOME\_G1\_S\_TRANSITION |  | 55 | 0.20 | 0.92 | 0.678 | 0.822 | 1.000 | 1206 | tags=27%, list=21%, signal=34% |
| 199 | NUNODA\_RESPONSE\_TO\_DASATINIB\_IMATINIB\_UP |  | 18 | 0.29 | 0.91 | 0.537 | 0.834 | 1.000 | 1190 | tags=33%, list=21%, signal=42% |
| 200 | PENG\_LEUCINE\_DEPRIVATION\_DN |  | 26 | 0.25 | 0.91 | 0.586 | 0.831 | 1.000 | 1187 | tags=38%, list=21%, signal=48% |
| 201 | KEGG\_LYSINE\_DEGRADATION |  | 27 | 0.24 | 0.91 | 0.669 | 0.839 | 1.000 | 266 | tags=11%, list=5%, signal=12% |
| 202 | PYEON\_CANCER\_HEAD\_AND\_NECK\_VS\_CERVICAL\_UP |  | 88 | 0.19 | 0.90 | 0.804 | 0.853 | 1.000 | 278 | tags=9%, list=5%, signal=9% |
| 203 | ODONNELL\_TFRC\_TARGETS\_DN |  | 61 | 0.20 | 0.90 | 0.662 | 0.850 | 1.000 | 947 | tags=23%, list=17%, signal=27% |
| 204 | PUIFFE\_INVASION\_INHIBITED\_BY\_ASCITES\_UP |  | 31 | 0.22 | 0.90 | 0.667 | 0.855 | 1.000 | 1049 | tags=32%, list=18%, signal=39% |
| 205 | REACTOME\_IRS\_RELATED\_EVENTS |  | 37 | 0.22 | 0.90 | 0.682 | 0.851 | 1.000 | 434 | tags=16%, list=8%, signal=17% |
| 206 | REACTOME\_TRANSLATION\_INITIATION\_COMPLEX\_FORMATION |  | 24 | 0.25 | 0.89 | 0.619 | 0.865 | 1.000 | 4298 | tags=100%, list=75%, signal=400% |
| 207 | MOLENAAR\_TARGETS\_OF\_CCND1\_AND\_CDK4\_DN |  | 27 | 0.24 | 0.89 | 0.641 | 0.865 | 1.000 | 990 | tags=30%, list=17%, signal=36% |
| 208 | REACTOME\_P75\_NTR\_RECEPTOR\_MEDIATED\_SIGNALLING |  | 40 | 0.21 | 0.88 | 0.722 | 0.880 | 1.000 | 467 | tags=13%, list=8%, signal=14% |
| 209 | GEORGES\_CELL\_CYCLE\_MIR192\_TARGETS |  | 35 | 0.22 | 0.87 | 0.659 | 0.893 | 1.000 | 657 | tags=20%, list=11%, signal=22% |
| 210 | MORI\_IMMATURE\_B\_LYMPHOCYTE\_UP |  | 25 | 0.24 | 0.87 | 0.693 | 0.889 | 1.000 | 1063 | tags=40%, list=19%, signal=49% |
| 211 | THEILGAARD\_NEUTROPHIL\_AT\_SKIN\_WOUND\_DN |  | 119 | 0.18 | 0.87 | 0.909 | 0.889 | 1.000 | 824 | tags=19%, list=14%, signal=22% |
| 212 | KEGG\_RNA\_DEGRADATION |  | 35 | 0.22 | 0.86 | 0.736 | 0.914 | 1.000 | 782 | tags=17%, list=14%, signal=20% |
| 213 | STARK\_HYPPOCAMPUS\_22Q11\_DELETION\_UP |  | 19 | 0.25 | 0.85 | 0.690 | 0.931 | 1.000 | 1179 | tags=37%, list=21%, signal=46% |
| 214 | REACTOME\_MRNA\_PROCESSING |  | 16 | 0.26 | 0.84 | 0.689 | 0.933 | 1.000 | 4243 | tags=100%, list=74%, signal=386% |
| 215 | RADAEVA\_RESPONSE\_TO\_IFNA1\_UP |  | 16 | 0.26 | 0.84 | 0.718 | 0.931 | 1.000 | 687 | tags=31%, list=12%, signal=35% |
| 216 | REACTOME\_FORMATION\_OF\_THE\_EARLY\_ELONGATION\_COMPLEX |  | 18 | 0.26 | 0.84 | 0.714 | 0.929 | 1.000 | 4243 | tags=100%, list=74%, signal=385% |
| 217 | KEGG\_NON\_SMALL\_CELL\_LUNG\_CANCER |  | 29 | 0.22 | 0.84 | 0.737 | 0.927 | 1.000 | 876 | tags=24%, list=15%, signal=28% |
| 218 | ST\_PHOSPHOINOSITIDE\_3\_KINASE\_PATHWAY |  | 17 | 0.25 | 0.84 | 0.716 | 0.931 | 1.000 | 4291 | tags=100%, list=75%, signal=398% |
| 219 | SHIPP\_DLBCL\_CURED\_VS\_FATAL\_DN |  | 24 | 0.22 | 0.84 | 0.732 | 0.929 | 1.000 | 897 | tags=21%, list=16%, signal=25% |
| 220 | LIN\_MELANOMA\_COPY\_NUMBER\_DN |  | 22 | 0.23 | 0.83 | 0.719 | 0.932 | 1.000 | 531 | tags=18%, list=9%, signal=20% |
| 221 | REACTOME\_DNA\_REPLICATION\_PRE\_INITIATION |  | 44 | 0.19 | 0.83 | 0.780 | 0.930 | 1.000 | 1206 | tags=27%, list=21%, signal=34% |
| 222 | YU\_MYC\_TARGETS\_UP |  | 22 | 0.23 | 0.83 | 0.723 | 0.926 | 1.000 | 1193 | tags=36%, list=21%, signal=46% |
| 223 | ST\_G\_ALPHA\_I\_PATHWAY |  | 16 | 0.26 | 0.82 | 0.728 | 0.935 | 1.000 | 25 | tags=6%, list=0%, signal=6% |
| 224 | SIG\_PIP3\_SIGNALING\_IN\_CARDIAC\_MYOCTES |  | 30 | 0.22 | 0.82 | 0.789 | 0.945 | 1.000 | 801 | tags=20%, list=14%, signal=23% |
| 225 | BYSTRYKH\_HEMATOPOIESIS\_STEM\_CELL\_AND\_BRAIN\_QTL\_CIS |  | 34 | 0.21 | 0.81 | 0.857 | 0.948 | 1.000 | 882 | tags=24%, list=15%, signal=28% |
| 226 | GAZDA\_DIAMOND\_BLACKFAN\_ANEMIA\_MYELOID\_DN |  | 26 | 0.23 | 0.81 | 0.793 | 0.950 | 1.000 | 17 | tags=4%, list=0%, signal=4% |
| 227 | BIOCARTA\_IL2\_PATHWAY |  | 15 | 0.26 | 0.81 | 0.711 | 0.949 | 1.000 | 425 | tags=13%, list=7%, signal=14% |
| 228 | KEGG\_MTOR\_SIGNALING\_PATHWAY |  | 27 | 0.21 | 0.79 | 0.800 | 0.970 | 1.000 | 784 | tags=22%, list=14%, signal=26% |
| 229 | KEGG\_RIG\_I\_LIKE\_RECEPTOR\_SIGNALING\_PATHWAY |  | 33 | 0.20 | 0.79 | 0.766 | 0.971 | 1.000 | 733 | tags=18%, list=13%, signal=21% |
| 230 | ELVIDGE\_HYPOXIA\_DN |  | 77 | 0.17 | 0.79 | 0.895 | 0.969 | 1.000 | 1146 | tags=29%, list=20%, signal=35% |
| 231 | REACTOME\_GENE\_EXPRESSION |  | 204 | 0.15 | 0.78 | 1.000 | 0.971 | 1.000 | 1141 | tags=25%, list=20%, signal=30% |
| 232 | ZHAN\_VARIABLE\_EARLY\_DIFFERENTIATION\_GENES\_DN |  | 18 | 0.23 | 0.78 | 0.784 | 0.971 | 1.000 | 753 | tags=17%, list=13%, signal=19% |
| 233 | REACTOME\_METABOLISM\_OF\_CARBOHYDRATES |  | 45 | 0.19 | 0.78 | 0.894 | 0.971 | 1.000 | 452 | tags=13%, list=8%, signal=14% |
| 234 | DIRMEIER\_LMP1\_RESPONSE\_LATE\_UP |  | 22 | 0.22 | 0.77 | 0.851 | 0.975 | 1.000 | 618 | tags=23%, list=11%, signal=25% |
| 235 | FOURNIER\_ACINAR\_DEVELOPMENT\_LATE\_2 |  | 148 | 0.14 | 0.77 | 1.000 | 0.971 | 1.000 | 1103 | tags=26%, list=19%, signal=31% |
| 236 | CAFFAREL\_RESPONSE\_TO\_THC\_24HR\_5\_UP |  | 15 | 0.25 | 0.77 | 0.815 | 0.972 | 1.000 | 770 | tags=20%, list=13%, signal=23% |
| 237 | REACTOME\_GLUCOSE\_TRANSPORT |  | 19 | 0.23 | 0.77 | 0.781 | 0.970 | 1.000 | 295 | tags=11%, list=5%, signal=11% |
| 238 | BIOCARTA\_HCMV\_PATHWAY |  | 15 | 0.24 | 0.77 | 0.804 | 0.966 | 1.000 | 918 | tags=27%, list=16%, signal=32% |
| 239 | REACTOME\_METABOLISM\_OF\_RNA |  | 55 | 0.17 | 0.77 | 0.969 | 0.963 | 1.000 | 1370 | tags=29%, list=24%, signal=38% |
| 240 | TARTE\_PLASMA\_CELL\_VS\_PLASMABLAST\_DN |  | 180 | 0.15 | 0.77 | 1.000 | 0.960 | 1.000 | 932 | tags=20%, list=16%, signal=23% |
| 241 | BIOCARTA\_MAPK\_PATHWAY |  | 47 | 0.18 | 0.76 | 0.903 | 0.961 | 1.000 | 918 | tags=19%, list=16%, signal=23% |
| 242 | BIOCARTA\_CHREBP2\_PATHWAY |  | 19 | 0.23 | 0.76 | 0.844 | 0.959 | 1.000 | 1000 | tags=26%, list=17%, signal=32% |
| 243 | PENG\_GLUTAMINE\_DEPRIVATION\_DN |  | 46 | 0.18 | 0.75 | 0.926 | 0.967 | 1.000 | 1191 | tags=33%, list=21%, signal=41% |
| 244 | REACTOME\_SIGNALLING\_BY\_NGF |  | 102 | 0.15 | 0.75 | 0.939 | 0.967 | 1.000 | 467 | tags=10%, list=8%, signal=10% |
| 245 | TCGA\_GLIOBLASTOMA\_COPY\_NUMBER\_DN |  | 18 | 0.22 | 0.75 | 0.856 | 0.966 | 1.000 | 424 | tags=11%, list=7%, signal=12% |
| 246 | FERRANDO\_T\_ALL\_WITH\_MLL\_ENL\_FUSION\_DN |  | 45 | 0.17 | 0.74 | 0.944 | 0.970 | 1.000 | 480 | tags=13%, list=8%, signal=14% |
| 247 | REACTOME\_TOLL\_RECEPTOR\_CASCADES |  | 49 | 0.18 | 0.74 | 0.939 | 0.973 | 1.000 | 1077 | tags=27%, list=19%, signal=32% |
| 248 | CHNG\_MULTIPLE\_MYELOMA\_HYPERPLOID\_UP |  | 23 | 0.21 | 0.73 | 0.892 | 0.972 | 1.000 | 1508 | tags=35%, list=26%, signal=47% |
| 249 | GENTILE\_RESPONSE\_CLUSTER\_D3 |  | 26 | 0.19 | 0.73 | 0.894 | 0.973 | 1.000 | 859 | tags=23%, list=15%, signal=27% |
| 250 | BENPORATH\_ES\_1 |  | 135 | 0.14 | 0.72 | 1.000 | 0.981 | 1.000 | 1106 | tags=21%, list=19%, signal=26% |
| 251 | KEGG\_BUTANOATE\_METABOLISM |  | 18 | 0.22 | 0.71 | 0.869 | 0.985 | 1.000 | 587 | tags=17%, list=10%, signal=19% |
| 252 | KEGG\_NEUROTROPHIN\_SIGNALING\_PATHWAY |  | 56 | 0.16 | 0.71 | 0.929 | 0.982 | 1.000 | 1090 | tags=20%, list=19%, signal=24% |
| 253 | REACTOME\_SNRNP\_ASSEMBLY |  | 27 | 0.19 | 0.71 | 0.937 | 0.982 | 1.000 | 1370 | tags=37%, list=24%, signal=48% |
| 254 | REACTOME\_TRKA\_SIGNALLING\_FROM\_THE\_PLASMA\_MEMBRANE |  | 47 | 0.17 | 0.70 | 0.946 | 0.986 | 1.000 | 707 | tags=15%, list=12%, signal=17% |
| 255 | VANHARANTA\_UTERINE\_FIBROID\_WITH\_7Q\_DELETION\_UP |  | 41 | 0.16 | 0.70 | 0.945 | 0.984 | 1.000 | 521 | tags=15%, list=9%, signal=16% |
| 256 | MARKEY\_RB1\_ACUTE\_LOF\_DN |  | 104 | 0.14 | 0.67 | 1.000 | 0.999 | 1.000 | 1118 | tags=25%, list=20%, signal=31% |
| 257 | LAU\_APOPTOSIS\_CDKN2A\_UP |  | 26 | 0.18 | 0.66 | 0.930 | 1.000 | 1.000 | 465 | tags=12%, list=8%, signal=13% |
| 258 | REACTOME\_HIV1\_TRANSCRIPTION\_INITIATION |  | 20 | 0.19 | 0.66 | 0.918 | 1.000 | 1.000 | 601 | tags=15%, list=11%, signal=17% |
| 259 | WINNEPENNINCKX\_MELANOMA\_METASTASIS\_UP |  | 86 | 0.14 | 0.66 | 1.000 | 1.000 | 1.000 | 180 | tags=6%, list=3%, signal=6% |
| 260 | MOREAUX\_B\_LYMPHOCYTE\_MATURATION\_BY\_TACI\_DN |  | 26 | 0.18 | 0.65 | 0.928 | 0.998 | 1.000 | 3818 | tags=96%, list=67%, signal=288% |
| 261 | BOYAULT\_LIVER\_CANCER\_SUBCLASS\_G3\_UP |  | 110 | 0.13 | 0.65 | 1.000 | 0.999 | 1.000 | 983 | tags=19%, list=17%, signal=23% |
| 262 | KEGG\_P53\_SIGNALING\_PATHWAY |  | 39 | 0.16 | 0.64 | 0.985 | 0.997 | 1.000 | 645 | tags=13%, list=11%, signal=14% |
| 263 | BILD\_SRC\_ONCOGENIC\_SIGNATURE |  | 29 | 0.16 | 0.61 | 0.986 | 1.000 | 1.000 | 1154 | tags=28%, list=20%, signal=34% |
| 264 | REACTOME\_INFLUENZA\_VIRAL\_RNA\_TRANSCRIPTION\_AND\_REPLICATION |  | 29 | 0.16 | 0.60 | 0.980 | 1.000 | 1.000 | 3465 | tags=93%, list=61%, signal=235% |
| 265 | REACTOME\_PYRUVATE\_METABOLISM\_AND\_TCA\_CYCLE |  | 20 | 0.16 | 0.57 | 0.985 | 1.000 | 1.000 | 414 | tags=10%, list=7%, signal=11% |
| 266 | BIOCARTA\_EGF\_PATHWAY |  | 19 | 0.17 | 0.56 | 0.990 | 1.000 | 1.000 | 744 | tags=16%, list=13%, signal=18% |
| 267 | REACTOME\_PI3K\_AKT\_SIGNALLING |  | 18 | 0.17 | 0.55 | 0.977 | 1.000 | 1.000 | 4767 | tags=100%, list=83%, signal=597% |
| 268 | KEGG\_SPLICEOSOME |  | 66 | 0.13 | 0.55 | 1.000 | 1.000 | 1.000 | 1042 | tags=18%, list=18%, signal=22% |
| 269 | REACTOME\_ORC1\_REMOVAL\_FROM\_CHROMATIN |  | 36 | 0.13 | 0.49 | 0.992 | 1.000 | 1.000 | 4431 | tags=97%, list=77%, signal=428% |
| 270 | REACTOME\_M\_G1\_TRANSITION |  | 34 | 0.12 | 0.49 | 1.000 | 1.000 | 1.000 | 4431 | tags=97%, list=77%, signal=427% |
| 271 | REACTOME\_TRANSPORT\_OF\_MATURE\_MRNA\_DERIVED\_FROM\_AN\_INTRON\_CONTAINING\_TRANSCRIPT |  | 27 | 0.13 | 0.49 | 1.000 | 1.000 | 1.000 | 1370 | tags=37%, list=24%, signal=48% |
| 272 | REACTOME\_TRANSCRIPTION\_OF\_THE\_HIV\_GENOME |  | 31 | 0.12 | 0.46 | 1.000 | 1.000 | 1.000 | 5023 | tags=100%, list=88%, signal=813% |
| 273 | KRIGE\_RESPONSE\_TO\_TOSEDOSTAT\_24HR\_DN |  | 432 | 0.17 |  |  | 1.000 | 0.000 | 818 | tags=19%, list=14%, signal=20% |
| 274 | MONNIER\_POSTRADIATION\_TUMOR\_ESCAPE\_UP |  | 189 | 0.25 | 1.38 | 0.000 | 0.013 | 1.000 | 1107 | tags=33%, list=19%, signal=39% |
| 275 | SHEDDEN\_LUNG\_CANCER\_POOR\_SURVIVAL\_A6 |  | 224 | 0.18 |  |  | 1.000 | 0.000 | 1060 | tags=25%, list=19%, signal=30% |
| 276 | SHEDDEN\_LUNG\_CANCER\_GOOD\_SURVIVAL\_A5 |  | 15 | 0.59 | 1.89 | 0.009 | 0.000 | 0.412 | 791 | tags=53%, list=14%, signal=62% |
Table: Gene sets enriched in phenotype **LymphomaSeq (4 samples)**[plain text format]****

  
