# Supplementary material for: Comparative RNA-Seq and Microarray Analysis of Gene Expression Changes in B-Cell Lymphomas of Canis familiaris
Source: PLoS One. 2013 Apr 4;8(4):e61088. doi: 10.1371/journal.pone.0061088 (PMC3617154; doi:10.1371/journal.pone.0061088)
Supplement: Data File S2 — GSEA Results Files. (ZIP) [file pone.0061088.s005.zip › RNA-Seq/gsea_report_for_NormalSeq_v2.html]

Report for NormalSeq 1334779864086 [GSEA]

| GS  follow link to MSigDB | GS DETAILS | SIZE | ES | NES | NOM p-val | FDR q-val | FWER p-val | RANK AT MAX | LEADING EDGE || 1 | BOQUEST\_CD31PLUS\_VS\_CD31MINUS\_DN | Details ... | 88 | -0.75 | -2.44 | 0.000 | 0.000 | 0.000 | 705 | tags=63%, list=12%, signal=70% |
| 2 | HSA04060\_CYTOKINE\_CYTOKINE\_RECEPTOR\_INTERACTION | Details ... | 66 | -0.73 | -2.29 | 0.000 | 0.000 | 0.000 | 752 | tags=58%, list=13%, signal=66% |
| 3 | BAF57\_BT549\_UP | Details ... | 84 | -0.68 | -2.25 | 0.000 | 0.000 | 0.000 | 723 | tags=54%, list=13%, signal=60% |
| 4 | BOQUEST\_CD31PLUS\_VS\_CD31MINUS\_UP | Details ... | 236 | -0.63 | -2.19 | 0.000 | 0.000 | 0.000 | 991 | tags=47%, list=17%, signal=55% |
| 5 | LI\_FETAL\_VS\_WT\_KIDNEY\_UP | Details ... | 83 | -0.65 | -2.14 | 0.000 | 0.000 | 0.000 | 869 | tags=52%, list=15%, signal=60% |
| 6 | VERHAAK\_AML\_NPM1\_MUT\_VS\_WT\_UP | Details ... | 55 | -0.67 | -2.11 | 0.000 | 0.000 | 0.000 | 1102 | tags=58%, list=19%, signal=71% |
| 7 | CMV\_24HRS\_DN | Details ... | 36 | -0.72 | -2.10 | 0.000 | 0.000 | 0.000 | 990 | tags=69%, list=17%, signal=83% |
| 8 | SANA\_TNFA\_ENDOTHELIAL\_DN | Details ... | 29 | -0.75 | -2.10 | 0.000 | 0.000 | 0.000 | 668 | tags=62%, list=12%, signal=70% |
| 9 | IGLESIAS\_E2FMINUS\_UP | Details ... | 78 | -0.65 | -2.09 | 0.000 | 0.000 | 0.000 | 954 | tags=53%, list=17%, signal=62% |
| 10 | LAL\_KO\_3MO\_UP | Details ... | 21 | -0.79 | -2.09 | 0.000 | 0.000 | 0.001 | 500 | tags=57%, list=9%, signal=62% |
| 11 | LAL\_KO\_6MO\_UP | Details ... | 22 | -0.79 | -2.08 | 0.000 | 0.000 | 0.001 | 500 | tags=55%, list=9%, signal=60% |
| 12 | TAKEDA\_NUP8\_HOXA9\_8D\_DN | Details ... | 71 | -0.65 | -2.08 | 0.000 | 0.000 | 0.001 | 930 | tags=48%, list=16%, signal=56% |
| 13 | KANG\_TERT\_UP | Details ... | 25 | -0.76 | -2.08 | 0.000 | 0.000 | 0.001 | 229 | tags=32%, list=4%, signal=33% |
| 14 | TSA\_HEPATOMA\_CANCER\_UP | Details ... | 16 | -0.81 | -2.07 | 0.000 | 0.000 | 0.002 | 421 | tags=50%, list=7%, signal=54% |
| 15 | HEARTFAILURE\_ATRIA\_DN | Details ... | 56 | -0.66 | -2.05 | 0.000 | 0.000 | 0.004 | 834 | tags=48%, list=15%, signal=56% |
| 16 | CMV\_ALL\_DN | Details ... | 48 | -0.67 | -2.05 | 0.000 | 0.000 | 0.004 | 884 | tags=56%, list=15%, signal=66% |
| 17 | TAKEDA\_NUP8\_HOXA9\_10D\_DN | Details ... | 48 | -0.67 | -2.03 | 0.000 | 0.000 | 0.006 | 634 | tags=48%, list=11%, signal=53% |
| 18 | CROONQUIST\_IL6\_STROMA\_UP | Details ... | 15 | -0.82 | -2.03 | 0.000 | 0.000 | 0.007 | 642 | tags=67%, list=11%, signal=75% |
| 19 | ROS\_MOUSE\_AORTA\_DN | Details ... | 41 | -0.69 | -2.03 | 0.000 | 0.000 | 0.007 | 1206 | tags=61%, list=21%, signal=77% |
| 20 | HOHENKIRK\_MONOCYTE\_DEND\_UP | Details ... | 49 | -0.66 | -2.03 | 0.000 | 0.000 | 0.007 | 1064 | tags=53%, list=19%, signal=65% |
| 21 | ALCALAY\_AML\_NPMC\_UP |  | 56 | -0.64 | -2.00 | 0.000 | 0.001 | 0.012 | 644 | tags=41%, list=11%, signal=46% |
| 22 | TAKEDA\_NUP8\_HOXA9\_6H\_UP |  | 28 | -0.71 | -1.99 | 0.000 | 0.001 | 0.013 | 795 | tags=54%, list=14%, signal=62% |
| 23 | HSA04514\_CELL\_ADHESION\_MOLECULES |  | 42 | -0.66 | -1.99 | 0.000 | 0.001 | 0.017 | 1267 | tags=67%, list=22%, signal=85% |
| 24 | VERHAAK\_AML\_NPM1\_MUT\_VS\_WT\_DN |  | 77 | -0.61 | -1.99 | 0.000 | 0.001 | 0.018 | 860 | tags=47%, list=15%, signal=54% |
| 25 | SHEPARD\_POS\_REG\_OF\_CELL\_PROLIFERATION |  | 31 | -0.70 | -1.98 | 0.000 | 0.001 | 0.021 | 728 | tags=52%, list=13%, signal=59% |
| 26 | HSA04512\_ECM\_RECEPTOR\_INTERACTION |  | 30 | -0.70 | -1.98 | 0.000 | 0.001 | 0.021 | 1218 | tags=73%, list=21%, signal=93% |
| 27 | TAVOR\_CEBP\_UP |  | 24 | -0.73 | -1.96 | 0.000 | 0.001 | 0.022 | 595 | tags=54%, list=10%, signal=60% |
| 28 | ET743\_HELA\_UP |  | 24 | -0.71 | -1.95 | 0.000 | 0.001 | 0.023 | 856 | tags=58%, list=15%, signal=68% |
| 29 | HADDAD\_HSC\_CD7\_UP |  | 27 | -0.70 | -1.95 | 0.000 | 0.001 | 0.028 | 489 | tags=48%, list=9%, signal=52% |
| 30 | TARTE\_MATURE\_PC |  | 117 | -0.57 | -1.94 | 0.000 | 0.001 | 0.031 | 746 | tags=36%, list=13%, signal=40% |
| 31 | HADDAD\_CD45CD7\_PLUS\_VS\_MINUS\_UP |  | 27 | -0.70 | -1.94 | 0.000 | 0.001 | 0.037 | 489 | tags=48%, list=9%, signal=52% |
| 32 | BASSO\_HCL\_DIFF |  | 36 | -0.66 | -1.93 | 0.000 | 0.001 | 0.043 | 1239 | tags=69%, list=22%, signal=88% |
| 33 | HSA04610\_COMPLEMENT\_AND\_COAGULATION\_CASCADES |  | 15 | -0.78 | -1.91 | 0.000 | 0.002 | 0.064 | 1036 | tags=87%, list=18%, signal=106% |
| 34 | JECHLINGER\_EMT\_UP |  | 28 | -0.67 | -1.89 | 0.000 | 0.003 | 0.095 | 671 | tags=46%, list=12%, signal=52% |
| 35 | ROSS\_CBF\_MYH |  | 30 | -0.67 | -1.89 | 0.000 | 0.003 | 0.097 | 1169 | tags=67%, list=20%, signal=83% |
| 36 | TGFBETA\_ALL\_UP |  | 31 | -0.66 | -1.89 | 0.000 | 0.003 | 0.100 | 678 | tags=48%, list=12%, signal=55% |
| 37 | LEE\_E2F1\_UP |  | 26 | -0.69 | -1.88 | 0.000 | 0.003 | 0.112 | 1313 | tags=69%, list=23%, signal=89% |
| 38 | IRITANI\_ADPROX\_VASC |  | 64 | -0.60 | -1.88 | 0.000 | 0.003 | 0.112 | 812 | tags=42%, list=14%, signal=49% |
| 39 | AGEING\_KIDNEY\_SPECIFIC\_UP |  | 71 | -0.59 | -1.88 | 0.000 | 0.003 | 0.115 | 1117 | tags=49%, list=20%, signal=60% |
| 40 | IDX\_TSA\_DN\_CLUSTER2 |  | 35 | -0.64 | -1.88 | 0.000 | 0.003 | 0.130 | 1189 | tags=54%, list=21%, signal=68% |
| 41 | LEE\_ACOX1\_UP |  | 19 | -0.73 | -1.87 | 0.001 | 0.004 | 0.154 | 795 | tags=53%, list=14%, signal=61% |
| 42 | CELL\_ADHESION\_MOLECULE\_ACTIVITY |  | 31 | -0.65 | -1.87 | 0.000 | 0.004 | 0.156 | 793 | tags=45%, list=14%, signal=52% |
| 43 | HSIAO\_LIVER\_SPECIFIC\_GENES |  | 58 | -0.60 | -1.86 | 0.000 | 0.004 | 0.173 | 720 | tags=38%, list=13%, signal=43% |
| 44 | PARP\_KO\_UP |  | 17 | -0.73 | -1.86 | 0.000 | 0.004 | 0.173 | 593 | tags=53%, list=10%, signal=59% |
| 45 | TAKEDA\_NUP8\_HOXA9\_8D\_UP |  | 40 | -0.63 | -1.85 | 0.001 | 0.005 | 0.189 | 806 | tags=45%, list=14%, signal=52% |
| 46 | HTERT\_DN |  | 22 | -0.69 | -1.85 | 0.001 | 0.005 | 0.195 | 718 | tags=55%, list=13%, signal=62% |
| 47 | HSA04640\_HEMATOPOIETIC\_CELL\_LINEAGE |  | 32 | -0.65 | -1.85 | 0.000 | 0.005 | 0.198 | 823 | tags=59%, list=14%, signal=69% |
| 48 | VEGF\_MMMEC\_ALL\_UP |  | 32 | -0.64 | -1.85 | 0.000 | 0.005 | 0.202 | 1309 | tags=66%, list=23%, signal=85% |
| 49 | NI2\_MOUSE\_DN |  | 19 | -0.72 | -1.84 | 0.000 | 0.005 | 0.202 | 416 | tags=32%, list=7%, signal=34% |
| 50 | BRCA\_BRCA1\_NEG |  | 46 | -0.61 | -1.84 | 0.000 | 0.005 | 0.211 | 1000 | tags=48%, list=17%, signal=57% |
| 51 | EMT\_UP |  | 30 | -0.65 | -1.84 | 0.001 | 0.005 | 0.231 | 671 | tags=47%, list=12%, signal=53% |
| 52 | HTERT\_UP |  | 30 | -0.65 | -1.83 | 0.000 | 0.005 | 0.236 | 636 | tags=40%, list=11%, signal=45% |
| 53 | AGEING\_BRAIN\_UP |  | 86 | -0.56 | -1.83 | 0.000 | 0.005 | 0.246 | 1163 | tags=47%, list=20%, signal=57% |
| 54 | DAC\_PANC\_UP |  | 90 | -0.56 | -1.83 | 0.000 | 0.005 | 0.248 | 620 | tags=31%, list=11%, signal=34% |
| 55 | POD1\_KO\_DN |  | 309 | -0.52 | -1.83 | 0.000 | 0.005 | 0.251 | 1470 | tags=47%, list=26%, signal=60% |
| 56 | LEE\_CIP\_UP |  | 15 | -0.74 | -1.83 | 0.003 | 0.006 | 0.268 | 799 | tags=67%, list=14%, signal=77% |
| 57 | HSA01430\_CELL\_COMMUNICATION |  | 20 | -0.71 | -1.83 | 0.000 | 0.006 | 0.273 | 860 | tags=60%, list=15%, signal=70% |
| 58 | NAKAJIMA\_MCSMBP\_MAST |  | 19 | -0.69 | -1.82 | 0.004 | 0.006 | 0.305 | 826 | tags=58%, list=14%, signal=67% |
| 59 | GERY\_CEBP\_TARGETS |  | 46 | -0.60 | -1.81 | 0.000 | 0.007 | 0.323 | 1243 | tags=50%, list=22%, signal=63% |
| 60 | LINDSTEDT\_DEND\_DN |  | 28 | -0.65 | -1.81 | 0.001 | 0.007 | 0.323 | 1206 | tags=54%, list=21%, signal=68% |
| 61 | ADIP\_DIFF\_CLUSTER1 |  | 24 | -0.67 | -1.81 | 0.000 | 0.007 | 0.323 | 983 | tags=63%, list=17%, signal=75% |
| 62 | ELONGINA\_KO\_UP |  | 70 | -0.56 | -1.81 | 0.001 | 0.006 | 0.325 | 1181 | tags=49%, list=21%, signal=60% |
| 63 | CARIES\_PULP\_DN |  | 20 | -0.69 | -1.81 | 0.001 | 0.006 | 0.325 | 718 | tags=50%, list=13%, signal=57% |
| 64 | GAY\_YY1\_UP |  | 49 | -0.59 | -1.81 | 0.000 | 0.007 | 0.358 | 1316 | tags=53%, list=23%, signal=68% |
| 65 | STOSSI\_ER\_UP |  | 15 | -0.74 | -1.80 | 0.003 | 0.007 | 0.376 | 623 | tags=47%, list=11%, signal=52% |
| 66 | CMV-UV\_HCMV\_6HRS\_DN |  | 41 | -0.60 | -1.80 | 0.000 | 0.008 | 0.394 | 479 | tags=32%, list=8%, signal=34% |
| 67 | ADIP\_VS\_PREADIP\_DN |  | 17 | -0.71 | -1.80 | 0.003 | 0.008 | 0.396 | 990 | tags=65%, list=17%, signal=78% |
| 68 | JISON\_SICKLE\_CELL |  | 16 | -0.72 | -1.80 | 0.003 | 0.008 | 0.396 | 768 | tags=50%, list=13%, signal=58% |
| 69 | LINDSTEDT\_DEND\_8H\_VS\_48H\_DN |  | 29 | -0.63 | -1.79 | 0.001 | 0.008 | 0.411 | 877 | tags=45%, list=15%, signal=53% |
| 70 | TGFBETA\_EARLY\_UP |  | 17 | -0.71 | -1.79 | 0.000 | 0.008 | 0.414 | 642 | tags=59%, list=11%, signal=66% |
| 71 | BECKER\_TAMOXIFEN\_RESISTANT\_DN |  | 21 | -0.67 | -1.79 | 0.002 | 0.008 | 0.419 | 619 | tags=48%, list=11%, signal=53% |
| 72 | CHIARETTI\_T\_ALL\_DIFF |  | 104 | -0.54 | -1.79 | 0.000 | 0.008 | 0.421 | 1273 | tags=46%, list=22%, signal=58% |
| 73 | NI2\_MOUSE\_UP |  | 21 | -0.68 | -1.79 | 0.001 | 0.008 | 0.449 | 715 | tags=52%, list=12%, signal=60% |
| 74 | HCC\_SURVIVAL\_GOOD\_VS\_POOR\_UP |  | 31 | -0.64 | -1.79 | 0.000 | 0.008 | 0.450 | 1108 | tags=45%, list=19%, signal=56% |
| 75 | ICHIBA\_GVHD |  | 95 | -0.54 | -1.79 | 0.000 | 0.008 | 0.452 | 1100 | tags=43%, list=19%, signal=53% |
| 76 | HOFFMANN\_BIVSBII\_IMVM |  | 37 | -0.61 | -1.78 | 0.002 | 0.009 | 0.487 | 475 | tags=32%, list=8%, signal=35% |
| 77 | ET743\_SARCOMA\_72HRS\_UP |  | 22 | -0.67 | -1.77 | 0.001 | 0.009 | 0.522 | 1059 | tags=59%, list=19%, signal=72% |
| 78 | CMV\_HCMV\_TIMECOURSE\_24HRS\_DN |  | 15 | -0.73 | -1.77 | 0.003 | 0.009 | 0.524 | 443 | tags=53%, list=8%, signal=58% |
| 79 | INFLAMMATORY\_RESPONSE\_PATHWAY |  | 17 | -0.71 | -1.76 | 0.001 | 0.011 | 0.606 | 860 | tags=59%, list=15%, signal=69% |
| 80 | CHIARETTI\_T\_ALL |  | 94 | -0.53 | -1.76 | 0.000 | 0.011 | 0.609 | 1012 | tags=41%, list=18%, signal=50% |
| 81 | DIAB\_NEPH\_DN |  | 169 | -0.51 | -1.75 | 0.000 | 0.012 | 0.641 | 1104 | tags=38%, list=19%, signal=46% |
| 82 | IRS1\_KO\_ADIP\_DN |  | 40 | -0.59 | -1.75 | 0.001 | 0.012 | 0.655 | 964 | tags=48%, list=17%, signal=57% |
| 83 | VEGF\_MMMEC\_3HRS\_UP |  | 22 | -0.66 | -1.75 | 0.001 | 0.012 | 0.673 | 1255 | tags=64%, list=22%, signal=81% |
| 84 | HEARTFAILURE\_VENTRICLE\_DN |  | 31 | -0.62 | -1.75 | 0.001 | 0.012 | 0.673 | 618 | tags=29%, list=11%, signal=32% |
| 85 | HSA04510\_FOCAL\_ADHESION |  | 87 | -0.53 | -1.75 | 0.000 | 0.013 | 0.695 | 1163 | tags=39%, list=20%, signal=48% |
| 86 | BRCA1\_OVEREXP\_PROSTATE\_UP |  | 64 | -0.55 | -1.75 | 0.000 | 0.013 | 0.698 | 1148 | tags=45%, list=20%, signal=56% |
| 87 | ADIP\_HUMAN\_UP |  | 18 | -0.68 | -1.74 | 0.001 | 0.013 | 0.715 | 613 | tags=50%, list=11%, signal=56% |
| 88 | CELL\_SURFACE\_RECEPTOR\_LINKED\_SIGNAL\_TRANSDUCTION |  | 41 | -0.59 | -1.74 | 0.000 | 0.014 | 0.729 | 715 | tags=44%, list=12%, signal=50% |
| 89 | BRUNO\_IL3\_DN |  | 26 | -0.63 | -1.74 | 0.004 | 0.014 | 0.748 | 856 | tags=42%, list=15%, signal=50% |
| 90 | CALRES\_RHESUS\_UP |  | 25 | -0.63 | -1.74 | 0.004 | 0.014 | 0.754 | 634 | tags=44%, list=11%, signal=49% |
| 91 | AGEING\_KIDNEY\_UP |  | 138 | -0.51 | -1.73 | 0.000 | 0.015 | 0.763 | 1218 | tags=38%, list=21%, signal=48% |
| 92 | LEI\_MYB\_REGULATED\_GENES |  | 119 | -0.51 | -1.73 | 0.000 | 0.016 | 0.787 | 990 | tags=39%, list=17%, signal=46% |
| 93 | TAKEDA\_NUP8\_HOXA9\_16D\_DN |  | 86 | -0.52 | -1.73 | 0.000 | 0.016 | 0.790 | 655 | tags=23%, list=11%, signal=26% |
| 94 | ADIP\_VS\_FIBRO\_UP |  | 16 | -0.69 | -1.73 | 0.003 | 0.016 | 0.792 | 407 | tags=31%, list=7%, signal=34% |
| 95 | CELL\_ADHESION |  | 53 | -0.56 | -1.72 | 0.002 | 0.017 | 0.817 | 793 | tags=40%, list=14%, signal=46% |
| 96 | HOUSTIS\_ROS |  | 15 | -0.70 | -1.71 | 0.004 | 0.019 | 0.843 | 1140 | tags=67%, list=20%, signal=83% |
| 97 | FLECHNER\_KIDNEY\_TRANSPLANT\_REJECTION\_UP |  | 38 | -0.58 | -1.71 | 0.001 | 0.019 | 0.853 | 534 | tags=34%, list=9%, signal=37% |
| 98 | ZHAN\_MM\_MOLECULAR\_CLASSI\_UP |  | 16 | -0.68 | -1.70 | 0.004 | 0.022 | 0.882 | 297 | tags=25%, list=5%, signal=26% |
| 99 | LEE\_DENA\_UP |  | 22 | -0.63 | -1.70 | 0.001 | 0.022 | 0.882 | 1313 | tags=59%, list=23%, signal=76% |
| 100 | TNFALPHA\_ALL\_UP |  | 33 | -0.60 | -1.70 | 0.000 | 0.021 | 0.882 | 846 | tags=45%, list=15%, signal=53% |
| 101 | FLECHNER\_KIDNEY\_TRANSPLANT\_REJECTION\_DN |  | 229 | -0.49 | -1.70 | 0.000 | 0.021 | 0.883 | 1402 | tags=40%, list=24%, signal=51% |
| 102 | HSC\_LTHSC\_FETAL |  | 88 | -0.51 | -1.69 | 0.000 | 0.023 | 0.899 | 1041 | tags=39%, list=18%, signal=46% |
| 103 | RADMACHER\_AMLNORMALKARYTYPE\_SIG |  | 24 | -0.62 | -1.69 | 0.001 | 0.022 | 0.899 | 838 | tags=38%, list=15%, signal=44% |
| 104 | HSC\_LTHSC\_SHARED |  | 88 | -0.51 | -1.69 | 0.001 | 0.023 | 0.908 | 1041 | tags=39%, list=18%, signal=46% |
| 105 | MYOD\_NIH3T3\_DN |  | 25 | -0.62 | -1.68 | 0.005 | 0.024 | 0.921 | 1309 | tags=56%, list=23%, signal=72% |
| 106 | IRITANI\_ADPROX\_DN |  | 30 | -0.60 | -1.68 | 0.006 | 0.024 | 0.924 | 1209 | tags=53%, list=21%, signal=67% |
| 107 | HINATA\_NFKB\_UP |  | 39 | -0.57 | -1.68 | 0.005 | 0.025 | 0.928 | 928 | tags=38%, list=16%, signal=46% |
| 108 | HSA04520\_ADHERENS\_JUNCTION |  | 25 | -0.62 | -1.68 | 0.005 | 0.025 | 0.932 | 789 | tags=40%, list=14%, signal=46% |
| 109 | CORDERO\_KRAS\_KD\_VS\_CONTROL\_UP |  | 33 | -0.59 | -1.68 | 0.003 | 0.025 | 0.934 | 675 | tags=36%, list=12%, signal=41% |
| 110 | IDX\_TSA\_DN\_CLUSTER1 |  | 16 | -0.67 | -1.68 | 0.006 | 0.025 | 0.935 | 1313 | tags=69%, list=23%, signal=89% |
| 111 | NUCLEAR\_RECEPTORS |  | 15 | -0.68 | -1.68 | 0.006 | 0.025 | 0.939 | 1299 | tags=60%, list=23%, signal=77% |
| 112 | KANG\_TERT\_DN |  | 26 | -0.61 | -1.68 | 0.006 | 0.025 | 0.939 | 975 | tags=50%, list=17%, signal=60% |
| 113 | RAS\_ONCOGENIC\_SIGNATURE |  | 93 | -0.50 | -1.67 | 0.001 | 0.027 | 0.952 | 1325 | tags=39%, list=23%, signal=50% |
| 114 | LEE\_MYC\_DN |  | 22 | -0.62 | -1.67 | 0.007 | 0.027 | 0.954 | 598 | tags=36%, list=10%, signal=40% |
| 115 | RUTELLA\_HEPATGFSNDCS\_UP |  | 61 | -0.53 | -1.67 | 0.000 | 0.027 | 0.961 | 875 | tags=39%, list=15%, signal=46% |
| 116 | KUMAR\_HOXA\_DIFF |  | 149 | -0.49 | -1.67 | 0.000 | 0.027 | 0.961 | 1100 | tags=40%, list=19%, signal=48% |
| 117 | JECHLINGER\_EMT\_DN |  | 20 | -0.63 | -1.67 | 0.008 | 0.028 | 0.965 | 718 | tags=50%, list=13%, signal=57% |
| 118 | LE\_MYELIN\_DN |  | 32 | -0.58 | -1.66 | 0.004 | 0.029 | 0.973 | 1146 | tags=47%, list=20%, signal=58% |
| 119 | IDX\_TSA\_UP\_CLUSTER2 |  | 31 | -0.58 | -1.66 | 0.002 | 0.030 | 0.976 | 578 | tags=39%, list=10%, signal=43% |
| 120 | GENOTOXINS\_4HRS\_DISCR |  | 15 | -0.67 | -1.66 | 0.016 | 0.030 | 0.982 | 365 | tags=33%, list=6%, signal=36% |
| 121 | NAB\_LUNG\_DN |  | 21 | -0.63 | -1.65 | 0.007 | 0.031 | 0.984 | 639 | tags=38%, list=11%, signal=43% |
| 122 | INOS\_ALL\_DN |  | 37 | -0.55 | -1.65 | 0.005 | 0.031 | 0.986 | 424 | tags=24%, list=7%, signal=26% |
| 123 | PASSERINI\_ADHESION |  | 20 | -0.63 | -1.65 | 0.014 | 0.031 | 0.986 | 937 | tags=50%, list=16%, signal=60% |
| 124 | ADIP\_DIFF\_UP |  | 30 | -0.58 | -1.65 | 0.011 | 0.032 | 0.987 | 1267 | tags=47%, list=22%, signal=60% |
| 125 | ADIP\_DIFF\_CLUSTER2 |  | 17 | -0.65 | -1.65 | 0.004 | 0.032 | 0.987 | 622 | tags=41%, list=11%, signal=46% |
| 126 | HSA05218\_MELANOMA |  | 28 | -0.60 | -1.64 | 0.006 | 0.033 | 0.988 | 540 | tags=29%, list=9%, signal=31% |
| 127 | FLECHNER\_KIDNEY\_TRANSPLANT\_WELL\_UP |  | 260 | -0.47 | -1.64 | 0.000 | 0.033 | 0.989 | 1506 | tags=42%, list=26%, signal=54% |
| 128 | ADIP\_VS\_PREADIP\_UP |  | 15 | -0.67 | -1.64 | 0.010 | 0.035 | 0.991 | 407 | tags=33%, list=7%, signal=36% |
| 129 | YAGI\_AML\_PROG\_FAB |  | 74 | -0.51 | -1.64 | 0.000 | 0.036 | 0.992 | 623 | tags=32%, list=11%, signal=36% |
| 130 | TGF\_BETA\_SIGNALING\_PATHWAY |  | 19 | -0.64 | -1.63 | 0.014 | 0.036 | 0.992 | 1291 | tags=53%, list=23%, signal=68% |
| 131 | RUTELLA\_HEMATOGFSNDCS\_DIFF |  | 284 | -0.47 | -1.63 | 0.000 | 0.036 | 0.992 | 1243 | tags=38%, list=22%, signal=46% |
| 132 | ZHAN\_MMPC\_SIMAL |  | 25 | -0.60 | -1.63 | 0.007 | 0.036 | 0.992 | 1245 | tags=56%, list=22%, signal=71% |
| 133 | HALMOS\_CEBP\_UP |  | 21 | -0.61 | -1.63 | 0.009 | 0.036 | 0.992 | 1421 | tags=62%, list=25%, signal=82% |
| 134 | HSA04360\_AXON\_GUIDANCE |  | 36 | -0.56 | -1.63 | 0.005 | 0.037 | 0.993 | 1163 | tags=44%, list=20%, signal=55% |
| 135 | TAKEDA\_NUP8\_HOXA9\_10D\_UP |  | 56 | -0.52 | -1.63 | 0.005 | 0.038 | 0.993 | 795 | tags=39%, list=14%, signal=45% |
| 136 | PASSERINI\_EM |  | 15 | -0.67 | -1.63 | 0.004 | 0.038 | 0.993 | 195 | tags=27%, list=3%, signal=28% |
| 137 | NAKAJIMA\_MCS\_UP |  | 33 | -0.57 | -1.62 | 0.003 | 0.039 | 0.994 | 760 | tags=42%, list=13%, signal=49% |
| 138 | LVAD\_HEARTFAILURE\_DN |  | 15 | -0.66 | -1.62 | 0.010 | 0.039 | 0.994 | 675 | tags=47%, list=12%, signal=53% |
| 139 | ATRIA\_UP |  | 52 | -0.53 | -1.62 | 0.003 | 0.040 | 0.994 | 585 | tags=31%, list=10%, signal=34% |
| 140 | FRASOR\_ER\_DN |  | 19 | -0.62 | -1.62 | 0.013 | 0.040 | 0.995 | 804 | tags=53%, list=14%, signal=61% |
| 141 | BRENTANI\_CELL\_ADHESION |  | 37 | -0.56 | -1.62 | 0.009 | 0.040 | 0.996 | 1218 | tags=49%, list=21%, signal=61% |
| 142 | CARIES\_PULP\_UP |  | 86 | -0.49 | -1.62 | 0.000 | 0.040 | 0.996 | 1152 | tags=36%, list=20%, signal=44% |
| 143 | ET743\_SARCOMA\_UP |  | 27 | -0.58 | -1.61 | 0.018 | 0.042 | 0.998 | 1059 | tags=44%, list=19%, signal=54% |
| 144 | AGED\_MOUSE\_NEOCORTEX\_UP |  | 36 | -0.55 | -1.61 | 0.009 | 0.043 | 0.998 | 1533 | tags=50%, list=27%, signal=68% |
| 145 | HSC\_LTHSC\_ADULT |  | 108 | -0.48 | -1.61 | 0.000 | 0.044 | 0.999 | 1041 | tags=34%, list=18%, signal=41% |
| 146 | HSA04670\_LEUKOCYTE\_TRANSENDOTHELIAL\_MIGRATION |  | 52 | -0.52 | -1.61 | 0.003 | 0.044 | 0.999 | 1309 | tags=37%, list=23%, signal=47% |
| 147 | HDACI\_COLON\_TSA\_UP |  | 40 | -0.54 | -1.61 | 0.006 | 0.044 | 0.999 | 1153 | tags=40%, list=20%, signal=50% |
| 148 | TNFALPHA\_4HRS\_UP |  | 17 | -0.64 | -1.61 | 0.013 | 0.044 | 0.999 | 679 | tags=47%, list=12%, signal=53% |
| 149 | UEDA\_MOUSE\_SCN |  | 35 | -0.56 | -1.61 | 0.014 | 0.044 | 0.999 | 1439 | tags=51%, list=25%, signal=68% |
| 150 | UV-CMV\_UNIQUE\_HCMV\_6HRS\_DN |  | 33 | -0.56 | -1.60 | 0.009 | 0.045 | 0.999 | 479 | tags=24%, list=8%, signal=26% |
| 151 | MANALO\_HYPOXIA\_UP |  | 38 | -0.55 | -1.60 | 0.005 | 0.046 | 0.999 | 896 | tags=45%, list=16%, signal=53% |
| 152 | JISON\_SICKLECELL\_DIFF |  | 130 | -0.47 | -1.60 | 0.000 | 0.047 | 0.999 | 1033 | tags=29%, list=18%, signal=35% |
| 153 | TAKEDA\_NUP8\_HOXA9\_3D\_UP |  | 55 | -0.52 | -1.59 | 0.003 | 0.048 | 1.000 | 952 | tags=36%, list=17%, signal=43% |
| 154 | EMT\_DN |  | 24 | -0.58 | -1.59 | 0.018 | 0.048 | 1.000 | 718 | tags=46%, list=13%, signal=52% |
| 155 | SANSOM\_APC\_4\_DN |  | 21 | -0.60 | -1.59 | 0.019 | 0.048 | 1.000 | 441 | tags=38%, list=8%, signal=41% |
| 156 | HSA04530\_TIGHT\_JUNCTION |  | 45 | -0.53 | -1.59 | 0.006 | 0.051 | 1.000 | 723 | tags=33%, list=13%, signal=38% |
| 157 | UVC\_HIGH\_D6\_DN |  | 17 | -0.63 | -1.59 | 0.010 | 0.051 | 1.000 | 1154 | tags=47%, list=20%, signal=59% |
| 158 | BRCA1\_OVEREXP\_UP |  | 81 | -0.49 | -1.59 | 0.003 | 0.051 | 1.000 | 1364 | tags=46%, list=24%, signal=59% |
| 159 | PROLIFERATION\_GENES |  | 126 | -0.47 | -1.59 | 0.001 | 0.051 | 1.000 | 728 | tags=25%, list=13%, signal=28% |
| 160 | HADDAD\_HSC\_CD7\_DN |  | 35 | -0.55 | -1.58 | 0.013 | 0.052 | 1.000 | 770 | tags=40%, list=13%, signal=46% |
| 161 | NADLER\_OBESITY\_UP |  | 30 | -0.56 | -1.58 | 0.021 | 0.052 | 1.000 | 1414 | tags=53%, list=25%, signal=70% |
| 162 | LEE\_MYC\_E2F1\_UP |  | 18 | -0.61 | -1.58 | 0.018 | 0.052 | 1.000 | 1313 | tags=56%, list=23%, signal=72% |
| 163 | HADDAD\_CD45CD7\_PLUS\_VS\_MINUS\_DN |  | 35 | -0.55 | -1.58 | 0.008 | 0.054 | 1.000 | 770 | tags=40%, list=13%, signal=46% |
| 164 | HYPOXIA\_REVIEW |  | 32 | -0.55 | -1.58 | 0.014 | 0.055 | 1.000 | 923 | tags=41%, list=16%, signal=48% |
| 165 | TNFALPHA\_ADIP\_DN |  | 25 | -0.58 | -1.58 | 0.012 | 0.055 | 1.000 | 642 | tags=32%, list=11%, signal=36% |
| 166 | CELL\_ADHESION\_RECEPTOR\_ACTIVITY |  | 16 | -0.63 | -1.58 | 0.022 | 0.055 | 1.000 | 1416 | tags=63%, list=25%, signal=83% |
| 167 | GH\_EXOGENOUS\_ANY\_DN |  | 32 | -0.55 | -1.57 | 0.014 | 0.058 | 1.000 | 1239 | tags=44%, list=22%, signal=56% |
| 168 | HSA04660\_T\_CELL\_RECEPTOR\_SIGNALING\_PATHWAY |  | 52 | -0.50 | -1.57 | 0.008 | 0.059 | 1.000 | 1083 | tags=35%, list=19%, signal=42% |
| 169 | CREB\_BRAIN\_8WKS\_UP |  | 20 | -0.61 | -1.57 | 0.022 | 0.059 | 1.000 | 1533 | tags=60%, list=27%, signal=82% |
| 170 | WIELAND\_HEPATITIS\_B\_INDUCED |  | 44 | -0.53 | -1.57 | 0.007 | 0.059 | 1.000 | 545 | tags=27%, list=10%, signal=30% |
| 171 | TAKEDA\_NUP8\_HOXA9\_6H\_DN |  | 15 | -0.63 | -1.57 | 0.025 | 0.059 | 1.000 | 965 | tags=53%, list=17%, signal=64% |
| 172 | UVB\_SCC\_UP |  | 44 | -0.52 | -1.57 | 0.016 | 0.059 | 1.000 | 2250 | tags=73%, list=39%, signal=119% |
| 173 | TARTE\_PC |  | 49 | -0.51 | -1.57 | 0.014 | 0.059 | 1.000 | 1194 | tags=51%, list=21%, signal=64% |
| 174 | LEE\_MYC\_TGFA\_UP |  | 19 | -0.60 | -1.56 | 0.025 | 0.062 | 1.000 | 1313 | tags=63%, list=23%, signal=82% |
| 175 | SANSOM\_APC\_5\_DN |  | 142 | -0.46 | -1.56 | 0.000 | 0.062 | 1.000 | 1129 | tags=35%, list=20%, signal=42% |
| 176 | HSA03320\_PPAR\_SIGNALING\_PATHWAY |  | 28 | -0.56 | -1.56 | 0.019 | 0.062 | 1.000 | 767 | tags=39%, list=13%, signal=45% |
| 177 | TPA\_SENS\_LATE\_UP |  | 22 | -0.59 | -1.56 | 0.025 | 0.063 | 1.000 | 597 | tags=32%, list=10%, signal=35% |
| 178 | HOHENKIRK\_MONOCYTE\_DEND\_DN |  | 57 | -0.50 | -1.55 | 0.012 | 0.063 | 1.000 | 1200 | tags=40%, list=21%, signal=51% |
| 179 | GOLDRATH\_MEMORY |  | 28 | -0.56 | -1.55 | 0.024 | 0.063 | 1.000 | 1075 | tags=46%, list=19%, signal=57% |
| 180 | HOFMANN\_MDS\_CD34\_LOW\_AND\_HIGH\_RISK |  | 17 | -0.62 | -1.55 | 0.020 | 0.065 | 1.000 | 1083 | tags=47%, list=19%, signal=58% |
| 181 | IL1\_CORNEA\_UP |  | 22 | -0.58 | -1.55 | 0.023 | 0.065 | 1.000 | 622 | tags=41%, list=11%, signal=46% |
| 182 | OXSTRESS\_RPE\_HNETBH\_DN |  | 18 | -0.60 | -1.55 | 0.032 | 0.066 | 1.000 | 821 | tags=39%, list=14%, signal=45% |
| 183 | GUO\_HEX\_DN |  | 26 | -0.56 | -1.55 | 0.019 | 0.067 | 1.000 | 1737 | tags=62%, list=30%, signal=88% |
| 184 | ROSS\_PML\_RAR |  | 26 | -0.56 | -1.54 | 0.016 | 0.067 | 1.000 | 1354 | tags=54%, list=24%, signal=70% |
| 185 | ZHAN\_MULTIPLE\_MYELOMA\_VS\_NORMAL\_DN |  | 15 | -0.62 | -1.54 | 0.034 | 0.068 | 1.000 | 1100 | tags=60%, list=19%, signal=74% |
| 186 | HYPOPHYSECTOMY\_RAT\_DN |  | 22 | -0.59 | -1.54 | 0.022 | 0.069 | 1.000 | 1838 | tags=68%, list=32%, signal=100% |
| 187 | ALCALAY\_AML\_NPMC\_DN |  | 71 | -0.48 | -1.54 | 0.005 | 0.069 | 1.000 | 558 | tags=25%, list=10%, signal=28% |
| 188 | TNFALPHA\_30MIN\_UP |  | 18 | -0.61 | -1.54 | 0.027 | 0.069 | 1.000 | 1305 | tags=56%, list=23%, signal=72% |
| 189 | RUIZ\_TENASCIN\_TARGETS |  | 26 | -0.56 | -1.54 | 0.022 | 0.069 | 1.000 | 723 | tags=35%, list=13%, signal=39% |
| 190 | EGF\_HDMEC\_UP |  | 25 | -0.56 | -1.54 | 0.025 | 0.069 | 1.000 | 1891 | tags=72%, list=33%, signal=107% |
| 191 | VEGF\_MMMEC\_6HRS\_UP |  | 16 | -0.61 | -1.54 | 0.025 | 0.070 | 1.000 | 1309 | tags=69%, list=23%, signal=89% |
| 192 | CIRCADIAN\_EXERCISE |  | 17 | -0.61 | -1.53 | 0.029 | 0.073 | 1.000 | 1273 | tags=59%, list=22%, signal=75% |
| 193 | H2O2\_CSBRESCUED\_C1\_UP |  | 15 | -0.61 | -1.53 | 0.040 | 0.073 | 1.000 | 678 | tags=40%, list=12%, signal=45% |
| 194 | PASSERINI\_SIGNAL |  | 130 | -0.45 | -1.53 | 0.004 | 0.074 | 1.000 | 1523 | tags=40%, list=27%, signal=53% |
| 195 | APPEL\_IMATINIB\_UP |  | 20 | -0.58 | -1.53 | 0.027 | 0.076 | 1.000 | 1414 | tags=55%, list=25%, signal=73% |
| 196 | WELCSH\_BRCA\_UP |  | 19 | -0.59 | -1.52 | 0.038 | 0.081 | 1.000 | 856 | tags=47%, list=15%, signal=56% |
| 197 | BREAST\_CANCER\_ESTROGEN\_SIGNALING |  | 30 | -0.53 | -1.52 | 0.029 | 0.082 | 1.000 | 1414 | tags=50%, list=25%, signal=66% |
| 198 | HSA04080\_NEUROACTIVE\_LIGAND\_RECEPTOR\_INTERACTION |  | 26 | -0.55 | -1.52 | 0.028 | 0.081 | 1.000 | 1137 | tags=54%, list=20%, signal=67% |
| 199 | ECMPATHWAY |  | 16 | -0.61 | -1.52 | 0.045 | 0.081 | 1.000 | 1305 | tags=50%, list=23%, signal=65% |
| 200 | CPR\_NULL\_LIVER\_UP |  | 15 | -0.62 | -1.52 | 0.032 | 0.081 | 1.000 | 254 | tags=33%, list=4%, signal=35% |
| 201 | CARIES\_PULP\_HIGH\_UP |  | 32 | -0.53 | -1.51 | 0.018 | 0.083 | 1.000 | 752 | tags=28%, list=13%, signal=32% |
| 202 | HDACI\_COLON\_BUT12HRS\_UP |  | 18 | -0.59 | -1.51 | 0.035 | 0.085 | 1.000 | 1797 | tags=61%, list=31%, signal=89% |
| 203 | HSA04540\_GAP\_JUNCTION |  | 34 | -0.52 | -1.51 | 0.023 | 0.089 | 1.000 | 536 | tags=26%, list=9%, signal=29% |
| 204 | CMV\_HCMV\_TIMECOURSE\_ALL\_DN |  | 162 | -0.44 | -1.50 | 0.001 | 0.091 | 1.000 | 838 | tags=27%, list=15%, signal=30% |
| 205 | IL1\_CORNEA\_DN |  | 32 | -0.53 | -1.50 | 0.030 | 0.091 | 1.000 | 1510 | tags=50%, list=26%, signal=68% |
| 206 | TPA\_RESIST\_LATE\_DN |  | 24 | -0.56 | -1.50 | 0.029 | 0.092 | 1.000 | 938 | tags=33%, list=16%, signal=40% |
| 207 | NING\_COPD\_DN |  | 48 | -0.49 | -1.50 | 0.025 | 0.094 | 1.000 | 958 | tags=31%, list=17%, signal=37% |
| 208 | HSA00530\_AMINOSUGARS\_METABOLISM |  | 20 | -0.57 | -1.49 | 0.031 | 0.099 | 1.000 | 812 | tags=45%, list=14%, signal=52% |
| 209 | NING\_COPD\_UP |  | 56 | -0.48 | -1.49 | 0.017 | 0.099 | 1.000 | 975 | tags=27%, list=17%, signal=32% |
| 210 | IGF\_VS\_PDGF\_DN |  | 20 | -0.57 | -1.49 | 0.046 | 0.102 | 1.000 | 794 | tags=45%, list=14%, signal=52% |
| 211 | RIBAVIRIN\_RSV\_DN |  | 17 | -0.59 | -1.49 | 0.039 | 0.102 | 1.000 | 1387 | tags=65%, list=24%, signal=85% |
| 212 | HDACI\_COLON\_CUR\_UP |  | 42 | -0.49 | -1.49 | 0.026 | 0.103 | 1.000 | 1560 | tags=40%, list=27%, signal=55% |
| 213 | MAMMARY\_DEV\_UP |  | 21 | -0.56 | -1.48 | 0.045 | 0.104 | 1.000 | 945 | tags=38%, list=17%, signal=45% |
| 214 | GH\_AUTOCRINE\_UP |  | 59 | -0.47 | -1.48 | 0.021 | 0.107 | 1.000 | 850 | tags=29%, list=15%, signal=33% |
| 215 | SMOOTH\_MUSCLE\_CONTRACTION |  | 52 | -0.48 | -1.48 | 0.026 | 0.108 | 1.000 | 565 | tags=29%, list=10%, signal=32% |
| 216 | BRCA2\_BRCA1\_DN |  | 20 | -0.56 | -1.48 | 0.034 | 0.107 | 1.000 | 291 | tags=20%, list=5%, signal=21% |
| 217 | ROME\_INSULIN\_2F\_UP |  | 84 | -0.45 | -1.48 | 0.016 | 0.108 | 1.000 | 1482 | tags=36%, list=26%, signal=47% |
| 218 | KRETZSCHMAR\_IL6\_DIFF |  | 64 | -0.47 | -1.48 | 0.012 | 0.108 | 1.000 | 1826 | tags=53%, list=32%, signal=77% |
| 219 | LVAD\_HEARTFAILURE\_UP |  | 28 | -0.53 | -1.48 | 0.046 | 0.108 | 1.000 | 663 | tags=29%, list=12%, signal=32% |
| 220 | BROCKE\_IL6 |  | 64 | -0.47 | -1.47 | 0.019 | 0.111 | 1.000 | 1826 | tags=53%, list=32%, signal=77% |
| 221 | IRITANI\_ADPROX\_LYMPH |  | 56 | -0.47 | -1.47 | 0.026 | 0.115 | 1.000 | 629 | tags=27%, list=11%, signal=30% |
| 222 | HSA04350\_TGF\_BETA\_SIGNALING\_PATHWAY |  | 31 | -0.51 | -1.47 | 0.049 | 0.115 | 1.000 | 1303 | tags=39%, list=23%, signal=50% |
| 223 | HSC\_MATURE\_ADULT |  | 147 | -0.43 | -1.47 | 0.004 | 0.115 | 1.000 | 1437 | tags=36%, list=25%, signal=47% |
| 224 | CALCINEURIN\_NF\_AT\_SIGNALING |  | 36 | -0.50 | -1.46 | 0.039 | 0.123 | 1.000 | 694 | tags=22%, list=12%, signal=25% |
| 225 | TPA\_SENS\_LATE\_DN |  | 95 | -0.43 | -1.46 | 0.023 | 0.126 | 1.000 | 1444 | tags=40%, list=25%, signal=53% |
| 226 | SHEPARD\_NEG\_REG\_OF\_CELL\_PROLIFERATION |  | 40 | -0.49 | -1.45 | 0.038 | 0.127 | 1.000 | 1714 | tags=48%, list=30%, signal=67% |
| 227 | LEE\_MYC\_UP |  | 23 | -0.54 | -1.45 | 0.044 | 0.127 | 1.000 | 476 | tags=22%, list=8%, signal=24% |
| 228 | TPA\_SENS\_MIDDLE\_UP |  | 28 | -0.52 | -1.45 | 0.046 | 0.130 | 1.000 | 1388 | tags=43%, list=24%, signal=56% |
| 229 | LEE\_CIP\_DN |  | 16 | -0.58 | -1.45 | 0.051 | 0.131 | 1.000 | 590 | tags=31%, list=10%, signal=35% |
| 230 | GAMMA\_UNIQUE\_FIBRO\_DN |  | 19 | -0.55 | -1.45 | 0.065 | 0.132 | 1.000 | 1838 | tags=58%, list=32%, signal=85% |
| 231 | ADDYA\_K562\_HEMIN\_TREATMENT |  | 27 | -0.52 | -1.45 | 0.054 | 0.133 | 1.000 | 1064 | tags=41%, list=19%, signal=50% |
| 232 | AGED\_MOUSE\_CEREBELLUM\_UP |  | 27 | -0.53 | -1.45 | 0.056 | 0.133 | 1.000 | 1243 | tags=44%, list=22%, signal=57% |
| 233 | BRG1\_ALAB\_DN |  | 19 | -0.56 | -1.44 | 0.060 | 0.134 | 1.000 | 220 | tags=21%, list=4%, signal=22% |
| 234 | NELSON\_ANDROGEN\_UP |  | 27 | -0.52 | -1.44 | 0.045 | 0.136 | 1.000 | 1181 | tags=48%, list=21%, signal=60% |
| 235 | PASSERINI\_PROLIFERATION |  | 29 | -0.51 | -1.44 | 0.037 | 0.142 | 1.000 | 603 | tags=28%, list=11%, signal=31% |
| 236 | FATTY\_ACID\_METABOLISM |  | 26 | -0.51 | -1.43 | 0.066 | 0.144 | 1.000 | 1203 | tags=31%, list=21%, signal=39% |
| 237 | ROSS\_CBF |  | 21 | -0.54 | -1.43 | 0.061 | 0.145 | 1.000 | 1146 | tags=48%, list=20%, signal=59% |
| 238 | ADIPOGENESIS\_HMSC\_CLASS3\_UP |  | 30 | -0.51 | -1.43 | 0.055 | 0.145 | 1.000 | 1167 | tags=37%, list=20%, signal=46% |
| 239 | FSH\_OVARY\_MCV152\_UP |  | 30 | -0.51 | -1.43 | 0.062 | 0.145 | 1.000 | 856 | tags=37%, list=15%, signal=43% |
| 240 | HSC\_MATURE\_SHARED |  | 112 | -0.42 | -1.43 | 0.019 | 0.147 | 1.000 | 1437 | tags=38%, list=25%, signal=49% |
| 241 | CMV\_8HRS\_DN |  | 20 | -0.55 | -1.42 | 0.078 | 0.159 | 1.000 | 884 | tags=35%, list=15%, signal=41% |
| 242 | NEMETH\_TNF\_UP |  | 32 | -0.49 | -1.42 | 0.053 | 0.160 | 1.000 | 1083 | tags=44%, list=19%, signal=54% |
| 243 | LINDSTEDT\_DEND\_8H\_VS\_48H\_UP |  | 29 | -0.50 | -1.42 | 0.044 | 0.159 | 1.000 | 888 | tags=38%, list=16%, signal=45% |
| 244 | FERRANDO\_MLL\_T\_ALL\_UP |  | 37 | -0.48 | -1.41 | 0.061 | 0.163 | 1.000 | 1218 | tags=43%, list=21%, signal=55% |
| 245 | POD1\_KO\_UP |  | 133 | -0.41 | -1.41 | 0.017 | 0.167 | 1.000 | 765 | tags=25%, list=13%, signal=28% |
| 246 | CMV\_HCMV\_6HRS\_DN |  | 16 | -0.57 | -1.41 | 0.079 | 0.167 | 1.000 | 458 | tags=38%, list=8%, signal=41% |
| 247 | ALZHEIMERS\_DISEASE\_DN |  | 482 | -0.39 | -1.41 | 0.000 | 0.172 | 1.000 | 2256 | tags=52%, list=39%, signal=78% |
| 248 | H2O2\_CSBRESCUED\_UP |  | 19 | -0.55 | -1.40 | 0.072 | 0.174 | 1.000 | 678 | tags=37%, list=12%, signal=42% |
| 249 | CIS\_XPC\_DN |  | 77 | -0.43 | -1.40 | 0.034 | 0.174 | 1.000 | 1506 | tags=36%, list=26%, signal=49% |
| 250 | ET743\_RESIST\_DN |  | 17 | -0.56 | -1.40 | 0.068 | 0.177 | 1.000 | 551 | tags=35%, list=10%, signal=39% |
| 251 | CMV\_HCMV\_TIMECOURSE\_20HRS\_DN |  | 18 | -0.55 | -1.40 | 0.073 | 0.176 | 1.000 | 804 | tags=39%, list=14%, signal=45% |
| 252 | BRCA\_ER\_NEG |  | 400 | -0.39 | -1.40 | 0.000 | 0.179 | 1.000 | 1120 | tags=28%, list=20%, signal=32% |
| 253 | SMITH\_HTERT\_UP |  | 56 | -0.45 | -1.40 | 0.035 | 0.179 | 1.000 | 1335 | tags=32%, list=23%, signal=42% |
| 254 | HSC\_HSC\_FETAL |  | 81 | -0.43 | -1.39 | 0.031 | 0.181 | 1.000 | 658 | tags=19%, list=11%, signal=21% |
| 255 | BRCA1\_MES\_UP |  | 24 | -0.52 | -1.39 | 0.081 | 0.181 | 1.000 | 593 | tags=25%, list=10%, signal=28% |
| 256 | WERNER\_FIBRO\_UP |  | 22 | -0.53 | -1.39 | 0.079 | 0.181 | 1.000 | 1330 | tags=36%, list=23%, signal=47% |
| 257 | IDX\_TSA\_UP\_CLUSTER6 |  | 76 | -0.43 | -1.39 | 0.045 | 0.186 | 1.000 | 2242 | tags=55%, list=39%, signal=90% |
| 258 | HSC\_HSC\_SHARED |  | 75 | -0.43 | -1.38 | 0.038 | 0.192 | 1.000 | 658 | tags=19%, list=11%, signal=21% |
| 259 | SHEPARD\_CELL\_PROLIFERATION |  | 70 | -0.43 | -1.38 | 0.056 | 0.193 | 1.000 | 505 | tags=20%, list=9%, signal=22% |
| 260 | MATSUDA\_VALPHAINKT\_DIFF |  | 174 | -0.41 | -1.38 | 0.005 | 0.194 | 1.000 | 1194 | tags=30%, list=21%, signal=37% |
| 261 | CELL\_PROLIFERATION |  | 70 | -0.43 | -1.38 | 0.037 | 0.195 | 1.000 | 505 | tags=20%, list=9%, signal=22% |
| 262 | YAO\_P4\_KO\_VS\_WT\_UP |  | 21 | -0.52 | -1.38 | 0.094 | 0.195 | 1.000 | 1030 | tags=43%, list=18%, signal=52% |
| 263 | HPV31\_DN |  | 17 | -0.56 | -1.38 | 0.093 | 0.195 | 1.000 | 147 | tags=18%, list=3%, signal=18% |
| 264 | HDACI\_COLON\_TSA2HRS\_UP |  | 22 | -0.52 | -1.38 | 0.090 | 0.195 | 1.000 | 1153 | tags=45%, list=20%, signal=57% |
| 265 | ESR\_FIBROBLAST\_UP |  | 19 | -0.54 | -1.38 | 0.083 | 0.195 | 1.000 | 958 | tags=37%, list=17%, signal=44% |
| 266 | BRENTANI\_IMMUNE\_FUNCTION |  | 26 | -0.50 | -1.38 | 0.086 | 0.196 | 1.000 | 1243 | tags=58%, list=22%, signal=73% |
| 267 | HSC\_HSCANDPROGENITORS\_ADULT |  | 204 | -0.40 | -1.38 | 0.007 | 0.196 | 1.000 | 1129 | tags=30%, list=20%, signal=37% |
| 268 | GALINDO\_ACT\_UP |  | 27 | -0.49 | -1.38 | 0.078 | 0.196 | 1.000 | 1001 | tags=37%, list=17%, signal=45% |
| 269 | UVB\_NHEK3\_C8 |  | 34 | -0.48 | -1.38 | 0.076 | 0.198 | 1.000 | 1162 | tags=41%, list=20%, signal=51% |
| 270 | OXSTRESS\_RPETWO\_DN |  | 50 | -0.45 | -1.37 | 0.062 | 0.198 | 1.000 | 1239 | tags=38%, list=22%, signal=48% |
| 271 | WALKER\_MM\_SNP\_DIFF |  | 17 | -0.55 | -1.37 | 0.096 | 0.198 | 1.000 | 766 | tags=35%, list=13%, signal=41% |
| 272 | ZHAN\_MMPC\_LATEVS |  | 23 | -0.52 | -1.37 | 0.086 | 0.199 | 1.000 | 1874 | tags=70%, list=33%, signal=103% |
| 273 | GH\_EXOGENOUS\_LATE\_DN |  | 26 | -0.51 | -1.37 | 0.081 | 0.198 | 1.000 | 1239 | tags=42%, list=22%, signal=54% |
| 274 | TAKEDA\_NUP8\_HOXA9\_16D\_UP |  | 47 | -0.45 | -1.37 | 0.057 | 0.202 | 1.000 | 884 | tags=36%, list=15%, signal=42% |
| 275 | HSC\_MATURE\_FETAL |  | 138 | -0.40 | -1.37 | 0.017 | 0.201 | 1.000 | 1516 | tags=37%, list=26%, signal=49% |
| 276 | BHATTACHARYA\_ESC\_UP |  | 17 | -0.55 | -1.37 | 0.109 | 0.205 | 1.000 | 419 | tags=18%, list=7%, signal=19% |
| 277 | LEE\_E2F1\_DN |  | 21 | -0.51 | -1.36 | 0.102 | 0.207 | 1.000 | 693 | tags=33%, list=12%, signal=38% |
| 278 | SERUM\_FIBROBLAST\_CORE\_DN |  | 79 | -0.42 | -1.36 | 0.039 | 0.207 | 1.000 | 991 | tags=28%, list=17%, signal=33% |
| 279 | HYPOXIA\_NORMAL\_UP |  | 85 | -0.42 | -1.36 | 0.040 | 0.211 | 1.000 | 1196 | tags=32%, list=21%, signal=40% |
| 280 | TPA\_RESIST\_EARLY\_DN |  | 30 | -0.49 | -1.36 | 0.098 | 0.211 | 1.000 | 987 | tags=27%, list=17%, signal=32% |
| 281 | RAY\_P210\_DIFF |  | 18 | -0.53 | -1.36 | 0.091 | 0.213 | 1.000 | 639 | tags=33%, list=11%, signal=37% |
| 282 | STEMCELL\_COMMON\_UP |  | 103 | -0.41 | -1.36 | 0.052 | 0.212 | 1.000 | 1510 | tags=38%, list=26%, signal=51% |
| 283 | BRENTANI\_SIGNALING |  | 76 | -0.42 | -1.36 | 0.048 | 0.212 | 1.000 | 1163 | tags=33%, list=20%, signal=41% |
| 284 | HSA04630\_JAK\_STAT\_SIGNALING\_PATHWAY |  | 52 | -0.44 | -1.36 | 0.074 | 0.215 | 1.000 | 990 | tags=27%, list=17%, signal=32% |
| 285 | KNUDSEN\_PMNS\_UP |  | 35 | -0.47 | -1.36 | 0.078 | 0.214 | 1.000 | 1152 | tags=43%, list=20%, signal=53% |
| 286 | GN\_CAMP\_GRANULOSA\_DN |  | 26 | -0.49 | -1.35 | 0.097 | 0.215 | 1.000 | 830 | tags=35%, list=15%, signal=40% |
| 287 | BRCA1KO\_MEF\_DN |  | 38 | -0.45 | -1.35 | 0.096 | 0.225 | 1.000 | 2536 | tags=66%, list=44%, signal=117% |
| 288 | HSA05130\_PATHOGENIC\_ESCHERICHIA\_COLI\_INFECTION\_EHEC |  | 16 | -0.54 | -1.35 | 0.102 | 0.225 | 1.000 | 1163 | tags=44%, list=20%, signal=55% |
| 289 | BRG1\_SW13\_UP |  | 16 | -0.54 | -1.35 | 0.106 | 0.227 | 1.000 | 1902 | tags=63%, list=33%, signal=93% |
| 290 | HSC\_HSCANDPROGENITORS\_SHARED |  | 200 | -0.39 | -1.34 | 0.012 | 0.231 | 1.000 | 1239 | tags=32%, list=22%, signal=39% |
| 291 | ST\_T\_CELL\_SIGNAL\_TRANSDUCTION |  | 25 | -0.50 | -1.34 | 0.105 | 0.231 | 1.000 | 1083 | tags=32%, list=19%, signal=39% |
| 292 | CHANG\_SERUM\_RESPONSE\_DN |  | 54 | -0.43 | -1.34 | 0.088 | 0.231 | 1.000 | 918 | tags=31%, list=16%, signal=37% |
| 293 | BAF57\_BT549\_DN |  | 116 | -0.40 | -1.34 | 0.040 | 0.231 | 1.000 | 974 | tags=22%, list=17%, signal=26% |
| 294 | HOFFMANN\_BIVSBII\_LGBII |  | 43 | -0.45 | -1.34 | 0.089 | 0.230 | 1.000 | 1233 | tags=40%, list=22%, signal=50% |
| 295 | HSC\_HSCANDPROGENITORS\_FETAL |  | 200 | -0.39 | -1.34 | 0.013 | 0.230 | 1.000 | 1239 | tags=32%, list=22%, signal=39% |
| 296 | TPA\_SENS\_EARLY\_DN |  | 110 | -0.40 | -1.34 | 0.044 | 0.229 | 1.000 | 898 | tags=25%, list=16%, signal=29% |
| 297 | ASTON\_DEPRESSION\_DN |  | 48 | -0.44 | -1.34 | 0.082 | 0.228 | 1.000 | 1104 | tags=38%, list=19%, signal=46% |
| 298 | LEE\_ACOX1\_DN |  | 17 | -0.54 | -1.34 | 0.120 | 0.228 | 1.000 | 590 | tags=35%, list=10%, signal=39% |
| 299 | HSA05131\_PATHOGENIC\_ESCHERICHIA\_COLI\_INFECTION\_EPEC |  | 16 | -0.54 | -1.33 | 0.141 | 0.235 | 1.000 | 1163 | tags=44%, list=20%, signal=55% |
| 300 | PYRUVATE\_METABOLISM |  | 15 | -0.53 | -1.33 | 0.102 | 0.237 | 1.000 | 693 | tags=27%, list=12%, signal=30% |
| 301 | MOREAUX\_TACI\_HI\_VS\_LOW\_UP |  | 81 | -0.41 | -1.33 | 0.070 | 0.236 | 1.000 | 644 | tags=19%, list=11%, signal=21% |
| 302 | HDACI\_COLON\_SUL16HRS\_DN |  | 22 | -0.51 | -1.33 | 0.115 | 0.237 | 1.000 | 568 | tags=23%, list=10%, signal=25% |
| 303 | VENTRICLES\_UP |  | 79 | -0.41 | -1.33 | 0.062 | 0.239 | 1.000 | 1118 | tags=25%, list=20%, signal=31% |
| 304 | ELECTRON\_TRANSPORT |  | 21 | -0.51 | -1.33 | 0.120 | 0.241 | 1.000 | 797 | tags=33%, list=14%, signal=39% |
| 305 | GUO\_HEX\_UP |  | 35 | -0.45 | -1.33 | 0.119 | 0.244 | 1.000 | 853 | tags=31%, list=15%, signal=37% |
| 306 | VANTVEER\_BREAST\_OUTCOME\_GOOD\_VS\_POOR\_DN |  | 33 | -0.46 | -1.33 | 0.116 | 0.245 | 1.000 | 579 | tags=21%, list=10%, signal=23% |
| 307 | LEE\_MYC\_TGFA\_DN |  | 20 | -0.51 | -1.32 | 0.130 | 0.247 | 1.000 | 767 | tags=35%, list=13%, signal=40% |
| 308 | HDACI\_COLON\_CUR48HRS\_UP |  | 26 | -0.48 | -1.32 | 0.119 | 0.248 | 1.000 | 1029 | tags=35%, list=18%, signal=42% |
| 309 | HDACI\_COLON\_BUT16HRS\_UP |  | 16 | -0.54 | -1.32 | 0.132 | 0.250 | 1.000 | 1992 | tags=69%, list=35%, signal=105% |
| 310 | HSA00190\_OXIDATIVE\_PHOSPHORYLATION |  | 55 | -0.42 | -1.32 | 0.088 | 0.251 | 1.000 | 2492 | tags=64%, list=44%, signal=112% |
| 311 | UV-4NQO\_FIBRO\_UP |  | 16 | -0.52 | -1.32 | 0.154 | 0.253 | 1.000 | 1336 | tags=56%, list=23%, signal=73% |
| 312 | LINDSTEDT\_DEND\_UP |  | 23 | -0.49 | -1.32 | 0.126 | 0.255 | 1.000 | 962 | tags=35%, list=17%, signal=42% |
| 313 | VANASSE\_BCL2\_TARGETS |  | 37 | -0.45 | -1.31 | 0.105 | 0.256 | 1.000 | 1204 | tags=41%, list=21%, signal=51% |
| 314 | UVC\_TTD\_8HR\_DN |  | 75 | -0.40 | -1.31 | 0.084 | 0.258 | 1.000 | 1481 | tags=43%, list=26%, signal=57% |
| 315 | HDACI\_COLON\_CURSUL\_UP |  | 18 | -0.51 | -1.31 | 0.142 | 0.261 | 1.000 | 1026 | tags=33%, list=18%, signal=40% |
| 316 | HDACI\_COLON\_BUT2HRS\_UP |  | 24 | -0.48 | -1.31 | 0.144 | 0.269 | 1.000 | 1099 | tags=38%, list=19%, signal=46% |
| 317 | HDACI\_COLON\_BUT\_UP |  | 62 | -0.41 | -1.31 | 0.112 | 0.270 | 1.000 | 1267 | tags=34%, list=22%, signal=43% |
| 318 | ALZHEIMERS\_INCIPIENT\_UP |  | 134 | -0.39 | -1.30 | 0.050 | 0.274 | 1.000 | 1717 | tags=40%, list=30%, signal=55% |
| 319 | ELECTRON\_TRANSPORT\_CHAIN |  | 46 | -0.43 | -1.30 | 0.105 | 0.273 | 1.000 | 2356 | tags=59%, list=41%, signal=99% |
| 320 | HSC\_HSC\_ADULT |  | 96 | -0.40 | -1.30 | 0.073 | 0.275 | 1.000 | 661 | tags=19%, list=12%, signal=21% |
| 321 | OLD\_FIBRO\_UP |  | 26 | -0.47 | -1.30 | 0.148 | 0.275 | 1.000 | 1294 | tags=31%, list=23%, signal=40% |
| 322 | AGED\_RHESUS\_DN |  | 58 | -0.41 | -1.30 | 0.105 | 0.278 | 1.000 | 1162 | tags=31%, list=20%, signal=39% |
| 323 | OXIDATIVE\_PHOSPHORYLATION |  | 26 | -0.48 | -1.30 | 0.143 | 0.279 | 1.000 | 2469 | tags=65%, list=43%, signal=114% |
| 324 | UVB\_NHEK3\_ALL |  | 197 | -0.38 | -1.29 | 0.031 | 0.286 | 1.000 | 838 | tags=21%, list=15%, signal=24% |
| 325 | IDX\_TSA\_DN\_CLUSTER5 |  | 25 | -0.46 | -1.29 | 0.116 | 0.288 | 1.000 | 2109 | tags=56%, list=37%, signal=88% |
| 326 | E2F3\_ONCOGENIC\_SIGNATURE |  | 77 | -0.40 | -1.29 | 0.077 | 0.289 | 1.000 | 964 | tags=27%, list=17%, signal=32% |
| 327 | HCC\_SURVIVAL\_GOOD\_VS\_POOR\_DN |  | 56 | -0.42 | -1.29 | 0.114 | 0.291 | 1.000 | 817 | tags=18%, list=14%, signal=21% |
| 328 | HSA00640\_PROPANOATE\_METABOLISM |  | 19 | -0.49 | -1.29 | 0.165 | 0.291 | 1.000 | 1805 | tags=53%, list=32%, signal=77% |
| 329 | INTEGRIN\_MEDIATED\_CELL\_ADHESION\_KEGG |  | 46 | -0.42 | -1.28 | 0.122 | 0.296 | 1.000 | 1605 | tags=41%, list=28%, signal=57% |
| 330 | UEDA\_MOUSE\_LIVER |  | 47 | -0.42 | -1.28 | 0.134 | 0.305 | 1.000 | 1348 | tags=36%, list=24%, signal=47% |
| 331 | MYOD\_NIH3T3\_UP |  | 29 | -0.45 | -1.28 | 0.153 | 0.308 | 1.000 | 960 | tags=28%, list=17%, signal=33% |
| 332 | KLEIN\_PEL\_UP |  | 20 | -0.48 | -1.27 | 0.152 | 0.311 | 1.000 | 1766 | tags=55%, list=31%, signal=79% |
| 333 | SANA\_IFNG\_ENDOTHELIAL\_UP |  | 19 | -0.49 | -1.27 | 0.179 | 0.311 | 1.000 | 545 | tags=21%, list=10%, signal=23% |
| 334 | HSA04916\_MELANOGENESIS |  | 28 | -0.46 | -1.27 | 0.162 | 0.311 | 1.000 | 678 | tags=32%, list=12%, signal=36% |
| 335 | BRCA1\_OVEREXP\_PROSTATE\_DN |  | 35 | -0.44 | -1.27 | 0.174 | 0.319 | 1.000 | 956 | tags=31%, list=17%, signal=37% |
| 336 | ET743PT650\_COLONCA\_DN |  | 21 | -0.48 | -1.27 | 0.185 | 0.324 | 1.000 | 1919 | tags=52%, list=34%, signal=79% |
| 337 | GPCRS\_CLASS\_A\_RHODOPSIN\_LIKE |  | 18 | -0.50 | -1.26 | 0.169 | 0.324 | 1.000 | 548 | tags=33%, list=10%, signal=37% |
| 338 | MOOTHA\_VOXPHOS |  | 35 | -0.43 | -1.26 | 0.152 | 0.324 | 1.000 | 2356 | tags=57%, list=41%, signal=97% |
| 339 | TPA\_SENS\_MIDDLE\_DN |  | 116 | -0.37 | -1.26 | 0.073 | 0.323 | 1.000 | 1305 | tags=31%, list=23%, signal=39% |
| 340 | IFN\_BETA\_GLIOMA\_DN |  | 21 | -0.49 | -1.26 | 0.190 | 0.323 | 1.000 | 1309 | tags=38%, list=23%, signal=49% |
| 341 | PENG\_LEUCINE\_UP |  | 46 | -0.42 | -1.26 | 0.140 | 0.323 | 1.000 | 597 | tags=17%, list=10%, signal=19% |
| 342 | BYSTRYKH\_HSC\_TRANS\_GLOCUS |  | 314 | -0.36 | -1.26 | 0.017 | 0.323 | 1.000 | 1103 | tags=25%, list=19%, signal=29% |
| 343 | FLOTHO\_CASP8AP2\_MRD\_DIFF |  | 26 | -0.46 | -1.26 | 0.166 | 0.322 | 1.000 | 1120 | tags=38%, list=20%, signal=48% |
| 344 | GH\_GHRHR\_KO\_24HRS\_UP |  | 56 | -0.41 | -1.26 | 0.148 | 0.326 | 1.000 | 866 | tags=29%, list=15%, signal=33% |
| 345 | BREASTCA\_TWO\_CLASSES |  | 64 | -0.40 | -1.26 | 0.131 | 0.328 | 1.000 | 1481 | tags=30%, list=26%, signal=40% |
| 346 | HDACI\_COLON\_SUL\_DN |  | 81 | -0.38 | -1.25 | 0.102 | 0.338 | 1.000 | 1571 | tags=36%, list=27%, signal=49% |
| 347 | HSA04810\_REGULATION\_OF\_ACTIN\_CYTOSKELETON |  | 85 | -0.39 | -1.25 | 0.115 | 0.338 | 1.000 | 1163 | tags=26%, list=20%, signal=32% |
| 348 | HOFFMANN\_BIVSBII\_BI |  | 44 | -0.42 | -1.25 | 0.145 | 0.337 | 1.000 | 1335 | tags=34%, list=23%, signal=44% |
| 349 | PARK\_RARALPHA\_MOD |  | 26 | -0.46 | -1.25 | 0.185 | 0.343 | 1.000 | 884 | tags=27%, list=15%, signal=32% |
| 350 | FCER1PATHWAY |  | 25 | -0.46 | -1.25 | 0.167 | 0.345 | 1.000 | 582 | tags=16%, list=10%, signal=18% |
| 351 | AGED\_MOUSE\_NEOCORTEX\_DN |  | 17 | -0.49 | -1.25 | 0.201 | 0.346 | 1.000 | 480 | tags=24%, list=8%, signal=26% |
| 352 | HSA00510\_N\_GLYCAN\_BIOSYNTHESIS |  | 27 | -0.45 | -1.24 | 0.203 | 0.355 | 1.000 | 2334 | tags=59%, list=41%, signal=100% |
| 353 | HSA04730\_LONG\_TERM\_DEPRESSION |  | 30 | -0.44 | -1.24 | 0.171 | 0.355 | 1.000 | 1746 | tags=50%, list=31%, signal=72% |
| 354 | HDACI\_COLON\_BUT30MIN\_DN |  | 24 | -0.46 | -1.24 | 0.178 | 0.354 | 1.000 | 1426 | tags=38%, list=25%, signal=50% |
| 355 | BYSTRYKH\_HSC\_CIS\_GLOCUS |  | 48 | -0.40 | -1.24 | 0.172 | 0.354 | 1.000 | 1352 | tags=40%, list=24%, signal=51% |
| 356 | HDACI\_COLON\_BUT24HRS\_UP |  | 31 | -0.44 | -1.24 | 0.195 | 0.353 | 1.000 | 1215 | tags=35%, list=21%, signal=45% |
| 357 | BRCA\_ER\_POS |  | 162 | -0.36 | -1.24 | 0.082 | 0.356 | 1.000 | 1403 | tags=31%, list=25%, signal=41% |
| 358 | CMV\_HCMV\_TIMECOURSE\_48HRS\_DN |  | 42 | -0.42 | -1.24 | 0.176 | 0.356 | 1.000 | 1075 | tags=33%, list=19%, signal=41% |
| 359 | MUNSHI\_MM\_UP |  | 26 | -0.45 | -1.24 | 0.185 | 0.356 | 1.000 | 1977 | tags=46%, list=35%, signal=70% |
| 360 | AGED\_MOUSE\_CEREBELLUM\_DN |  | 15 | -0.51 | -1.24 | 0.209 | 0.355 | 1.000 | 622 | tags=33%, list=11%, signal=37% |
| 361 | GLYCOGEN\_METABOLISM |  | 18 | -0.48 | -1.23 | 0.215 | 0.364 | 1.000 | 2215 | tags=56%, list=39%, signal=90% |
| 362 | PGC |  | 156 | -0.36 | -1.23 | 0.099 | 0.365 | 1.000 | 2076 | tags=43%, list=36%, signal=66% |
| 363 | ELECTRON\_TRANSPORTER\_ACTIVITY |  | 35 | -0.42 | -1.23 | 0.200 | 0.365 | 1.000 | 1172 | tags=26%, list=20%, signal=32% |
| 364 | ALZHEIMERS\_INCIPIENT\_DN |  | 71 | -0.38 | -1.23 | 0.164 | 0.365 | 1.000 | 2534 | tags=61%, list=44%, signal=107% |
| 365 | AGED\_RHESUS\_UP |  | 68 | -0.38 | -1.23 | 0.150 | 0.368 | 1.000 | 887 | tags=25%, list=15%, signal=29% |
| 366 | HSA00620\_PYRUVATE\_METABOLISM |  | 17 | -0.48 | -1.23 | 0.207 | 0.370 | 1.000 | 693 | tags=24%, list=12%, signal=27% |
| 367 | CALRES\_MOUSE\_NEOCORTEX\_UP |  | 23 | -0.46 | -1.23 | 0.205 | 0.369 | 1.000 | 1111 | tags=30%, list=19%, signal=38% |
| 368 | IDX\_TSA\_DN\_CLUSTER3 |  | 31 | -0.43 | -1.22 | 0.207 | 0.371 | 1.000 | 1692 | tags=61%, list=30%, signal=87% |
| 369 | NGUYEN\_KERATO\_DN |  | 34 | -0.43 | -1.22 | 0.198 | 0.371 | 1.000 | 585 | tags=21%, list=10%, signal=23% |
| 370 | OXSTRESS\_RPE\_H2O2TBH\_DN |  | 16 | -0.49 | -1.22 | 0.218 | 0.375 | 1.000 | 639 | tags=31%, list=11%, signal=35% |
| 371 | MUNSHI\_MM\_VS\_PCS\_UP |  | 29 | -0.43 | -1.22 | 0.228 | 0.374 | 1.000 | 1233 | tags=34%, list=22%, signal=44% |
| 372 | ST\_INTEGRIN\_SIGNALING\_PATHWAY |  | 42 | -0.40 | -1.22 | 0.206 | 0.377 | 1.000 | 1605 | tags=38%, list=28%, signal=53% |
| 373 | DORSAM\_HOXA9\_DN |  | 15 | -0.50 | -1.22 | 0.246 | 0.383 | 1.000 | 1461 | tags=40%, list=26%, signal=54% |
| 374 | AS3\_FIBRO\_C2 |  | 16 | -0.49 | -1.21 | 0.248 | 0.388 | 1.000 | 639 | tags=31%, list=11%, signal=35% |
| 375 | KIM\_TH\_CELLS\_UP |  | 27 | -0.43 | -1.21 | 0.230 | 0.387 | 1.000 | 2102 | tags=56%, list=37%, signal=87% |
| 376 | IFN\_BETA\_GLIOMA\_UP |  | 25 | -0.44 | -1.21 | 0.245 | 0.387 | 1.000 | 1100 | tags=36%, list=19%, signal=44% |
| 377 | ZHAN\_TONSIL\_BONEMARROW |  | 21 | -0.46 | -1.21 | 0.224 | 0.386 | 1.000 | 1324 | tags=48%, list=23%, signal=62% |
| 378 | PPARAPATHWAY |  | 27 | -0.44 | -1.21 | 0.208 | 0.387 | 1.000 | 479 | tags=11%, list=8%, signal=12% |
| 379 | GOLDRATH\_HP |  | 80 | -0.37 | -1.21 | 0.186 | 0.387 | 1.000 | 1975 | tags=48%, list=35%, signal=72% |
| 380 | STEMCELL\_COMMON\_DN |  | 25 | -0.43 | -1.21 | 0.230 | 0.393 | 1.000 | 236 | tags=20%, list=4%, signal=21% |
| 381 | OLDONLY\_FIBRO\_UP |  | 17 | -0.48 | -1.21 | 0.225 | 0.394 | 1.000 | 1184 | tags=29%, list=21%, signal=37% |
| 382 | AGED\_MOUSE\_HIPPOCAMPUS\_ANY\_DN |  | 17 | -0.48 | -1.21 | 0.244 | 0.393 | 1.000 | 817 | tags=35%, list=14%, signal=41% |
| 383 | AS3\_FIBRO\_C1 |  | 16 | -0.49 | -1.21 | 0.224 | 0.393 | 1.000 | 639 | tags=31%, list=11%, signal=35% |
| 384 | ZHAN\_MM\_CD1\_VS\_CD2\_DN |  | 21 | -0.46 | -1.21 | 0.255 | 0.394 | 1.000 | 606 | tags=33%, list=11%, signal=37% |
| 385 | AGEING\_BRAIN\_DN |  | 51 | -0.39 | -1.20 | 0.220 | 0.394 | 1.000 | 1527 | tags=37%, list=27%, signal=50% |
| 386 | CMV\_HCMV\_TIMECOURSE\_16HRS\_UP |  | 34 | -0.42 | -1.20 | 0.209 | 0.395 | 1.000 | 384 | tags=18%, list=7%, signal=19% |
| 387 | PROPANOATE\_METABOLISM |  | 19 | -0.46 | -1.20 | 0.240 | 0.398 | 1.000 | 1805 | tags=47%, list=32%, signal=69% |
| 388 | HDACI\_COLON\_SUL30MIN\_DN |  | 18 | -0.46 | -1.20 | 0.226 | 0.401 | 1.000 | 2033 | tags=50%, list=36%, signal=77% |
| 389 | FALT\_BCLL\_DN |  | 22 | -0.45 | -1.20 | 0.255 | 0.401 | 1.000 | 1454 | tags=41%, list=25%, signal=55% |
| 390 | IRS1\_KO\_ADIP\_UP |  | 36 | -0.41 | -1.20 | 0.219 | 0.402 | 1.000 | 671 | tags=22%, list=12%, signal=25% |
| 391 | UVB\_NHEK3\_C0 |  | 42 | -0.40 | -1.20 | 0.205 | 0.404 | 1.000 | 536 | tags=17%, list=9%, signal=18% |
| 392 | HSA04020\_CALCIUM\_SIGNALING\_PATHWAY |  | 42 | -0.40 | -1.20 | 0.224 | 0.403 | 1.000 | 548 | tags=21%, list=10%, signal=24% |
| 393 | ADIP\_DIFF\_CLUSTER3 |  | 18 | -0.47 | -1.20 | 0.251 | 0.406 | 1.000 | 2453 | tags=72%, list=43%, signal=126% |
| 394 | ZHAN\_MULTIPLE\_MYELOMA\_VS\_NORMAL\_UP |  | 26 | -0.43 | -1.19 | 0.240 | 0.409 | 1.000 | 1317 | tags=38%, list=23%, signal=50% |
| 395 | CORDERO\_KRAS\_KD\_VS\_CONTROL\_DN |  | 16 | -0.48 | -1.19 | 0.252 | 0.408 | 1.000 | 179 | tags=13%, list=3%, signal=13% |
| 396 | DRUG\_RESISTANCE\_AND\_METABOLISM |  | 38 | -0.40 | -1.19 | 0.231 | 0.407 | 1.000 | 990 | tags=26%, list=17%, signal=32% |
| 397 | ROSS\_CBF\_LEUKEMIA |  | 18 | -0.47 | -1.19 | 0.240 | 0.409 | 1.000 | 1146 | tags=44%, list=20%, signal=55% |
| 398 | HDACI\_COLON\_SUL\_UP |  | 54 | -0.38 | -1.19 | 0.206 | 0.409 | 1.000 | 1074 | tags=24%, list=19%, signal=29% |
| 399 | CHAUHAN\_2ME2 |  | 18 | -0.47 | -1.19 | 0.250 | 0.409 | 1.000 | 1084 | tags=33%, list=19%, signal=41% |
| 400 | MENSE\_HYPOXIA\_UP |  | 36 | -0.41 | -1.18 | 0.232 | 0.422 | 1.000 | 1838 | tags=44%, list=32%, signal=65% |
| 401 | FALT\_BCLL\_IG\_MUTATED\_VS\_WT\_UP |  | 20 | -0.45 | -1.18 | 0.260 | 0.423 | 1.000 | 499 | tags=25%, list=9%, signal=27% |
| 402 | OLD\_FIBRO\_DN |  | 70 | -0.37 | -1.18 | 0.197 | 0.422 | 1.000 | 1887 | tags=44%, list=33%, signal=65% |
| 403 | ELONGINA\_KO\_DN |  | 65 | -0.37 | -1.18 | 0.205 | 0.422 | 1.000 | 595 | tags=18%, list=10%, signal=20% |
| 404 | MMS\_MOUSE\_LYMPH\_HIGH\_4HRS\_UP |  | 21 | -0.45 | -1.18 | 0.261 | 0.426 | 1.000 | 2310 | tags=62%, list=40%, signal=103% |
| 405 | HDACI\_COLON\_SUL48HRS\_UP |  | 43 | -0.39 | -1.18 | 0.248 | 0.427 | 1.000 | 1074 | tags=26%, list=19%, signal=31% |
| 406 | HDACI\_COLON\_BUT48HRS\_UP |  | 32 | -0.41 | -1.17 | 0.247 | 0.443 | 1.000 | 1239 | tags=31%, list=22%, signal=40% |
| 407 | CMV-UV\_HCMV\_6HRS\_UP |  | 38 | -0.39 | -1.17 | 0.261 | 0.442 | 1.000 | 1033 | tags=26%, list=18%, signal=32% |
| 408 | HSA05221\_ACUTE\_MYELOID\_LEUKEMIA |  | 33 | -0.40 | -1.17 | 0.254 | 0.442 | 1.000 | 357 | tags=15%, list=6%, signal=16% |
| 409 | UV-CMV\_UNIQUE\_HCMV\_6HRS\_UP |  | 32 | -0.41 | -1.17 | 0.247 | 0.445 | 1.000 | 1033 | tags=28%, list=18%, signal=34% |
| 410 | HDACI\_COLON\_SUL48HRS\_DN |  | 28 | -0.41 | -1.17 | 0.282 | 0.447 | 1.000 | 1805 | tags=46%, list=32%, signal=67% |
| 411 | HESS\_HOXAANMEIS1\_UP |  | 32 | -0.41 | -1.17 | 0.289 | 0.447 | 1.000 | 645 | tags=19%, list=11%, signal=21% |
| 412 | HESS\_HOXAANMEIS1\_DN |  | 32 | -0.41 | -1.16 | 0.272 | 0.450 | 1.000 | 645 | tags=19%, list=11%, signal=21% |
| 413 | UVC\_TTD-XPCS\_COMMON\_DN |  | 71 | -0.36 | -1.16 | 0.234 | 0.451 | 1.000 | 838 | tags=20%, list=15%, signal=23% |
| 414 | HDACI\_COLON\_TSA\_DN |  | 32 | -0.41 | -1.16 | 0.275 | 0.455 | 1.000 | 1401 | tags=38%, list=24%, signal=49% |
| 415 | HSA04664\_FC\_EPSILON\_RI\_SIGNALING\_PATHWAY |  | 41 | -0.39 | -1.16 | 0.273 | 0.457 | 1.000 | 682 | tags=20%, list=12%, signal=22% |
| 416 | LEE\_TCELLS2\_UP |  | 422 | -0.32 | -1.16 | 0.085 | 0.460 | 1.000 | 1326 | tags=27%, list=23%, signal=32% |
| 417 | OKUMURA\_MC\_LPS |  | 64 | -0.37 | -1.16 | 0.229 | 0.462 | 1.000 | 622 | tags=17%, list=11%, signal=19% |
| 418 | GH\_GHRHR\_KO\_24HRS\_DN |  | 82 | -0.35 | -1.15 | 0.246 | 0.467 | 1.000 | 1448 | tags=30%, list=25%, signal=40% |
| 419 | LH\_GRANULOSA\_DN |  | 38 | -0.39 | -1.15 | 0.276 | 0.467 | 1.000 | 957 | tags=29%, list=17%, signal=35% |
| 420 | PURINE\_METABOLISM |  | 50 | -0.38 | -1.15 | 0.275 | 0.469 | 1.000 | 876 | tags=22%, list=15%, signal=26% |
| 421 | ZHAN\_MM\_CD138\_HP\_VS\_REST |  | 15 | -0.47 | -1.15 | 0.313 | 0.473 | 1.000 | 1019 | tags=27%, list=18%, signal=32% |
| 422 | GAY\_YY1\_DN |  | 97 | -0.35 | -1.15 | 0.255 | 0.478 | 1.000 | 675 | tags=19%, list=12%, signal=21% |
| 423 | AGEING\_KIDNEY\_SPECIFIC\_DN |  | 51 | -0.37 | -1.14 | 0.276 | 0.483 | 1.000 | 1452 | tags=35%, list=25%, signal=47% |
| 424 | N\_GLYCAN\_BIOSYNTHESIS |  | 17 | -0.46 | -1.14 | 0.308 | 0.489 | 1.000 | 1992 | tags=47%, list=35%, signal=72% |
| 425 | SANA\_TNFA\_ENDOTHELIAL\_UP |  | 26 | -0.41 | -1.14 | 0.297 | 0.488 | 1.000 | 1103 | tags=35%, list=19%, signal=43% |
| 426 | AGED\_MOUSE\_HYPOTH\_DN |  | 21 | -0.44 | -1.14 | 0.320 | 0.487 | 1.000 | 918 | tags=29%, list=16%, signal=34% |
| 427 | SHEPARD\_BMYB\_MORPHOLINO\_DN |  | 61 | -0.36 | -1.14 | 0.267 | 0.490 | 1.000 | 856 | tags=23%, list=15%, signal=27% |
| 428 | HDACI\_COLON\_CUR\_DN |  | 18 | -0.44 | -1.14 | 0.319 | 0.494 | 1.000 | 324 | tags=17%, list=6%, signal=18% |
| 429 | SHEPARD\_BMYB\_MORPHOLINO\_UP |  | 61 | -0.36 | -1.13 | 0.284 | 0.494 | 1.000 | 2130 | tags=48%, list=37%, signal=75% |
| 430 | FERNANDEZ\_MYC\_TARGETS |  | 64 | -0.36 | -1.13 | 0.291 | 0.497 | 1.000 | 1792 | tags=42%, list=31%, signal=61% |
| 431 | HSA05222\_SMALL\_CELL\_LUNG\_CANCER |  | 42 | -0.38 | -1.13 | 0.295 | 0.497 | 1.000 | 1163 | tags=26%, list=20%, signal=33% |
| 432 | HSA04370\_VEGF\_SIGNALING\_PATHWAY |  | 28 | -0.41 | -1.13 | 0.313 | 0.497 | 1.000 | 770 | tags=18%, list=13%, signal=21% |
| 433 | HSA04010\_MAPK\_SIGNALING\_PATHWAY |  | 90 | -0.34 | -1.13 | 0.269 | 0.503 | 1.000 | 1097 | tags=23%, list=19%, signal=28% |
| 434 | HSA04612\_ANTIGEN\_PROCESSING\_AND\_PRESENTATION |  | 21 | -0.42 | -1.13 | 0.341 | 0.503 | 1.000 | 1443 | tags=48%, list=25%, signal=63% |
| 435 | ZHAN\_MM\_MOLECULAR\_CLASSI\_DN |  | 16 | -0.46 | -1.13 | 0.334 | 0.502 | 1.000 | 595 | tags=25%, list=10%, signal=28% |
| 436 | OLDWERNER\_FIBRO\_DN |  | 49 | -0.36 | -1.13 | 0.299 | 0.503 | 1.000 | 1872 | tags=43%, list=33%, signal=63% |
| 437 | GPCRDB\_CLASS\_A\_RHODOPSIN\_LIKE |  | 24 | -0.41 | -1.13 | 0.318 | 0.502 | 1.000 | 1137 | tags=42%, list=20%, signal=52% |
| 438 | FSH\_GRANULOSA\_DN |  | 38 | -0.39 | -1.13 | 0.312 | 0.504 | 1.000 | 957 | tags=29%, list=17%, signal=35% |
| 439 | REOVIRUS\_HEK293\_UP |  | 116 | -0.33 | -1.13 | 0.253 | 0.503 | 1.000 | 1804 | tags=39%, list=32%, signal=55% |
| 440 | UVB\_NHEK3\_C7 |  | 25 | -0.40 | -1.13 | 0.325 | 0.502 | 1.000 | 1449 | tags=40%, list=25%, signal=53% |
| 441 | REFRACTORY\_GASTRIC\_UP |  | 21 | -0.43 | -1.12 | 0.318 | 0.505 | 1.000 | 542 | tags=19%, list=9%, signal=21% |
| 442 | GAMMA-UV\_FIBRO\_UP |  | 17 | -0.44 | -1.12 | 0.333 | 0.511 | 1.000 | 1747 | tags=47%, list=31%, signal=68% |
| 443 | TENEDINI\_MEGAKARYOCYTIC\_GENES |  | 21 | -0.42 | -1.12 | 0.328 | 0.512 | 1.000 | 534 | tags=19%, list=9%, signal=21% |
| 444 | SMITH\_HTERT\_DN |  | 26 | -0.40 | -1.12 | 0.338 | 0.517 | 1.000 | 1585 | tags=42%, list=28%, signal=58% |
| 445 | HSA00860\_PORPHYRIN\_AND\_CHLOROPHYLL\_METABOLISM |  | 17 | -0.44 | -1.12 | 0.342 | 0.517 | 1.000 | 1172 | tags=29%, list=20%, signal=37% |
| 446 | FSH\_GRANULOSA\_UP |  | 28 | -0.40 | -1.11 | 0.334 | 0.523 | 1.000 | 1980 | tags=43%, list=35%, signal=65% |
| 447 | ROSS\_MLL\_FUSION |  | 33 | -0.39 | -1.11 | 0.327 | 0.526 | 1.000 | 2227 | tags=52%, list=39%, signal=84% |
| 448 | HSA05213\_ENDOMETRIAL\_CANCER |  | 30 | -0.38 | -1.10 | 0.353 | 0.539 | 1.000 | 336 | tags=13%, list=6%, signal=14% |
| 449 | GH\_AUTOCRINE\_DN |  | 43 | -0.37 | -1.10 | 0.319 | 0.538 | 1.000 | 983 | tags=23%, list=17%, signal=28% |
| 450 | HDACI\_COLON\_TSABUT\_UP |  | 22 | -0.42 | -1.10 | 0.345 | 0.546 | 1.000 | 1099 | tags=32%, list=19%, signal=39% |
| 451 | VHL\_NORMAL\_UP |  | 189 | -0.32 | -1.10 | 0.276 | 0.545 | 1.000 | 1687 | tags=35%, list=29%, signal=48% |
| 452 | IDX\_TSA\_UP\_CLUSTER5 |  | 44 | -0.37 | -1.10 | 0.347 | 0.545 | 1.000 | 1857 | tags=45%, list=32%, signal=67% |
| 453 | GH\_EXOGENOUS\_ANY\_UP |  | 52 | -0.36 | -1.10 | 0.356 | 0.547 | 1.000 | 820 | tags=25%, list=14%, signal=29% |
| 454 | LH\_GRANULOSA\_UP |  | 29 | -0.39 | -1.10 | 0.339 | 0.549 | 1.000 | 1980 | tags=41%, list=35%, signal=63% |
| 455 | DORSAM\_HOXA9\_UP |  | 18 | -0.43 | -1.10 | 0.379 | 0.549 | 1.000 | 1535 | tags=50%, list=27%, signal=68% |
| 456 | CIS\_XPC\_UP |  | 53 | -0.36 | -1.09 | 0.344 | 0.551 | 1.000 | 622 | tags=21%, list=11%, signal=23% |
| 457 | AT1RPATHWAY |  | 17 | -0.44 | -1.09 | 0.358 | 0.554 | 1.000 | 1547 | tags=35%, list=27%, signal=48% |
| 458 | OLDONLY\_FIBRO\_DN |  | 21 | -0.41 | -1.09 | 0.354 | 0.557 | 1.000 | 1573 | tags=43%, list=27%, signal=59% |
| 459 | BIOPEPTIDESPATHWAY |  | 18 | -0.43 | -1.09 | 0.381 | 0.557 | 1.000 | 1547 | tags=33%, list=27%, signal=46% |
| 460 | YAGI\_AML\_PROGNOSIS |  | 18 | -0.42 | -1.09 | 0.371 | 0.558 | 1.000 | 2557 | tags=72%, list=45%, signal=130% |
| 461 | CALCIUM\_REGULATION\_IN\_CARDIAC\_CELLS |  | 42 | -0.37 | -1.09 | 0.373 | 0.558 | 1.000 | 492 | tags=19%, list=9%, signal=21% |
| 462 | VEGFPATHWAY |  | 17 | -0.43 | -1.09 | 0.370 | 0.559 | 1.000 | 2870 | tags=71%, list=50%, signal=141% |
| 463 | ROSS\_FAB\_M7 |  | 30 | -0.38 | -1.09 | 0.371 | 0.559 | 1.000 | 1206 | tags=33%, list=21%, signal=42% |
| 464 | BRENTANI\_PROTEIN\_MODIFICATION |  | 78 | -0.33 | -1.09 | 0.333 | 0.560 | 1.000 | 1042 | tags=26%, list=18%, signal=31% |
| 465 | UVC\_HIGH\_D4\_DN |  | 29 | -0.39 | -1.08 | 0.359 | 0.563 | 1.000 | 1079 | tags=28%, list=19%, signal=34% |
| 466 | UVC\_TTD\_ALL\_DN |  | 176 | -0.31 | -1.08 | 0.312 | 0.565 | 1.000 | 1300 | tags=26%, list=23%, signal=32% |
| 467 | NADLER\_OBESITY\_HYPERGLYCEMIA |  | 20 | -0.42 | -1.08 | 0.375 | 0.567 | 1.000 | 1203 | tags=35%, list=21%, signal=44% |
| 468 | HSA04650\_NATURAL\_KILLER\_CELL\_MEDIATED\_CYTOTOXICITY |  | 45 | -0.36 | -1.08 | 0.367 | 0.568 | 1.000 | 1083 | tags=24%, list=19%, signal=30% |
| 469 | TRYPTOPHAN\_METABOLISM |  | 16 | -0.43 | -1.08 | 0.376 | 0.567 | 1.000 | 359 | tags=13%, list=6%, signal=13% |
| 470 | ST\_P38\_MAPK\_PATHWAY |  | 17 | -0.42 | -1.08 | 0.380 | 0.567 | 1.000 | 582 | tags=24%, list=10%, signal=26% |
| 471 | BRCA\_PROGNOSIS\_POS |  | 18 | -0.43 | -1.08 | 0.378 | 0.567 | 1.000 | 613 | tags=17%, list=11%, signal=19% |
| 472 | CROMER\_HYPOPHARYNGEAL\_MET\_VS\_NON\_UP |  | 36 | -0.37 | -1.08 | 0.378 | 0.567 | 1.000 | 1171 | tags=36%, list=20%, signal=45% |
| 473 | GSK3PATHWAY |  | 15 | -0.44 | -1.08 | 0.405 | 0.568 | 1.000 | 1303 | tags=40%, list=23%, signal=52% |
| 474 | UVC\_HIGH\_ALL\_DN |  | 141 | -0.32 | -1.08 | 0.342 | 0.568 | 1.000 | 960 | tags=21%, list=17%, signal=24% |
| 475 | KENNY\_WNT\_DN |  | 23 | -0.40 | -1.07 | 0.390 | 0.573 | 1.000 | 806 | tags=22%, list=14%, signal=25% |
| 476 | NF90\_DN |  | 15 | -0.44 | -1.07 | 0.389 | 0.573 | 1.000 | 639 | tags=27%, list=11%, signal=30% |
| 477 | ROSS\_AML1\_ETO |  | 23 | -0.39 | -1.07 | 0.386 | 0.574 | 1.000 | 990 | tags=35%, list=17%, signal=42% |
| 478 | HSA04130\_SNARE\_INTERACTIONS\_IN\_VESICULAR\_TRANSPORT |  | 15 | -0.44 | -1.07 | 0.366 | 0.573 | 1.000 | 1292 | tags=40%, list=23%, signal=52% |
| 479 | ZHAN\_MMPC\_SIM |  | 20 | -0.41 | -1.07 | 0.404 | 0.575 | 1.000 | 2512 | tags=65%, list=44%, signal=115% |
| 480 | BRCA\_BRCA1\_POS |  | 54 | -0.35 | -1.07 | 0.386 | 0.581 | 1.000 | 675 | tags=17%, list=12%, signal=19% |
| 481 | UVC\_TTD\_4HR\_DN |  | 149 | -0.31 | -1.07 | 0.366 | 0.580 | 1.000 | 1344 | tags=25%, list=23%, signal=32% |
| 482 | UVB\_NHEK2\_UP |  | 20 | -0.41 | -1.07 | 0.404 | 0.580 | 1.000 | 1770 | tags=40%, list=31%, signal=58% |
| 483 | GH\_GHRHR\_KO\_6HRS\_UP |  | 31 | -0.38 | -1.07 | 0.392 | 0.582 | 1.000 | 985 | tags=26%, list=17%, signal=31% |
| 484 | HSA00380\_TRYPTOPHAN\_METABOLISM |  | 29 | -0.38 | -1.06 | 0.405 | 0.595 | 1.000 | 1511 | tags=31%, list=26%, signal=42% |
| 485 | TPA\_SENS\_EARLY\_UP |  | 23 | -0.40 | -1.06 | 0.400 | 0.596 | 1.000 | 218 | tags=13%, list=4%, signal=14% |
| 486 | H2O2\_CSBDIFF\_C1 |  | 15 | -0.43 | -1.05 | 0.397 | 0.606 | 1.000 | 1440 | tags=47%, list=25%, signal=62% |
| 487 | KUROKAWA\_5FU\_IFN\_SENSITIVE\_VS\_RESISTANT\_DN |  | 16 | -0.42 | -1.05 | 0.422 | 0.606 | 1.000 | 1162 | tags=38%, list=20%, signal=47% |
| 488 | HSC\_LATEPROGENITORS\_ADULT |  | 193 | -0.30 | -1.05 | 0.366 | 0.610 | 1.000 | 2050 | tags=41%, list=36%, signal=62% |
| 489 | HOFMANN\_MANTEL\_LYMPHOMA\_VS\_LYMPH\_NODES\_UP |  | 29 | -0.37 | -1.05 | 0.423 | 0.613 | 1.000 | 1547 | tags=48%, list=27%, signal=66% |
| 490 | HIPPOCAMPUS\_DEVELOPMENT\_POSTNATAL |  | 19 | -0.41 | -1.05 | 0.426 | 0.613 | 1.000 | 718 | tags=21%, list=13%, signal=24% |
| 491 | UVC\_XPCS\_4HR\_DN |  | 120 | -0.31 | -1.05 | 0.409 | 0.616 | 1.000 | 1095 | tags=23%, list=19%, signal=27% |
| 492 | GAMMA-UV\_FIBRO\_DN |  | 20 | -0.39 | -1.04 | 0.422 | 0.619 | 1.000 | 1498 | tags=35%, list=26%, signal=47% |
| 493 | SHEPARD\_CRASH\_AND\_BURN\_MUT\_VS\_WT\_UP |  | 51 | -0.34 | -1.04 | 0.398 | 0.620 | 1.000 | 1585 | tags=25%, list=28%, signal=35% |
| 494 | LEE\_DENA\_DN |  | 20 | -0.40 | -1.04 | 0.422 | 0.622 | 1.000 | 590 | tags=25%, list=10%, signal=28% |
| 495 | TPA\_RESIST\_MIDDLE\_DN |  | 47 | -0.34 | -1.04 | 0.420 | 0.626 | 1.000 | 935 | tags=19%, list=16%, signal=23% |
| 496 | P38MAPKPATHWAY |  | 22 | -0.39 | -1.04 | 0.425 | 0.627 | 1.000 | 1016 | tags=23%, list=18%, signal=28% |
| 497 | PASSERINI\_TRANSCRIPTION |  | 32 | -0.36 | -1.04 | 0.436 | 0.631 | 1.000 | 1325 | tags=28%, list=23%, signal=36% |
| 498 | CALRES\_RHESUS\_DN |  | 27 | -0.38 | -1.03 | 0.428 | 0.630 | 1.000 | 1522 | tags=41%, list=27%, signal=55% |
| 499 | HSA00230\_PURINE\_METABOLISM |  | 73 | -0.32 | -1.03 | 0.425 | 0.630 | 1.000 | 876 | tags=19%, list=15%, signal=22% |
| 500 | HSA04920\_ADIPOCYTOKINE\_SIGNALING\_PATHWAY |  | 34 | -0.36 | -1.03 | 0.439 | 0.637 | 1.000 | 614 | tags=15%, list=11%, signal=16% |
| 501 | BRCA\_PROGNOSIS\_NEG |  | 52 | -0.33 | -1.03 | 0.455 | 0.637 | 1.000 | 1712 | tags=35%, list=30%, signal=49% |
| 502 | CELL\_GROWTH\_AND\_OR\_MAINTENANCE |  | 23 | -0.38 | -1.03 | 0.450 | 0.638 | 1.000 | 1470 | tags=30%, list=26%, signal=41% |
| 503 | LEE\_MYC\_E2F1\_DN |  | 21 | -0.39 | -1.03 | 0.441 | 0.639 | 1.000 | 767 | tags=29%, list=13%, signal=33% |
| 504 | 4NQO\_ESR\_WS\_UNREG |  | 17 | -0.41 | -1.03 | 0.459 | 0.638 | 1.000 | 1038 | tags=29%, list=18%, signal=36% |
| 505 | AGED\_MOUSE\_CORTEX\_DN |  | 24 | -0.38 | -1.03 | 0.444 | 0.639 | 1.000 | 1527 | tags=42%, list=27%, signal=57% |
| 506 | UVB\_NHEK3\_C2 |  | 25 | -0.38 | -1.03 | 0.460 | 0.639 | 1.000 | 2953 | tags=84%, list=52%, signal=173% |
| 507 | GOLDRATH\_CELLCYCLE |  | 17 | -0.40 | -1.02 | 0.459 | 0.645 | 1.000 | 964 | tags=24%, list=17%, signal=28% |
| 508 | WERNER\_FIBRO\_DN |  | 72 | -0.32 | -1.02 | 0.467 | 0.645 | 1.000 | 1872 | tags=39%, list=33%, signal=57% |
| 509 | HDACI\_COLON\_CUR24HRS\_UP |  | 20 | -0.38 | -1.02 | 0.453 | 0.644 | 1.000 | 2334 | tags=50%, list=41%, signal=84% |
| 510 | CHIARETTI\_ZAP70\_DIFF |  | 31 | -0.36 | -1.02 | 0.448 | 0.646 | 1.000 | 528 | tags=16%, list=9%, signal=18% |
| 511 | YAGI\_AML\_PROG\_ASSOC |  | 59 | -0.32 | -1.02 | 0.471 | 0.652 | 1.000 | 513 | tags=14%, list=9%, signal=15% |
| 512 | AGED\_MOUSE\_HIPPOCAMPUS\_ANY\_UP |  | 22 | -0.38 | -1.01 | 0.475 | 0.661 | 1.000 | 1315 | tags=32%, list=23%, signal=41% |
| 513 | SANA\_IFNG\_ENDOTHELIAL\_DN |  | 27 | -0.36 | -1.01 | 0.464 | 0.666 | 1.000 | 1338 | tags=33%, list=23%, signal=43% |
| 514 | CMV\_HCMV\_TIMECOURSE\_48HRS\_UP |  | 30 | -0.36 | -1.01 | 0.456 | 0.668 | 1.000 | 888 | tags=23%, list=16%, signal=27% |
| 515 | UVB\_NHEK1\_UP |  | 52 | -0.33 | -1.01 | 0.493 | 0.669 | 1.000 | 934 | tags=17%, list=16%, signal=20% |
| 516 | HDACI\_COLON\_SUL24HRS\_DN |  | 50 | -0.33 | -1.00 | 0.486 | 0.673 | 1.000 | 1731 | tags=38%, list=30%, signal=54% |
| 517 | DSRNA\_UP |  | 16 | -0.40 | -1.00 | 0.479 | 0.672 | 1.000 | 2582 | tags=75%, list=45%, signal=136% |
| 518 | HSC\_LATEPROGENITORS\_FETAL |  | 191 | -0.29 | -1.00 | 0.484 | 0.672 | 1.000 | 2050 | tags=41%, list=36%, signal=62% |
| 519 | HSC\_LATEPROGENITORS\_SHARED |  | 189 | -0.29 | -1.00 | 0.484 | 0.672 | 1.000 | 2050 | tags=41%, list=36%, signal=62% |
| 520 | FASPATHWAY |  | 20 | -0.39 | -1.00 | 0.485 | 0.673 | 1.000 | 1547 | tags=35%, list=27%, signal=48% |
| 521 | GH\_EXOGENOUS\_ALL\_UP |  | 16 | -0.40 | -1.00 | 0.493 | 0.680 | 1.000 | 1403 | tags=50%, list=25%, signal=66% |
| 522 | HDACI\_COLON\_BUT48HRS\_DN |  | 46 | -0.33 | -1.00 | 0.492 | 0.680 | 1.000 | 2465 | tags=59%, list=43%, signal=102% |
| 523 | AS3\_FIBRO\_UP |  | 24 | -0.37 | -1.00 | 0.505 | 0.682 | 1.000 | 1585 | tags=38%, list=28%, signal=52% |
| 524 | INTEGRINPATHWAY |  | 24 | -0.37 | -1.00 | 0.486 | 0.682 | 1.000 | 1605 | tags=38%, list=28%, signal=52% |
| 525 | HSA01031\_GLYCAN\_STRUCTURES\_BIOSYNTHESIS\_2 |  | 22 | -0.37 | -0.99 | 0.487 | 0.681 | 1.000 | 2098 | tags=45%, list=37%, signal=71% |
| 526 | RCC\_NL\_UP |  | 254 | -0.28 | -0.99 | 0.519 | 0.686 | 1.000 | 1568 | tags=30%, list=27%, signal=39% |
| 527 | LU\_IL4BCELL |  | 27 | -0.35 | -0.99 | 0.502 | 0.690 | 1.000 | 1303 | tags=30%, list=23%, signal=38% |
| 528 | CMV\_HCMV\_TIMECOURSE\_6HRS\_DN |  | 26 | -0.36 | -0.99 | 0.524 | 0.694 | 1.000 | 315 | tags=12%, list=6%, signal=12% |
| 529 | PYK2PATHWAY |  | 19 | -0.39 | -0.99 | 0.511 | 0.695 | 1.000 | 1605 | tags=32%, list=28%, signal=44% |
| 530 | PRMT5\_KD\_UP |  | 95 | -0.30 | -0.99 | 0.523 | 0.694 | 1.000 | 586 | tags=13%, list=10%, signal=14% |
| 531 | HSA01510\_NEURODEGENERATIVE\_DISEASES |  | 16 | -0.39 | -0.99 | 0.504 | 0.694 | 1.000 | 1405 | tags=31%, list=25%, signal=41% |
| 532 | ZHAN\_MM\_CD1\_VS\_CD2\_UP |  | 27 | -0.35 | -0.98 | 0.508 | 0.697 | 1.000 | 797 | tags=26%, list=14%, signal=30% |
| 533 | OXSTRESS\_RPE\_H2O2HNE\_DN |  | 17 | -0.39 | -0.98 | 0.535 | 0.699 | 1.000 | 1771 | tags=53%, list=31%, signal=76% |
| 534 | HADDAD\_HSC\_CD10\_UP |  | 104 | -0.29 | -0.98 | 0.534 | 0.699 | 1.000 | 579 | tags=18%, list=10%, signal=20% |
| 535 | TSA\_HEPATOMA\_UP |  | 15 | -0.40 | -0.98 | 0.525 | 0.700 | 1.000 | 962 | tags=27%, list=17%, signal=32% |
| 536 | CALRES\_MOUSE\_DN |  | 18 | -0.39 | -0.98 | 0.535 | 0.701 | 1.000 | 1388 | tags=33%, list=24%, signal=44% |
| 537 | AS3\_FIBRO\_C3 |  | 24 | -0.37 | -0.98 | 0.530 | 0.700 | 1.000 | 1585 | tags=38%, list=28%, signal=52% |
| 538 | GLYCOLYSIS |  | 15 | -0.39 | -0.97 | 0.503 | 0.706 | 1.000 | 1162 | tags=33%, list=20%, signal=42% |
| 539 | HSA00564\_GLYCEROPHOSPHOLIPID\_METABOLISM |  | 25 | -0.36 | -0.97 | 0.514 | 0.705 | 1.000 | 437 | tags=16%, list=8%, signal=17% |
| 540 | ST\_GA13\_PATHWAY |  | 16 | -0.39 | -0.97 | 0.543 | 0.706 | 1.000 | 2702 | tags=75%, list=47%, signal=142% |
| 541 | GH\_EXOGENOUS\_MIDDLE\_UP |  | 15 | -0.39 | -0.97 | 0.521 | 0.710 | 1.000 | 603 | tags=20%, list=11%, signal=22% |
| 542 | HSA05120\_EPITHELIAL\_CELL\_SIGNALING\_IN\_HELICOBACTER\_PYLORI\_INFECTION |  | 35 | -0.33 | -0.97 | 0.519 | 0.709 | 1.000 | 1927 | tags=49%, list=34%, signal=73% |
| 543 | STEFFEN\_AML\_PML\_PLZF\_TRGT |  | 16 | -0.39 | -0.97 | 0.524 | 0.708 | 1.000 | 1224 | tags=31%, list=21%, signal=40% |
| 544 | TCRPATHWAY |  | 27 | -0.35 | -0.97 | 0.521 | 0.712 | 1.000 | 694 | tags=19%, list=12%, signal=21% |
| 545 | FLECHNER\_KIDNEY\_TRANSPLANT\_REJECTION\_PBL\_UP |  | 37 | -0.33 | -0.97 | 0.549 | 0.713 | 1.000 | 1644 | tags=38%, list=29%, signal=53% |
| 546 | HSA05215\_PROSTATE\_CANCER |  | 42 | -0.32 | -0.97 | 0.552 | 0.714 | 1.000 | 1324 | tags=26%, list=23%, signal=34% |
| 547 | LEE\_TCELLS8\_UP |  | 78 | -0.30 | -0.96 | 0.562 | 0.716 | 1.000 | 1740 | tags=37%, list=30%, signal=53% |
| 548 | HSA05110\_CHOLERA\_INFECTION |  | 21 | -0.36 | -0.96 | 0.525 | 0.717 | 1.000 | 2058 | tags=62%, list=36%, signal=96% |
| 549 | HSA00252\_ALANINE\_AND\_ASPARTATE\_METABOLISM |  | 16 | -0.38 | -0.96 | 0.531 | 0.716 | 1.000 | 418 | tags=13%, list=7%, signal=13% |
| 550 | LEE\_TCELLS1\_UP |  | 78 | -0.30 | -0.96 | 0.557 | 0.718 | 1.000 | 1740 | tags=37%, list=30%, signal=53% |
| 551 | GLUCONEOGENESIS |  | 15 | -0.39 | -0.96 | 0.544 | 0.723 | 1.000 | 1162 | tags=33%, list=20%, signal=42% |
| 552 | SIG\_CD40PATHWAYMAP |  | 15 | -0.39 | -0.95 | 0.553 | 0.732 | 1.000 | 1016 | tags=20%, list=18%, signal=24% |
| 553 | HSA01030\_GLYCAN\_STRUCTURES\_BIOSYNTHESIS\_1 |  | 43 | -0.32 | -0.95 | 0.576 | 0.733 | 1.000 | 1992 | tags=42%, list=35%, signal=64% |
| 554 | G\_PROTEIN\_SIGNALING |  | 40 | -0.32 | -0.95 | 0.550 | 0.732 | 1.000 | 876 | tags=25%, list=15%, signal=29% |
| 555 | CELL\_MOTILITY |  | 45 | -0.32 | -0.95 | 0.569 | 0.737 | 1.000 | 2036 | tags=40%, list=36%, signal=62% |
| 556 | HADDAD\_HPCLYMPHO\_ENRICHED |  | 113 | -0.28 | -0.95 | 0.611 | 0.737 | 1.000 | 579 | tags=17%, list=10%, signal=18% |
| 557 | UVB\_NHEK3\_C5 |  | 26 | -0.34 | -0.95 | 0.551 | 0.737 | 1.000 | 814 | tags=23%, list=14%, signal=27% |
| 558 | UVC\_LOW\_ALL\_DN |  | 22 | -0.36 | -0.95 | 0.540 | 0.738 | 1.000 | 1674 | tags=41%, list=29%, signal=58% |
| 559 | LEE\_TCELLS10\_UP |  | 78 | -0.30 | -0.94 | 0.590 | 0.745 | 1.000 | 1740 | tags=37%, list=30%, signal=53% |
| 560 | PENG\_GLUTAMINE\_UP |  | 115 | -0.28 | -0.94 | 0.622 | 0.746 | 1.000 | 1042 | tags=19%, list=18%, signal=23% |
| 561 | PARK\_RARALPHA\_UP |  | 21 | -0.36 | -0.94 | 0.577 | 0.747 | 1.000 | 794 | tags=24%, list=14%, signal=28% |
| 562 | ST\_DIFFERENTIATION\_PATHWAY\_IN\_PC12\_CELLS |  | 20 | -0.36 | -0.94 | 0.571 | 0.750 | 1.000 | 2865 | tags=70%, list=50%, signal=140% |
| 563 | FSH\_OVARY\_MCV152\_DN |  | 21 | -0.36 | -0.93 | 0.599 | 0.762 | 1.000 | 3009 | tags=71%, list=53%, signal=150% |
| 564 | CHESLER\_BRAIN\_CIS\_GENES |  | 31 | -0.32 | -0.92 | 0.595 | 0.782 | 1.000 | 1630 | tags=39%, list=28%, signal=54% |
| 565 | LEE\_TCELLS4\_UP |  | 17 | -0.36 | -0.91 | 0.587 | 0.787 | 1.000 | 591 | tags=18%, list=10%, signal=20% |
| 566 | AGED\_MOUSE\_HYPOTH\_UP |  | 23 | -0.34 | -0.91 | 0.594 | 0.788 | 1.000 | 1960 | tags=48%, list=34%, signal=72% |
| 567 | GLYCEROPHOSPHOLIPID\_METABOLISM |  | 22 | -0.34 | -0.91 | 0.592 | 0.787 | 1.000 | 323 | tags=14%, list=6%, signal=14% |
| 568 | KLEIN\_PEL\_DN |  | 35 | -0.31 | -0.91 | 0.610 | 0.792 | 1.000 | 521 | tags=17%, list=9%, signal=19% |
| 569 | BASSO\_GERMINAL\_CENTER\_CD40\_DN |  | 27 | -0.33 | -0.91 | 0.599 | 0.796 | 1.000 | 500 | tags=19%, list=9%, signal=20% |
| 570 | GN\_CAMP\_GRANULOSA\_UP |  | 18 | -0.36 | -0.90 | 0.619 | 0.798 | 1.000 | 323 | tags=11%, list=6%, signal=12% |
| 571 | SIG\_CHEMOTAXIS |  | 21 | -0.34 | -0.90 | 0.613 | 0.803 | 1.000 | 1019 | tags=19%, list=18%, signal=23% |
| 572 | AGEING\_KIDNEY\_DN |  | 49 | -0.30 | -0.90 | 0.633 | 0.805 | 1.000 | 858 | tags=18%, list=15%, signal=21% |
| 573 | RHOPATHWAY |  | 15 | -0.37 | -0.90 | 0.610 | 0.805 | 1.000 | 261 | tags=13%, list=5%, signal=14% |
| 574 | CHEN\_HOXA5\_TARGETS\_UP |  | 104 | -0.27 | -0.90 | 0.686 | 0.804 | 1.000 | 2072 | tags=38%, list=36%, signal=58% |
| 575 | STRESS\_TPA\_SPECIFIC\_UP |  | 19 | -0.35 | -0.90 | 0.609 | 0.803 | 1.000 | 2314 | tags=58%, list=40%, signal=97% |
| 576 | HSC\_INTERMEDIATEPROGENITORS\_ADULT |  | 57 | -0.29 | -0.89 | 0.665 | 0.808 | 1.000 | 1911 | tags=40%, list=33%, signal=60% |
| 577 | CMV\_ALL\_UP |  | 41 | -0.29 | -0.89 | 0.639 | 0.807 | 1.000 | 1875 | tags=39%, list=33%, signal=58% |
| 578 | CMV\_HCMV\_TIMECOURSE\_ALL\_UP |  | 209 | -0.26 | -0.89 | 0.753 | 0.806 | 1.000 | 1122 | tags=19%, list=20%, signal=23% |
| 579 | PHOSPHATIDYLINOSITOL\_SIGNALING\_SYSTEM |  | 43 | -0.29 | -0.89 | 0.641 | 0.808 | 1.000 | 501 | tags=16%, list=9%, signal=18% |
| 580 | SANSOM\_APC\_LOSS4\_UP |  | 39 | -0.30 | -0.89 | 0.660 | 0.812 | 1.000 | 994 | tags=23%, list=17%, signal=28% |
| 581 | LIZUKA\_G1\_SM\_G2 |  | 17 | -0.35 | -0.89 | 0.627 | 0.817 | 1.000 | 1638 | tags=29%, list=29%, signal=41% |
| 582 | HSA04912\_GNRH\_SIGNALING\_PATHWAY |  | 34 | -0.31 | -0.88 | 0.652 | 0.828 | 1.000 | 582 | tags=18%, list=10%, signal=20% |
| 583 | SMITH\_HCV\_INDUCED\_HCC\_UP |  | 15 | -0.36 | -0.88 | 0.647 | 0.828 | 1.000 | 1232 | tags=33%, list=22%, signal=42% |
| 584 | CHESLER\_HIGHEST\_FOLD\_RANGE\_GENES |  | 22 | -0.33 | -0.88 | 0.643 | 0.828 | 1.000 | 2084 | tags=55%, list=36%, signal=85% |
| 585 | MOREAUX\_TACI\_HI\_IN\_BMPC |  | 15 | -0.35 | -0.88 | 0.642 | 0.827 | 1.000 | 22 | tags=7%, list=0%, signal=7% |
| 586 | BRG1\_ALAB\_UP |  | 17 | -0.35 | -0.88 | 0.651 | 0.828 | 1.000 | 1439 | tags=47%, list=25%, signal=63% |
| 587 | SASAKI\_ATL\_UP |  | 71 | -0.27 | -0.87 | 0.701 | 0.832 | 1.000 | 2118 | tags=45%, list=37%, signal=71% |
| 588 | ST\_DICTYOSTELIUM\_DISCOIDEUM\_CAMP\_CHEMOTAXIS\_PATHWAY |  | 21 | -0.33 | -0.87 | 0.669 | 0.839 | 1.000 | 2017 | tags=38%, list=35%, signal=59% |
| 589 | ZHAN\_MMPC\_SIM\_BC\_AND\_MM |  | 23 | -0.32 | -0.86 | 0.691 | 0.850 | 1.000 | 777 | tags=13%, list=14%, signal=15% |
| 590 | HSA05210\_COLORECTAL\_CANCER |  | 43 | -0.28 | -0.86 | 0.700 | 0.849 | 1.000 | 700 | tags=14%, list=12%, signal=16% |
| 591 | STRESS\_ARSENIC\_SPECIFIC\_UP |  | 63 | -0.27 | -0.86 | 0.717 | 0.849 | 1.000 | 1925 | tags=32%, list=34%, signal=47% |
| 592 | SASAKI\_TCELL\_LYMPHOMA\_VS\_CD4\_UP |  | 71 | -0.27 | -0.86 | 0.731 | 0.848 | 1.000 | 2118 | tags=45%, list=37%, signal=71% |
| 593 | SERUM\_FIBROBLAST\_CORE\_UP |  | 86 | -0.26 | -0.85 | 0.746 | 0.856 | 1.000 | 1522 | tags=27%, list=27%, signal=36% |
| 594 | HDACI\_COLON\_BUT\_DN |  | 114 | -0.25 | -0.85 | 0.765 | 0.856 | 1.000 | 2085 | tags=41%, list=36%, signal=64% |
| 595 | IFN\_ANY\_UP |  | 33 | -0.29 | -0.84 | 0.697 | 0.868 | 1.000 | 2412 | tags=52%, list=42%, signal=89% |
| 596 | UVC\_XPCS\_ALL\_DN |  | 248 | -0.24 | -0.84 | 0.856 | 0.867 | 1.000 | 1104 | tags=19%, list=19%, signal=22% |
| 597 | PARK\_MSCS\_LIN2 |  | 24 | -0.32 | -0.84 | 0.705 | 0.867 | 1.000 | 1126 | tags=25%, list=20%, signal=31% |
| 598 | P53\_SIGNALING |  | 43 | -0.28 | -0.84 | 0.715 | 0.866 | 1.000 | 826 | tags=16%, list=14%, signal=19% |
| 599 | UV\_UNIQUE\_FIBRO\_DN |  | 15 | -0.34 | -0.84 | 0.690 | 0.872 | 1.000 | 2238 | tags=60%, list=39%, signal=98% |
| 600 | MYC\_TARGETS |  | 18 | -0.33 | -0.83 | 0.707 | 0.882 | 1.000 | 143 | tags=11%, list=2%, signal=11% |
| 601 | CTNNB1\_ONCOGENIC\_SIGNATURE |  | 32 | -0.29 | -0.83 | 0.719 | 0.881 | 1.000 | 1837 | tags=41%, list=32%, signal=59% |
| 602 | VERNELL\_PRB\_CLSTR1 |  | 35 | -0.28 | -0.83 | 0.727 | 0.881 | 1.000 | 1515 | tags=23%, list=26%, signal=31% |
| 603 | HDACI\_COLON\_CLUSTER9 |  | 25 | -0.30 | -0.83 | 0.712 | 0.884 | 1.000 | 1749 | tags=40%, list=31%, signal=57% |
| 604 | HEMATOPOESIS\_RELATED\_TRANSCRIPTION\_FACTORS |  | 37 | -0.28 | -0.83 | 0.731 | 0.883 | 1.000 | 718 | tags=19%, list=13%, signal=21% |
| 605 | LYSINE\_DEGRADATION |  | 17 | -0.32 | -0.82 | 0.719 | 0.890 | 1.000 | 359 | tags=12%, list=6%, signal=13% |
| 606 | HSC\_INTERMEDIATEPROGENITORS\_SHARED |  | 52 | -0.27 | -0.82 | 0.777 | 0.896 | 1.000 | 1991 | tags=40%, list=35%, signal=61% |
| 607 | CALRES\_MOUSE\_NEOCORTEX\_DN |  | 32 | -0.28 | -0.81 | 0.732 | 0.898 | 1.000 | 675 | tags=16%, list=12%, signal=18% |
| 608 | DER\_IFNG\_UP |  | 20 | -0.31 | -0.81 | 0.737 | 0.897 | 1.000 | 2017 | tags=45%, list=35%, signal=69% |
| 609 | FLECHNER\_KIDNEY\_TRANSPLANT\_WELL\_PBL\_DN |  | 21 | -0.31 | -0.81 | 0.727 | 0.897 | 1.000 | 2323 | tags=48%, list=41%, signal=80% |
| 610 | SANSOM\_APC\_LOSS5\_UP |  | 24 | -0.30 | -0.81 | 0.734 | 0.898 | 1.000 | 804 | tags=21%, list=14%, signal=24% |
| 611 | COCAINE\_BRAIN\_4WKS\_UP |  | 25 | -0.29 | -0.81 | 0.727 | 0.898 | 1.000 | 458 | tags=16%, list=8%, signal=17% |
| 612 | AGUIRRE\_PANCREAS\_CHR12 |  | 25 | -0.29 | -0.81 | 0.730 | 0.901 | 1.000 | 2101 | tags=44%, list=37%, signal=69% |
| 613 | LE\_MYELIN\_UP |  | 43 | -0.27 | -0.80 | 0.790 | 0.912 | 1.000 | 990 | tags=16%, list=17%, signal=20% |
| 614 | HSA05212\_PANCREATIC\_CANCER |  | 35 | -0.27 | -0.80 | 0.769 | 0.911 | 1.000 | 1553 | tags=26%, list=27%, signal=35% |
| 615 | HYPOXIA\_RCC\_NOVHL\_UP |  | 28 | -0.29 | -0.79 | 0.755 | 0.916 | 1.000 | 1295 | tags=25%, list=23%, signal=32% |
| 616 | BRG1\_H1299\_UP |  | 16 | -0.32 | -0.79 | 0.749 | 0.916 | 1.000 | 639 | tags=19%, list=11%, signal=21% |
| 617 | VALINE\_LEUCINE\_AND\_ISOLEUCINE\_DEGRADATION |  | 25 | -0.29 | -0.79 | 0.761 | 0.916 | 1.000 | 1925 | tags=36%, list=34%, signal=54% |
| 618 | UVC\_XPCS\_8HR\_DN |  | 215 | -0.23 | -0.79 | 0.895 | 0.915 | 1.000 | 2716 | tags=53%, list=47%, signal=98% |
| 619 | NOUZOVA\_CPG\_H4\_UP |  | 50 | -0.26 | -0.79 | 0.800 | 0.918 | 1.000 | 2171 | tags=38%, list=38%, signal=61% |
| 620 | TNFR1PATHWAY |  | 16 | -0.31 | -0.78 | 0.745 | 0.922 | 1.000 | 1547 | tags=25%, list=27%, signal=34% |
| 621 | CMV\_24HRS\_UP |  | 31 | -0.27 | -0.78 | 0.774 | 0.923 | 1.000 | 718 | tags=16%, list=13%, signal=18% |
| 622 | HSA04620\_TOLL\_LIKE\_RECEPTOR\_SIGNALING\_PATHWAY |  | 47 | -0.26 | -0.78 | 0.794 | 0.923 | 1.000 | 1891 | tags=36%, list=33%, signal=54% |
| 623 | ST\_ERK1\_ERK2\_MAPK\_PATHWAY |  | 15 | -0.32 | -0.78 | 0.772 | 0.923 | 1.000 | 2811 | tags=67%, list=49%, signal=131% |
| 624 | HUMAN\_MITODB\_6\_2002 |  | 184 | -0.23 | -0.78 | 0.897 | 0.921 | 1.000 | 2086 | tags=36%, list=36%, signal=55% |
| 625 | HSA05219\_BLADDER\_CANCER |  | 18 | -0.30 | -0.78 | 0.775 | 0.920 | 1.000 | 2079 | tags=50%, list=36%, signal=78% |
| 626 | DEATHPATHWAY |  | 17 | -0.31 | -0.78 | 0.771 | 0.921 | 1.000 | 2687 | tags=53%, list=47%, signal=99% |
| 627 | HDACI\_COLON\_BUT12HRS\_DN |  | 34 | -0.26 | -0.78 | 0.798 | 0.924 | 1.000 | 2161 | tags=38%, list=38%, signal=61% |
| 628 | METPATHWAY |  | 23 | -0.29 | -0.77 | 0.775 | 0.923 | 1.000 | 1547 | tags=30%, list=27%, signal=42% |
| 629 | HSA00280\_VALINE\_LEUCINE\_AND\_ISOLEUCINE\_DEGRADATION |  | 32 | -0.27 | -0.77 | 0.804 | 0.923 | 1.000 | 2054 | tags=38%, list=36%, signal=58% |
| 630 | FALT\_BCLL\_IG\_MUTATED\_VS\_WT\_DN |  | 22 | -0.28 | -0.76 | 0.794 | 0.937 | 1.000 | 1389 | tags=27%, list=24%, signal=36% |
| 631 | TARTE\_BCELL |  | 21 | -0.29 | -0.76 | 0.791 | 0.936 | 1.000 | 930 | tags=24%, list=16%, signal=28% |
| 632 | CROMER\_HYPOPHARYNGEAL\_MET\_VS\_NON\_DN |  | 30 | -0.27 | -0.76 | 0.795 | 0.935 | 1.000 | 1403 | tags=37%, list=25%, signal=48% |
| 633 | AGUIRRE\_PANCREAS\_CHR19 |  | 27 | -0.28 | -0.76 | 0.828 | 0.936 | 1.000 | 1149 | tags=22%, list=20%, signal=28% |
| 634 | HIVNEFPATHWAY |  | 29 | -0.27 | -0.75 | 0.822 | 0.942 | 1.000 | 1547 | tags=31%, list=27%, signal=42% |
| 635 | PDGFPATHWAY |  | 18 | -0.30 | -0.75 | 0.802 | 0.942 | 1.000 | 1547 | tags=22%, list=27%, signal=30% |
| 636 | HSC\_INTERMEDIATEPROGENITORS\_FETAL |  | 64 | -0.24 | -0.75 | 0.858 | 0.943 | 1.000 | 1911 | tags=38%, list=33%, signal=56% |
| 637 | BASSO\_GERMINAL\_CENTER\_CD40\_UP |  | 32 | -0.26 | -0.75 | 0.800 | 0.944 | 1.000 | 1393 | tags=28%, list=24%, signal=37% |
| 638 | BYSTRYKH\_HSC\_BRAIN\_TRANS\_GLOCUS |  | 75 | -0.23 | -0.75 | 0.864 | 0.944 | 1.000 | 1492 | tags=27%, list=26%, signal=36% |
| 639 | CHANG\_SERUM\_RESPONSE\_UP |  | 69 | -0.24 | -0.75 | 0.860 | 0.943 | 1.000 | 1481 | tags=25%, list=26%, signal=33% |
| 640 | PENG\_GLUCOSE\_DN |  | 61 | -0.24 | -0.75 | 0.867 | 0.942 | 1.000 | 2355 | tags=44%, list=41%, signal=74% |
| 641 | APOPTOSIS\_KEGG |  | 19 | -0.29 | -0.74 | 0.801 | 0.943 | 1.000 | 1792 | tags=32%, list=31%, signal=46% |
| 642 | VHL\_RCC\_UP |  | 56 | -0.24 | -0.74 | 0.851 | 0.943 | 1.000 | 1861 | tags=39%, list=33%, signal=58% |
| 643 | HSA05211\_RENAL\_CELL\_CARCINOMA |  | 43 | -0.25 | -0.74 | 0.843 | 0.944 | 1.000 | 2996 | tags=56%, list=52%, signal=116% |
| 644 | UVC\_HIGH\_D7\_DN |  | 17 | -0.30 | -0.74 | 0.800 | 0.942 | 1.000 | 1505 | tags=35%, list=26%, signal=48% |
| 645 | XU\_CBP\_DN |  | 17 | -0.29 | -0.74 | 0.811 | 0.942 | 1.000 | 637 | tags=18%, list=11%, signal=20% |
| 646 | HSA05214\_GLIOMA |  | 30 | -0.26 | -0.74 | 0.819 | 0.941 | 1.000 | 1489 | tags=23%, list=26%, signal=31% |
| 647 | O6BG\_RESIST\_MEDULLOBLASTOMA\_DN |  | 25 | -0.27 | -0.74 | 0.837 | 0.941 | 1.000 | 772 | tags=20%, list=13%, signal=23% |
| 648 | HYPOXIA\_RCC\_UP |  | 40 | -0.25 | -0.74 | 0.845 | 0.942 | 1.000 | 2024 | tags=38%, list=35%, signal=58% |
| 649 | HSA00500\_STARCH\_AND\_SUCROSE\_METABOLISM |  | 26 | -0.27 | -0.73 | 0.823 | 0.946 | 1.000 | 567 | tags=15%, list=10%, signal=17% |
| 650 | HDACI\_COLON\_SUL24HRS\_UP |  | 25 | -0.27 | -0.73 | 0.823 | 0.944 | 1.000 | 1026 | tags=16%, list=18%, signal=19% |
| 651 | INSULIN\_SIGNALING |  | 43 | -0.25 | -0.73 | 0.862 | 0.944 | 1.000 | 1512 | tags=21%, list=26%, signal=28% |
| 652 | HDACI\_COLON\_BUT16HRS\_DN |  | 45 | -0.24 | -0.73 | 0.857 | 0.945 | 1.000 | 1746 | tags=36%, list=31%, signal=51% |
| 653 | BASSO\_REGULATORY\_HUBS |  | 69 | -0.23 | -0.73 | 0.886 | 0.944 | 1.000 | 658 | tags=10%, list=11%, signal=11% |
| 654 | ERKPATHWAY |  | 15 | -0.29 | -0.73 | 0.825 | 0.945 | 1.000 | 1305 | tags=27%, list=23%, signal=34% |
| 655 | HDACI\_COLON\_BUT24HRS\_DN |  | 42 | -0.24 | -0.72 | 0.857 | 0.945 | 1.000 | 596 | tags=14%, list=10%, signal=16% |
| 656 | HSC\_EARLYPROGENITORS\_ADULT |  | 193 | -0.21 | -0.72 | 0.955 | 0.948 | 1.000 | 1729 | tags=31%, list=30%, signal=42% |
| 657 | ASTIER\_FN\_DIFF |  | 21 | -0.27 | -0.72 | 0.838 | 0.952 | 1.000 | 1059 | tags=24%, list=19%, signal=29% |
| 658 | MENSE\_HYPOXIA\_TRANSPORTER\_GENES |  | 20 | -0.27 | -0.72 | 0.846 | 0.951 | 1.000 | 1462 | tags=30%, list=26%, signal=40% |
| 659 | WERNERONLY\_FIBRO\_DN |  | 24 | -0.27 | -0.71 | 0.857 | 0.950 | 1.000 | 962 | tags=21%, list=17%, signal=25% |
| 660 | HDACI\_COLON\_CLUSTER10 |  | 17 | -0.28 | -0.70 | 0.840 | 0.960 | 1.000 | 1051 | tags=24%, list=18%, signal=29% |
| 661 | RADAEVA\_IFNA\_UP |  | 18 | -0.28 | -0.70 | 0.860 | 0.962 | 1.000 | 560 | tags=17%, list=10%, signal=18% |
| 662 | ASTIER\_BCELL |  | 21 | -0.27 | -0.70 | 0.864 | 0.961 | 1.000 | 1059 | tags=24%, list=19%, signal=29% |
| 663 | KENNY\_WNT\_UP |  | 23 | -0.26 | -0.70 | 0.852 | 0.961 | 1.000 | 2594 | tags=48%, list=45%, signal=87% |
| 664 | BLEO\_HUMAN\_LYMPH\_HIGH\_24HRS\_UP |  | 46 | -0.23 | -0.70 | 0.896 | 0.960 | 1.000 | 928 | tags=17%, list=16%, signal=21% |
| 665 | HSA00071\_FATTY\_ACID\_METABOLISM |  | 24 | -0.26 | -0.70 | 0.864 | 0.962 | 1.000 | 1510 | tags=25%, list=26%, signal=34% |
| 666 | CANCER\_NEOPLASTIC\_META\_UP |  | 32 | -0.24 | -0.69 | 0.881 | 0.961 | 1.000 | 524 | tags=13%, list=9%, signal=14% |
| 667 | HOFFMANN\_BIVSBII\_BI\_TABLE2 |  | 98 | -0.21 | -0.69 | 0.933 | 0.962 | 1.000 | 1303 | tags=19%, list=23%, signal=25% |
| 668 | PEART\_HISTONE\_UP |  | 28 | -0.25 | -0.69 | 0.870 | 0.964 | 1.000 | 1856 | tags=36%, list=32%, signal=53% |
| 669 | IFNA\_HCMV\_6HRS\_UP |  | 18 | -0.27 | -0.68 | 0.875 | 0.967 | 1.000 | 728 | tags=17%, list=13%, signal=19% |
| 670 | HSA04210\_APOPTOSIS |  | 38 | -0.23 | -0.68 | 0.897 | 0.970 | 1.000 | 1282 | tags=18%, list=22%, signal=24% |
| 671 | IFN\_BETA\_UP |  | 29 | -0.24 | -0.68 | 0.881 | 0.972 | 1.000 | 2412 | tags=48%, list=42%, signal=83% |
| 672 | HSC\_EARLYPROGENITORS\_SHARED |  | 192 | -0.20 | -0.68 | 0.971 | 0.970 | 1.000 | 1729 | tags=30%, list=30%, signal=42% |
| 673 | LIN\_WNT\_UP |  | 24 | -0.24 | -0.67 | 0.892 | 0.970 | 1.000 | 990 | tags=17%, list=17%, signal=20% |
| 674 | HSC\_EARLYPROGENITORS\_FETAL |  | 192 | -0.20 | -0.67 | 0.970 | 0.969 | 1.000 | 1729 | tags=30%, list=30%, signal=42% |
| 675 | FERRANDO\_MLL\_T\_ALL\_DN |  | 46 | -0.22 | -0.66 | 0.918 | 0.976 | 1.000 | 814 | tags=15%, list=14%, signal=18% |
| 676 | HSA04120\_UBIQUITIN\_MEDIATED\_PROTEOLYSIS |  | 26 | -0.24 | -0.66 | 0.909 | 0.978 | 1.000 | 2704 | tags=50%, list=47%, signal=94% |
| 677 | UVB\_NHEK1\_C6 |  | 59 | -0.21 | -0.66 | 0.945 | 0.979 | 1.000 | 770 | tags=12%, list=13%, signal=14% |
| 678 | TRNA\_SYNTHETASES |  | 15 | -0.26 | -0.65 | 0.888 | 0.983 | 1.000 | 3052 | tags=67%, list=53%, signal=142% |
| 679 | OLDAGE\_DN |  | 24 | -0.24 | -0.65 | 0.913 | 0.982 | 1.000 | 838 | tags=13%, list=15%, signal=15% |
| 680 | MOREAUX\_TACI\_HI\_IN\_PPC\_UP |  | 33 | -0.23 | -0.64 | 0.923 | 0.984 | 1.000 | 2171 | tags=36%, list=38%, signal=58% |
| 681 | PENG\_RAPAMYCIN\_DN |  | 106 | -0.19 | -0.64 | 0.967 | 0.984 | 1.000 | 1628 | tags=25%, list=28%, signal=35% |
| 682 | FLECHNER\_KIDNEY\_TRANSPLANT\_WELL\_PBL\_UP |  | 69 | -0.20 | -0.64 | 0.967 | 0.984 | 1.000 | 1919 | tags=30%, list=34%, signal=45% |
| 683 | HSC\_STHSC\_ADULT |  | 18 | -0.25 | -0.64 | 0.905 | 0.983 | 1.000 | 125 | tags=6%, list=2%, signal=6% |
| 684 | IL1RPATHWAY |  | 16 | -0.25 | -0.64 | 0.895 | 0.984 | 1.000 | 957 | tags=19%, list=17%, signal=22% |
| 685 | IDX\_TSA\_DN\_CLUSTER6 |  | 15 | -0.26 | -0.63 | 0.898 | 0.985 | 1.000 | 1274 | tags=27%, list=22%, signal=34% |
| 686 | SIG\_INSULIN\_RECEPTOR\_PATHWAY\_IN\_CARDIAC\_MYOCYTES |  | 23 | -0.23 | -0.63 | 0.914 | 0.985 | 1.000 | 132 | tags=4%, list=2%, signal=4% |
| 687 | CERAMIDEPATHWAY |  | 15 | -0.26 | -0.63 | 0.915 | 0.984 | 1.000 | 2906 | tags=60%, list=51%, signal=122% |
| 688 | HSA00260\_GLYCINE\_SERINE\_AND\_THREONINE\_METABOLISM |  | 17 | -0.25 | -0.63 | 0.905 | 0.985 | 1.000 | 1121 | tags=18%, list=20%, signal=22% |
| 689 | CMV\_HCMV\_TIMECOURSE\_24HRS\_UP |  | 31 | -0.22 | -0.62 | 0.948 | 0.990 | 1.000 | 495 | tags=13%, list=9%, signal=14% |
| 690 | ST\_JNK\_MAPK\_PATHWAY |  | 17 | -0.24 | -0.61 | 0.939 | 0.991 | 1.000 | 582 | tags=12%, list=10%, signal=13% |
| 691 | FLECHNER\_KIDNEY\_TRANSPLANT\_REJECTION\_PBL\_DN |  | 24 | -0.23 | -0.61 | 0.944 | 0.992 | 1.000 | 1730 | tags=25%, list=30%, signal=36% |
| 692 | MITOCHONDRIA |  | 183 | -0.17 | -0.60 | 0.995 | 0.996 | 1.000 | 2086 | tags=34%, list=36%, signal=52% |
| 693 | CMV\_HCMV\_TIMECOURSE\_20HRS\_UP |  | 40 | -0.20 | -0.60 | 0.951 | 0.995 | 1.000 | 1444 | tags=23%, list=25%, signal=30% |
| 694 | GPCRPATHWAY |  | 16 | -0.24 | -0.60 | 0.942 | 0.994 | 1.000 | 1305 | tags=19%, list=23%, signal=24% |
| 695 | AGUIRRE\_PANCREAS\_CHR6 |  | 15 | -0.24 | -0.59 | 0.940 | 0.994 | 1.000 | 2764 | tags=80%, list=48%, signal=154% |
| 696 | KNUDSEN\_PMNS\_DN |  | 107 | -0.18 | -0.59 | 0.986 | 0.994 | 1.000 | 1553 | tags=25%, list=27%, signal=34% |
| 697 | HUMAN\_CD34\_ENRICHED\_TRANSCRIPTION\_FACTORS |  | 73 | -0.18 | -0.59 | 0.972 | 0.993 | 1.000 | 1471 | tags=22%, list=26%, signal=29% |
| 698 | MOREAUX\_TACI\_HI\_VS\_LOW\_DN |  | 94 | -0.18 | -0.59 | 0.991 | 0.993 | 1.000 | 2412 | tags=41%, list=42%, signal=71% |
| 699 | DER\_IFNA\_UP |  | 27 | -0.21 | -0.58 | 0.957 | 0.994 | 1.000 | 2094 | tags=37%, list=37%, signal=58% |
| 700 | ET743\_SARCOMA\_72HRS\_DN |  | 116 | -0.17 | -0.57 | 0.996 | 0.998 | 1.000 | 2248 | tags=34%, list=39%, signal=54% |
| 701 | SHIPP\_FL\_VS\_DLBCL\_DN |  | 15 | -0.22 | -0.56 | 0.946 | 1.000 | 1.000 | 1232 | tags=20%, list=22%, signal=25% |
| 702 | ET743\_SARCOMA\_24HRS\_DN |  | 62 | -0.18 | -0.55 | 0.985 | 1.000 | 1.000 | 2515 | tags=42%, list=44%, signal=74% |
| 703 | TRANSLATION\_FACTORS |  | 20 | -0.21 | -0.55 | 0.965 | 1.000 | 1.000 | 2870 | tags=70%, list=50%, signal=140% |
| 704 | HSA00020\_CITRATE\_CYCLE |  | 18 | -0.22 | -0.54 | 0.972 | 1.000 | 1.000 | 1698 | tags=28%, list=30%, signal=39% |
| 705 | INOS\_ALL\_UP |  | 30 | -0.19 | -0.54 | 0.980 | 1.000 | 1.000 | 1960 | tags=30%, list=34%, signal=45% |
| 706 | TARTE\_PLASMA\_BLASTIC |  | 165 | -0.15 | -0.53 | 1.000 | 1.000 | 1.000 | 89 | tags=3%, list=2%, signal=3% |
| 707 | CMV\_HCMV\_TIMECOURSE\_14HRS\_UP |  | 21 | -0.20 | -0.53 | 0.970 | 1.000 | 1.000 | 2348 | tags=48%, list=41%, signal=80% |
| 708 | GENOTOXINS\_24HRS\_DISCR |  | 16 | -0.21 | -0.52 | 0.981 | 1.000 | 1.000 | 1434 | tags=25%, list=25%, signal=33% |
| 709 | ST\_FAS\_SIGNALING\_PATHWAY |  | 30 | -0.18 | -0.52 | 0.985 | 1.000 | 1.000 | 582 | tags=10%, list=10%, signal=11% |
| 710 | GHPATHWAY |  | 17 | -0.21 | -0.52 | 0.978 | 1.000 | 1.000 | 240 | tags=6%, list=4%, signal=6% |
| 711 | CMV\_HCMV\_TIMECOURSE\_18HRS\_UP |  | 33 | -0.18 | -0.51 | 0.990 | 1.000 | 1.000 | 2323 | tags=45%, list=41%, signal=76% |
| 712 | UVB\_NHEK3\_C1 |  | 19 | -0.20 | -0.51 | 0.978 | 1.000 | 1.000 | 241 | tags=5%, list=4%, signal=5% |
| 713 | HSA05220\_CHRONIC\_MYELOID\_LEUKEMIA |  | 41 | -0.17 | -0.50 | 0.991 | 1.000 | 1.000 | 990 | tags=12%, list=17%, signal=15% |
| 714 | GLEEVECPATHWAY |  | 17 | -0.20 | -0.50 | 0.979 | 1.000 | 1.000 | 1547 | tags=24%, list=27%, signal=32% |
| 715 | DER\_IFNB\_UP |  | 41 | -0.16 | -0.49 | 0.995 | 1.000 | 1.000 | 2412 | tags=44%, list=42%, signal=75% |
| 716 | BYSTRYKH\_HSC\_BRAIN\_CIS\_GLOCUS |  | 28 | -0.17 | -0.48 | 0.989 | 1.000 | 1.000 | 1352 | tags=25%, list=24%, signal=33% |
| 717 | RACCYCDPATHWAY |  | 15 | -0.19 | -0.48 | 0.992 | 0.999 | 1.000 | 2906 | tags=53%, list=51%, signal=108% |
| 718 | EGFPATHWAY |  | 17 | -0.19 | -0.47 | 0.991 | 0.999 | 1.000 | 1547 | tags=18%, list=27%, signal=24% |
| 719 | KREBS\_TCA\_CYCLE |  | 18 | -0.18 | -0.46 | 0.982 | 0.999 | 1.000 | 693 | tags=11%, list=12%, signal=13% |
| 720 | ET743\_SARCOMA\_DN |  | 139 | -0.13 | -0.45 | 1.000 | 0.999 | 1.000 | 2248 | tags=32%, list=39%, signal=52% |
| 721 | ET743\_SARCOMA\_48HRS\_DN |  | 106 | -0.12 | -0.41 | 1.000 | 1.000 | 1.000 | 2515 | tags=38%, list=44%, signal=66% |
| 722 | APOPTOSIS\_GENMAPP |  | 20 | -0.14 | -0.37 | 0.999 | 1.000 | 1.000 | 2692 | tags=50%, list=47%, signal=94% |
Table: Gene sets enriched in phenotype **NormalSeq (3 samples)**[plain text format]****

  
