# Supplementary material for: Comparative RNA-Seq and Microarray Analysis of Gene Expression Changes in B-Cell Lymphomas of Canis familiaris
Source: PLoS One. 2013 Apr 4;8(4):e61088. doi: 10.1371/journal.pone.0061088 (PMC3617154; doi:10.1371/journal.pone.0061088)
Supplement: Data File S2 — GSEA Results Files. (ZIP) [file pone.0061088.s005.zip › RNA-Seq/gsea_report_for_NormalSeq_v3.html]

Report for Normal 1334928352252 [GSEA]

| GS  follow link to MSigDB | GS DETAILS | SIZE | ES | NES | NOM p-val | FDR q-val | FWER p-val | RANK AT MAX | LEADING EDGE || 1 | PICCALUGA\_ANGIOIMMUNOBLASTIC\_LYMPHOMA\_UP | Details ... | 94 | -0.76 | -2.49 | 0.000 | 0.000 | 0.000 | 1102 | tags=79%, list=19%, signal=96% |
| 2 | LINDGREN\_BLADDER\_CANCER\_CLUSTER\_2B | Details ... | 168 | -0.69 | -2.35 | 0.000 | 0.000 | 0.000 | 813 | tags=50%, list=14%, signal=57% |
| 3 | SCHUETZ\_BREAST\_CANCER\_DUCTAL\_INVASIVE\_UP | Details ... | 141 | -0.67 | -2.28 | 0.000 | 0.000 | 0.000 | 954 | tags=57%, list=17%, signal=67% |
| 4 | SWEET\_LUNG\_CANCER\_KRAS\_DN | Details ... | 157 | -0.66 | -2.27 | 0.000 | 0.000 | 0.000 | 894 | tags=51%, list=16%, signal=59% |
| 5 | KEGG\_CYTOKINE\_CYTOKINE\_RECEPTOR\_INTERACTION | Details ... | 67 | -0.71 | -2.26 | 0.000 | 0.000 | 0.000 | 752 | tags=57%, list=13%, signal=65% |
| 6 | ONDER\_CDH1\_TARGETS\_2\_UP | Details ... | 101 | -0.68 | -2.26 | 0.000 | 0.000 | 0.000 | 808 | tags=53%, list=14%, signal=61% |
| 7 | SABATES\_COLORECTAL\_ADENOMA\_DN | Details ... | 64 | -0.70 | -2.26 | 0.000 | 0.000 | 0.000 | 752 | tags=53%, list=13%, signal=60% |
| 8 | LIU\_PROSTATE\_CANCER\_DN | Details ... | 142 | -0.66 | -2.25 | 0.000 | 0.000 | 0.000 | 736 | tags=48%, list=13%, signal=54% |
| 9 | VECCHI\_GASTRIC\_CANCER\_ADVANCED\_VS\_EARLY\_UP | Details ... | 55 | -0.73 | -2.25 | 0.000 | 0.000 | 0.000 | 808 | tags=62%, list=14%, signal=71% |
| 10 | LEE\_NEURAL\_CREST\_STEM\_CELL\_UP | Details ... | 46 | -0.74 | -2.24 | 0.000 | 0.000 | 0.000 | 598 | tags=65%, list=10%, signal=72% |
| 11 | SMID\_BREAST\_CANCER\_LUMINAL\_B\_DN | Details ... | 183 | -0.65 | -2.24 | 0.000 | 0.000 | 0.000 | 823 | tags=48%, list=14%, signal=54% |
| 12 | VECCHI\_GASTRIC\_CANCER\_EARLY\_DN | Details ... | 88 | -0.68 | -2.23 | 0.000 | 0.000 | 0.000 | 869 | tags=53%, list=15%, signal=62% |
| 13 | WANG\_SMARCE1\_TARGETS\_UP | Details ... | 64 | -0.71 | -2.22 | 0.000 | 0.000 | 0.000 | 671 | tags=58%, list=12%, signal=65% |
| 14 | NAKAYAMA\_SOFT\_TISSUE\_TUMORS\_PCA1\_UP | Details ... | 32 | -0.77 | -2.19 | 0.000 | 0.000 | 0.000 | 675 | tags=59%, list=12%, signal=67% |
| 15 | MOHANKUMAR\_TLX1\_TARGETS\_DN | Details ... | 36 | -0.76 | -2.19 | 0.000 | 0.000 | 0.000 | 885 | tags=67%, list=15%, signal=78% |
| 16 | SANA\_TNF\_SIGNALING\_DN | Details ... | 31 | -0.76 | -2.18 | 0.000 | 0.000 | 0.000 | 668 | tags=65%, list=12%, signal=73% |
| 17 | LIAN\_LIPA\_TARGETS\_3M | Details ... | 23 | -0.80 | -2.18 | 0.000 | 0.000 | 0.000 | 500 | tags=61%, list=9%, signal=66% |
| 18 | DELYS\_THYROID\_CANCER\_DN | Details ... | 72 | -0.68 | -2.17 | 0.000 | 0.000 | 0.000 | 945 | tags=56%, list=17%, signal=66% |
| 19 | KAAB\_FAILED\_HEART\_ATRIUM\_DN | Details ... | 78 | -0.66 | -2.15 | 0.000 | 0.000 | 0.000 | 955 | tags=51%, list=17%, signal=61% |
| 20 | TAKEDA\_TARGETS\_OF\_NUP98\_HOXA9\_FUSION\_10D\_DN | Details ... | 44 | -0.71 | -2.14 | 0.000 | 0.000 | 0.000 | 638 | tags=57%, list=11%, signal=63% |
| 21 | LIAN\_LIPA\_TARGETS\_6M |  | 25 | -0.79 | -2.13 | 0.000 | 0.000 | 0.001 | 500 | tags=56%, list=9%, signal=61% |
| 22 | JAATINEN\_HEMATOPOIETIC\_STEM\_CELL\_DN |  | 82 | -0.66 | -2.13 | 0.000 | 0.000 | 0.001 | 875 | tags=54%, list=15%, signal=62% |
| 23 | TAKEDA\_TARGETS\_OF\_NUP98\_HOXA9\_FUSION\_8D\_DN |  | 69 | -0.66 | -2.11 | 0.000 | 0.000 | 0.001 | 940 | tags=52%, list=16%, signal=62% |
| 24 | GAUSSMANN\_MLL\_AF4\_FUSION\_TARGETS\_E\_UP |  | 31 | -0.75 | -2.11 | 0.000 | 0.000 | 0.001 | 841 | tags=61%, list=15%, signal=71% |
| 25 | SMID\_BREAST\_CANCER\_NORMAL\_LIKE\_UP |  | 191 | -0.61 | -2.11 | 0.000 | 0.000 | 0.001 | 823 | tags=42%, list=14%, signal=48% |
| 26 | CHIANG\_LIVER\_CANCER\_SUBCLASS\_CTNNB1\_DN |  | 50 | -0.68 | -2.10 | 0.000 | 0.000 | 0.001 | 838 | tags=54%, list=15%, signal=63% |
| 27 | SMIRNOV\_CIRCULATING\_ENDOTHELIOCYTES\_IN\_CANCER\_UP |  | 60 | -0.67 | -2.10 | 0.000 | 0.000 | 0.001 | 951 | tags=47%, list=17%, signal=55% |
| 28 | WAMUNYOKOLI\_OVARIAN\_CANCER\_LMP\_DN |  | 88 | -0.64 | -2.10 | 0.000 | 0.000 | 0.001 | 736 | tags=43%, list=13%, signal=49% |
| 29 | HUANG\_DASATINIB\_RESISTANCE\_UP |  | 29 | -0.75 | -2.10 | 0.000 | 0.000 | 0.001 | 937 | tags=62%, list=16%, signal=74% |
| 30 | BERTUCCI\_MEDULLARY\_VS\_DUCTAL\_BREAST\_CANCER\_DN |  | 49 | -0.68 | -2.10 | 0.000 | 0.000 | 0.001 | 661 | tags=45%, list=12%, signal=50% |
| 31 | LI\_WILMS\_TUMOR\_VS\_FETAL\_KIDNEY\_1\_UP |  | 87 | -0.64 | -2.09 | 0.000 | 0.000 | 0.003 | 869 | tags=51%, list=15%, signal=59% |
| 32 | VERHAAK\_AML\_WITH\_NPM1\_MUTATED\_UP |  | 56 | -0.67 | -2.08 | 0.000 | 0.000 | 0.003 | 1102 | tags=57%, list=19%, signal=70% |
| 33 | NAKAYAMA\_SOFT\_TISSUE\_TUMORS\_PCA2\_DN |  | 17 | -0.81 | -2.06 | 0.000 | 0.000 | 0.005 | 634 | tags=65%, list=11%, signal=73% |
| 34 | BENPORATH\_PRC2\_TARGETS |  | 58 | -0.66 | -2.06 | 0.000 | 0.000 | 0.005 | 1000 | tags=53%, list=17%, signal=64% |
| 35 | MARKEY\_RB1\_CHRONIC\_LOF\_DN |  | 44 | -0.68 | -2.05 | 0.000 | 0.000 | 0.006 | 1109 | tags=61%, list=19%, signal=76% |
| 36 | LIU\_VAV3\_PROSTATE\_CARCINOGENESIS\_UP |  | 35 | -0.71 | -2.05 | 0.000 | 0.000 | 0.006 | 964 | tags=57%, list=17%, signal=68% |
| 37 | ZHU\_CMV\_24\_HR\_DN |  | 28 | -0.74 | -2.05 | 0.000 | 0.000 | 0.006 | 990 | tags=75%, list=17%, signal=90% |
| 38 | KANG\_IMMORTALIZED\_BY\_TERT\_UP |  | 26 | -0.75 | -2.04 | 0.000 | 0.000 | 0.007 | 229 | tags=31%, list=4%, signal=32% |
| 39 | TURASHVILI\_BREAST\_DUCTAL\_CARCINOMA\_VS\_DUCTAL\_NORMAL\_DN |  | 45 | -0.67 | -2.04 | 0.000 | 0.000 | 0.007 | 718 | tags=51%, list=13%, signal=58% |
| 40 | TAKEDA\_TARGETS\_OF\_NUP98\_HOXA9\_FUSION\_16D\_DN |  | 40 | -0.68 | -2.04 | 0.000 | 0.000 | 0.007 | 1003 | tags=52%, list=18%, signal=63% |
| 41 | WU\_CELL\_MIGRATION |  | 65 | -0.65 | -2.04 | 0.000 | 0.000 | 0.007 | 838 | tags=51%, list=15%, signal=59% |
| 42 | ZHU\_CMV\_ALL\_DN |  | 34 | -0.70 | -2.02 | 0.000 | 0.000 | 0.012 | 990 | tags=68%, list=17%, signal=81% |
| 43 | CHIARADONNA\_NEOPLASTIC\_TRANSFORMATION\_CDC25\_UP |  | 47 | -0.66 | -2.02 | 0.000 | 0.000 | 0.013 | 731 | tags=45%, list=13%, signal=51% |
| 44 | TAKEDA\_TARGETS\_OF\_NUP98\_HOXA9\_FUSION\_6HR\_UP |  | 30 | -0.70 | -2.01 | 0.000 | 0.000 | 0.014 | 795 | tags=53%, list=14%, signal=62% |
| 45 | REN\_ALVEOLAR\_RHABDOMYOSARCOMA\_DN |  | 217 | -0.58 | -2.01 | 0.000 | 0.000 | 0.015 | 1029 | tags=45%, list=18%, signal=53% |
| 46 | CHARAFE\_BREAST\_CANCER\_LUMINAL\_VS\_BASAL\_DN |  | 185 | -0.57 | -2.01 | 0.000 | 0.000 | 0.015 | 1557 | tags=58%, list=27%, signal=77% |
| 47 | ROZANOV\_MMP14\_TARGETS\_UP |  | 98 | -0.61 | -2.01 | 0.000 | 0.000 | 0.015 | 800 | tags=41%, list=14%, signal=47% |
| 48 | TURASHVILI\_BREAST\_LOBULAR\_CARCINOMA\_VS\_LOBULAR\_NORMAL\_UP |  | 33 | -0.70 | -2.01 | 0.000 | 0.000 | 0.015 | 1257 | tags=67%, list=22%, signal=85% |
| 49 | ALCALAY\_AML\_BY\_NPM1\_LOCALIZATION\_UP |  | 57 | -0.65 | -2.01 | 0.000 | 0.000 | 0.015 | 644 | tags=42%, list=11%, signal=47% |
| 50 | LENAOUR\_DENDRITIC\_CELL\_MATURATION\_UP |  | 40 | -0.67 | -2.01 | 0.000 | 0.000 | 0.015 | 1015 | tags=50%, list=18%, signal=60% |
| 51 | CHIBA\_RESPONSE\_TO\_TSA\_UP |  | 16 | -0.81 | -2.00 | 0.000 | 0.000 | 0.015 | 421 | tags=50%, list=7%, signal=54% |
| 52 | GAJATE\_RESPONSE\_TO\_TRABECTEDIN\_UP |  | 25 | -0.73 | -1.99 | 0.000 | 0.000 | 0.018 | 856 | tags=64%, list=15%, signal=75% |
| 53 | TURASHVILI\_BREAST\_LOBULAR\_CARCINOMA\_VS\_DUCTAL\_NORMAL\_DN |  | 28 | -0.71 | -1.99 | 0.000 | 0.000 | 0.018 | 663 | tags=50%, list=12%, signal=56% |
| 54 | KEGG\_CELL\_ADHESION\_MOLECULES\_CAMS |  | 42 | -0.66 | -1.99 | 0.000 | 0.000 | 0.018 | 1267 | tags=67%, list=22%, signal=85% |
| 55 | GRAHAM\_CML\_QUIESCENT\_VS\_NORMAL\_QUIESCENT\_DN |  | 16 | -0.79 | -1.99 | 0.000 | 0.000 | 0.018 | 802 | tags=69%, list=14%, signal=80% |
| 56 | TONKS\_TARGETS\_OF\_RUNX1\_RUNX1T1\_FUSION\_HSC\_UP |  | 81 | -0.61 | -1.98 | 0.000 | 0.000 | 0.020 | 888 | tags=47%, list=16%, signal=55% |
| 57 | KEGG\_ECM\_RECEPTOR\_INTERACTION |  | 30 | -0.70 | -1.98 | 0.000 | 0.000 | 0.021 | 1218 | tags=73%, list=21%, signal=93% |
| 58 | HINATA\_NFKB\_TARGETS\_KERATINOCYTE\_UP |  | 32 | -0.69 | -1.98 | 0.000 | 0.000 | 0.022 | 928 | tags=50%, list=16%, signal=59% |
| 59 | BOYLAN\_MULTIPLE\_MYELOMA\_C\_D\_DN |  | 98 | -0.60 | -1.98 | 0.000 | 0.000 | 0.022 | 1184 | tags=54%, list=21%, signal=67% |
| 60 | GRAHAM\_CML\_DIVIDING\_VS\_NORMAL\_QUIESCENT\_DN |  | 41 | -0.66 | -1.97 | 0.000 | 0.000 | 0.022 | 888 | tags=51%, list=16%, signal=60% |
| 61 | BENPORATH\_SUZ12\_TARGETS |  | 138 | -0.58 | -1.97 | 0.000 | 0.000 | 0.025 | 1027 | tags=47%, list=18%, signal=56% |
| 62 | BERTUCCI\_INVASIVE\_CARCINOMA\_DUCTAL\_VS\_LOBULAR\_DN |  | 19 | -0.76 | -1.96 | 0.000 | 0.000 | 0.025 | 700 | tags=68%, list=12%, signal=78% |
| 63 | SMID\_BREAST\_CANCER\_LUMINAL\_A\_UP |  | 24 | -0.72 | -1.96 | 0.000 | 0.000 | 0.027 | 945 | tags=58%, list=17%, signal=70% |
| 64 | RODWELL\_AGING\_KIDNEY\_NO\_BLOOD\_UP |  | 63 | -0.62 | -1.96 | 0.000 | 0.000 | 0.027 | 713 | tags=44%, list=12%, signal=50% |
| 65 | BASSO\_HAIRY\_CELL\_LEUKEMIA\_DN |  | 34 | -0.68 | -1.96 | 0.000 | 0.000 | 0.029 | 1239 | tags=74%, list=22%, signal=93% |
| 66 | IZADPANAH\_STEM\_CELL\_ADIPOSE\_VS\_BONE\_DN |  | 43 | -0.66 | -1.96 | 0.000 | 0.000 | 0.034 | 1349 | tags=67%, list=24%, signal=88% |
| 67 | REACTOME\_BIOLOGICAL\_OXIDATIONS |  | 23 | -0.72 | -1.95 | 0.000 | 0.001 | 0.040 | 636 | tags=52%, list=11%, signal=58% |
| 68 | WAMUNYOKOLI\_OVARIAN\_CANCER\_GRADES\_1\_2\_DN |  | 28 | -0.70 | -1.94 | 0.000 | 0.001 | 0.044 | 636 | tags=50%, list=11%, signal=56% |
| 69 | TSENG\_IRS1\_TARGETS\_DN |  | 46 | -0.64 | -1.94 | 0.000 | 0.001 | 0.047 | 964 | tags=54%, list=17%, signal=65% |
| 70 | GRAHAM\_NORMAL\_QUIESCENT\_VS\_NORMAL\_DIVIDING\_UP |  | 26 | -0.71 | -1.93 | 0.000 | 0.001 | 0.064 | 940 | tags=58%, list=16%, signal=69% |
| 71 | CHIANG\_LIVER\_CANCER\_SUBCLASS\_PROLIFERATION\_DN |  | 37 | -0.66 | -1.92 | 0.000 | 0.001 | 0.086 | 890 | tags=43%, list=16%, signal=51% |
| 72 | LEE\_LIVER\_CANCER\_E2F1\_UP |  | 26 | -0.69 | -1.91 | 0.000 | 0.001 | 0.088 | 1313 | tags=69%, list=23%, signal=89% |
| 73 | KINSEY\_TARGETS\_OF\_EWSR1\_FLII\_FUSION\_DN |  | 112 | -0.57 | -1.91 | 0.000 | 0.001 | 0.093 | 1169 | tags=50%, list=20%, signal=62% |
| 74 | GERY\_CEBP\_TARGETS |  | 48 | -0.62 | -1.91 | 0.000 | 0.001 | 0.095 | 1243 | tags=50%, list=22%, signal=63% |
| 75 | BENPORATH\_ES\_WITH\_H3K27ME3 |  | 135 | -0.56 | -1.91 | 0.000 | 0.001 | 0.095 | 864 | tags=41%, list=15%, signal=47% |
| 76 | DAVICIONI\_RHABDOMYOSARCOMA\_PAX\_FOXO1\_FUSION\_UP |  | 26 | -0.69 | -1.91 | 0.000 | 0.001 | 0.095 | 534 | tags=35%, list=9%, signal=38% |
| 77 | ROSS\_AML\_WITH\_CBFB\_MYH11\_FUSION |  | 32 | -0.67 | -1.91 | 0.000 | 0.001 | 0.095 | 1287 | tags=72%, list=22%, signal=92% |
| 78 | DAVICIONI\_TARGETS\_OF\_PAX\_FOXO1\_FUSIONS\_UP |  | 100 | -0.58 | -1.91 | 0.000 | 0.001 | 0.101 | 984 | tags=42%, list=17%, signal=50% |
| 79 | LU\_AGING\_BRAIN\_DN |  | 114 | -0.56 | -1.90 | 0.000 | 0.001 | 0.106 | 1163 | tags=46%, list=20%, signal=57% |
| 80 | HADDAD\_T\_LYMPHOCYTE\_AND\_NK\_PROGENITOR\_DN |  | 28 | -0.68 | -1.90 | 0.000 | 0.001 | 0.109 | 489 | tags=46%, list=9%, signal=51% |
| 81 | RAGHAVACHARI\_PLATELET\_SPECIFIC\_GENES |  | 35 | -0.66 | -1.90 | 0.000 | 0.001 | 0.115 | 815 | tags=46%, list=14%, signal=53% |
| 82 | KEGG\_COMPLEMENT\_AND\_COAGULATION\_CASCADES |  | 15 | -0.78 | -1.90 | 0.000 | 0.001 | 0.117 | 1036 | tags=87%, list=18%, signal=106% |
| 83 | KORKOLA\_YOLK\_SAC\_TUMOR |  | 18 | -0.73 | -1.89 | 0.000 | 0.002 | 0.128 | 444 | tags=50%, list=8%, signal=54% |
| 84 | AMIT\_SERUM\_RESPONSE\_40\_MCF10A |  | 16 | -0.77 | -1.89 | 0.000 | 0.002 | 0.131 | 763 | tags=56%, list=13%, signal=65% |
| 85 | JAEGER\_METASTASIS\_DN |  | 52 | -0.62 | -1.89 | 0.000 | 0.002 | 0.133 | 846 | tags=56%, list=15%, signal=65% |
| 86 | VERHAAK\_AML\_WITH\_NPM1\_MUTATED\_DN |  | 85 | -0.58 | -1.89 | 0.000 | 0.002 | 0.139 | 860 | tags=44%, list=15%, signal=50% |
| 87 | BERENJENO\_TRANSFORMED\_BY\_RHOA\_DN |  | 186 | -0.54 | -1.89 | 0.000 | 0.002 | 0.146 | 972 | tags=38%, list=17%, signal=44% |
| 88 | CHIANG\_LIVER\_CANCER\_SUBCLASS\_CTNNB1\_UP |  | 40 | -0.64 | -1.89 | 0.000 | 0.002 | 0.150 | 692 | tags=43%, list=12%, signal=48% |
| 89 | HOEBEKE\_LYMPHOID\_STEM\_CELL\_DN |  | 40 | -0.64 | -1.89 | 0.000 | 0.002 | 0.154 | 1095 | tags=52%, list=19%, signal=64% |
| 90 | ONDER\_CDH1\_SIGNALING\_VIA\_CTNNB1 |  | 36 | -0.65 | -1.89 | 0.000 | 0.002 | 0.155 | 856 | tags=50%, list=15%, signal=58% |
| 91 | SENESE\_HDAC1\_AND\_HDAC2\_TARGETS\_DN |  | 62 | -0.60 | -1.88 | 0.000 | 0.002 | 0.169 | 1206 | tags=55%, list=21%, signal=69% |
| 92 | HOQUE\_METHYLATED\_IN\_CANCER |  | 20 | -0.72 | -1.88 | 0.000 | 0.002 | 0.172 | 628 | tags=60%, list=11%, signal=67% |
| 93 | PODAR\_RESPONSE\_TO\_ADAPHOSTIN\_UP |  | 70 | -0.59 | -1.88 | 0.000 | 0.002 | 0.175 | 940 | tags=41%, list=16%, signal=49% |
| 94 | JAATINEN\_HEMATOPOIETIC\_STEM\_CELL\_UP |  | 118 | -0.56 | -1.88 | 0.000 | 0.002 | 0.188 | 726 | tags=37%, list=13%, signal=42% |
| 95 | LEE\_LIVER\_CANCER\_ACOX1\_UP |  | 19 | -0.73 | -1.88 | 0.000 | 0.002 | 0.188 | 795 | tags=53%, list=14%, signal=61% |
| 96 | BERENJENO\_TRANSFORMED\_BY\_RHOA\_REVERSIBLY\_DN |  | 16 | -0.77 | -1.88 | 0.000 | 0.002 | 0.193 | 525 | tags=56%, list=9%, signal=62% |
| 97 | REACTOME\_HEMOSTASIS |  | 118 | -0.56 | -1.87 | 0.000 | 0.002 | 0.199 | 1324 | tags=46%, list=23%, signal=58% |
| 98 | KHETCHOUMIAN\_TRIM24\_TARGETS\_UP |  | 21 | -0.71 | -1.87 | 0.000 | 0.002 | 0.200 | 1103 | tags=62%, list=19%, signal=76% |
| 99 | NAKAJIMA\_MAST\_CELL |  | 15 | -0.77 | -1.87 | 0.001 | 0.002 | 0.201 | 806 | tags=73%, list=14%, signal=85% |
| 100 | RIGGI\_EWING\_SARCOMA\_PROGENITOR\_DN |  | 60 | -0.60 | -1.87 | 0.000 | 0.002 | 0.203 | 718 | tags=40%, list=13%, signal=45% |
| 101 | TONKS\_TARGETS\_OF\_RUNX1\_RUNX1T1\_FUSION\_ERYTHROCYTE\_UP |  | 50 | -0.61 | -1.87 | 0.000 | 0.002 | 0.205 | 900 | tags=48%, list=16%, signal=56% |
| 102 | CROONQUIST\_STROMAL\_STIMULATION\_UP |  | 18 | -0.73 | -1.87 | 0.002 | 0.002 | 0.205 | 642 | tags=61%, list=11%, signal=69% |
| 103 | JISON\_SICKLE\_CELL\_DISEASE\_UP |  | 79 | -0.58 | -1.87 | 0.000 | 0.002 | 0.207 | 1033 | tags=42%, list=18%, signal=50% |
| 104 | REACTOME\_PLATELET\_DEGRANULATION |  | 29 | -0.67 | -1.87 | 0.000 | 0.002 | 0.211 | 1185 | tags=55%, list=21%, signal=69% |
| 105 | TAVAZOIE\_METASTASIS |  | 29 | -0.66 | -1.87 | 0.001 | 0.002 | 0.211 | 924 | tags=45%, list=16%, signal=53% |
| 106 | GAUSSMANN\_MLL\_AF4\_FUSION\_TARGETS\_F\_UP |  | 42 | -0.62 | -1.87 | 0.000 | 0.002 | 0.217 | 1126 | tags=60%, list=20%, signal=74% |
| 107 | KAAB\_HEART\_ATRIUM\_VS\_VENTRICLE\_UP |  | 76 | -0.57 | -1.86 | 0.000 | 0.002 | 0.241 | 923 | tags=38%, list=16%, signal=45% |
| 108 | LANDIS\_ERBB2\_BREAST\_TUMORS\_324\_DN |  | 67 | -0.58 | -1.86 | 0.000 | 0.002 | 0.247 | 794 | tags=37%, list=14%, signal=43% |
| 109 | GOZGIT\_ESR1\_TARGETS\_UP |  | 44 | -0.62 | -1.86 | 0.000 | 0.002 | 0.251 | 860 | tags=43%, list=15%, signal=50% |
| 110 | KAYO\_CALORIE\_RESTRICTION\_MUSCLE\_UP |  | 25 | -0.68 | -1.86 | 0.000 | 0.003 | 0.264 | 634 | tags=48%, list=11%, signal=54% |
| 111 | GU\_PDEF\_TARGETS\_UP |  | 30 | -0.65 | -1.85 | 0.001 | 0.003 | 0.277 | 937 | tags=47%, list=16%, signal=56% |
| 112 | CHIARADONNA\_NEOPLASTIC\_TRANSFORMATION\_KRAS\_CDC25\_DN |  | 18 | -0.72 | -1.85 | 0.000 | 0.003 | 0.283 | 715 | tags=50%, list=12%, signal=57% |
| 113 | RUIZ\_TNC\_TARGETS\_UP |  | 68 | -0.58 | -1.85 | 0.000 | 0.003 | 0.287 | 1145 | tags=50%, list=20%, signal=62% |
| 114 | KONDO\_EZH2\_TARGETS |  | 43 | -0.62 | -1.85 | 0.000 | 0.003 | 0.288 | 1169 | tags=53%, list=20%, signal=67% |
| 115 | CHARAFE\_BREAST\_CANCER\_LUMINAL\_VS\_MESENCHYMAL\_DN |  | 193 | -0.53 | -1.85 | 0.000 | 0.003 | 0.292 | 1309 | tags=42%, list=23%, signal=53% |
| 116 | KIM\_RESPONSE\_TO\_TSA\_AND\_DECITABINE\_UP |  | 30 | -0.66 | -1.85 | 0.001 | 0.003 | 0.299 | 797 | tags=53%, list=14%, signal=62% |
| 117 | ICHIBA\_GRAFT\_VERSUS\_HOST\_DISEASE\_35D\_UP |  | 55 | -0.59 | -1.84 | 0.000 | 0.003 | 0.299 | 1100 | tags=51%, list=19%, signal=62% |
| 118 | TOMLINS\_PROSTATE\_CANCER\_DN |  | 21 | -0.70 | -1.84 | 0.000 | 0.003 | 0.300 | 1253 | tags=76%, list=22%, signal=97% |
| 119 | TURASHVILI\_BREAST\_DUCTAL\_CARCINOMA\_VS\_LOBULAR\_NORMAL\_DN |  | 17 | -0.73 | -1.84 | 0.001 | 0.003 | 0.304 | 563 | tags=59%, list=10%, signal=65% |
| 120 | HESS\_TARGETS\_OF\_HOXA9\_AND\_MEIS1\_DN |  | 37 | -0.63 | -1.84 | 0.001 | 0.003 | 0.304 | 1045 | tags=51%, list=18%, signal=62% |
| 121 | MCBRYAN\_PUBERTAL\_BREAST\_4\_5WK\_UP |  | 65 | -0.58 | -1.84 | 0.000 | 0.003 | 0.310 | 704 | tags=38%, list=12%, signal=43% |
| 122 | KEGG\_HEMATOPOIETIC\_CELL\_LINEAGE |  | 32 | -0.65 | -1.84 | 0.000 | 0.003 | 0.312 | 823 | tags=59%, list=14%, signal=69% |
| 123 | VART\_KSHV\_INFECTION\_ANGIOGENIC\_MARKERS\_UP |  | 49 | -0.60 | -1.84 | 0.000 | 0.003 | 0.317 | 796 | tags=43%, list=14%, signal=49% |
| 124 | PETRETTO\_CARDIAC\_HYPERTROPHY |  | 17 | -0.72 | -1.84 | 0.001 | 0.003 | 0.334 | 945 | tags=59%, list=17%, signal=70% |
| 125 | BOYLAN\_MULTIPLE\_MYELOMA\_PCA1\_UP |  | 27 | -0.67 | -1.83 | 0.000 | 0.003 | 0.356 | 875 | tags=59%, list=15%, signal=70% |
| 126 | MANALO\_HYPOXIA\_UP |  | 73 | -0.57 | -1.83 | 0.000 | 0.003 | 0.363 | 896 | tags=44%, list=16%, signal=51% |
| 127 | TARTE\_PLASMA\_CELL\_VS\_PLASMABLAST\_UP |  | 121 | -0.55 | -1.83 | 0.000 | 0.003 | 0.363 | 746 | tags=35%, list=13%, signal=39% |
| 128 | WOOD\_EBV\_EBNA1\_TARGETS\_DN |  | 22 | -0.69 | -1.83 | 0.000 | 0.003 | 0.370 | 1207 | tags=68%, list=21%, signal=86% |
| 129 | KEGG\_INTESTINAL\_IMMUNE\_NETWORK\_FOR\_IGA\_PRODUCTION |  | 15 | -0.74 | -1.82 | 0.001 | 0.003 | 0.382 | 827 | tags=60%, list=14%, signal=70% |
| 130 | SENESE\_HDAC1\_TARGETS\_DN |  | 79 | -0.57 | -1.82 | 0.000 | 0.004 | 0.385 | 1281 | tags=47%, list=22%, signal=60% |
| 131 | HOOI\_ST7\_TARGETS\_DN |  | 34 | -0.63 | -1.82 | 0.000 | 0.004 | 0.395 | 856 | tags=47%, list=15%, signal=55% |
| 132 | ROY\_WOUND\_BLOOD\_VESSEL\_UP |  | 22 | -0.67 | -1.82 | 0.001 | 0.004 | 0.398 | 588 | tags=45%, list=10%, signal=50% |
| 133 | VERRECCHIA\_EARLY\_RESPONSE\_TO\_TGFB1 |  | 17 | -0.71 | -1.82 | 0.001 | 0.004 | 0.402 | 642 | tags=59%, list=11%, signal=66% |
| 134 | HELLER\_SILENCED\_BY\_METHYLATION\_UP |  | 85 | -0.55 | -1.82 | 0.000 | 0.004 | 0.409 | 1169 | tags=46%, list=20%, signal=57% |
| 135 | ONDER\_CDH1\_TARGETS\_2\_DN |  | 115 | -0.54 | -1.82 | 0.000 | 0.004 | 0.411 | 863 | tags=36%, list=15%, signal=41% |
| 136 | HSIAO\_LIVER\_SPECIFIC\_GENES |  | 59 | -0.58 | -1.82 | 0.000 | 0.004 | 0.411 | 720 | tags=36%, list=13%, signal=40% |
| 137 | REACTOME\_PLATELET\_ACTIVATION |  | 73 | -0.57 | -1.82 | 0.000 | 0.004 | 0.416 | 1111 | tags=41%, list=19%, signal=50% |
| 138 | LINDGREN\_BLADDER\_CANCER\_HIGH\_RECURRENCE |  | 21 | -0.68 | -1.82 | 0.001 | 0.004 | 0.419 | 1217 | tags=67%, list=21%, signal=84% |
| 139 | BEGUM\_TARGETS\_OF\_PAX3\_FOXO1\_FUSION\_UP |  | 23 | -0.67 | -1.82 | 0.000 | 0.004 | 0.419 | 799 | tags=48%, list=14%, signal=55% |
| 140 | BROWNE\_HCMV\_INFECTION\_20HR\_DN |  | 55 | -0.58 | -1.82 | 0.000 | 0.004 | 0.439 | 706 | tags=36%, list=12%, signal=41% |
| 141 | TURASHVILI\_BREAST\_LOBULAR\_CARCINOMA\_VS\_DUCTAL\_NORMAL\_UP |  | 30 | -0.65 | -1.82 | 0.000 | 0.004 | 0.440 | 1120 | tags=60%, list=20%, signal=74% |
| 142 | DIAZ\_CHRONIC\_MEYLOGENOUS\_LEUKEMIA\_DN |  | 56 | -0.59 | -1.81 | 0.001 | 0.004 | 0.451 | 655 | tags=36%, list=11%, signal=40% |
| 143 | KAN\_RESPONSE\_TO\_ARSENIC\_TRIOXIDE |  | 44 | -0.60 | -1.81 | 0.000 | 0.004 | 0.453 | 871 | tags=41%, list=15%, signal=48% |
| 144 | LIEN\_BREAST\_CARCINOMA\_METAPLASTIC\_VS\_DUCTAL\_UP |  | 25 | -0.66 | -1.81 | 0.000 | 0.004 | 0.454 | 536 | tags=44%, list=9%, signal=48% |
| 145 | LEE\_LIVER\_CANCER\_CIPROFIBRATE\_UP |  | 15 | -0.74 | -1.81 | 0.001 | 0.004 | 0.462 | 799 | tags=67%, list=14%, signal=77% |
| 146 | CHIARADONNA\_NEOPLASTIC\_TRANSFORMATION\_KRAS\_DN |  | 54 | -0.58 | -1.81 | 0.000 | 0.004 | 0.463 | 989 | tags=46%, list=17%, signal=55% |
| 147 | REACTOME\_INTEGRIN\_CELL\_SURFACE\_INTERACTIONS |  | 40 | -0.60 | -1.80 | 0.000 | 0.005 | 0.499 | 1163 | tags=55%, list=20%, signal=69% |
| 148 | LINDGREN\_BLADDER\_CANCER\_CLUSTER\_2A\_DN |  | 55 | -0.58 | -1.80 | 0.002 | 0.005 | 0.518 | 736 | tags=33%, list=13%, signal=37% |
| 149 | WILCOX\_PRESPONSE\_TO\_ROGESTERONE\_DN |  | 19 | -0.70 | -1.80 | 0.000 | 0.005 | 0.529 | 1309 | tags=74%, list=23%, signal=95% |
| 150 | GOZGIT\_ESR1\_TARGETS\_DN |  | 213 | -0.51 | -1.80 | 0.000 | 0.005 | 0.533 | 848 | tags=36%, list=15%, signal=41% |
| 151 | POOLA\_INVASIVE\_BREAST\_CANCER\_DN |  | 33 | -0.62 | -1.79 | 0.001 | 0.005 | 0.541 | 1003 | tags=45%, list=18%, signal=55% |
| 152 | LEE\_LIVER\_CANCER\_SURVIVAL\_UP |  | 31 | -0.64 | -1.79 | 0.000 | 0.005 | 0.541 | 1108 | tags=45%, list=19%, signal=56% |
| 153 | MARTORIATI\_MDM4\_TARGETS\_NEUROEPITHELIUM\_DN |  | 27 | -0.64 | -1.79 | 0.001 | 0.005 | 0.558 | 787 | tags=52%, list=14%, signal=60% |
| 154 | SMID\_BREAST\_CANCER\_LUMINAL\_B\_UP |  | 34 | -0.61 | -1.78 | 0.000 | 0.006 | 0.595 | 1279 | tags=53%, list=22%, signal=68% |
| 155 | WALLACE\_PROSTATE\_CANCER\_RACE\_UP |  | 125 | -0.53 | -1.78 | 0.000 | 0.006 | 0.598 | 1120 | tags=42%, list=20%, signal=52% |
| 156 | PAPASPYRIDONOS\_UNSTABLE\_ATEROSCLEROTIC\_PLAQUE\_DN |  | 17 | -0.70 | -1.78 | 0.002 | 0.006 | 0.598 | 1206 | tags=59%, list=21%, signal=74% |
| 157 | ZHAN\_V1\_LATE\_DIFFERENTIATION\_GENES\_UP |  | 18 | -0.69 | -1.78 | 0.000 | 0.006 | 0.622 | 1245 | tags=67%, list=22%, signal=85% |
| 158 | VALK\_AML\_CLUSTER\_11 |  | 20 | -0.69 | -1.78 | 0.000 | 0.006 | 0.623 | 802 | tags=55%, list=14%, signal=64% |
| 159 | REACTOME\_FORMATION\_OF\_PLATELET\_PLUG |  | 84 | -0.54 | -1.77 | 0.000 | 0.007 | 0.660 | 1185 | tags=40%, list=21%, signal=50% |
| 160 | TONKS\_TARGETS\_OF\_RUNX1\_RUNX1T1\_FUSION\_GRANULOCYTE\_UP |  | 21 | -0.68 | -1.77 | 0.000 | 0.007 | 0.668 | 586 | tags=43%, list=10%, signal=48% |
| 161 | HORIUCHI\_WTAP\_TARGETS\_UP |  | 119 | -0.53 | -1.77 | 0.000 | 0.007 | 0.668 | 1284 | tags=50%, list=22%, signal=64% |
| 162 | ABRAHAM\_ALPC\_VS\_MULTIPLE\_MYELOMA\_UP |  | 17 | -0.71 | -1.77 | 0.000 | 0.007 | 0.673 | 1129 | tags=53%, list=20%, signal=66% |
| 163 | LANDIS\_BREAST\_CANCER\_PROGRESSION\_DN |  | 31 | -0.62 | -1.77 | 0.001 | 0.007 | 0.680 | 794 | tags=48%, list=14%, signal=56% |
| 164 | HOSHIDA\_LIVER\_CANCER\_LATE\_RECURRENCE\_UP |  | 30 | -0.62 | -1.77 | 0.001 | 0.007 | 0.682 | 1033 | tags=50%, list=18%, signal=61% |
| 165 | URS\_ADIPOCYTE\_DIFFERENTIATION\_UP |  | 19 | -0.68 | -1.77 | 0.004 | 0.007 | 0.682 | 613 | tags=47%, list=11%, signal=53% |
| 166 | SAMOLS\_TARGETS\_OF\_KHSV\_MIRNAS\_DN |  | 21 | -0.66 | -1.77 | 0.007 | 0.007 | 0.696 | 1169 | tags=57%, list=20%, signal=72% |
| 167 | EBAUER\_TARGETS\_OF\_PAX3\_FOXO1\_FUSION\_UP |  | 65 | -0.55 | -1.77 | 0.000 | 0.007 | 0.699 | 813 | tags=34%, list=14%, signal=39% |
| 168 | CAIRO\_HEPATOBLASTOMA\_DN |  | 85 | -0.54 | -1.77 | 0.000 | 0.007 | 0.706 | 897 | tags=31%, list=16%, signal=36% |
| 169 | KIM\_MYCN\_AMPLIFICATION\_TARGETS\_UP |  | 23 | -0.66 | -1.77 | 0.001 | 0.007 | 0.711 | 252 | tags=22%, list=4%, signal=23% |
| 170 | MCMURRAY\_TP53\_HRAS\_COOPERATION\_RESPONSE\_DN |  | 17 | -0.69 | -1.77 | 0.000 | 0.007 | 0.714 | 1314 | tags=71%, list=23%, signal=91% |
| 171 | TAKEDA\_TARGETS\_OF\_NUP98\_HOXA9\_FUSION\_8D\_UP |  | 44 | -0.58 | -1.76 | 0.001 | 0.008 | 0.735 | 806 | tags=43%, list=14%, signal=50% |
| 172 | DOANE\_BREAST\_CANCER\_ESR1\_UP |  | 17 | -0.70 | -1.76 | 0.000 | 0.008 | 0.750 | 944 | tags=53%, list=16%, signal=63% |
| 173 | DAVICIONI\_PAX\_FOXO1\_SIGNATURE\_IN\_ARMS\_UP |  | 16 | -0.71 | -1.76 | 0.000 | 0.008 | 0.752 | 900 | tags=56%, list=16%, signal=67% |
| 174 | KEGG\_SYSTEMIC\_LUPUS\_ERYTHEMATOSUS |  | 19 | -0.69 | -1.76 | 0.001 | 0.008 | 0.753 | 971 | tags=53%, list=17%, signal=63% |
| 175 | BECKER\_TAMOXIFEN\_RESISTANCE\_DN |  | 21 | -0.67 | -1.76 | 0.001 | 0.008 | 0.763 | 619 | tags=48%, list=11%, signal=53% |
| 176 | HOUSTIS\_ROS |  | 15 | -0.70 | -1.75 | 0.003 | 0.008 | 0.777 | 1140 | tags=67%, list=20%, signal=83% |
| 177 | REACTOME\_CELL\_SURFACE\_INTERACTIONS\_AT\_THE\_VASCULAR\_WALL |  | 41 | -0.59 | -1.75 | 0.001 | 0.008 | 0.781 | 1267 | tags=46%, list=22%, signal=59% |
| 178 | PEREZ\_TP63\_TARGETS |  | 67 | -0.55 | -1.75 | 0.000 | 0.008 | 0.786 | 796 | tags=37%, list=14%, signal=43% |
| 179 | MORI\_PLASMA\_CELL\_UP |  | 20 | -0.68 | -1.75 | 0.000 | 0.008 | 0.788 | 1715 | tags=90%, list=30%, signal=128% |
| 180 | CHEBOTAEV\_GR\_TARGETS\_UP |  | 24 | -0.65 | -1.75 | 0.000 | 0.008 | 0.790 | 695 | tags=46%, list=12%, signal=52% |
| 181 | REACTOME\_SEMAPHORIN\_INTERACTIONS |  | 26 | -0.63 | -1.75 | 0.001 | 0.009 | 0.806 | 1163 | tags=46%, list=20%, signal=58% |
| 182 | IVANOVA\_HEMATOPOIESIS\_STEM\_CELL\_LONG\_TERM |  | 24 | -0.64 | -1.74 | 0.002 | 0.009 | 0.819 | 921 | tags=38%, list=16%, signal=45% |
| 183 | BROWNE\_HCMV\_INFECTION\_24HR\_DN |  | 56 | -0.56 | -1.74 | 0.000 | 0.009 | 0.821 | 1000 | tags=46%, list=17%, signal=56% |
| 184 | TAVOR\_CEBPA\_TARGETS\_UP |  | 22 | -0.66 | -1.74 | 0.005 | 0.009 | 0.823 | 595 | tags=45%, list=10%, signal=51% |
| 185 | SPIRA\_SMOKERS\_LUNG\_CANCER\_UP |  | 20 | -0.68 | -1.74 | 0.000 | 0.009 | 0.828 | 1209 | tags=65%, list=21%, signal=82% |
| 186 | FLECHNER\_BIOPSY\_KIDNEY\_TRANSPLANT\_REJECTED\_VS\_OK\_UP |  | 43 | -0.58 | -1.74 | 0.002 | 0.009 | 0.831 | 1090 | tags=47%, list=19%, signal=57% |
| 187 | SENESE\_HDAC1\_AND\_HDAC2\_TARGETS\_UP |  | 88 | -0.53 | -1.74 | 0.000 | 0.009 | 0.832 | 1163 | tags=41%, list=20%, signal=51% |
| 188 | FONTAINE\_PAPILLARY\_THYROID\_CARCINOMA\_UP |  | 19 | -0.66 | -1.74 | 0.004 | 0.009 | 0.832 | 1218 | tags=53%, list=21%, signal=67% |
| 189 | SHIPP\_DLBCL\_VS\_FOLLICULAR\_LYMPHOMA\_DN |  | 20 | -0.66 | -1.74 | 0.001 | 0.009 | 0.841 | 1120 | tags=50%, list=20%, signal=62% |
| 190 | SATO\_SILENCED\_BY\_METHYLATION\_IN\_PANCREATIC\_CANCER\_1 |  | 103 | -0.53 | -1.74 | 0.000 | 0.009 | 0.842 | 620 | tags=28%, list=11%, signal=31% |
| 191 | BOYLAN\_MULTIPLE\_MYELOMA\_PCA3\_UP |  | 15 | -0.71 | -1.74 | 0.003 | 0.010 | 0.853 | 134 | tags=33%, list=2%, signal=34% |
| 192 | SUNG\_METASTASIS\_STROMA\_UP |  | 46 | -0.56 | -1.74 | 0.001 | 0.010 | 0.856 | 535 | tags=30%, list=9%, signal=33% |
| 193 | HELLER\_HDAC\_TARGETS\_SILENCED\_BY\_METHYLATION\_UP |  | 115 | -0.52 | -1.73 | 0.000 | 0.010 | 0.866 | 973 | tags=37%, list=17%, signal=43% |
| 194 | LINDSTEDT\_DENDRITIC\_CELL\_MATURATION\_D |  | 26 | -0.63 | -1.73 | 0.002 | 0.010 | 0.872 | 699 | tags=38%, list=12%, signal=44% |
| 195 | WINTER\_HYPOXIA\_METAGENE |  | 87 | -0.53 | -1.73 | 0.000 | 0.010 | 0.879 | 1090 | tags=39%, list=19%, signal=48% |
| 196 | ONDER\_CDH1\_TARGETS\_1\_UP |  | 48 | -0.57 | -1.73 | 0.002 | 0.011 | 0.886 | 728 | tags=38%, list=13%, signal=43% |
| 197 | GRUETZMANN\_PANCREATIC\_CANCER\_UP |  | 162 | -0.51 | -1.72 | 0.000 | 0.011 | 0.900 | 876 | tags=32%, list=15%, signal=37% |
| 198 | WOO\_LIVER\_CANCER\_RECURRENCE\_UP |  | 38 | -0.59 | -1.72 | 0.000 | 0.012 | 0.912 | 969 | tags=47%, list=17%, signal=57% |
| 199 | SENGUPTA\_NASOPHARYNGEAL\_CARCINOMA\_WITH\_LMP1\_DN |  | 33 | -0.60 | -1.72 | 0.000 | 0.012 | 0.914 | 952 | tags=45%, list=17%, signal=54% |
| 200 | STREICHER\_LSM1\_TARGETS\_UP |  | 15 | -0.70 | -1.72 | 0.004 | 0.012 | 0.914 | 619 | tags=40%, list=11%, signal=45% |
| 201 | RODRIGUES\_THYROID\_CARCINOMA\_ANAPLASTIC\_DN |  | 202 | -0.49 | -1.72 | 0.000 | 0.012 | 0.919 | 1101 | tags=37%, list=19%, signal=44% |
| 202 | FOSTER\_INFLAMMATORY\_RESPONSE\_LPS\_UP |  | 60 | -0.55 | -1.72 | 0.000 | 0.012 | 0.919 | 843 | tags=43%, list=15%, signal=50% |
| 203 | GAL\_LEUKEMIC\_STEM\_CELL\_DN |  | 73 | -0.53 | -1.72 | 0.000 | 0.012 | 0.920 | 642 | tags=29%, list=11%, signal=32% |
| 204 | WANG\_ESOPHAGUS\_CANCER\_VS\_NORMAL\_UP |  | 44 | -0.57 | -1.72 | 0.002 | 0.012 | 0.923 | 962 | tags=43%, list=17%, signal=52% |
| 205 | LE\_EGR2\_TARGETS\_DN |  | 33 | -0.59 | -1.71 | 0.002 | 0.012 | 0.924 | 1146 | tags=45%, list=20%, signal=57% |
| 206 | FRASOR\_RESPONSE\_TO\_ESTRADIOL\_DN |  | 20 | -0.65 | -1.71 | 0.002 | 0.012 | 0.925 | 804 | tags=55%, list=14%, signal=64% |
| 207 | HELLER\_SILENCED\_BY\_METHYLATION\_DN |  | 43 | -0.57 | -1.71 | 0.001 | 0.012 | 0.927 | 885 | tags=40%, list=15%, signal=46% |
| 208 | WEST\_ADRENOCORTICAL\_TUMOR\_DN |  | 182 | -0.50 | -1.71 | 0.000 | 0.013 | 0.932 | 1454 | tags=40%, list=25%, signal=52% |
| 209 | KOBAYASHI\_EGFR\_SIGNALING\_24HR\_UP |  | 39 | -0.58 | -1.71 | 0.002 | 0.013 | 0.934 | 991 | tags=38%, list=17%, signal=46% |
| 210 | SMID\_BREAST\_CANCER\_RELAPSE\_IN\_BONE\_DN |  | 86 | -0.52 | -1.71 | 0.001 | 0.013 | 0.936 | 790 | tags=34%, list=14%, signal=39% |
| 211 | KANG\_IMMORTALIZED\_BY\_TERT\_DN |  | 28 | -0.61 | -1.71 | 0.000 | 0.013 | 0.938 | 975 | tags=50%, list=17%, signal=60% |
| 212 | LEE\_LIVER\_CANCER\_DENA\_UP |  | 22 | -0.63 | -1.71 | 0.002 | 0.013 | 0.941 | 1313 | tags=59%, list=23%, signal=76% |
| 213 | LAIHO\_COLORECTAL\_CANCER\_SERRATED\_UP |  | 64 | -0.54 | -1.71 | 0.000 | 0.013 | 0.942 | 1917 | tags=63%, list=33%, signal=93% |
| 214 | ACEVEDO\_LIVER\_CANCER\_WITH\_H3K27ME3\_UP |  | 64 | -0.54 | -1.71 | 0.001 | 0.014 | 0.946 | 880 | tags=41%, list=15%, signal=47% |
| 215 | THUM\_SYSTOLIC\_HEART\_FAILURE\_UP |  | 187 | -0.49 | -1.70 | 0.000 | 0.014 | 0.950 | 1163 | tags=35%, list=20%, signal=43% |
| 216 | GRADE\_COLON\_AND\_RECTAL\_CANCER\_DN |  | 20 | -0.65 | -1.70 | 0.004 | 0.014 | 0.957 | 1132 | tags=60%, list=20%, signal=75% |
| 217 | YAO\_TEMPORAL\_RESPONSE\_TO\_PROGESTERONE\_CLUSTER\_16 |  | 20 | -0.66 | -1.70 | 0.001 | 0.014 | 0.959 | 563 | tags=40%, list=10%, signal=44% |
| 218 | KEGG\_FOCAL\_ADHESION |  | 89 | -0.52 | -1.70 | 0.000 | 0.014 | 0.960 | 1485 | tags=45%, list=26%, signal=60% |
| 219 | SETLUR\_PROSTATE\_CANCER\_TMPRSS2\_ERG\_FUSION\_UP |  | 28 | -0.61 | -1.70 | 0.006 | 0.015 | 0.965 | 533 | tags=29%, list=9%, signal=31% |
| 220 | YAO\_TEMPORAL\_RESPONSE\_TO\_PROGESTERONE\_CLUSTER\_12 |  | 37 | -0.58 | -1.70 | 0.000 | 0.015 | 0.965 | 1209 | tags=49%, list=21%, signal=61% |
| 221 | RODWELL\_AGING\_KIDNEY\_UP |  | 130 | -0.51 | -1.70 | 0.000 | 0.015 | 0.967 | 1218 | tags=39%, list=21%, signal=49% |
| 222 | KEGG\_ADHERENS\_JUNCTION |  | 25 | -0.62 | -1.69 | 0.004 | 0.015 | 0.972 | 789 | tags=40%, list=14%, signal=46% |
| 223 | HOOI\_ST7\_TARGETS\_UP |  | 32 | -0.59 | -1.69 | 0.006 | 0.015 | 0.972 | 1152 | tags=47%, list=20%, signal=58% |
| 224 | RIZKI\_TUMOR\_INVASIVENESS\_3D\_UP |  | 73 | -0.53 | -1.69 | 0.001 | 0.015 | 0.972 | 883 | tags=27%, list=15%, signal=32% |
| 225 | TURASHVILI\_BREAST\_LOBULAR\_CARCINOMA\_VS\_LOBULAR\_NORMAL\_DN |  | 33 | -0.59 | -1.69 | 0.001 | 0.016 | 0.974 | 1083 | tags=52%, list=19%, signal=63% |
| 226 | RUTELLA\_RESPONSE\_TO\_HGF\_UP |  | 207 | -0.49 | -1.69 | 0.000 | 0.016 | 0.976 | 1243 | tags=42%, list=22%, signal=51% |
| 227 | SCHUETZ\_BREAST\_CANCER\_DUCTAL\_INVASIVE\_DN |  | 20 | -0.65 | -1.69 | 0.004 | 0.016 | 0.976 | 476 | tags=35%, list=8%, signal=38% |
| 228 | KEGG\_MELANOMA |  | 28 | -0.60 | -1.69 | 0.007 | 0.016 | 0.976 | 540 | tags=29%, list=9%, signal=31% |
| 229 | HALMOS\_CEBPA\_TARGETS\_UP |  | 20 | -0.64 | -1.69 | 0.003 | 0.016 | 0.976 | 1421 | tags=65%, list=25%, signal=86% |
| 230 | SMID\_BREAST\_CANCER\_BASAL\_DN |  | 169 | -0.49 | -1.69 | 0.000 | 0.016 | 0.977 | 945 | tags=34%, list=17%, signal=40% |
| 231 | BENPORATH\_EED\_TARGETS |  | 156 | -0.50 | -1.69 | 0.000 | 0.016 | 0.978 | 884 | tags=33%, list=15%, signal=38% |
| 232 | PASQUALUCCI\_LYMPHOMA\_BY\_GC\_STAGE\_UP |  | 117 | -0.50 | -1.68 | 0.000 | 0.017 | 0.982 | 1150 | tags=45%, list=20%, signal=56% |
| 233 | DELYS\_THYROID\_CANCER\_UP |  | 136 | -0.49 | -1.68 | 0.000 | 0.018 | 0.985 | 1536 | tags=51%, list=27%, signal=68% |
| 234 | GARGALOVIC\_RESPONSE\_TO\_OXIDIZED\_PHOSPHOLIPIDS\_TURQUOISE\_UP |  | 33 | -0.58 | -1.68 | 0.000 | 0.018 | 0.985 | 1239 | tags=48%, list=22%, signal=62% |
| 235 | FLECHNER\_BIOPSY\_KIDNEY\_TRANSPLANT\_REJECTED\_VS\_OK\_DN |  | 246 | -0.48 | -1.68 | 0.000 | 0.018 | 0.986 | 1402 | tags=38%, list=24%, signal=48% |
| 236 | WEIGEL\_OXIDATIVE\_STRESS\_BY\_HNE\_AND\_TBH |  | 22 | -0.63 | -1.67 | 0.004 | 0.019 | 0.989 | 821 | tags=41%, list=14%, signal=48% |
| 237 | BROWNE\_HCMV\_INFECTION\_48HR\_DN |  | 176 | -0.48 | -1.67 | 0.000 | 0.019 | 0.989 | 1189 | tags=36%, list=21%, signal=44% |
| 238 | WIELAND\_UP\_BY\_HBV\_INFECTION |  | 47 | -0.55 | -1.67 | 0.001 | 0.019 | 0.990 | 545 | tags=30%, list=10%, signal=33% |
| 239 | KOYAMA\_SEMA3B\_TARGETS\_UP |  | 56 | -0.54 | -1.67 | 0.001 | 0.020 | 0.990 | 633 | tags=29%, list=11%, signal=32% |
| 240 | PAPASPYRIDONOS\_UNSTABLE\_ATEROSCLEROTIC\_PLAQUE\_UP |  | 28 | -0.59 | -1.67 | 0.005 | 0.020 | 0.990 | 1414 | tags=61%, list=25%, signal=80% |
| 241 | GRUETZMANN\_PANCREATIC\_CANCER\_DN |  | 80 | -0.51 | -1.67 | 0.000 | 0.020 | 0.991 | 1560 | tags=44%, list=27%, signal=59% |
| 242 | RUTELLA\_RESPONSE\_TO\_HGF\_VS\_CSF2RB\_AND\_IL4\_UP |  | 193 | -0.48 | -1.67 | 0.000 | 0.020 | 0.992 | 1243 | tags=40%, list=22%, signal=49% |
| 243 | HADDAD\_T\_LYMPHOCYTE\_AND\_NK\_PROGENITOR\_UP |  | 36 | -0.56 | -1.66 | 0.004 | 0.020 | 0.992 | 770 | tags=42%, list=13%, signal=48% |
| 244 | GINESTIER\_BREAST\_CANCER\_20Q13\_AMPLIFICATION\_UP |  | 45 | -0.55 | -1.66 | 0.001 | 0.021 | 0.993 | 1377 | tags=49%, list=24%, signal=64% |
| 245 | REACTOME\_GENERATION\_OF\_SECOND\_MESSENGER\_MOLECULES |  | 15 | -0.69 | -1.66 | 0.008 | 0.021 | 0.993 | 1202 | tags=67%, list=21%, signal=84% |
| 246 | REACTOME\_NUCLEAR\_RECEPTOR\_TRANSCRIPTION\_PATHWAY |  | 19 | -0.64 | -1.66 | 0.009 | 0.021 | 0.993 | 1409 | tags=58%, list=25%, signal=77% |
| 247 | YAO\_TEMPORAL\_RESPONSE\_TO\_PROGESTERONE\_CLUSTER\_0 |  | 24 | -0.61 | -1.66 | 0.005 | 0.022 | 0.994 | 1175 | tags=54%, list=21%, signal=68% |
| 248 | CHIARADONNA\_NEOPLASTIC\_TRANSFORMATION\_KRAS\_CDC25\_UP |  | 26 | -0.60 | -1.66 | 0.005 | 0.022 | 0.994 | 990 | tags=46%, list=17%, signal=56% |
| 249 | REACTOME\_PEPTIDE\_LIGAND\_BINDING\_RECEPTORS |  | 23 | -0.60 | -1.66 | 0.005 | 0.022 | 0.995 | 1137 | tags=61%, list=20%, signal=76% |
| 250 | SMID\_BREAST\_CANCER\_BASAL\_UP |  | 190 | -0.48 | -1.66 | 0.000 | 0.022 | 0.995 | 675 | tags=26%, list=12%, signal=28% |
| 251 | MCBRYAN\_PUBERTAL\_BREAST\_3\_4WK\_UP |  | 64 | -0.52 | -1.65 | 0.002 | 0.023 | 0.996 | 680 | tags=31%, list=12%, signal=35% |
| 252 | MONNIER\_POSTRADIATION\_TUMOR\_ESCAPE\_DN |  | 141 | -0.49 | -1.65 | 0.000 | 0.023 | 0.996 | 1207 | tags=37%, list=21%, signal=46% |
| 253 | PELLICCIOTTA\_HDAC\_IN\_ANTIGEN\_PRESENTATION\_UP |  | 42 | -0.56 | -1.65 | 0.002 | 0.023 | 0.996 | 1851 | tags=64%, list=32%, signal=94% |
| 254 | CHEN\_LVAD\_SUPPORT\_OF\_FAILING\_HEART\_DN |  | 17 | -0.66 | -1.65 | 0.004 | 0.024 | 0.997 | 675 | tags=47%, list=12%, signal=53% |
| 255 | JAEGER\_METASTASIS\_UP |  | 16 | -0.67 | -1.65 | 0.005 | 0.024 | 0.997 | 466 | tags=31%, list=8%, signal=34% |
| 256 | BRUECKNER\_TARGETS\_OF\_MIRLET7A3\_DN |  | 26 | -0.60 | -1.65 | 0.005 | 0.024 | 0.997 | 593 | tags=35%, list=10%, signal=38% |
| 257 | ALONSO\_METASTASIS\_UP |  | 80 | -0.50 | -1.65 | 0.001 | 0.024 | 0.997 | 1852 | tags=54%, list=32%, signal=78% |
| 258 | BOYLAN\_MULTIPLE\_MYELOMA\_D\_DN |  | 30 | -0.58 | -1.65 | 0.005 | 0.024 | 0.997 | 1026 | tags=40%, list=18%, signal=48% |
| 259 | BONOME\_OVARIAN\_CANCER\_POOR\_SURVIVAL\_UP |  | 15 | -0.67 | -1.64 | 0.012 | 0.025 | 0.998 | 848 | tags=47%, list=15%, signal=55% |
| 260 | REACTOME\_AXON\_GUIDANCE |  | 56 | -0.53 | -1.64 | 0.004 | 0.025 | 0.998 | 1344 | tags=43%, list=23%, signal=55% |
| 261 | WU\_SILENCED\_BY\_METHYLATION\_IN\_BLADDER\_CANCER |  | 17 | -0.66 | -1.64 | 0.011 | 0.025 | 0.999 | 808 | tags=59%, list=14%, signal=68% |
| 262 | JIANG\_TIP30\_TARGETS\_UP |  | 28 | -0.59 | -1.64 | 0.007 | 0.025 | 0.999 | 1093 | tags=43%, list=19%, signal=53% |
| 263 | LEE\_LIVER\_CANCER\_MYC\_DN |  | 22 | -0.62 | -1.64 | 0.007 | 0.025 | 0.999 | 598 | tags=36%, list=10%, signal=40% |
| 264 | RUTELLA\_RESPONSE\_TO\_CSF2RB\_AND\_IL4\_DN |  | 137 | -0.49 | -1.64 | 0.000 | 0.025 | 0.999 | 1078 | tags=37%, list=19%, signal=45% |
| 265 | AKL\_HTLV1\_INFECTION\_DN |  | 38 | -0.56 | -1.64 | 0.002 | 0.026 | 0.999 | 1300 | tags=50%, list=23%, signal=64% |
| 266 | SCHLESINGER\_METHYLATED\_DE\_NOVO\_IN\_CANCER |  | 16 | -0.66 | -1.64 | 0.014 | 0.026 | 0.999 | 1000 | tags=56%, list=17%, signal=68% |
| 267 | BILBAN\_B\_CLL\_LPL\_DN |  | 17 | -0.65 | -1.63 | 0.006 | 0.027 | 0.999 | 923 | tags=41%, list=16%, signal=49% |
| 268 | ZHOU\_INFLAMMATORY\_RESPONSE\_FIMA\_UP |  | 125 | -0.48 | -1.63 | 0.000 | 0.027 | 0.999 | 564 | tags=24%, list=10%, signal=26% |
| 269 | HOSHIDA\_LIVER\_CANCER\_SUBCLASS\_S1 |  | 127 | -0.48 | -1.63 | 0.000 | 0.027 | 0.999 | 1019 | tags=33%, list=18%, signal=39% |
| 270 | PEREZ\_TP53\_AND\_TP63\_TARGETS |  | 35 | -0.56 | -1.63 | 0.003 | 0.027 | 0.999 | 795 | tags=40%, list=14%, signal=46% |
| 271 | ODONNELL\_TFRC\_TARGETS\_UP |  | 102 | -0.49 | -1.63 | 0.001 | 0.027 | 0.999 | 1405 | tags=43%, list=25%, signal=56% |
| 272 | ELVIDGE\_HIF1A\_AND\_HIF2A\_TARGETS\_DN |  | 39 | -0.55 | -1.63 | 0.009 | 0.028 | 0.999 | 871 | tags=36%, list=15%, signal=42% |
| 273 | RIGGI\_EWING\_SARCOMA\_PROGENITOR\_UP |  | 114 | -0.48 | -1.63 | 0.000 | 0.028 | 0.999 | 797 | tags=30%, list=14%, signal=34% |
| 274 | KEGG\_AXON\_GUIDANCE |  | 36 | -0.56 | -1.63 | 0.008 | 0.028 | 0.999 | 1163 | tags=44%, list=20%, signal=55% |
| 275 | BROWNE\_HCMV\_INFECTION\_8HR\_UP |  | 48 | -0.53 | -1.63 | 0.004 | 0.029 | 0.999 | 545 | tags=23%, list=10%, signal=25% |
| 276 | TURASHVILI\_BREAST\_DUCTAL\_CARCINOMA\_VS\_DUCTAL\_NORMAL\_UP |  | 18 | -0.64 | -1.62 | 0.012 | 0.030 | 1.000 | 466 | tags=44%, list=8%, signal=48% |
| 277 | CHIARADONNA\_NEOPLASTIC\_TRANSFORMATION\_CDC25\_DN |  | 61 | -0.52 | -1.62 | 0.005 | 0.030 | 1.000 | 989 | tags=39%, list=17%, signal=47% |
| 278 | DAZARD\_RESPONSE\_TO\_UV\_SCC\_UP |  | 44 | -0.53 | -1.62 | 0.009 | 0.030 | 1.000 | 2250 | tags=73%, list=39%, signal=119% |
| 279 | XU\_GH1\_AUTOCRINE\_TARGETS\_UP |  | 56 | -0.52 | -1.62 | 0.005 | 0.030 | 1.000 | 1471 | tags=41%, list=26%, signal=55% |
| 280 | KOKKINAKIS\_METHIONINE\_DEPRIVATION\_96HR\_UP |  | 59 | -0.52 | -1.62 | 0.004 | 0.030 | 1.000 | 930 | tags=36%, list=16%, signal=42% |
| 281 | LIANG\_HEMATOPOIESIS\_STEM\_CELL\_NUMBER\_SMALL\_VS\_HUGE\_UP |  | 21 | -0.62 | -1.62 | 0.014 | 0.031 | 1.000 | 597 | tags=38%, list=10%, signal=42% |
| 282 | MCCLUNG\_CREB1\_TARGETS\_UP |  | 30 | -0.57 | -1.62 | 0.013 | 0.031 | 1.000 | 1533 | tags=57%, list=27%, signal=77% |
| 283 | TONKS\_TARGETS\_OF\_RUNX1\_RUNX1T1\_FUSION\_HSC\_DN |  | 72 | -0.50 | -1.62 | 0.002 | 0.031 | 1.000 | 990 | tags=40%, list=17%, signal=48% |
| 284 | PRAMOONJAGO\_SOX4\_TARGETS\_UP |  | 20 | -0.62 | -1.62 | 0.012 | 0.032 | 1.000 | 856 | tags=30%, list=15%, signal=35% |
| 285 | HOSHIDA\_LIVER\_CANCER\_LATE\_RECURRENCE\_DN |  | 22 | -0.61 | -1.62 | 0.007 | 0.032 | 1.000 | 395 | tags=27%, list=7%, signal=29% |
| 286 | AMIT\_SERUM\_RESPONSE\_60\_MCF10A |  | 24 | -0.59 | -1.62 | 0.013 | 0.032 | 1.000 | 1346 | tags=54%, list=24%, signal=71% |
| 287 | REACTOME\_IMMUNOREGULATORY\_INTERACTIONS\_BETWEEN\_A\_LYMPHOID\_AND\_A\_NON\_LYMPHOID\_CELL |  | 26 | -0.58 | -1.61 | 0.014 | 0.033 | 1.000 | 1181 | tags=62%, list=21%, signal=77% |
| 288 | MAHAJAN\_RESPONSE\_TO\_IL1A\_UP |  | 25 | -0.59 | -1.61 | 0.008 | 0.033 | 1.000 | 622 | tags=40%, list=11%, signal=45% |
| 289 | REACTOME\_SIGNAL\_AMPLIFICATION |  | 15 | -0.65 | -1.61 | 0.017 | 0.033 | 1.000 | 1070 | tags=40%, list=19%, signal=49% |
| 290 | NELSON\_RESPONSE\_TO\_ANDROGEN\_UP |  | 37 | -0.55 | -1.61 | 0.006 | 0.032 | 1.000 | 1181 | tags=49%, list=21%, signal=61% |
| 291 | KOKKINAKIS\_METHIONINE\_DEPRIVATION\_48HR\_UP |  | 60 | -0.52 | -1.61 | 0.001 | 0.033 | 1.000 | 598 | tags=28%, list=10%, signal=31% |
| 292 | ZHANG\_ANTIVIRAL\_RESPONSE\_TO\_RIBAVIRIN\_DN |  | 16 | -0.63 | -1.61 | 0.015 | 0.033 | 1.000 | 928 | tags=56%, list=16%, signal=67% |
| 293 | REACTOME\_SIGNALING\_IN\_IMMUNE\_SYSTEM |  | 153 | -0.47 | -1.61 | 0.000 | 0.033 | 1.000 | 1305 | tags=39%, list=23%, signal=49% |
| 294 | HOFFMANN\_IMMATURE\_TO\_MATURE\_B\_LYMPHOCYTE\_DN |  | 15 | -0.65 | -1.61 | 0.006 | 0.033 | 1.000 | 1141 | tags=60%, list=20%, signal=75% |
| 295 | SENESE\_HDAC2\_TARGETS\_DN |  | 36 | -0.55 | -1.61 | 0.005 | 0.034 | 1.000 | 1206 | tags=53%, list=21%, signal=66% |
| 296 | WANG\_METHYLATED\_IN\_BREAST\_CANCER |  | 15 | -0.66 | -1.61 | 0.018 | 0.034 | 1.000 | 804 | tags=53%, list=14%, signal=62% |
| 297 | GOLDRATH\_IMMUNE\_MEMORY |  | 30 | -0.56 | -1.60 | 0.014 | 0.034 | 1.000 | 1075 | tags=47%, list=19%, signal=57% |
| 298 | BROWNE\_HCMV\_INFECTION\_18HR\_DN |  | 55 | -0.52 | -1.60 | 0.008 | 0.034 | 1.000 | 1224 | tags=44%, list=21%, signal=55% |
| 299 | COULOUARN\_TEMPORAL\_TGFB1\_SIGNATURE\_UP |  | 33 | -0.55 | -1.60 | 0.005 | 0.035 | 1.000 | 930 | tags=36%, list=16%, signal=43% |
| 300 | BIOCARTA\_ECM\_PATHWAY |  | 17 | -0.63 | -1.60 | 0.015 | 0.036 | 1.000 | 1305 | tags=53%, list=23%, signal=68% |
| 301 | KUMAR\_TARGETS\_OF\_MLL\_AF9\_FUSION |  | 170 | -0.47 | -1.60 | 0.000 | 0.036 | 1.000 | 1100 | tags=36%, list=19%, signal=44% |
| 302 | RASHI\_RESPONSE\_TO\_IONIZING\_RADIATION\_1 |  | 18 | -0.63 | -1.59 | 0.011 | 0.038 | 1.000 | 718 | tags=28%, list=13%, signal=32% |
| 303 | REACTOME\_GPCR\_LIGAND\_BINDING |  | 48 | -0.52 | -1.59 | 0.010 | 0.038 | 1.000 | 1137 | tags=48%, list=20%, signal=59% |
| 304 | LANDIS\_ERBB2\_BREAST\_TUMORS\_324\_UP |  | 60 | -0.51 | -1.59 | 0.005 | 0.038 | 1.000 | 1439 | tags=45%, list=25%, signal=59% |
| 305 | TAKEDA\_TARGETS\_OF\_NUP98\_HOXA9\_FUSION\_10D\_UP |  | 61 | -0.51 | -1.59 | 0.001 | 0.038 | 1.000 | 842 | tags=38%, list=15%, signal=44% |
| 306 | KEGG\_TIGHT\_JUNCTION |  | 46 | -0.52 | -1.59 | 0.008 | 0.039 | 1.000 | 723 | tags=33%, list=13%, signal=37% |
| 307 | BROWNE\_HCMV\_INFECTION\_12HR\_DN |  | 42 | -0.53 | -1.59 | 0.009 | 0.039 | 1.000 | 682 | tags=31%, list=12%, signal=35% |
| 308 | KEGG\_LEUKOCYTE\_TRANSENDOTHELIAL\_MIGRATION |  | 53 | -0.51 | -1.59 | 0.008 | 0.040 | 1.000 | 1309 | tags=36%, list=23%, signal=46% |
| 309 | BOYAULT\_LIVER\_CANCER\_SUBCLASS\_G1\_DN |  | 18 | -0.61 | -1.59 | 0.015 | 0.041 | 1.000 | 1674 | tags=67%, list=29%, signal=94% |
| 310 | MARKEY\_RB1\_ACUTE\_LOF\_UP |  | 99 | -0.48 | -1.58 | 0.001 | 0.043 | 1.000 | 1139 | tags=39%, list=20%, signal=48% |
| 311 | FULCHER\_INFLAMMATORY\_RESPONSE\_LECTIN\_VS\_LPS\_DN |  | 190 | -0.46 | -1.58 | 0.000 | 0.044 | 1.000 | 1519 | tags=43%, list=27%, signal=57% |
| 312 | BOYLAN\_MULTIPLE\_MYELOMA\_D\_CLUSTER\_DN |  | 16 | -0.62 | -1.58 | 0.019 | 0.044 | 1.000 | 439 | tags=31%, list=8%, signal=34% |
| 313 | CHEN\_LVAD\_SUPPORT\_OF\_FAILING\_HEART\_UP |  | 35 | -0.54 | -1.58 | 0.006 | 0.044 | 1.000 | 663 | tags=31%, list=12%, signal=35% |
| 314 | ALONSO\_METASTASIS\_EMT\_UP |  | 18 | -0.61 | -1.58 | 0.022 | 0.044 | 1.000 | 1414 | tags=61%, list=25%, signal=81% |
| 315 | BARRIER\_COLON\_CANCER\_RECURRENCE\_UP |  | 22 | -0.59 | -1.58 | 0.012 | 0.044 | 1.000 | 1338 | tags=55%, list=23%, signal=71% |
| 316 | COATES\_MACROPHAGE\_M1\_VS\_M2\_DN |  | 24 | -0.59 | -1.58 | 0.017 | 0.044 | 1.000 | 1090 | tags=46%, list=19%, signal=56% |
| 317 | DANG\_REGULATED\_BY\_MYC\_DN |  | 121 | -0.47 | -1.57 | 0.001 | 0.046 | 1.000 | 1218 | tags=39%, list=21%, signal=48% |
| 318 | HARRIS\_HYPOXIA |  | 32 | -0.55 | -1.57 | 0.016 | 0.046 | 1.000 | 923 | tags=41%, list=16%, signal=48% |
| 319 | LEE\_NEURAL\_CREST\_STEM\_CELL\_DN |  | 31 | -0.55 | -1.57 | 0.011 | 0.046 | 1.000 | 1806 | tags=61%, list=32%, signal=89% |
| 320 | REACTOME\_CELL\_JUNCTION\_ORGANIZATION |  | 20 | -0.61 | -1.57 | 0.022 | 0.046 | 1.000 | 678 | tags=40%, list=12%, signal=45% |
| 321 | IVANOVA\_HEMATOPOIESIS\_MATURE\_CELL |  | 42 | -0.52 | -1.57 | 0.010 | 0.046 | 1.000 | 1414 | tags=43%, list=25%, signal=57% |
| 322 | DAVICIONI\_MOLECULAR\_ARMS\_VS\_ERMS\_DN |  | 39 | -0.54 | -1.57 | 0.007 | 0.046 | 1.000 | 1376 | tags=54%, list=24%, signal=70% |
| 323 | KEGG\_PPAR\_SIGNALING\_PATHWAY |  | 28 | -0.56 | -1.57 | 0.015 | 0.048 | 1.000 | 767 | tags=39%, list=13%, signal=45% |
| 324 | BILD\_HRAS\_ONCOGENIC\_SIGNATURE |  | 93 | -0.47 | -1.56 | 0.002 | 0.050 | 1.000 | 1325 | tags=34%, list=23%, signal=44% |
| 325 | FRIDMAN\_SENESCENCE\_UP |  | 34 | -0.55 | -1.56 | 0.015 | 0.050 | 1.000 | 622 | tags=41%, list=11%, signal=46% |
| 326 | ZHAN\_MULTIPLE\_MYELOMA\_CD1\_VS\_CD2\_UP |  | 18 | -0.61 | -1.56 | 0.022 | 0.050 | 1.000 | 297 | tags=22%, list=5%, signal=23% |
| 327 | TARTE\_PLASMA\_CELL\_VS\_B\_LYMPHOCYTE\_UP |  | 51 | -0.51 | -1.56 | 0.011 | 0.051 | 1.000 | 1194 | tags=51%, list=21%, signal=64% |
| 328 | LENAOUR\_DENDRITIC\_CELL\_MATURATION\_DN |  | 54 | -0.50 | -1.56 | 0.004 | 0.050 | 1.000 | 1200 | tags=43%, list=21%, signal=53% |
| 329 | LINDSTEDT\_DENDRITIC\_CELL\_MATURATION\_C |  | 26 | -0.56 | -1.56 | 0.020 | 0.050 | 1.000 | 877 | tags=38%, list=15%, signal=45% |
| 330 | ROSS\_AML\_WITH\_PML\_RARA\_FUSION |  | 27 | -0.57 | -1.56 | 0.022 | 0.050 | 1.000 | 1354 | tags=56%, list=24%, signal=72% |
| 331 | CAIRO\_LIVER\_DEVELOPMENT\_UP |  | 56 | -0.50 | -1.56 | 0.003 | 0.050 | 1.000 | 1015 | tags=38%, list=18%, signal=45% |
| 332 | ZHANG\_PROLIFERATING\_VS\_QUIESCENT |  | 27 | -0.57 | -1.56 | 0.019 | 0.050 | 1.000 | 1891 | tags=70%, list=33%, signal=105% |
| 333 | RODRIGUES\_THYROID\_CARCINOMA\_DN |  | 25 | -0.56 | -1.56 | 0.019 | 0.051 | 1.000 | 838 | tags=40%, list=15%, signal=47% |
| 334 | CAIRO\_LIVER\_DEVELOPMENT\_DN |  | 64 | -0.50 | -1.56 | 0.003 | 0.051 | 1.000 | 694 | tags=30%, list=12%, signal=33% |
| 335 | TAKEDA\_TARGETS\_OF\_NUP98\_HOXA9\_FUSION\_3D\_UP |  | 56 | -0.49 | -1.56 | 0.011 | 0.052 | 1.000 | 952 | tags=36%, list=17%, signal=42% |
| 336 | MASSARWEH\_TAMOXIFEN\_RESISTANCE\_UP |  | 234 | -0.45 | -1.55 | 0.000 | 0.052 | 1.000 | 1408 | tags=41%, list=25%, signal=53% |
| 337 | RADMACHER\_AML\_PROGNOSIS |  | 26 | -0.57 | -1.55 | 0.027 | 0.052 | 1.000 | 838 | tags=31%, list=15%, signal=36% |
| 338 | LEE\_LIVER\_CANCER\_MYC\_TGFA\_UP |  | 19 | -0.60 | -1.55 | 0.022 | 0.052 | 1.000 | 1313 | tags=63%, list=23%, signal=82% |
| 339 | AMUNDSON\_POOR\_SURVIVAL\_AFTER\_GAMMA\_RADIATION\_8G |  | 35 | -0.53 | -1.55 | 0.014 | 0.052 | 1.000 | 1103 | tags=34%, list=19%, signal=42% |
| 340 | MCBRYAN\_PUBERTAL\_BREAST\_5\_6WK\_UP |  | 49 | -0.51 | -1.55 | 0.010 | 0.052 | 1.000 | 704 | tags=27%, list=12%, signal=30% |
| 341 | LEE\_LIVER\_CANCER\_MYC\_E2F1\_UP |  | 18 | -0.61 | -1.55 | 0.018 | 0.052 | 1.000 | 1313 | tags=56%, list=23%, signal=72% |
| 342 | UEDA\_CENTRAL\_CLOCK |  | 39 | -0.53 | -1.55 | 0.019 | 0.052 | 1.000 | 1439 | tags=49%, list=25%, signal=65% |
| 343 | FLECHNER\_BIOPSY\_KIDNEY\_TRANSPLANT\_OK\_VS\_DONOR\_UP |  | 290 | -0.44 | -1.55 | 0.000 | 0.054 | 1.000 | 1506 | tags=39%, list=26%, signal=50% |
| 344 | PACHER\_TARGETS\_OF\_IGF1\_AND\_IGF2\_UP |  | 16 | -0.63 | -1.55 | 0.022 | 0.054 | 1.000 | 1324 | tags=50%, list=23%, signal=65% |
| 345 | CHEBOTAEV\_GR\_TARGETS\_DN |  | 30 | -0.55 | -1.55 | 0.019 | 0.055 | 1.000 | 559 | tags=33%, list=10%, signal=37% |
| 346 | ENGELMANN\_CANCER\_PROGENITORS\_UP |  | 25 | -0.56 | -1.55 | 0.026 | 0.055 | 1.000 | 578 | tags=32%, list=10%, signal=35% |
| 347 | BOYAULT\_LIVER\_CANCER\_SUBCLASS\_G6\_UP |  | 28 | -0.56 | -1.55 | 0.016 | 0.055 | 1.000 | 483 | tags=32%, list=8%, signal=35% |
| 348 | ELVIDGE\_HIF1A\_TARGETS\_DN |  | 34 | -0.54 | -1.55 | 0.018 | 0.055 | 1.000 | 871 | tags=35%, list=15%, signal=41% |
| 349 | BASAKI\_YBX1\_TARGETS\_DN |  | 141 | -0.45 | -1.55 | 0.000 | 0.055 | 1.000 | 1461 | tags=39%, list=26%, signal=51% |
| 350 | MCLACHLAN\_DENTAL\_CARIES\_DN |  | 96 | -0.47 | -1.54 | 0.004 | 0.056 | 1.000 | 1202 | tags=39%, list=21%, signal=48% |
| 351 | YANG\_BREAST\_CANCER\_ESR1\_LASER\_DN |  | 23 | -0.58 | -1.54 | 0.020 | 0.056 | 1.000 | 574 | tags=35%, list=10%, signal=39% |
| 352 | KEGG\_NEUROACTIVE\_LIGAND\_RECEPTOR\_INTERACTION |  | 28 | -0.56 | -1.54 | 0.030 | 0.056 | 1.000 | 1137 | tags=54%, list=20%, signal=67% |
| 353 | REACTOME\_INSULIN\_SYNTHESIS\_AND\_SECRETION |  | 40 | -0.52 | -1.54 | 0.020 | 0.056 | 1.000 | 2058 | tags=52%, list=36%, signal=81% |
| 354 | REACTOME\_COSTIMULATION\_BY\_THE\_CD28\_FAMILY |  | 39 | -0.52 | -1.54 | 0.015 | 0.057 | 1.000 | 1529 | tags=49%, list=27%, signal=66% |
| 355 | YAGI\_AML\_FAB\_MARKERS |  | 81 | -0.47 | -1.54 | 0.006 | 0.057 | 1.000 | 623 | tags=30%, list=11%, signal=33% |
| 356 | ICHIBA\_GRAFT\_VERSUS\_HOST\_DISEASE\_D7\_UP |  | 47 | -0.51 | -1.54 | 0.013 | 0.058 | 1.000 | 1109 | tags=43%, list=19%, signal=52% |
| 357 | HAN\_SATB1\_TARGETS\_DN |  | 125 | -0.46 | -1.54 | 0.000 | 0.058 | 1.000 | 848 | tags=30%, list=15%, signal=34% |
| 358 | KIM\_MYC\_AMPLIFICATION\_TARGETS\_DN |  | 26 | -0.55 | -1.53 | 0.030 | 0.060 | 1.000 | 258 | tags=23%, list=5%, signal=24% |
| 359 | SENGUPTA\_NASOPHARYNGEAL\_CARCINOMA\_WITH\_LMP1\_UP |  | 167 | -0.44 | -1.53 | 0.000 | 0.061 | 1.000 | 1370 | tags=35%, list=24%, signal=44% |
| 360 | PARK\_APL\_PATHOGENESIS\_DN |  | 18 | -0.60 | -1.53 | 0.028 | 0.061 | 1.000 | 360 | tags=28%, list=6%, signal=30% |
| 361 | WELCSH\_BRCA1\_TARGETS\_1\_UP |  | 93 | -0.47 | -1.53 | 0.005 | 0.062 | 1.000 | 1784 | tags=51%, list=31%, signal=72% |
| 362 | KEGG\_GLUTATHIONE\_METABOLISM |  | 16 | -0.62 | -1.53 | 0.028 | 0.062 | 1.000 | 565 | tags=31%, list=10%, signal=35% |
| 363 | ALCALAY\_AML\_BY\_NPM1\_LOCALIZATION\_DN |  | 77 | -0.47 | -1.53 | 0.004 | 0.063 | 1.000 | 558 | tags=23%, list=10%, signal=26% |
| 364 | MARTINEZ\_RESPONSE\_TO\_TRABECTEDIN\_UP |  | 23 | -0.57 | -1.53 | 0.028 | 0.063 | 1.000 | 1059 | tags=43%, list=19%, signal=53% |
| 365 | MAHAJAN\_RESPONSE\_TO\_IL1A\_DN |  | 32 | -0.54 | -1.53 | 0.024 | 0.063 | 1.000 | 1510 | tags=53%, list=26%, signal=72% |
| 366 | CAIRO\_HEPATOBLASTOMA\_CLASSES\_DN |  | 78 | -0.48 | -1.53 | 0.007 | 0.064 | 1.000 | 836 | tags=31%, list=15%, signal=36% |
| 367 | CHANG\_IMMORTALIZED\_BY\_HPV31\_DN |  | 18 | -0.59 | -1.53 | 0.030 | 0.064 | 1.000 | 158 | tags=22%, list=3%, signal=23% |
| 368 | ELVIDGE\_HYPOXIA\_BY\_DMOG\_UP |  | 54 | -0.49 | -1.52 | 0.013 | 0.065 | 1.000 | 923 | tags=35%, list=16%, signal=42% |
| 369 | AIYAR\_COBRA1\_TARGETS\_UP |  | 16 | -0.61 | -1.52 | 0.024 | 0.065 | 1.000 | 828 | tags=44%, list=14%, signal=51% |
| 370 | AMIT\_SERUM\_RESPONSE\_240\_MCF10A |  | 20 | -0.59 | -1.52 | 0.024 | 0.065 | 1.000 | 937 | tags=45%, list=16%, signal=54% |
| 371 | NUYTTEN\_EZH2\_TARGETS\_UP |  | 385 | -0.43 | -1.52 | 0.000 | 0.065 | 1.000 | 1528 | tags=39%, list=27%, signal=50% |
| 372 | SABATES\_COLORECTAL\_ADENOMA\_UP |  | 17 | -0.60 | -1.52 | 0.041 | 0.066 | 1.000 | 615 | tags=35%, list=11%, signal=39% |
| 373 | NAKAMURA\_TUMOR\_ZONE\_PERIPHERAL\_VS\_CENTRAL\_DN |  | 223 | -0.44 | -1.52 | 0.000 | 0.066 | 1.000 | 1600 | tags=38%, list=28%, signal=51% |
| 374 | VANHARANTA\_UTERINE\_FIBROID\_DN |  | 24 | -0.56 | -1.52 | 0.034 | 0.067 | 1.000 | 1325 | tags=42%, list=23%, signal=54% |
| 375 | IWANAGA\_CARCINOGENESIS\_BY\_KRAS\_PTEN\_DN |  | 122 | -0.45 | -1.52 | 0.005 | 0.067 | 1.000 | 972 | tags=30%, list=17%, signal=36% |
| 376 | BROWNE\_HCMV\_INFECTION\_16HR\_DN |  | 31 | -0.54 | -1.52 | 0.014 | 0.067 | 1.000 | 1253 | tags=52%, list=22%, signal=66% |
| 377 | VANTVEER\_BREAST\_CANCER\_BRCA1\_DN |  | 16 | -0.61 | -1.52 | 0.045 | 0.068 | 1.000 | 1000 | tags=50%, list=17%, signal=60% |
| 378 | KEGG\_VIRAL\_MYOCARDITIS |  | 21 | -0.57 | -1.51 | 0.035 | 0.070 | 1.000 | 1146 | tags=43%, list=20%, signal=53% |
| 379 | RUTELLA\_RESPONSE\_TO\_CSF2RB\_AND\_IL4\_UP |  | 177 | -0.44 | -1.51 | 0.002 | 0.071 | 1.000 | 990 | tags=32%, list=17%, signal=37% |
| 380 | BOYLAN\_MULTIPLE\_MYELOMA\_PCA3\_DN |  | 26 | -0.55 | -1.51 | 0.028 | 0.071 | 1.000 | 1248 | tags=38%, list=22%, signal=49% |
| 381 | VALK\_AML\_CLUSTER\_3 |  | 15 | -0.63 | -1.51 | 0.042 | 0.073 | 1.000 | 912 | tags=47%, list=16%, signal=55% |
| 382 | HINATA\_NFKB\_TARGETS\_FIBROBLAST\_UP |  | 27 | -0.55 | -1.51 | 0.031 | 0.075 | 1.000 | 800 | tags=33%, list=14%, signal=39% |
| 383 | HAHTOLA\_SEZARY\_SYNDROM\_DN |  | 16 | -0.61 | -1.50 | 0.034 | 0.078 | 1.000 | 1573 | tags=69%, list=27%, signal=95% |
| 384 | MARTINEZ\_RB1\_AND\_TP53\_TARGETS\_UP |  | 215 | -0.44 | -1.50 | 0.000 | 0.077 | 1.000 | 1566 | tags=41%, list=27%, signal=55% |
| 385 | MCLACHLAN\_DENTAL\_CARIES\_UP |  | 90 | -0.46 | -1.50 | 0.008 | 0.079 | 1.000 | 1202 | tags=38%, list=21%, signal=47% |
| 386 | BIDUS\_METASTASIS\_DN |  | 60 | -0.48 | -1.50 | 0.011 | 0.079 | 1.000 | 1003 | tags=33%, list=18%, signal=40% |
| 387 | PROVENZANI\_METASTASIS\_DN |  | 66 | -0.48 | -1.49 | 0.010 | 0.082 | 1.000 | 1072 | tags=38%, list=19%, signal=46% |
| 388 | XU\_GH1\_EXOGENOUS\_TARGETS\_DN |  | 35 | -0.52 | -1.49 | 0.034 | 0.085 | 1.000 | 1239 | tags=43%, list=22%, signal=54% |
| 389 | LANDIS\_ERBB2\_BREAST\_TUMORS\_65\_DN |  | 18 | -0.58 | -1.49 | 0.040 | 0.085 | 1.000 | 1563 | tags=67%, list=27%, signal=91% |
| 390 | BONOME\_OVARIAN\_CANCER\_SURVIVAL\_SUBOPTIMAL\_DEBULKING |  | 179 | -0.43 | -1.49 | 0.001 | 0.085 | 1.000 | 876 | tags=26%, list=15%, signal=29% |
| 391 | REACTOME\_G\_ALPHA\_Q\_SIGNALLING\_EVENTS |  | 27 | -0.54 | -1.49 | 0.029 | 0.086 | 1.000 | 1070 | tags=41%, list=19%, signal=50% |
| 392 | ONKEN\_UVEAL\_MELANOMA\_DN |  | 252 | -0.42 | -1.49 | 0.001 | 0.086 | 1.000 | 1290 | tags=31%, list=23%, signal=38% |
| 393 | ELVIDGE\_HYPOXIA\_UP |  | 69 | -0.47 | -1.49 | 0.006 | 0.086 | 1.000 | 923 | tags=32%, list=16%, signal=38% |
| 394 | APPEL\_IMATINIB\_RESPONSE |  | 21 | -0.56 | -1.49 | 0.036 | 0.086 | 1.000 | 1414 | tags=52%, list=25%, signal=69% |
| 395 | FONTAINE\_PAPILLARY\_THYROID\_CARCINOMA\_DN |  | 19 | -0.58 | -1.49 | 0.034 | 0.086 | 1.000 | 746 | tags=37%, list=13%, signal=42% |
| 396 | UZONYI\_RESPONSE\_TO\_LEUKOTRIENE\_AND\_THROMBIN |  | 16 | -0.59 | -1.49 | 0.059 | 0.086 | 1.000 | 888 | tags=50%, list=16%, signal=59% |
| 397 | GRAHAM\_CML\_QUIESCENT\_VS\_NORMAL\_DIVIDING\_UP |  | 22 | -0.56 | -1.49 | 0.039 | 0.086 | 1.000 | 752 | tags=32%, list=13%, signal=36% |
| 398 | HOSHIDA\_LIVER\_CANCER\_SUBCLASS\_S3 |  | 105 | -0.45 | -1.49 | 0.008 | 0.086 | 1.000 | 1542 | tags=42%, list=27%, signal=56% |
| 399 | JAZAERI\_BREAST\_CANCER\_BRCA1\_VS\_BRCA2\_DN |  | 20 | -0.56 | -1.49 | 0.053 | 0.086 | 1.000 | 291 | tags=20%, list=5%, signal=21% |
| 400 | DAVICIONI\_TARGETS\_OF\_PAX\_FOXO1\_FUSIONS\_DN |  | 20 | -0.57 | -1.49 | 0.047 | 0.087 | 1.000 | 675 | tags=35%, list=12%, signal=40% |
| 401 | HELLER\_HDAC\_TARGETS\_UP |  | 90 | -0.45 | -1.48 | 0.004 | 0.088 | 1.000 | 973 | tags=30%, list=17%, signal=36% |
| 402 | CASORELLI\_ACUTE\_PROMYELOCYTIC\_LEUKEMIA\_UP |  | 70 | -0.47 | -1.48 | 0.008 | 0.089 | 1.000 | 1491 | tags=40%, list=26%, signal=53% |
| 403 | KAAB\_FAILED\_HEART\_VENTRICLE\_DN |  | 20 | -0.56 | -1.48 | 0.039 | 0.089 | 1.000 | 562 | tags=25%, list=10%, signal=28% |
| 404 | HELLEBREKERS\_SILENCED\_DURING\_TUMOR\_ANGIOGENESIS |  | 15 | -0.61 | -1.48 | 0.049 | 0.091 | 1.000 | 744 | tags=40%, list=13%, signal=46% |
| 405 | KRIGE\_RESPONSE\_TO\_TOSEDOSTAT\_24HR\_UP |  | 337 | -0.42 | -1.48 | 0.000 | 0.091 | 1.000 | 1757 | tags=45%, list=31%, signal=60% |
| 406 | VART\_KSHV\_INFECTION\_ANGIOGENIC\_MARKERS\_DN |  | 32 | -0.52 | -1.48 | 0.041 | 0.091 | 1.000 | 746 | tags=41%, list=13%, signal=46% |
| 407 | HELLER\_HDAC\_TARGETS\_SILENCED\_BY\_METHYLATION\_DN |  | 110 | -0.44 | -1.48 | 0.003 | 0.092 | 1.000 | 990 | tags=32%, list=17%, signal=38% |
| 408 | RUTELLA\_RESPONSE\_TO\_HGF\_VS\_CSF2RB\_AND\_IL4\_DN |  | 121 | -0.44 | -1.48 | 0.005 | 0.093 | 1.000 | 974 | tags=31%, list=17%, signal=37% |
| 409 | MCBRYAN\_PUBERTAL\_BREAST\_6\_7WK\_DN |  | 28 | -0.54 | -1.48 | 0.029 | 0.093 | 1.000 | 697 | tags=36%, list=12%, signal=40% |
| 410 | LANDIS\_ERBB2\_BREAST\_PRENEOPLASTIC\_DN |  | 31 | -0.52 | -1.47 | 0.029 | 0.093 | 1.000 | 1710 | tags=65%, list=30%, signal=92% |
| 411 | STEARMAN\_LUNG\_CANCER\_EARLY\_VS\_LATE\_UP |  | 64 | -0.46 | -1.47 | 0.015 | 0.094 | 1.000 | 1845 | tags=53%, list=32%, signal=78% |
| 412 | DASU\_IL6\_SIGNALING\_UP |  | 16 | -0.59 | -1.47 | 0.034 | 0.094 | 1.000 | 675 | tags=38%, list=12%, signal=42% |
| 413 | ACEVEDO\_METHYLATED\_IN\_LIVER\_CANCER\_DN |  | 171 | -0.43 | -1.47 | 0.001 | 0.094 | 1.000 | 1227 | tags=32%, list=21%, signal=39% |
| 414 | CADWELL\_ATG16L1\_TARGETS\_UP |  | 15 | -0.60 | -1.47 | 0.047 | 0.094 | 1.000 | 289 | tags=27%, list=5%, signal=28% |
| 415 | ADDYA\_ERYTHROID\_DIFFERENTIATION\_BY\_HEMIN |  | 24 | -0.55 | -1.47 | 0.040 | 0.095 | 1.000 | 1064 | tags=46%, list=19%, signal=56% |
| 416 | LINDSTEDT\_DENDRITIC\_CELL\_MATURATION\_A |  | 27 | -0.53 | -1.47 | 0.042 | 0.095 | 1.000 | 888 | tags=41%, list=16%, signal=48% |
| 417 | VERRECCHIA\_DELAYED\_RESPONSE\_TO\_TGFB1 |  | 15 | -0.60 | -1.47 | 0.040 | 0.095 | 1.000 | 937 | tags=40%, list=16%, signal=48% |
| 418 | HENDRICKS\_SMARCA4\_TARGETS\_DN |  | 21 | -0.57 | -1.47 | 0.042 | 0.096 | 1.000 | 1690 | tags=57%, list=30%, signal=81% |
| 419 | LI\_AMPLIFIED\_IN\_LUNG\_CANCER |  | 51 | -0.48 | -1.47 | 0.035 | 0.097 | 1.000 | 1063 | tags=29%, list=19%, signal=36% |
| 420 | LUI\_THYROID\_CANCER\_PAX8\_PPARG\_DN |  | 21 | -0.56 | -1.47 | 0.047 | 0.098 | 1.000 | 1181 | tags=33%, list=21%, signal=42% |
| 421 | NIKOLSKY\_BREAST\_CANCER\_11Q12\_Q14\_AMPLICON |  | 46 | -0.49 | -1.46 | 0.033 | 0.100 | 1.000 | 1019 | tags=35%, list=18%, signal=42% |
| 422 | POOLA\_INVASIVE\_BREAST\_CANCER\_UP |  | 122 | -0.43 | -1.46 | 0.006 | 0.102 | 1.000 | 1120 | tags=31%, list=20%, signal=38% |
| 423 | LEE\_LIVER\_CANCER\_CIPROFIBRATE\_DN |  | 16 | -0.58 | -1.46 | 0.050 | 0.102 | 1.000 | 590 | tags=31%, list=10%, signal=35% |
| 424 | LEE\_LIVER\_CANCER\_MYC\_UP |  | 23 | -0.54 | -1.46 | 0.054 | 0.102 | 1.000 | 476 | tags=22%, list=8%, signal=24% |
| 425 | KEGG\_T\_CELL\_RECEPTOR\_SIGNALING\_PATHWAY |  | 61 | -0.46 | -1.46 | 0.026 | 0.102 | 1.000 | 1346 | tags=34%, list=24%, signal=45% |
| 426 | REACTOME\_CTLA4\_INHIBITORY\_SIGNALING |  | 16 | -0.59 | -1.46 | 0.056 | 0.104 | 1.000 | 985 | tags=44%, list=17%, signal=53% |
| 427 | DODD\_NASOPHARYNGEAL\_CARCINOMA\_UP |  | 380 | -0.41 | -1.46 | 0.000 | 0.104 | 1.000 | 944 | tags=26%, list=16%, signal=29% |
| 428 | GARGALOVIC\_RESPONSE\_TO\_OXIDIZED\_PHOSPHOLIPIDS\_BLUE\_UP |  | 54 | -0.47 | -1.46 | 0.034 | 0.104 | 1.000 | 1957 | tags=46%, list=34%, signal=70% |
| 429 | GENTILE\_UV\_RESPONSE\_CLUSTER\_D6 |  | 19 | -0.57 | -1.46 | 0.059 | 0.105 | 1.000 | 898 | tags=42%, list=16%, signal=50% |
| 430 | XU\_HGF\_TARGETS\_REPRESSED\_BY\_AKT1\_DN |  | 22 | -0.55 | -1.46 | 0.058 | 0.105 | 1.000 | 1139 | tags=45%, list=20%, signal=57% |
| 431 | KEGG\_ARRHYTHMOGENIC\_RIGHT\_VENTRICULAR\_CARDIOMYOPATHY\_ARVC |  | 26 | -0.53 | -1.45 | 0.058 | 0.106 | 1.000 | 1163 | tags=46%, list=20%, signal=58% |
| 432 | REACTOME\_SIGNALING\_BY\_PDGF |  | 30 | -0.51 | -1.45 | 0.042 | 0.106 | 1.000 | 898 | tags=30%, list=16%, signal=35% |
| 433 | MARTINEZ\_TP53\_TARGETS\_UP |  | 214 | -0.42 | -1.45 | 0.002 | 0.106 | 1.000 | 1570 | tags=39%, list=27%, signal=51% |
| 434 | CERVERA\_SDHB\_TARGETS\_2 |  | 37 | -0.49 | -1.45 | 0.050 | 0.108 | 1.000 | 991 | tags=43%, list=17%, signal=52% |
| 435 | HAHTOLA\_MYCOSIS\_FUNGOIDES\_SKIN\_UP |  | 87 | -0.44 | -1.45 | 0.015 | 0.109 | 1.000 | 1460 | tags=34%, list=26%, signal=46% |
| 436 | KYNG\_DNA\_DAMAGE\_UP |  | 47 | -0.48 | -1.45 | 0.029 | 0.109 | 1.000 | 1737 | tags=47%, list=30%, signal=67% |
| 437 | AMIT\_EGF\_RESPONSE\_40\_HELA |  | 18 | -0.56 | -1.45 | 0.069 | 0.110 | 1.000 | 1183 | tags=44%, list=21%, signal=56% |
| 438 | UDAYAKUMAR\_MED1\_TARGETS\_DN |  | 101 | -0.44 | -1.45 | 0.018 | 0.111 | 1.000 | 1075 | tags=30%, list=19%, signal=36% |
| 439 | IZADPANAH\_STEM\_CELL\_ADIPOSE\_VS\_BONE\_UP |  | 49 | -0.48 | -1.45 | 0.027 | 0.111 | 1.000 | 796 | tags=29%, list=14%, signal=33% |
| 440 | KEGG\_TGF\_BETA\_SIGNALING\_PATHWAY |  | 31 | -0.51 | -1.45 | 0.048 | 0.110 | 1.000 | 1303 | tags=39%, list=23%, signal=50% |
| 441 | FALVELLA\_SMOKERS\_WITH\_LUNG\_CANCER |  | 31 | -0.51 | -1.45 | 0.036 | 0.111 | 1.000 | 1295 | tags=35%, list=23%, signal=46% |
| 442 | EBAUER\_MYOGENIC\_TARGETS\_OF\_PAX3\_FOXO1\_FUSION |  | 16 | -0.59 | -1.44 | 0.050 | 0.113 | 1.000 | 739 | tags=38%, list=13%, signal=43% |
| 443 | CHANDRAN\_METASTASIS\_DN |  | 25 | -0.54 | -1.44 | 0.046 | 0.113 | 1.000 | 956 | tags=36%, list=17%, signal=43% |
| 444 | TOOKER\_GEMCITABINE\_RESISTANCE\_DN |  | 79 | -0.44 | -1.44 | 0.013 | 0.113 | 1.000 | 1943 | tags=47%, list=34%, signal=70% |
| 445 | BROWNE\_HCMV\_INFECTION\_48HR\_UP |  | 78 | -0.44 | -1.44 | 0.030 | 0.114 | 1.000 | 1231 | tags=31%, list=22%, signal=39% |
| 446 | HOLLMAN\_APOPTOSIS\_VIA\_CD40\_DN |  | 111 | -0.43 | -1.44 | 0.013 | 0.114 | 1.000 | 597 | tags=24%, list=10%, signal=27% |
| 447 | REACTOME\_CHEMOKINE\_RECEPTORS\_BIND\_CHEMOKINES |  | 16 | -0.58 | -1.44 | 0.055 | 0.116 | 1.000 | 833 | tags=50%, list=15%, signal=58% |
| 448 | GRAHAM\_CML\_QUIESCENT\_VS\_NORMAL\_QUIESCENT\_UP |  | 32 | -0.51 | -1.44 | 0.037 | 0.116 | 1.000 | 1232 | tags=38%, list=22%, signal=48% |
| 449 | MISSIAGLIA\_REGULATED\_BY\_METHYLATION\_UP |  | 33 | -0.50 | -1.44 | 0.044 | 0.116 | 1.000 | 962 | tags=39%, list=17%, signal=47% |
| 450 | GARGALOVIC\_RESPONSE\_TO\_OXIDIZED\_PHOSPHOLIPIDS\_BLUE\_DN |  | 19 | -0.55 | -1.44 | 0.057 | 0.118 | 1.000 | 414 | tags=26%, list=7%, signal=28% |
| 451 | LIN\_SILENCED\_BY\_TUMOR\_MICROENVIRONMENT |  | 20 | -0.55 | -1.43 | 0.068 | 0.120 | 1.000 | 1433 | tags=55%, list=25%, signal=73% |
| 452 | KEGG\_DILATED\_CARDIOMYOPATHY |  | 27 | -0.52 | -1.43 | 0.056 | 0.120 | 1.000 | 1416 | tags=52%, list=25%, signal=69% |
| 453 | CHARAFE\_BREAST\_CANCER\_BASAL\_VS\_MESENCHYMAL\_UP |  | 31 | -0.51 | -1.43 | 0.054 | 0.121 | 1.000 | 911 | tags=35%, list=16%, signal=42% |
| 454 | DEURIG\_T\_CELL\_PROLYMPHOCYTIC\_LEUKEMIA\_DN |  | 165 | -0.42 | -1.43 | 0.007 | 0.121 | 1.000 | 2056 | tags=52%, list=36%, signal=79% |
| 455 | KEGG\_AMINO\_SUGAR\_AND\_NUCLEOTIDE\_SUGAR\_METABOLISM |  | 23 | -0.54 | -1.43 | 0.046 | 0.123 | 1.000 | 812 | tags=43%, list=14%, signal=50% |
| 456 | BILBAN\_B\_CLL\_LPL\_UP |  | 29 | -0.51 | -1.43 | 0.055 | 0.123 | 1.000 | 292 | tags=17%, list=5%, signal=18% |
| 457 | BORCZUK\_MALIGNANT\_MESOTHELIOMA\_DN |  | 41 | -0.48 | -1.43 | 0.056 | 0.124 | 1.000 | 746 | tags=32%, list=13%, signal=36% |
| 458 | OSWALD\_HEMATOPOIETIC\_STEM\_CELL\_IN\_COLLAGEN\_GEL\_UP |  | 93 | -0.44 | -1.43 | 0.024 | 0.125 | 1.000 | 952 | tags=28%, list=17%, signal=33% |
| 459 | OSWALD\_HEMATOPOIETIC\_STEM\_CELL\_IN\_COLLAGEN\_GEL\_DN |  | 93 | -0.44 | -1.42 | 0.017 | 0.129 | 1.000 | 952 | tags=28%, list=17%, signal=33% |
| 460 | REACTOME\_CLASS\_A1\_RHODOPSIN\_LIKE\_RECEPTORS |  | 38 | -0.49 | -1.42 | 0.058 | 0.131 | 1.000 | 1137 | tags=50%, list=20%, signal=62% |
| 461 | BROCKE\_APOPTOSIS\_REVERSED\_BY\_IL6 |  | 74 | -0.44 | -1.42 | 0.023 | 0.131 | 1.000 | 1826 | tags=51%, list=32%, signal=74% |
| 462 | TIEN\_INTESTINE\_PROBIOTICS\_24HR\_DN |  | 106 | -0.43 | -1.42 | 0.017 | 0.133 | 1.000 | 1931 | tags=49%, list=34%, signal=73% |
| 463 | LIU\_SOX4\_TARGETS\_UP |  | 59 | -0.45 | -1.42 | 0.031 | 0.133 | 1.000 | 1243 | tags=36%, list=22%, signal=45% |
| 464 | ZHANG\_RESPONSE\_TO\_IKK\_INHIBITOR\_AND\_TNF\_UP |  | 79 | -0.43 | -1.42 | 0.030 | 0.133 | 1.000 | 1494 | tags=41%, list=26%, signal=54% |
| 465 | CREIGHTON\_ENDOCRINE\_THERAPY\_RESISTANCE\_3 |  | 285 | -0.41 | -1.42 | 0.000 | 0.133 | 1.000 | 1524 | tags=42%, list=27%, signal=54% |
| 466 | BAELDE\_DIABETIC\_NEPHROPATHY\_DN |  | 16 | -0.57 | -1.42 | 0.076 | 0.133 | 1.000 | 1196 | tags=50%, list=21%, signal=63% |
| 467 | TOOKER\_RESPONSE\_TO\_BEXAROTENE\_UP |  | 79 | -0.44 | -1.42 | 0.023 | 0.134 | 1.000 | 1943 | tags=47%, list=34%, signal=70% |
| 468 | BONOME\_OVARIAN\_CANCER\_SURVIVAL\_OPTIMAL\_DEBULKING |  | 87 | -0.43 | -1.41 | 0.023 | 0.136 | 1.000 | 1467 | tags=36%, list=26%, signal=47% |
| 469 | ROVERSI\_GLIOMA\_COPY\_NUMBER\_DN |  | 19 | -0.55 | -1.41 | 0.076 | 0.136 | 1.000 | 1287 | tags=42%, list=22%, signal=54% |
| 470 | NUTT\_GBM\_VS\_AO\_GLIOMA\_UP |  | 22 | -0.53 | -1.41 | 0.080 | 0.138 | 1.000 | 1588 | tags=45%, list=28%, signal=63% |
| 471 | HAMAI\_APOPTOSIS\_VIA\_TRAIL\_UP |  | 165 | -0.41 | -1.41 | 0.009 | 0.139 | 1.000 | 1898 | tags=45%, list=33%, signal=65% |
| 472 | JIANG\_HYPOXIA\_NORMAL |  | 107 | -0.42 | -1.41 | 0.023 | 0.140 | 1.000 | 1223 | tags=31%, list=21%, signal=38% |
| 473 | REACTOME\_G\_ALPHA\_I\_SIGNALLING\_EVENTS |  | 41 | -0.48 | -1.41 | 0.065 | 0.140 | 1.000 | 1137 | tags=44%, list=20%, signal=54% |
| 474 | SHAFFER\_IRF4\_TARGETS\_IN\_PLASMA\_CELL\_VS\_MATURE\_B\_LYMPHOCYTE |  | 26 | -0.51 | -1.41 | 0.062 | 0.140 | 1.000 | 1084 | tags=46%, list=19%, signal=57% |
| 475 | KIM\_MYCN\_AMPLIFICATION\_TARGETS\_DN |  | 40 | -0.48 | -1.41 | 0.058 | 0.141 | 1.000 | 1777 | tags=52%, list=31%, signal=76% |
| 476 | MILI\_PSEUDOPODIA\_CHEMOTAXIS\_DN |  | 216 | -0.40 | -1.41 | 0.004 | 0.141 | 1.000 | 1167 | tags=33%, list=20%, signal=40% |
| 477 | KIM\_WT1\_TARGETS\_UP |  | 92 | -0.43 | -1.41 | 0.021 | 0.141 | 1.000 | 869 | tags=27%, list=15%, signal=32% |
| 478 | FULCHER\_INFLAMMATORY\_RESPONSE\_LECTIN\_VS\_LPS\_UP |  | 228 | -0.40 | -1.41 | 0.003 | 0.142 | 1.000 | 953 | tags=26%, list=17%, signal=30% |
| 479 | REACTOME\_PLATELET\_ACTIVATION\_TRIGGERS |  | 33 | -0.48 | -1.41 | 0.068 | 0.142 | 1.000 | 1083 | tags=33%, list=19%, signal=41% |
| 480 | SWEET\_LUNG\_CANCER\_KRAS\_UP |  | 207 | -0.40 | -1.40 | 0.004 | 0.146 | 1.000 | 1466 | tags=37%, list=26%, signal=48% |
| 481 | DOANE\_RESPONSE\_TO\_ANDROGEN\_DN |  | 89 | -0.43 | -1.40 | 0.027 | 0.146 | 1.000 | 658 | tags=25%, list=11%, signal=27% |
| 482 | JI\_RESPONSE\_TO\_FSH\_UP |  | 29 | -0.50 | -1.40 | 0.057 | 0.147 | 1.000 | 856 | tags=38%, list=15%, signal=44% |
| 483 | PROVENZANI\_METASTASIS\_UP |  | 90 | -0.43 | -1.40 | 0.028 | 0.147 | 1.000 | 964 | tags=29%, list=17%, signal=34% |
| 484 | FARMER\_BREAST\_CANCER\_BASAL\_VS\_LULMINAL |  | 120 | -0.42 | -1.40 | 0.014 | 0.150 | 1.000 | 1487 | tags=40%, list=26%, signal=53% |
| 485 | VANTVEER\_BREAST\_CANCER\_POOR\_PROGNOSIS |  | 20 | -0.53 | -1.40 | 0.092 | 0.150 | 1.000 | 579 | tags=25%, list=10%, signal=28% |
| 486 | ODONNELL\_TARGETS\_OF\_MYC\_AND\_TFRC\_UP |  | 32 | -0.49 | -1.40 | 0.066 | 0.151 | 1.000 | 1346 | tags=44%, list=24%, signal=57% |
| 487 | REACTOME\_DOWNSTREAM\_EVENTS\_IN\_GPCR\_SIGNALING |  | 96 | -0.43 | -1.40 | 0.019 | 0.152 | 1.000 | 1070 | tags=31%, list=19%, signal=38% |
| 488 | SEKI\_INFLAMMATORY\_RESPONSE\_LPS\_UP |  | 25 | -0.51 | -1.40 | 0.084 | 0.152 | 1.000 | 1100 | tags=44%, list=19%, signal=54% |
| 489 | SEIDEN\_ONCOGENESIS\_BY\_MET |  | 52 | -0.45 | -1.39 | 0.058 | 0.155 | 1.000 | 2378 | tags=63%, list=42%, signal=108% |
| 490 | YAMASHITA\_LIVER\_CANCER\_STEM\_CELL\_DN |  | 19 | -0.54 | -1.39 | 0.081 | 0.155 | 1.000 | 890 | tags=42%, list=16%, signal=50% |
| 491 | WENDT\_COHESIN\_TARGETS\_UP |  | 18 | -0.54 | -1.39 | 0.086 | 0.155 | 1.000 | 1758 | tags=50%, list=31%, signal=72% |
| 492 | FERRANDO\_T\_ALL\_WITH\_MLL\_ENL\_FUSION\_UP |  | 35 | -0.47 | -1.39 | 0.064 | 0.158 | 1.000 | 1218 | tags=43%, list=21%, signal=54% |
| 493 | RIGGINS\_TAMOXIFEN\_RESISTANCE\_DN |  | 96 | -0.42 | -1.39 | 0.025 | 0.158 | 1.000 | 863 | tags=27%, list=15%, signal=31% |
| 494 | MORI\_SMALL\_PRE\_BII\_LYMPHOCYTE\_DN |  | 34 | -0.48 | -1.39 | 0.072 | 0.159 | 1.000 | 195 | tags=12%, list=3%, signal=12% |
| 495 | HUTTMANN\_B\_CLL\_POOR\_SURVIVAL\_UP |  | 95 | -0.42 | -1.39 | 0.027 | 0.161 | 1.000 | 622 | tags=21%, list=11%, signal=23% |
| 496 | MOLENAAR\_TARGETS\_OF\_CCND1\_AND\_CDK4\_UP |  | 25 | -0.50 | -1.39 | 0.086 | 0.161 | 1.000 | 1525 | tags=52%, list=27%, signal=71% |
| 497 | MARCHINI\_TRABECTEDIN\_RESISTANCE\_DN |  | 20 | -0.53 | -1.39 | 0.076 | 0.161 | 1.000 | 551 | tags=30%, list=10%, signal=33% |
| 498 | GAZDA\_DIAMOND\_BLACKFAN\_ANEMIA\_PROGENITOR\_UP |  | 15 | -0.57 | -1.39 | 0.083 | 0.161 | 1.000 | 903 | tags=40%, list=16%, signal=47% |
| 499 | KLEIN\_PRIMARY\_EFFUSION\_LYMPHOMA\_UP |  | 22 | -0.52 | -1.38 | 0.089 | 0.161 | 1.000 | 1766 | tags=55%, list=31%, signal=79% |
| 500 | ZHAN\_MULTIPLE\_MYELOMA\_HP\_DN |  | 22 | -0.52 | -1.38 | 0.071 | 0.162 | 1.000 | 1348 | tags=50%, list=24%, signal=65% |
| 501 | RUGO\_STRESS\_RESPONSE\_SUBSET\_G |  | 17 | -0.55 | -1.38 | 0.090 | 0.163 | 1.000 | 1324 | tags=53%, list=23%, signal=69% |
| 502 | HAHTOLA\_MYCOSIS\_FUNGOIDES\_CD4\_UP |  | 22 | -0.52 | -1.38 | 0.088 | 0.163 | 1.000 | 744 | tags=32%, list=13%, signal=36% |
| 503 | HAHTOLA\_SEZARY\_SYNDROM\_UP |  | 48 | -0.46 | -1.38 | 0.051 | 0.164 | 1.000 | 1424 | tags=31%, list=25%, signal=41% |
| 504 | BOYLAN\_MULTIPLE\_MYELOMA\_C\_DN |  | 19 | -0.53 | -1.38 | 0.079 | 0.164 | 1.000 | 923 | tags=32%, list=16%, signal=38% |
| 505 | NAKAYAMA\_FRA2\_TARGETS |  | 18 | -0.54 | -1.38 | 0.086 | 0.166 | 1.000 | 684 | tags=33%, list=12%, signal=38% |
| 506 | KYNG\_DNA\_DAMAGE\_BY\_4NQO\_OR\_UV |  | 17 | -0.55 | -1.38 | 0.109 | 0.167 | 1.000 | 1324 | tags=53%, list=23%, signal=69% |
| 507 | CROONQUIST\_NRAS\_VS\_STROMAL\_STIMULATION\_DN |  | 35 | -0.47 | -1.38 | 0.078 | 0.167 | 1.000 | 795 | tags=31%, list=14%, signal=36% |
| 508 | MILI\_PSEUDOPODIA\_HAPTOTAXIS\_DN |  | 276 | -0.39 | -1.38 | 0.002 | 0.167 | 1.000 | 1606 | tags=41%, list=28%, signal=55% |
| 509 | KEGG\_PROPANOATE\_METABOLISM |  | 20 | -0.52 | -1.38 | 0.104 | 0.167 | 1.000 | 1805 | tags=55%, list=32%, signal=80% |
| 510 | BRUECKNER\_TARGETS\_OF\_MIRLET7A3\_UP |  | 45 | -0.46 | -1.38 | 0.070 | 0.170 | 1.000 | 1449 | tags=36%, list=25%, signal=47% |
| 511 | ZHONG\_RESPONSE\_TO\_AZACITIDINE\_AND\_TSA\_UP |  | 67 | -0.43 | -1.37 | 0.044 | 0.170 | 1.000 | 744 | tags=21%, list=13%, signal=24% |
| 512 | KEGG\_GAP\_JUNCTION |  | 35 | -0.47 | -1.37 | 0.076 | 0.170 | 1.000 | 1039 | tags=31%, list=18%, signal=38% |
| 513 | EHLERS\_ANEUPLOIDY\_UP |  | 17 | -0.55 | -1.37 | 0.091 | 0.170 | 1.000 | 470 | tags=24%, list=8%, signal=26% |
| 514 | TAKEDA\_TARGETS\_OF\_NUP98\_HOXA9\_FUSION\_16D\_UP |  | 52 | -0.44 | -1.37 | 0.048 | 0.170 | 1.000 | 884 | tags=35%, list=15%, signal=41% |
| 515 | WILCOX\_PRESPONSE\_TO\_ROGESTERONE\_UP |  | 62 | -0.44 | -1.37 | 0.058 | 0.172 | 1.000 | 991 | tags=29%, list=17%, signal=35% |
| 516 | RICKMAN\_TUMOR\_DIFFERENTIATED\_WELL\_VS\_MODERATELY\_DN |  | 27 | -0.49 | -1.37 | 0.099 | 0.174 | 1.000 | 2362 | tags=67%, list=41%, signal=113% |
| 517 | MORI\_MATURE\_B\_LYMPHOCYTE\_DN |  | 26 | -0.50 | -1.37 | 0.093 | 0.178 | 1.000 | 482 | tags=23%, list=8%, signal=25% |
| 518 | NING\_CHRONIC\_OBSTRUCTIVE\_PULMONARY\_DISEASE\_DN |  | 30 | -0.48 | -1.36 | 0.081 | 0.181 | 1.000 | 1269 | tags=47%, list=22%, signal=60% |
| 519 | BARRIER\_CANCER\_RELAPSE\_NORMAL\_SAMPLE\_UP |  | 16 | -0.54 | -1.36 | 0.098 | 0.181 | 1.000 | 1452 | tags=44%, list=25%, signal=58% |
| 520 | OUILLETTE\_CLL\_13Q14\_DELETION\_DN |  | 21 | -0.51 | -1.36 | 0.096 | 0.184 | 1.000 | 525 | tags=29%, list=9%, signal=31% |
| 521 | SESTO\_RESPONSE\_TO\_UV\_C8 |  | 36 | -0.47 | -1.36 | 0.085 | 0.184 | 1.000 | 1162 | tags=39%, list=20%, signal=48% |
| 522 | LIANG\_HEMATOPOIESIS\_STEM\_CELL\_NUMBER\_LARGE\_VS\_TINY\_UP |  | 16 | -0.55 | -1.36 | 0.105 | 0.184 | 1.000 | 542 | tags=25%, list=9%, signal=28% |
| 523 | BIOCARTA\_EDG1\_PATHWAY |  | 15 | -0.55 | -1.36 | 0.111 | 0.184 | 1.000 | 1103 | tags=40%, list=19%, signal=49% |
| 524 | THEILGAARD\_NEUTROPHIL\_AT\_SKIN\_WOUND\_UP |  | 38 | -0.46 | -1.36 | 0.083 | 0.185 | 1.000 | 1152 | tags=39%, list=20%, signal=49% |
| 525 | VECCHI\_GASTRIC\_CANCER\_ADVANCED\_VS\_EARLY\_DN |  | 32 | -0.47 | -1.36 | 0.099 | 0.186 | 1.000 | 872 | tags=28%, list=15%, signal=33% |
| 526 | MORI\_SMALL\_PRE\_BII\_LYMPHOCYTE\_UP |  | 32 | -0.48 | -1.36 | 0.097 | 0.186 | 1.000 | 965 | tags=25%, list=17%, signal=30% |
| 527 | ST\_T\_CELL\_SIGNAL\_TRANSDUCTION |  | 25 | -0.50 | -1.36 | 0.104 | 0.187 | 1.000 | 1083 | tags=32%, list=19%, signal=39% |
| 528 | SESTO\_RESPONSE\_TO\_UV\_C6 |  | 21 | -0.52 | -1.36 | 0.105 | 0.187 | 1.000 | 636 | tags=33%, list=11%, signal=37% |
| 529 | AMIT\_SERUM\_RESPONSE\_120\_MCF10A |  | 25 | -0.50 | -1.36 | 0.107 | 0.188 | 1.000 | 739 | tags=32%, list=13%, signal=37% |
| 530 | BARIS\_THYROID\_CANCER\_DN |  | 27 | -0.49 | -1.36 | 0.116 | 0.188 | 1.000 | 1424 | tags=44%, list=25%, signal=59% |
| 531 | KEGG\_VASCULAR\_SMOOTH\_MUSCLE\_CONTRACTION |  | 46 | -0.45 | -1.36 | 0.071 | 0.188 | 1.000 | 611 | tags=28%, list=11%, signal=31% |
| 532 | LEE\_LIVER\_CANCER\_E2F1\_DN |  | 21 | -0.51 | -1.35 | 0.126 | 0.190 | 1.000 | 693 | tags=33%, list=12%, signal=38% |
| 533 | CUI\_GLUCOSE\_DEPRIVATION |  | 27 | -0.48 | -1.35 | 0.103 | 0.193 | 1.000 | 1436 | tags=41%, list=25%, signal=54% |
| 534 | GAUSSMANN\_MLL\_AF4\_FUSION\_TARGETS\_G\_UP |  | 51 | -0.44 | -1.35 | 0.074 | 0.193 | 1.000 | 1324 | tags=35%, list=23%, signal=46% |
| 535 | KEGG\_SPHINGOLIPID\_METABOLISM |  | 16 | -0.54 | -1.35 | 0.103 | 0.193 | 1.000 | 1429 | tags=50%, list=25%, signal=66% |
| 536 | KEGG\_JAK\_STAT\_SIGNALING\_PATHWAY |  | 52 | -0.44 | -1.35 | 0.073 | 0.194 | 1.000 | 990 | tags=27%, list=17%, signal=32% |
| 537 | AMIT\_EGF\_RESPONSE\_240\_HELA |  | 28 | -0.48 | -1.35 | 0.094 | 0.194 | 1.000 | 1001 | tags=32%, list=17%, signal=39% |
| 538 | RIGGINS\_TAMOXIFEN\_RESISTANCE\_UP |  | 19 | -0.53 | -1.35 | 0.113 | 0.194 | 1.000 | 1531 | tags=47%, list=27%, signal=64% |
| 539 | BEIER\_GLIOMA\_STEM\_CELL\_DN |  | 32 | -0.47 | -1.35 | 0.100 | 0.194 | 1.000 | 1035 | tags=34%, list=18%, signal=42% |
| 540 | PUIFFE\_INVASION\_INHIBITED\_BY\_ASCITES\_DN |  | 74 | -0.42 | -1.35 | 0.069 | 0.195 | 1.000 | 1923 | tags=49%, list=34%, signal=72% |
| 541 | GINESTIER\_BREAST\_CANCER\_ZNF217\_AMPLIFIED\_UP |  | 38 | -0.46 | -1.35 | 0.083 | 0.197 | 1.000 | 2379 | tags=63%, list=42%, signal=107% |
| 542 | LEE\_LIVER\_CANCER\_ACOX1\_DN |  | 17 | -0.54 | -1.35 | 0.122 | 0.197 | 1.000 | 590 | tags=35%, list=10%, signal=39% |
| 543 | KEGG\_REGULATION\_OF\_ACTIN\_CYTOSKELETON |  | 87 | -0.42 | -1.35 | 0.051 | 0.197 | 1.000 | 770 | tags=22%, list=13%, signal=25% |
| 544 | ROSS\_ACUTE\_MYELOID\_LEUKEMIA\_CBF |  | 24 | -0.49 | -1.35 | 0.127 | 0.197 | 1.000 | 535 | tags=33%, list=9%, signal=37% |
| 545 | REACTOME\_CD28\_CO\_STIMULATION |  | 21 | -0.51 | -1.35 | 0.111 | 0.198 | 1.000 | 1019 | tags=38%, list=18%, signal=46% |
| 546 | BILD\_E2F3\_ONCOGENIC\_SIGNATURE |  | 79 | -0.42 | -1.34 | 0.058 | 0.198 | 1.000 | 964 | tags=28%, list=17%, signal=33% |
| 547 | TIEN\_INTESTINE\_PROBIOTICS\_6HR\_DN |  | 87 | -0.41 | -1.34 | 0.045 | 0.198 | 1.000 | 1698 | tags=44%, list=30%, signal=61% |
| 548 | BIOCARTA\_FCER1\_PATHWAY |  | 27 | -0.48 | -1.34 | 0.107 | 0.200 | 1.000 | 582 | tags=19%, list=10%, signal=21% |
| 549 | VALK\_AML\_CLUSTER\_6 |  | 15 | -0.55 | -1.34 | 0.118 | 0.199 | 1.000 | 273 | tags=27%, list=5%, signal=28% |
| 550 | REACTOME\_REGULATION\_OF\_BETA\_CELL\_DEVELOPMENT |  | 19 | -0.51 | -1.34 | 0.115 | 0.199 | 1.000 | 108 | tags=5%, list=2%, signal=5% |
| 551 | RICKMAN\_TUMOR\_DIFFERENTIATED\_WELL\_VS\_POORLY\_DN |  | 133 | -0.40 | -1.34 | 0.031 | 0.200 | 1.000 | 1015 | tags=26%, list=18%, signal=30% |
| 552 | TONKS\_TARGETS\_OF\_RUNX1\_RUNX1T1\_FUSION\_MONOCYTE\_DN |  | 29 | -0.48 | -1.34 | 0.125 | 0.201 | 1.000 | 1045 | tags=34%, list=18%, signal=42% |
| 553 | YAO\_TEMPORAL\_RESPONSE\_TO\_PROGESTERONE\_CLUSTER\_6 |  | 28 | -0.48 | -1.34 | 0.105 | 0.203 | 1.000 | 1277 | tags=43%, list=22%, signal=55% |
| 554 | PRAMOONJAGO\_SOX4\_TARGETS\_DN |  | 31 | -0.47 | -1.34 | 0.102 | 0.203 | 1.000 | 681 | tags=19%, list=12%, signal=22% |
| 555 | SMITH\_TERT\_TARGETS\_UP |  | 72 | -0.41 | -1.34 | 0.077 | 0.205 | 1.000 | 1335 | tags=29%, list=23%, signal=38% |
| 556 | MARTORIATI\_MDM4\_TARGETS\_FETAL\_LIVER\_DN |  | 127 | -0.40 | -1.34 | 0.038 | 0.205 | 1.000 | 1100 | tags=24%, list=19%, signal=30% |
| 557 | YAO\_HOXA10\_TARGETS\_VIA\_PROGESTERONE\_UP |  | 21 | -0.51 | -1.34 | 0.117 | 0.206 | 1.000 | 1030 | tags=43%, list=18%, signal=52% |
| 558 | YAO\_TEMPORAL\_RESPONSE\_TO\_PROGESTERONE\_CLUSTER\_10 |  | 35 | -0.46 | -1.33 | 0.110 | 0.209 | 1.000 | 1745 | tags=51%, list=30%, signal=74% |
| 559 | PEREZ\_TP53\_TARGETS |  | 269 | -0.38 | -1.33 | 0.007 | 0.211 | 1.000 | 1006 | tags=26%, list=18%, signal=30% |
| 560 | ACEVEDO\_LIVER\_TUMOR\_VS\_NORMAL\_ADJACENT\_TISSUE\_DN |  | 86 | -0.40 | -1.33 | 0.058 | 0.214 | 1.000 | 990 | tags=30%, list=17%, signal=36% |
| 561 | GOLDRATH\_ANTIGEN\_RESPONSE |  | 170 | -0.39 | -1.33 | 0.032 | 0.219 | 1.000 | 842 | tags=23%, list=15%, signal=26% |
| 562 | SHEN\_SMARCA2\_TARGETS\_UP |  | 284 | -0.38 | -1.33 | 0.011 | 0.222 | 1.000 | 2621 | tags=61%, list=46%, signal=107% |
| 563 | NAKAMURA\_TUMOR\_ZONE\_PERIPHERAL\_VS\_CENTRAL\_UP |  | 118 | -0.40 | -1.32 | 0.045 | 0.223 | 1.000 | 642 | tags=19%, list=11%, signal=21% |
| 564 | RASHI\_RESPONSE\_TO\_IONIZING\_RADIATION\_2 |  | 52 | -0.42 | -1.32 | 0.101 | 0.223 | 1.000 | 1534 | tags=35%, list=27%, signal=47% |
| 565 | MASSARWEH\_TAMOXIFEN\_RESISTANCE\_DN |  | 66 | -0.42 | -1.32 | 0.076 | 0.225 | 1.000 | 1585 | tags=45%, list=28%, signal=62% |
| 566 | REACTOME\_TCR\_SIGNALING |  | 34 | -0.46 | -1.32 | 0.110 | 0.224 | 1.000 | 1202 | tags=38%, list=21%, signal=48% |
| 567 | GUENTHER\_GROWTH\_SPHERICAL\_VS\_ADHERENT\_DN |  | 15 | -0.54 | -1.32 | 0.138 | 0.225 | 1.000 | 1169 | tags=60%, list=20%, signal=75% |
| 568 | WEIGEL\_OXIDATIVE\_STRESS\_BY\_TBH\_AND\_H2O2 |  | 19 | -0.51 | -1.32 | 0.131 | 0.225 | 1.000 | 639 | tags=32%, list=11%, signal=35% |
| 569 | WANG\_CISPLATIN\_RESPONSE\_AND\_XPC\_DN |  | 62 | -0.42 | -1.32 | 0.074 | 0.225 | 1.000 | 1771 | tags=42%, list=31%, signal=60% |
| 570 | REACTOME\_THE\_ROLE\_OF\_NEF\_IN\_HIV1\_REPLICATION\_AND\_DISEASE\_PATHOGENESIS |  | 19 | -0.52 | -1.32 | 0.120 | 0.225 | 1.000 | 1202 | tags=42%, list=21%, signal=53% |
| 571 | AMUNDSON\_POOR\_SURVIVAL\_AFTER\_GAMMA\_RADIATION\_2G |  | 67 | -0.42 | -1.32 | 0.074 | 0.227 | 1.000 | 1262 | tags=34%, list=22%, signal=44% |
| 572 | BROWNE\_HCMV\_INFECTION\_1HR\_UP |  | 18 | -0.52 | -1.32 | 0.138 | 0.228 | 1.000 | 559 | tags=28%, list=10%, signal=31% |
| 573 | RHEIN\_ALL\_GLUCOCORTICOID\_THERAPY\_UP |  | 44 | -0.44 | -1.32 | 0.086 | 0.228 | 1.000 | 1190 | tags=34%, list=21%, signal=43% |
| 574 | KEGG\_GLYCEROPHOSPHOLIPID\_METABOLISM |  | 31 | -0.46 | -1.32 | 0.136 | 0.228 | 1.000 | 1323 | tags=35%, list=23%, signal=46% |
| 575 | LIANG\_HEMATOPOIESIS\_STEM\_CELL\_NUMBER\_SMALL\_VS\_HUGE\_DN |  | 17 | -0.53 | -1.32 | 0.157 | 0.229 | 1.000 | 979 | tags=41%, list=17%, signal=50% |
| 576 | COLDREN\_GEFITINIB\_RESISTANCE\_DN |  | 61 | -0.42 | -1.32 | 0.086 | 0.230 | 1.000 | 1492 | tags=44%, list=26%, signal=59% |
| 577 | GARGALOVIC\_RESPONSE\_TO\_OXIDIZED\_PHOSPHOLIPIDS\_GREY\_DN |  | 23 | -0.49 | -1.32 | 0.140 | 0.230 | 1.000 | 1384 | tags=48%, list=24%, signal=63% |
| 578 | AMIT\_EGF\_RESPONSE\_60\_MCF10A |  | 15 | -0.54 | -1.31 | 0.132 | 0.231 | 1.000 | 1433 | tags=47%, list=25%, signal=62% |
| 579 | REACTOME\_G\_ALPHA\_S\_SIGNALLING\_EVENTS |  | 24 | -0.48 | -1.31 | 0.128 | 0.231 | 1.000 | 536 | tags=21%, list=9%, signal=23% |
| 580 | CREIGHTON\_ENDOCRINE\_THERAPY\_RESISTANCE\_5 |  | 199 | -0.38 | -1.31 | 0.019 | 0.235 | 1.000 | 1522 | tags=41%, list=27%, signal=54% |
| 581 | RUTELLA\_RESPONSE\_TO\_HGF\_DN |  | 107 | -0.40 | -1.31 | 0.066 | 0.236 | 1.000 | 1072 | tags=29%, list=19%, signal=35% |
| 582 | LEE\_LIVER\_CANCER\_MYC\_TGFA\_DN |  | 20 | -0.51 | -1.31 | 0.165 | 0.236 | 1.000 | 767 | tags=35%, list=13%, signal=40% |
| 583 | KEGG\_OXIDATIVE\_PHOSPHORYLATION |  | 56 | -0.42 | -1.31 | 0.084 | 0.236 | 1.000 | 2492 | tags=64%, list=44%, signal=113% |
| 584 | TURASHVILI\_BREAST\_DUCTAL\_CARCINOMA\_VS\_LOBULAR\_NORMAL\_UP |  | 41 | -0.44 | -1.31 | 0.102 | 0.236 | 1.000 | 1649 | tags=41%, list=29%, signal=58% |
| 585 | REACTOME\_APOPTOTIC\_EXECUTION\_PHASE |  | 22 | -0.49 | -1.31 | 0.138 | 0.236 | 1.000 | 802 | tags=32%, list=14%, signal=37% |
| 586 | LEE\_LIVER\_CANCER\_SURVIVAL\_DN |  | 56 | -0.42 | -1.31 | 0.105 | 0.237 | 1.000 | 817 | tags=18%, list=14%, signal=21% |
| 587 | REACTOME\_AMINO\_ACID\_AND\_OLIGOPEPTIDE\_SLC\_TRANSPORTERS |  | 15 | -0.53 | -1.31 | 0.153 | 0.242 | 1.000 | 520 | tags=27%, list=9%, signal=29% |
| 588 | FONTAINE\_FOLLICULAR\_THYROID\_ADENOMA\_DN |  | 24 | -0.48 | -1.30 | 0.109 | 0.246 | 1.000 | 693 | tags=21%, list=12%, signal=24% |
| 589 | CHEN\_NEUROBLASTOMA\_COPY\_NUMBER\_GAINS |  | 19 | -0.51 | -1.30 | 0.136 | 0.248 | 1.000 | 1183 | tags=32%, list=21%, signal=40% |
| 590 | NATSUME\_RESPONSE\_TO\_INTERFERON\_BETA\_DN |  | 21 | -0.49 | -1.30 | 0.155 | 0.250 | 1.000 | 1309 | tags=38%, list=23%, signal=49% |
| 591 | ACEVEDO\_LIVER\_CANCER\_UP |  | 486 | -0.36 | -1.30 | 0.003 | 0.250 | 1.000 | 1996 | tags=44%, list=35%, signal=62% |
| 592 | KEGG\_PATHWAYS\_IN\_CANCER |  | 135 | -0.38 | -1.30 | 0.045 | 0.252 | 1.000 | 1324 | tags=27%, list=23%, signal=35% |
| 593 | AMIT\_EGF\_RESPONSE\_120\_HELA |  | 24 | -0.47 | -1.30 | 0.142 | 0.252 | 1.000 | 1199 | tags=33%, list=21%, signal=42% |
| 594 | RODRIGUES\_THYROID\_CARCINOMA\_POORLY\_DIFFERENTIATED\_DN |  | 317 | -0.37 | -1.30 | 0.011 | 0.254 | 1.000 | 1297 | tags=30%, list=23%, signal=36% |
| 595 | NIKOLSKY\_BREAST\_CANCER\_7Q21\_Q22\_AMPLICON |  | 25 | -0.48 | -1.30 | 0.153 | 0.254 | 1.000 | 1079 | tags=40%, list=19%, signal=49% |
| 596 | LEE\_DIFFERENTIATING\_T\_LYMPHOCYTE |  | 67 | -0.41 | -1.30 | 0.107 | 0.255 | 1.000 | 912 | tags=25%, list=16%, signal=30% |
| 597 | RAMASWAMY\_METASTASIS\_DN |  | 18 | -0.50 | -1.30 | 0.162 | 0.255 | 1.000 | 563 | tags=22%, list=10%, signal=25% |
| 598 | REACTOME\_GENES\_INVOLVED\_IN\_APOPTOTIC\_CLEAVAGE\_OF\_CELLULAR\_PROTEINS |  | 17 | -0.51 | -1.29 | 0.160 | 0.256 | 1.000 | 770 | tags=35%, list=13%, signal=41% |
| 599 | FOSTER\_INFLAMMATORY\_RESPONSE\_LPS\_DN |  | 168 | -0.38 | -1.29 | 0.040 | 0.258 | 1.000 | 1125 | tags=29%, list=20%, signal=35% |
| 600 | WAMUNYOKOLI\_OVARIAN\_CANCER\_GRADES\_1\_2\_UP |  | 51 | -0.41 | -1.29 | 0.115 | 0.259 | 1.000 | 1943 | tags=49%, list=34%, signal=74% |
| 601 | COATES\_MACROPHAGE\_M1\_VS\_M2\_UP |  | 36 | -0.45 | -1.29 | 0.124 | 0.260 | 1.000 | 659 | tags=28%, list=12%, signal=31% |
| 602 | LUI\_TARGETS\_OF\_PAX8\_PPARG\_FUSION |  | 16 | -0.53 | -1.29 | 0.169 | 0.261 | 1.000 | 1181 | tags=31%, list=21%, signal=39% |
| 603 | KRIGE\_AMINO\_ACID\_DEPRIVATION |  | 15 | -0.52 | -1.29 | 0.181 | 0.260 | 1.000 | 885 | tags=33%, list=15%, signal=39% |
| 604 | CROONQUIST\_NRAS\_SIGNALING\_UP |  | 15 | -0.54 | -1.29 | 0.166 | 0.261 | 1.000 | 555 | tags=33%, list=10%, signal=37% |
| 605 | YAO\_TEMPORAL\_RESPONSE\_TO\_PROGESTERONE\_CLUSTER\_1 |  | 29 | -0.46 | -1.29 | 0.144 | 0.264 | 1.000 | 971 | tags=34%, list=17%, signal=41% |
| 606 | SENESE\_HDAC1\_TARGETS\_UP |  | 220 | -0.37 | -1.29 | 0.032 | 0.267 | 1.000 | 1190 | tags=25%, list=21%, signal=30% |
| 607 | BIOCARTA\_PPARA\_PATHWAY |  | 30 | -0.46 | -1.28 | 0.147 | 0.270 | 1.000 | 479 | tags=13%, list=8%, signal=14% |
| 608 | MARTINEZ\_RB1\_TARGETS\_UP |  | 242 | -0.36 | -1.28 | 0.028 | 0.271 | 1.000 | 1530 | tags=36%, list=27%, signal=47% |
| 609 | KIM\_WT1\_TARGETS\_12HR\_UP |  | 53 | -0.42 | -1.28 | 0.122 | 0.271 | 1.000 | 835 | tags=28%, list=15%, signal=33% |
| 610 | RICKMAN\_METASTASIS\_UP |  | 140 | -0.38 | -1.28 | 0.064 | 0.271 | 1.000 | 1683 | tags=39%, list=29%, signal=54% |
| 611 | KEGG\_ARGININE\_AND\_PROLINE\_METABOLISM |  | 18 | -0.49 | -1.28 | 0.163 | 0.273 | 1.000 | 1773 | tags=56%, list=31%, signal=80% |
| 612 | REACTOME\_ELECTRON\_TRANSPORT\_CHAIN |  | 41 | -0.43 | -1.28 | 0.140 | 0.274 | 1.000 | 2492 | tags=59%, list=44%, signal=103% |
| 613 | DUNNE\_TARGETS\_OF\_AML1\_MTG8\_FUSION\_UP |  | 21 | -0.48 | -1.28 | 0.162 | 0.274 | 1.000 | 1388 | tags=38%, list=24%, signal=50% |
| 614 | MARSON\_FOXP3\_TARGETS\_UP |  | 31 | -0.45 | -1.28 | 0.165 | 0.277 | 1.000 | 1535 | tags=42%, list=27%, signal=57% |
| 615 | MATSUDA\_NATURAL\_KILLER\_DIFFERENTIATION |  | 191 | -0.37 | -1.28 | 0.044 | 0.277 | 1.000 | 1194 | tags=29%, list=21%, signal=35% |
| 616 | LIEN\_BREAST\_CARCINOMA\_METAPLASTIC\_VS\_DUCTAL\_DN |  | 22 | -0.48 | -1.28 | 0.157 | 0.278 | 1.000 | 746 | tags=41%, list=13%, signal=47% |
| 617 | KIM\_WT1\_TARGETS\_12HR\_DN |  | 84 | -0.39 | -1.28 | 0.097 | 0.279 | 1.000 | 845 | tags=23%, list=15%, signal=26% |
| 618 | HOLLMAN\_APOPTOSIS\_VIA\_CD40\_UP |  | 96 | -0.38 | -1.28 | 0.080 | 0.280 | 1.000 | 1402 | tags=34%, list=24%, signal=45% |
| 619 | KEGG\_PATHOGENIC\_ESCHERICHIA\_COLI\_INFECTION |  | 19 | -0.49 | -1.28 | 0.171 | 0.279 | 1.000 | 1163 | tags=37%, list=20%, signal=46% |
| 620 | CHIANG\_LIVER\_CANCER\_SUBCLASS\_UNANNOTATED\_DN |  | 88 | -0.39 | -1.28 | 0.101 | 0.279 | 1.000 | 1412 | tags=31%, list=25%, signal=40% |
| 621 | RAMALHO\_STEMNESS\_UP |  | 124 | -0.38 | -1.27 | 0.075 | 0.282 | 1.000 | 1510 | tags=35%, list=26%, signal=46% |
| 622 | GRABARCZYK\_BCL11B\_TARGETS\_UP |  | 41 | -0.41 | -1.27 | 0.158 | 0.283 | 1.000 | 2112 | tags=49%, list=37%, signal=77% |
| 623 | MILI\_PSEUDOPODIA\_HAPTOTAXIS\_UP |  | 256 | -0.36 | -1.27 | 0.034 | 0.283 | 1.000 | 2638 | tags=64%, list=46%, signal=113% |
| 624 | HELLER\_HDAC\_TARGETS\_DN |  | 103 | -0.38 | -1.27 | 0.094 | 0.286 | 1.000 | 928 | tags=26%, list=16%, signal=31% |
| 625 | MULLIGHAN\_MLL\_SIGNATURE\_1\_DN |  | 79 | -0.39 | -1.27 | 0.104 | 0.286 | 1.000 | 1585 | tags=42%, list=28%, signal=57% |
| 626 | ENK\_UV\_RESPONSE\_EPIDERMIS\_DN |  | 228 | -0.37 | -1.27 | 0.026 | 0.289 | 1.000 | 1534 | tags=33%, list=27%, signal=43% |
| 627 | SENESE\_HDAC3\_TARGETS\_DN |  | 184 | -0.37 | -1.27 | 0.053 | 0.289 | 1.000 | 844 | tags=23%, list=15%, signal=26% |
| 628 | SANA\_TNF\_SIGNALING\_UP |  | 26 | -0.46 | -1.27 | 0.175 | 0.289 | 1.000 | 1396 | tags=42%, list=24%, signal=56% |
| 629 | MCBRYAN\_PUBERTAL\_BREAST\_4\_5WK\_DN |  | 78 | -0.40 | -1.27 | 0.120 | 0.292 | 1.000 | 818 | tags=22%, list=14%, signal=25% |
| 630 | KEGG\_VEGF\_SIGNALING\_PATHWAY |  | 30 | -0.45 | -1.27 | 0.160 | 0.292 | 1.000 | 437 | tags=17%, list=8%, signal=18% |
| 631 | KEGG\_HYPERTROPHIC\_CARDIOMYOPATHY\_HCM |  | 28 | -0.45 | -1.27 | 0.153 | 0.292 | 1.000 | 1416 | tags=46%, list=25%, signal=61% |
| 632 | SHAFFER\_IRF4\_TARGETS\_IN\_MYELOMA\_VS\_MATURE\_B\_LYMPHOCYTE |  | 50 | -0.41 | -1.27 | 0.128 | 0.292 | 1.000 | 1093 | tags=34%, list=19%, signal=42% |
| 633 | SHEDDEN\_LUNG\_CANCER\_GOOD\_SURVIVAL\_A12 |  | 84 | -0.39 | -1.26 | 0.091 | 0.293 | 1.000 | 989 | tags=30%, list=17%, signal=35% |
| 634 | KEGG\_CHEMOKINE\_SIGNALING\_PATHWAY |  | 89 | -0.38 | -1.26 | 0.105 | 0.294 | 1.000 | 1080 | tags=25%, list=19%, signal=30% |
| 635 | LINDGREN\_BLADDER\_CANCER\_CLUSTER\_1\_DN |  | 167 | -0.37 | -1.26 | 0.059 | 0.295 | 1.000 | 964 | tags=24%, list=17%, signal=28% |
| 636 | BLUM\_RESPONSE\_TO\_SALIRASIB\_UP |  | 135 | -0.37 | -1.26 | 0.069 | 0.299 | 1.000 | 1084 | tags=27%, list=19%, signal=33% |
| 637 | ASTON\_MAJOR\_DEPRESSIVE\_DISORDER\_DN |  | 54 | -0.41 | -1.26 | 0.136 | 0.300 | 1.000 | 1109 | tags=35%, list=19%, signal=43% |
| 638 | TAKAO\_RESPONSE\_TO\_UVB\_RADIATION\_UP |  | 28 | -0.45 | -1.26 | 0.175 | 0.299 | 1.000 | 1770 | tags=43%, list=31%, signal=62% |
| 639 | BLALOCK\_ALZHEIMERS\_DISEASE\_INCIPIENT\_DN |  | 82 | -0.39 | -1.26 | 0.123 | 0.299 | 1.000 | 2114 | tags=49%, list=37%, signal=76% |
| 640 | ACEVEDO\_LIVER\_CANCER\_WITH\_H3K27ME3\_DN |  | 29 | -0.45 | -1.26 | 0.164 | 0.299 | 1.000 | 1853 | tags=59%, list=32%, signal=86% |
| 641 | TIEN\_INTESTINE\_PROBIOTICS\_2HR\_DN |  | 44 | -0.42 | -1.26 | 0.160 | 0.300 | 1.000 | 1424 | tags=41%, list=25%, signal=54% |
| 642 | COULOUARN\_TEMPORAL\_TGFB1\_SIGNATURE\_DN |  | 39 | -0.43 | -1.25 | 0.180 | 0.307 | 1.000 | 565 | tags=26%, list=10%, signal=28% |
| 643 | SWEET\_KRAS\_ONCOGENIC\_SIGNATURE |  | 22 | -0.47 | -1.25 | 0.173 | 0.308 | 1.000 | 1458 | tags=45%, list=25%, signal=61% |
| 644 | BROWNE\_HCMV\_INFECTION\_12HR\_UP |  | 48 | -0.41 | -1.25 | 0.155 | 0.307 | 1.000 | 582 | tags=17%, list=10%, signal=18% |
| 645 | NING\_CHRONIC\_OBSTRUCTIVE\_PULMONARY\_DISEASE\_UP |  | 26 | -0.45 | -1.25 | 0.173 | 0.308 | 1.000 | 718 | tags=23%, list=13%, signal=26% |
| 646 | MARTORIATI\_MDM4\_TARGETS\_FETAL\_LIVER\_UP |  | 50 | -0.41 | -1.25 | 0.158 | 0.309 | 1.000 | 2546 | tags=68%, list=44%, signal=121% |
| 647 | SCHLOSSER\_SERUM\_RESPONSE\_DN |  | 365 | -0.35 | -1.25 | 0.025 | 0.309 | 1.000 | 2351 | tags=53%, list=41%, signal=84% |
| 648 | KAAB\_HEART\_ATRIUM\_VS\_VENTRICLE\_DN |  | 108 | -0.37 | -1.25 | 0.103 | 0.310 | 1.000 | 1118 | tags=23%, list=20%, signal=28% |
| 649 | TENEDINI\_MEGAKARYOCYTE\_MARKERS |  | 21 | -0.47 | -1.25 | 0.194 | 0.315 | 1.000 | 542 | tags=19%, list=9%, signal=21% |
| 650 | GUTIERREZ\_CHRONIC\_LYMPHOCYTIC\_LEUKEMIA\_DN |  | 24 | -0.46 | -1.25 | 0.185 | 0.315 | 1.000 | 578 | tags=21%, list=10%, signal=23% |
| 651 | KEGG\_TRYPTOPHAN\_METABOLISM |  | 19 | -0.48 | -1.25 | 0.208 | 0.318 | 1.000 | 459 | tags=16%, list=8%, signal=17% |
| 652 | CASTELLANO\_NRAS\_TARGETS\_UP |  | 23 | -0.47 | -1.25 | 0.193 | 0.319 | 1.000 | 2003 | tags=65%, list=35%, signal=100% |
| 653 | REACTOME\_NEF\_MEDIATES\_DOWN\_MODULATION\_OF\_CELL\_SURFACE\_RECEPTORS\_BY\_RECRUITING\_THEM\_TO\_CLATHRIN\_ADAPTERS |  | 15 | -0.50 | -1.24 | 0.213 | 0.321 | 1.000 | 528 | tags=27%, list=9%, signal=29% |
| 654 | LINDGREN\_BLADDER\_CANCER\_CLUSTER\_3\_DN |  | 83 | -0.39 | -1.24 | 0.130 | 0.321 | 1.000 | 990 | tags=24%, list=17%, signal=29% |
| 655 | MARTINEZ\_RB1\_TARGETS\_DN |  | 166 | -0.36 | -1.24 | 0.077 | 0.324 | 1.000 | 1226 | tags=27%, list=21%, signal=33% |
| 656 | MARTINEZ\_RESPONSE\_TO\_TRABECTEDIN |  | 22 | -0.46 | -1.24 | 0.206 | 0.323 | 1.000 | 1919 | tags=50%, list=34%, signal=75% |
| 657 | BOYLAN\_MULTIPLE\_MYELOMA\_D\_UP |  | 37 | -0.42 | -1.24 | 0.174 | 0.324 | 1.000 | 990 | tags=32%, list=17%, signal=39% |
| 658 | SENGUPTA\_NASOPHARYNGEAL\_CARCINOMA\_UP |  | 131 | -0.37 | -1.24 | 0.091 | 0.324 | 1.000 | 860 | tags=21%, list=15%, signal=25% |
| 659 | RIZKI\_TUMOR\_INVASIVENESS\_3D\_DN |  | 100 | -0.38 | -1.24 | 0.123 | 0.324 | 1.000 | 1240 | tags=30%, list=22%, signal=38% |
| 660 | VANTVEER\_BREAST\_CANCER\_ESR1\_UP |  | 53 | -0.40 | -1.24 | 0.152 | 0.327 | 1.000 | 883 | tags=23%, list=15%, signal=27% |
| 661 | KEGG\_N\_GLYCAN\_BIOSYNTHESIS |  | 27 | -0.45 | -1.24 | 0.188 | 0.331 | 1.000 | 2334 | tags=59%, list=41%, signal=100% |
| 662 | LIU\_PROSTATE\_CANCER\_UP |  | 30 | -0.44 | -1.24 | 0.181 | 0.331 | 1.000 | 914 | tags=30%, list=16%, signal=36% |
| 663 | LEE\_EARLY\_T\_LYMPHOCYTE\_DN |  | 18 | -0.48 | -1.24 | 0.219 | 0.331 | 1.000 | 717 | tags=28%, list=13%, signal=32% |
| 664 | KEGG\_MELANOGENESIS |  | 29 | -0.45 | -1.23 | 0.193 | 0.339 | 1.000 | 678 | tags=31%, list=12%, signal=35% |
| 665 | TSENG\_IRS1\_TARGETS\_UP |  | 43 | -0.41 | -1.23 | 0.167 | 0.341 | 1.000 | 671 | tags=21%, list=12%, signal=24% |
| 666 | UEDA\_PERIFERAL\_CLOCK |  | 57 | -0.39 | -1.23 | 0.174 | 0.341 | 1.000 | 1494 | tags=37%, list=26%, signal=49% |
| 667 | MAYBURD\_RESPONSE\_TO\_L663536\_DN |  | 27 | -0.44 | -1.23 | 0.187 | 0.342 | 1.000 | 2015 | tags=48%, list=35%, signal=74% |
| 668 | FAELT\_B\_CLL\_WITH\_VH3\_21\_DN |  | 23 | -0.46 | -1.23 | 0.204 | 0.343 | 1.000 | 1454 | tags=43%, list=25%, signal=58% |
| 669 | HOFFMANN\_SMALL\_PRE\_BII\_TO\_IMMATURE\_B\_LYMPHOCYTE\_UP |  | 17 | -0.49 | -1.23 | 0.203 | 0.346 | 1.000 | 962 | tags=35%, list=17%, signal=42% |
| 670 | MULLIGHAN\_NPM1\_SIGNATURE\_3\_DN |  | 53 | -0.40 | -1.23 | 0.168 | 0.347 | 1.000 | 1294 | tags=34%, list=23%, signal=43% |
| 671 | SASSON\_RESPONSE\_TO\_GONADOTROPHINS\_UP |  | 29 | -0.43 | -1.23 | 0.215 | 0.347 | 1.000 | 1589 | tags=34%, list=28%, signal=47% |
| 672 | MENSE\_HYPOXIA\_UP |  | 41 | -0.41 | -1.23 | 0.169 | 0.347 | 1.000 | 1838 | tags=41%, list=32%, signal=61% |
| 673 | REACTOME\_DIABETES\_PATHWAYS |  | 146 | -0.36 | -1.23 | 0.100 | 0.347 | 1.000 | 2228 | tags=49%, list=39%, signal=78% |
| 674 | LEE\_TARGETS\_OF\_PTCH1\_AND\_SUFU\_DN |  | 16 | -0.49 | -1.22 | 0.203 | 0.351 | 1.000 | 1181 | tags=50%, list=21%, signal=63% |
| 675 | FRIDMAN\_IMMORTALIZATION\_DN |  | 16 | -0.49 | -1.22 | 0.225 | 0.351 | 1.000 | 884 | tags=44%, list=15%, signal=52% |
| 676 | MULLIGHAN\_NPM1\_MUTATED\_SIGNATURE\_1\_DN |  | 45 | -0.41 | -1.22 | 0.193 | 0.352 | 1.000 | 1294 | tags=31%, list=23%, signal=40% |
| 677 | ENK\_UV\_RESPONSE\_KERATINOCYTE\_UP |  | 218 | -0.35 | -1.22 | 0.080 | 0.353 | 1.000 | 1202 | tags=24%, list=21%, signal=30% |
| 678 | KEGG\_LYSOSOME |  | 72 | -0.38 | -1.22 | 0.181 | 0.357 | 1.000 | 1425 | tags=35%, list=25%, signal=46% |
| 679 | DOUGLAS\_BMI1\_TARGETS\_DN |  | 149 | -0.36 | -1.22 | 0.104 | 0.359 | 1.000 | 981 | tags=23%, list=17%, signal=28% |
| 680 | DORSAM\_HOXA9\_TARGETS\_DN |  | 15 | -0.50 | -1.22 | 0.215 | 0.359 | 1.000 | 1461 | tags=40%, list=26%, signal=54% |
| 681 | FARMER\_BREAST\_CANCER\_APOCRINE VS LUMINAL |  | 123 | -0.36 | -1.22 | 0.128 | 0.360 | 1.000 | 1183 | tags=33%, list=21%, signal=40% |
| 682 | MOOTHA\_PGC |  | 155 | -0.36 | -1.22 | 0.110 | 0.361 | 1.000 | 2076 | tags=43%, list=36%, signal=66% |
| 683 | ZHAN\_MULTIPLE\_MYELOMA\_CD1\_DN |  | 20 | -0.46 | -1.22 | 0.229 | 0.362 | 1.000 | 1267 | tags=40%, list=22%, signal=51% |
| 684 | SASSON\_RESPONSE\_TO\_FORSKOLIN\_DN |  | 35 | -0.42 | -1.21 | 0.192 | 0.362 | 1.000 | 830 | tags=26%, list=15%, signal=30% |
| 685 | KEGG\_PYRUVATE\_METABOLISM |  | 17 | -0.48 | -1.21 | 0.238 | 0.364 | 1.000 | 693 | tags=24%, list=12%, signal=27% |
| 686 | BERENJENO\_ROCK\_SIGNALING\_NOT\_VIA\_RHOA\_DN |  | 22 | -0.47 | -1.21 | 0.217 | 0.365 | 1.000 | 1714 | tags=59%, list=30%, signal=84% |
| 687 | MARSON\_FOXP3\_TARGETS\_DN |  | 17 | -0.48 | -1.21 | 0.233 | 0.366 | 1.000 | 458 | tags=24%, list=8%, signal=26% |
| 688 | MARZEC\_IL2\_SIGNALING\_DN |  | 21 | -0.46 | -1.21 | 0.232 | 0.366 | 1.000 | 1585 | tags=43%, list=28%, signal=59% |
| 689 | ACEVEDO\_LIVER\_CANCER\_WITH\_H3K9ME3\_UP |  | 24 | -0.44 | -1.21 | 0.218 | 0.366 | 1.000 | 1430 | tags=58%, list=25%, signal=77% |
| 690 | REACTOME\_DOWNSTREAM\_SIGNALING\_OF\_ACTIVATED\_FGFR |  | 15 | -0.50 | -1.21 | 0.225 | 0.369 | 1.000 | 18 | tags=7%, list=0%, signal=7% |
| 691 | IVANOVA\_HEMATOPOIESIS\_STEM\_CELL |  | 32 | -0.42 | -1.21 | 0.223 | 0.370 | 1.000 | 718 | tags=22%, list=13%, signal=25% |
| 692 | OUELLET\_OVARIAN\_CANCER\_INVASIVE\_VS\_LMP\_UP |  | 67 | -0.38 | -1.21 | 0.182 | 0.370 | 1.000 | 1759 | tags=45%, list=31%, signal=64% |
| 693 | LINDSTEDT\_DENDRITIC\_CELL\_MATURATION\_B |  | 23 | -0.45 | -1.21 | 0.222 | 0.373 | 1.000 | 928 | tags=30%, list=16%, signal=36% |
| 694 | TONKS\_TARGETS\_OF\_RUNX1\_RUNX1T1\_FUSION\_MONOCYTE\_UP |  | 94 | -0.37 | -1.21 | 0.154 | 0.375 | 1.000 | 661 | tags=20%, list=12%, signal=22% |
| 695 | YAO\_TEMPORAL\_RESPONSE\_TO\_PROGESTERONE\_CLUSTER\_17 |  | 100 | -0.36 | -1.21 | 0.156 | 0.376 | 1.000 | 2639 | tags=56%, list=46%, signal=102% |
| 696 | NATSUME\_RESPONSE\_TO\_INTERFERON\_BETA\_UP |  | 26 | -0.44 | -1.20 | 0.238 | 0.377 | 1.000 | 1100 | tags=35%, list=19%, signal=43% |
| 697 | ZHOU\_INFLAMMATORY\_RESPONSE\_LIVE\_UP |  | 115 | -0.36 | -1.20 | 0.152 | 0.377 | 1.000 | 989 | tags=26%, list=17%, signal=31% |
| 698 | NIKOLSKY\_BREAST\_CANCER\_8Q12\_Q22\_AMPLICON |  | 49 | -0.40 | -1.20 | 0.191 | 0.378 | 1.000 | 2076 | tags=59%, list=36%, signal=92% |
| 699 | SESTO\_RESPONSE\_TO\_UV\_C7 |  | 32 | -0.42 | -1.20 | 0.224 | 0.379 | 1.000 | 1449 | tags=38%, list=25%, signal=50% |
| 700 | YAUCH\_HEDGEHOG\_SIGNALING\_PARACRINE\_UP |  | 26 | -0.44 | -1.20 | 0.234 | 0.378 | 1.000 | 787 | tags=35%, list=14%, signal=40% |
| 701 | ZHAN\_V2\_LATE\_DIFFERENTIATION\_GENES |  | 18 | -0.47 | -1.20 | 0.229 | 0.378 | 1.000 | 2512 | tags=72%, list=44%, signal=128% |
| 702 | BIOCARTA\_AGR\_PATHWAY |  | 16 | -0.48 | -1.20 | 0.240 | 0.377 | 1.000 | 1163 | tags=38%, list=20%, signal=47% |
| 703 | BROWNE\_HCMV\_INFECTION\_4HR\_UP |  | 18 | -0.47 | -1.20 | 0.233 | 0.379 | 1.000 | 782 | tags=33%, list=14%, signal=38% |
| 704 | ZHOU\_INFLAMMATORY\_RESPONSE\_LPS\_UP |  | 102 | -0.36 | -1.20 | 0.156 | 0.381 | 1.000 | 1160 | tags=29%, list=20%, signal=36% |
| 705 | KRIGE\_RESPONSE\_TO\_TOSEDOSTAT\_6HR\_UP |  | 417 | -0.34 | -1.20 | 0.055 | 0.380 | 1.000 | 1792 | tags=37%, list=31%, signal=50% |
| 706 | SENESE\_HDAC3\_TARGETS\_UP |  | 221 | -0.34 | -1.20 | 0.096 | 0.380 | 1.000 | 1190 | tags=27%, list=21%, signal=32% |
| 707 | NUYTTEN\_NIPP1\_TARGETS\_UP |  | 286 | -0.34 | -1.20 | 0.075 | 0.381 | 1.000 | 1755 | tags=36%, list=31%, signal=49% |
| 708 | MOREAUX\_MULTIPLE\_MYELOMA\_BY\_TACI\_UP |  | 70 | -0.38 | -1.20 | 0.195 | 0.381 | 1.000 | 731 | tags=19%, list=13%, signal=21% |
| 709 | WANG\_CLIM2\_TARGETS\_DN |  | 87 | -0.37 | -1.20 | 0.197 | 0.383 | 1.000 | 1926 | tags=41%, list=34%, signal=61% |
| 710 | VANTVEER\_BREAST\_CANCER\_ESR1\_DN |  | 91 | -0.36 | -1.20 | 0.156 | 0.385 | 1.000 | 898 | tags=23%, list=16%, signal=27% |
| 711 | MOOTHA\_VOXPHOS |  | 38 | -0.41 | -1.20 | 0.229 | 0.386 | 1.000 | 2492 | tags=58%, list=44%, signal=102% |
| 712 | BLALOCK\_ALZHEIMERS\_DISEASE\_INCIPIENT\_UP |  | 163 | -0.34 | -1.19 | 0.145 | 0.390 | 1.000 | 1792 | tags=36%, list=31%, signal=51% |
| 713 | WONG\_MITOCHONDRIA\_GENE\_MODULE |  | 105 | -0.36 | -1.19 | 0.176 | 0.391 | 1.000 | 1875 | tags=42%, list=33%, signal=61% |
| 714 | CHUNG\_BLISTER\_CYTOTOXICITY\_UP |  | 59 | -0.38 | -1.19 | 0.211 | 0.391 | 1.000 | 1620 | tags=37%, list=28%, signal=51% |
| 715 | BIOCARTA\_INTEGRIN\_PATHWAY |  | 25 | -0.43 | -1.19 | 0.243 | 0.390 | 1.000 | 1605 | tags=40%, list=28%, signal=55% |
| 716 | MMS\_MOUSE\_LYMPH\_HIGH\_4HRS\_UP |  | 21 | -0.45 | -1.19 | 0.245 | 0.392 | 1.000 | 2310 | tags=62%, list=40%, signal=103% |
| 717 | YAUCH\_HEDGEHOG\_SIGNALING\_PARACRINE\_DN |  | 54 | -0.39 | -1.19 | 0.204 | 0.392 | 1.000 | 662 | tags=19%, list=12%, signal=21% |
| 718 | SHAFFER\_IRF4\_TARGETS\_IN\_ACTIVATED\_DENDRITIC\_CELL |  | 30 | -0.42 | -1.19 | 0.230 | 0.392 | 1.000 | 1826 | tags=50%, list=32%, signal=73% |
| 719 | SAKAI\_TUMOR\_INFILTRATING\_MONOCYTES\_DN |  | 48 | -0.39 | -1.19 | 0.216 | 0.393 | 1.000 | 2306 | tags=54%, list=40%, signal=90% |
| 720 | ST\_INTEGRIN\_SIGNALING\_PATHWAY |  | 42 | -0.40 | -1.19 | 0.229 | 0.392 | 1.000 | 1605 | tags=38%, list=28%, signal=53% |
| 721 | IVANOVA\_HEMATOPOIESIS\_STEM\_CELL\_AND\_PROGENITOR |  | 87 | -0.36 | -1.19 | 0.187 | 0.393 | 1.000 | 1129 | tags=30%, list=20%, signal=37% |
| 722 | ROPERO\_HDAC2\_TARGETS |  | 26 | -0.43 | -1.19 | 0.243 | 0.396 | 1.000 | 1506 | tags=38%, list=26%, signal=52% |
| 723 | MARZEC\_IL2\_SIGNALING\_UP |  | 45 | -0.40 | -1.19 | 0.240 | 0.401 | 1.000 | 612 | tags=18%, list=11%, signal=20% |
| 724 | RODRIGUES\_DCC\_TARGETS\_DN |  | 65 | -0.38 | -1.18 | 0.199 | 0.405 | 1.000 | 1193 | tags=28%, list=21%, signal=35% |
| 725 | KEGG\_CALCIUM\_SIGNALING\_PATHWAY |  | 43 | -0.40 | -1.18 | 0.247 | 0.406 | 1.000 | 548 | tags=21%, list=10%, signal=23% |
| 726 | FARMER\_BREAST\_CANCER\_APOCRINE\_VS\_BASAL |  | 125 | -0.35 | -1.18 | 0.164 | 0.406 | 1.000 | 1183 | tags=30%, list=21%, signal=37% |
| 727 | ROSS\_LEUKEMIA\_WITH\_MLL\_FUSIONS |  | 36 | -0.41 | -1.18 | 0.249 | 0.409 | 1.000 | 1344 | tags=39%, list=23%, signal=51% |
| 728 | WANG\_SMARCE1\_TARGETS\_DN |  | 143 | -0.35 | -1.18 | 0.162 | 0.409 | 1.000 | 642 | tags=16%, list=11%, signal=18% |
| 729 | FLOTHO\_PEDIATRIC\_ALL\_THERAPY\_RESPONSE\_UP |  | 20 | -0.45 | -1.18 | 0.272 | 0.410 | 1.000 | 1651 | tags=45%, list=29%, signal=63% |
| 730 | ZHAN\_MULTIPLE\_MYELOMA\_CD1\_AND\_CD2\_DN |  | 25 | -0.43 | -1.18 | 0.267 | 0.412 | 1.000 | 930 | tags=36%, list=16%, signal=43% |
| 731 | ROSS\_AML\_WITH\_MLL\_FUSIONS |  | 34 | -0.41 | -1.18 | 0.246 | 0.418 | 1.000 | 2227 | tags=53%, list=39%, signal=86% |
| 732 | LIANG\_HEMATOPOIESIS\_STEM\_CELL\_NUMBER\_LARGE\_VS\_TINY\_DN |  | 22 | -0.44 | -1.18 | 0.267 | 0.419 | 1.000 | 1053 | tags=27%, list=18%, signal=33% |
| 733 | BYSTRYKH\_HEMATOPOIESIS\_STEM\_CELL\_QTL\_TRANS |  | 343 | -0.33 | -1.18 | 0.087 | 0.419 | 1.000 | 1756 | tags=36%, list=31%, signal=48% |
| 734 | KAYO\_AGING\_MUSCLE\_DN |  | 59 | -0.37 | -1.18 | 0.210 | 0.418 | 1.000 | 1162 | tags=29%, list=20%, signal=36% |
| 735 | XU\_GH1\_AUTOCRINE\_TARGETS\_DN |  | 45 | -0.39 | -1.17 | 0.236 | 0.420 | 1.000 | 983 | tags=24%, list=17%, signal=29% |
| 736 | SMITH\_TERT\_TARGETS\_DN |  | 31 | -0.42 | -1.17 | 0.256 | 0.419 | 1.000 | 567 | tags=23%, list=10%, signal=25% |
| 737 | FIRESTEIN\_PROLIFERATION |  | 70 | -0.37 | -1.17 | 0.211 | 0.419 | 1.000 | 1026 | tags=26%, list=18%, signal=31% |
| 738 | BROWNE\_HCMV\_INFECTION\_6HR\_DN |  | 71 | -0.37 | -1.17 | 0.207 | 0.420 | 1.000 | 1498 | tags=37%, list=26%, signal=49% |
| 739 | DE\_YY1\_TARGETS\_DN |  | 52 | -0.38 | -1.17 | 0.236 | 0.419 | 1.000 | 2362 | tags=48%, list=41%, signal=81% |
| 740 | IWANAGA\_CARCINOGENESIS\_BY\_KRAS\_PTEN\_UP |  | 67 | -0.37 | -1.17 | 0.218 | 0.420 | 1.000 | 482 | tags=13%, list=8%, signal=14% |
| 741 | GOLDRATH\_HOMEOSTATIC\_PROLIFERATION |  | 88 | -0.36 | -1.17 | 0.206 | 0.421 | 1.000 | 1756 | tags=42%, list=31%, signal=60% |
| 742 | OSMAN\_BLADDER\_CANCER\_UP |  | 218 | -0.33 | -1.17 | 0.130 | 0.420 | 1.000 | 1795 | tags=37%, list=31%, signal=52% |
| 743 | YAGI\_AML\_WITH\_INV\_16\_TRANSLOCATION |  | 151 | -0.34 | -1.17 | 0.169 | 0.421 | 1.000 | 1239 | tags=25%, list=22%, signal=30% |
| 744 | BROWNE\_HCMV\_INFECTION\_1HR\_DN |  | 79 | -0.35 | -1.17 | 0.230 | 0.421 | 1.000 | 1019 | tags=20%, list=18%, signal=24% |
| 745 | ZHAN\_MULTIPLE\_MYELOMA\_HP\_UP |  | 16 | -0.47 | -1.17 | 0.277 | 0.421 | 1.000 | 1019 | tags=25%, list=18%, signal=30% |
| 746 | SHAFFER\_IRF4\_MULTIPLE\_MYELOMA\_PROGRAM |  | 17 | -0.47 | -1.17 | 0.273 | 0.421 | 1.000 | 1838 | tags=53%, list=32%, signal=78% |
| 747 | REACTOME\_GOLGI\_ASSOCIATED\_VESICLE\_BIOGENESIS |  | 26 | -0.43 | -1.17 | 0.268 | 0.424 | 1.000 | 1201 | tags=38%, list=21%, signal=48% |
| 748 | OKUMURA\_INFLAMMATORY\_RESPONSE\_LPS |  | 70 | -0.37 | -1.17 | 0.230 | 0.424 | 1.000 | 657 | tags=19%, list=11%, signal=21% |
| 749 | RASHI\_RESPONSE\_TO\_IONIZING\_RADIATION\_6 |  | 38 | -0.39 | -1.17 | 0.267 | 0.426 | 1.000 | 1273 | tags=34%, list=22%, signal=44% |
| 750 | BOYAULT\_LIVER\_CANCER\_SUBCLASS\_G3\_DN |  | 17 | -0.46 | -1.17 | 0.270 | 0.425 | 1.000 | 1099 | tags=29%, list=19%, signal=36% |
| 751 | ACEVEDO\_LIVER\_TUMOR\_VS\_NORMAL\_ADJACENT\_TISSUE\_UP |  | 437 | -0.33 | -1.17 | 0.073 | 0.426 | 1.000 | 2016 | tags=42%, list=35%, signal=60% |
| 752 | WATANABE\_RECTAL\_CANCER\_RADIOTHERAPY\_RESPONSIVE\_DN |  | 51 | -0.38 | -1.16 | 0.250 | 0.434 | 1.000 | 1461 | tags=35%, list=26%, signal=47% |
| 753 | BOYAULT\_LIVER\_CANCER\_SUBCLASS\_G1\_UP |  | 47 | -0.38 | -1.16 | 0.249 | 0.434 | 1.000 | 1091 | tags=28%, list=19%, signal=34% |
| 754 | SEITZ\_NEOPLASTIC\_TRANSFORMATION\_BY\_8P\_DELETION\_UP |  | 29 | -0.41 | -1.16 | 0.280 | 0.437 | 1.000 | 828 | tags=24%, list=14%, signal=28% |
| 755 | YAO\_TEMPORAL\_RESPONSE\_TO\_PROGESTERONE\_CLUSTER\_9 |  | 33 | -0.41 | -1.16 | 0.289 | 0.441 | 1.000 | 1481 | tags=48%, list=26%, signal=65% |
| 756 | REACTOME\_OPIOID\_SIGNALLING |  | 34 | -0.41 | -1.16 | 0.277 | 0.443 | 1.000 | 1070 | tags=26%, list=19%, signal=32% |
| 757 | KEGG\_LONG\_TERM\_DEPRESSION |  | 29 | -0.41 | -1.16 | 0.272 | 0.442 | 1.000 | 1746 | tags=48%, list=31%, signal=69% |
| 758 | WANG\_LMO4\_TARGETS\_UP |  | 147 | -0.34 | -1.16 | 0.181 | 0.445 | 1.000 | 1630 | tags=33%, list=28%, signal=45% |
| 759 | AMBROSINI\_FLAVOPIRIDOL\_TREATMENT\_TP53 |  | 39 | -0.39 | -1.16 | 0.272 | 0.444 | 1.000 | 869 | tags=26%, list=15%, signal=30% |
| 760 | DITTMER\_PTHLH\_TARGETS\_UP |  | 65 | -0.36 | -1.16 | 0.249 | 0.446 | 1.000 | 1535 | tags=35%, list=27%, signal=48% |
| 761 | REACTOME\_PURINE\_METABOLISM |  | 18 | -0.46 | -1.16 | 0.277 | 0.447 | 1.000 | 1030 | tags=33%, list=18%, signal=41% |
| 762 | BOHN\_PRIMARY\_IMMUNODEFICIENCY\_SYNDROM\_UP |  | 21 | -0.44 | -1.16 | 0.289 | 0.447 | 1.000 | 555 | tags=19%, list=10%, signal=21% |
| 763 | CHNG\_MULTIPLE\_MYELOMA\_HYPERPLOID\_DN |  | 17 | -0.45 | -1.16 | 0.321 | 0.446 | 1.000 | 1937 | tags=59%, list=34%, signal=89% |
| 764 | CHUNG\_BLISTER\_CYTOTOXICITY\_DN |  | 23 | -0.42 | -1.15 | 0.278 | 0.447 | 1.000 | 965 | tags=22%, list=17%, signal=26% |
| 765 | JAZAG\_TGFB1\_SIGNALING\_VIA\_SMAD4\_DN |  | 19 | -0.44 | -1.15 | 0.300 | 0.446 | 1.000 | 623 | tags=21%, list=11%, signal=24% |
| 766 | KEGG\_PROSTATE\_CANCER |  | 45 | -0.38 | -1.15 | 0.278 | 0.448 | 1.000 | 1324 | tags=29%, list=23%, signal=37% |
| 767 | FERNANDEZ\_BOUND\_BY\_MYC |  | 65 | -0.36 | -1.15 | 0.255 | 0.448 | 1.000 | 1792 | tags=43%, list=31%, signal=62% |
| 768 | YEGNASUBRAMANIAN\_PROSTATE\_CANCER |  | 35 | -0.40 | -1.15 | 0.278 | 0.453 | 1.000 | 1053 | tags=26%, list=18%, signal=31% |
| 769 | CROMER\_METASTASIS\_UP |  | 30 | -0.41 | -1.15 | 0.284 | 0.455 | 1.000 | 1161 | tags=40%, list=20%, signal=50% |
| 770 | CHAUHAN\_RESPONSE\_TO\_METHOXYESTRADIOL\_DN |  | 45 | -0.38 | -1.15 | 0.253 | 0.456 | 1.000 | 1903 | tags=42%, list=33%, signal=63% |
| 771 | SPIELMAN\_LYMPHOBLAST\_EUROPEAN\_VS\_ASIAN\_DN |  | 335 | -0.32 | -1.15 | 0.131 | 0.458 | 1.000 | 2518 | tags=53%, list=44%, signal=90% |
| 772 | BROWNE\_HCMV\_INFECTION\_14HR\_DN |  | 117 | -0.34 | -1.15 | 0.237 | 0.458 | 1.000 | 804 | tags=20%, list=14%, signal=22% |
| 773 | SHEPARD\_BMYB\_MORPHOLINO\_UP |  | 60 | -0.36 | -1.15 | 0.253 | 0.458 | 1.000 | 2130 | tags=48%, list=37%, signal=76% |
| 774 | OUELLET\_CULTURED\_OVARIAN\_CANCER\_INVASIVE\_VS\_LMP\_UP |  | 31 | -0.40 | -1.15 | 0.281 | 0.457 | 1.000 | 1335 | tags=35%, list=23%, signal=46% |
| 775 | GENTILE\_UV\_RESPONSE\_CLUSTER\_D4 |  | 32 | -0.40 | -1.15 | 0.295 | 0.459 | 1.000 | 1079 | tags=31%, list=19%, signal=38% |
| 776 | ZHAN\_MULTIPLE\_MYELOMA\_CD1\_VS\_CD2\_DN |  | 19 | -0.44 | -1.15 | 0.295 | 0.460 | 1.000 | 595 | tags=26%, list=10%, signal=29% |
| 777 | REACTOME\_MEMBRANE\_TRAFFICKING |  | 47 | -0.38 | -1.15 | 0.266 | 0.460 | 1.000 | 1522 | tags=38%, list=27%, signal=52% |
| 778 | DAZARD\_RESPONSE\_TO\_UV\_NHEK\_UP |  | 49 | -0.37 | -1.14 | 0.291 | 0.460 | 1.000 | 921 | tags=18%, list=16%, signal=22% |
| 779 | FRASOR\_TAMOXIFEN\_RESPONSE\_UP |  | 21 | -0.43 | -1.14 | 0.281 | 0.461 | 1.000 | 2057 | tags=67%, list=36%, signal=104% |
| 780 | KIM\_WT1\_TARGETS\_DN |  | 231 | -0.33 | -1.14 | 0.165 | 0.462 | 1.000 | 1475 | tags=30%, list=26%, signal=39% |
| 781 | CHAUHAN\_RESPONSE\_TO\_METHOXYESTRADIOL\_UP |  | 16 | -0.45 | -1.14 | 0.314 | 0.462 | 1.000 | 596 | tags=19%, list=10%, signal=21% |
| 782 | CREIGHTON\_ENDOCRINE\_THERAPY\_RESISTANCE\_4 |  | 114 | -0.34 | -1.14 | 0.227 | 0.461 | 1.000 | 1189 | tags=25%, list=21%, signal=31% |
| 783 | DAIRKEE\_TERT\_TARGETS\_DN |  | 36 | -0.40 | -1.14 | 0.283 | 0.462 | 1.000 | 724 | tags=25%, list=13%, signal=28% |
| 784 | LIN\_NPAS4\_TARGETS\_DN |  | 19 | -0.44 | -1.14 | 0.314 | 0.465 | 1.000 | 819 | tags=32%, list=14%, signal=37% |
| 785 | CHEMNITZ\_RESPONSE\_TO\_PROSTAGLANDIN\_E2\_DN |  | 119 | -0.34 | -1.14 | 0.219 | 0.465 | 1.000 | 1038 | tags=28%, list=18%, signal=33% |
| 786 | REACTOME\_NCAM\_SIGNALING\_FOR\_NEURITE\_OUT\_GROWTH |  | 23 | -0.42 | -1.14 | 0.296 | 0.465 | 1.000 | 1305 | tags=39%, list=23%, signal=50% |
| 787 | KEGG\_FC\_EPSILON\_RI\_SIGNALING\_PATHWAY |  | 42 | -0.38 | -1.14 | 0.286 | 0.464 | 1.000 | 682 | tags=19%, list=12%, signal=21% |
| 788 | AMIT\_EGF\_RESPONSE\_60\_HELA |  | 18 | -0.44 | -1.14 | 0.302 | 0.469 | 1.000 | 1198 | tags=39%, list=21%, signal=49% |
| 789 | ACEVEDO\_LIVER\_CANCER\_DN |  | 178 | -0.33 | -1.14 | 0.195 | 0.469 | 1.000 | 990 | tags=20%, list=17%, signal=24% |
| 790 | BIOCARTA\_RAC1\_PATHWAY |  | 15 | -0.48 | -1.14 | 0.320 | 0.468 | 1.000 | 443 | tags=20%, list=8%, signal=22% |
| 791 | BUYTAERT\_PHOTODYNAMIC\_THERAPY\_STRESS\_UP |  | 390 | -0.32 | -1.14 | 0.137 | 0.470 | 1.000 | 1931 | tags=39%, list=34%, signal=56% |
| 792 | ACEVEDO\_LIVER\_CANCER\_WITH\_H3K9ME3\_DN |  | 32 | -0.39 | -1.14 | 0.294 | 0.472 | 1.000 | 817 | tags=25%, list=14%, signal=29% |
| 793 | WANG\_CLIM2\_TARGETS\_UP |  | 83 | -0.35 | -1.14 | 0.275 | 0.472 | 1.000 | 1212 | tags=25%, list=21%, signal=32% |
| 794 | BECKER\_TAMOXIFEN\_RESISTANCE\_UP |  | 15 | -0.46 | -1.14 | 0.302 | 0.472 | 1.000 | 1678 | tags=60%, list=29%, signal=85% |
| 795 | LINDGREN\_BLADDER\_CANCER\_CLUSTER\_1\_UP |  | 46 | -0.37 | -1.14 | 0.298 | 0.472 | 1.000 | 1358 | tags=30%, list=24%, signal=40% |
| 796 | THUM\_SYSTOLIC\_HEART\_FAILURE\_DN |  | 80 | -0.35 | -1.13 | 0.270 | 0.472 | 1.000 | 1172 | tags=24%, list=20%, signal=29% |
| 797 | FARMER\_BREAST\_CANCER\_CLUSTER\_1 |  | 23 | -0.43 | -1.13 | 0.304 | 0.472 | 1.000 | 1083 | tags=39%, list=19%, signal=48% |
| 798 | REACTOME\_REGULATION\_OF\_INSULIN\_SECRETION\_BY\_GLUCAGON\_LIKE\_PEPTIDE\_1 |  | 16 | -0.46 | -1.13 | 0.338 | 0.473 | 1.000 | 1080 | tags=31%, list=19%, signal=38% |
| 799 | CERVERA\_SDHB\_TARGETS\_1\_UP |  | 30 | -0.40 | -1.13 | 0.326 | 0.474 | 1.000 | 726 | tags=30%, list=13%, signal=34% |
| 800 | SESTO\_RESPONSE\_TO\_UV\_C0 |  | 55 | -0.36 | -1.13 | 0.281 | 0.483 | 1.000 | 536 | tags=15%, list=9%, signal=16% |
| 801 | KAYO\_AGING\_MUSCLE\_UP |  | 61 | -0.36 | -1.13 | 0.296 | 0.486 | 1.000 | 800 | tags=20%, list=14%, signal=23% |
| 802 | RASHI\_RESPONSE\_TO\_IONIZING\_RADIATION\_5 |  | 59 | -0.36 | -1.13 | 0.300 | 0.487 | 1.000 | 1353 | tags=29%, list=24%, signal=37% |
| 803 | WILENSKY\_RESPONSE\_TO\_DARAPLADIB |  | 16 | -0.45 | -1.13 | 0.333 | 0.487 | 1.000 | 2303 | tags=69%, list=40%, signal=115% |
| 804 | STEIN\_ESRRA\_TARGETS\_DN |  | 45 | -0.37 | -1.13 | 0.307 | 0.488 | 1.000 | 1033 | tags=27%, list=18%, signal=32% |
| 805 | SASSON\_RESPONSE\_TO\_GONADOTROPHINS\_DN |  | 39 | -0.38 | -1.13 | 0.290 | 0.488 | 1.000 | 957 | tags=28%, list=17%, signal=34% |
| 806 | SHEPARD\_BMYB\_MORPHOLINO\_DN |  | 61 | -0.36 | -1.13 | 0.301 | 0.488 | 1.000 | 856 | tags=23%, list=15%, signal=27% |
| 807 | APPIERTO\_RESPONSE\_TO\_FENRETINIDE\_UP |  | 16 | -0.45 | -1.12 | 0.318 | 0.497 | 1.000 | 1308 | tags=31%, list=23%, signal=40% |
| 808 | BORCZUK\_MALIGNANT\_MESOTHELIOMA\_UP |  | 171 | -0.33 | -1.12 | 0.247 | 0.498 | 1.000 | 2457 | tags=51%, list=43%, signal=86% |
| 809 | YAGI\_AML\_WITH\_T\_8\_21\_TRANSLOCATION |  | 147 | -0.32 | -1.12 | 0.249 | 0.500 | 1.000 | 1032 | tags=24%, list=18%, signal=29% |
| 810 | BIOCARTA\_VEGF\_PATHWAY |  | 20 | -0.44 | -1.12 | 0.344 | 0.503 | 1.000 | 770 | tags=20%, list=13%, signal=23% |
| 811 | IWANAGA\_CARCINOGENESIS\_BY\_KRAS\_DN |  | 42 | -0.37 | -1.12 | 0.322 | 0.509 | 1.000 | 1999 | tags=45%, list=35%, signal=69% |
| 812 | VANTVEER\_BREAST\_CANCER\_METASTASIS\_UP |  | 18 | -0.43 | -1.11 | 0.328 | 0.508 | 1.000 | 613 | tags=17%, list=11%, signal=19% |
| 813 | LIU\_VMYB\_TARGETS\_UP |  | 58 | -0.36 | -1.11 | 0.317 | 0.510 | 1.000 | 2397 | tags=59%, list=42%, signal=100% |
| 814 | KEGG\_PURINE\_METABOLISM |  | 75 | -0.34 | -1.11 | 0.293 | 0.513 | 1.000 | 876 | tags=21%, list=15%, signal=25% |
| 815 | KEGG\_PARKINSONS\_DISEASE |  | 49 | -0.37 | -1.11 | 0.327 | 0.514 | 1.000 | 2687 | tags=63%, list=47%, signal=118% |
| 816 | NUYTTEN\_NIPP1\_TARGETS\_DN |  | 347 | -0.31 | -1.11 | 0.195 | 0.513 | 1.000 | 1671 | tags=34%, list=29%, signal=45% |
| 817 | KYNG\_DNA\_DAMAGE\_DN |  | 41 | -0.38 | -1.11 | 0.333 | 0.515 | 1.000 | 1838 | tags=44%, list=32%, signal=64% |
| 818 | BIOCARTA\_TCR\_PATHWAY |  | 29 | -0.40 | -1.11 | 0.314 | 0.516 | 1.000 | 694 | tags=21%, list=12%, signal=23% |
| 819 | KEGG\_PRIMARY\_IMMUNODEFICIENCY |  | 17 | -0.44 | -1.11 | 0.350 | 0.517 | 1.000 | 528 | tags=35%, list=9%, signal=39% |
| 820 | KEGG\_PORPHYRIN\_AND\_CHLOROPHYLL\_METABOLISM |  | 17 | -0.44 | -1.11 | 0.335 | 0.517 | 1.000 | 1172 | tags=29%, list=20%, signal=37% |
| 821 | GRADE\_COLON\_CANCER\_DN |  | 16 | -0.44 | -1.11 | 0.348 | 0.518 | 1.000 | 5 | tags=6%, list=0%, signal=6% |
| 822 | ZHAN\_MULTIPLE\_MYELOMA\_CD2\_DN |  | 19 | -0.43 | -1.11 | 0.359 | 0.522 | 1.000 | 525 | tags=21%, list=9%, signal=23% |
| 823 | WANG\_ESOPHAGUS\_CANCER\_VS\_NORMAL\_DN |  | 40 | -0.37 | -1.11 | 0.334 | 0.522 | 1.000 | 2005 | tags=48%, list=35%, signal=73% |
| 824 | HATADA\_METHYLATED\_IN\_LUNG\_CANCER\_UP |  | 83 | -0.34 | -1.11 | 0.310 | 0.522 | 1.000 | 932 | tags=29%, list=16%, signal=34% |
| 825 | BHATI\_G2M\_ARREST\_BY\_2METHOXYESTRADIOL\_DN |  | 49 | -0.36 | -1.10 | 0.338 | 0.525 | 1.000 | 1010 | tags=22%, list=18%, signal=27% |
| 826 | RAMALHO\_STEMNESS\_DN |  | 30 | -0.39 | -1.10 | 0.350 | 0.526 | 1.000 | 1243 | tags=37%, list=22%, signal=47% |
| 827 | SESTO\_RESPONSE\_TO\_UV\_C2 |  | 29 | -0.39 | -1.10 | 0.347 | 0.526 | 1.000 | 1585 | tags=41%, list=28%, signal=57% |
| 828 | DORSAM\_HOXA9\_TARGETS\_UP |  | 18 | -0.43 | -1.10 | 0.354 | 0.527 | 1.000 | 1535 | tags=50%, list=27%, signal=68% |
| 829 | KEGG\_ACUTE\_MYELOID\_LEUKEMIA |  | 38 | -0.37 | -1.10 | 0.324 | 0.529 | 1.000 | 357 | tags=13%, list=6%, signal=14% |
| 830 | RIZKI\_TUMOR\_INVASIVENESS\_2D\_UP |  | 24 | -0.41 | -1.10 | 0.354 | 0.530 | 1.000 | 163 | tags=13%, list=3%, signal=13% |
| 831 | BROWNE\_HCMV\_INFECTION\_10HR\_DN |  | 19 | -0.42 | -1.10 | 0.355 | 0.530 | 1.000 | 1371 | tags=47%, list=24%, signal=62% |
| 832 | LU\_AGING\_BRAIN\_UP |  | 63 | -0.35 | -1.10 | 0.324 | 0.532 | 1.000 | 1130 | tags=27%, list=20%, signal=33% |
| 833 | IVANOVA\_HEMATOPOIESIS\_EARLY\_PROGENITOR |  | 58 | -0.35 | -1.10 | 0.342 | 0.535 | 1.000 | 1698 | tags=40%, list=30%, signal=56% |
| 834 | LUCAS\_HNF4A\_TARGETS\_UP |  | 22 | -0.41 | -1.09 | 0.346 | 0.543 | 1.000 | 2034 | tags=64%, list=36%, signal=98% |
| 835 | CADWELL\_ATG16L1\_TARGETS\_DN |  | 28 | -0.39 | -1.09 | 0.344 | 0.543 | 1.000 | 1148 | tags=36%, list=20%, signal=44% |
| 836 | FAELT\_B\_CLL\_WITH\_VH\_REARRANGEMENTS\_UP |  | 22 | -0.41 | -1.09 | 0.345 | 0.546 | 1.000 | 499 | tags=23%, list=9%, signal=25% |
| 837 | SHEN\_SMARCA2\_TARGETS\_DN |  | 38 | -0.37 | -1.09 | 0.355 | 0.548 | 1.000 | 442 | tags=16%, list=8%, signal=17% |
| 838 | DOANE\_BREAST\_CANCER\_CLASSES\_DN |  | 15 | -0.44 | -1.09 | 0.362 | 0.548 | 1.000 | 2324 | tags=73%, list=41%, signal=123% |
| 839 | RHEIN\_ALL\_GLUCOCORTICOID\_THERAPY\_DN |  | 174 | -0.31 | -1.09 | 0.303 | 0.549 | 1.000 | 1693 | tags=33%, list=30%, signal=45% |
| 840 | KEGG\_SNARE\_INTERACTIONS\_IN\_VESICULAR\_TRANSPORT |  | 15 | -0.44 | -1.09 | 0.393 | 0.549 | 1.000 | 1292 | tags=40%, list=23%, signal=52% |
| 841 | MULLIGHAN\_NPM1\_SIGNATURE\_3\_UP |  | 144 | -0.32 | -1.09 | 0.301 | 0.548 | 1.000 | 1458 | tags=28%, list=25%, signal=36% |
| 842 | BROWNE\_HCMV\_INFECTION\_2HR\_DN |  | 21 | -0.42 | -1.09 | 0.359 | 0.552 | 1.000 | 638 | tags=33%, list=11%, signal=37% |
| 843 | WANG\_PROSTATE\_CANCER\_ANDROGEN\_INDEPENDENT |  | 23 | -0.40 | -1.09 | 0.363 | 0.552 | 1.000 | 671 | tags=22%, list=12%, signal=25% |
| 844 | STARK\_PREFRONTAL\_CORTEX\_22Q11\_DELETION\_DN |  | 212 | -0.31 | -1.09 | 0.291 | 0.552 | 1.000 | 2424 | tags=50%, list=42%, signal=84% |
| 845 | KEGG\_MAPK\_SIGNALING\_PATHWAY |  | 96 | -0.33 | -1.09 | 0.328 | 0.554 | 1.000 | 1097 | tags=23%, list=19%, signal=28% |
| 846 | REACTOME\_GLUCOSE\_AND\_OTHER\_SUGAR\_SLC\_TRANSPORTERS |  | 15 | -0.44 | -1.09 | 0.373 | 0.554 | 1.000 | 1577 | tags=47%, list=28%, signal=64% |
| 847 | PICCALUGA\_ANGIOIMMUNOBLASTIC\_LYMPHOMA\_DN |  | 64 | -0.34 | -1.09 | 0.345 | 0.554 | 1.000 | 2394 | tags=50%, list=42%, signal=85% |
| 848 | IWANAGA\_CARCINOGENESIS\_BY\_KRAS\_UP |  | 64 | -0.35 | -1.09 | 0.347 | 0.555 | 1.000 | 2000 | tags=45%, list=35%, signal=69% |
| 849 | RODRIGUES\_NTN1\_TARGETS\_DN |  | 42 | -0.36 | -1.08 | 0.372 | 0.555 | 1.000 | 700 | tags=21%, list=12%, signal=24% |
| 850 | ZHOU\_INFLAMMATORY\_RESPONSE\_FIMA\_DN |  | 89 | -0.33 | -1.08 | 0.371 | 0.556 | 1.000 | 674 | tags=16%, list=12%, signal=18% |
| 851 | BIOCARTA\_GSK3\_PATHWAY |  | 15 | -0.44 | -1.08 | 0.382 | 0.557 | 1.000 | 1303 | tags=40%, list=23%, signal=52% |
| 852 | SASSON\_RESPONSE\_TO\_FORSKOLIN\_UP |  | 33 | -0.38 | -1.08 | 0.356 | 0.560 | 1.000 | 1589 | tags=36%, list=28%, signal=50% |
| 853 | CAFFAREL\_RESPONSE\_TO\_THC\_24HR\_5\_DN |  | 22 | -0.41 | -1.08 | 0.368 | 0.559 | 1.000 | 595 | tags=18%, list=10%, signal=20% |
| 854 | ZHANG\_BREAST\_CANCER\_PROGENITORS\_DN |  | 74 | -0.33 | -1.08 | 0.340 | 0.562 | 1.000 | 1890 | tags=38%, list=33%, signal=56% |
| 855 | YAO\_TEMPORAL\_RESPONSE\_TO\_PROGESTERONE\_CLUSTER\_11 |  | 55 | -0.34 | -1.08 | 0.353 | 0.564 | 1.000 | 1217 | tags=25%, list=21%, signal=32% |
| 856 | KEGG\_PEROXISOME |  | 36 | -0.38 | -1.08 | 0.375 | 0.565 | 1.000 | 2113 | tags=47%, list=37%, signal=74% |
| 857 | MULLIGHAN\_NPM1\_MUTATED\_SIGNATURE\_1\_UP |  | 116 | -0.32 | -1.08 | 0.334 | 0.565 | 1.000 | 1458 | tags=29%, list=25%, signal=39% |
| 858 | KEGG\_ENDOMETRIAL\_CANCER |  | 30 | -0.38 | -1.08 | 0.379 | 0.565 | 1.000 | 336 | tags=13%, list=6%, signal=14% |
| 859 | GENTILE\_UV\_HIGH\_DOSE\_DN |  | 129 | -0.32 | -1.08 | 0.342 | 0.565 | 1.000 | 1426 | tags=29%, list=25%, signal=38% |
| 860 | REACTOME\_CLATHRIN\_DERIVED\_VESICLE\_BUDDING |  | 32 | -0.38 | -1.08 | 0.390 | 0.568 | 1.000 | 1201 | tags=34%, list=21%, signal=43% |
| 861 | KEGG\_SMALL\_CELL\_LUNG\_CANCER |  | 43 | -0.36 | -1.08 | 0.379 | 0.568 | 1.000 | 1163 | tags=26%, list=20%, signal=32% |
| 862 | DITTMER\_PTHLH\_TARGETS\_DN |  | 34 | -0.37 | -1.07 | 0.380 | 0.571 | 1.000 | 1532 | tags=38%, list=27%, signal=52% |
| 863 | TOMLINS\_PROSTATE\_CANCER\_UP |  | 19 | -0.41 | -1.07 | 0.391 | 0.582 | 1.000 | 1436 | tags=32%, list=25%, signal=42% |
| 864 | KEGG\_ALZHEIMERS\_DISEASE |  | 67 | -0.34 | -1.07 | 0.373 | 0.584 | 1.000 | 2518 | tags=58%, list=44%, signal=103% |
| 865 | ELLWOOD\_MYC\_TARGETS\_DN |  | 15 | -0.44 | -1.07 | 0.390 | 0.587 | 1.000 | 2284 | tags=67%, list=40%, signal=111% |
| 866 | KENNY\_CTNNB1\_TARGETS\_DN |  | 22 | -0.40 | -1.07 | 0.388 | 0.588 | 1.000 | 806 | tags=23%, list=14%, signal=26% |
| 867 | MULLIGHAN\_MLL\_SIGNATURE\_2\_DN |  | 112 | -0.32 | -1.07 | 0.377 | 0.589 | 1.000 | 1309 | tags=29%, list=23%, signal=37% |
| 868 | MUNSHI\_MULTIPLE\_MYELOMA\_UP |  | 24 | -0.40 | -1.06 | 0.395 | 0.592 | 1.000 | 1977 | tags=50%, list=35%, signal=76% |
| 869 | CHIARETTI\_T\_ALL\_REFRACTORY\_TO\_THERAPY |  | 16 | -0.43 | -1.06 | 0.414 | 0.592 | 1.000 | 987 | tags=38%, list=17%, signal=45% |
| 870 | ROSS\_AML\_WITH\_AML1\_ETO\_FUSION |  | 24 | -0.39 | -1.06 | 0.408 | 0.595 | 1.000 | 990 | tags=33%, list=17%, signal=40% |
| 871 | ONKEN\_UVEAL\_MELANOMA\_UP |  | 372 | -0.30 | -1.06 | 0.307 | 0.598 | 1.000 | 2009 | tags=39%, list=35%, signal=56% |
| 872 | HENDRICKS\_SMARCA4\_TARGETS\_UP |  | 20 | -0.41 | -1.06 | 0.411 | 0.597 | 1.000 | 1557 | tags=55%, list=27%, signal=75% |
| 873 | ST\_P38\_MAPK\_PATHWAY |  | 17 | -0.42 | -1.06 | 0.409 | 0.598 | 1.000 | 582 | tags=24%, list=10%, signal=26% |
| 874 | DEBIASI\_APOPTOSIS\_BY\_REOVIRUS\_INFECTION\_UP |  | 131 | -0.31 | -1.06 | 0.378 | 0.602 | 1.000 | 2119 | tags=44%, list=37%, signal=68% |
| 875 | GRESHOCK\_CANCER\_COPY\_NUMBER\_UP |  | 145 | -0.31 | -1.06 | 0.387 | 0.605 | 1.000 | 990 | tags=21%, list=17%, signal=24% |
| 876 | KOKKINAKIS\_METHIONINE\_DEPRIVATION\_96HR\_DN |  | 28 | -0.38 | -1.06 | 0.409 | 0.606 | 1.000 | 336 | tags=11%, list=6%, signal=11% |
| 877 | HOFMANN\_CELL\_LYMPHOMA\_UP |  | 29 | -0.38 | -1.05 | 0.404 | 0.608 | 1.000 | 1547 | tags=48%, list=27%, signal=66% |
| 878 | BYSTRYKH\_HEMATOPOIESIS\_STEM\_CELL\_QTL\_CIS |  | 54 | -0.34 | -1.05 | 0.404 | 0.607 | 1.000 | 1352 | tags=35%, list=24%, signal=46% |
| 879 | ENK\_UV\_RESPONSE\_KERATINOCYTE\_DN |  | 277 | -0.30 | -1.05 | 0.347 | 0.608 | 1.000 | 1308 | tags=22%, list=23%, signal=28% |
| 880 | SMID\_BREAST\_CANCER\_ERBB2\_UP |  | 38 | -0.36 | -1.05 | 0.410 | 0.608 | 1.000 | 681 | tags=24%, list=12%, signal=27% |
| 881 | TONG\_INTERACT\_WITH\_PTTG1 |  | 20 | -0.40 | -1.05 | 0.426 | 0.612 | 1.000 | 664 | tags=20%, list=12%, signal=23% |
| 882 | CAFFAREL\_RESPONSE\_TO\_THC\_UP |  | 17 | -0.41 | -1.05 | 0.429 | 0.611 | 1.000 | 1468 | tags=35%, list=26%, signal=47% |
| 883 | SHEPARD\_CRUSH\_AND\_BURN\_MUTANT\_UP |  | 50 | -0.34 | -1.05 | 0.390 | 0.612 | 1.000 | 1585 | tags=26%, list=28%, signal=36% |
| 884 | HUTTMANN\_B\_CLL\_POOR\_SURVIVAL\_DN |  | 28 | -0.38 | -1.05 | 0.422 | 0.613 | 1.000 | 330 | tags=14%, list=6%, signal=15% |
| 885 | BIOCARTA\_CXCR4\_PATHWAY |  | 15 | -0.42 | -1.05 | 0.412 | 0.612 | 1.000 | 1605 | tags=40%, list=28%, signal=55% |
| 886 | LIU\_CMYB\_TARGETS\_UP |  | 66 | -0.33 | -1.05 | 0.414 | 0.615 | 1.000 | 2136 | tags=47%, list=37%, signal=74% |
| 887 | BIOCARTA\_P38MAPK\_PATHWAY |  | 22 | -0.39 | -1.05 | 0.421 | 0.617 | 1.000 | 1016 | tags=23%, list=18%, signal=28% |
| 888 | GRESHOCK\_CANCER\_COPY\_NUMBER\_DN |  | 148 | -0.31 | -1.05 | 0.399 | 0.618 | 1.000 | 990 | tags=20%, list=17%, signal=24% |
| 889 | NAGASHIMA\_NRG1\_SIGNALING\_UP |  | 61 | -0.33 | -1.05 | 0.406 | 0.619 | 1.000 | 843 | tags=21%, list=15%, signal=25% |
| 890 | LEE\_LIVER\_CANCER\_DENA\_DN |  | 20 | -0.40 | -1.05 | 0.427 | 0.620 | 1.000 | 590 | tags=25%, list=10%, signal=28% |
| 891 | BILD\_CTNNB1\_ONCOGENIC\_SIGNATURE |  | 31 | -0.36 | -1.05 | 0.418 | 0.620 | 1.000 | 1837 | tags=39%, list=32%, signal=57% |
| 892 | REACTOME\_METABOLISM\_OF\_LIPIDS\_AND\_LIPOPROTEINS |  | 96 | -0.32 | -1.04 | 0.420 | 0.620 | 1.000 | 1498 | tags=30%, list=26%, signal=40% |
| 893 | KRASNOSELSKAYA\_ILF3\_TARGETS\_DN |  | 17 | -0.41 | -1.04 | 0.407 | 0.625 | 1.000 | 799 | tags=29%, list=14%, signal=34% |
| 894 | KEGG\_NATURAL\_KILLER\_CELL\_MEDIATED\_CYTOTOXICITY |  | 46 | -0.35 | -1.04 | 0.432 | 0.625 | 1.000 | 1083 | tags=24%, list=19%, signal=29% |
| 895 | SAKAI\_CHRONIC\_HEPATITIS\_VS\_LIVER\_CANCER\_UP |  | 35 | -0.36 | -1.04 | 0.428 | 0.625 | 1.000 | 2544 | tags=57%, list=44%, signal=102% |
| 896 | CHIARADONNA\_NEOPLASTIC\_TRANSFORMATION\_KRAS\_UP |  | 54 | -0.33 | -1.04 | 0.415 | 0.625 | 1.000 | 1532 | tags=31%, list=27%, signal=43% |
| 897 | GAZDA\_DIAMOND\_BLACKFAN\_ANEMIA\_ERYTHROID\_DN |  | 200 | -0.30 | -1.04 | 0.394 | 0.625 | 1.000 | 1451 | tags=29%, list=25%, signal=37% |
| 898 | ROSS\_AML\_OF\_FAB\_M7\_TYPE |  | 33 | -0.37 | -1.04 | 0.437 | 0.628 | 1.000 | 1206 | tags=33%, list=21%, signal=42% |
| 899 | SENGUPTA\_NASOPHARYNGEAL\_CARCINOMA\_DN |  | 53 | -0.33 | -1.04 | 0.434 | 0.632 | 1.000 | 1542 | tags=43%, list=27%, signal=59% |
| 900 | HESS\_TARGETS\_OF\_HOXA9\_AND\_MEIS1\_UP |  | 35 | -0.37 | -1.04 | 0.447 | 0.631 | 1.000 | 645 | tags=17%, list=11%, signal=19% |
| 901 | LEE\_LIVER\_CANCER\_MYC\_E2F1\_DN |  | 21 | -0.39 | -1.04 | 0.429 | 0.636 | 1.000 | 767 | tags=29%, list=13%, signal=33% |
| 902 | LUI\_THYROID\_CANCER\_CLUSTER\_1 |  | 22 | -0.39 | -1.03 | 0.437 | 0.637 | 1.000 | 990 | tags=32%, list=17%, signal=38% |
| 903 | ACEVEDO\_NORMAL\_TISSUE\_ADJACENT\_TO\_LIVER\_TUMOR\_UP |  | 78 | -0.32 | -1.03 | 0.429 | 0.637 | 1.000 | 1331 | tags=29%, list=23%, signal=38% |
| 904 | XU\_CREBBP\_TARGETS\_UP |  | 15 | -0.41 | -1.03 | 0.434 | 0.640 | 1.000 | 641 | tags=27%, list=11%, signal=30% |
| 905 | STEARMAN\_LUNG\_CANCER\_EARLY\_VS\_LATE\_DN |  | 24 | -0.38 | -1.03 | 0.455 | 0.640 | 1.000 | 1542 | tags=46%, list=27%, signal=62% |
| 906 | BASSO\_CD40\_SIGNALING\_DN |  | 28 | -0.37 | -1.03 | 0.434 | 0.643 | 1.000 | 500 | tags=21%, list=9%, signal=23% |
| 907 | WINTER\_HYPOXIA\_DN |  | 22 | -0.38 | -1.03 | 0.454 | 0.643 | 1.000 | 1209 | tags=27%, list=21%, signal=34% |
| 908 | DAVICIONI\_MOLECULAR\_ARMS\_VS\_ERMS\_UP |  | 136 | -0.30 | -1.03 | 0.440 | 0.645 | 1.000 | 1554 | tags=33%, list=27%, signal=44% |
| 909 | CREIGHTON\_ENDOCRINE\_THERAPY\_RESISTANCE\_2 |  | 113 | -0.31 | -1.03 | 0.421 | 0.645 | 1.000 | 1555 | tags=35%, list=27%, signal=46% |
| 910 | LEE\_METASTASIS\_AND\_ALTERNATIVE\_SPLICING\_UP |  | 20 | -0.39 | -1.03 | 0.458 | 0.645 | 1.000 | 878 | tags=25%, list=15%, signal=29% |
| 911 | BROWNE\_HCMV\_INFECTION\_10HR\_UP |  | 40 | -0.35 | -1.03 | 0.443 | 0.650 | 1.000 | 888 | tags=23%, list=16%, signal=26% |
| 912 | FONTAINE\_FOLLICULAR\_THYROID\_ADENOMA\_UP |  | 23 | -0.38 | -1.03 | 0.469 | 0.652 | 1.000 | 1136 | tags=30%, list=20%, signal=38% |
| 913 | WEI\_MIR34A\_TARGETS |  | 70 | -0.32 | -1.02 | 0.452 | 0.652 | 1.000 | 1754 | tags=41%, list=31%, signal=59% |
| 914 | BROWNE\_HCMV\_INFECTION\_18HR\_UP |  | 87 | -0.31 | -1.02 | 0.487 | 0.662 | 1.000 | 1472 | tags=32%, list=26%, signal=43% |
| 915 | CHANDRAN\_METASTASIS\_UP |  | 36 | -0.35 | -1.02 | 0.470 | 0.665 | 1.000 | 1023 | tags=22%, list=18%, signal=27% |
| 916 | CROONQUIST\_NRAS\_VS\_STROMAL\_STIMULATION\_UP |  | 19 | -0.40 | -1.02 | 0.459 | 0.664 | 1.000 | 578 | tags=26%, list=10%, signal=29% |
| 917 | STEIN\_ESRRA\_TARGETS |  | 244 | -0.29 | -1.02 | 0.444 | 0.666 | 1.000 | 1715 | tags=33%, list=30%, signal=45% |
| 918 | REACTOME\_TRANSMEMBRANE\_TRANSPORT\_OF\_SMALL\_MOLECULES |  | 60 | -0.33 | -1.02 | 0.445 | 0.666 | 1.000 | 832 | tags=17%, list=15%, signal=19% |
| 919 | REACTOME\_METABOLISM\_OF\_AMINO\_ACIDS |  | 79 | -0.31 | -1.02 | 0.457 | 0.667 | 1.000 | 2054 | tags=37%, list=36%, signal=56% |
| 920 | KEGG\_CARDIAC\_MUSCLE\_CONTRACTION |  | 20 | -0.38 | -1.02 | 0.445 | 0.668 | 1.000 | 1719 | tags=40%, list=30%, signal=57% |
| 921 | KYNG\_DNA\_DAMAGE\_BY\_GAMMA\_AND\_UV\_RADIATION |  | 21 | -0.38 | -1.01 | 0.458 | 0.676 | 1.000 | 1690 | tags=33%, list=30%, signal=47% |
| 922 | REACTOME\_DOWNSTREAM\_TCR\_SIGNALING |  | 22 | -0.38 | -1.01 | 0.464 | 0.676 | 1.000 | 1202 | tags=32%, list=21%, signal=40% |
| 923 | DACOSTA\_UV\_RESPONSE\_VIA\_ERCC3\_UP |  | 136 | -0.30 | -1.01 | 0.482 | 0.679 | 1.000 | 964 | tags=19%, list=17%, signal=22% |
| 924 | STEIN\_ESRRA\_TARGETS\_UP |  | 182 | -0.29 | -1.01 | 0.476 | 0.680 | 1.000 | 1818 | tags=36%, list=32%, signal=51% |
| 925 | REACTOME\_RHO\_GTPASE\_CYCLE |  | 56 | -0.33 | -1.01 | 0.482 | 0.684 | 1.000 | 1071 | tags=23%, list=19%, signal=28% |
| 926 | PARENT\_MTOR\_SIGNALING\_UP |  | 226 | -0.29 | -1.01 | 0.504 | 0.684 | 1.000 | 856 | tags=15%, list=15%, signal=17% |
| 927 | BOYLAN\_MULTIPLE\_MYELOMA\_C\_D\_UP |  | 47 | -0.33 | -1.01 | 0.468 | 0.688 | 1.000 | 770 | tags=19%, list=13%, signal=22% |
| 928 | LINDGREN\_BLADDER\_CANCER\_CLUSTER\_3\_UP |  | 166 | -0.29 | -1.00 | 0.477 | 0.688 | 1.000 | 1207 | tags=22%, list=21%, signal=27% |
| 929 | PELLICCIOTTA\_HDAC\_IN\_ANTIGEN\_PRESENTATION\_DN |  | 28 | -0.36 | -1.00 | 0.476 | 0.689 | 1.000 | 2601 | tags=54%, list=45%, signal=98% |
| 930 | KOKKINAKIS\_METHIONINE\_DEPRIVATION\_48HR\_DN |  | 27 | -0.36 | -1.00 | 0.475 | 0.689 | 1.000 | 1066 | tags=22%, list=19%, signal=27% |
| 931 | IGARASHI\_ATF4\_TARGETS\_DN |  | 41 | -0.34 | -1.00 | 0.486 | 0.689 | 1.000 | 814 | tags=20%, list=14%, signal=23% |
| 932 | LIAO\_METASTASIS |  | 181 | -0.29 | -1.00 | 0.495 | 0.689 | 1.000 | 514 | tags=13%, list=9%, signal=14% |
| 933 | BROWNE\_HCMV\_INFECTION\_8HR\_DN |  | 17 | -0.40 | -1.00 | 0.490 | 0.688 | 1.000 | 430 | tags=18%, list=8%, signal=19% |
| 934 | SHAFFER\_IRF4\_TARGETS\_IN\_ACTIVATED\_B\_LYMPHOCYTE |  | 38 | -0.34 | -1.00 | 0.494 | 0.693 | 1.000 | 1090 | tags=24%, list=19%, signal=29% |
| 935 | NAGASHIMA\_EGF\_SIGNALING\_UP |  | 19 | -0.39 | -1.00 | 0.491 | 0.694 | 1.000 | 718 | tags=21%, list=13%, signal=24% |
| 936 | CHIARETTI\_ACUTE\_LYMPHOBLASTIC\_LEUKEMIA\_ZAP70 |  | 33 | -0.35 | -1.00 | 0.491 | 0.695 | 1.000 | 528 | tags=15%, list=9%, signal=17% |
| 937 | WNT\_SIGNALING |  | 32 | -0.35 | -1.00 | 0.488 | 0.695 | 1.000 | 1687 | tags=38%, list=29%, signal=53% |
| 938 | BIOCARTA\_FAS\_PATHWAY |  | 20 | -0.39 | -1.00 | 0.489 | 0.694 | 1.000 | 1547 | tags=35%, list=27%, signal=48% |
| 939 | BENPORATH\_MYC\_TARGETS\_WITH\_EBOX |  | 82 | -0.31 | -1.00 | 0.504 | 0.694 | 1.000 | 1542 | tags=29%, list=27%, signal=39% |
| 940 | RUGO\_STRESS\_RESPONSE\_SUBSET\_H |  | 21 | -0.38 | -1.00 | 0.492 | 0.695 | 1.000 | 1690 | tags=33%, list=30%, signal=47% |
| 941 | BIOCARTA\_IGF1\_PATHWAY |  | 15 | -0.40 | -1.00 | 0.487 | 0.698 | 1.000 | 1547 | tags=27%, list=27%, signal=36% |
| 942 | CHARAFE\_BREAST\_CANCER\_LUMINAL\_VS\_MESENCHYMAL\_UP |  | 116 | -0.30 | -0.99 | 0.509 | 0.706 | 1.000 | 1429 | tags=31%, list=25%, signal=41% |
| 943 | MCCABE\_BOUND\_BY\_HOXC6 |  | 128 | -0.30 | -0.99 | 0.522 | 0.709 | 1.000 | 1120 | tags=22%, list=20%, signal=27% |
| 944 | VANTVEER\_BREAST\_CANCER\_METASTASIS\_DN |  | 61 | -0.32 | -0.99 | 0.485 | 0.709 | 1.000 | 837 | tags=16%, list=15%, signal=19% |
| 945 | NGUYEN\_NOTCH1\_TARGETS\_DN |  | 34 | -0.34 | -0.99 | 0.506 | 0.709 | 1.000 | 585 | tags=18%, list=10%, signal=20% |
| 946 | LOPEZ\_MBD\_TARGETS |  | 380 | -0.28 | -0.99 | 0.541 | 0.709 | 1.000 | 972 | tags=19%, list=17%, signal=21% |
| 947 | TSAI\_RESPONSE\_TO\_IONIZING\_RADIATION |  | 54 | -0.32 | -0.99 | 0.495 | 0.709 | 1.000 | 930 | tags=26%, list=16%, signal=31% |
| 948 | RASHI\_RESPONSE\_TO\_IONIZING\_RADIATION\_4 |  | 24 | -0.36 | -0.99 | 0.501 | 0.710 | 1.000 | 417 | tags=13%, list=7%, signal=13% |
| 949 | LEE\_METASTASIS\_AND\_ALTERNATIVE\_SPLICING\_DN |  | 19 | -0.38 | -0.99 | 0.499 | 0.713 | 1.000 | 868 | tags=26%, list=15%, signal=31% |
| 950 | BIOCARTA\_AT1R\_PATHWAY |  | 18 | -0.38 | -0.99 | 0.530 | 0.717 | 1.000 | 1547 | tags=33%, list=27%, signal=46% |
| 951 | KEGG\_ADIPOCYTOKINE\_SIGNALING\_PATHWAY |  | 34 | -0.34 | -0.99 | 0.511 | 0.716 | 1.000 | 614 | tags=15%, list=11%, signal=16% |
| 952 | YAO\_TEMPORAL\_RESPONSE\_TO\_PROGESTERONE\_CLUSTER\_13 |  | 90 | -0.30 | -0.98 | 0.544 | 0.720 | 1.000 | 2228 | tags=47%, list=39%, signal=75% |
| 953 | MULLIGHAN\_NPM1\_MUTATED\_SIGNATURE\_2\_UP |  | 64 | -0.31 | -0.98 | 0.524 | 0.721 | 1.000 | 1675 | tags=33%, list=29%, signal=46% |
| 954 | REACTOME\_INORGANIC\_CATION\_ANION\_SLC\_TRANSPORTERS |  | 23 | -0.37 | -0.98 | 0.521 | 0.726 | 1.000 | 520 | tags=17%, list=9%, signal=19% |
| 955 | HOFFMANN\_PRE\_BI\_TO\_LARGE\_PRE\_BII\_LYMPHOCYTE\_DN |  | 23 | -0.36 | -0.98 | 0.495 | 0.727 | 1.000 | 1129 | tags=26%, list=20%, signal=32% |
| 956 | RIZKI\_TUMOR\_INVASIVENESS\_2D\_DN |  | 21 | -0.37 | -0.98 | 0.511 | 0.727 | 1.000 | 1784 | tags=48%, list=31%, signal=69% |
| 957 | RODRIGUES\_THYROID\_CARCINOMA\_POORLY\_DIFFERENTIATED\_UP |  | 333 | -0.27 | -0.98 | 0.581 | 0.732 | 1.000 | 2471 | tags=47%, list=43%, signal=78% |
| 958 | ST\_GA13\_PATHWAY |  | 16 | -0.39 | -0.97 | 0.509 | 0.734 | 1.000 | 2702 | tags=75%, list=47%, signal=142% |
| 959 | WEIGEL\_OXIDATIVE\_STRESS\_BY\_HNE\_AND\_H2O2 |  | 18 | -0.39 | -0.97 | 0.525 | 0.735 | 1.000 | 1771 | tags=50%, list=31%, signal=72% |
| 960 | AMUNDSON\_RESPONSE\_TO\_ARSENITE |  | 86 | -0.30 | -0.97 | 0.530 | 0.734 | 1.000 | 1925 | tags=36%, list=34%, signal=54% |
| 961 | WANG\_HCP\_PROSTATE\_CANCER |  | 27 | -0.35 | -0.97 | 0.521 | 0.735 | 1.000 | 2256 | tags=48%, list=39%, signal=79% |
| 962 | KIM\_MYC\_AMPLIFICATION\_TARGETS\_UP |  | 60 | -0.31 | -0.97 | 0.538 | 0.737 | 1.000 | 1660 | tags=33%, list=29%, signal=46% |
| 963 | OUILLETTE\_CLL\_13Q14\_DELETION\_UP |  | 31 | -0.34 | -0.97 | 0.523 | 0.737 | 1.000 | 470 | tags=10%, list=8%, signal=10% |
| 964 | MOHANKUMAR\_TLX1\_TARGETS\_UP |  | 200 | -0.28 | -0.97 | 0.550 | 0.739 | 1.000 | 1535 | tags=26%, list=27%, signal=34% |
| 965 | GEORGES\_TARGETS\_OF\_MIR192\_AND\_MIR215 |  | 386 | -0.28 | -0.97 | 0.612 | 0.739 | 1.000 | 1472 | tags=28%, list=26%, signal=35% |
| 966 | BREDEMEYER\_RAG\_SIGNALING\_NOT\_VIA\_ATM\_DN |  | 20 | -0.37 | -0.97 | 0.535 | 0.742 | 1.000 | 1139 | tags=25%, list=20%, signal=31% |
| 967 | SANA\_RESPONSE\_TO\_IFNG\_DN |  | 28 | -0.35 | -0.97 | 0.529 | 0.741 | 1.000 | 1534 | tags=39%, list=27%, signal=53% |
| 968 | KEGG\_ANTIGEN\_PROCESSING\_AND\_PRESENTATION |  | 24 | -0.36 | -0.97 | 0.519 | 0.741 | 1.000 | 1443 | tags=42%, list=25%, signal=55% |
| 969 | LI\_LUNG\_CANCER |  | 16 | -0.39 | -0.97 | 0.523 | 0.740 | 1.000 | 951 | tags=31%, list=17%, signal=37% |
| 970 | BIOCARTA\_BIOPEPTIDES\_PATHWAY |  | 20 | -0.37 | -0.97 | 0.515 | 0.742 | 1.000 | 1547 | tags=30%, list=27%, signal=41% |
| 971 | LU\_IL4\_SIGNALING |  | 27 | -0.35 | -0.97 | 0.530 | 0.742 | 1.000 | 1303 | tags=30%, list=23%, signal=38% |
| 972 | MCBRYAN\_PUBERTAL\_BREAST\_6\_7WK\_UP |  | 80 | -0.30 | -0.97 | 0.558 | 0.744 | 1.000 | 818 | tags=20%, list=14%, signal=23% |
| 973 | KEGG\_EPITHELIAL\_CELL\_SIGNALING\_IN\_HELICOBACTER\_PYLORI\_INFECTION |  | 35 | -0.33 | -0.96 | 0.527 | 0.745 | 1.000 | 1927 | tags=49%, list=34%, signal=73% |
| 974 | BHATI\_G2M\_ARREST\_BY\_2METHOXYESTRADIOL\_UP |  | 57 | -0.31 | -0.96 | 0.538 | 0.745 | 1.000 | 2488 | tags=60%, list=43%, signal=104% |
| 975 | BOYAULT\_LIVER\_CANCER\_SUBCLASS\_G23\_UP |  | 29 | -0.34 | -0.96 | 0.528 | 0.746 | 1.000 | 61 | tags=7%, list=1%, signal=7% |
| 976 | BIOCARTA\_PDGF\_PATHWAY |  | 21 | -0.37 | -0.96 | 0.524 | 0.748 | 1.000 | 582 | tags=14%, list=10%, signal=16% |
| 977 | KAYO\_CALORIE\_RESTRICTION\_MUSCLE\_DN |  | 32 | -0.34 | -0.96 | 0.534 | 0.748 | 1.000 | 1522 | tags=38%, list=27%, signal=51% |
| 978 | IVANOVA\_HEMATOPOIESIS\_LATE\_PROGENITOR |  | 69 | -0.30 | -0.96 | 0.559 | 0.747 | 1.000 | 2516 | tags=55%, list=44%, signal=97% |
| 979 | MILI\_PSEUDOPODIA\_CHEMOTAXIS\_UP |  | 32 | -0.34 | -0.96 | 0.540 | 0.748 | 1.000 | 2278 | tags=56%, list=40%, signal=93% |
| 980 | BIOCARTA\_MET\_PATHWAY |  | 24 | -0.35 | -0.96 | 0.525 | 0.752 | 1.000 | 1547 | tags=33%, list=27%, signal=45% |
| 981 | STEIN\_ESR1\_TARGETS |  | 38 | -0.32 | -0.96 | 0.539 | 0.756 | 1.000 | 1534 | tags=26%, list=27%, signal=36% |
| 982 | MIDORIKAWA\_AMPLIFIED\_IN\_LIVER\_CANCER |  | 17 | -0.38 | -0.96 | 0.536 | 0.755 | 1.000 | 1685 | tags=53%, list=29%, signal=75% |
| 983 | REACTOME\_APOPTOSIS |  | 62 | -0.30 | -0.96 | 0.561 | 0.755 | 1.000 | 2043 | tags=35%, list=36%, signal=55% |
| 984 | SANA\_RESPONSE\_TO\_IFNG\_UP |  | 21 | -0.36 | -0.96 | 0.546 | 0.757 | 1.000 | 545 | tags=19%, list=10%, signal=21% |
| 985 | MARKEY\_RB1\_CHRONIC\_LOF\_UP |  | 42 | -0.32 | -0.95 | 0.539 | 0.762 | 1.000 | 718 | tags=21%, list=13%, signal=24% |
| 986 | LEE\_RECENT\_THYMIC\_EMIGRANT |  | 43 | -0.31 | -0.95 | 0.556 | 0.763 | 1.000 | 1740 | tags=37%, list=30%, signal=53% |
| 987 | REN\_ALVEOLAR\_RHABDOMYOSARCOMA\_UP |  | 22 | -0.35 | -0.95 | 0.554 | 0.765 | 1.000 | 595 | tags=23%, list=10%, signal=25% |
| 988 | MORI\_MATURE\_B\_LYMPHOCYTE\_UP |  | 45 | -0.31 | -0.95 | 0.554 | 0.766 | 1.000 | 1471 | tags=31%, list=26%, signal=42% |
| 989 | SCHEIDEREIT\_IKK\_INTERACTING\_PROTEINS |  | 15 | -0.39 | -0.95 | 0.560 | 0.773 | 1.000 | 678 | tags=20%, list=12%, signal=23% |
| 990 | ST\_DIFFERENTIATION\_PATHWAY\_IN\_PC12\_CELLS |  | 20 | -0.36 | -0.95 | 0.561 | 0.773 | 1.000 | 2865 | tags=70%, list=50%, signal=140% |
| 991 | SIG\_CD40PATHWAYMAP |  | 15 | -0.39 | -0.95 | 0.528 | 0.773 | 1.000 | 1016 | tags=20%, list=18%, signal=24% |
| 992 | CHIANG\_LIVER\_CANCER\_SUBCLASS\_PROLIFERATION\_UP |  | 53 | -0.31 | -0.94 | 0.568 | 0.773 | 1.000 | 665 | tags=15%, list=12%, signal=17% |
| 993 | DANG\_MYC\_TARGETS\_DN |  | 22 | -0.35 | -0.94 | 0.558 | 0.775 | 1.000 | 1163 | tags=23%, list=20%, signal=28% |
| 994 | HOSHIDA\_LIVER\_CANCER\_SURVIVAL\_UP |  | 31 | -0.33 | -0.94 | 0.563 | 0.777 | 1.000 | 593 | tags=23%, list=10%, signal=25% |
| 995 | JI\_RESPONSE\_TO\_FSH\_DN |  | 23 | -0.35 | -0.94 | 0.553 | 0.778 | 1.000 | 3009 | tags=65%, list=53%, signal=137% |
| 996 | REACTOME\_HOST\_INTERACTIONS\_OF\_HIV\_FACTORS |  | 73 | -0.30 | -0.94 | 0.605 | 0.778 | 1.000 | 2306 | tags=38%, list=40%, signal=63% |
| 997 | BIOCARTA\_PAR1\_PATHWAY |  | 17 | -0.38 | -0.94 | 0.587 | 0.780 | 1.000 | 1039 | tags=24%, list=18%, signal=29% |
| 998 | WOO\_LIVER\_CANCER\_RECURRENCE\_DN |  | 19 | -0.36 | -0.94 | 0.574 | 0.783 | 1.000 | 1336 | tags=32%, list=23%, signal=41% |
| 999 | LEONARD\_HYPOXIA |  | 16 | -0.38 | -0.94 | 0.562 | 0.788 | 1.000 | 391 | tags=19%, list=7%, signal=20% |
| 1000 | HOFFMANN\_LARGE\_TO\_SMALL\_PRE\_BII\_LYMPHOCYTE\_DN |  | 16 | -0.38 | -0.93 | 0.566 | 0.788 | 1.000 | 1139 | tags=31%, list=20%, signal=39% |
| 1001 | CREIGHTON\_ENDOCRINE\_THERAPY\_RESISTANCE\_1 |  | 204 | -0.27 | -0.93 | 0.658 | 0.789 | 1.000 | 1284 | tags=23%, list=22%, signal=29% |
| 1002 | HSIAO\_HOUSEKEEPING\_GENES |  | 172 | -0.27 | -0.93 | 0.667 | 0.790 | 1.000 | 1790 | tags=30%, list=31%, signal=42% |
| 1003 | LIAO\_HAVE\_SOX4\_BINDING\_SITES |  | 20 | -0.36 | -0.93 | 0.590 | 0.791 | 1.000 | 304 | tags=15%, list=5%, signal=16% |
| 1004 | MITSIADES\_RESPONSE\_TO\_APLIDIN\_UP |  | 192 | -0.27 | -0.93 | 0.676 | 0.793 | 1.000 | 1937 | tags=36%, list=34%, signal=53% |
| 1005 | CHARAFE\_BREAST\_CANCER\_LUMINAL\_VS\_BASAL\_UP |  | 106 | -0.28 | -0.93 | 0.637 | 0.796 | 1.000 | 619 | tags=14%, list=11%, signal=16% |
| 1006 | REACTOME\_METABLISM\_OF\_NUCLEOTIDES |  | 31 | -0.33 | -0.93 | 0.598 | 0.797 | 1.000 | 1066 | tags=26%, list=19%, signal=32% |
| 1007 | SESTO\_RESPONSE\_TO\_UV\_C5 |  | 33 | -0.33 | -0.93 | 0.573 | 0.798 | 1.000 | 1104 | tags=27%, list=19%, signal=34% |
| 1008 | HADDAD\_B\_LYMPHOCYTE\_PROGENITOR |  | 119 | -0.28 | -0.93 | 0.651 | 0.800 | 1.000 | 579 | tags=17%, list=10%, signal=18% |
| 1009 | YAO\_TEMPORAL\_RESPONSE\_TO\_PROGESTERONE\_CLUSTER\_8 |  | 23 | -0.34 | -0.92 | 0.573 | 0.802 | 1.000 | 788 | tags=22%, list=14%, signal=25% |
| 1010 | SUNG\_METASTASIS\_STROMA\_DN |  | 24 | -0.34 | -0.92 | 0.598 | 0.804 | 1.000 | 852 | tags=17%, list=15%, signal=19% |
| 1011 | KEGG\_ENDOCYTOSIS |  | 90 | -0.28 | -0.92 | 0.639 | 0.808 | 1.000 | 1104 | tags=21%, list=19%, signal=26% |
| 1012 | PENG\_RAPAMYCIN\_RESPONSE\_DN |  | 38 | -0.31 | -0.92 | 0.605 | 0.813 | 1.000 | 1770 | tags=32%, list=31%, signal=45% |
| 1013 | BOGNI\_TREATMENT\_RELATED\_MYELOID\_LEUKEMIA\_DN |  | 15 | -0.37 | -0.92 | 0.588 | 0.813 | 1.000 | 1826 | tags=47%, list=32%, signal=68% |
| 1014 | KEGG\_VALINE\_LEUCINE\_AND\_ISOLEUCINE\_DEGRADATION |  | 34 | -0.31 | -0.92 | 0.610 | 0.815 | 1.000 | 2054 | tags=41%, list=36%, signal=64% |
| 1015 | CAIRO\_HEPATOBLASTOMA\_UP |  | 106 | -0.28 | -0.92 | 0.656 | 0.814 | 1.000 | 1175 | tags=23%, list=21%, signal=28% |
| 1016 | NIKOLSKY\_BREAST\_CANCER\_17Q21\_Q25\_AMPLICON |  | 105 | -0.27 | -0.92 | 0.661 | 0.814 | 1.000 | 1521 | tags=26%, list=27%, signal=34% |
| 1017 | FERREIRA\_EWINGS\_SARCOMA\_UNSTABLE\_VS\_STABLE\_DN |  | 28 | -0.32 | -0.92 | 0.604 | 0.813 | 1.000 | 1680 | tags=39%, list=29%, signal=55% |
| 1018 | TAYLOR\_METHYLATED\_IN\_ACUTE\_LYMPHOBLASTIC\_LEUKEMIA |  | 22 | -0.35 | -0.92 | 0.599 | 0.813 | 1.000 | 1171 | tags=32%, list=20%, signal=40% |
| 1019 | MILI\_PSEUDOPODIA |  | 21 | -0.35 | -0.91 | 0.597 | 0.815 | 1.000 | 2192 | tags=57%, list=38%, signal=92% |
| 1020 | REACTOME\_COLLAGEN\_MEDIATED\_ACTIVATION\_CASCADE |  | 17 | -0.36 | -0.91 | 0.600 | 0.814 | 1.000 | 682 | tags=24%, list=12%, signal=27% |
| 1021 | PAL\_PRMT5\_TARGETS\_UP |  | 101 | -0.28 | -0.91 | 0.666 | 0.817 | 1.000 | 586 | tags=12%, list=10%, signal=13% |
| 1022 | BROWNE\_HCMV\_INFECTION\_20HR\_UP |  | 95 | -0.28 | -0.91 | 0.659 | 0.816 | 1.000 | 789 | tags=15%, list=14%, signal=17% |
| 1023 | YAGI\_AML\_RELAPSE\_PROGNOSIS |  | 20 | -0.35 | -0.91 | 0.577 | 0.816 | 1.000 | 2557 | tags=65%, list=45%, signal=117% |
| 1024 | KIM\_WT1\_TARGETS\_8HR\_UP |  | 65 | -0.29 | -0.91 | 0.626 | 0.817 | 1.000 | 1104 | tags=22%, list=19%, signal=26% |
| 1025 | REACTOME\_SLC\_MEDIATED\_TRANSMEMBRANE\_TRANSPORT |  | 38 | -0.31 | -0.91 | 0.622 | 0.818 | 1.000 | 1577 | tags=32%, list=28%, signal=43% |
| 1026 | KIM\_GASTRIC\_CANCER\_CHEMOSENSITIVITY |  | 29 | -0.32 | -0.91 | 0.603 | 0.820 | 1.000 | 1362 | tags=28%, list=24%, signal=36% |
| 1027 | GENTILE\_UV\_RESPONSE\_CLUSTER\_D7 |  | 19 | -0.35 | -0.91 | 0.610 | 0.823 | 1.000 | 1359 | tags=37%, list=24%, signal=48% |
| 1028 | REACTOME\_VIF\_MEDIATED\_DEGRADATION\_OF\_APOBEC3G |  | 30 | -0.32 | -0.91 | 0.623 | 0.825 | 1.000 | 2689 | tags=53%, list=47%, signal=100% |
| 1029 | ACEVEDO\_NORMAL\_TISSUE\_ADJACENT\_TO\_LIVER\_TUMOR\_DN |  | 139 | -0.27 | -0.91 | 0.694 | 0.825 | 1.000 | 1200 | tags=21%, list=21%, signal=26% |
| 1030 | ROME\_INSULIN\_TARGETS\_IN\_MUSCLE\_UP |  | 48 | -0.30 | -0.91 | 0.626 | 0.825 | 1.000 | 1932 | tags=33%, list=34%, signal=50% |
| 1031 | CONCANNON\_APOPTOSIS\_BY\_EPOXOMICIN\_DN |  | 63 | -0.29 | -0.90 | 0.648 | 0.831 | 1.000 | 639 | tags=19%, list=11%, signal=21% |
| 1032 | REACTOME\_ASSOCIATION\_OF\_TRIC\_CCT\_WITH\_TARGET\_PROTEINS\_DURING\_BIOSYNTHESIS |  | 16 | -0.36 | -0.90 | 0.602 | 0.831 | 1.000 | 511 | tags=13%, list=9%, signal=14% |
| 1033 | KEGG\_TOLL\_LIKE\_RECEPTOR\_SIGNALING\_PATHWAY |  | 49 | -0.29 | -0.90 | 0.647 | 0.831 | 1.000 | 1891 | tags=39%, list=33%, signal=57% |
| 1034 | KEGG\_VASOPRESSIN\_REGULATED\_WATER\_REABSORPTION |  | 21 | -0.34 | -0.90 | 0.622 | 0.832 | 1.000 | 2153 | tags=52%, list=38%, signal=84% |
| 1035 | RICKMAN\_TUMOR\_DIFFERENTIATED\_WELL\_VS\_MODERATELY\_UP |  | 52 | -0.29 | -0.90 | 0.636 | 0.831 | 1.000 | 1362 | tags=33%, list=24%, signal=43% |
| 1036 | CHEN\_HOXA5\_TARGETS\_9HR\_UP |  | 115 | -0.27 | -0.90 | 0.678 | 0.833 | 1.000 | 2697 | tags=52%, list=47%, signal=97% |
| 1037 | LASTOWSKA\_NEUROBLASTOMA\_COPY\_NUMBER\_UP |  | 92 | -0.27 | -0.89 | 0.683 | 0.840 | 1.000 | 1240 | tags=22%, list=22%, signal=27% |
| 1038 | KEGG\_VIBRIO\_CHOLERAE\_INFECTION |  | 26 | -0.32 | -0.89 | 0.635 | 0.839 | 1.000 | 2058 | tags=54%, list=36%, signal=84% |
| 1039 | APPIERTO\_RESPONSE\_TO\_FENRETINIDE\_DN |  | 19 | -0.34 | -0.89 | 0.633 | 0.838 | 1.000 | 693 | tags=16%, list=12%, signal=18% |
| 1040 | HOSHIDA\_LIVER\_CANCER\_SUBCLASS\_S2 |  | 64 | -0.28 | -0.89 | 0.668 | 0.838 | 1.000 | 1998 | tags=34%, list=35%, signal=52% |
| 1041 | DEURIG\_T\_CELL\_PROLYMPHOCYTIC\_LEUKEMIA\_UP |  | 119 | -0.27 | -0.89 | 0.710 | 0.839 | 1.000 | 914 | tags=21%, list=16%, signal=24% |
| 1042 | BENPORATH\_NANOG\_TARGETS |  | 449 | -0.25 | -0.89 | 0.842 | 0.844 | 1.000 | 1333 | tags=23%, list=23%, signal=27% |
| 1043 | VERNELL\_RETINOBLASTOMA\_PATHWAY\_UP |  | 25 | -0.32 | -0.89 | 0.638 | 0.844 | 1.000 | 1393 | tags=24%, list=24%, signal=32% |
| 1044 | SMITH\_LIVER\_CANCER |  | 15 | -0.36 | -0.89 | 0.630 | 0.845 | 1.000 | 1232 | tags=33%, list=22%, signal=42% |
| 1045 | DOUGLAS\_BMI1\_TARGETS\_UP |  | 169 | -0.26 | -0.89 | 0.746 | 0.844 | 1.000 | 1376 | tags=24%, list=24%, signal=30% |
| 1046 | TANAKA\_METHYLATED\_IN\_ESOPHAGEAL\_CARCINOMA |  | 24 | -0.32 | -0.89 | 0.635 | 0.845 | 1.000 | 374 | tags=13%, list=7%, signal=13% |
| 1047 | SIG\_CHEMOTAXIS |  | 21 | -0.34 | -0.89 | 0.646 | 0.846 | 1.000 | 1019 | tags=19%, list=18%, signal=23% |
| 1048 | CHANG\_CORE\_SERUM\_RESPONSE\_UP |  | 32 | -0.31 | -0.89 | 0.660 | 0.845 | 1.000 | 579 | tags=16%, list=10%, signal=17% |
| 1049 | KESHELAVA\_MULTIPLE\_DRUG\_RESISTANCE |  | 29 | -0.32 | -0.89 | 0.639 | 0.847 | 1.000 | 1613 | tags=41%, list=28%, signal=57% |
| 1050 | REACTOME\_BRANCHED\_CHAIN\_AMINO\_ACID\_CATABOLISM |  | 16 | -0.36 | -0.89 | 0.635 | 0.848 | 1.000 | 2054 | tags=50%, list=36%, signal=78% |
| 1051 | REACTOME\_REGULATION\_OF\_ORNITHINE\_DECARBOXYLASE |  | 29 | -0.32 | -0.88 | 0.626 | 0.849 | 1.000 | 2573 | tags=48%, list=45%, signal=87% |
| 1052 | FLECHNER\_PBL\_KIDNEY\_TRANSPLANT\_REJECTED\_VS\_OK\_UP |  | 36 | -0.30 | -0.88 | 0.664 | 0.849 | 1.000 | 2003 | tags=44%, list=35%, signal=68% |
| 1053 | MARTINEZ\_TP53\_TARGETS\_DN |  | 184 | -0.25 | -0.88 | 0.756 | 0.849 | 1.000 | 1190 | tags=23%, list=21%, signal=28% |
| 1054 | NAKAYAMA\_SOFT\_TISSUE\_TUMORS\_PCA2\_UP |  | 30 | -0.32 | -0.88 | 0.674 | 0.849 | 1.000 | 787 | tags=20%, list=14%, signal=23% |
| 1055 | CASORELLI\_ACUTE\_PROMYELOCYTIC\_LEUKEMIA\_DN |  | 346 | -0.25 | -0.88 | 0.824 | 0.853 | 1.000 | 661 | tags=12%, list=12%, signal=12% |
| 1056 | LASTOWSKA\_COAMPLIFIED\_WITH\_MYCN |  | 17 | -0.34 | -0.88 | 0.648 | 0.854 | 1.000 | 2668 | tags=59%, list=47%, signal=110% |
| 1057 | KOYAMA\_SEMA3B\_TARGETS\_DN |  | 84 | -0.27 | -0.88 | 0.703 | 0.854 | 1.000 | 1684 | tags=32%, list=29%, signal=45% |
| 1058 | COLDREN\_GEFITINIB\_RESISTANCE\_UP |  | 38 | -0.30 | -0.88 | 0.652 | 0.854 | 1.000 | 1705 | tags=34%, list=30%, signal=48% |
| 1059 | JIANG\_HYPOXIA\_CANCER |  | 25 | -0.32 | -0.88 | 0.638 | 0.853 | 1.000 | 1295 | tags=28%, list=23%, signal=36% |
| 1060 | BIOCARTA\_RHO\_PATHWAY |  | 16 | -0.35 | -0.88 | 0.644 | 0.853 | 1.000 | 261 | tags=13%, list=5%, signal=13% |
| 1061 | BENPORATH\_SOX2\_TARGETS |  | 316 | -0.25 | -0.88 | 0.817 | 0.853 | 1.000 | 1093 | tags=18%, list=19%, signal=21% |
| 1062 | BIOCARTA\_PYK2\_PATHWAY |  | 20 | -0.34 | -0.88 | 0.667 | 0.854 | 1.000 | 1605 | tags=30%, list=28%, signal=42% |
| 1063 | DAIRKEE\_CANCER\_PRONE\_RESPONSE\_BPA\_E2 |  | 50 | -0.28 | -0.88 | 0.680 | 0.854 | 1.000 | 2557 | tags=52%, list=45%, signal=93% |
| 1064 | DACOSTA\_UV\_RESPONSE\_VIA\_ERCC3\_TTD\_DN |  | 39 | -0.30 | -0.87 | 0.663 | 0.856 | 1.000 | 1095 | tags=26%, list=19%, signal=31% |
| 1065 | CONCANNON\_APOPTOSIS\_BY\_EPOXOMICIN\_UP |  | 89 | -0.27 | -0.87 | 0.732 | 0.856 | 1.000 | 1861 | tags=36%, list=33%, signal=52% |
| 1066 | DACOSTA\_UV\_RESPONSE\_VIA\_ERCC3\_COMMON\_UP |  | 22 | -0.32 | -0.87 | 0.652 | 0.856 | 1.000 | 514 | tags=14%, list=9%, signal=15% |
| 1067 | BROWNE\_HCMV\_INFECTION\_14HR\_UP |  | 81 | -0.27 | -0.87 | 0.708 | 0.858 | 1.000 | 1494 | tags=26%, list=26%, signal=35% |
| 1068 | ZHAN\_MULTIPLE\_MYELOMA\_PR\_DN |  | 17 | -0.35 | -0.87 | 0.638 | 0.862 | 1.000 | 814 | tags=24%, list=14%, signal=27% |
| 1069 | YAGI\_AML\_WITH\_11Q23\_REARRANGED |  | 146 | -0.25 | -0.87 | 0.765 | 0.862 | 1.000 | 1494 | tags=29%, list=26%, signal=39% |
| 1070 | JAZAG\_TGFB1\_SIGNALING\_VIA\_SMAD4\_UP |  | 38 | -0.30 | -0.87 | 0.669 | 0.864 | 1.000 | 496 | tags=16%, list=9%, signal=17% |
| 1071 | KEGG\_PROTEASOME |  | 25 | -0.32 | -0.87 | 0.670 | 0.863 | 1.000 | 2977 | tags=56%, list=52%, signal=116% |
| 1072 | KORKOLA\_SEMINOMA\_UP |  | 21 | -0.32 | -0.86 | 0.669 | 0.870 | 1.000 | 385 | tags=10%, list=7%, signal=10% |
| 1073 | BERTUCCI\_MEDULLARY\_VS\_DUCTAL\_BREAST\_CANCER\_UP |  | 92 | -0.26 | -0.86 | 0.735 | 0.870 | 1.000 | 747 | tags=15%, list=13%, signal=17% |
| 1074 | KEGG\_NOD\_LIKE\_RECEPTOR\_SIGNALING\_PATHWAY |  | 22 | -0.32 | -0.86 | 0.650 | 0.870 | 1.000 | 2731 | tags=73%, list=48%, signal=139% |
| 1075 | GOTTWEIN\_TARGETS\_OF\_KSHV\_MIR\_K12\_11 |  | 29 | -0.31 | -0.86 | 0.664 | 0.872 | 1.000 | 1480 | tags=34%, list=26%, signal=46% |
| 1076 | NAGASHIMA\_NRG1\_SIGNALING\_DN |  | 19 | -0.34 | -0.86 | 0.667 | 0.872 | 1.000 | 996 | tags=26%, list=17%, signal=32% |
| 1077 | KEGG\_FC\_GAMMA\_R\_MEDIATED\_PHAGOCYTOSIS |  | 49 | -0.28 | -0.86 | 0.696 | 0.872 | 1.000 | 323 | tags=10%, list=6%, signal=11% |
| 1078 | MULLIGHAN\_MLL\_SIGNATURE\_2\_UP |  | 189 | -0.25 | -0.86 | 0.802 | 0.873 | 1.000 | 1126 | tags=21%, list=20%, signal=25% |
| 1079 | FLECHNER\_PBL\_KIDNEY\_TRANSPLANT\_OK\_VS\_DONOR\_DN |  | 22 | -0.33 | -0.86 | 0.685 | 0.877 | 1.000 | 927 | tags=23%, list=16%, signal=27% |
| 1080 | BACOLOD\_RESISTANCE\_TO\_ALKYLATING\_AGENTS\_DN |  | 24 | -0.32 | -0.86 | 0.679 | 0.876 | 1.000 | 772 | tags=21%, list=13%, signal=24% |
| 1081 | SHIPP\_DLBCL\_VS\_FOLLICULAR\_LYMPHOMA\_UP |  | 19 | -0.33 | -0.85 | 0.668 | 0.877 | 1.000 | 1232 | tags=26%, list=22%, signal=33% |
| 1082 | BREDEMEYER\_RAG\_SIGNALING\_VIA\_ATM\_NOT\_VIA\_NFKB\_UP |  | 21 | -0.32 | -0.85 | 0.689 | 0.878 | 1.000 | 1381 | tags=29%, list=24%, signal=38% |
| 1083 | VANHARANTA\_UTERINE\_FIBROID\_UP |  | 16 | -0.35 | -0.85 | 0.684 | 0.878 | 1.000 | 990 | tags=31%, list=17%, signal=38% |
| 1084 | WAMUNYOKOLI\_OVARIAN\_CANCER\_LMP\_UP |  | 90 | -0.26 | -0.85 | 0.743 | 0.878 | 1.000 | 1749 | tags=34%, list=31%, signal=49% |
| 1085 | GALLUZZI\_PERMEABILIZE\_MITOCHONDRIA |  | 18 | -0.34 | -0.85 | 0.685 | 0.881 | 1.000 | 736 | tags=22%, list=13%, signal=25% |
| 1086 | LUI\_THYROID\_CANCER\_PAX8\_PPARG\_UP |  | 17 | -0.34 | -0.85 | 0.676 | 0.881 | 1.000 | 542 | tags=24%, list=9%, signal=26% |
| 1087 | BIOCARTA\_GH\_PATHWAY |  | 19 | -0.33 | -0.85 | 0.685 | 0.881 | 1.000 | 259 | tags=11%, list=5%, signal=11% |
| 1088 | DIRMEIER\_LMP1\_RESPONSE\_EARLY |  | 23 | -0.31 | -0.85 | 0.714 | 0.885 | 1.000 | 1814 | tags=39%, list=32%, signal=57% |
| 1089 | DING\_LUNG\_CANCER\_EXPRESSION\_BY\_COPY\_NUMBER |  | 59 | -0.27 | -0.85 | 0.743 | 0.885 | 1.000 | 1306 | tags=24%, list=23%, signal=30% |
| 1090 | KEGG\_GNRH\_SIGNALING\_PATHWAY |  | 35 | -0.30 | -0.85 | 0.711 | 0.884 | 1.000 | 582 | tags=17%, list=10%, signal=19% |
| 1091 | ZHAN\_MULTIPLE\_MYELOMA\_CD2\_UP |  | 17 | -0.33 | -0.84 | 0.704 | 0.887 | 1.000 | 671 | tags=24%, list=12%, signal=27% |
| 1092 | BONCI\_TARGETS\_OF\_MIR15A\_AND\_MIR16\_1 |  | 36 | -0.29 | -0.84 | 0.724 | 0.887 | 1.000 | 582 | tags=14%, list=10%, signal=15% |
| 1093 | KONDO\_PROSTATE\_CANCER\_HCP\_WITH\_H3K27ME3 |  | 22 | -0.32 | -0.84 | 0.692 | 0.888 | 1.000 | 705 | tags=23%, list=12%, signal=26% |
| 1094 | REACTOME\_GLUCOSE\_METABOLISM |  | 21 | -0.32 | -0.84 | 0.699 | 0.888 | 1.000 | 2018 | tags=48%, list=35%, signal=73% |
| 1095 | BOYLAN\_MULTIPLE\_MYELOMA\_C\_CLUSTER\_DN |  | 15 | -0.34 | -0.84 | 0.667 | 0.888 | 1.000 | 1120 | tags=27%, list=20%, signal=33% |
| 1096 | RICKMAN\_METASTASIS\_DN |  | 80 | -0.26 | -0.84 | 0.770 | 0.891 | 1.000 | 1313 | tags=23%, list=23%, signal=29% |
| 1097 | ZHOU\_INFLAMMATORY\_RESPONSE\_LIVE\_DN |  | 145 | -0.25 | -0.84 | 0.800 | 0.890 | 1.000 | 1372 | tags=22%, list=24%, signal=28% |
| 1098 | DER\_IFN\_GAMMA\_RESPONSE\_UP |  | 19 | -0.32 | -0.84 | 0.675 | 0.890 | 1.000 | 2017 | tags=47%, list=35%, signal=73% |
| 1099 | DOANE\_RESPONSE\_TO\_ANDROGEN\_UP |  | 62 | -0.27 | -0.84 | 0.743 | 0.890 | 1.000 | 1098 | tags=24%, list=19%, signal=30% |
| 1100 | CHOW\_RASSF1\_TARGETS\_DN |  | 16 | -0.33 | -0.84 | 0.668 | 0.889 | 1.000 | 1777 | tags=38%, list=31%, signal=54% |
| 1101 | UDAYAKUMAR\_MED1\_TARGETS\_UP |  | 65 | -0.26 | -0.84 | 0.763 | 0.890 | 1.000 | 1218 | tags=20%, list=21%, signal=25% |
| 1102 | REACTOME\_TRANSMISSION\_ACROSS\_CHEMICAL\_SYNAPSES |  | 34 | -0.29 | -0.84 | 0.731 | 0.890 | 1.000 | 1097 | tags=21%, list=19%, signal=25% |
| 1103 | ZHOU\_INFLAMMATORY\_RESPONSE\_LPS\_DN |  | 145 | -0.25 | -0.84 | 0.802 | 0.890 | 1.000 | 1372 | tags=22%, list=24%, signal=28% |
| 1104 | KEGG\_HUNTINGTONS\_DISEASE |  | 76 | -0.26 | -0.84 | 0.767 | 0.890 | 1.000 | 2356 | tags=46%, list=41%, signal=77% |
| 1105 | BIOCARTA\_DEATH\_PATHWAY |  | 18 | -0.33 | -0.84 | 0.700 | 0.891 | 1.000 | 2687 | tags=56%, list=47%, signal=104% |
| 1106 | ZHANG\_TARGETS\_OF\_EWSR1\_FLI1\_FUSION |  | 33 | -0.29 | -0.83 | 0.723 | 0.896 | 1.000 | 990 | tags=27%, list=17%, signal=33% |
| 1107 | BENPORATH\_NOS\_TARGETS |  | 77 | -0.26 | -0.83 | 0.782 | 0.896 | 1.000 | 806 | tags=16%, list=14%, signal=18% |
| 1108 | WANG\_CISPLATIN\_RESPONSE\_AND\_XPC\_UP |  | 51 | -0.27 | -0.83 | 0.746 | 0.896 | 1.000 | 975 | tags=22%, list=17%, signal=26% |
| 1109 | REACTOME\_REGULATION\_OF\_LIPID\_METABOLISM\_BY\_PEROXISOME\_PROLIFERATOR\_ACTIVATED\_RECEPTOR\_ALPHA |  | 24 | -0.31 | -0.83 | 0.710 | 0.897 | 1.000 | 618 | tags=13%, list=11%, signal=14% |
| 1110 | SENESE\_HDAC2\_TARGETS\_UP |  | 69 | -0.26 | -0.83 | 0.764 | 0.898 | 1.000 | 894 | tags=19%, list=16%, signal=22% |
| 1111 | HAMAI\_APOPTOSIS\_VIA\_TRAIL\_DN |  | 31 | -0.29 | -0.83 | 0.726 | 0.897 | 1.000 | 618 | tags=19%, list=11%, signal=22% |
| 1112 | RODRIGUES\_THYROID\_CARCINOMA\_ANAPLASTIC\_UP |  | 359 | -0.23 | -0.83 | 0.913 | 0.897 | 1.000 | 2641 | tags=47%, list=46%, signal=81% |
| 1113 | DACOSTA\_UV\_RESPONSE\_VIA\_ERCC3\_XPCS\_DN |  | 43 | -0.28 | -0.83 | 0.739 | 0.896 | 1.000 | 2333 | tags=51%, list=41%, signal=86% |
| 1114 | LEE\_TARGETS\_OF\_PTCH1\_AND\_SUFU\_UP |  | 23 | -0.31 | -0.83 | 0.724 | 0.897 | 1.000 | 526 | tags=17%, list=9%, signal=19% |
| 1115 | BOYAULT\_LIVER\_CANCER\_SUBCLASS\_G123\_DN |  | 17 | -0.33 | -0.83 | 0.722 | 0.897 | 1.000 | 682 | tags=18%, list=12%, signal=20% |
| 1116 | REACTOME\_CDT1\_ASSOCIATION\_WITH\_THE\_CDC6\_ORC\_ORIGIN\_COMPLEX |  | 29 | -0.30 | -0.83 | 0.723 | 0.897 | 1.000 | 2573 | tags=45%, list=45%, signal=81% |
| 1117 | AMIT\_EGF\_RESPONSE\_480\_HELA |  | 46 | -0.27 | -0.83 | 0.737 | 0.896 | 1.000 | 2003 | tags=46%, list=35%, signal=70% |
| 1118 | HOSHIDA\_LIVER\_CANCER\_SURVIVAL\_DN |  | 45 | -0.27 | -0.82 | 0.748 | 0.898 | 1.000 | 1684 | tags=36%, list=29%, signal=50% |
| 1119 | LASTOWSKA\_NEUROBLASTOMA\_COPY\_NUMBER\_DN |  | 369 | -0.23 | -0.82 | 0.914 | 0.900 | 1.000 | 1624 | tags=27%, list=28%, signal=35% |
| 1120 | BENPORATH\_OCT4\_TARGETS |  | 129 | -0.25 | -0.82 | 0.828 | 0.899 | 1.000 | 1325 | tags=24%, list=23%, signal=31% |
| 1121 | BIOCARTA\_CTCF\_PATHWAY |  | 19 | -0.32 | -0.82 | 0.723 | 0.900 | 1.000 | 866 | tags=26%, list=15%, signal=31% |
| 1122 | MARTINEZ\_RB1\_AND\_TP53\_TARGETS\_DN |  | 183 | -0.24 | -0.82 | 0.849 | 0.899 | 1.000 | 1190 | tags=21%, list=21%, signal=26% |
| 1123 | ZHAN\_MULTIPLE\_MYELOMA\_UP |  | 26 | -0.30 | -0.82 | 0.744 | 0.899 | 1.000 | 1622 | tags=38%, list=28%, signal=53% |
| 1124 | DACOSTA\_UV\_RESPONSE\_VIA\_ERCC3\_COMMON\_DN |  | 242 | -0.23 | -0.82 | 0.882 | 0.899 | 1.000 | 1348 | tags=22%, list=24%, signal=28% |
| 1125 | DACOSTA\_UV\_RESPONSE\_VIA\_ERCC3\_DN |  | 484 | -0.23 | -0.82 | 0.950 | 0.899 | 1.000 | 1047 | tags=18%, list=18%, signal=20% |
| 1126 | BROWNE\_HCMV\_INFECTION\_4HR\_DN |  | 96 | -0.25 | -0.82 | 0.820 | 0.898 | 1.000 | 474 | tags=10%, list=8%, signal=11% |
| 1127 | GARGALOVIC\_RESPONSE\_TO\_OXIDIZED\_PHOSPHOLIPIDS\_YELLOW\_UP |  | 15 | -0.33 | -0.82 | 0.705 | 0.899 | 1.000 | 208 | tags=13%, list=4%, signal=14% |
| 1128 | RODWELL\_AGING\_KIDNEY\_NO\_BLOOD\_DN |  | 45 | -0.27 | -0.82 | 0.764 | 0.900 | 1.000 | 2054 | tags=40%, list=36%, signal=62% |
| 1129 | GAZDA\_DIAMOND\_BLACKFAN\_ANEMIA\_PROGENITOR\_DN |  | 38 | -0.28 | -0.82 | 0.730 | 0.901 | 1.000 | 1860 | tags=45%, list=33%, signal=66% |
| 1130 | GRADE\_METASTASIS\_DN |  | 26 | -0.29 | -0.82 | 0.742 | 0.901 | 1.000 | 2026 | tags=46%, list=35%, signal=71% |
| 1131 | REACTOME\_P53\_INDEPENDENT\_DNA\_DAMAGE\_RESPONSE |  | 26 | -0.30 | -0.81 | 0.760 | 0.904 | 1.000 | 2573 | tags=46%, list=45%, signal=83% |
| 1132 | LIN\_NPAS4\_TARGETS\_UP |  | 59 | -0.26 | -0.81 | 0.773 | 0.904 | 1.000 | 1585 | tags=32%, list=28%, signal=44% |
| 1133 | BERNARD\_PPAPDC1B\_TARGETS\_UP |  | 16 | -0.33 | -0.81 | 0.719 | 0.904 | 1.000 | 1165 | tags=31%, list=20%, signal=39% |
| 1134 | GRADE\_COLON\_AND\_RECTAL\_CANCER\_UP |  | 110 | -0.24 | -0.81 | 0.820 | 0.905 | 1.000 | 758 | tags=15%, list=13%, signal=16% |
| 1135 | WANG\_LMO4\_TARGETS\_DN |  | 188 | -0.24 | -0.81 | 0.877 | 0.905 | 1.000 | 1934 | tags=30%, list=34%, signal=44% |
| 1136 | BIOCARTA\_HIVNEF\_PATHWAY |  | 30 | -0.29 | -0.81 | 0.752 | 0.908 | 1.000 | 1547 | tags=33%, list=27%, signal=45% |
| 1137 | REACTOME\_DOWN\_STREAM\_SIGNAL\_TRANSDUCTION |  | 21 | -0.31 | -0.81 | 0.735 | 0.907 | 1.000 | 1746 | tags=29%, list=31%, signal=41% |
| 1138 | SASAKI\_ADULT\_T\_CELL\_LEUKEMIA |  | 63 | -0.26 | -0.81 | 0.800 | 0.909 | 1.000 | 1950 | tags=41%, list=34%, signal=62% |
| 1139 | BASSO\_CD40\_SIGNALING\_UP |  | 37 | -0.27 | -0.81 | 0.769 | 0.909 | 1.000 | 1500 | tags=32%, list=26%, signal=44% |
| 1140 | WALLACE\_PROSTATE\_CANCER\_RACE\_DN |  | 30 | -0.29 | -0.81 | 0.743 | 0.909 | 1.000 | 877 | tags=17%, list=15%, signal=20% |
| 1141 | YAGI\_AML\_SURVIVAL |  | 66 | -0.26 | -0.80 | 0.779 | 0.908 | 1.000 | 513 | tags=11%, list=9%, signal=12% |
| 1142 | BIOCARTA\_MYOSIN\_PATHWAY |  | 16 | -0.32 | -0.80 | 0.730 | 0.911 | 1.000 | 1071 | tags=25%, list=19%, signal=31% |
| 1143 | REACTOME\_SIGNALING\_BY\_WNT |  | 36 | -0.28 | -0.80 | 0.769 | 0.911 | 1.000 | 2289 | tags=39%, list=40%, signal=64% |
| 1144 | BYSTRYKH\_HEMATOPOIESIS\_STEM\_CELL\_AND\_BRAIN\_QTL\_TRANS |  | 76 | -0.25 | -0.80 | 0.810 | 0.912 | 1.000 | 1429 | tags=26%, list=25%, signal=35% |
| 1145 | REACTOME\_AUTODEGRADATION\_OF\_CDH1\_BY\_CDH1\_APC |  | 36 | -0.27 | -0.80 | 0.764 | 0.915 | 1.000 | 2665 | tags=47%, list=47%, signal=88% |
| 1146 | TIEN\_INTESTINE\_PROBIOTICS\_24HR\_UP |  | 295 | -0.23 | -0.80 | 0.932 | 0.917 | 1.000 | 1786 | tags=31%, list=31%, signal=43% |
| 1147 | KEGG\_PANCREATIC\_CANCER |  | 35 | -0.27 | -0.80 | 0.769 | 0.917 | 1.000 | 1553 | tags=26%, list=27%, signal=35% |
| 1148 | ZHONG\_RESPONSE\_TO\_AZACITIDINE\_AND\_TSA\_DN |  | 34 | -0.28 | -0.79 | 0.788 | 0.917 | 1.000 | 2215 | tags=41%, list=39%, signal=67% |
| 1149 | BENPORATH\_PROLIFERATION |  | 79 | -0.25 | -0.79 | 0.827 | 0.917 | 1.000 | 1301 | tags=22%, list=23%, signal=27% |
| 1150 | MOREAUX\_B\_LYMPHOCYTE\_MATURATION\_BY\_TACI\_UP |  | 15 | -0.32 | -0.79 | 0.739 | 0.917 | 1.000 | 1959 | tags=47%, list=34%, signal=71% |
| 1151 | CHESLER\_BRAIN\_HIGHEST\_EXPRESSION |  | 21 | -0.30 | -0.79 | 0.756 | 0.918 | 1.000 | 2076 | tags=48%, list=36%, signal=74% |
| 1152 | NIKOLSKY\_MUTATED\_AND\_AMPLIFIED\_IN\_BREAST\_CANCER |  | 24 | -0.30 | -0.79 | 0.775 | 0.921 | 1.000 | 1111 | tags=25%, list=19%, signal=31% |
| 1153 | MULLIGHAN\_NPM1\_MUTATED\_SIGNATURE\_2\_DN |  | 22 | -0.29 | -0.79 | 0.759 | 0.922 | 1.000 | 427 | tags=9%, list=7%, signal=10% |
| 1154 | YU\_MYC\_TARGETS\_DN |  | 23 | -0.29 | -0.79 | 0.773 | 0.923 | 1.000 | 730 | tags=17%, list=13%, signal=20% |
| 1155 | WEIGEL\_OXIDATIVE\_STRESS\_RESPONSE |  | 17 | -0.31 | -0.79 | 0.773 | 0.922 | 1.000 | 937 | tags=24%, list=16%, signal=28% |
| 1156 | ST\_ERK1\_ERK2\_MAPK\_PATHWAY |  | 15 | -0.32 | -0.79 | 0.750 | 0.923 | 1.000 | 2811 | tags=67%, list=49%, signal=131% |
| 1157 | HUANG\_DASATINIB\_RESISTANCE\_DN |  | 20 | -0.30 | -0.79 | 0.763 | 0.923 | 1.000 | 552 | tags=15%, list=10%, signal=17% |
| 1158 | BIOCARTA\_GLEEVEC\_PATHWAY |  | 18 | -0.31 | -0.79 | 0.769 | 0.922 | 1.000 | 1547 | tags=28%, list=27%, signal=38% |
| 1159 | RUIZ\_TNC\_TARGETS\_DN |  | 69 | -0.24 | -0.78 | 0.818 | 0.924 | 1.000 | 433 | tags=10%, list=8%, signal=11% |
| 1160 | NAKAMURA\_CANCER\_MICROENVIRONMENT\_DN |  | 23 | -0.29 | -0.78 | 0.780 | 0.930 | 1.000 | 490 | tags=9%, list=9%, signal=9% |
| 1161 | CHOI\_ATL\_STAGE\_PREDICTOR |  | 15 | -0.32 | -0.78 | 0.756 | 0.930 | 1.000 | 75 | tags=7%, list=1%, signal=7% |
| 1162 | SOTIRIOU\_BREAST\_CANCER\_GRADE\_1\_VS\_3\_DN |  | 19 | -0.30 | -0.78 | 0.748 | 0.930 | 1.000 | 611 | tags=16%, list=11%, signal=18% |
| 1163 | BIOCARTA\_TNFR1\_PATHWAY |  | 16 | -0.31 | -0.78 | 0.772 | 0.931 | 1.000 | 1547 | tags=25%, list=27%, signal=34% |
| 1164 | BIOCARTA\_INSULIN\_PATHWAY |  | 15 | -0.31 | -0.77 | 0.773 | 0.932 | 1.000 | 1547 | tags=20%, list=27%, signal=27% |
| 1165 | REACTOME\_REGULATION\_OF\_INSULIN\_SECRETION |  | 85 | -0.23 | -0.77 | 0.856 | 0.931 | 1.000 | 1999 | tags=35%, list=35%, signal=53% |
| 1166 | ZHAN\_MULTIPLE\_MYELOMA\_CD1\_AND\_CD2\_UP |  | 31 | -0.27 | -0.77 | 0.790 | 0.931 | 1.000 | 2119 | tags=52%, list=37%, signal=82% |
| 1167 | BASSO\_B\_LYMPHOCYTE\_NETWORK |  | 82 | -0.24 | -0.77 | 0.850 | 0.935 | 1.000 | 1367 | tags=20%, list=24%, signal=25% |
| 1168 | REACTOME\_NEURORANSMITTER\_RECEPTOR\_BINDING\_AND\_DOWNSTREAM\_TRANSMISSION\_IN\_THE\_POSTSYNAPTIC\_CELL |  | 26 | -0.28 | -0.77 | 0.792 | 0.935 | 1.000 | 1070 | tags=19%, list=19%, signal=24% |
| 1169 | KEGG\_BLADDER\_CANCER |  | 18 | -0.30 | -0.77 | 0.789 | 0.934 | 1.000 | 2079 | tags=50%, list=36%, signal=78% |
| 1170 | REACTOME\_HIV\_INFECTION |  | 106 | -0.23 | -0.77 | 0.870 | 0.934 | 1.000 | 2202 | tags=32%, list=38%, signal=51% |
| 1171 | KANNAN\_TP53\_TARGETS\_UP |  | 17 | -0.30 | -0.77 | 0.792 | 0.937 | 1.000 | 722 | tags=12%, list=13%, signal=13% |
| 1172 | ZHU\_CMV\_ALL\_UP |  | 25 | -0.28 | -0.77 | 0.770 | 0.937 | 1.000 | 1875 | tags=40%, list=33%, signal=59% |
| 1173 | AGUIRRE\_PANCREATIC\_CANCER\_COPY\_NUMBER\_UP |  | 134 | -0.23 | -0.77 | 0.901 | 0.936 | 1.000 | 1078 | tags=16%, list=19%, signal=19% |
| 1174 | MARTORIATI\_MDM4\_TARGETS\_NEUROEPITHELIUM\_UP |  | 33 | -0.27 | -0.76 | 0.812 | 0.938 | 1.000 | 437 | tags=12%, list=8%, signal=13% |
| 1175 | REACTOME\_INTEGRATION\_OF\_ENERGY\_METABOLISM |  | 102 | -0.23 | -0.76 | 0.888 | 0.945 | 1.000 | 1820 | tags=29%, list=32%, signal=42% |
| 1176 | BROWNE\_INTERFERON\_RESPONSIVE\_GENES |  | 24 | -0.28 | -0.76 | 0.794 | 0.944 | 1.000 | 728 | tags=17%, list=13%, signal=19% |
| 1177 | BROWNE\_HCMV\_INFECTION\_24HR\_UP |  | 60 | -0.24 | -0.76 | 0.842 | 0.945 | 1.000 | 888 | tags=15%, list=16%, signal=18% |
| 1178 | CHESLER\_BRAIN\_QTL\_CIS |  | 34 | -0.26 | -0.75 | 0.862 | 0.952 | 1.000 | 1630 | tags=35%, list=28%, signal=49% |
| 1179 | REACTOME\_CHAPERONIN\_MEDIATED\_PROTEIN\_FOLDING |  | 19 | -0.29 | -0.74 | 0.802 | 0.957 | 1.000 | 511 | tags=11%, list=9%, signal=12% |
| 1180 | REACTOME\_METABOLISM\_OF\_VITAMINS\_AND\_COFACTORS |  | 18 | -0.29 | -0.74 | 0.805 | 0.956 | 1.000 | 565 | tags=11%, list=10%, signal=12% |
| 1181 | REACTOME\_INNATE\_IMMUNITY\_SIGNALING |  | 54 | -0.24 | -0.74 | 0.866 | 0.956 | 1.000 | 1914 | tags=33%, list=33%, signal=50% |
| 1182 | LIU\_TARGETS\_OF\_VMYB\_VS\_CMYB\_DN |  | 18 | -0.29 | -0.74 | 0.821 | 0.960 | 1.000 | 2229 | tags=50%, list=39%, signal=82% |
| 1183 | WONG\_PROTEASOME\_GENE\_MODULE |  | 30 | -0.26 | -0.74 | 0.851 | 0.962 | 1.000 | 260 | tags=7%, list=5%, signal=7% |
| 1184 | RHODES\_CANCER\_META\_SIGNATURE |  | 29 | -0.26 | -0.74 | 0.837 | 0.963 | 1.000 | 524 | tags=14%, list=9%, signal=15% |
| 1185 | REACTOME\_SIGNALING\_BY\_EGFR |  | 27 | -0.26 | -0.74 | 0.819 | 0.963 | 1.000 | 3016 | tags=63%, list=53%, signal=132% |
| 1186 | KEGG\_ALDOSTERONE\_REGULATED\_SODIUM\_REABSORPTION |  | 18 | -0.29 | -0.73 | 0.833 | 0.965 | 1.000 | 1449 | tags=22%, list=25%, signal=30% |
| 1187 | REACTOME\_SCF\_BETA\_TRCP\_MEDIATED\_DEGRADATION\_OF\_EMI1 |  | 28 | -0.26 | -0.73 | 0.826 | 0.967 | 1.000 | 2573 | tags=43%, list=45%, signal=77% |
| 1188 | BOYAULT\_LIVER\_CANCER\_SUBCLASS\_G12\_UP |  | 16 | -0.29 | -0.73 | 0.826 | 0.967 | 1.000 | 802 | tags=13%, list=14%, signal=14% |
| 1189 | TURASHVILI\_BREAST\_NORMAL\_DUCTAL\_VS\_LOBULAR\_UP |  | 25 | -0.27 | -0.73 | 0.837 | 0.966 | 1.000 | 2221 | tags=40%, list=39%, signal=65% |
| 1190 | REACTOME\_SIGNALLING\_TO\_ERKS |  | 17 | -0.29 | -0.73 | 0.851 | 0.968 | 1.000 | 2176 | tags=41%, list=38%, signal=66% |
| 1191 | REACTOME\_MAPK\_TARGETS\_NUCLEAR\_EVENTS\_MEDIATED\_BY\_MAP\_KINASES |  | 18 | -0.28 | -0.73 | 0.824 | 0.967 | 1.000 | 2811 | tags=56%, list=49%, signal=109% |
| 1192 | MOOTHA\_HUMAN\_MITODB\_6\_2002 |  | 206 | -0.21 | -0.73 | 0.961 | 0.968 | 1.000 | 2086 | tags=35%, list=36%, signal=54% |
| 1193 | GRADE\_COLON\_VS\_RECTAL\_CANCER\_DN |  | 15 | -0.30 | -0.72 | 0.833 | 0.969 | 1.000 | 1602 | tags=47%, list=28%, signal=65% |
| 1194 | SLEBOS\_HEAD\_AND\_NECK\_CANCER\_WITH\_HPV\_UP |  | 31 | -0.25 | -0.72 | 0.839 | 0.973 | 1.000 | 2880 | tags=58%, list=50%, signal=116% |
| 1195 | ENK\_UV\_RESPONSE\_EPIDERMIS\_UP |  | 121 | -0.21 | -0.72 | 0.922 | 0.973 | 1.000 | 856 | tags=12%, list=15%, signal=14% |
| 1196 | MASSARWEH\_RESPONSE\_TO\_ESTRADIOL |  | 18 | -0.28 | -0.72 | 0.845 | 0.973 | 1.000 | 1534 | tags=44%, list=27%, signal=61% |
| 1197 | BROWNE\_HCMV\_INFECTION\_30MIN\_DN |  | 45 | -0.24 | -0.71 | 0.870 | 0.976 | 1.000 | 2018 | tags=38%, list=35%, signal=58% |
| 1198 | BIOCARTA\_ERK\_PATHWAY |  | 15 | -0.29 | -0.71 | 0.846 | 0.976 | 1.000 | 1305 | tags=27%, list=23%, signal=34% |
| 1199 | REACTOME\_GLUCOSE\_REGULATION\_OF\_INSULIN\_SECRETION |  | 69 | -0.22 | -0.71 | 0.904 | 0.980 | 1.000 | 2499 | tags=45%, list=44%, signal=79% |
| 1200 | KEGG\_RENAL\_CELL\_CARCINOMA |  | 44 | -0.23 | -0.71 | 0.889 | 0.980 | 1.000 | 2996 | tags=55%, list=52%, signal=114% |
| 1201 | LIU\_NASOPHARYNGEAL\_CARCINOMA |  | 29 | -0.25 | -0.71 | 0.849 | 0.980 | 1.000 | 836 | tags=17%, list=15%, signal=20% |
| 1202 | REACTOME\_POST\_TRANSLATIONAL\_PROTEIN\_MODIFICATION |  | 17 | -0.28 | -0.71 | 0.848 | 0.980 | 1.000 | 1939 | tags=35%, list=34%, signal=53% |
| 1203 | MULLIGHAN\_MLL\_SIGNATURE\_1\_UP |  | 179 | -0.20 | -0.70 | 0.961 | 0.981 | 1.000 | 1405 | tags=23%, list=25%, signal=29% |
| 1204 | YAGI\_AML\_WITH\_T\_9\_11\_TRANSLOCATION |  | 59 | -0.23 | -0.70 | 0.891 | 0.982 | 1.000 | 1403 | tags=24%, list=25%, signal=31% |
| 1205 | GAL\_LEUKEMIC\_STEM\_CELL\_UP |  | 51 | -0.23 | -0.70 | 0.890 | 0.983 | 1.000 | 2124 | tags=39%, list=37%, signal=62% |
| 1206 | KEGG\_LEISHMANIA\_INFECTION |  | 28 | -0.25 | -0.70 | 0.883 | 0.984 | 1.000 | 1808 | tags=36%, list=32%, signal=52% |
| 1207 | LY\_AGING\_OLD\_DN |  | 26 | -0.25 | -0.70 | 0.869 | 0.985 | 1.000 | 868 | tags=15%, list=15%, signal=18% |
| 1208 | KENNY\_CTNNB1\_TARGETS\_UP |  | 23 | -0.26 | -0.70 | 0.871 | 0.984 | 1.000 | 2594 | tags=48%, list=45%, signal=87% |
| 1209 | BHATTACHARYA\_EMBRYONIC\_STEM\_CELL |  | 22 | -0.26 | -0.70 | 0.863 | 0.985 | 1.000 | 419 | tags=9%, list=7%, signal=10% |
| 1210 | TCGA\_GLIOBLASTOMA\_COPY\_NUMBER\_UP |  | 38 | -0.23 | -0.70 | 0.884 | 0.984 | 1.000 | 1834 | tags=29%, list=32%, signal=42% |
| 1211 | GRAHAM\_NORMAL\_QUIESCENT\_VS\_NORMAL\_DIVIDING\_DN |  | 47 | -0.23 | -0.69 | 0.893 | 0.985 | 1.000 | 5 | tags=2%, list=0%, signal=2% |
| 1212 | XU\_CREBBP\_TARGETS\_DN |  | 19 | -0.26 | -0.69 | 0.869 | 0.985 | 1.000 | 637 | tags=16%, list=11%, signal=18% |
| 1213 | BIOCARTA\_TPO\_PATHWAY |  | 17 | -0.27 | -0.69 | 0.870 | 0.984 | 1.000 | 1421 | tags=18%, list=25%, signal=23% |
| 1214 | LE\_EGR2\_TARGETS\_UP |  | 48 | -0.23 | -0.69 | 0.893 | 0.984 | 1.000 | 990 | tags=15%, list=17%, signal=17% |
| 1215 | NIKOLSKY\_BREAST\_CANCER\_8Q23\_Q24\_AMPLICON |  | 47 | -0.23 | -0.69 | 0.901 | 0.983 | 1.000 | 806 | tags=11%, list=14%, signal=12% |
| 1216 | KEGG\_PROGESTERONE\_MEDIATED\_OOCYTE\_MATURATION |  | 42 | -0.23 | -0.69 | 0.890 | 0.986 | 1.000 | 1547 | tags=19%, list=27%, signal=26% |
| 1217 | FLECHNER\_PBL\_KIDNEY\_TRANSPLANT\_OK\_VS\_DONOR\_UP |  | 71 | -0.21 | -0.69 | 0.932 | 0.987 | 1.000 | 1919 | tags=31%, list=34%, signal=46% |
| 1218 | OSMAN\_BLADDER\_CANCER\_DN |  | 158 | -0.20 | -0.69 | 0.972 | 0.986 | 1.000 | 737 | tags=11%, list=13%, signal=12% |
| 1219 | REACTOME\_NRAGE\_SIGNALS\_DEATH\_THROUGH\_JNK |  | 20 | -0.26 | -0.68 | 0.864 | 0.988 | 1.000 | 611 | tags=10%, list=11%, signal=11% |
| 1220 | JIANG\_VHL\_TARGETS |  | 51 | -0.22 | -0.68 | 0.903 | 0.988 | 1.000 | 1861 | tags=39%, list=33%, signal=58% |
| 1221 | RODWELL\_AGING\_KIDNEY\_DN |  | 41 | -0.23 | -0.68 | 0.909 | 0.988 | 1.000 | 1563 | tags=27%, list=27%, signal=37% |
| 1222 | BREDEMEYER\_RAG\_SIGNALING\_NOT\_VIA\_ATM\_UP |  | 27 | -0.25 | -0.68 | 0.897 | 0.989 | 1.000 | 1782 | tags=41%, list=31%, signal=59% |
| 1223 | BREDEMEYER\_RAG\_SIGNALING\_VIA\_ATM\_NOT\_VIA\_NFKB\_DN |  | 18 | -0.26 | -0.68 | 0.867 | 0.988 | 1.000 | 1893 | tags=50%, list=33%, signal=74% |
| 1224 | KORKOLA\_EMBRYONAL\_CARCINOMA\_UP |  | 23 | -0.25 | -0.68 | 0.880 | 0.991 | 1.000 | 1655 | tags=30%, list=29%, signal=43% |
| 1225 | NOUZOVA\_TRETINOIN\_AND\_H4\_ACETYLATION |  | 66 | -0.21 | -0.67 | 0.930 | 0.990 | 1.000 | 2171 | tags=38%, list=38%, signal=60% |
| 1226 | YOSHIOKA\_LIVER\_CANCER\_EARLY\_RECURRENCE\_DN |  | 17 | -0.26 | -0.67 | 0.860 | 0.990 | 1.000 | 2073 | tags=47%, list=36%, signal=74% |
| 1227 | REACTOME\_CELL\_DEATH\_SIGNALLING\_VIA\_NRAGE\_NRIF\_AND\_NADE |  | 25 | -0.24 | -0.67 | 0.896 | 0.991 | 1.000 | 725 | tags=12%, list=13%, signal=14% |
| 1228 | ZHU\_CMV\_24\_HR\_UP |  | 17 | -0.26 | -0.67 | 0.875 | 0.992 | 1.000 | 845 | tags=18%, list=15%, signal=21% |
| 1229 | MATTIOLI\_MGUS\_VS\_PCL |  | 56 | -0.22 | -0.67 | 0.934 | 0.993 | 1.000 | 647 | tags=11%, list=11%, signal=12% |
| 1230 | KEGG\_GLIOMA |  | 32 | -0.23 | -0.66 | 0.917 | 0.994 | 1.000 | 443 | tags=9%, list=8%, signal=10% |
| 1231 | ONDER\_CDH1\_TARGETS\_1\_DN |  | 71 | -0.21 | -0.66 | 0.946 | 0.994 | 1.000 | 1028 | tags=14%, list=18%, signal=17% |
| 1232 | HORIUCHI\_WTAP\_TARGETS\_DN |  | 151 | -0.19 | -0.66 | 0.974 | 0.994 | 1.000 | 1754 | tags=27%, list=31%, signal=38% |
| 1233 | KEGG\_COLORECTAL\_CANCER |  | 36 | -0.23 | -0.66 | 0.918 | 0.995 | 1.000 | 990 | tags=14%, list=17%, signal=17% |
| 1234 | ASTIER\_INTEGRIN\_SIGNALING |  | 23 | -0.24 | -0.66 | 0.911 | 0.995 | 1.000 | 1059 | tags=22%, list=19%, signal=27% |
| 1235 | BASAKI\_YBX1\_TARGETS\_UP |  | 131 | -0.20 | -0.66 | 0.967 | 0.995 | 1.000 | 1281 | tags=21%, list=22%, signal=26% |
| 1236 | REACTOME\_STABILIZATION\_OF\_P53 |  | 30 | -0.24 | -0.65 | 0.918 | 0.998 | 1.000 | 2573 | tags=47%, list=45%, signal=84% |
| 1237 | FUJII\_YBX1\_TARGETS\_DN |  | 74 | -0.20 | -0.65 | 0.960 | 0.998 | 1.000 | 833 | tags=11%, list=15%, signal=12% |
| 1238 | DANG\_MYC\_TARGETS\_UP |  | 48 | -0.21 | -0.65 | 0.952 | 1.000 | 1.000 | 103 | tags=4%, list=2%, signal=4% |
| 1239 | FAELT\_B\_CLL\_WITH\_VH\_REARRANGEMENTS\_DN |  | 25 | -0.24 | -0.64 | 0.917 | 1.000 | 1.000 | 1093 | tags=20%, list=19%, signal=25% |
| 1240 | GRAHAM\_CML\_DIVIDING\_VS\_NORMAL\_QUIESCENT\_UP |  | 89 | -0.20 | -0.64 | 0.966 | 1.000 | 1.000 | 295 | tags=4%, list=5%, signal=5% |
| 1241 | SCHLOSSER\_MYC\_TARGETS\_REPRESSED\_BY\_SERUM |  | 100 | -0.19 | -0.64 | 0.970 | 1.000 | 1.000 | 2829 | tags=55%, list=49%, signal=107% |
| 1242 | BIOCARTA\_RACCYCD\_PATHWAY |  | 16 | -0.25 | -0.64 | 0.902 | 1.000 | 1.000 | 1019 | tags=19%, list=18%, signal=23% |
| 1243 | BIOCARTA\_IL1R\_PATHWAY |  | 16 | -0.25 | -0.63 | 0.910 | 1.000 | 1.000 | 957 | tags=19%, list=17%, signal=22% |
| 1244 | BUYTAERT\_PHOTODYNAMIC\_THERAPY\_STRESS\_DN |  | 256 | -0.18 | -0.63 | 0.994 | 1.000 | 1.000 | 1704 | tags=26%, list=30%, signal=36% |
| 1245 | HEIDENBLAD\_AMPLICON\_8Q24\_DN |  | 15 | -0.26 | -0.63 | 0.898 | 1.000 | 1.000 | 2165 | tags=40%, list=38%, signal=64% |
| 1246 | BERENJENO\_TRANSFORMED\_BY\_RHOA\_UP |  | 262 | -0.18 | -0.63 | 0.994 | 1.000 | 1.000 | 871 | tags=13%, list=15%, signal=15% |
| 1247 | HOEBEKE\_LYMPHOID\_STEM\_CELL\_UP |  | 42 | -0.21 | -0.63 | 0.946 | 1.000 | 1.000 | 1139 | tags=17%, list=20%, signal=21% |
| 1248 | SIG\_INSULIN\_RECEPTOR\_PATHWAY\_IN\_CARDIAC\_MYOCYTES |  | 23 | -0.23 | -0.63 | 0.912 | 1.000 | 1.000 | 132 | tags=4%, list=2%, signal=4% |
| 1249 | NIKOLSKY\_BREAST\_CANCER\_12Q13\_Q21\_AMPLICON |  | 22 | -0.24 | -0.62 | 0.924 | 1.000 | 1.000 | 2176 | tags=45%, list=38%, signal=73% |
| 1250 | WATANABE\_RECTAL\_CANCER\_RADIOTHERAPY\_RESPONSIVE\_UP |  | 48 | -0.21 | -0.62 | 0.950 | 1.000 | 1.000 | 2084 | tags=35%, list=36%, signal=55% |
| 1251 | ST\_JNK\_MAPK\_PATHWAY |  | 17 | -0.24 | -0.62 | 0.925 | 1.000 | 1.000 | 582 | tags=12%, list=10%, signal=13% |
| 1252 | NUYTTEN\_EZH2\_TARGETS\_DN |  | 413 | -0.17 | -0.62 | 0.998 | 1.000 | 1.000 | 1402 | tags=21%, list=24%, signal=25% |
| 1253 | KEGG\_UBIQUITIN\_MEDIATED\_PROTEOLYSIS |  | 73 | -0.19 | -0.62 | 0.971 | 1.000 | 1.000 | 2736 | tags=49%, list=48%, signal=93% |
| 1254 | REACTOME\_STEROID\_METABOLISM |  | 22 | -0.23 | -0.61 | 0.935 | 1.000 | 1.000 | 590 | tags=14%, list=10%, signal=15% |
| 1255 | HAHTOLA\_MYCOSIS\_FUNGOIDES\_CD4\_DN |  | 69 | -0.19 | -0.61 | 0.973 | 1.000 | 1.000 | 1622 | tags=29%, list=28%, signal=40% |
| 1256 | GARY\_CD5\_TARGETS\_DN |  | 250 | -0.17 | -0.60 | 0.999 | 1.000 | 1.000 | 2453 | tags=42%, list=43%, signal=70% |
| 1257 | JAERVINEN\_AMPLIFIED\_IN\_LARYNGEAL\_CANCER |  | 17 | -0.24 | -0.60 | 0.936 | 1.000 | 1.000 | 1367 | tags=24%, list=24%, signal=31% |
| 1258 | SCHLOSSER\_SERUM\_RESPONSE\_AUGMENTED\_BY\_MYC |  | 54 | -0.20 | -0.60 | 0.963 | 1.000 | 1.000 | 1023 | tags=13%, list=18%, signal=16% |
| 1259 | BROWNE\_HCMV\_INFECTION\_16HR\_UP |  | 123 | -0.18 | -0.60 | 0.983 | 1.000 | 1.000 | 454 | tags=7%, list=8%, signal=8% |
| 1260 | REACTOME\_NEP\_NS2\_INTERACTS\_WITH\_THE\_CELLULAR\_EXPORT\_MACHINERY |  | 17 | -0.24 | -0.60 | 0.935 | 1.000 | 1.000 | 2632 | tags=41%, list=46%, signal=76% |
| 1261 | KEGG\_FATTY\_ACID\_METABOLISM |  | 22 | -0.23 | -0.60 | 0.933 | 1.000 | 1.000 | 1510 | tags=23%, list=26%, signal=31% |
| 1262 | MOREAUX\_MULTIPLE\_MYELOMA\_BY\_TACI\_DN |  | 79 | -0.18 | -0.60 | 0.980 | 1.000 | 1.000 | 2278 | tags=34%, list=40%, signal=56% |
| 1263 | BIOCARTA\_CERAMIDE\_PATHWAY |  | 16 | -0.24 | -0.60 | 0.930 | 1.000 | 1.000 | 3038 | tags=63%, list=53%, signal=133% |
| 1264 | BENPORATH\_CYCLING\_GENES |  | 306 | -0.17 | -0.59 | 0.999 | 1.000 | 1.000 | 1580 | tags=22%, list=28%, signal=29% |
| 1265 | FLECHNER\_PBL\_KIDNEY\_TRANSPLANT\_REJECTED\_VS\_OK\_DN |  | 26 | -0.22 | -0.59 | 0.954 | 1.000 | 1.000 | 1730 | tags=23%, list=30%, signal=33% |
| 1266 | BIOCARTA\_NFAT\_PATHWAY |  | 19 | -0.22 | -0.59 | 0.932 | 1.000 | 1.000 | 2974 | tags=63%, list=52%, signal=131% |
| 1267 | KEGG\_APOPTOSIS |  | 40 | -0.20 | -0.59 | 0.959 | 1.000 | 1.000 | 1282 | tags=18%, list=22%, signal=22% |
| 1268 | REACTOME\_TRAF6\_MEDIATED\_INDUCTION\_OF\_THE\_ANTIVIRAL\_CYTOKINE\_IFN\_ALPHA\_BETA\_CASCADE |  | 29 | -0.21 | -0.58 | 0.960 | 1.000 | 1.000 | 2811 | tags=59%, list=49%, signal=115% |
| 1269 | KRIGE\_RESPONSE\_TO\_TOSEDOSTAT\_6HR\_DN |  | 394 | -0.16 | -0.58 | 0.999 | 1.000 | 1.000 | 1192 | tags=16%, list=21%, signal=19% |
| 1270 | MCBRYAN\_PUBERTAL\_BREAST\_5\_6WK\_DN |  | 59 | -0.19 | -0.58 | 0.982 | 1.000 | 1.000 | 1175 | tags=17%, list=21%, signal=21% |
| 1271 | YAO\_TEMPORAL\_RESPONSE\_TO\_PROGESTERONE\_CLUSTER\_14 |  | 74 | -0.18 | -0.58 | 0.984 | 1.000 | 1.000 | 1632 | tags=26%, list=29%, signal=35% |
| 1272 | CHENG\_IMPRINTED\_BY\_ESTRADIOL |  | 38 | -0.20 | -0.58 | 0.957 | 1.000 | 1.000 | 150 | tags=5%, list=3%, signal=5% |
| 1273 | BENPORATH\_MYC\_MAX\_TARGETS |  | 424 | -0.16 | -0.58 | 1.000 | 1.000 | 1.000 | 2123 | tags=34%, list=37%, signal=51% |
| 1274 | ZHANG\_BREAST\_CANCER\_PROGENITORS\_UP |  | 216 | -0.17 | -0.58 | 0.994 | 1.000 | 1.000 | 2635 | tags=46%, list=46%, signal=82% |
| 1275 | AMUNDSON\_GENOTOXIC\_SIGNATURE |  | 37 | -0.20 | -0.58 | 0.967 | 1.000 | 1.000 | 1903 | tags=38%, list=33%, signal=56% |
| 1276 | DER\_IFN\_ALPHA\_RESPONSE\_UP |  | 22 | -0.22 | -0.58 | 0.957 | 1.000 | 1.000 | 2094 | tags=41%, list=37%, signal=64% |
| 1277 | DER\_IFN\_BETA\_RESPONSE\_UP |  | 38 | -0.20 | -0.57 | 0.964 | 1.000 | 1.000 | 2412 | tags=45%, list=42%, signal=77% |
| 1278 | ALCALA\_APOPTOSIS |  | 38 | -0.20 | -0.57 | 0.964 | 1.000 | 1.000 | 1521 | tags=21%, list=27%, signal=28% |
| 1279 | REACTOME\_VIRAL\_MRNA\_TRANSLATION |  | 19 | -0.22 | -0.57 | 0.937 | 1.000 | 1.000 | 4454 | tags=100%, list=78%, signal=449% |
| 1280 | LIU\_SOX4\_TARGETS\_DN |  | 155 | -0.16 | -0.57 | 0.999 | 1.000 | 1.000 | 922 | tags=12%, list=16%, signal=14% |
| 1281 | REACTOME\_REGULATION\_OF\_GLUCOKINASE\_BY\_GLUCOKINASE\_REGULATORY\_PROTEIN |  | 16 | -0.23 | -0.56 | 0.952 | 1.000 | 1.000 | 368 | tags=6%, list=6%, signal=7% |
| 1282 | BIOCARTA\_EIF4\_PATHWAY |  | 15 | -0.23 | -0.56 | 0.964 | 1.000 | 1.000 | 240 | tags=7%, list=4%, signal=7% |
| 1283 | GRADE\_COLON\_CANCER\_UP |  | 300 | -0.16 | -0.56 | 0.998 | 1.000 | 1.000 | 1924 | tags=25%, list=34%, signal=35% |
| 1284 | DAIRKEE\_TERT\_TARGETS\_UP |  | 154 | -0.16 | -0.55 | 0.999 | 1.000 | 1.000 | 1327 | tags=17%, list=23%, signal=21% |
| 1285 | REACTOME\_REV\_MEDIATED\_NUCLEAR\_EXPORT\_OF\_HIV1\_RNA |  | 18 | -0.21 | -0.55 | 0.968 | 1.000 | 1.000 | 2632 | tags=39%, list=46%, signal=72% |
| 1286 | REACTOME\_VPR\_MEDIATED\_NUCLEAR\_IMPORT\_OF\_PICS |  | 18 | -0.21 | -0.55 | 0.970 | 1.000 | 1.000 | 368 | tags=6%, list=6%, signal=6% |
| 1287 | REACTOME\_SCF\_SKP2\_MEDIATED\_DEGRADATION\_OF\_P27\_P21 |  | 32 | -0.19 | -0.54 | 0.976 | 1.000 | 1.000 | 2573 | tags=41%, list=45%, signal=73% |
| 1288 | LOCKWOOD\_AMPLIFIED\_IN\_LUNG\_CANCER |  | 79 | -0.17 | -0.54 | 0.996 | 1.000 | 1.000 | 1296 | tags=18%, list=23%, signal=23% |
| 1289 | AGUIRRE\_PANCREATIC\_CANCER\_COPY\_NUMBER\_DN |  | 101 | -0.16 | -0.54 | 0.995 | 1.000 | 1.000 | 2318 | tags=40%, list=41%, signal=65% |
| 1290 | LINDGREN\_BLADDER\_CANCER\_WITH\_LOH\_IN\_CHR9Q |  | 53 | -0.17 | -0.53 | 0.992 | 1.000 | 1.000 | 1715 | tags=30%, list=30%, signal=43% |
| 1291 | SESTO\_RESPONSE\_TO\_UV\_C1 |  | 26 | -0.19 | -0.53 | 0.974 | 1.000 | 1.000 | 2412 | tags=38%, list=42%, signal=66% |
| 1292 | MOOTHA\_MITOCHONDRIA |  | 208 | -0.15 | -0.53 | 1.000 | 1.000 | 1.000 | 2086 | tags=33%, list=36%, signal=50% |
| 1293 | REACTOME\_CYTOSOLIC\_TRNA\_AMINOACYLATION |  | 19 | -0.20 | -0.53 | 0.964 | 1.000 | 1.000 | 612 | tags=11%, list=11%, signal=12% |
| 1294 | RHODES\_UNDIFFERENTIATED\_CANCER |  | 32 | -0.19 | -0.53 | 0.977 | 1.000 | 1.000 | 1444 | tags=22%, list=25%, signal=29% |
| 1295 | KEGG\_CITRATE\_CYCLE\_TCA\_CYCLE |  | 19 | -0.21 | -0.53 | 0.975 | 1.000 | 1.000 | 1698 | tags=26%, list=30%, signal=37% |
| 1296 | REACTOME\_NUCLEAR\_IMPORT\_OF\_REV\_PROTEIN |  | 17 | -0.21 | -0.52 | 0.962 | 1.000 | 1.000 | 368 | tags=6%, list=6%, signal=6% |
| 1297 | SCHLOSSER\_MYC\_TARGETS\_AND\_SERUM\_RESPONSE\_DN |  | 27 | -0.19 | -0.52 | 0.986 | 1.000 | 1.000 | 411 | tags=7%, list=7%, signal=8% |
| 1298 | GENTILE\_UV\_RESPONSE\_CLUSTER\_D2 |  | 15 | -0.21 | -0.52 | 0.980 | 1.000 | 1.000 | 1814 | tags=33%, list=32%, signal=49% |
| 1299 | WEI\_MYCN\_TARGETS\_WITH\_E\_BOX |  | 412 | -0.15 | -0.52 | 1.000 | 1.000 | 1.000 | 2112 | tags=32%, list=37%, signal=47% |
| 1300 | CAMPS\_COLON\_CANCER\_COPY\_NUMBER\_UP |  | 18 | -0.20 | -0.52 | 0.976 | 1.000 | 1.000 | 1016 | tags=17%, list=18%, signal=20% |
| 1301 | KEGG\_CHRONIC\_MYELOID\_LEUKEMIA |  | 42 | -0.17 | -0.52 | 0.992 | 1.000 | 1.000 | 1305 | tags=17%, list=23%, signal=21% |
| 1302 | REACTOME\_CYCLIN\_E\_ASSOCIATED\_EVENTS\_DURING\_G1\_S\_TRANSITION\_ |  | 36 | -0.18 | -0.52 | 0.992 | 1.000 | 1.000 | 2573 | tags=39%, list=45%, signal=70% |
| 1303 | REACTOME\_TRANSPORT\_OF\_RIBONUCLEOPROTEINS\_INTO\_THE\_HOST\_NUCLEUS |  | 18 | -0.20 | -0.51 | 0.975 | 1.000 | 1.000 | 368 | tags=6%, list=6%, signal=6% |
| 1304 | ST\_FAS\_SIGNALING\_PATHWAY |  | 30 | -0.18 | -0.51 | 0.988 | 1.000 | 1.000 | 582 | tags=10%, list=10%, signal=11% |
| 1305 | REACTOME\_MAP\_KINASES\_ACTIVATION\_IN\_TLR\_CASCADE |  | 24 | -0.18 | -0.51 | 0.983 | 1.000 | 1.000 | 2079 | tags=33%, list=36%, signal=52% |
| 1306 | BIOCARTA\_GPCR\_PATHWAY |  | 17 | -0.20 | -0.50 | 0.990 | 1.000 | 1.000 | 1305 | tags=18%, list=23%, signal=23% |
| 1307 | KEGG\_CYTOSOLIC\_DNA\_SENSING\_PATHWAY |  | 20 | -0.19 | -0.50 | 0.986 | 1.000 | 1.000 | 1852 | tags=40%, list=32%, signal=59% |
| 1308 | REACTOME\_METABOLISM\_OF\_PROTEINS |  | 79 | -0.15 | -0.49 | 0.996 | 1.000 | 1.000 | 2256 | tags=25%, list=39%, signal=41% |
| 1309 | REACTOME\_CDC20\_PHOSPHO\_APC\_MEDIATED\_DEGRADATION\_OF\_CYCLIN\_A |  | 41 | -0.16 | -0.48 | 0.997 | 1.000 | 1.000 | 2665 | tags=41%, list=47%, signal=77% |
| 1310 | WEST\_ADRENOCORTICAL\_TUMOR\_UP |  | 145 | -0.14 | -0.46 | 1.000 | 1.000 | 1.000 | 2651 | tags=48%, list=46%, signal=86% |
| 1311 | MARTINEZ\_RESPONSE\_TO\_TRABECTEDIN\_DN |  | 129 | -0.14 | -0.46 | 1.000 | 1.000 | 1.000 | 2248 | tags=33%, list=39%, signal=54% |
| 1312 | REACTOME\_REGULATION\_OF\_APC\_ACTIVATORS\_BETWEEN\_G1\_S\_AND\_EARLY\_ANAPHASE |  | 44 | -0.15 | -0.46 | 0.996 | 1.000 | 1.000 | 2665 | tags=41%, list=47%, signal=76% |
| 1313 | KEGG\_OOCYTE\_MEIOSIS |  | 49 | -0.15 | -0.46 | 0.999 | 1.000 | 1.000 | 2741 | tags=51%, list=48%, signal=97% |
| 1314 | CHOW\_RASSF1\_TARGETS\_UP |  | 17 | -0.18 | -0.45 | 0.994 | 1.000 | 1.000 | 330 | tags=6%, list=6%, signal=6% |
| 1315 | ZHANG\_RESPONSE\_TO\_IKK\_INHIBITOR\_AND\_TNF\_DN |  | 35 | -0.15 | -0.44 | 0.998 | 1.000 | 1.000 | 1516 | tags=20%, list=26%, signal=27% |
| 1316 | REACTOME\_HIV\_LIFE\_CYCLE |  | 56 | -0.14 | -0.42 | 1.000 | 1.000 | 1.000 | 2632 | tags=36%, list=46%, signal=65% |
| 1317 | REACTOME\_TRANSPORT\_OF\_THE\_SLBP\_INDEPENDENT\_MATURE\_MRNA |  | 20 | -0.16 | -0.40 | 0.998 | 1.000 | 1.000 | 368 | tags=5%, list=6%, signal=5% |
| 1318 | REACTOME\_TOLL\_LIKE\_RECEPTOR\_3\_CASCADE |  | 34 | -0.14 | -0.40 | 0.998 | 1.000 | 1.000 | 2811 | tags=53%, list=49%, signal=103% |
| 1319 | RAMASWAMY\_METASTASIS\_UP |  | 29 | -0.14 | -0.39 | 0.999 | 1.000 | 1.000 | 460 | tags=7%, list=8%, signal=7% |
| 1320 | REACTOME\_TRANSLATION |  | 41 | -0.13 | -0.38 | 1.000 | 1.000 | 1.000 | 3536 | tags=61%, list=62%, signal=158% |
| 1321 | BIDUS\_METASTASIS\_UP |  | 113 | -0.11 | -0.38 | 1.000 | 1.000 | 1.000 | 1978 | tags=26%, list=35%, signal=38% |
| 1322 | REACTOME\_INFLUENZA\_LIFE\_CYCLE |  | 52 | -0.11 | -0.35 | 1.000 | 1.000 | 1.000 | 3428 | tags=56%, list=60%, signal=138% |
| 1323 | NIKOLSKY\_BREAST\_CANCER\_20Q11\_AMPLICON |  | 16 | -0.14 | -0.35 | 0.999 | 1.000 | 1.000 | 1446 | tags=19%, list=25%, signal=25% |
| 1324 | REACTOME\_LATE\_PHASE\_OF\_HIV\_LIFE\_CYCLE |  | 49 | -0.09 | -0.28 | 1.000 | 1.000 | 1.000 | 2632 | tags=35%, list=46%, signal=64% |
| 1325 | KONDO\_PROSTATE\_CANCER\_WITH\_H3K27ME3 |  | 27 | -0.73 | -2.02 | 0.000 | 0.000 | 0.013 | 806 | tags=70%, list=14%, signal=82% |
| 1326 | CLASPER\_LYMPHATIC\_VESSELS\_DURING\_METASTASIS\_DN |  | 16 | -0.69 | -1.71 | 0.003 | 0.002 | 0.925 | 808 | tags=63%, list=14%, signal=73% |
| 1327 | HAN\_SATB1\_TARGETS\_UP |  | 109 | -0.52 | -1.74 | 0.000 | 0.001 | 0.841 | 923 | tags=35%, list=16%, signal=41% |
| 1328 | SHEDDEN\_LUNG\_CANCER\_GOOD\_SURVIVAL\_A4 |  | 68 | -0.50 | -1.59 | 0.003 | 0.007 | 1.000 | 1150 | tags=40%, list=20%, signal=49% |
| 1329 | MELLMAN\_TUT1\_TARGETS\_DN |  | 20 | -0.27 | -0.71 | 0.855 | 0.732 | 1.000 | 1548 | tags=30%, list=27%, signal=41% |
Table: Gene sets enriched in phenotype **Normal (3 samples)**[plain text format]****

  
