# Supplementary material for: Comparative RNA-Seq and Microarray Analysis of Gene Expression Changes in B-Cell Lymphomas of Canis familiaris
Source: PLoS One. 2013 Apr 4;8(4):e61088. doi: 10.1371/journal.pone.0061088 (PMC3617154; doi:10.1371/journal.pone.0061088)
Supplement: Data File S2 — GSEA Results Files. (ZIP) [file pone.0061088.s005.zip › RNA-Seq/GSEA-Lymphoma-Enriched-by-RNA-Seq.pdf]

Lymphoma Enriched by RNA-Seq v2.5	*Lymphoma Enriched by RNA-Seq v3.0*
SIG_BCR_SIGNALING_PATHWAY	GINESTIER_BREAST_CANCER_ZNF217_AMPLIFIED_DN
HSA04662_B_CELL_RECEPTOR_SIGNALING_PATHWAY	GINESTIER_BREAST_CANCER_20Q13_AMPLIFICATION_DN
SHEPARD_GENES_COMMON_BW_CB_MO	CROONQUIST_IL6_DEPRIVATION_DN
MANALO_HYPOXIA_DN	WELCSH_BRCA1_TARGETS_1_DN
CELL_CYCLE	NADERI_BREAST_CANCER_PROGNOSIS_UP
CROONQUIST_IL6_STARVE_UP	SHEPARD_BMYB_TARGETS
ZHAN_MM_CD138_PR_VS_REST	SIG_BCR_SIGNALING_PATHWAY
BRENTANI_REPAIR	SOTIRIOU_BREAST_CANCER_GRADE_1_VS_3_UP
BRCA1_OVEREXP_DN	REN_BOUND_BY_E2F
P21_P53_MIDDLE_DN	KANG_DOXORUBICIN_RESISTANCE_UP
DOX_RESIST_GASTRIC_UP	ROSTY_CERVICAL_CANCER_PROLIFERATION_CLUSTER
P21_P53_ANY_DN	SCHLOSSER_MYC_TARGETS_AND_SERUM_RESPONSE_UP
DNA_REPLICATION_REACTOME	FRASOR_RESPONSE_TO_SERM_OR_FULVESTRANT_DN
BCRPATHWAY	CROONQUIST_NRAS_SIGNALING_DN
ADIP_DIFF_CLUSTER5	KEGG_B_CELL_RECEPTOR_SIGNALING_PATHWAY
LI_FETAL_VS_WT_KIDNEY_DN	PUJANA_BREAST_CANCER_LIT_INT_NETWORK
CANTHARIDIN_DN	REACTOME_DNA_STRAND_ELONGATION
RIBOSOMAL_PROTEINS	REACTOME_DNA_REPAIR
SIG_PIP3_SIGNALING_IN_B_LYMPHOCYTES	PASQUALUCCI_LYMPHOMA_BY_GC_STAGE_DN
ST_B_CELL_ANTIGEN_RECEPTOR	KAUFFMANN_DNA_REPAIR_GENES

Supporting gene set enrichments for B cell biology (highlighted) from the top 20 enriched gene sets in lymphoma RNA-Seq by GSEA using the version 2.5 and 3.0 gene set databases. The three B-cell maps shown in version 2.5 but not version 3.0 are still represented in the 3.0 enrichment list, but at a lower rank. Namely, BIOCARTA_BCR_PATHWAY is rank 22, ST_B_CELL_ANTIGEN_RECEPTOR is rank 27, and SIG_PIP3_SIGNALING_IN_B_LYMPHOCYTES is rank 33.
