# Supplementary material for: Comparative RNA-Seq and Microarray Analysis of Gene Expression Changes in B-Cell Lymphomas of Canis familiaris
Source: PLoS One. 2013 Apr 4;8(4):e61088. doi: 10.1371/journal.pone.0061088 (PMC3617154; doi:10.1371/journal.pone.0061088)
Supplement: Data File S2 — GSEA Results Files. (ZIP) [file pone.0061088.s005.zip › RNA-Seq/index-RNA-Seq-v3.html]

Index for xtools.gsea.Gsea my\_analysis.Gsea.1334928352252

### GSEA Report for Dataset master-all-probes

#### Enrichment in phenotype: **LymphomaSeq (4 samples)**

- 276 / 1605 gene sets are upregulated in phenotype **LymphomaSeq**- 103 gene sets are significant at FDR < 25%- 55 gene sets are significantly enriched at nominal pvalue < 1%- 87 gene sets are significantly enriched at nominal pvalue < 5%- Snapshot of enrichment results- Detailed enrichment results in html format- Detailed enrichment results in excel format (tab delimited text)- Guide to interpret results

#### Enrichment in phenotype: **Normal (3 samples)**

- 1329 / 1605 gene sets are upregulated in phenotype **Normal**- 594 gene sets are significantly enriched at FDR < 25%- 346 gene sets are significantly enriched at nominal pvalue < 1%- 486 gene sets are significantly enriched at nominal pvalue < 5%- Snapshot of enrichment results- Detailed enrichment results in html format- Detailed enrichment results in excel format (tab delimited text)- Guide to interpret results

#### Dataset details

- The dataset has 5723 features (genes)- No probe set => gene symbol collapsing was requested, so all 5723 features were used

#### Gene set details

- Gene set size filters (min=15, max=500) resulted in filtering out 1667 / 3272 gene sets- The remaining 1605 gene sets were used in the analysis- List of gene sets used and their sizes (restricted to features in the specified dataset)

#### Gene markers for the **LymphomaSeq** *versus* **Normal** comparison

- The dataset has 5723 features (genes)- # of markers for phenotype **LymphomaSeq**: 1959 (34.2% ) with correlation area 27.4%- # of markers for phenotype **Normal**: 3764 (65.8% ) with correlation area 72.6%- Detailed rank ordered gene list for all features in the dataset- Heat map and gene list correlation  profile for all features in the dataset

#### Global statistics and plots

- Plot of p-values *vs.* NES- Global ES histogram

#### Other

- Parameters used for this analysis

---

Report: my\_analysis.Gsea.1334928352252.rpt   by user: Marie.Mooney

xtools.gsea.Gsea [Fri, Apr 20, '12 9 AM 25]

Website: www.broadinstitute.org/GSEA
Questions & Suggestions: Email
