# Supplementary material for: Comparative RNA-Seq and Microarray Analysis of Gene Expression Changes in B-Cell Lymphomas of Canis familiaris
Source: PLoS One. 2013 Apr 4;8(4):e61088. doi: 10.1371/journal.pone.0061088 (PMC3617154; doi:10.1371/journal.pone.0061088)
Supplement: Data File S2 — GSEA Results Files. (ZIP) [file pone.0061088.s005.zip › Array/gsea_report_for_LymphomaArray_v3.html]

Report for LymphomaArray 1334936519408 [GSEA]

| GS  follow link to MSigDB | GS DETAILS | SIZE | ES | NES | NOM p-val | FDR q-val | FWER p-val | RANK AT MAX | LEADING EDGE || 1 | CROONQUIST\_IL6\_DEPRIVATION\_DN | Details ... | 43 | 0.76 | 2.92 | 0.000 | 0.000 | 0.000 | 704 | tags=77%, list=12%, signal=87% |
| 2 | ROSTY\_CERVICAL\_CANCER\_PROLIFERATION\_CLUSTER | Details ... | 65 | 0.71 | 2.89 | 0.000 | 0.000 | 0.000 | 559 | tags=62%, list=10%, signal=67% |
| 3 | CROONQUIST\_NRAS\_SIGNALING\_DN | Details ... | 40 | 0.77 | 2.87 | 0.000 | 0.000 | 0.000 | 662 | tags=75%, list=12%, signal=84% |
| 4 | REACTOME\_CELL\_CYCLE\_MITOTIC | Details ... | 176 | 0.53 | 2.75 | 0.000 | 0.000 | 0.000 | 895 | tags=39%, list=16%, signal=44% |
| 5 | SOTIRIOU\_BREAST\_CANCER\_GRADE\_1\_VS\_3\_UP | Details ... | 72 | 0.66 | 2.74 | 0.000 | 0.000 | 0.000 | 704 | tags=54%, list=12%, signal=61% |
| 6 | ZHAN\_MULTIPLE\_MYELOMA\_PR\_UP | Details ... | 22 | 0.85 | 2.71 | 0.000 | 0.000 | 0.000 | 422 | tags=77%, list=7%, signal=83% |
| 7 | KANG\_DOXORUBICIN\_RESISTANCE\_UP | Details ... | 25 | 0.76 | 2.61 | 0.000 | 0.000 | 0.000 | 1072 | tags=88%, list=19%, signal=108% |
| 8 | KOBAYASHI\_EGFR\_SIGNALING\_24HR\_DN | Details ... | 123 | 0.57 | 2.58 | 0.000 | 0.000 | 0.000 | 759 | tags=59%, list=13%, signal=66% |
| 9 | REACTOME\_CELL\_CYCLE\_CHECKPOINTS | Details ... | 71 | 0.61 | 2.58 | 0.000 | 0.000 | 0.000 | 1516 | tags=58%, list=26%, signal=78% |
| 10 | ODONNELL\_TARGETS\_OF\_MYC\_AND\_TFRC\_DN | Details ... | 25 | 0.77 | 2.56 | 0.000 | 0.000 | 0.000 | 555 | tags=60%, list=10%, signal=66% |
| 11 | REACTOME\_ACTIVATION\_OF\_ATR\_IN\_RESPONSE\_TO\_REPLICATION\_STRESS | Details ... | 23 | 0.76 | 2.55 | 0.000 | 0.000 | 0.000 | 1219 | tags=83%, list=21%, signal=105% |
| 12 | REACTOME\_G2\_M\_CHECKPOINTS | Details ... | 27 | 0.76 | 2.55 | 0.000 | 0.000 | 0.000 | 965 | tags=74%, list=17%, signal=89% |
| 13 | REACTOME\_MITOTIC\_M\_M\_G1\_PHASES | Details ... | 93 | 0.58 | 2.54 | 0.000 | 0.000 | 0.000 | 927 | tags=39%, list=16%, signal=45% |
| 14 | REN\_BOUND\_BY\_E2F | Details ... | 30 | 0.74 | 2.51 | 0.000 | 0.000 | 0.000 | 1059 | tags=77%, list=19%, signal=94% |
| 15 | REACTOME\_G1\_S\_TRANSITION | Details ... | 55 | 0.63 | 2.50 | 0.000 | 0.000 | 0.000 | 970 | tags=47%, list=17%, signal=56% |
| 16 | GRAHAM\_NORMAL\_QUIESCENT\_VS\_NORMAL\_DIVIDING\_DN | Details ... | 47 | 0.63 | 2.47 | 0.000 | 0.000 | 0.000 | 759 | tags=74%, list=13%, signal=85% |
| 17 | NADERI\_BREAST\_CANCER\_PROGNOSIS\_UP | Details ... | 15 | 0.86 | 2.46 | 0.000 | 0.000 | 0.001 | 692 | tags=73%, list=12%, signal=83% |
| 18 | KAUFFMANN\_DNA\_REPAIR\_GENES | Details ... | 101 | 0.55 | 2.44 | 0.000 | 0.000 | 0.001 | 1159 | tags=51%, list=20%, signal=63% |
| 19 | SONG\_TARGETS\_OF\_IE86\_CMV\_PROTEIN | Details ... | 30 | 0.68 | 2.42 | 0.000 | 0.000 | 0.001 | 1072 | tags=77%, list=19%, signal=94% |
| 20 | FARMER\_BREAST\_CANCER\_CLUSTER\_2 | Details ... | 19 | 0.79 | 2.42 | 0.000 | 0.000 | 0.001 | 662 | tags=74%, list=12%, signal=83% |
| 21 | REACTOME\_ACTIVATION\_OF\_THE\_PRE\_REPLICATIVE\_COMPLEX |  | 17 | 0.78 | 2.41 | 0.000 | 0.000 | 0.001 | 970 | tags=76%, list=17%, signal=92% |
| 22 | REACTOME\_MITOTIC\_PROMETAPHASE |  | 56 | 0.61 | 2.39 | 0.000 | 0.000 | 0.001 | 414 | tags=32%, list=7%, signal=34% |
| 23 | CHANG\_CYCLING\_GENES |  | 22 | 0.73 | 2.39 | 0.000 | 0.000 | 0.001 | 759 | tags=68%, list=13%, signal=78% |
| 24 | WHITEFORD\_PEDIATRIC\_CANCER\_MARKERS |  | 37 | 0.64 | 2.37 | 0.000 | 0.000 | 0.003 | 1075 | tags=68%, list=19%, signal=83% |
| 25 | MUELLER\_PLURINET |  | 141 | 0.52 | 2.36 | 0.000 | 0.000 | 0.003 | 914 | tags=48%, list=16%, signal=56% |
| 26 | SHEPARD\_BMYB\_TARGETS |  | 21 | 0.73 | 2.36 | 0.000 | 0.000 | 0.003 | 466 | tags=62%, list=8%, signal=67% |
| 27 | PUJANA\_XPRSS\_INT\_NETWORK |  | 112 | 0.54 | 2.34 | 0.000 | 0.000 | 0.003 | 1383 | tags=56%, list=24%, signal=73% |
| 28 | REACTOME\_DNA\_REPLICATION\_PRE\_INITIATION |  | 44 | 0.61 | 2.34 | 0.000 | 0.000 | 0.003 | 1516 | tags=55%, list=26%, signal=74% |
| 29 | MORI\_LARGE\_PRE\_BII\_LYMPHOCYTE\_UP |  | 23 | 0.68 | 2.26 | 0.000 | 0.000 | 0.006 | 1007 | tags=74%, list=18%, signal=89% |
| 30 | MANALO\_HYPOXIA\_DN |  | 165 | 0.50 | 2.25 | 0.000 | 0.001 | 0.009 | 1135 | tags=58%, list=20%, signal=70% |
| 31 | REACTOME\_METABOLISM\_OF\_RNA |  | 55 | 0.53 | 2.23 | 0.000 | 0.001 | 0.009 | 1753 | tags=62%, list=31%, signal=88% |
| 32 | WAKASUGI\_HAVE\_ZNF143\_BINDING\_SITES |  | 32 | 0.62 | 2.23 | 0.000 | 0.001 | 0.010 | 1332 | tags=66%, list=23%, signal=85% |
| 33 | REACTOME\_FORMATION\_OF\_A\_POOL\_OF\_FREE\_40S\_SUBUNITS |  | 26 | 0.64 | 2.22 | 0.000 | 0.001 | 0.012 | 1617 | tags=77%, list=28%, signal=107% |
| 34 | PUJANA\_BREAST\_CANCER\_WITH\_BRCA1\_MUTATED\_UP |  | 41 | 0.56 | 2.20 | 0.000 | 0.001 | 0.018 | 899 | tags=63%, list=16%, signal=75% |
| 35 | GRAHAM\_CML\_DIVIDING\_VS\_NORMAL\_QUIESCENT\_UP |  | 89 | 0.50 | 2.19 | 0.000 | 0.001 | 0.019 | 759 | tags=61%, list=13%, signal=69% |
| 36 | REACTOME\_SYNTHESIS\_OF\_DNA |  | 53 | 0.56 | 2.19 | 0.000 | 0.001 | 0.021 | 1128 | tags=49%, list=20%, signal=61% |
| 37 | REACTOME\_SNRNP\_ASSEMBLY |  | 27 | 0.62 | 2.19 | 0.000 | 0.001 | 0.021 | 1753 | tags=74%, list=31%, signal=106% |
| 38 | ODONNELL\_TFRC\_TARGETS\_DN |  | 61 | 0.56 | 2.18 | 0.000 | 0.001 | 0.022 | 555 | tags=49%, list=10%, signal=54% |
| 39 | REACTOME\_PROCESSING\_OF\_CAPPED\_INTRON\_CONTAINING\_PRE\_MRNA |  | 80 | 0.51 | 2.17 | 0.000 | 0.001 | 0.031 | 1968 | tags=69%, list=34%, signal=103% |
| 40 | FERREIRA\_EWINGS\_SARCOMA\_UNSTABLE\_VS\_STABLE\_UP |  | 68 | 0.54 | 2.17 | 0.000 | 0.001 | 0.031 | 710 | tags=51%, list=12%, signal=58% |
| 41 | REACTOME\_GTP\_HYDROLYSIS\_AND\_JOINING\_OF\_THE\_60S\_RIBOSOMAL\_SUBUNIT |  | 33 | 0.58 | 2.13 | 0.000 | 0.002 | 0.045 | 1746 | tags=76%, list=31%, signal=108% |
| 42 | YU\_MYC\_TARGETS\_UP |  | 22 | 0.64 | 2.13 | 0.000 | 0.002 | 0.046 | 548 | tags=68%, list=10%, signal=75% |
| 43 | REACTOME\_S\_PHASE |  | 62 | 0.53 | 2.13 | 0.000 | 0.002 | 0.047 | 895 | tags=45%, list=16%, signal=53% |
| 44 | REACTOME\_TRANSLATION |  | 41 | 0.54 | 2.13 | 0.000 | 0.002 | 0.048 | 2174 | tags=88%, list=38%, signal=141% |
| 45 | REACTOME\_DNA\_REPAIR |  | 62 | 0.53 | 2.12 | 0.000 | 0.002 | 0.049 | 1263 | tags=55%, list=22%, signal=70% |
| 46 | LEE\_EARLY\_T\_LYMPHOCYTE\_UP |  | 30 | 0.60 | 2.12 | 0.000 | 0.002 | 0.052 | 704 | tags=73%, list=12%, signal=83% |
| 47 | REACTOME\_DOUBLE\_STRAND\_BREAK\_REPAIR |  | 15 | 0.72 | 2.10 | 0.000 | 0.002 | 0.061 | 1219 | tags=73%, list=21%, signal=93% |
| 48 | REACTOME\_DNA\_STRAND\_ELONGATION |  | 22 | 0.64 | 2.10 | 0.000 | 0.002 | 0.061 | 1219 | tags=86%, list=21%, signal=109% |
| 49 | KEGG\_CELL\_CYCLE |  | 68 | 0.47 | 2.06 | 0.000 | 0.004 | 0.089 | 691 | tags=40%, list=12%, signal=45% |
| 50 | KEGG\_RIBOSOME |  | 20 | 0.68 | 2.04 | 0.000 | 0.004 | 0.102 | 1617 | tags=90%, list=28%, signal=125% |
| 51 | RHODES\_UNDIFFERENTIATED\_CANCER |  | 32 | 0.57 | 2.04 | 0.000 | 0.004 | 0.104 | 555 | tags=47%, list=10%, signal=52% |
| 52 | KEGG\_DNA\_REPLICATION |  | 24 | 0.62 | 2.04 | 0.000 | 0.004 | 0.104 | 1219 | tags=79%, list=21%, signal=100% |
| 53 | REACTOME\_FORMATION\_OF\_THE\_TERNARY\_COMPLEX\_AND\_SUBSEQUENTLY\_THE\_43S\_COMPLEX |  | 21 | 0.65 | 2.02 | 0.000 | 0.004 | 0.117 | 1617 | tags=76%, list=28%, signal=106% |
| 54 | FURUKAWA\_DUSP6\_TARGETS\_PCI35\_DN |  | 29 | 0.55 | 2.02 | 0.000 | 0.005 | 0.120 | 972 | tags=69%, list=17%, signal=83% |
| 55 | REACTOME\_INFLUENZA\_LIFE\_CYCLE |  | 52 | 0.48 | 2.01 | 0.000 | 0.005 | 0.134 | 1906 | tags=73%, list=33%, signal=109% |
| 56 | MOLENAAR\_TARGETS\_OF\_CCND1\_AND\_CDK4\_DN |  | 27 | 0.59 | 1.99 | 0.000 | 0.006 | 0.169 | 623 | tags=63%, list=11%, signal=70% |
| 57 | PUJANA\_BREAST\_CANCER\_LIT\_INT\_NETWORK |  | 56 | 0.48 | 1.99 | 0.000 | 0.007 | 0.177 | 912 | tags=39%, list=16%, signal=46% |
| 58 | REACTOME\_ORC1\_REMOVAL\_FROM\_CHROMATIN |  | 36 | 0.53 | 1.96 | 0.000 | 0.008 | 0.211 | 781 | tags=31%, list=14%, signal=35% |
| 59 | BIDUS\_METASTASIS\_UP |  | 113 | 0.42 | 1.95 | 0.000 | 0.008 | 0.216 | 1138 | tags=38%, list=20%, signal=47% |
| 60 | HORIUCHI\_WTAP\_TARGETS\_DN |  | 151 | 0.41 | 1.95 | 0.000 | 0.008 | 0.216 | 718 | tags=36%, list=13%, signal=40% |
| 61 | SCHLOSSER\_MYC\_TARGETS\_REPRESSED\_BY\_SERUM |  | 100 | 0.43 | 1.95 | 0.000 | 0.008 | 0.216 | 1943 | tags=65%, list=34%, signal=97% |
| 62 | REACTOME\_TRANSLATION\_INITIATION\_COMPLEX\_FORMATION |  | 24 | 0.59 | 1.94 | 0.000 | 0.009 | 0.238 | 1617 | tags=71%, list=28%, signal=98% |
| 63 | REACTOME\_TRANSPORT\_OF\_THE\_SLBP\_INDEPENDENT\_MATURE\_MRNA |  | 20 | 0.60 | 1.94 | 0.000 | 0.009 | 0.240 | 1906 | tags=75%, list=33%, signal=112% |
| 64 | REACTOME\_M\_G1\_TRANSITION |  | 34 | 0.55 | 1.94 | 0.000 | 0.008 | 0.240 | 1516 | tags=47%, list=26%, signal=64% |
| 65 | REACTOME\_MRNA\_SPLICING |  | 61 | 0.47 | 1.92 | 0.000 | 0.010 | 0.269 | 1968 | tags=67%, list=34%, signal=101% |
| 66 | FRASOR\_RESPONSE\_TO\_SERM\_OR\_FULVESTRANT\_DN |  | 21 | 0.62 | 1.91 | 0.006 | 0.010 | 0.281 | 753 | tags=67%, list=13%, signal=76% |
| 67 | REACTOME\_REV\_MEDIATED\_NUCLEAR\_EXPORT\_OF\_HIV1\_RNA |  | 18 | 0.60 | 1.89 | 0.000 | 0.011 | 0.316 | 1906 | tags=78%, list=33%, signal=116% |
| 68 | REACTOME\_CYCLIN\_E\_ASSOCIATED\_EVENTS\_DURING\_G1\_S\_TRANSITION\_ |  | 36 | 0.51 | 1.89 | 0.010 | 0.011 | 0.317 | 1691 | tags=50%, list=30%, signal=71% |
| 69 | RIZ\_ERYTHROID\_DIFFERENTIATION |  | 29 | 0.54 | 1.89 | 0.000 | 0.011 | 0.325 | 662 | tags=38%, list=12%, signal=43% |
| 70 | BENPORATH\_ES\_1 |  | 135 | 0.39 | 1.88 | 0.000 | 0.011 | 0.332 | 1059 | tags=43%, list=19%, signal=51% |
| 71 | VECCHI\_GASTRIC\_CANCER\_EARLY\_UP |  | 163 | 0.46 | 1.88 | 0.000 | 0.011 | 0.335 | 837 | tags=49%, list=15%, signal=56% |
| 72 | MISSIAGLIA\_REGULATED\_BY\_METHYLATION\_DN |  | 45 | 0.44 | 1.87 | 0.000 | 0.012 | 0.356 | 1476 | tags=67%, list=26%, signal=89% |
| 73 | MOREAUX\_B\_LYMPHOCYTE\_MATURATION\_BY\_TACI\_DN |  | 26 | 0.54 | 1.87 | 0.000 | 0.012 | 0.356 | 1311 | tags=46%, list=23%, signal=60% |
| 74 | REACTOME\_TRANSPORT\_OF\_MATURE\_MRNA\_DERIVED\_FROM\_AN\_INTRON\_CONTAINING\_TRANSCRIPT |  | 27 | 0.54 | 1.87 | 0.000 | 0.012 | 0.367 | 1936 | tags=70%, list=34%, signal=106% |
| 75 | REACTOME\_ELONGATION\_AND\_PROCESSING\_OF\_CAPPED\_TRANSCRIPTS |  | 76 | 0.44 | 1.87 | 0.000 | 0.012 | 0.368 | 2024 | tags=68%, list=35%, signal=104% |
| 76 | REACTOME\_EXTENSION\_OF\_TELOMERES |  | 18 | 0.58 | 1.86 | 0.000 | 0.012 | 0.383 | 1219 | tags=83%, list=21%, signal=106% |
| 77 | SHEPARD\_CRUSH\_AND\_BURN\_MUTANT\_DN |  | 47 | 0.48 | 1.86 | 0.000 | 0.013 | 0.396 | 466 | tags=38%, list=8%, signal=41% |
| 78 | KEGG\_MISMATCH\_REPAIR |  | 18 | 0.61 | 1.86 | 0.006 | 0.013 | 0.397 | 895 | tags=72%, list=16%, signal=85% |
| 79 | REACTOME\_LATE\_PHASE\_OF\_HIV\_LIFE\_CYCLE |  | 49 | 0.45 | 1.86 | 0.000 | 0.013 | 0.403 | 2024 | tags=71%, list=35%, signal=110% |
| 80 | REACTOME\_GLUCOSE\_TRANSPORT |  | 19 | 0.59 | 1.85 | 0.000 | 0.013 | 0.410 | 1906 | tags=74%, list=33%, signal=110% |
| 81 | REACTOME\_FORMATION\_AND\_MATURATION\_OF\_MRNA\_TRANSCRIPT |  | 83 | 0.45 | 1.85 | 0.000 | 0.013 | 0.414 | 2024 | tags=67%, list=35%, signal=103% |
| 82 | LI\_WILMS\_TUMOR\_VS\_FETAL\_KIDNEY\_1\_DN |  | 76 | 0.44 | 1.85 | 0.000 | 0.013 | 0.415 | 1059 | tags=50%, list=19%, signal=61% |
| 83 | REACTOME\_PEPTIDE\_CHAIN\_ELONGATION |  | 18 | 0.60 | 1.85 | 0.006 | 0.013 | 0.421 | 1617 | tags=83%, list=28%, signal=116% |
| 84 | KEGG\_NUCLEOTIDE\_EXCISION\_REPAIR |  | 31 | 0.50 | 1.85 | 0.000 | 0.013 | 0.423 | 1263 | tags=61%, list=22%, signal=78% |
| 85 | REACTOME\_NUCLEAR\_IMPORT\_OF\_REV\_PROTEIN |  | 17 | 0.57 | 1.83 | 0.005 | 0.014 | 0.461 | 1906 | tags=71%, list=33%, signal=106% |
| 86 | ZHANG\_RESPONSE\_TO\_CANTHARIDIN\_DN |  | 30 | 0.53 | 1.83 | 0.000 | 0.015 | 0.491 | 1745 | tags=67%, list=30%, signal=95% |
| 87 | REACTOME\_REGULATION\_OF\_APC\_ACTIVATORS\_BETWEEN\_G1\_S\_AND\_EARLY\_ANAPHASE |  | 44 | 0.46 | 1.82 | 0.000 | 0.016 | 0.523 | 1516 | tags=41%, list=26%, signal=55% |
| 88 | FUJII\_YBX1\_TARGETS\_DN |  | 74 | 0.42 | 1.82 | 0.000 | 0.016 | 0.529 | 769 | tags=42%, list=13%, signal=48% |
| 89 | CHNG\_MULTIPLE\_MYELOMA\_HYPERPLOID\_UP |  | 23 | 0.53 | 1.81 | 0.000 | 0.016 | 0.535 | 1661 | tags=61%, list=29%, signal=85% |
| 90 | REACTOME\_CDC20\_PHOSPHO\_APC\_MEDIATED\_DEGRADATION\_OF\_CYCLIN\_A |  | 41 | 0.50 | 1.81 | 0.000 | 0.016 | 0.536 | 1516 | tags=44%, list=26%, signal=59% |
| 91 | REACTOME\_INFLUENZA\_VIRAL\_RNA\_TRANSCRIPTION\_AND\_REPLICATION |  | 29 | 0.50 | 1.81 | 0.008 | 0.016 | 0.539 | 1893 | tags=83%, list=33%, signal=123% |
| 92 | REACTOME\_REGULATION\_OF\_GLUCOKINASE\_BY\_GLUCOKINASE\_REGULATORY\_PROTEIN |  | 16 | 0.59 | 1.81 | 0.016 | 0.016 | 0.556 | 1906 | tags=75%, list=33%, signal=112% |
| 93 | MOOTHA\_VOXPHOS |  | 38 | 0.49 | 1.79 | 0.000 | 0.018 | 0.599 | 1712 | tags=58%, list=30%, signal=82% |
| 94 | REACTOME\_TRANSPORT\_OF\_RIBONUCLEOPROTEINS\_INTO\_THE\_HOST\_NUCLEUS |  | 18 | 0.56 | 1.79 | 0.011 | 0.018 | 0.603 | 1906 | tags=67%, list=33%, signal=100% |
| 95 | REACTOME\_SCF\_SKP2\_MEDIATED\_DEGRADATION\_OF\_P27\_P21 |  | 32 | 0.50 | 1.79 | 0.000 | 0.018 | 0.607 | 1516 | tags=44%, list=26%, signal=59% |
| 96 | SHEDDEN\_LUNG\_CANCER\_POOR\_SURVIVAL\_A6 |  | 224 | 0.46 | 1.78 | 0.000 | 0.019 | 0.622 | 1072 | tags=47%, list=19%, signal=55% |
| 97 | REACTOME\_ELECTRON\_TRANSPORT\_CHAIN |  | 41 | 0.47 | 1.78 | 0.000 | 0.020 | 0.643 | 1712 | tags=59%, list=30%, signal=83% |
| 98 | REACTOME\_VPR\_MEDIATED\_NUCLEAR\_IMPORT\_OF\_PICS |  | 18 | 0.59 | 1.78 | 0.011 | 0.020 | 0.644 | 1906 | tags=72%, list=33%, signal=108% |
| 99 | MORI\_IMMATURE\_B\_LYMPHOCYTE\_DN |  | 24 | 0.55 | 1.77 | 0.007 | 0.020 | 0.656 | 753 | tags=67%, list=13%, signal=76% |
| 100 | REACTOME\_TELOMERE\_MAINTENANCE |  | 22 | 0.53 | 1.77 | 0.000 | 0.020 | 0.657 | 1219 | tags=68%, list=21%, signal=86% |
| 101 | REACTOME\_NEP\_NS2\_INTERACTS\_WITH\_THE\_CELLULAR\_EXPORT\_MACHINERY |  | 17 | 0.60 | 1.76 | 0.021 | 0.022 | 0.688 | 1906 | tags=76%, list=33%, signal=114% |
| 102 | REACTOME\_VIRAL\_MRNA\_TRANSLATION |  | 19 | 0.56 | 1.75 | 0.006 | 0.022 | 0.693 | 1893 | tags=89%, list=33%, signal=133% |
| 103 | SCIBETTA\_KDM5B\_TARGETS\_DN |  | 34 | 0.47 | 1.75 | 0.010 | 0.022 | 0.710 | 466 | tags=29%, list=8%, signal=32% |
| 104 | HOFFMANN\_LARGE\_TO\_SMALL\_PRE\_BII\_LYMPHOCYTE\_UP |  | 53 | 0.44 | 1.74 | 0.000 | 0.024 | 0.746 | 567 | tags=43%, list=10%, signal=48% |
| 105 | REACTOME\_RNA\_POLYMERASE\_II\_TRANSCRIPTION |  | 49 | 0.43 | 1.74 | 0.000 | 0.024 | 0.748 | 2024 | tags=69%, list=35%, signal=106% |
| 106 | REACTOME\_TRANSCRIPTION\_COUPLED\_NER |  | 28 | 0.51 | 1.72 | 0.009 | 0.027 | 0.786 | 1376 | tags=64%, list=24%, signal=84% |
| 107 | STEIN\_ESRRA\_TARGETS\_RESPONSIVE\_TO\_ESTROGEN\_DN |  | 24 | 0.52 | 1.71 | 0.006 | 0.028 | 0.801 | 759 | tags=67%, list=13%, signal=77% |
| 108 | BLUM\_RESPONSE\_TO\_SALIRASIB\_DN |  | 162 | 0.35 | 1.70 | 0.000 | 0.031 | 0.836 | 759 | tags=38%, list=13%, signal=42% |
| 109 | REACTOME\_MRNA\_3\_END\_PROCESSING |  | 19 | 0.54 | 1.69 | 0.006 | 0.032 | 0.851 | 1936 | tags=74%, list=34%, signal=111% |
| 110 | REACTOME\_RNA\_POLYMERASE\_III\_TRANSCRIPTION\_INITIATION |  | 15 | 0.56 | 1.68 | 0.019 | 0.034 | 0.872 | 2279 | tags=80%, list=40%, signal=133% |
| 111 | REACTOME\_NUCLEOTIDE\_EXCISION\_REPAIR |  | 33 | 0.48 | 1.67 | 0.010 | 0.035 | 0.878 | 1263 | tags=58%, list=22%, signal=73% |
| 112 | MARKEY\_RB1\_ACUTE\_LOF\_DN |  | 104 | 0.37 | 1.67 | 0.000 | 0.035 | 0.878 | 1325 | tags=54%, list=23%, signal=69% |
| 113 | REACTOME\_REGULATION\_OF\_GENE\_EXPRESSION\_IN\_BETA\_CELLS |  | 18 | 0.53 | 1.67 | 0.028 | 0.035 | 0.880 | 1617 | tags=83%, list=28%, signal=116% |
| 114 | GARGALOVIC\_RESPONSE\_TO\_OXIDIZED\_PHOSPHOLIPIDS\_TURQUOISE\_DN |  | 28 | 0.47 | 1.66 | 0.007 | 0.037 | 0.899 | 555 | tags=39%, list=10%, signal=43% |
| 115 | KEGG\_PARKINSONS\_DISEASE |  | 49 | 0.42 | 1.65 | 0.000 | 0.039 | 0.913 | 1569 | tags=51%, list=27%, signal=70% |
| 116 | SU\_TESTIS |  | 28 | 0.47 | 1.64 | 0.031 | 0.041 | 0.923 | 895 | tags=39%, list=16%, signal=46% |
| 117 | WONG\_MITOCHONDRIA\_GENE\_MODULE |  | 105 | 0.35 | 1.64 | 0.083 | 0.042 | 0.924 | 1618 | tags=54%, list=28%, signal=74% |
| 118 | REACTOME\_CDT1\_ASSOCIATION\_WITH\_THE\_CDC6\_ORC\_ORIGIN\_COMPLEX |  | 29 | 0.49 | 1.62 | 0.016 | 0.047 | 0.952 | 1516 | tags=41%, list=26%, signal=56% |
| 119 | CHIANG\_LIVER\_CANCER\_SUBCLASS\_PROLIFERATION\_UP |  | 53 | 0.41 | 1.61 | 0.000 | 0.050 | 0.962 | 557 | tags=47%, list=10%, signal=52% |
| 120 | GARY\_CD5\_TARGETS\_DN |  | 250 | 0.32 | 1.60 | 0.000 | 0.052 | 0.967 | 1429 | tags=41%, list=25%, signal=53% |
| 121 | KEGG\_SPLICEOSOME |  | 66 | 0.38 | 1.57 | 0.000 | 0.060 | 0.976 | 2166 | tags=64%, list=38%, signal=101% |
| 122 | WU\_APOPTOSIS\_BY\_CDKN1A\_VIA\_TP53 |  | 17 | 0.53 | 1.56 | 0.050 | 0.064 | 0.983 | 1210 | tags=71%, list=21%, signal=89% |
| 123 | PYEON\_CANCER\_HEAD\_AND\_NECK\_VS\_CERVICAL\_UP |  | 88 | 0.38 | 1.55 | 0.000 | 0.068 | 0.986 | 669 | tags=31%, list=12%, signal=34% |
| 124 | PYEON\_HPV\_POSITIVE\_TUMORS\_UP |  | 38 | 0.41 | 1.55 | 0.021 | 0.068 | 0.987 | 457 | tags=34%, list=8%, signal=37% |
| 125 | VERNELL\_RETINOBLASTOMA\_PATHWAY\_UP |  | 25 | 0.45 | 1.55 | 0.007 | 0.068 | 0.988 | 1091 | tags=64%, list=19%, signal=79% |
| 126 | RHEIN\_ALL\_GLUCOCORTICOID\_THERAPY\_DN |  | 174 | 0.33 | 1.55 | 0.000 | 0.068 | 0.988 | 1006 | tags=39%, list=18%, signal=46% |
| 127 | LE\_EGR2\_TARGETS\_UP |  | 48 | 0.40 | 1.55 | 0.015 | 0.068 | 0.988 | 753 | tags=42%, list=13%, signal=48% |
| 128 | KEGG\_HOMOLOGOUS\_RECOMBINATION |  | 16 | 0.53 | 1.55 | 0.055 | 0.067 | 0.988 | 1297 | tags=75%, list=23%, signal=97% |
| 129 | SLEBOS\_HEAD\_AND\_NECK\_CANCER\_WITH\_HPV\_UP |  | 31 | 0.43 | 1.54 | 0.020 | 0.068 | 0.990 | 740 | tags=42%, list=13%, signal=48% |
| 130 | MITSIADES\_RESPONSE\_TO\_APLIDIN\_DN |  | 135 | 0.32 | 1.53 | 0.000 | 0.071 | 0.993 | 791 | tags=32%, list=14%, signal=36% |
| 131 | REACTOME\_DUAL\_INCISION\_REACTION\_IN\_TC\_NER |  | 15 | 0.53 | 1.53 | 0.049 | 0.071 | 0.993 | 2007 | tags=80%, list=35%, signal=123% |
| 132 | KEGG\_RNA\_POLYMERASE |  | 16 | 0.52 | 1.53 | 0.060 | 0.073 | 0.993 | 1980 | tags=75%, list=35%, signal=114% |
| 133 | BOYAULT\_LIVER\_CANCER\_SUBCLASS\_G3\_UP |  | 110 | 0.34 | 1.52 | 0.000 | 0.077 | 0.995 | 1303 | tags=40%, list=23%, signal=51% |
| 134 | REACTOME\_P53\_INDEPENDENT\_DNA\_DAMAGE\_RESPONSE |  | 26 | 0.43 | 1.51 | 0.009 | 0.079 | 0.996 | 2337 | tags=69%, list=41%, signal=116% |
| 135 | TOYOTA\_TARGETS\_OF\_MIR34B\_AND\_MIR34C |  | 192 | 0.29 | 1.51 | 0.000 | 0.078 | 0.996 | 869 | tags=29%, list=15%, signal=33% |
| 136 | REACTOME\_AUTODEGRADATION\_OF\_CDH1\_BY\_CDH1\_APC |  | 36 | 0.40 | 1.50 | 0.000 | 0.081 | 0.997 | 2337 | tags=72%, list=41%, signal=121% |
| 137 | REACTOME\_HIV1\_TRANSCRIPTION\_INITIATION |  | 20 | 0.47 | 1.50 | 0.069 | 0.084 | 0.999 | 2007 | tags=70%, list=35%, signal=107% |
| 138 | AMUNDSON\_GAMMA\_RADIATION\_RESPONSE |  | 15 | 0.50 | 1.49 | 0.060 | 0.087 | 1.000 | 555 | tags=47%, list=10%, signal=52% |
| 139 | REACTOME\_TRNA\_AMINOACYLATION |  | 29 | 0.42 | 1.48 | 0.037 | 0.093 | 1.000 | 1055 | tags=38%, list=18%, signal=46% |
| 140 | ZHAN\_EARLY\_DIFFERENTIATION\_GENES\_DN |  | 21 | 0.46 | 1.47 | 0.040 | 0.095 | 1.000 | 1745 | tags=62%, list=30%, signal=89% |
| 141 | REACTOME\_REGULATION\_OF\_ORNITHINE\_DECARBOXYLASE |  | 29 | 0.43 | 1.47 | 0.030 | 0.096 | 1.000 | 2337 | tags=66%, list=41%, signal=110% |
| 142 | SAKAI\_CHRONIC\_HEPATITIS\_VS\_LIVER\_CANCER\_UP |  | 35 | 0.39 | 1.46 | 0.019 | 0.103 | 1.000 | 1993 | tags=60%, list=35%, signal=91% |
| 143 | KEGG\_RNA\_DEGRADATION |  | 35 | 0.39 | 1.45 | 0.010 | 0.104 | 1.000 | 1475 | tags=46%, list=26%, signal=61% |
| 144 | FOURNIER\_ACINAR\_DEVELOPMENT\_LATE\_2 |  | 148 | 0.32 | 1.45 | 0.000 | 0.104 | 1.000 | 911 | tags=34%, list=16%, signal=39% |
| 145 | REACTOME\_TRANSCRIPTION |  | 75 | 0.33 | 1.44 | 0.029 | 0.110 | 1.000 | 1666 | tags=51%, list=29%, signal=71% |
| 146 | KEGG\_AMINOACYL\_TRNA\_BIOSYNTHESIS |  | 28 | 0.41 | 1.44 | 0.018 | 0.111 | 1.000 | 1048 | tags=36%, list=18%, signal=44% |
| 147 | MOREAUX\_MULTIPLE\_MYELOMA\_BY\_TACI\_DN |  | 79 | 0.35 | 1.44 | 0.000 | 0.112 | 1.000 | 2140 | tags=59%, list=37%, signal=94% |
| 148 | REACTOME\_G2\_M\_TRANSITION |  | 45 | 0.38 | 1.43 | 0.028 | 0.112 | 1.000 | 1263 | tags=31%, list=22%, signal=40% |
| 149 | SUNG\_METASTASIS\_STROMA\_DN |  | 24 | 0.43 | 1.43 | 0.050 | 0.115 | 1.000 | 665 | tags=50%, list=12%, signal=56% |
| 150 | REACTOME\_SCF\_BETA\_TRCP\_MEDIATED\_DEGRADATION\_OF\_EMI1 |  | 28 | 0.41 | 1.42 | 0.024 | 0.117 | 1.000 | 2337 | tags=68%, list=41%, signal=114% |
| 151 | REACTOME\_CENTROSOME\_MATURATION |  | 39 | 0.39 | 1.42 | 0.013 | 0.118 | 1.000 | 882 | tags=23%, list=15%, signal=27% |
| 152 | RHODES\_CANCER\_META\_SIGNATURE |  | 29 | 0.40 | 1.41 | 0.053 | 0.123 | 1.000 | 1177 | tags=41%, list=21%, signal=52% |
| 153 | BOYAULT\_LIVER\_CANCER\_SUBCLASS\_G23\_UP |  | 29 | 0.41 | 1.41 | 0.067 | 0.125 | 1.000 | 465 | tags=34%, list=8%, signal=37% |
| 154 | ZHONG\_RESPONSE\_TO\_AZACITIDINE\_AND\_TSA\_DN |  | 34 | 0.38 | 1.40 | 0.078 | 0.126 | 1.000 | 1117 | tags=35%, list=20%, signal=44% |
| 155 | REACTOME\_METABOLISM\_OF\_MRNA |  | 28 | 0.41 | 1.40 | 0.016 | 0.126 | 1.000 | 1721 | tags=50%, list=30%, signal=71% |
| 156 | REACTOME\_TRANSCRIPTION\_OF\_THE\_HIV\_GENOME |  | 31 | 0.38 | 1.40 | 0.044 | 0.126 | 1.000 | 2024 | tags=68%, list=35%, signal=104% |
| 157 | REACTOME\_HIV\_LIFE\_CYCLE |  | 56 | 0.35 | 1.40 | 0.034 | 0.129 | 1.000 | 1609 | tags=54%, list=28%, signal=74% |
| 158 | BROWNE\_INTERFERON\_RESPONSIVE\_GENES |  | 24 | 0.41 | 1.39 | 0.083 | 0.129 | 1.000 | 248 | tags=29%, list=4%, signal=30% |
| 159 | REACTOME\_GLUCOSE\_REGULATION\_OF\_INSULIN\_SECRETION |  | 69 | 0.33 | 1.39 | 0.042 | 0.133 | 1.000 | 1569 | tags=46%, list=27%, signal=63% |
| 160 | GARCIA\_TARGETS\_OF\_FLI1\_AND\_DAX1\_DN |  | 64 | 0.34 | 1.39 | 0.027 | 0.134 | 1.000 | 1170 | tags=44%, list=20%, signal=54% |
| 161 | SCHUHMACHER\_MYC\_TARGETS\_UP |  | 34 | 0.38 | 1.38 | 0.046 | 0.136 | 1.000 | 1702 | tags=68%, list=30%, signal=96% |
| 162 | TIEN\_INTESTINE\_PROBIOTICS\_24HR\_UP |  | 295 | 0.27 | 1.38 | 0.000 | 0.139 | 1.000 | 1264 | tags=36%, list=22%, signal=43% |
| 163 | RUIZ\_TNC\_TARGETS\_DN |  | 69 | 0.32 | 1.38 | 0.000 | 0.139 | 1.000 | 704 | tags=42%, list=12%, signal=47% |
| 164 | REACTOME\_STABILIZATION\_OF\_P53 |  | 30 | 0.40 | 1.36 | 0.073 | 0.150 | 1.000 | 2337 | tags=63%, list=41%, signal=106% |
| 165 | KEGG\_PROTEASOME |  | 25 | 0.40 | 1.34 | 0.116 | 0.162 | 1.000 | 2172 | tags=60%, list=38%, signal=96% |
| 166 | KEGG\_PYRIMIDINE\_METABOLISM |  | 47 | 0.34 | 1.33 | 0.077 | 0.170 | 1.000 | 970 | tags=45%, list=17%, signal=53% |
| 167 | ZHAN\_MULTIPLE\_MYELOMA\_SUBGROUPS |  | 16 | 0.45 | 1.33 | 0.139 | 0.171 | 1.000 | 1106 | tags=50%, list=19%, signal=62% |
| 168 | BROWNE\_HCMV\_INFECTION\_6HR\_UP |  | 18 | 0.45 | 1.33 | 0.119 | 0.172 | 1.000 | 91 | tags=17%, list=2%, signal=17% |
| 169 | DANG\_MYC\_TARGETS\_UP |  | 48 | 0.34 | 1.32 | 0.053 | 0.179 | 1.000 | 1381 | tags=58%, list=24%, signal=76% |
| 170 | GENTILE\_RESPONSE\_CLUSTER\_D3 |  | 26 | 0.38 | 1.32 | 0.107 | 0.181 | 1.000 | 316 | tags=19%, list=6%, signal=20% |
| 171 | REACTOME\_VIF\_MEDIATED\_DEGRADATION\_OF\_APOBEC3G |  | 30 | 0.39 | 1.31 | 0.101 | 0.182 | 1.000 | 2337 | tags=67%, list=41%, signal=112% |
| 172 | REACTOME\_MRNA\_SPLICING\_MINOR\_PATHWAY |  | 23 | 0.40 | 1.31 | 0.118 | 0.181 | 1.000 | 1968 | tags=61%, list=34%, signal=92% |
| 173 | NAKAMURA\_CANCER\_MICROENVIRONMENT\_DN |  | 23 | 0.41 | 1.31 | 0.145 | 0.181 | 1.000 | 1184 | tags=52%, list=21%, signal=66% |
| 174 | DER\_IFN\_ALPHA\_RESPONSE\_UP |  | 22 | 0.41 | 1.31 | 0.105 | 0.183 | 1.000 | 612 | tags=27%, list=11%, signal=30% |
| 175 | SCHLOSSER\_MYC\_TARGETS\_AND\_SERUM\_RESPONSE\_DN |  | 27 | 0.39 | 1.31 | 0.112 | 0.183 | 1.000 | 1161 | tags=52%, list=20%, signal=65% |
| 176 | SANA\_RESPONSE\_TO\_IFNG\_UP |  | 21 | 0.40 | 1.30 | 0.136 | 0.185 | 1.000 | 777 | tags=48%, list=14%, signal=55% |
| 177 | BOYAULT\_LIVER\_CANCER\_SUBCLASS\_G123\_UP |  | 20 | 0.41 | 1.28 | 0.120 | 0.209 | 1.000 | 1678 | tags=80%, list=29%, signal=113% |
| 178 | CHANG\_CORE\_SERUM\_RESPONSE\_UP |  | 32 | 0.35 | 1.27 | 0.140 | 0.212 | 1.000 | 1125 | tags=47%, list=20%, signal=58% |
| 179 | KAUFFMANN\_DNA\_REPLICATION\_GENES |  | 62 | 0.30 | 1.27 | 0.091 | 0.212 | 1.000 | 895 | tags=40%, list=16%, signal=47% |
| 180 | CHEMNITZ\_RESPONSE\_TO\_PROSTAGLANDIN\_E2\_UP |  | 61 | 0.32 | 1.27 | 0.053 | 0.218 | 1.000 | 909 | tags=41%, list=16%, signal=48% |
| 181 | SHIPP\_DLBCL\_VS\_FOLLICULAR\_LYMPHOMA\_UP |  | 19 | 0.41 | 1.26 | 0.146 | 0.224 | 1.000 | 554 | tags=47%, list=10%, signal=52% |
| 182 | BERENJENO\_TRANSFORMED\_BY\_RHOA\_UP |  | 262 | 0.28 | 1.26 | 0.000 | 0.227 | 1.000 | 910 | tags=35%, list=16%, signal=40% |
| 183 | REACTOME\_REGULATION\_OF\_BETA\_CELL\_DEVELOPMENT |  | 19 | 0.39 | 1.25 | 0.123 | 0.229 | 1.000 | 1617 | tags=79%, list=28%, signal=110% |
| 184 | TARTE\_PLASMA\_CELL\_VS\_PLASMABLAST\_DN |  | 180 | 0.29 | 1.25 | 0.000 | 0.234 | 1.000 | 1197 | tags=39%, list=21%, signal=48% |
| 185 | REACTOME\_FORMATION\_OF\_THE\_EARLY\_ELONGATION\_COMPLEX |  | 18 | 0.40 | 1.25 | 0.169 | 0.232 | 1.000 | 2024 | tags=78%, list=35%, signal=120% |
| 186 | KEGG\_BUTANOATE\_METABOLISM |  | 18 | 0.40 | 1.25 | 0.145 | 0.234 | 1.000 | 1064 | tags=39%, list=19%, signal=48% |
| 187 | REACTOME\_METABLISM\_OF\_NUCLEOTIDES |  | 31 | 0.33 | 1.24 | 0.115 | 0.233 | 1.000 | 1016 | tags=52%, list=18%, signal=62% |
| 188 | FARMER\_BREAST\_CANCER\_CLUSTER\_1 |  | 23 | 0.37 | 1.24 | 0.172 | 0.233 | 1.000 | 334 | tags=39%, list=6%, signal=41% |
| 189 | MORI\_EMU\_MYC\_LYMPHOMA\_BY\_ONSET\_TIME\_UP |  | 48 | 0.31 | 1.24 | 0.127 | 0.236 | 1.000 | 1100 | tags=35%, list=19%, signal=43% |
| 190 | REACTOME\_LOSS\_OF\_NLP\_FROM\_MITOTIC\_CENTROSOMES |  | 36 | 0.34 | 1.24 | 0.129 | 0.240 | 1.000 | 1222 | tags=25%, list=21%, signal=32% |
| 191 | LINDGREN\_BLADDER\_CANCER\_CLUSTER\_3\_UP |  | 166 | 0.26 | 1.23 | 0.200 | 0.242 | 1.000 | 903 | tags=34%, list=16%, signal=40% |
| 192 | SEITZ\_NEOPLASTIC\_TRANSFORMATION\_BY\_8P\_DELETION\_UP |  | 29 | 0.35 | 1.23 | 0.179 | 0.246 | 1.000 | 325 | tags=34%, list=6%, signal=36% |
| 193 | RADAEVA\_RESPONSE\_TO\_IFNA1\_UP |  | 16 | 0.40 | 1.22 | 0.175 | 0.255 | 1.000 | 217 | tags=25%, list=4%, signal=26% |
| 194 | ELVIDGE\_HYPOXIA\_BY\_DMOG\_DN |  | 26 | 0.36 | 1.22 | 0.167 | 0.257 | 1.000 | 1197 | tags=38%, list=21%, signal=48% |
| 195 | BHATTACHARYA\_EMBRYONIC\_STEM\_CELL |  | 22 | 0.37 | 1.20 | 0.224 | 0.279 | 1.000 | 1197 | tags=50%, list=21%, signal=63% |
| 196 | POMEROY\_MEDULLOBLASTOMA\_PROGNOSIS\_DN |  | 17 | 0.40 | 1.20 | 0.215 | 0.282 | 1.000 | 1478 | tags=47%, list=26%, signal=63% |
| 197 | SASAKI\_ADULT\_T\_CELL\_LEUKEMIA |  | 63 | 0.27 | 1.18 | 0.075 | 0.299 | 1.000 | 1072 | tags=38%, list=19%, signal=46% |
| 198 | TCGA\_GLIOBLASTOMA\_COPY\_NUMBER\_DN |  | 18 | 0.37 | 1.18 | 0.214 | 0.303 | 1.000 | 768 | tags=28%, list=13%, signal=32% |
| 199 | REACTOME\_PURINE\_METABOLISM |  | 18 | 0.38 | 1.17 | 0.268 | 0.313 | 1.000 | 1441 | tags=67%, list=25%, signal=89% |
| 200 | BASAKI\_YBX1\_TARGETS\_UP |  | 131 | 0.27 | 1.15 | 0.222 | 0.345 | 1.000 | 567 | tags=28%, list=10%, signal=31% |
| 201 | LIN\_MELANOMA\_COPY\_NUMBER\_DN |  | 22 | 0.34 | 1.15 | 0.238 | 0.345 | 1.000 | 478 | tags=18%, list=8%, signal=20% |
| 202 | DAIRKEE\_CANCER\_PRONE\_RESPONSE\_BPA |  | 20 | 0.35 | 1.13 | 0.279 | 0.369 | 1.000 | 1712 | tags=55%, list=30%, signal=78% |
| 203 | CREIGHTON\_ENDOCRINE\_THERAPY\_RESISTANCE\_2 |  | 113 | 0.25 | 1.13 | 0.167 | 0.370 | 1.000 | 430 | tags=18%, list=8%, signal=19% |
| 204 | REACTOME\_METABOLISM\_OF\_CARBOHYDRATES |  | 45 | 0.29 | 1.13 | 0.197 | 0.369 | 1.000 | 1398 | tags=40%, list=24%, signal=53% |
| 205 | CHIANG\_LIVER\_CANCER\_SUBCLASS\_UNANNOTATED\_DN |  | 88 | 0.26 | 1.13 | 0.280 | 0.372 | 1.000 | 1352 | tags=49%, list=24%, signal=63% |
| 206 | DER\_IFN\_BETA\_RESPONSE\_UP |  | 38 | 0.31 | 1.12 | 0.281 | 0.374 | 1.000 | 612 | tags=18%, list=11%, signal=20% |
| 207 | REACTOME\_METABOLISM\_OF\_PROTEINS |  | 79 | 0.26 | 1.12 | 0.200 | 0.375 | 1.000 | 1820 | tags=56%, list=32%, signal=81% |
| 208 | PAL\_PRMT5\_TARGETS\_UP |  | 101 | 0.25 | 1.12 | 0.222 | 0.379 | 1.000 | 903 | tags=32%, list=16%, signal=37% |
| 209 | ELVIDGE\_HIF1A\_AND\_HIF2A\_TARGETS\_UP |  | 22 | 0.34 | 1.12 | 0.295 | 0.377 | 1.000 | 496 | tags=23%, list=9%, signal=25% |
| 210 | REACTOME\_MRNA\_PROCESSING |  | 16 | 0.37 | 1.12 | 0.267 | 0.383 | 1.000 | 2007 | tags=75%, list=35%, signal=115% |
| 211 | REACTOME\_HIV1\_TRANSCRIPTION\_ELONGATION |  | 24 | 0.34 | 1.11 | 0.281 | 0.384 | 1.000 | 2024 | tags=71%, list=35%, signal=109% |
| 212 | DOANE\_BREAST\_CANCER\_CLASSES\_UP |  | 16 | 0.37 | 1.11 | 0.305 | 0.395 | 1.000 | 34 | tags=13%, list=1%, signal=13% |
| 213 | KEGG\_P53\_SIGNALING\_PATHWAY |  | 39 | 0.28 | 1.10 | 0.284 | 0.400 | 1.000 | 983 | tags=41%, list=17%, signal=49% |
| 214 | KEGG\_OXIDATIVE\_PHOSPHORYLATION |  | 56 | 0.28 | 1.10 | 0.259 | 0.411 | 1.000 | 1569 | tags=45%, list=27%, signal=61% |
| 215 | VANHARANTA\_UTERINE\_FIBROID\_WITH\_7Q\_DELETION\_UP |  | 41 | 0.28 | 1.09 | 0.305 | 0.424 | 1.000 | 1304 | tags=37%, list=23%, signal=47% |
| 216 | REACTOME\_MTOR\_SIGNALLING |  | 15 | 0.37 | 1.07 | 0.352 | 0.454 | 1.000 | 351 | tags=20%, list=6%, signal=21% |
| 217 | BACOLOD\_RESISTANCE\_TO\_ALKYLATING\_AGENTS\_DN |  | 24 | 0.32 | 1.07 | 0.318 | 0.455 | 1.000 | 1010 | tags=33%, list=18%, signal=40% |
| 218 | TOOKER\_GEMCITABINE\_RESISTANCE\_UP |  | 43 | 0.28 | 1.06 | 0.345 | 0.467 | 1.000 | 1403 | tags=44%, list=25%, signal=58% |
| 219 | STEIN\_ESR1\_TARGETS |  | 38 | 0.28 | 1.06 | 0.370 | 0.467 | 1.000 | 895 | tags=47%, list=16%, signal=56% |
| 220 | TOOKER\_RESPONSE\_TO\_BEXAROTENE\_DN |  | 43 | 0.28 | 1.06 | 0.375 | 0.467 | 1.000 | 1403 | tags=44%, list=25%, signal=58% |
| 221 | JAERVINEN\_AMPLIFIED\_IN\_LARYNGEAL\_CANCER |  | 17 | 0.33 | 1.05 | 0.371 | 0.481 | 1.000 | 964 | tags=35%, list=17%, signal=42% |
| 222 | PASQUALUCCI\_LYMPHOMA\_BY\_GC\_STAGE\_DN |  | 72 | 0.25 | 1.04 | 0.371 | 0.495 | 1.000 | 1112 | tags=28%, list=19%, signal=34% |
| 223 | ELVIDGE\_HIF1A\_TARGETS\_UP |  | 38 | 0.28 | 1.03 | 0.402 | 0.524 | 1.000 | 635 | tags=24%, list=11%, signal=26% |
| 224 | GRABARCZYK\_BCL11B\_TARGETS\_DN |  | 22 | 0.31 | 1.02 | 0.423 | 0.527 | 1.000 | 363 | tags=18%, list=6%, signal=19% |
| 225 | RODWELL\_AGING\_KIDNEY\_DN |  | 41 | 0.27 | 1.02 | 0.415 | 0.534 | 1.000 | 660 | tags=20%, list=12%, signal=22% |
| 226 | LEE\_LIVER\_CANCER\_SURVIVAL\_DN |  | 56 | 0.25 | 1.02 | 0.457 | 0.533 | 1.000 | 1123 | tags=43%, list=20%, signal=53% |
| 227 | DANG\_REGULATED\_BY\_MYC\_UP |  | 30 | 0.30 | 1.01 | 0.441 | 0.551 | 1.000 | 972 | tags=33%, list=17%, signal=40% |
| 228 | CHOI\_ATL\_STAGE\_PREDICTOR |  | 15 | 0.33 | 0.99 | 0.484 | 0.596 | 1.000 | 484 | tags=27%, list=8%, signal=29% |
| 229 | KLEIN\_TARGETS\_OF\_BCR\_ABL1\_FUSION |  | 15 | 0.35 | 0.99 | 0.476 | 0.598 | 1.000 | 690 | tags=40%, list=12%, signal=45% |
| 230 | KEGG\_TRYPTOPHAN\_METABOLISM |  | 19 | 0.32 | 0.99 | 0.510 | 0.597 | 1.000 | 89 | tags=16%, list=2%, signal=16% |
| 231 | DAZARD\_UV\_RESPONSE\_CLUSTER\_G6 |  | 64 | 0.24 | 0.98 | 0.574 | 0.605 | 1.000 | 590 | tags=17%, list=10%, signal=19% |
| 232 | KESHELAVA\_MULTIPLE\_DRUG\_RESISTANCE |  | 29 | 0.27 | 0.98 | 0.527 | 0.610 | 1.000 | 346 | tags=17%, list=6%, signal=18% |
| 233 | GAZDA\_DIAMOND\_BLACKFAN\_ANEMIA\_PROGENITOR\_DN |  | 38 | 0.26 | 0.97 | 0.524 | 0.617 | 1.000 | 1275 | tags=42%, list=22%, signal=54% |
| 234 | ZHAN\_VARIABLE\_EARLY\_DIFFERENTIATION\_GENES\_DN |  | 18 | 0.31 | 0.97 | 0.522 | 0.620 | 1.000 | 1946 | tags=50%, list=34%, signal=76% |
| 235 | WEST\_ADRENOCORTICAL\_TUMOR\_UP |  | 145 | 0.21 | 0.97 | 0.750 | 0.628 | 1.000 | 846 | tags=21%, list=15%, signal=24% |
| 236 | REACTOME\_RNA\_POLYMERASE\_I\_III\_AND\_MITOCHONDRIAL\_TRANSCRIPTION |  | 33 | 0.26 | 0.97 | 0.519 | 0.627 | 1.000 | 1732 | tags=52%, list=30%, signal=73% |
| 237 | DAZARD\_RESPONSE\_TO\_UV\_NHEK\_DN |  | 140 | 0.18 | 0.95 | 0.800 | 0.649 | 1.000 | 706 | tags=15%, list=12%, signal=17% |
| 238 | REACTOME\_BRANCHED\_CHAIN\_AMINO\_ACID\_CATABOLISM |  | 16 | 0.31 | 0.95 | 0.527 | 0.661 | 1.000 | 1083 | tags=31%, list=19%, signal=38% |
| 239 | FAELT\_B\_CLL\_WITH\_VH\_REARRANGEMENTS\_DN |  | 25 | 0.29 | 0.95 | 0.472 | 0.659 | 1.000 | 696 | tags=24%, list=12%, signal=27% |
| 240 | LAU\_APOPTOSIS\_CDKN2A\_UP |  | 26 | 0.28 | 0.95 | 0.539 | 0.657 | 1.000 | 1332 | tags=38%, list=23%, signal=50% |
| 241 | KEGG\_OOCYTE\_MEIOSIS |  | 49 | 0.25 | 0.95 | 0.591 | 0.655 | 1.000 | 691 | tags=16%, list=12%, signal=18% |
| 242 | LEE\_LIVER\_CANCER\_CIPROFIBRATE\_DN |  | 16 | 0.31 | 0.95 | 0.527 | 0.653 | 1.000 | 549 | tags=31%, list=10%, signal=34% |
| 243 | DAZARD\_RESPONSE\_TO\_UV\_SCC\_DN |  | 47 | 0.25 | 0.94 | 0.657 | 0.658 | 1.000 | 653 | tags=15%, list=11%, signal=17% |
| 244 | KEGG\_RIG\_I\_LIKE\_RECEPTOR\_SIGNALING\_PATHWAY |  | 33 | 0.26 | 0.94 | 0.587 | 0.666 | 1.000 | 304 | tags=12%, list=5%, signal=13% |
| 245 | BECKER\_TAMOXIFEN\_RESISTANCE\_UP |  | 15 | 0.31 | 0.93 | 0.575 | 0.689 | 1.000 | 444 | tags=27%, list=8%, signal=29% |
| 246 | REACTOME\_STEROID\_METABOLISM |  | 22 | 0.28 | 0.92 | 0.584 | 0.700 | 1.000 | 520 | tags=18%, list=9%, signal=20% |
| 247 | REACTOME\_CYTOSOLIC\_TRNA\_AMINOACYLATION |  | 19 | 0.29 | 0.92 | 0.581 | 0.702 | 1.000 | 1713 | tags=47%, list=30%, signal=67% |
| 248 | NUNODA\_RESPONSE\_TO\_DASATINIB\_IMATINIB\_UP |  | 18 | 0.29 | 0.90 | 0.588 | 0.738 | 1.000 | 691 | tags=33%, list=12%, signal=38% |
| 249 | HOSHIDA\_LIVER\_CANCER\_SURVIVAL\_DN |  | 45 | 0.24 | 0.89 | 0.712 | 0.752 | 1.000 | 846 | tags=27%, list=15%, signal=31% |
| 250 | MARTINEZ\_RESPONSE\_TO\_TRABECTEDIN\_DN |  | 129 | 0.19 | 0.88 | 0.667 | 0.777 | 1.000 | 985 | tags=24%, list=17%, signal=28% |
| 251 | REACTOME\_SIGNALING\_BY\_WNT |  | 36 | 0.23 | 0.86 | 0.675 | 0.804 | 1.000 | 4395 | tags=100%, list=77%, signal=428% |
| 252 | TURASHVILI\_BREAST\_NORMAL\_DUCTAL\_VS\_LOBULAR\_UP |  | 25 | 0.26 | 0.86 | 0.656 | 0.803 | 1.000 | 755 | tags=28%, list=13%, signal=32% |
| 253 | DER\_IFN\_GAMMA\_RESPONSE\_UP |  | 19 | 0.28 | 0.86 | 0.659 | 0.802 | 1.000 | 612 | tags=21%, list=11%, signal=23% |
| 254 | WONG\_PROTEASOME\_GENE\_MODULE |  | 30 | 0.24 | 0.86 | 0.683 | 0.803 | 1.000 | 1734 | tags=53%, list=30%, signal=76% |
| 255 | LIU\_COMMON\_CANCER\_GENES |  | 20 | 0.26 | 0.84 | 0.746 | 0.835 | 1.000 | 983 | tags=35%, list=17%, signal=42% |
| 256 | SANA\_RESPONSE\_TO\_IFNG\_DN |  | 28 | 0.24 | 0.83 | 0.754 | 0.848 | 1.000 | 1153 | tags=50%, list=20%, signal=62% |
| 257 | LEE\_LIVER\_CANCER\_MYC\_E2F1\_DN |  | 21 | 0.25 | 0.83 | 0.727 | 0.848 | 1.000 | 786 | tags=33%, list=14%, signal=38% |
| 258 | OUELLET\_OVARIAN\_CANCER\_INVASIVE\_VS\_LMP\_UP |  | 67 | 0.20 | 0.83 | 0.909 | 0.849 | 1.000 | 1153 | tags=33%, list=20%, signal=41% |
| 259 | JISON\_SICKLE\_CELL\_DISEASE\_DN |  | 64 | 0.20 | 0.82 | 0.846 | 0.857 | 1.000 | 1548 | tags=42%, list=27%, signal=57% |
| 260 | BIOCARTA\_CHREBP2\_PATHWAY |  | 19 | 0.26 | 0.82 | 0.747 | 0.864 | 1.000 | 47 | tags=5%, list=1%, signal=5% |
| 261 | SCHLOSSER\_MYC\_TARGETS\_AND\_SERUM\_RESPONSE\_UP |  | 26 | 0.24 | 0.81 | 0.756 | 0.865 | 1.000 | 1687 | tags=58%, list=29%, signal=81% |
| 262 | KEGG\_VALINE\_LEUCINE\_AND\_ISOLEUCINE\_DEGRADATION |  | 34 | 0.22 | 0.81 | 0.813 | 0.863 | 1.000 | 1176 | tags=32%, list=21%, signal=40% |
| 263 | GINESTIER\_BREAST\_CANCER\_20Q13\_AMPLIFICATION\_DN |  | 75 | 0.20 | 0.81 | 0.931 | 0.871 | 1.000 | 1425 | tags=35%, list=25%, signal=46% |
| 264 | KEGG\_CARDIAC\_MUSCLE\_CONTRACTION |  | 20 | 0.25 | 0.79 | 0.758 | 0.888 | 1.000 | 1399 | tags=40%, list=24%, signal=53% |
| 265 | GALE\_APL\_WITH\_FLT3\_MUTATED\_UP |  | 32 | 0.22 | 0.79 | 0.817 | 0.895 | 1.000 | 1111 | tags=31%, list=19%, signal=39% |
| 266 | REACTOME\_REGULATION\_OF\_INSULIN\_SECRETION |  | 85 | 0.18 | 0.78 | 0.850 | 0.894 | 1.000 | 1338 | tags=33%, list=23%, signal=42% |
| 267 | VANTVEER\_BREAST\_CANCER\_BRCA1\_UP |  | 21 | 0.24 | 0.77 | 0.852 | 0.910 | 1.000 | 502 | tags=24%, list=9%, signal=26% |
| 268 | REACTOME\_ASSOCIATION\_OF\_TRIC\_CCT\_WITH\_TARGET\_PROTEINS\_DURING\_BIOSYNTHESIS |  | 16 | 0.26 | 0.77 | 0.815 | 0.908 | 1.000 | 964 | tags=31%, list=17%, signal=37% |
| 269 | YAO\_TEMPORAL\_RESPONSE\_TO\_PROGESTERONE\_CLUSTER\_17 |  | 100 | 0.17 | 0.76 | 1.000 | 0.925 | 1.000 | 1333 | tags=35%, list=23%, signal=45% |
| 270 | TCGA\_GLIOBLASTOMA\_COPY\_NUMBER\_UP |  | 38 | 0.20 | 0.75 | 0.909 | 0.933 | 1.000 | 1428 | tags=39%, list=25%, signal=52% |
| 271 | KEGG\_CYTOSOLIC\_DNA\_SENSING\_PATHWAY |  | 20 | 0.22 | 0.74 | 0.874 | 0.940 | 1.000 | 905 | tags=25%, list=16%, signal=30% |
| 272 | CAFFAREL\_RESPONSE\_TO\_THC\_24HR\_5\_UP |  | 15 | 0.25 | 0.74 | 0.845 | 0.939 | 1.000 | 1496 | tags=47%, list=26%, signal=63% |
| 273 | RODWELL\_AGING\_KIDNEY\_NO\_BLOOD\_DN |  | 45 | 0.19 | 0.72 | 0.921 | 0.957 | 1.000 | 660 | tags=18%, list=12%, signal=20% |
| 274 | REACTOME\_CHAPERONIN\_MEDIATED\_PROTEIN\_FOLDING |  | 19 | 0.23 | 0.71 | 0.917 | 0.958 | 1.000 | 964 | tags=26%, list=17%, signal=32% |
| 275 | KIM\_WT1\_TARGETS\_8HR\_DN |  | 37 | 0.19 | 0.70 | 0.949 | 0.962 | 1.000 | 1176 | tags=24%, list=21%, signal=30% |
| 276 | NOUZOVA\_TRETINOIN\_AND\_H4\_ACETYLATION |  | 66 | 0.17 | 0.70 | 1.000 | 0.960 | 1.000 | 1647 | tags=38%, list=29%, signal=53% |
| 277 | REACTOME\_TAT\_MEDIATED\_HIV1\_ELONGATION\_ARREST\_AND\_RECOVERY |  | 18 | 0.22 | 0.69 | 0.931 | 0.968 | 1.000 | 2024 | tags=61%, list=35%, signal=94% |
| 278 | YAO\_TEMPORAL\_RESPONSE\_TO\_PROGESTERONE\_CLUSTER\_13 |  | 90 | 0.16 | 0.68 | 1.000 | 0.968 | 1.000 | 1861 | tags=44%, list=33%, signal=65% |
| 279 | ROME\_INSULIN\_TARGETS\_IN\_MUSCLE\_UP |  | 48 | 0.18 | 0.68 | 0.962 | 0.970 | 1.000 | 1258 | tags=38%, list=22%, signal=48% |
| 280 | DING\_LUNG\_CANCER\_EXPRESSION\_BY\_COPY\_NUMBER |  | 59 | 0.16 | 0.62 | 0.977 | 1.000 | 1.000 | 1792 | tags=42%, list=31%, signal=61% |
| 281 | NIKOLSKY\_BREAST\_CANCER\_16Q24\_AMPLICON |  | 19 | 0.20 | 0.62 | 0.964 | 0.999 | 1.000 | 1849 | tags=58%, list=32%, signal=85% |
| 282 | NIKOLSKY\_BREAST\_CANCER\_16P13\_AMPLICON |  | 18 | 0.19 | 0.58 | 0.965 | 1.000 | 1.000 | 120 | tags=6%, list=2%, signal=6% |
| 283 | KEGG\_GLYCOLYSIS\_GLUCONEOGENESIS |  | 15 | 0.18 | 0.54 | 0.966 | 1.000 | 1.000 | 789 | tags=20%, list=14%, signal=23% |
| 284 | RODRIGUES\_THYROID\_CARCINOMA\_POORLY\_DIFFERENTIATED\_UP |  | 333 | 0.29 |  |  | 1.000 | 0.000 | 1323 | tags=42%, list=23%, signal=52% |
| 285 | RODRIGUES\_THYROID\_CARCINOMA\_ANAPLASTIC\_UP |  | 359 | 0.22 |  |  | 1.000 | 0.000 | 1068 | tags=28%, list=19%, signal=32% |
| 286 | PUJANA\_BRCA2\_PCC\_NETWORK |  | 248 | 0.37 |  |  | 1.000 | 0.000 | 1031 | tags=40%, list=18%, signal=46% |
| 287 | PUJANA\_CHEK2\_PCC\_NETWORK |  | 407 | 0.45 |  |  | 1.000 | 0.000 | 1412 | tags=49%, list=25%, signal=60% |
| 288 | KAUFFMANN\_MELANOMA\_RELAPSE\_UP |  | 28 | 0.78 | 2.67 | 0.000 | 0.000 | 0.000 | 895 | tags=71%, list=16%, signal=84% |
| 289 | PUJANA\_BRCA\_CENTERED\_NETWORK |  | 64 | 0.60 | 2.53 | 0.000 | 0.000 | 0.000 | 1678 | tags=69%, list=29%, signal=96% |
| 290 | ALCALA\_APOPTOSIS |  | 38 | 0.38 | 1.38 | 0.024 | 0.015 | 1.000 | 1554 | tags=47%, list=27%, signal=65% |
| 291 | NUYTTEN\_EZH2\_TARGETS\_DN |  | 413 | 0.26 |  |  | 1.000 | 0.000 | 583 | tags=21%, list=10%, signal=22% |
| 292 | WEI\_MYCN\_TARGETS\_WITH\_E\_BOX |  | 412 | 0.36 |  |  | 1.000 | 0.000 | 1229 | tags=39%, list=21%, signal=46% |
| 293 | BENPORATH\_MYC\_MAX\_TARGETS |  | 424 | 0.16 |  |  | 1.000 | 0.000 | 1129 | tags=23%, list=20%, signal=26% |
| 294 | BENPORATH\_CYCLING\_GENES |  | 306 | 0.30 |  |  | 1.000 | 0.000 | 580 | tags=25%, list=10%, signal=26% |
| 295 | BENPORATH\_PROLIFERATION |  | 79 | 0.43 | 1.89 | 0.000 | 0.000 | 0.317 | 1093 | tags=52%, list=19%, signal=63% |
| 296 | ZHANG\_BREAST\_CANCER\_PROGENITORS\_UP |  | 216 | 0.31 |  |  | 1.000 | 0.000 | 1122 | tags=32%, list=20%, signal=39% |
| 297 | MILI\_PSEUDOPODIA\_HAPTOTAXIS\_UP |  | 256 | 0.17 |  |  | 1.000 | 0.000 | 1772 | tags=39%, list=31%, signal=55% |
| 298 | CAIRO\_HEPATOBLASTOMA\_CLASSES\_UP |  | 310 | 0.38 |  |  | 1.000 | 0.000 | 1316 | tags=47%, list=23%, signal=57% |
| 299 | WINNEPENNINCKX\_MELANOMA\_METASTASIS\_UP |  | 86 | 0.56 | 2.44 | 0.000 | 0.000 | 0.001 | 971 | tags=52%, list=17%, signal=62% |
| 300 | WONG\_EMBRYONIC\_STEM\_CELL\_CORE |  | 186 | 0.55 |  |  | 1.000 | 0.000 | 1234 | tags=56%, list=22%, signal=70% |
| 301 | NAKAYAMA\_SOFT\_TISSUE\_TUMORS\_PCA2\_UP |  | 30 | 0.36 | 1.26 | 0.116 | 0.026 | 1.000 | 704 | tags=67%, list=12%, signal=76% |
| 302 | MOOTHA\_HUMAN\_MITODB\_6\_2002 |  | 206 | 0.32 |  |  | 1.000 | 0.000 | 1343 | tags=42%, list=23%, signal=53% |
| 303 | MOOTHA\_MITOCHONDRIA |  | 208 | 0.32 |  |  | 1.000 | 0.000 | 1399 | tags=44%, list=24%, signal=56% |
| 304 | REACTOME\_GENE\_EXPRESSION |  | 204 | 0.27 |  |  | 1.000 | 0.000 | 1994 | tags=60%, list=35%, signal=89% |
| 305 | REACTOME\_GLOBAL\_GENOMIC\_NER |  | 23 | 0.51 | 1.67 | 0.028 | 0.002 | 0.880 | 1263 | tags=65%, list=22%, signal=83% |
| 306 | REACTOME\_GENERIC\_TRANSCRIPTION\_PATHWAY |  | 16 | 0.45 | 1.34 | 0.149 | 0.017 | 1.000 | 1418 | tags=44%, list=25%, signal=58% |
Table: Gene sets enriched in phenotype **LymphomaArray (4 samples)**[plain text format]****

  
