# Supplementary material for: Comparative RNA-Seq and Microarray Analysis of Gene Expression Changes in B-Cell Lymphomas of Canis familiaris
Source: PLoS One. 2013 Apr 4;8(4):e61088. doi: 10.1371/journal.pone.0061088 (PMC3617154; doi:10.1371/journal.pone.0061088)
Supplement: Data File S2 — GSEA Results Files. (ZIP) [file pone.0061088.s005.zip › Array/gsea_report_for_LymphomaMicroarray_v2.html]

Report for LymphomaMicroarray 1334779850806 [GSEA]

| GS  follow link to MSigDB | GS DETAILS | SIZE | ES | NES | NOM p-val | FDR q-val | FWER p-val | RANK AT MAX | LEADING EDGE || 1 | SERUM\_FIBROBLAST\_CELLCYCLE | Details ... | 58 | 0.64 | 2.63 | 0.000 | 0.000 | 0.000 | 984 | tags=67%, list=17%, signal=80% |
| 2 | ZHAN\_MM\_CD138\_PR\_VS\_REST | Details ... | 16 | 0.84 | 2.56 | 0.000 | 0.000 | 0.000 | 423 | tags=75%, list=7%, signal=81% |
| 3 | CMV\_IE86\_UP | Details ... | 29 | 0.72 | 2.52 | 0.000 | 0.000 | 0.000 | 1298 | tags=86%, list=23%, signal=111% |
| 4 | CROONQUIST\_IL6\_STARVE\_UP | Details ... | 15 | 0.85 | 2.42 | 0.000 | 0.000 | 0.000 | 705 | tags=87%, list=12%, signal=99% |
| 5 | SHEPARD\_GENES\_COMMON\_BW\_CB\_MO | Details ... | 21 | 0.73 | 2.39 | 0.000 | 0.000 | 0.001 | 467 | tags=62%, list=8%, signal=67% |
| 6 | BRENTANI\_REPAIR | Details ... | 24 | 0.73 | 2.38 | 0.000 | 0.000 | 0.001 | 1160 | tags=79%, list=20%, signal=99% |
| 7 | DOX\_RESIST\_GASTRIC\_UP | Details ... | 19 | 0.77 | 2.36 | 0.000 | 0.000 | 0.001 | 1073 | tags=89%, list=19%, signal=110% |
| 8 | IDX\_TSA\_UP\_CLUSTER3 | Details ... | 43 | 0.61 | 2.32 | 0.000 | 0.000 | 0.002 | 754 | tags=63%, list=13%, signal=72% |
| 9 | ADIP\_DIFF\_CLUSTER4 | Details ... | 17 | 0.74 | 2.27 | 0.000 | 0.001 | 0.005 | 1450 | tags=94%, list=25%, signal=126% |
| 10 | HSA03010\_RIBOSOME | Details ... | 18 | 0.72 | 2.23 | 0.000 | 0.001 | 0.009 | 1618 | tags=100%, list=28%, signal=139% |
| 11 | LEE\_TCELLS3\_UP | Details ... | 35 | 0.60 | 2.22 | 0.000 | 0.001 | 0.009 | 705 | tags=69%, list=12%, signal=78% |
| 12 | MANALO\_HYPOXIA\_DN | Details ... | 45 | 0.58 | 2.22 | 0.000 | 0.001 | 0.011 | 1485 | tags=73%, list=26%, signal=98% |
| 13 | P21\_P53\_ANY\_DN | Details ... | 22 | 0.64 | 2.16 | 0.007 | 0.002 | 0.019 | 920 | tags=68%, list=16%, signal=81% |
| 14 | P21\_ANY\_DN | Details ... | 19 | 0.68 | 2.16 | 0.000 | 0.001 | 0.019 | 560 | tags=63%, list=10%, signal=70% |
| 15 | DNA\_REPLICATION\_REACTOME | Details ... | 24 | 0.64 | 2.14 | 0.000 | 0.002 | 0.024 | 971 | tags=75%, list=17%, signal=90% |
| 16 | ADIP\_DIFF\_CLUSTER5 | Details ... | 16 | 0.69 | 2.13 | 0.000 | 0.002 | 0.024 | 556 | tags=75%, list=10%, signal=83% |
| 17 | P21\_P53\_MIDDLE\_DN | Details ... | 15 | 0.74 | 2.13 | 0.000 | 0.002 | 0.025 | 1211 | tags=87%, list=21%, signal=110% |
| 18 | RIBOSOMAL\_PROTEINS | Details ... | 22 | 0.65 | 2.08 | 0.000 | 0.003 | 0.046 | 1618 | tags=82%, list=28%, signal=114% |
| 19 | YU\_CMYC\_UP | Details ... | 20 | 0.64 | 2.05 | 0.000 | 0.004 | 0.065 | 549 | tags=70%, list=10%, signal=77% |
| 20 | CANCER\_UNDIFFERENTIATED\_META\_UP | Details ... | 37 | 0.55 | 2.05 | 0.000 | 0.004 | 0.065 | 556 | tags=49%, list=10%, signal=54% |
| 21 | CELL\_CYCLE |  | 40 | 0.54 | 2.02 | 0.000 | 0.005 | 0.085 | 692 | tags=48%, list=12%, signal=54% |
| 22 | DNA\_DAMAGE\_SIGNALING |  | 51 | 0.49 | 1.99 | 0.000 | 0.006 | 0.110 | 1160 | tags=59%, list=20%, signal=73% |
| 23 | REN\_E2F1\_TARGETS |  | 25 | 0.61 | 1.97 | 0.000 | 0.006 | 0.120 | 1060 | tags=68%, list=19%, signal=83% |
| 24 | CELL\_CYCLE\_KEGG |  | 43 | 0.53 | 1.93 | 0.000 | 0.008 | 0.167 | 692 | tags=49%, list=12%, signal=55% |
| 25 | MOOTHA\_VOXPHOS |  | 35 | 0.51 | 1.92 | 0.000 | 0.009 | 0.184 | 1487 | tags=54%, list=26%, signal=73% |
| 26 | SHEPARD\_CRASH\_AND\_BURN\_MUT\_VS\_WT\_DN |  | 47 | 0.48 | 1.88 | 0.000 | 0.012 | 0.246 | 467 | tags=38%, list=8%, signal=41% |
| 27 | LI\_FETAL\_VS\_WT\_KIDNEY\_DN |  | 69 | 0.45 | 1.84 | 0.000 | 0.018 | 0.352 | 829 | tags=46%, list=14%, signal=54% |
| 28 | HSA04110\_CELL\_CYCLE |  | 59 | 0.45 | 1.81 | 0.000 | 0.022 | 0.430 | 692 | tags=39%, list=12%, signal=44% |
| 29 | MOREAUX\_TACI\_HI\_IN\_PPC\_UP |  | 33 | 0.50 | 1.78 | 0.000 | 0.025 | 0.487 | 1312 | tags=45%, list=23%, signal=59% |
| 30 | SCHUMACHER\_MYC\_UP |  | 22 | 0.53 | 1.72 | 0.013 | 0.037 | 0.626 | 1796 | tags=82%, list=31%, signal=119% |
| 31 | CANTHARIDIN\_DN |  | 21 | 0.53 | 1.68 | 0.006 | 0.048 | 0.739 | 1746 | tags=67%, list=31%, signal=96% |
| 32 | HUMAN\_MITODB\_6\_2002 |  | 184 | 0.34 | 1.67 | 0.000 | 0.051 | 0.776 | 1344 | tags=42%, list=23%, signal=54% |
| 33 | G1\_TO\_S\_CELL\_CYCLE\_REACTOME |  | 32 | 0.46 | 1.65 | 0.015 | 0.056 | 0.809 | 692 | tags=44%, list=12%, signal=49% |
| 34 | VERNELL\_PRB\_CLSTR1 |  | 35 | 0.44 | 1.63 | 0.010 | 0.061 | 0.843 | 910 | tags=60%, list=16%, signal=71% |
| 35 | BRENTANI\_CELL\_CYCLE |  | 40 | 0.44 | 1.63 | 0.023 | 0.060 | 0.847 | 692 | tags=43%, list=12%, signal=48% |
| 36 | MOREAUX\_TACI\_HI\_VS\_LOW\_DN |  | 94 | 0.35 | 1.60 | 0.000 | 0.070 | 0.901 | 1891 | tags=52%, list=33%, signal=77% |
| 37 | MRNA\_PROCESSING\_REACTOME |  | 50 | 0.38 | 1.52 | 0.033 | 0.111 | 0.982 | 1960 | tags=60%, list=34%, signal=90% |
| 38 | LE\_MYELIN\_UP |  | 43 | 0.39 | 1.49 | 0.018 | 0.125 | 0.991 | 754 | tags=44%, list=13%, signal=51% |
| 39 | ELECTRON\_TRANSPORT\_CHAIN |  | 46 | 0.38 | 1.48 | 0.000 | 0.129 | 0.992 | 2042 | tags=63%, list=36%, signal=97% |
| 40 | MITOCHONDRIA |  | 183 | 0.33 | 1.48 | 0.000 | 0.127 | 0.992 | 1344 | tags=44%, list=23%, signal=55% |
| 41 | BLEO\_MOUSE\_LYMPH\_HIGH\_24HRS\_DN |  | 18 | 0.48 | 1.48 | 0.034 | 0.126 | 0.992 | 1954 | tags=78%, list=34%, signal=118% |
| 42 | DNMT1\_KO\_UP |  | 25 | 0.43 | 1.46 | 0.046 | 0.137 | 0.995 | 273 | tags=20%, list=5%, signal=21% |
| 43 | HSA00970\_AMINOACYL\_TRNA\_BIOSYNTHESIS |  | 27 | 0.41 | 1.43 | 0.035 | 0.152 | 0.999 | 1049 | tags=37%, list=18%, signal=45% |
| 44 | SERUM\_FIBROBLAST\_CORE\_UP |  | 86 | 0.33 | 1.42 | 0.000 | 0.158 | 0.999 | 1140 | tags=43%, list=20%, signal=53% |
| 45 | RADAEVA\_IFNA\_UP |  | 18 | 0.47 | 1.40 | 0.096 | 0.174 | 0.999 | 217 | tags=33%, list=4%, signal=35% |
| 46 | IFNA\_HCMV\_6HRS\_UP |  | 18 | 0.45 | 1.39 | 0.090 | 0.180 | 0.999 | 448 | tags=39%, list=8%, signal=42% |
| 47 | TARTE\_PLASMA\_BLASTIC |  | 165 | 0.32 | 1.38 | 0.000 | 0.188 | 0.999 | 1198 | tags=41%, list=21%, signal=50% |
| 48 | UVC\_HIGH\_D3\_DN |  | 27 | 0.39 | 1.35 | 0.096 | 0.212 | 1.000 | 316 | tags=19%, list=6%, signal=20% |
| 49 | CELL\_CYCLE\_CHECKPOINT |  | 15 | 0.46 | 1.34 | 0.122 | 0.219 | 1.000 | 1333 | tags=80%, list=23%, signal=104% |
| 50 | HSA00240\_PYRIMIDINE\_METABOLISM |  | 45 | 0.35 | 1.32 | 0.073 | 0.237 | 1.000 | 971 | tags=44%, list=17%, signal=53% |
| 51 | RNA\_TRANSCRIPTION\_REACTOME |  | 19 | 0.41 | 1.32 | 0.102 | 0.233 | 1.000 | 1610 | tags=53%, list=28%, signal=73% |
| 52 | IFN\_ALPHA\_UP |  | 16 | 0.45 | 1.31 | 0.135 | 0.235 | 1.000 | 531 | tags=31%, list=9%, signal=34% |
| 53 | GLYCINE\_SERINE\_AND\_THREONINE\_METABOLISM |  | 15 | 0.44 | 1.30 | 0.141 | 0.246 | 1.000 | 660 | tags=40%, list=12%, signal=45% |
| 54 | GOLDRATH\_CELLCYCLE |  | 17 | 0.43 | 1.29 | 0.155 | 0.251 | 1.000 | 556 | tags=53%, list=10%, signal=58% |
| 55 | SANA\_IFNG\_ENDOTHELIAL\_UP |  | 19 | 0.41 | 1.29 | 0.148 | 0.247 | 1.000 | 778 | tags=47%, list=14%, signal=55% |
| 56 | ZHAN\_MULTIPLE\_MYELOMA\_SUBCLASSES\_DIFF |  | 16 | 0.45 | 1.29 | 0.172 | 0.249 | 1.000 | 1107 | tags=50%, list=19%, signal=62% |
| 57 | ROTH\_HTERT\_DIFF |  | 16 | 0.43 | 1.28 | 0.164 | 0.246 | 1.000 | 984 | tags=50%, list=17%, signal=60% |
| 58 | CANCER\_NEOPLASTIC\_META\_UP |  | 32 | 0.35 | 1.28 | 0.133 | 0.243 | 1.000 | 1178 | tags=41%, list=21%, signal=51% |
| 59 | HUMAN\_TISSUE\_TESTIS |  | 19 | 0.41 | 1.28 | 0.137 | 0.241 | 1.000 | 727 | tags=37%, list=13%, signal=42% |
| 60 | SHIPP\_FL\_VS\_DLBCL\_DN |  | 15 | 0.40 | 1.20 | 0.251 | 0.344 | 1.000 | 1129 | tags=67%, list=20%, signal=83% |
| 61 | SASAKI\_TCELL\_LYMPHOMA\_VS\_CD4\_UP |  | 71 | 0.28 | 1.19 | 0.098 | 0.354 | 1.000 | 1073 | tags=39%, list=19%, signal=48% |
| 62 | CHANG\_SERUM\_RESPONSE\_UP |  | 69 | 0.28 | 1.19 | 0.184 | 0.351 | 1.000 | 1119 | tags=39%, list=20%, signal=48% |
| 63 | HSA00380\_TRYPTOPHAN\_METABOLISM |  | 29 | 0.34 | 1.18 | 0.188 | 0.366 | 1.000 | 967 | tags=41%, list=17%, signal=50% |
| 64 | SASAKI\_ATL\_UP |  | 71 | 0.28 | 1.18 | 0.146 | 0.364 | 1.000 | 1073 | tags=39%, list=19%, signal=48% |
| 65 | DER\_IFNA\_UP |  | 27 | 0.35 | 1.16 | 0.244 | 0.380 | 1.000 | 613 | tags=26%, list=11%, signal=29% |
| 66 | HSA00280\_VALINE\_LEUCINE\_AND\_ISOLEUCINE\_DEGRADATION |  | 32 | 0.32 | 1.16 | 0.210 | 0.375 | 1.000 | 1177 | tags=38%, list=21%, signal=47% |
| 67 | DER\_IFNB\_UP |  | 41 | 0.29 | 1.12 | 0.256 | 0.443 | 1.000 | 613 | tags=20%, list=11%, signal=22% |
| 68 | TRYPTOPHAN\_METABOLISM |  | 16 | 0.38 | 1.12 | 0.290 | 0.442 | 1.000 | 89 | tags=19%, list=2%, signal=19% |
| 69 | HSA00790\_FOLATE\_BIOSYNTHESIS |  | 18 | 0.36 | 1.12 | 0.286 | 0.436 | 1.000 | 2056 | tags=61%, list=36%, signal=95% |
| 70 | ET743\_SARCOMA\_48HRS\_DN |  | 106 | 0.24 | 1.09 | 0.357 | 0.499 | 1.000 | 1950 | tags=52%, list=34%, signal=77% |
| 71 | ZHAN\_TONSIL\_PCBC |  | 21 | 0.32 | 1.08 | 0.318 | 0.499 | 1.000 | 474 | tags=24%, list=8%, signal=26% |
| 72 | PYRIMIDINE\_METABOLISM |  | 24 | 0.32 | 1.08 | 0.265 | 0.503 | 1.000 | 899 | tags=42%, list=16%, signal=49% |
| 73 | ZHAN\_MMPC\_EARLYVS |  | 23 | 0.32 | 1.08 | 0.353 | 0.498 | 1.000 | 1746 | tags=61%, list=31%, signal=87% |
| 74 | GALE\_FLT3ANDAPL\_UP |  | 23 | 0.32 | 1.08 | 0.374 | 0.494 | 1.000 | 701 | tags=30%, list=12%, signal=35% |
| 75 | MRNA\_SPLICING |  | 17 | 0.36 | 1.07 | 0.354 | 0.500 | 1.000 | 1842 | tags=47%, list=32%, signal=69% |
| 76 | O6BG\_RESIST\_MEDULLOBLASTOMA\_DN |  | 25 | 0.31 | 1.05 | 0.382 | 0.537 | 1.000 | 1011 | tags=32%, list=18%, signal=39% |
| 77 | IFN\_BETA\_UP |  | 29 | 0.30 | 1.05 | 0.352 | 0.533 | 1.000 | 531 | tags=17%, list=9%, signal=19% |
| 78 | HSA04115\_P53\_SIGNALING\_PATHWAY |  | 37 | 0.29 | 1.02 | 0.394 | 0.597 | 1.000 | 984 | tags=41%, list=17%, signal=49% |
| 79 | HSA00260\_GLYCINE\_SERINE\_AND\_THREONINE\_METABOLISM |  | 17 | 0.35 | 1.01 | 0.391 | 0.603 | 1.000 | 660 | tags=29%, list=12%, signal=33% |
| 80 | HCC\_SURVIVAL\_GOOD\_VS\_POOR\_DN |  | 56 | 0.25 | 1.01 | 0.452 | 0.602 | 1.000 | 1124 | tags=43%, list=20%, signal=53% |
| 81 | HSA00190\_OXIDATIVE\_PHOSPHORYLATION |  | 55 | 0.24 | 1.00 | 0.508 | 0.604 | 1.000 | 1772 | tags=49%, list=31%, signal=70% |
| 82 | ET743\_SARCOMA\_DN |  | 139 | 0.21 | 0.98 | 0.375 | 0.641 | 1.000 | 986 | tags=25%, list=17%, signal=30% |
| 83 | TRANSLATION\_FACTORS |  | 20 | 0.29 | 0.97 | 0.484 | 0.661 | 1.000 | 2124 | tags=75%, list=37%, signal=119% |
| 84 | HSA00650\_BUTANOATE\_METABOLISM |  | 24 | 0.29 | 0.96 | 0.532 | 0.673 | 1.000 | 1065 | tags=29%, list=19%, signal=36% |
| 85 | PENG\_LEUCINE\_DN |  | 79 | 0.23 | 0.94 | 0.704 | 0.724 | 1.000 | 1308 | tags=35%, list=23%, signal=45% |
| 86 | CELL\_CYCLE\_ARREST |  | 15 | 0.31 | 0.93 | 0.585 | 0.731 | 1.000 | 515 | tags=27%, list=9%, signal=29% |
| 87 | PARK\_MSCS\_BOTH |  | 17 | 0.30 | 0.91 | 0.556 | 0.758 | 1.000 | 419 | tags=18%, list=7%, signal=19% |
| 88 | LEE\_CIP\_DN |  | 16 | 0.31 | 0.90 | 0.559 | 0.787 | 1.000 | 550 | tags=31%, list=10%, signal=34% |
| 89 | ET743\_SARCOMA\_72HRS\_DN |  | 116 | 0.19 | 0.89 | 0.667 | 0.803 | 1.000 | 1287 | tags=32%, list=22%, signal=40% |
| 90 | IFN\_ANY\_UP |  | 33 | 0.25 | 0.88 | 0.683 | 0.814 | 1.000 | 304 | tags=12%, list=5%, signal=13% |
| 91 | DER\_IFNG\_UP |  | 20 | 0.28 | 0.87 | 0.661 | 0.809 | 1.000 | 782 | tags=25%, list=14%, signal=29% |
| 92 | ET743\_SARCOMA\_24HRS\_DN |  | 62 | 0.21 | 0.86 | 0.745 | 0.823 | 1.000 | 1924 | tags=47%, list=34%, signal=70% |
| 93 | HYPOXIA\_RCC\_UP |  | 40 | 0.23 | 0.86 | 0.762 | 0.823 | 1.000 | 1033 | tags=38%, list=18%, signal=45% |
| 94 | UVB\_SCC\_DN |  | 50 | 0.20 | 0.85 | 0.815 | 0.838 | 1.000 | 654 | tags=14%, list=11%, signal=16% |
| 95 | UVB\_NHEK2\_UP |  | 20 | 0.26 | 0.84 | 0.733 | 0.846 | 1.000 | 1539 | tags=55%, list=27%, signal=75% |
| 96 | LEE\_MYC\_E2F1\_DN |  | 21 | 0.25 | 0.83 | 0.721 | 0.856 | 1.000 | 787 | tags=33%, list=14%, signal=39% |
| 97 | APOPTOSIS |  | 27 | 0.25 | 0.83 | 0.740 | 0.847 | 1.000 | 474 | tags=15%, list=8%, signal=16% |
| 98 | HSA00310\_LYSINE\_DEGRADATION |  | 27 | 0.23 | 0.80 | 0.832 | 0.892 | 1.000 | 882 | tags=30%, list=15%, signal=35% |
| 99 | AGEING\_KIDNEY\_DN |  | 49 | 0.21 | 0.79 | 0.859 | 0.886 | 1.000 | 661 | tags=18%, list=12%, signal=21% |
| 100 | PENG\_GLUTAMINE\_DN |  | 131 | 0.17 | 0.79 | 1.000 | 0.882 | 1.000 | 1646 | tags=40%, list=29%, signal=54% |
| 101 | HSA00251\_GLUTAMATE\_METABOLISM |  | 15 | 0.27 | 0.78 | 0.764 | 0.885 | 1.000 | 2102 | tags=67%, list=37%, signal=105% |
| 102 | HSA04120\_UBIQUITIN\_MEDIATED\_PROTEOLYSIS |  | 26 | 0.22 | 0.77 | 0.849 | 0.892 | 1.000 | 1488 | tags=38%, list=26%, signal=52% |
| 103 | KREBS\_TCA\_CYCLE |  | 18 | 0.25 | 0.76 | 0.822 | 0.894 | 1.000 | 905 | tags=22%, list=16%, signal=26% |
| 104 | FALT\_BCLL\_IG\_MUTATED\_VS\_WT\_DN |  | 22 | 0.22 | 0.70 | 0.860 | 0.954 | 1.000 | 697 | tags=23%, list=12%, signal=26% |
| 105 | TRNA\_SYNTHETASES |  | 15 | 0.25 | 0.70 | 0.855 | 0.949 | 1.000 | 4324 | tags=100%, list=76%, signal=408% |
| 106 | AMINOACYL\_TRNA\_BIOSYNTHESIS |  | 16 | 0.23 | 0.68 | 0.901 | 0.959 | 1.000 | 4401 | tags=100%, list=77%, signal=431% |
| 107 | KUROKAWA\_5FU\_IFN\_SENSITIVE\_VS\_RESISTANT\_DN |  | 16 | 0.23 | 0.68 | 0.870 | 0.950 | 1.000 | 49 | tags=6%, list=1%, signal=6% |
| 108 | JAIN\_NEMO\_DIFF |  | 36 | 0.18 | 0.66 | 0.980 | 0.957 | 1.000 | 1357 | tags=36%, list=24%, signal=47% |
| 109 | CMV\_HCMV\_TIMECOURSE\_18HRS\_UP |  | 33 | 0.18 | 0.65 | 0.970 | 0.954 | 1.000 | 4716 | tags=100%, list=82%, signal=565% |
| 110 | UV\_UNIQUE\_FIBRO\_DN |  | 15 | 0.16 | 0.45 | 1.000 | 0.999 | 1.000 | 4821 | tags=100%, list=84%, signal=632% |
Table: Gene sets enriched in phenotype **LymphomaMicroarray (4 samples)**[plain text format]****

  
