# Supplementary material for: Comparative RNA-Seq and Microarray Analysis of Gene Expression Changes in B-Cell Lymphomas of Canis familiaris
Source: PLoS One. 2013 Apr 4;8(4):e61088. doi: 10.1371/journal.pone.0061088 (PMC3617154; doi:10.1371/journal.pone.0061088)
Supplement: Data File S2 — GSEA Results Files. (ZIP) [file pone.0061088.s005.zip › Array/gsea_report_for_NormalMicroarray_v2.html]

Report for NormalMicroarray 1334779850806 [GSEA]

| GS  follow link to MSigDB | GS DETAILS | SIZE | ES | NES | NOM p-val | FDR q-val | FWER p-val | RANK AT MAX | LEADING EDGE || 1 | BOQUEST\_CD31PLUS\_VS\_CD31MINUS\_DN | Details ... | 88 | -0.81 | -2.27 | 0.000 | 0.000 | 0.000 | 820 | tags=78%, list=14%, signal=90% |
| 2 | BAF57\_BT549\_UP | Details ... | 84 | -0.72 | -2.06 | 0.000 | 0.000 | 0.000 | 709 | tags=56%, list=12%, signal=63% |
| 3 | LAL\_KO\_6MO\_UP | Details ... | 22 | -0.84 | -2.05 | 0.000 | 0.000 | 0.000 | 332 | tags=55%, list=6%, signal=58% |
| 4 | SANA\_TNFA\_ENDOTHELIAL\_DN | Details ... | 29 | -0.80 | -2.02 | 0.000 | 0.000 | 0.000 | 787 | tags=72%, list=14%, signal=84% |
| 5 | HSA04060\_CYTOKINE\_CYTOKINE\_RECEPTOR\_INTERACTION | Details ... | 66 | -0.73 | -2.01 | 0.000 | 0.000 | 0.000 | 942 | tags=67%, list=16%, signal=79% |
| 6 | LAL\_KO\_3MO\_UP | Details ... | 21 | -0.84 | -2.01 | 0.000 | 0.000 | 0.000 | 332 | tags=57%, list=6%, signal=60% |
| 7 | TAKEDA\_NUP8\_HOXA9\_10D\_DN | Details ... | 48 | -0.75 | -1.99 | 0.000 | 0.000 | 0.000 | 533 | tags=50%, list=9%, signal=55% |
| 8 | BOQUEST\_CD31PLUS\_VS\_CD31MINUS\_UP | Details ... | 236 | -0.68 | -1.98 | 0.000 | 0.000 | 0.000 | 974 | tags=52%, list=17%, signal=60% |
| 9 | HSA04512\_ECM\_RECEPTOR\_INTERACTION | Details ... | 30 | -0.78 | -1.97 | 0.000 | 0.000 | 0.000 | 811 | tags=77%, list=14%, signal=89% |
| 10 | TAKEDA\_NUP8\_HOXA9\_8D\_DN | Details ... | 71 | -0.71 | -1.97 | 0.000 | 0.000 | 0.000 | 725 | tags=46%, list=13%, signal=53% |
| 11 | CMV\_24HRS\_DN | Details ... | 36 | -0.75 | -1.96 | 0.000 | 0.000 | 0.000 | 957 | tags=72%, list=17%, signal=86% |
| 12 | HSA04514\_CELL\_ADHESION\_MOLECULES | Details ... | 42 | -0.74 | -1.95 | 0.000 | 0.000 | 0.001 | 1140 | tags=79%, list=20%, signal=97% |
| 13 | HSA04610\_COMPLEMENT\_AND\_COAGULATION\_CASCADES | Details ... | 15 | -0.87 | -1.94 | 0.000 | 0.000 | 0.001 | 469 | tags=80%, list=8%, signal=87% |
| 14 | HSA04510\_FOCAL\_ADHESION | Details ... | 87 | -0.69 | -1.93 | 0.000 | 0.000 | 0.001 | 1329 | tags=61%, list=23%, signal=78% |
| 15 | NI2\_MOUSE\_UP | Details ... | 21 | -0.81 | -1.91 | 0.000 | 0.000 | 0.001 | 647 | tags=62%, list=11%, signal=70% |
| 16 | HSA04640\_HEMATOPOIETIC\_CELL\_LINEAGE | Details ... | 32 | -0.75 | -1.91 | 0.000 | 0.000 | 0.001 | 942 | tags=69%, list=16%, signal=82% |
| 17 | TARTE\_MATURE\_PC | Details ... | 117 | -0.67 | -1.91 | 0.000 | 0.000 | 0.001 | 1166 | tags=57%, list=20%, signal=70% |
| 18 | IRITANI\_ADPROX\_VASC | Details ... | 64 | -0.69 | -1.90 | 0.000 | 0.000 | 0.001 | 1066 | tags=59%, list=19%, signal=72% |
| 19 | TGFBETA\_ALL\_UP | Details ... | 31 | -0.75 | -1.90 | 0.000 | 0.000 | 0.001 | 962 | tags=68%, list=17%, signal=81% |
| 20 | VERHAAK\_AML\_NPM1\_MUT\_VS\_WT\_UP | Details ... | 55 | -0.70 | -1.90 | 0.000 | 0.000 | 0.002 | 793 | tags=51%, list=14%, signal=59% |
| 21 | CELL\_ADHESION |  | 53 | -0.70 | -1.90 | 0.000 | 0.000 | 0.003 | 1140 | tags=62%, list=20%, signal=77% |
| 22 | ROS\_MOUSE\_AORTA\_DN |  | 41 | -0.73 | -1.89 | 0.000 | 0.000 | 0.004 | 620 | tags=44%, list=11%, signal=49% |
| 23 | CMV\_ALL\_DN |  | 48 | -0.70 | -1.88 | 0.000 | 0.000 | 0.006 | 957 | tags=58%, list=17%, signal=69% |
| 24 | IGLESIAS\_E2FMINUS\_UP |  | 78 | -0.68 | -1.88 | 0.000 | 0.000 | 0.006 | 1118 | tags=59%, list=20%, signal=72% |
| 25 | BASSO\_HCL\_DIFF |  | 36 | -0.73 | -1.87 | 0.000 | 0.001 | 0.015 | 896 | tags=58%, list=16%, signal=69% |
| 26 | ALCALAY\_AML\_NPMC\_UP |  | 56 | -0.69 | -1.85 | 0.000 | 0.001 | 0.017 | 793 | tags=48%, list=14%, signal=55% |
| 27 | SHEPARD\_POS\_REG\_OF\_CELL\_PROLIFERATION |  | 31 | -0.73 | -1.84 | 0.000 | 0.001 | 0.037 | 749 | tags=55%, list=13%, signal=63% |
| 28 | RAS\_ONCOGENIC\_SIGNATURE |  | 93 | -0.65 | -1.83 | 0.000 | 0.002 | 0.043 | 1234 | tags=51%, list=22%, signal=63% |
| 29 | VERHAAK\_AML\_NPM1\_MUT\_VS\_WT\_DN |  | 77 | -0.65 | -1.83 | 0.000 | 0.001 | 0.043 | 899 | tags=51%, list=16%, signal=59% |
| 30 | CARIES\_PULP\_DN |  | 20 | -0.78 | -1.83 | 0.000 | 0.002 | 0.049 | 787 | tags=60%, list=14%, signal=69% |
| 31 | TAVOR\_CEBP\_UP |  | 24 | -0.75 | -1.83 | 0.000 | 0.002 | 0.052 | 627 | tags=58%, list=11%, signal=65% |
| 32 | ADIP\_DIFF\_CLUSTER2 |  | 17 | -0.79 | -1.82 | 0.000 | 0.002 | 0.052 | 560 | tags=59%, list=10%, signal=65% |
| 33 | IL1\_CORNEA\_UP |  | 22 | -0.76 | -1.82 | 0.000 | 0.002 | 0.058 | 682 | tags=55%, list=12%, signal=62% |
| 34 | IRITANI\_ADPROX\_DN |  | 30 | -0.73 | -1.82 | 0.000 | 0.002 | 0.062 | 1066 | tags=57%, list=19%, signal=69% |
| 35 | BECKER\_TAMOXIFEN\_RESISTANT\_DN |  | 21 | -0.76 | -1.81 | 0.000 | 0.002 | 0.068 | 473 | tags=52%, list=8%, signal=57% |
| 36 | IRS1\_KO\_ADIP\_DN |  | 40 | -0.69 | -1.81 | 0.000 | 0.002 | 0.068 | 724 | tags=43%, list=13%, signal=48% |
| 37 | PARP\_KO\_UP |  | 17 | -0.78 | -1.81 | 0.001 | 0.002 | 0.070 | 589 | tags=47%, list=10%, signal=52% |
| 38 | GN\_CAMP\_GRANULOSA\_DN |  | 26 | -0.73 | -1.81 | 0.000 | 0.002 | 0.073 | 819 | tags=50%, list=14%, signal=58% |
| 39 | NELSON\_ANDROGEN\_UP |  | 27 | -0.72 | -1.80 | 0.000 | 0.002 | 0.080 | 746 | tags=48%, list=13%, signal=55% |
| 40 | EMT\_UP |  | 30 | -0.72 | -1.80 | 0.000 | 0.002 | 0.080 | 720 | tags=63%, list=13%, signal=72% |
| 41 | ADIP\_VS\_FIBRO\_UP |  | 16 | -0.79 | -1.80 | 0.000 | 0.002 | 0.085 | 559 | tags=44%, list=10%, signal=48% |
| 42 | LI\_FETAL\_VS\_WT\_KIDNEY\_UP |  | 83 | -0.64 | -1.79 | 0.000 | 0.002 | 0.095 | 1108 | tags=52%, list=19%, signal=63% |
| 43 | TSA\_HEPATOMA\_CANCER\_UP |  | 16 | -0.79 | -1.79 | 0.000 | 0.002 | 0.100 | 825 | tags=75%, list=14%, signal=87% |
| 44 | HSA01430\_CELL\_COMMUNICATION |  | 20 | -0.77 | -1.79 | 0.000 | 0.002 | 0.100 | 811 | tags=70%, list=14%, signal=81% |
| 45 | GERY\_CEBP\_TARGETS |  | 46 | -0.68 | -1.79 | 0.000 | 0.002 | 0.102 | 1062 | tags=48%, list=19%, signal=58% |
| 46 | JECHLINGER\_EMT\_UP |  | 28 | -0.72 | -1.79 | 0.000 | 0.002 | 0.103 | 664 | tags=61%, list=12%, signal=68% |
| 47 | TGFBETA\_EARLY\_UP |  | 17 | -0.77 | -1.79 | 0.000 | 0.002 | 0.104 | 852 | tags=71%, list=15%, signal=83% |
| 48 | HSA04530\_TIGHT\_JUNCTION |  | 45 | -0.67 | -1.79 | 0.000 | 0.002 | 0.104 | 1063 | tags=51%, list=19%, signal=62% |
| 49 | STOSSI\_ER\_UP |  | 15 | -0.80 | -1.78 | 0.000 | 0.003 | 0.122 | 885 | tags=73%, list=15%, signal=87% |
| 50 | HSA04360\_AXON\_GUIDANCE |  | 36 | -0.69 | -1.78 | 0.000 | 0.003 | 0.127 | 964 | tags=50%, list=17%, signal=60% |
| 51 | FLECHNER\_KIDNEY\_TRANSPLANT\_REJECTION\_UP |  | 38 | -0.69 | -1.78 | 0.000 | 0.003 | 0.133 | 899 | tags=47%, list=16%, signal=56% |
| 52 | BRCA\_BRCA1\_NEG |  | 46 | -0.67 | -1.78 | 0.000 | 0.003 | 0.138 | 1032 | tags=57%, list=18%, signal=68% |
| 53 | INFLAMMATORY\_RESPONSE\_PATHWAY |  | 17 | -0.77 | -1.77 | 0.000 | 0.003 | 0.141 | 633 | tags=65%, list=11%, signal=73% |
| 54 | ADIP\_VS\_PREADIP\_DN |  | 17 | -0.77 | -1.77 | 0.000 | 0.003 | 0.146 | 460 | tags=47%, list=8%, signal=51% |
| 55 | CALRES\_RHESUS\_UP |  | 25 | -0.72 | -1.77 | 0.000 | 0.003 | 0.146 | 940 | tags=64%, list=16%, signal=76% |
| 56 | NING\_COPD\_UP |  | 56 | -0.66 | -1.77 | 0.000 | 0.003 | 0.147 | 1222 | tags=50%, list=21%, signal=63% |
| 57 | HSA04660\_T\_CELL\_RECEPTOR\_SIGNALING\_PATHWAY |  | 52 | -0.65 | -1.77 | 0.000 | 0.003 | 0.153 | 1190 | tags=50%, list=21%, signal=63% |
| 58 | CELL\_ADHESION\_MOLECULE\_ACTIVITY |  | 31 | -0.70 | -1.77 | 0.000 | 0.003 | 0.155 | 1329 | tags=65%, list=23%, signal=84% |
| 59 | NAKAJIMA\_MCS\_UP |  | 33 | -0.70 | -1.77 | 0.000 | 0.003 | 0.159 | 1106 | tags=67%, list=19%, signal=82% |
| 60 | HSA04810\_REGULATION\_OF\_ACTIN\_CYTOSKELETON |  | 85 | -0.62 | -1.77 | 0.000 | 0.003 | 0.163 | 1222 | tags=45%, list=21%, signal=56% |
| 61 | ATRIA\_UP |  | 52 | -0.65 | -1.76 | 0.000 | 0.003 | 0.165 | 1367 | tags=63%, list=24%, signal=83% |
| 62 | AGEING\_KIDNEY\_SPECIFIC\_UP |  | 71 | -0.64 | -1.76 | 0.000 | 0.003 | 0.194 | 845 | tags=45%, list=15%, signal=52% |
| 63 | PASSERINI\_EM |  | 15 | -0.78 | -1.75 | 0.001 | 0.004 | 0.214 | 648 | tags=53%, list=11%, signal=60% |
| 64 | LINDSTEDT\_DEND\_DN |  | 28 | -0.71 | -1.75 | 0.000 | 0.004 | 0.215 | 1245 | tags=71%, list=22%, signal=91% |
| 65 | CORDERO\_KRAS\_KD\_VS\_CONTROL\_UP |  | 33 | -0.69 | -1.75 | 0.001 | 0.004 | 0.218 | 1226 | tags=70%, list=21%, signal=88% |
| 66 | PASSERINI\_ADHESION |  | 20 | -0.74 | -1.75 | 0.000 | 0.004 | 0.218 | 1269 | tags=80%, list=22%, signal=102% |
| 67 | HTERT\_DN |  | 22 | -0.74 | -1.75 | 0.000 | 0.004 | 0.223 | 939 | tags=64%, list=16%, signal=76% |
| 68 | EMT\_DN |  | 24 | -0.72 | -1.75 | 0.000 | 0.004 | 0.240 | 882 | tags=63%, list=15%, signal=74% |
| 69 | MYOD\_NIH3T3\_DN |  | 25 | -0.71 | -1.74 | 0.001 | 0.004 | 0.252 | 995 | tags=52%, list=17%, signal=63% |
| 70 | NI2\_MOUSE\_DN |  | 19 | -0.74 | -1.74 | 0.000 | 0.004 | 0.252 | 875 | tags=53%, list=15%, signal=62% |
| 71 | POD1\_KO\_DN |  | 309 | -0.59 | -1.74 | 0.000 | 0.004 | 0.263 | 1070 | tags=37%, list=19%, signal=43% |
| 72 | LEE\_E2F1\_UP |  | 26 | -0.70 | -1.74 | 0.000 | 0.004 | 0.275 | 826 | tags=54%, list=14%, signal=63% |
| 73 | DAC\_PANC\_UP |  | 90 | -0.62 | -1.73 | 0.000 | 0.004 | 0.282 | 1051 | tags=46%, list=18%, signal=55% |
| 74 | CELL\_SURFACE\_RECEPTOR\_LINKED\_SIGNAL\_TRANSDUCTION |  | 41 | -0.66 | -1.73 | 0.000 | 0.004 | 0.292 | 942 | tags=49%, list=16%, signal=58% |
| 75 | IDX\_TSA\_UP\_CLUSTER2 |  | 31 | -0.69 | -1.73 | 0.000 | 0.005 | 0.297 | 488 | tags=35%, list=9%, signal=39% |
| 76 | ROSS\_PML\_RAR |  | 26 | -0.69 | -1.72 | 0.001 | 0.005 | 0.337 | 762 | tags=46%, list=13%, signal=53% |
| 77 | AGED\_MOUSE\_CEREBELLUM\_DN |  | 15 | -0.78 | -1.72 | 0.000 | 0.005 | 0.337 | 460 | tags=40%, list=8%, signal=43% |
| 78 | ADIP\_VS\_PREADIP\_UP |  | 15 | -0.78 | -1.72 | 0.001 | 0.005 | 0.342 | 559 | tags=47%, list=10%, signal=52% |
| 79 | BRG1\_ALAB\_DN |  | 19 | -0.73 | -1.72 | 0.000 | 0.005 | 0.353 | 393 | tags=32%, list=7%, signal=34% |
| 80 | HSA05218\_MELANOMA |  | 28 | -0.69 | -1.72 | 0.000 | 0.005 | 0.369 | 1185 | tags=57%, list=21%, signal=72% |
| 81 | CROONQUIST\_IL6\_STROMA\_UP |  | 15 | -0.77 | -1.72 | 0.000 | 0.005 | 0.369 | 1321 | tags=100%, list=23%, signal=130% |
| 82 | AGEING\_KIDNEY\_UP |  | 138 | -0.60 | -1.71 | 0.000 | 0.006 | 0.396 | 1132 | tags=41%, list=20%, signal=50% |
| 83 | BRENTANI\_CELL\_ADHESION |  | 37 | -0.67 | -1.71 | 0.000 | 0.006 | 0.404 | 825 | tags=51%, list=14%, signal=60% |
| 84 | RUTELLA\_HEPATGFSNDCS\_UP |  | 61 | -0.62 | -1.71 | 0.000 | 0.006 | 0.409 | 941 | tags=39%, list=16%, signal=47% |
| 85 | HINATA\_NFKB\_UP |  | 39 | -0.66 | -1.71 | 0.000 | 0.006 | 0.420 | 1196 | tags=54%, list=21%, signal=68% |
| 86 | EGF\_HDMEC\_UP |  | 25 | -0.70 | -1.71 | 0.001 | 0.006 | 0.420 | 1066 | tags=52%, list=19%, signal=64% |
| 87 | PASSERINI\_SIGNAL |  | 130 | -0.59 | -1.71 | 0.000 | 0.006 | 0.420 | 1158 | tags=46%, list=20%, signal=57% |
| 88 | AGEING\_BRAIN\_UP |  | 86 | -0.61 | -1.71 | 0.000 | 0.006 | 0.429 | 952 | tags=43%, list=17%, signal=51% |
| 89 | HOUSTIS\_ROS |  | 15 | -0.76 | -1.71 | 0.001 | 0.006 | 0.441 | 782 | tags=53%, list=14%, signal=62% |
| 90 | LVAD\_HEARTFAILURE\_UP |  | 28 | -0.68 | -1.70 | 0.001 | 0.006 | 0.447 | 692 | tags=39%, list=12%, signal=44% |
| 91 | HOHENKIRK\_MONOCYTE\_DEND\_UP |  | 49 | -0.65 | -1.70 | 0.000 | 0.006 | 0.448 | 1337 | tags=61%, list=23%, signal=79% |
| 92 | MANALO\_HYPOXIA\_UP |  | 38 | -0.66 | -1.70 | 0.000 | 0.006 | 0.466 | 1235 | tags=66%, list=22%, signal=83% |
| 93 | TAKEDA\_NUP8\_HOXA9\_6H\_UP |  | 28 | -0.69 | -1.70 | 0.000 | 0.007 | 0.471 | 878 | tags=57%, list=15%, signal=67% |
| 94 | LE\_MYELIN\_DN |  | 32 | -0.66 | -1.70 | 0.002 | 0.007 | 0.482 | 1419 | tags=56%, list=25%, signal=74% |
| 95 | CELL\_ADHESION\_RECEPTOR\_ACTIVITY |  | 16 | -0.75 | -1.70 | 0.000 | 0.007 | 0.505 | 730 | tags=56%, list=13%, signal=64% |
| 96 | ADIP\_DIFF\_UP |  | 30 | -0.67 | -1.69 | 0.000 | 0.007 | 0.506 | 919 | tags=37%, list=16%, signal=43% |
| 97 | LEE\_DENA\_UP |  | 22 | -0.70 | -1.69 | 0.000 | 0.008 | 0.535 | 725 | tags=50%, list=13%, signal=57% |
| 98 | TGF\_BETA\_SIGNALING\_PATHWAY |  | 19 | -0.73 | -1.69 | 0.001 | 0.008 | 0.543 | 1547 | tags=89%, list=27%, signal=122% |
| 99 | YAGI\_AML\_PROG\_FAB |  | 74 | -0.61 | -1.69 | 0.000 | 0.008 | 0.555 | 600 | tags=30%, list=10%, signal=33% |
| 100 | KUMAR\_HOXA\_DIFF |  | 149 | -0.58 | -1.69 | 0.000 | 0.008 | 0.570 | 820 | tags=36%, list=14%, signal=40% |
| 101 | HSC\_LTHSC\_FETAL |  | 88 | -0.60 | -1.68 | 0.000 | 0.008 | 0.593 | 914 | tags=36%, list=16%, signal=43% |
| 102 | CARIES\_PULP\_UP |  | 86 | -0.60 | -1.68 | 0.000 | 0.009 | 0.621 | 1160 | tags=48%, list=20%, signal=59% |
| 103 | IDX\_TSA\_DN\_CLUSTER1 |  | 16 | -0.74 | -1.68 | 0.002 | 0.009 | 0.623 | 598 | tags=56%, list=10%, signal=63% |
| 104 | HADDAD\_HSC\_CD7\_UP |  | 27 | -0.67 | -1.68 | 0.002 | 0.009 | 0.631 | 1043 | tags=70%, list=18%, signal=86% |
| 105 | VEGF\_MMMEC\_ALL\_UP |  | 32 | -0.66 | -1.68 | 0.000 | 0.009 | 0.631 | 666 | tags=44%, list=12%, signal=49% |
| 106 | SMOOTH\_MUSCLE\_CONTRACTION |  | 52 | -0.62 | -1.68 | 0.000 | 0.009 | 0.635 | 1201 | tags=48%, list=21%, signal=60% |
| 107 | WIELAND\_HEPATITIS\_B\_INDUCED |  | 44 | -0.63 | -1.67 | 0.001 | 0.009 | 0.646 | 1214 | tags=41%, list=21%, signal=52% |
| 108 | PARK\_RARALPHA\_UP |  | 21 | -0.70 | -1.67 | 0.002 | 0.010 | 0.661 | 1394 | tags=76%, list=24%, signal=100% |
| 109 | HTERT\_UP |  | 30 | -0.67 | -1.67 | 0.003 | 0.010 | 0.662 | 792 | tags=40%, list=14%, signal=46% |
| 110 | TAKEDA\_NUP8\_HOXA9\_16D\_DN |  | 86 | -0.60 | -1.67 | 0.000 | 0.010 | 0.667 | 612 | tags=24%, list=11%, signal=27% |
| 111 | KANG\_TERT\_UP |  | 25 | -0.67 | -1.67 | 0.002 | 0.010 | 0.670 | 1256 | tags=56%, list=22%, signal=71% |
| 112 | CMV\_HCMV\_TIMECOURSE\_24HRS\_DN |  | 15 | -0.74 | -1.66 | 0.001 | 0.011 | 0.702 | 1091 | tags=67%, list=19%, signal=82% |
| 113 | HSA04540\_GAP\_JUNCTION |  | 34 | -0.65 | -1.66 | 0.001 | 0.011 | 0.706 | 1186 | tags=50%, list=21%, signal=63% |
| 114 | HSC\_LTHSC\_SHARED |  | 88 | -0.60 | -1.66 | 0.000 | 0.011 | 0.709 | 914 | tags=36%, list=16%, signal=43% |
| 115 | HSA04670\_LEUKOCYTE\_TRANSENDOTHELIAL\_MIGRATION |  | 52 | -0.61 | -1.66 | 0.001 | 0.011 | 0.711 | 1329 | tags=50%, list=23%, signal=65% |
| 116 | NAKAJIMA\_MCSMBP\_MAST |  | 19 | -0.71 | -1.66 | 0.004 | 0.011 | 0.719 | 939 | tags=63%, list=16%, signal=75% |
| 117 | NADLER\_OBESITY\_UP |  | 30 | -0.65 | -1.66 | 0.003 | 0.011 | 0.727 | 1118 | tags=47%, list=20%, signal=58% |
| 118 | FERRANDO\_MLL\_T\_ALL\_UP |  | 37 | -0.65 | -1.66 | 0.000 | 0.011 | 0.730 | 1473 | tags=65%, list=26%, signal=87% |
| 119 | ROSS\_CBF\_MYH |  | 30 | -0.67 | -1.66 | 0.000 | 0.011 | 0.735 | 893 | tags=53%, list=16%, signal=63% |
| 120 | HSA04350\_TGF\_BETA\_SIGNALING\_PATHWAY |  | 31 | -0.66 | -1.66 | 0.001 | 0.011 | 0.741 | 451 | tags=29%, list=8%, signal=31% |
| 121 | HADDAD\_CD45CD7\_PLUS\_VS\_MINUS\_UP |  | 27 | -0.67 | -1.66 | 0.005 | 0.011 | 0.744 | 1043 | tags=70%, list=18%, signal=86% |
| 122 | CHIARETTI\_T\_ALL |  | 94 | -0.59 | -1.66 | 0.000 | 0.011 | 0.745 | 1091 | tags=49%, list=19%, signal=59% |
| 123 | KANG\_TERT\_DN |  | 26 | -0.67 | -1.65 | 0.000 | 0.012 | 0.764 | 705 | tags=50%, list=12%, signal=57% |
| 124 | JECHLINGER\_EMT\_DN |  | 20 | -0.71 | -1.65 | 0.001 | 0.012 | 0.781 | 882 | tags=65%, list=15%, signal=77% |
| 125 | RIBAVIRIN\_RSV\_DN |  | 17 | -0.73 | -1.65 | 0.007 | 0.012 | 0.789 | 676 | tags=47%, list=12%, signal=53% |
| 126 | LEI\_MYB\_REGULATED\_GENES |  | 119 | -0.58 | -1.65 | 0.000 | 0.013 | 0.804 | 1090 | tags=41%, list=19%, signal=50% |
| 127 | CHIARETTI\_T\_ALL\_DIFF |  | 104 | -0.59 | -1.65 | 0.000 | 0.013 | 0.808 | 1091 | tags=50%, list=19%, signal=61% |
| 128 | BREAST\_CANCER\_ESTROGEN\_SIGNALING |  | 30 | -0.66 | -1.64 | 0.002 | 0.013 | 0.815 | 942 | tags=53%, list=16%, signal=64% |
| 129 | HOFFMANN\_BIVSBII\_LGBII |  | 43 | -0.62 | -1.64 | 0.001 | 0.013 | 0.826 | 673 | tags=33%, list=12%, signal=37% |
| 130 | BRG1\_SW13\_UP |  | 16 | -0.72 | -1.64 | 0.006 | 0.015 | 0.855 | 837 | tags=56%, list=15%, signal=66% |
| 131 | IDX\_TSA\_DN\_CLUSTER2 |  | 35 | -0.64 | -1.64 | 0.002 | 0.015 | 0.857 | 1805 | tags=80%, list=32%, signal=116% |
| 132 | ET743\_RESIST\_DN |  | 17 | -0.71 | -1.63 | 0.005 | 0.015 | 0.873 | 1050 | tags=59%, list=18%, signal=72% |
| 133 | SANSOM\_APC\_5\_DN |  | 142 | -0.57 | -1.63 | 0.000 | 0.015 | 0.875 | 1196 | tags=44%, list=21%, signal=55% |
| 134 | YAO\_P4\_KO\_VS\_WT\_UP |  | 21 | -0.68 | -1.63 | 0.005 | 0.016 | 0.884 | 712 | tags=48%, list=12%, signal=54% |
| 135 | HSC\_LTHSC\_ADULT |  | 108 | -0.57 | -1.63 | 0.000 | 0.016 | 0.884 | 914 | tags=33%, list=16%, signal=39% |
| 136 | GH\_EXOGENOUS\_ANY\_UP |  | 52 | -0.60 | -1.62 | 0.002 | 0.017 | 0.911 | 1007 | tags=35%, list=18%, signal=42% |
| 137 | LH\_GRANULOSA\_DN |  | 38 | -0.63 | -1.62 | 0.007 | 0.017 | 0.911 | 1160 | tags=42%, list=20%, signal=52% |
| 138 | ESR\_FIBROBLAST\_UP |  | 19 | -0.69 | -1.62 | 0.001 | 0.018 | 0.912 | 1396 | tags=63%, list=24%, signal=83% |
| 139 | TPA\_SENS\_LATE\_UP |  | 22 | -0.68 | -1.62 | 0.004 | 0.018 | 0.912 | 893 | tags=41%, list=16%, signal=48% |
| 140 | DIAB\_NEPH\_DN |  | 169 | -0.56 | -1.62 | 0.000 | 0.018 | 0.918 | 1091 | tags=36%, list=19%, signal=43% |
| 141 | CALCIUM\_REGULATION\_IN\_CARDIAC\_CELLS |  | 42 | -0.61 | -1.62 | 0.004 | 0.018 | 0.922 | 1150 | tags=45%, list=20%, signal=56% |
| 142 | FSH\_GRANULOSA\_DN |  | 38 | -0.63 | -1.62 | 0.002 | 0.019 | 0.935 | 1160 | tags=42%, list=20%, signal=52% |
| 143 | VEGF\_MMMEC\_3HRS\_UP |  | 22 | -0.67 | -1.62 | 0.009 | 0.019 | 0.935 | 620 | tags=32%, list=11%, signal=36% |
| 144 | TCRPATHWAY |  | 27 | -0.65 | -1.61 | 0.005 | 0.019 | 0.937 | 1321 | tags=56%, list=23%, signal=72% |
| 145 | LEE\_MYC\_TGFA\_UP |  | 19 | -0.69 | -1.61 | 0.007 | 0.020 | 0.944 | 826 | tags=53%, list=14%, signal=61% |
| 146 | FALT\_BCLL\_IG\_MUTATED\_VS\_WT\_UP |  | 20 | -0.68 | -1.61 | 0.004 | 0.020 | 0.947 | 957 | tags=50%, list=17%, signal=60% |
| 147 | TPA\_SENS\_MIDDLE\_UP |  | 28 | -0.64 | -1.61 | 0.006 | 0.020 | 0.949 | 1321 | tags=46%, list=23%, signal=60% |
| 148 | HSA05221\_ACUTE\_MYELOID\_LEUKEMIA |  | 33 | -0.64 | -1.61 | 0.002 | 0.020 | 0.950 | 1258 | tags=55%, list=22%, signal=70% |
| 149 | BRENTANI\_SIGNALING |  | 76 | -0.57 | -1.61 | 0.002 | 0.020 | 0.953 | 730 | tags=30%, list=13%, signal=34% |
| 150 | LEE\_ACOX1\_UP |  | 19 | -0.68 | -1.61 | 0.010 | 0.020 | 0.953 | 694 | tags=47%, list=12%, signal=54% |
| 151 | HYPOPHYSECTOMY\_RAT\_DN |  | 22 | -0.67 | -1.61 | 0.005 | 0.021 | 0.954 | 764 | tags=36%, list=13%, signal=42% |
| 152 | HOFMANN\_MDS\_CD34\_LOW\_AND\_HIGH\_RISK |  | 17 | -0.69 | -1.61 | 0.007 | 0.021 | 0.957 | 911 | tags=47%, list=16%, signal=56% |
| 153 | CIS\_XPC\_DN |  | 77 | -0.58 | -1.61 | 0.000 | 0.021 | 0.959 | 1280 | tags=35%, list=22%, signal=45% |
| 154 | CHANG\_SERUM\_RESPONSE\_DN |  | 54 | -0.59 | -1.60 | 0.001 | 0.021 | 0.960 | 1424 | tags=52%, list=25%, signal=68% |
| 155 | RUTELLA\_HEMATOGFSNDCS\_DIFF |  | 284 | -0.54 | -1.60 | 0.000 | 0.021 | 0.962 | 1191 | tags=37%, list=21%, signal=44% |
| 156 | GAMMA\_UNIQUE\_FIBRO\_DN |  | 19 | -0.67 | -1.60 | 0.025 | 0.021 | 0.965 | 826 | tags=32%, list=14%, signal=37% |
| 157 | KRETZSCHMAR\_IL6\_DIFF |  | 64 | -0.58 | -1.60 | 0.002 | 0.022 | 0.970 | 912 | tags=34%, list=16%, signal=40% |
| 158 | RADMACHER\_AMLNORMALKARYTYPE\_SIG |  | 24 | -0.65 | -1.60 | 0.013 | 0.022 | 0.970 | 1118 | tags=50%, list=20%, signal=62% |
| 159 | BROCKE\_IL6 |  | 64 | -0.58 | -1.60 | 0.003 | 0.022 | 0.970 | 912 | tags=34%, list=16%, signal=40% |
| 160 | HOFFMANN\_BIVSBII\_BI |  | 44 | -0.60 | -1.60 | 0.003 | 0.022 | 0.970 | 1245 | tags=50%, list=22%, signal=63% |
| 161 | TAKEDA\_NUP8\_HOXA9\_6H\_DN |  | 15 | -0.72 | -1.60 | 0.013 | 0.022 | 0.972 | 1191 | tags=67%, list=21%, signal=84% |
| 162 | MAMMARY\_DEV\_UP |  | 21 | -0.68 | -1.59 | 0.006 | 0.023 | 0.974 | 497 | tags=33%, list=9%, signal=36% |
| 163 | ROSS\_CBF |  | 21 | -0.67 | -1.59 | 0.005 | 0.023 | 0.978 | 1220 | tags=57%, list=21%, signal=72% |
| 164 | BRCA1\_OVEREXP\_PROSTATE\_UP |  | 64 | -0.58 | -1.59 | 0.002 | 0.024 | 0.978 | 1176 | tags=47%, list=21%, signal=58% |
| 165 | MOREAUX\_TACI\_HI\_VS\_LOW\_UP |  | 81 | -0.57 | -1.59 | 0.001 | 0.024 | 0.979 | 1085 | tags=41%, list=19%, signal=50% |
| 166 | CALCINEURIN\_NF\_AT\_SIGNALING |  | 36 | -0.62 | -1.59 | 0.003 | 0.024 | 0.979 | 1209 | tags=42%, list=21%, signal=52% |
| 167 | HOFFMANN\_BIVSBII\_IMVM |  | 37 | -0.61 | -1.59 | 0.007 | 0.025 | 0.984 | 912 | tags=35%, list=16%, signal=42% |
| 168 | HSA00562\_INOSITOL\_PHOSPHATE\_METABOLISM |  | 22 | -0.65 | -1.58 | 0.012 | 0.026 | 0.989 | 849 | tags=41%, list=15%, signal=48% |
| 169 | GAY\_YY1\_UP |  | 49 | -0.59 | -1.58 | 0.002 | 0.027 | 0.989 | 1062 | tags=49%, list=19%, signal=60% |
| 170 | HCC\_SURVIVAL\_GOOD\_VS\_POOR\_UP |  | 31 | -0.63 | -1.58 | 0.006 | 0.027 | 0.989 | 1060 | tags=39%, list=19%, signal=47% |
| 171 | ZHAN\_MMPC\_SIMAL |  | 25 | -0.65 | -1.58 | 0.014 | 0.027 | 0.992 | 1241 | tags=48%, list=22%, signal=61% |
| 172 | UEDA\_MOUSE\_SCN |  | 35 | -0.61 | -1.58 | 0.004 | 0.028 | 0.993 | 1001 | tags=43%, list=17%, signal=52% |
| 173 | TSA\_HEPATOMA\_UP |  | 15 | -0.71 | -1.58 | 0.011 | 0.028 | 0.993 | 797 | tags=33%, list=14%, signal=39% |
| 174 | TPA\_RESIST\_LATE\_DN |  | 24 | -0.64 | -1.57 | 0.009 | 0.029 | 0.994 | 648 | tags=38%, list=11%, signal=42% |
| 175 | CMV-UV\_HCMV\_6HRS\_DN |  | 41 | -0.59 | -1.57 | 0.007 | 0.029 | 0.994 | 630 | tags=34%, list=11%, signal=38% |
| 176 | ICHIBA\_GVHD |  | 95 | -0.56 | -1.57 | 0.000 | 0.029 | 0.994 | 1113 | tags=43%, list=19%, signal=53% |
| 177 | UVC\_TTD\_8HR\_DN |  | 75 | -0.56 | -1.57 | 0.003 | 0.030 | 0.994 | 1206 | tags=40%, list=21%, signal=50% |
| 178 | HALMOS\_CEBP\_UP |  | 21 | -0.65 | -1.57 | 0.010 | 0.030 | 0.994 | 1131 | tags=57%, list=20%, signal=71% |
| 179 | HDACI\_COLON\_TSA\_UP |  | 40 | -0.59 | -1.57 | 0.003 | 0.030 | 0.994 | 648 | tags=35%, list=11%, signal=39% |
| 180 | HSA05219\_BLADDER\_CANCER |  | 18 | -0.69 | -1.57 | 0.011 | 0.030 | 0.994 | 1052 | tags=50%, list=18%, signal=61% |
| 181 | JISON\_SICKLE\_CELL |  | 16 | -0.70 | -1.57 | 0.004 | 0.030 | 0.994 | 739 | tags=44%, list=13%, signal=50% |
| 182 | BASSO\_GERMINAL\_CENTER\_CD40\_DN |  | 27 | -0.63 | -1.57 | 0.009 | 0.031 | 0.995 | 841 | tags=41%, list=15%, signal=48% |
| 183 | ST\_INTEGRIN\_SIGNALING\_PATHWAY |  | 42 | -0.59 | -1.56 | 0.007 | 0.032 | 0.996 | 1269 | tags=43%, list=22%, signal=55% |
| 184 | TNFALPHA\_30MIN\_UP |  | 18 | -0.67 | -1.56 | 0.020 | 0.032 | 0.997 | 1332 | tags=61%, list=23%, signal=79% |
| 185 | BRENTANI\_IMMUNE\_FUNCTION |  | 26 | -0.63 | -1.56 | 0.009 | 0.033 | 0.997 | 899 | tags=46%, list=16%, signal=55% |
| 186 | ET743\_HELA\_UP |  | 24 | -0.64 | -1.56 | 0.012 | 0.033 | 0.997 | 1257 | tags=67%, list=22%, signal=85% |
| 187 | GUO\_HEX\_DN |  | 26 | -0.63 | -1.56 | 0.008 | 0.033 | 0.997 | 1100 | tags=38%, list=19%, signal=47% |
| 188 | TAKEDA\_NUP8\_HOXA9\_10D\_UP |  | 56 | -0.58 | -1.55 | 0.004 | 0.035 | 1.000 | 780 | tags=45%, list=14%, signal=51% |
| 189 | HSA04080\_NEUROACTIVE\_LIGAND\_RECEPTOR\_INTERACTION |  | 26 | -0.63 | -1.55 | 0.008 | 0.037 | 1.000 | 841 | tags=54%, list=15%, signal=63% |
| 190 | ROSS\_CBF\_LEUKEMIA |  | 18 | -0.66 | -1.55 | 0.017 | 0.037 | 1.000 | 1695 | tags=72%, list=30%, signal=102% |
| 191 | HSA04520\_ADHERENS\_JUNCTION |  | 25 | -0.63 | -1.55 | 0.017 | 0.037 | 1.000 | 584 | tags=36%, list=10%, signal=40% |
| 192 | TNFALPHA\_ALL\_UP |  | 33 | -0.61 | -1.55 | 0.012 | 0.038 | 1.000 | 1332 | tags=45%, list=23%, signal=59% |
| 193 | PARK\_RARALPHA\_MOD |  | 26 | -0.62 | -1.54 | 0.016 | 0.038 | 1.000 | 887 | tags=38%, list=15%, signal=45% |
| 194 | INOS\_ALL\_DN |  | 37 | -0.59 | -1.54 | 0.007 | 0.038 | 1.000 | 1473 | tags=43%, list=26%, signal=58% |
| 195 | AGED\_RHESUS\_UP |  | 68 | -0.57 | -1.54 | 0.006 | 0.038 | 1.000 | 1217 | tags=40%, list=21%, signal=50% |
| 196 | RHOPATHWAY |  | 15 | -0.69 | -1.54 | 0.022 | 0.039 | 1.000 | 882 | tags=47%, list=15%, signal=55% |
| 197 | HOHENKIRK\_MONOCYTE\_DEND\_DN |  | 57 | -0.57 | -1.54 | 0.003 | 0.039 | 1.000 | 893 | tags=40%, list=16%, signal=47% |
| 198 | BASSO\_GERMINAL\_CENTER\_CD40\_UP |  | 32 | -0.61 | -1.54 | 0.009 | 0.040 | 1.000 | 1559 | tags=56%, list=27%, signal=77% |
| 199 | TPA\_SENS\_LATE\_DN |  | 95 | -0.54 | -1.54 | 0.001 | 0.040 | 1.000 | 1158 | tags=37%, list=20%, signal=45% |
| 200 | BRUNO\_IL3\_DN |  | 26 | -0.63 | -1.54 | 0.010 | 0.040 | 1.000 | 1032 | tags=50%, list=18%, signal=61% |
| 201 | CROMER\_HYPOPHARYNGEAL\_MET\_VS\_NON\_DN |  | 30 | -0.61 | -1.54 | 0.019 | 0.041 | 1.000 | 887 | tags=43%, list=15%, signal=51% |
| 202 | HSA04020\_CALCIUM\_SIGNALING\_PATHWAY |  | 42 | -0.59 | -1.53 | 0.005 | 0.042 | 1.000 | 804 | tags=36%, list=14%, signal=41% |
| 203 | GALINDO\_ACT\_UP |  | 27 | -0.62 | -1.53 | 0.011 | 0.042 | 1.000 | 1090 | tags=56%, list=19%, signal=68% |
| 204 | HSA04630\_JAK\_STAT\_SIGNALING\_PATHWAY |  | 52 | -0.57 | -1.53 | 0.002 | 0.042 | 1.000 | 1220 | tags=42%, list=21%, signal=53% |
| 205 | HSIAO\_LIVER\_SPECIFIC\_GENES |  | 58 | -0.56 | -1.53 | 0.005 | 0.043 | 1.000 | 1074 | tags=43%, list=19%, signal=53% |
| 206 | GH\_EXOGENOUS\_MIDDLE\_UP |  | 15 | -0.68 | -1.53 | 0.020 | 0.043 | 1.000 | 914 | tags=40%, list=16%, signal=47% |
| 207 | BRENTANI\_PROTEIN\_MODIFICATION |  | 78 | -0.55 | -1.53 | 0.002 | 0.044 | 1.000 | 1115 | tags=36%, list=19%, signal=44% |
| 208 | ZHAN\_MMPC\_LATEVS |  | 23 | -0.62 | -1.53 | 0.021 | 0.044 | 1.000 | 1183 | tags=57%, list=21%, signal=71% |
| 209 | SHEPARD\_NEG\_REG\_OF\_CELL\_PROLIFERATION |  | 40 | -0.59 | -1.53 | 0.008 | 0.044 | 1.000 | 1171 | tags=45%, list=20%, signal=56% |
| 210 | INTEGRIN\_MEDIATED\_CELL\_ADHESION\_KEGG |  | 46 | -0.57 | -1.53 | 0.014 | 0.045 | 1.000 | 1269 | tags=48%, list=22%, signal=61% |
| 211 | PROLIFERATION\_GENES |  | 126 | -0.53 | -1.53 | 0.000 | 0.045 | 1.000 | 1290 | tags=42%, list=23%, signal=53% |
| 212 | OKUMURA\_MC\_LPS |  | 64 | -0.55 | -1.52 | 0.004 | 0.045 | 1.000 | 569 | tags=23%, list=10%, signal=26% |
| 213 | HADDAD\_HSC\_CD7\_DN |  | 35 | -0.59 | -1.52 | 0.012 | 0.046 | 1.000 | 660 | tags=34%, list=12%, signal=39% |
| 214 | NING\_COPD\_DN |  | 48 | -0.57 | -1.52 | 0.009 | 0.047 | 1.000 | 1396 | tags=46%, list=24%, signal=60% |
| 215 | IDX\_TSA\_DN\_CLUSTER3 |  | 31 | -0.61 | -1.52 | 0.011 | 0.048 | 1.000 | 969 | tags=35%, list=17%, signal=42% |
| 216 | HEARTFAILURE\_VENTRICLE\_DN |  | 31 | -0.61 | -1.52 | 0.011 | 0.048 | 1.000 | 424 | tags=29%, list=7%, signal=31% |
| 217 | IL1\_CORNEA\_DN |  | 32 | -0.60 | -1.52 | 0.011 | 0.048 | 1.000 | 487 | tags=22%, list=9%, signal=24% |
| 218 | UVC\_HIGH\_D6\_DN |  | 17 | -0.65 | -1.52 | 0.033 | 0.048 | 1.000 | 606 | tags=35%, list=11%, signal=39% |
| 219 | HADDAD\_CD45CD7\_PLUS\_VS\_MINUS\_DN |  | 35 | -0.59 | -1.52 | 0.014 | 0.048 | 1.000 | 660 | tags=34%, list=12%, signal=39% |
| 220 | ELONGINA\_KO\_UP |  | 70 | -0.55 | -1.52 | 0.004 | 0.048 | 1.000 | 1316 | tags=49%, list=23%, signal=62% |
| 221 | HSA04916\_MELANOGENESIS |  | 28 | -0.61 | -1.52 | 0.022 | 0.048 | 1.000 | 1150 | tags=43%, list=20%, signal=53% |
| 222 | JISON\_SICKLECELL\_DIFF |  | 130 | -0.52 | -1.51 | 0.000 | 0.049 | 1.000 | 807 | tags=28%, list=14%, signal=32% |
| 223 | KNUDSEN\_PMNS\_UP |  | 35 | -0.59 | -1.51 | 0.015 | 0.049 | 1.000 | 904 | tags=37%, list=16%, signal=44% |
| 224 | ADDYA\_K562\_HEMIN\_TREATMENT |  | 27 | -0.61 | -1.51 | 0.018 | 0.049 | 1.000 | 1103 | tags=44%, list=19%, signal=55% |
| 225 | HEARTFAILURE\_ATRIA\_DN |  | 56 | -0.56 | -1.51 | 0.004 | 0.049 | 1.000 | 1052 | tags=36%, list=18%, signal=43% |
| 226 | ADIP\_HUMAN\_UP |  | 18 | -0.66 | -1.51 | 0.021 | 0.050 | 1.000 | 710 | tags=44%, list=12%, signal=51% |
| 227 | HSA05120\_EPITHELIAL\_CELL\_SIGNALING\_IN\_HELICOBACTER\_PYLORI\_INFECTION |  | 35 | -0.59 | -1.51 | 0.014 | 0.050 | 1.000 | 1321 | tags=46%, list=23%, signal=59% |
| 228 | HDACI\_COLON\_BUT24HRS\_UP |  | 31 | -0.60 | -1.51 | 0.017 | 0.051 | 1.000 | 1390 | tags=48%, list=24%, signal=64% |
| 229 | UVB\_NHEK3\_C8 |  | 34 | -0.59 | -1.51 | 0.012 | 0.051 | 1.000 | 1070 | tags=38%, list=19%, signal=47% |
| 230 | CALRES\_MOUSE\_NEOCORTEX\_UP |  | 23 | -0.62 | -1.51 | 0.027 | 0.052 | 1.000 | 1039 | tags=43%, list=18%, signal=53% |
| 231 | AGED\_MOUSE\_CEREBELLUM\_UP |  | 27 | -0.61 | -1.50 | 0.021 | 0.053 | 1.000 | 542 | tags=30%, list=9%, signal=33% |
| 232 | WERNER\_FIBRO\_UP |  | 22 | -0.62 | -1.50 | 0.021 | 0.053 | 1.000 | 1217 | tags=50%, list=21%, signal=63% |
| 233 | HYPOXIA\_REVIEW |  | 32 | -0.60 | -1.50 | 0.016 | 0.056 | 1.000 | 1235 | tags=50%, list=22%, signal=63% |
| 234 | OXSTRESS\_RPE\_HNETBH\_DN |  | 18 | -0.65 | -1.49 | 0.035 | 0.057 | 1.000 | 399 | tags=33%, list=7%, signal=36% |
| 235 | ZHAN\_MULTIPLE\_MYELOMA\_VS\_NORMAL\_DN |  | 15 | -0.66 | -1.49 | 0.025 | 0.057 | 1.000 | 1337 | tags=67%, list=23%, signal=87% |
| 236 | PASSERINI\_PROLIFERATION |  | 29 | -0.60 | -1.49 | 0.021 | 0.058 | 1.000 | 1611 | tags=66%, list=28%, signal=91% |
| 237 | ST\_T\_CELL\_SIGNAL\_TRANSDUCTION |  | 25 | -0.62 | -1.49 | 0.020 | 0.057 | 1.000 | 1345 | tags=48%, list=23%, signal=62% |
| 238 | HDACI\_COLON\_BUT12HRS\_UP |  | 18 | -0.64 | -1.49 | 0.030 | 0.060 | 1.000 | 1240 | tags=50%, list=22%, signal=64% |
| 239 | ADIPOGENESIS\_HMSC\_CLASS3\_UP |  | 30 | -0.60 | -1.49 | 0.026 | 0.061 | 1.000 | 919 | tags=33%, list=16%, signal=40% |
| 240 | APPEL\_IMATINIB\_UP |  | 20 | -0.63 | -1.49 | 0.031 | 0.061 | 1.000 | 1337 | tags=55%, list=23%, signal=72% |
| 241 | ECMPATHWAY |  | 16 | -0.66 | -1.49 | 0.034 | 0.062 | 1.000 | 1243 | tags=56%, list=22%, signal=72% |
| 242 | LINDSTEDT\_DEND\_8H\_VS\_48H\_DN |  | 29 | -0.59 | -1.48 | 0.018 | 0.062 | 1.000 | 698 | tags=38%, list=12%, signal=43% |
| 243 | HSA04650\_NATURAL\_KILLER\_CELL\_MEDIATED\_CYTOTOXICITY |  | 45 | -0.56 | -1.48 | 0.015 | 0.063 | 1.000 | 1243 | tags=42%, list=22%, signal=54% |
| 244 | ADIP\_DIFF\_CLUSTER1 |  | 24 | -0.61 | -1.48 | 0.036 | 0.064 | 1.000 | 995 | tags=54%, list=17%, signal=65% |
| 245 | NGUYEN\_KERATO\_DN |  | 34 | -0.58 | -1.48 | 0.033 | 0.064 | 1.000 | 1231 | tags=44%, list=22%, signal=56% |
| 246 | TNFALPHA\_ADIP\_DN |  | 25 | -0.61 | -1.48 | 0.024 | 0.065 | 1.000 | 764 | tags=28%, list=13%, signal=32% |
| 247 | LEE\_CIP\_UP |  | 15 | -0.66 | -1.48 | 0.033 | 0.065 | 1.000 | 533 | tags=47%, list=9%, signal=51% |
| 248 | CARIES\_PULP\_HIGH\_UP |  | 32 | -0.59 | -1.48 | 0.020 | 0.066 | 1.000 | 1160 | tags=44%, list=20%, signal=55% |
| 249 | SERUM\_FIBROBLAST\_CORE\_DN |  | 79 | -0.52 | -1.47 | 0.005 | 0.067 | 1.000 | 1463 | tags=42%, list=26%, signal=55% |
| 250 | HSA05215\_PROSTATE\_CANCER |  | 42 | -0.56 | -1.47 | 0.022 | 0.067 | 1.000 | 1329 | tags=43%, list=23%, signal=55% |
| 251 | HSA04070\_PHOSPHATIDYLINOSITOL\_SIGNALING\_SYSTEM |  | 32 | -0.59 | -1.47 | 0.029 | 0.067 | 1.000 | 1300 | tags=53%, list=23%, signal=68% |
| 252 | ET743PT650\_COLONCA\_DN |  | 21 | -0.62 | -1.47 | 0.035 | 0.067 | 1.000 | 1271 | tags=43%, list=22%, signal=55% |
| 253 | TAKEDA\_NUP8\_HOXA9\_16D\_UP |  | 47 | -0.54 | -1.47 | 0.008 | 0.068 | 1.000 | 875 | tags=47%, list=15%, signal=55% |
| 254 | MYOD\_NIH3T3\_UP |  | 29 | -0.58 | -1.47 | 0.034 | 0.067 | 1.000 | 1501 | tags=52%, list=26%, signal=70% |
| 255 | BRCA2\_BRCA1\_DN |  | 20 | -0.62 | -1.47 | 0.038 | 0.067 | 1.000 | 1130 | tags=35%, list=20%, signal=43% |
| 256 | HSA05220\_CHRONIC\_MYELOID\_LEUKEMIA |  | 41 | -0.56 | -1.47 | 0.031 | 0.067 | 1.000 | 1415 | tags=49%, list=25%, signal=64% |
| 257 | TPA\_SENS\_EARLY\_UP |  | 23 | -0.61 | -1.47 | 0.028 | 0.068 | 1.000 | 233 | tags=22%, list=4%, signal=23% |
| 258 | HSA04370\_VEGF\_SIGNALING\_PATHWAY |  | 28 | -0.60 | -1.47 | 0.022 | 0.068 | 1.000 | 1190 | tags=46%, list=21%, signal=58% |
| 259 | LINDSTEDT\_DEND\_8H\_VS\_48H\_UP |  | 29 | -0.59 | -1.47 | 0.028 | 0.072 | 1.000 | 1134 | tags=45%, list=20%, signal=56% |
| 260 | HSA03320\_PPAR\_SIGNALING\_PATHWAY |  | 28 | -0.58 | -1.46 | 0.035 | 0.072 | 1.000 | 306 | tags=29%, list=5%, signal=30% |
| 261 | RAY\_P210\_DIFF |  | 18 | -0.63 | -1.46 | 0.032 | 0.073 | 1.000 | 825 | tags=50%, list=14%, signal=58% |
| 262 | HEMATOPOESIS\_RELATED\_TRANSCRIPTION\_FACTORS |  | 37 | -0.57 | -1.46 | 0.020 | 0.073 | 1.000 | 692 | tags=30%, list=12%, signal=34% |
| 263 | TARTE\_PC |  | 49 | -0.55 | -1.46 | 0.010 | 0.077 | 1.000 | 1636 | tags=57%, list=29%, signal=79% |
| 264 | HDACI\_COLON\_CUR\_DN |  | 18 | -0.63 | -1.46 | 0.043 | 0.077 | 1.000 | 1480 | tags=50%, list=26%, signal=67% |
| 265 | VEGF\_MMMEC\_6HRS\_UP |  | 16 | -0.64 | -1.46 | 0.040 | 0.078 | 1.000 | 1256 | tags=69%, list=22%, signal=88% |
| 266 | CHAUHAN\_2ME2 |  | 18 | -0.62 | -1.45 | 0.044 | 0.078 | 1.000 | 955 | tags=33%, list=17%, signal=40% |
| 267 | NAB\_LUNG\_DN |  | 21 | -0.62 | -1.45 | 0.036 | 0.079 | 1.000 | 931 | tags=43%, list=16%, signal=51% |
| 268 | ET743\_SARCOMA\_72HRS\_UP |  | 22 | -0.60 | -1.45 | 0.038 | 0.081 | 1.000 | 1035 | tags=41%, list=18%, signal=50% |
| 269 | HDACI\_COLON\_BUT48HRS\_UP |  | 32 | -0.57 | -1.45 | 0.033 | 0.081 | 1.000 | 1608 | tags=59%, list=28%, signal=82% |
| 270 | ALZHEIMERS\_INCIPIENT\_UP |  | 134 | -0.50 | -1.45 | 0.003 | 0.081 | 1.000 | 1180 | tags=29%, list=21%, signal=36% |
| 271 | BRCA1KO\_MEF\_DN |  | 38 | -0.56 | -1.45 | 0.038 | 0.081 | 1.000 | 919 | tags=26%, list=16%, signal=31% |
| 272 | ZHAN\_MM\_CD138\_HP\_VS\_REST |  | 15 | -0.65 | -1.45 | 0.042 | 0.082 | 1.000 | 857 | tags=47%, list=15%, signal=55% |
| 273 | PHOSPHATIDYLINOSITOL\_SIGNALING\_SYSTEM |  | 43 | -0.55 | -1.45 | 0.027 | 0.082 | 1.000 | 1185 | tags=40%, list=21%, signal=49% |
| 274 | ST\_GA13\_PATHWAY |  | 16 | -0.65 | -1.45 | 0.037 | 0.082 | 1.000 | 1321 | tags=50%, list=23%, signal=65% |
| 275 | MOREAUX\_TACI\_HI\_IN\_BMPC |  | 15 | -0.64 | -1.45 | 0.043 | 0.083 | 1.000 | 1085 | tags=47%, list=19%, signal=57% |
| 276 | HSA04730\_LONG\_TERM\_DEPRESSION |  | 30 | -0.58 | -1.44 | 0.034 | 0.084 | 1.000 | 1332 | tags=47%, list=23%, signal=61% |
| 277 | NUCLEAR\_RECEPTORS |  | 15 | -0.64 | -1.44 | 0.049 | 0.084 | 1.000 | 1276 | tags=60%, list=22%, signal=77% |
| 278 | GSK3PATHWAY |  | 15 | -0.65 | -1.44 | 0.051 | 0.087 | 1.000 | 1329 | tags=53%, list=23%, signal=69% |
| 279 | HSA05214\_GLIOMA |  | 30 | -0.58 | -1.44 | 0.031 | 0.088 | 1.000 | 1243 | tags=47%, list=22%, signal=59% |
| 280 | G\_PROTEIN\_SIGNALING |  | 40 | -0.55 | -1.44 | 0.034 | 0.088 | 1.000 | 1207 | tags=38%, list=21%, signal=47% |
| 281 | VANASSE\_BCL2\_TARGETS |  | 37 | -0.57 | -1.44 | 0.031 | 0.088 | 1.000 | 696 | tags=30%, list=12%, signal=34% |
| 282 | ST\_DIFFERENTIATION\_PATHWAY\_IN\_PC12\_CELLS |  | 20 | -0.60 | -1.44 | 0.052 | 0.088 | 1.000 | 1243 | tags=40%, list=22%, signal=51% |
| 283 | GH\_AUTOCRINE\_UP |  | 59 | -0.52 | -1.44 | 0.028 | 0.089 | 1.000 | 830 | tags=25%, list=15%, signal=29% |
| 284 | CMV\_8HRS\_DN |  | 20 | -0.61 | -1.44 | 0.051 | 0.089 | 1.000 | 1790 | tags=75%, list=31%, signal=109% |
| 285 | AGED\_MOUSE\_NEOCORTEX\_UP |  | 36 | -0.56 | -1.43 | 0.039 | 0.090 | 1.000 | 1000 | tags=28%, list=17%, signal=33% |
| 286 | HDACI\_COLON\_BUT\_UP |  | 62 | -0.52 | -1.43 | 0.023 | 0.090 | 1.000 | 1390 | tags=44%, list=24%, signal=57% |
| 287 | 4NQO\_ESR\_WS\_UNREG |  | 17 | -0.63 | -1.43 | 0.044 | 0.090 | 1.000 | 1193 | tags=41%, list=21%, signal=52% |
| 288 | AS3\_FIBRO\_C2 |  | 16 | -0.62 | -1.43 | 0.046 | 0.091 | 1.000 | 1256 | tags=50%, list=22%, signal=64% |
| 289 | LINDSTEDT\_DEND\_UP |  | 23 | -0.59 | -1.43 | 0.048 | 0.097 | 1.000 | 1404 | tags=52%, list=25%, signal=69% |
| 290 | HDACI\_COLON\_CUR\_UP |  | 42 | -0.55 | -1.42 | 0.039 | 0.097 | 1.000 | 1367 | tags=45%, list=24%, signal=59% |
| 291 | INTEGRINPATHWAY |  | 24 | -0.58 | -1.42 | 0.058 | 0.101 | 1.000 | 2174 | tags=79%, list=38%, signal=127% |
| 292 | MUNSHI\_MM\_UP |  | 26 | -0.57 | -1.42 | 0.065 | 0.103 | 1.000 | 1604 | tags=50%, list=28%, signal=69% |
| 293 | KLEIN\_PEL\_UP |  | 20 | -0.60 | -1.42 | 0.070 | 0.103 | 1.000 | 1000 | tags=40%, list=17%, signal=48% |
| 294 | AS3\_FIBRO\_C1 |  | 16 | -0.62 | -1.42 | 0.069 | 0.103 | 1.000 | 1256 | tags=50%, list=22%, signal=64% |
| 295 | HDACI\_COLON\_TSA\_DN |  | 32 | -0.56 | -1.42 | 0.030 | 0.103 | 1.000 | 670 | tags=25%, list=12%, signal=28% |
| 296 | HSA05213\_ENDOMETRIAL\_CANCER |  | 30 | -0.57 | -1.42 | 0.044 | 0.104 | 1.000 | 1329 | tags=47%, list=23%, signal=60% |
| 297 | HDACI\_COLON\_CURSUL\_UP |  | 18 | -0.61 | -1.42 | 0.061 | 0.104 | 1.000 | 1160 | tags=50%, list=20%, signal=63% |
| 298 | HDACI\_COLON\_TSA2HRS\_UP |  | 22 | -0.59 | -1.41 | 0.050 | 0.104 | 1.000 | 1240 | tags=59%, list=22%, signal=75% |
| 299 | CMV\_HCMV\_TIMECOURSE\_48HRS\_DN |  | 42 | -0.54 | -1.41 | 0.046 | 0.104 | 1.000 | 666 | tags=29%, list=12%, signal=32% |
| 300 | PYK2PATHWAY |  | 19 | -0.60 | -1.41 | 0.056 | 0.106 | 1.000 | 2174 | tags=84%, list=38%, signal=135% |
| 301 | ZHAN\_MM\_MOLECULAR\_CLASSI\_UP |  | 16 | -0.63 | -1.41 | 0.066 | 0.108 | 1.000 | 1140 | tags=50%, list=20%, signal=62% |
| 302 | ZHAN\_TONSIL\_BONEMARROW |  | 21 | -0.59 | -1.41 | 0.058 | 0.110 | 1.000 | 1271 | tags=62%, list=22%, signal=79% |
| 303 | CMV\_HCMV\_TIMECOURSE\_20HRS\_DN |  | 18 | -0.61 | -1.41 | 0.053 | 0.110 | 1.000 | 1231 | tags=44%, list=22%, signal=56% |
| 304 | TENEDINI\_MEGAKARYOCYTIC\_GENES |  | 21 | -0.59 | -1.40 | 0.068 | 0.114 | 1.000 | 1220 | tags=48%, list=21%, signal=60% |
| 305 | CTNNB1\_ONCOGENIC\_SIGNATURE |  | 32 | -0.55 | -1.40 | 0.050 | 0.115 | 1.000 | 1283 | tags=31%, list=22%, signal=40% |
| 306 | CMV\_HCMV\_TIMECOURSE\_16HRS\_UP |  | 34 | -0.55 | -1.40 | 0.052 | 0.115 | 1.000 | 417 | tags=21%, list=7%, signal=22% |
| 307 | TPA\_SENS\_MIDDLE\_DN |  | 116 | -0.49 | -1.40 | 0.010 | 0.116 | 1.000 | 1179 | tags=34%, list=21%, signal=43% |
| 308 | OXSTRESS\_RPE\_H2O2TBH\_DN |  | 16 | -0.62 | -1.40 | 0.067 | 0.118 | 1.000 | 1256 | tags=50%, list=22%, signal=64% |
| 309 | ASTON\_DEPRESSION\_DN |  | 48 | -0.53 | -1.40 | 0.037 | 0.118 | 1.000 | 1184 | tags=29%, list=21%, signal=36% |
| 310 | SANSOM\_APC\_4\_DN |  | 21 | -0.59 | -1.40 | 0.066 | 0.118 | 1.000 | 591 | tags=33%, list=10%, signal=37% |
| 311 | HSA05130\_PATHOGENIC\_ESCHERICHIA\_COLI\_INFECTION\_EHEC |  | 16 | -0.61 | -1.40 | 0.087 | 0.120 | 1.000 | 1694 | tags=63%, list=30%, signal=89% |
| 312 | HYPOXIA\_NORMAL\_UP |  | 85 | -0.50 | -1.39 | 0.020 | 0.120 | 1.000 | 1648 | tags=46%, list=29%, signal=63% |
| 313 | BRCA\_PROGNOSIS\_POS |  | 18 | -0.60 | -1.39 | 0.070 | 0.122 | 1.000 | 999 | tags=39%, list=17%, signal=47% |
| 314 | PDGFPATHWAY |  | 18 | -0.59 | -1.39 | 0.076 | 0.124 | 1.000 | 1473 | tags=56%, list=26%, signal=75% |
| 315 | PASSERINI\_TRANSCRIPTION |  | 32 | -0.54 | -1.39 | 0.051 | 0.124 | 1.000 | 1530 | tags=44%, list=27%, signal=59% |
| 316 | CREB\_BRAIN\_8WKS\_UP |  | 20 | -0.59 | -1.39 | 0.066 | 0.124 | 1.000 | 649 | tags=30%, list=11%, signal=34% |
| 317 | BRCA\_ER\_POS |  | 162 | -0.48 | -1.39 | 0.002 | 0.124 | 1.000 | 1581 | tags=43%, list=28%, signal=58% |
| 318 | AGED\_MOUSE\_HYPOTH\_DN |  | 21 | -0.59 | -1.39 | 0.071 | 0.125 | 1.000 | 1332 | tags=48%, list=23%, signal=62% |
| 319 | HSA04010\_MAPK\_SIGNALING\_PATHWAY |  | 90 | -0.49 | -1.39 | 0.022 | 0.125 | 1.000 | 1226 | tags=37%, list=21%, signal=46% |
| 320 | FLECHNER\_KIDNEY\_TRANSPLANT\_REJECTION\_DN |  | 229 | -0.47 | -1.39 | 0.000 | 0.127 | 1.000 | 941 | tags=26%, list=16%, signal=30% |
| 321 | CPR\_NULL\_LIVER\_UP |  | 15 | -0.62 | -1.39 | 0.079 | 0.127 | 1.000 | 861 | tags=47%, list=15%, signal=55% |
| 322 | HSA04930\_TYPE\_II\_DIABETES\_MELLITUS |  | 15 | -0.61 | -1.38 | 0.073 | 0.127 | 1.000 | 1185 | tags=47%, list=21%, signal=59% |
| 323 | GUO\_HEX\_UP |  | 35 | -0.54 | -1.38 | 0.062 | 0.129 | 1.000 | 1250 | tags=40%, list=22%, signal=51% |
| 324 | UVC\_LOW\_ALL\_DN |  | 22 | -0.58 | -1.38 | 0.067 | 0.129 | 1.000 | 1393 | tags=41%, list=24%, signal=54% |
| 325 | POD1\_KO\_UP |  | 133 | -0.49 | -1.38 | 0.004 | 0.128 | 1.000 | 618 | tags=26%, list=11%, signal=28% |
| 326 | HSA05131\_PATHOGENIC\_ESCHERICHIA\_COLI\_INFECTION\_EPEC |  | 16 | -0.61 | -1.38 | 0.086 | 0.134 | 1.000 | 1694 | tags=63%, list=30%, signal=89% |
| 327 | OLD\_FIBRO\_UP |  | 26 | -0.56 | -1.38 | 0.081 | 0.134 | 1.000 | 512 | tags=19%, list=9%, signal=21% |
| 328 | HSC\_HSCANDPROGENITORS\_ADULT |  | 204 | -0.47 | -1.38 | 0.004 | 0.135 | 1.000 | 964 | tags=25%, list=17%, signal=29% |
| 329 | TPA\_SENS\_EARLY\_DN |  | 110 | -0.48 | -1.38 | 0.018 | 0.135 | 1.000 | 1052 | tags=31%, list=18%, signal=37% |
| 330 | GENOTOXINS\_4HRS\_DISCR |  | 15 | -0.62 | -1.37 | 0.084 | 0.136 | 1.000 | 846 | tags=40%, list=15%, signal=47% |
| 331 | CMV\_HCMV\_TIMECOURSE\_ALL\_DN |  | 162 | -0.47 | -1.37 | 0.010 | 0.136 | 1.000 | 1015 | tags=29%, list=18%, signal=34% |
| 332 | NEMETH\_TNF\_UP |  | 32 | -0.54 | -1.37 | 0.058 | 0.139 | 1.000 | 1514 | tags=47%, list=26%, signal=63% |
| 333 | FSH\_OVARY\_MCV152\_UP |  | 30 | -0.55 | -1.37 | 0.066 | 0.143 | 1.000 | 1329 | tags=43%, list=23%, signal=56% |
| 334 | ZHANG\_EFT\_EWSFLI1\_UP |  | 30 | -0.54 | -1.37 | 0.073 | 0.143 | 1.000 | 1143 | tags=47%, list=20%, signal=58% |
| 335 | UVB\_NHEK3\_ALL |  | 197 | -0.47 | -1.37 | 0.000 | 0.144 | 1.000 | 1091 | tags=27%, list=19%, signal=33% |
| 336 | MENSE\_HYPOXIA\_UP |  | 36 | -0.53 | -1.36 | 0.060 | 0.145 | 1.000 | 1089 | tags=33%, list=19%, signal=41% |
| 337 | OLDONLY\_FIBRO\_DN |  | 21 | -0.58 | -1.36 | 0.088 | 0.146 | 1.000 | 783 | tags=29%, list=14%, signal=33% |
| 338 | AT1RPATHWAY |  | 17 | -0.60 | -1.36 | 0.085 | 0.147 | 1.000 | 1243 | tags=53%, list=22%, signal=67% |
| 339 | BIOPEPTIDESPATHWAY |  | 18 | -0.58 | -1.36 | 0.097 | 0.147 | 1.000 | 2164 | tags=78%, list=38%, signal=125% |
| 340 | IRITANI\_ADPROX\_LYMPH |  | 56 | -0.50 | -1.36 | 0.037 | 0.148 | 1.000 | 1051 | tags=38%, list=18%, signal=45% |
| 341 | HDACI\_COLON\_SUL48HRS\_UP |  | 43 | -0.53 | -1.36 | 0.061 | 0.151 | 1.000 | 1240 | tags=37%, list=22%, signal=47% |
| 342 | GH\_EXOGENOUS\_ANY\_DN |  | 32 | -0.54 | -1.36 | 0.070 | 0.152 | 1.000 | 970 | tags=31%, list=17%, signal=37% |
| 343 | UV-CMV\_UNIQUE\_HCMV\_6HRS\_DN |  | 33 | -0.53 | -1.36 | 0.074 | 0.152 | 1.000 | 630 | tags=30%, list=11%, signal=34% |
| 344 | CMV\_HCMV\_6HRS\_DN |  | 16 | -0.60 | -1.36 | 0.125 | 0.153 | 1.000 | 1294 | tags=56%, list=23%, signal=72% |
| 345 | HDACI\_COLON\_BUT30MIN\_DN |  | 24 | -0.55 | -1.36 | 0.091 | 0.153 | 1.000 | 701 | tags=25%, list=12%, signal=28% |
| 346 | AGED\_MOUSE\_HIPPOCAMPUS\_ANY\_DN |  | 17 | -0.59 | -1.35 | 0.089 | 0.153 | 1.000 | 1845 | tags=76%, list=32%, signal=113% |
| 347 | CIRCADIAN\_EXERCISE |  | 17 | -0.58 | -1.35 | 0.105 | 0.153 | 1.000 | 2001 | tags=65%, list=35%, signal=99% |
| 348 | HSA04664\_FC\_EPSILON\_RI\_SIGNALING\_PATHWAY |  | 41 | -0.52 | -1.35 | 0.068 | 0.153 | 1.000 | 1185 | tags=41%, list=21%, signal=52% |
| 349 | CHIARETTI\_ZAP70\_DIFF |  | 31 | -0.53 | -1.35 | 0.076 | 0.153 | 1.000 | 381 | tags=19%, list=7%, signal=21% |
| 350 | ADIP\_DIFF\_CLUSTER3 |  | 18 | -0.59 | -1.35 | 0.097 | 0.154 | 1.000 | 525 | tags=22%, list=9%, signal=24% |
| 351 | MUNSHI\_MM\_VS\_PCS\_UP |  | 29 | -0.55 | -1.35 | 0.073 | 0.154 | 1.000 | 1793 | tags=55%, list=31%, signal=80% |
| 352 | TAKEDA\_NUP8\_HOXA9\_3D\_UP |  | 55 | -0.50 | -1.35 | 0.059 | 0.153 | 1.000 | 746 | tags=36%, list=13%, signal=41% |
| 353 | HSA05222\_SMALL\_CELL\_LUNG\_CANCER |  | 42 | -0.51 | -1.35 | 0.074 | 0.153 | 1.000 | 1415 | tags=43%, list=25%, signal=57% |
| 354 | LVAD\_HEARTFAILURE\_DN |  | 15 | -0.60 | -1.35 | 0.103 | 0.154 | 1.000 | 1021 | tags=53%, list=18%, signal=65% |
| 355 | GH\_EXOGENOUS\_ALL\_UP |  | 16 | -0.60 | -1.35 | 0.095 | 0.156 | 1.000 | 1479 | tags=50%, list=26%, signal=67% |
| 356 | BRG1\_ALAB\_UP |  | 17 | -0.59 | -1.34 | 0.093 | 0.161 | 1.000 | 1917 | tags=71%, list=33%, signal=106% |
| 357 | HDACI\_COLON\_SUL\_UP |  | 54 | -0.50 | -1.34 | 0.060 | 0.161 | 1.000 | 1512 | tags=43%, list=26%, signal=57% |
| 358 | KIM\_TH\_CELLS\_UP |  | 27 | -0.53 | -1.34 | 0.105 | 0.162 | 1.000 | 907 | tags=30%, list=16%, signal=35% |
| 359 | FRASOR\_ER\_DN |  | 19 | -0.58 | -1.34 | 0.097 | 0.161 | 1.000 | 1409 | tags=68%, list=25%, signal=90% |
| 360 | FCER1PATHWAY |  | 25 | -0.56 | -1.34 | 0.094 | 0.161 | 1.000 | 1243 | tags=44%, list=22%, signal=56% |
| 361 | HSA04912\_GNRH\_SIGNALING\_PATHWAY |  | 34 | -0.52 | -1.34 | 0.076 | 0.162 | 1.000 | 1157 | tags=41%, list=20%, signal=51% |
| 362 | UVB\_NHEK3\_C5 |  | 26 | -0.54 | -1.34 | 0.106 | 0.162 | 1.000 | 783 | tags=23%, list=14%, signal=27% |
| 363 | HSC\_MATURE\_ADULT |  | 147 | -0.47 | -1.34 | 0.008 | 0.168 | 1.000 | 1496 | tags=37%, list=26%, signal=48% |
| 364 | STEMCELL\_COMMON\_DN |  | 25 | -0.55 | -1.34 | 0.103 | 0.168 | 1.000 | 1110 | tags=40%, list=19%, signal=49% |
| 365 | HSC\_HSCANDPROGENITORS\_SHARED |  | 200 | -0.46 | -1.34 | 0.003 | 0.168 | 1.000 | 964 | tags=24%, list=17%, signal=28% |
| 366 | DORSAM\_HOXA9\_DN |  | 15 | -0.59 | -1.34 | 0.110 | 0.168 | 1.000 | 1530 | tags=53%, list=27%, signal=73% |
| 367 | HSA05211\_RENAL\_CELL\_CARCINOMA |  | 43 | -0.51 | -1.34 | 0.064 | 0.168 | 1.000 | 1974 | tags=63%, list=34%, signal=95% |
| 368 | LIN\_WNT\_UP |  | 24 | -0.55 | -1.33 | 0.101 | 0.170 | 1.000 | 1225 | tags=42%, list=21%, signal=53% |
| 369 | VENTRICLES\_UP |  | 79 | -0.48 | -1.33 | 0.047 | 0.172 | 1.000 | 1115 | tags=29%, list=19%, signal=36% |
| 370 | HSC\_HSCANDPROGENITORS\_FETAL |  | 200 | -0.46 | -1.33 | 0.002 | 0.173 | 1.000 | 964 | tags=24%, list=17%, signal=28% |
| 371 | GPCRPATHWAY |  | 16 | -0.59 | -1.33 | 0.117 | 0.174 | 1.000 | 1871 | tags=63%, list=33%, signal=93% |
| 372 | H2O2\_CSBRESCUED\_UP |  | 19 | -0.57 | -1.33 | 0.127 | 0.176 | 1.000 | 963 | tags=37%, list=17%, signal=44% |
| 373 | HSA05210\_COLORECTAL\_CANCER |  | 43 | -0.50 | -1.33 | 0.085 | 0.176 | 1.000 | 1384 | tags=44%, list=24%, signal=58% |
| 374 | SHEPARD\_CRASH\_AND\_BURN\_MUT\_VS\_WT\_UP |  | 51 | -0.50 | -1.33 | 0.072 | 0.177 | 1.000 | 1304 | tags=33%, list=23%, signal=43% |
| 375 | HDACI\_COLON\_BUT16HRS\_UP |  | 16 | -0.60 | -1.32 | 0.125 | 0.180 | 1.000 | 1381 | tags=50%, list=24%, signal=66% |
| 376 | WNT\_SIGNALING |  | 26 | -0.54 | -1.32 | 0.108 | 0.180 | 1.000 | 1329 | tags=35%, list=23%, signal=45% |
| 377 | BYSTRYKH\_HSC\_TRANS\_GLOCUS |  | 314 | -0.45 | -1.32 | 0.001 | 0.181 | 1.000 | 1091 | tags=24%, list=19%, signal=28% |
| 378 | TNFALPHA\_4HRS\_UP |  | 17 | -0.58 | -1.32 | 0.116 | 0.181 | 1.000 | 1321 | tags=35%, list=23%, signal=46% |
| 379 | TPA\_RESIST\_EARLY\_DN |  | 30 | -0.52 | -1.32 | 0.102 | 0.181 | 1.000 | 804 | tags=27%, list=14%, signal=31% |
| 380 | WELCSH\_BRCA\_UP |  | 19 | -0.57 | -1.32 | 0.116 | 0.182 | 1.000 | 1235 | tags=47%, list=22%, signal=60% |
| 381 | AGEING\_BRAIN\_DN |  | 51 | -0.49 | -1.32 | 0.074 | 0.183 | 1.000 | 1245 | tags=35%, list=22%, signal=45% |
| 382 | PENG\_LEUCINE\_UP |  | 46 | -0.50 | -1.32 | 0.076 | 0.187 | 1.000 | 971 | tags=30%, list=17%, signal=36% |
| 383 | GH\_EXOGENOUS\_LATE\_DN |  | 26 | -0.53 | -1.32 | 0.113 | 0.187 | 1.000 | 970 | tags=31%, list=17%, signal=37% |
| 384 | AGED\_MOUSE\_CORTEX\_DN |  | 24 | -0.55 | -1.32 | 0.101 | 0.188 | 1.000 | 1968 | tags=50%, list=34%, signal=76% |
| 385 | NF90\_DN |  | 15 | -0.59 | -1.31 | 0.142 | 0.190 | 1.000 | 505 | tags=27%, list=9%, signal=29% |
| 386 | FSH\_GRANULOSA\_UP |  | 28 | -0.52 | -1.31 | 0.112 | 0.197 | 1.000 | 1089 | tags=32%, list=19%, signal=40% |
| 387 | HSC\_HSC\_SHARED |  | 75 | -0.47 | -1.31 | 0.046 | 0.197 | 1.000 | 1191 | tags=31%, list=21%, signal=38% |
| 388 | WALKER\_MM\_SNP\_DIFF |  | 17 | -0.57 | -1.31 | 0.150 | 0.198 | 1.000 | 1396 | tags=47%, list=24%, signal=62% |
| 389 | SHEPARD\_CELL\_PROLIFERATION |  | 70 | -0.47 | -1.31 | 0.065 | 0.199 | 1.000 | 942 | tags=31%, list=16%, signal=37% |
| 390 | CELL\_PROLIFERATION |  | 70 | -0.47 | -1.30 | 0.066 | 0.202 | 1.000 | 942 | tags=31%, list=16%, signal=37% |
| 391 | REOVIRUS\_HEK293\_DN |  | 94 | -0.46 | -1.30 | 0.054 | 0.205 | 1.000 | 1480 | tags=41%, list=26%, signal=55% |
| 392 | INSULIN\_SIGNALING |  | 43 | -0.50 | -1.30 | 0.089 | 0.204 | 1.000 | 1345 | tags=37%, list=23%, signal=48% |
| 393 | HSA04612\_ANTIGEN\_PROCESSING\_AND\_PRESENTATION |  | 21 | -0.55 | -1.30 | 0.156 | 0.205 | 1.000 | 725 | tags=19%, list=13%, signal=22% |
| 394 | LH\_GRANULOSA\_UP |  | 29 | -0.52 | -1.30 | 0.115 | 0.206 | 1.000 | 1089 | tags=31%, list=19%, signal=38% |
| 395 | SMITH\_HCV\_INDUCED\_HCC\_UP |  | 15 | -0.57 | -1.30 | 0.141 | 0.206 | 1.000 | 1067 | tags=33%, list=19%, signal=41% |
| 396 | ST\_G\_ALPHA\_I\_PATHWAY |  | 16 | -0.58 | -1.30 | 0.135 | 0.209 | 1.000 | 1926 | tags=69%, list=34%, signal=103% |
| 397 | HDACI\_COLON\_SUL48HRS\_DN |  | 28 | -0.52 | -1.30 | 0.118 | 0.210 | 1.000 | 1547 | tags=46%, list=27%, signal=63% |
| 398 | MATSUDA\_VALPHAINKT\_DIFF |  | 174 | -0.45 | -1.29 | 0.017 | 0.210 | 1.000 | 881 | tags=27%, list=15%, signal=31% |
| 399 | METPATHWAY |  | 23 | -0.54 | -1.29 | 0.131 | 0.212 | 1.000 | 1926 | tags=61%, list=34%, signal=91% |
| 400 | UVC\_TTD-XPCS\_COMMON\_DN |  | 71 | -0.47 | -1.29 | 0.085 | 0.220 | 1.000 | 1206 | tags=30%, list=21%, signal=37% |
| 401 | HSA01031\_GLYCAN\_STRUCTURES\_BIOSYNTHESIS\_2 |  | 22 | -0.53 | -1.29 | 0.162 | 0.223 | 1.000 | 1467 | tags=45%, list=26%, signal=61% |
| 402 | IFN\_BETA\_GLIOMA\_UP |  | 25 | -0.52 | -1.28 | 0.150 | 0.225 | 1.000 | 1256 | tags=44%, list=22%, signal=56% |
| 403 | TAKEDA\_NUP8\_HOXA9\_8D\_UP |  | 40 | -0.48 | -1.28 | 0.117 | 0.227 | 1.000 | 385 | tags=33%, list=7%, signal=35% |
| 404 | VEGFPATHWAY |  | 17 | -0.55 | -1.28 | 0.155 | 0.232 | 1.000 | 1243 | tags=41%, list=22%, signal=52% |
| 405 | LEE\_TCELLS4\_UP |  | 17 | -0.56 | -1.28 | 0.176 | 0.231 | 1.000 | 1214 | tags=41%, list=21%, signal=52% |
| 406 | HSA04920\_ADIPOCYTOKINE\_SIGNALING\_PATHWAY |  | 34 | -0.50 | -1.28 | 0.135 | 0.231 | 1.000 | 230 | tags=15%, list=4%, signal=15% |
| 407 | BYSTRYKH\_HSC\_CIS\_GLOCUS |  | 48 | -0.47 | -1.28 | 0.122 | 0.233 | 1.000 | 1346 | tags=38%, list=24%, signal=49% |
| 408 | HSA05212\_PANCREATIC\_CANCER |  | 35 | -0.50 | -1.28 | 0.135 | 0.233 | 1.000 | 1473 | tags=49%, list=26%, signal=65% |
| 409 | IL2PATHWAY |  | 15 | -0.57 | -1.28 | 0.165 | 0.233 | 1.000 | 1243 | tags=40%, list=22%, signal=51% |
| 410 | BRENTANI\_TRANSCRIPTION\_FACTORS |  | 23 | -0.53 | -1.28 | 0.172 | 0.233 | 1.000 | 1258 | tags=43%, list=22%, signal=56% |
| 411 | HSC\_HSC\_FETAL |  | 81 | -0.46 | -1.28 | 0.083 | 0.233 | 1.000 | 1380 | tags=33%, list=24%, signal=43% |
| 412 | FATTY\_ACID\_METABOLISM |  | 26 | -0.52 | -1.27 | 0.142 | 0.234 | 1.000 | 799 | tags=23%, list=14%, signal=27% |
| 413 | GOLDRATH\_MEMORY |  | 28 | -0.51 | -1.27 | 0.147 | 0.236 | 1.000 | 1152 | tags=43%, list=20%, signal=53% |
| 414 | HSC\_HSC\_ADULT |  | 96 | -0.45 | -1.27 | 0.062 | 0.238 | 1.000 | 1191 | tags=29%, list=21%, signal=36% |
| 415 | GH\_GHRHR\_KO\_24HRS\_UP |  | 56 | -0.47 | -1.27 | 0.107 | 0.238 | 1.000 | 1134 | tags=30%, list=20%, signal=37% |
| 416 | ST\_DICTYOSTELIUM\_DISCOIDEUM\_CAMP\_CHEMOTAXIS\_PATHWAY |  | 21 | -0.54 | -1.27 | 0.164 | 0.238 | 1.000 | 1666 | tags=57%, list=29%, signal=80% |
| 417 | HSA00530\_AMINOSUGARS\_METABOLISM |  | 20 | -0.53 | -1.27 | 0.164 | 0.238 | 1.000 | 1558 | tags=50%, list=27%, signal=68% |
| 418 | OXSTRESS\_RPETWO\_DN |  | 50 | -0.47 | -1.27 | 0.130 | 0.242 | 1.000 | 1329 | tags=36%, list=23%, signal=46% |
| 419 | ET743\_SARCOMA\_UP |  | 27 | -0.51 | -1.27 | 0.154 | 0.241 | 1.000 | 1035 | tags=30%, list=18%, signal=36% |
| 420 | UVC\_XPCS\_4HR\_DN |  | 120 | -0.44 | -1.27 | 0.057 | 0.241 | 1.000 | 1231 | tags=29%, list=22%, signal=36% |
| 421 | HADDAD\_HPCLYMPHO\_ENRICHED |  | 113 | -0.44 | -1.27 | 0.067 | 0.241 | 1.000 | 1209 | tags=31%, list=21%, signal=38% |
| 422 | HADDAD\_HSC\_CD10\_UP |  | 104 | -0.45 | -1.27 | 0.068 | 0.240 | 1.000 | 692 | tags=23%, list=12%, signal=26% |
| 423 | HDACI\_COLON\_SUL30MIN\_DN |  | 18 | -0.54 | -1.26 | 0.174 | 0.244 | 1.000 | 570 | tags=22%, list=10%, signal=25% |
| 424 | ZHAN\_MMPC\_SIM\_BC\_AND\_MM |  | 23 | -0.52 | -1.26 | 0.179 | 0.245 | 1.000 | 476 | tags=17%, list=8%, signal=19% |
| 425 | EGFPATHWAY |  | 17 | -0.56 | -1.26 | 0.183 | 0.246 | 1.000 | 1473 | tags=53%, list=26%, signal=71% |
| 426 | XU\_CBP\_DN |  | 17 | -0.56 | -1.26 | 0.174 | 0.246 | 1.000 | 1960 | tags=65%, list=34%, signal=98% |
| 427 | IDX\_TSA\_DN\_CLUSTER5 |  | 25 | -0.51 | -1.26 | 0.182 | 0.255 | 1.000 | 1917 | tags=56%, list=33%, signal=84% |
| 428 | PARK\_MSCS\_LIN2 |  | 24 | -0.51 | -1.25 | 0.191 | 0.260 | 1.000 | 1113 | tags=33%, list=19%, signal=41% |
| 429 | LEE\_MYC\_E2F1\_UP |  | 18 | -0.54 | -1.25 | 0.195 | 0.264 | 1.000 | 1241 | tags=56%, list=22%, signal=71% |
| 430 | ALCALAY\_AML\_NPMC\_DN |  | 71 | -0.45 | -1.25 | 0.121 | 0.266 | 1.000 | 702 | tags=27%, list=12%, signal=30% |
| 431 | FLECHNER\_KIDNEY\_TRANSPLANT\_WELL\_UP |  | 260 | -0.42 | -1.25 | 0.029 | 0.267 | 1.000 | 1722 | tags=35%, list=30%, signal=47% |
| 432 | OLD\_FIBRO\_DN |  | 70 | -0.45 | -1.25 | 0.111 | 0.267 | 1.000 | 915 | tags=19%, list=16%, signal=22% |
| 433 | CELL\_GROWTH\_AND\_OR\_MAINTENANCE |  | 23 | -0.52 | -1.25 | 0.179 | 0.268 | 1.000 | 1062 | tags=35%, list=19%, signal=43% |
| 434 | GH\_AUTOCRINE\_DN |  | 43 | -0.46 | -1.25 | 0.155 | 0.269 | 1.000 | 1217 | tags=40%, list=21%, signal=50% |
| 435 | UVC\_TTD\_ALL\_DN |  | 176 | -0.43 | -1.24 | 0.046 | 0.273 | 1.000 | 1231 | tags=27%, list=22%, signal=34% |
| 436 | CORDERO\_KRAS\_KD\_VS\_CONTROL\_DN |  | 16 | -0.55 | -1.24 | 0.196 | 0.275 | 1.000 | 432 | tags=19%, list=8%, signal=20% |
| 437 | BRCA1\_OVEREXP\_PROSTATE\_DN |  | 35 | -0.48 | -1.24 | 0.173 | 0.279 | 1.000 | 1260 | tags=34%, list=22%, signal=44% |
| 438 | ROSS\_AML1\_ETO |  | 23 | -0.51 | -1.24 | 0.196 | 0.279 | 1.000 | 620 | tags=30%, list=11%, signal=34% |
| 439 | BRCA1\_OVEREXP\_UP |  | 81 | -0.44 | -1.24 | 0.098 | 0.278 | 1.000 | 1688 | tags=44%, list=29%, signal=62% |
| 440 | AGED\_MOUSE\_HIPPOCAMPUS\_ANY\_UP |  | 22 | -0.52 | -1.24 | 0.187 | 0.279 | 1.000 | 782 | tags=27%, list=14%, signal=31% |
| 441 | SMITH\_HTERT\_UP |  | 56 | -0.45 | -1.24 | 0.149 | 0.280 | 1.000 | 1088 | tags=25%, list=19%, signal=31% |
| 442 | KERATINOCYTEPATHWAY |  | 27 | -0.50 | -1.24 | 0.169 | 0.279 | 1.000 | 1321 | tags=44%, list=23%, signal=58% |
| 443 | HSC\_MATURE\_FETAL |  | 138 | -0.43 | -1.23 | 0.067 | 0.283 | 1.000 | 1712 | tags=38%, list=30%, signal=53% |
| 444 | HOFMANN\_MANTEL\_LYMPHOMA\_VS\_LYMPH\_NODES\_UP |  | 29 | -0.49 | -1.23 | 0.203 | 0.286 | 1.000 | 1385 | tags=38%, list=24%, signal=50% |
| 445 | CROMER\_HYPOPHARYNGEAL\_MET\_VS\_NON\_UP |  | 36 | -0.48 | -1.23 | 0.178 | 0.289 | 1.000 | 1329 | tags=42%, list=23%, signal=54% |
| 446 | HDACI\_COLON\_TSABUT\_UP |  | 22 | -0.51 | -1.23 | 0.185 | 0.292 | 1.000 | 1381 | tags=45%, list=24%, signal=60% |
| 447 | RUIZ\_TENASCIN\_TARGETS |  | 26 | -0.50 | -1.23 | 0.193 | 0.292 | 1.000 | 427 | tags=23%, list=7%, signal=25% |
| 448 | IRS1\_KO\_ADIP\_UP |  | 36 | -0.47 | -1.22 | 0.187 | 0.298 | 1.000 | 1058 | tags=33%, list=18%, signal=41% |
| 449 | UEDA\_MOUSE\_LIVER |  | 47 | -0.46 | -1.22 | 0.168 | 0.298 | 1.000 | 1854 | tags=51%, list=32%, signal=75% |
| 450 | HDACI\_COLON\_SUL\_DN |  | 81 | -0.44 | -1.22 | 0.133 | 0.301 | 1.000 | 804 | tags=21%, list=14%, signal=24% |
| 451 | P53\_SIGNALING |  | 43 | -0.47 | -1.22 | 0.169 | 0.302 | 1.000 | 1210 | tags=33%, list=21%, signal=41% |
| 452 | OXSTRESS\_RPETHREE\_DN |  | 15 | -0.54 | -1.22 | 0.240 | 0.305 | 1.000 | 1459 | tags=53%, list=25%, signal=71% |
| 453 | H2O2\_CSBRESCUED\_C1\_UP |  | 15 | -0.55 | -1.22 | 0.226 | 0.307 | 1.000 | 859 | tags=33%, list=15%, signal=39% |
| 454 | TPOPATHWAY |  | 15 | -0.55 | -1.22 | 0.209 | 0.310 | 1.000 | 2037 | tags=73%, list=36%, signal=114% |
| 455 | HSC\_MATURE\_SHARED |  | 112 | -0.42 | -1.21 | 0.120 | 0.313 | 1.000 | 1712 | tags=39%, list=30%, signal=55% |
| 456 | HSA05223\_NON\_SMALL\_CELL\_LUNG\_CANCER |  | 28 | -0.49 | -1.21 | 0.216 | 0.314 | 1.000 | 1185 | tags=36%, list=21%, signal=45% |
| 457 | ROME\_INSULIN\_2F\_UP |  | 84 | -0.44 | -1.21 | 0.134 | 0.316 | 1.000 | 663 | tags=14%, list=12%, signal=16% |
| 458 | ELECTRON\_TRANSPORT |  | 21 | -0.51 | -1.21 | 0.226 | 0.326 | 1.000 | 901 | tags=43%, list=16%, signal=51% |
| 459 | NADLER\_OBESITY\_HYPERGLYCEMIA |  | 20 | -0.52 | -1.20 | 0.230 | 0.326 | 1.000 | 790 | tags=25%, list=14%, signal=29% |
| 460 | HSA01030\_GLYCAN\_STRUCTURES\_BIOSYNTHESIS\_1 |  | 43 | -0.46 | -1.20 | 0.206 | 0.330 | 1.000 | 2191 | tags=58%, list=38%, signal=93% |
| 461 | N\_GLYCAN\_BIOSYNTHESIS |  | 17 | -0.52 | -1.20 | 0.248 | 0.329 | 1.000 | 2045 | tags=65%, list=36%, signal=100% |
| 462 | AGED\_MOUSE\_NEOCORTEX\_DN |  | 17 | -0.53 | -1.20 | 0.228 | 0.330 | 1.000 | 495 | tags=18%, list=9%, signal=19% |
| 463 | FALT\_BCLL\_DN |  | 22 | -0.50 | -1.20 | 0.242 | 0.340 | 1.000 | 1417 | tags=45%, list=25%, signal=60% |
| 464 | AS3\_FIBRO\_C3 |  | 24 | -0.49 | -1.20 | 0.243 | 0.341 | 1.000 | 1256 | tags=33%, list=22%, signal=43% |
| 465 | SIG\_CD40PATHWAYMAP |  | 15 | -0.54 | -1.19 | 0.251 | 0.346 | 1.000 | 1814 | tags=53%, list=32%, signal=78% |
| 466 | ROSS\_FAB\_M7 |  | 30 | -0.47 | -1.19 | 0.227 | 0.346 | 1.000 | 1108 | tags=30%, list=19%, signal=37% |
| 467 | BRCA\_ER\_NEG |  | 400 | -0.40 | -1.19 | 0.024 | 0.346 | 1.000 | 995 | tags=24%, list=17%, signal=27% |
| 468 | AGED\_RHESUS\_DN |  | 58 | -0.44 | -1.19 | 0.186 | 0.349 | 1.000 | 1406 | tags=28%, list=25%, signal=36% |
| 469 | BRCA1\_MES\_UP |  | 24 | -0.49 | -1.19 | 0.241 | 0.349 | 1.000 | 1163 | tags=29%, list=20%, signal=36% |
| 470 | GN\_CAMP\_GRANULOSA\_UP |  | 18 | -0.52 | -1.19 | 0.255 | 0.352 | 1.000 | 1332 | tags=33%, list=23%, signal=43% |
| 471 | G13\_SIGNALING\_PATHWAY |  | 18 | -0.52 | -1.19 | 0.263 | 0.353 | 1.000 | 1802 | tags=56%, list=31%, signal=81% |
| 472 | HSA05110\_CHOLERA\_INFECTION |  | 21 | -0.50 | -1.18 | 0.263 | 0.360 | 1.000 | 1245 | tags=29%, list=22%, signal=36% |
| 473 | GLEEVECPATHWAY |  | 17 | -0.51 | -1.18 | 0.247 | 0.361 | 1.000 | 2037 | tags=71%, list=36%, signal=109% |
| 474 | AS3\_FIBRO\_UP |  | 24 | -0.49 | -1.18 | 0.249 | 0.363 | 1.000 | 1256 | tags=33%, list=22%, signal=43% |
| 475 | GPCRDB\_CLASS\_A\_RHODOPSIN\_LIKE |  | 24 | -0.48 | -1.18 | 0.270 | 0.366 | 1.000 | 691 | tags=38%, list=12%, signal=42% |
| 476 | SIG\_PIP3\_SIGNALING\_IN\_CARDIAC\_MYOCTES |  | 30 | -0.47 | -1.18 | 0.246 | 0.366 | 1.000 | 1577 | tags=43%, list=28%, signal=59% |
| 477 | HDACI\_COLON\_SUL24HRS\_DN |  | 50 | -0.44 | -1.17 | 0.224 | 0.372 | 1.000 | 1547 | tags=36%, list=27%, signal=49% |
| 478 | TNFR1PATHWAY |  | 16 | -0.51 | -1.17 | 0.270 | 0.375 | 1.000 | 1157 | tags=44%, list=20%, signal=55% |
| 479 | UV-4NQO\_FIBRO\_UP |  | 16 | -0.51 | -1.17 | 0.255 | 0.378 | 1.000 | 1230 | tags=44%, list=21%, signal=56% |
| 480 | HDACI\_COLON\_SUL24HRS\_UP |  | 25 | -0.47 | -1.17 | 0.285 | 0.378 | 1.000 | 1160 | tags=36%, list=20%, signal=45% |
| 481 | ASTIER\_BCELL |  | 21 | -0.49 | -1.17 | 0.285 | 0.381 | 1.000 | 1062 | tags=29%, list=19%, signal=35% |
| 482 | LEE\_MYC\_DN |  | 22 | -0.49 | -1.17 | 0.282 | 0.380 | 1.000 | 981 | tags=36%, list=17%, signal=44% |
| 483 | BRG1\_H1299\_UP |  | 16 | -0.52 | -1.17 | 0.296 | 0.380 | 1.000 | 505 | tags=19%, list=9%, signal=21% |
| 484 | E2F3\_ONCOGENIC\_SIGNATURE |  | 77 | -0.42 | -1.17 | 0.209 | 0.379 | 1.000 | 939 | tags=29%, list=16%, signal=34% |
| 485 | UVB\_NHEK3\_C0 |  | 42 | -0.44 | -1.17 | 0.262 | 0.381 | 1.000 | 859 | tags=21%, list=15%, signal=25% |
| 486 | COCAINE\_BRAIN\_4WKS\_UP |  | 25 | -0.47 | -1.16 | 0.264 | 0.384 | 1.000 | 1210 | tags=28%, list=21%, signal=35% |
| 487 | UVC\_TTD\_4HR\_DN |  | 149 | -0.40 | -1.16 | 0.150 | 0.387 | 1.000 | 1231 | tags=25%, list=22%, signal=31% |
| 488 | HSA04620\_TOLL\_LIKE\_RECEPTOR\_SIGNALING\_PATHWAY |  | 47 | -0.44 | -1.16 | 0.260 | 0.388 | 1.000 | 1327 | tags=45%, list=23%, signal=58% |
| 489 | LEE\_TCELLS2\_UP |  | 422 | -0.39 | -1.16 | 0.042 | 0.394 | 1.000 | 1050 | tags=26%, list=18%, signal=29% |
| 490 | GH\_GHRHR\_KO\_6HRS\_UP |  | 31 | -0.46 | -1.16 | 0.267 | 0.394 | 1.000 | 559 | tags=16%, list=10%, signal=18% |
| 491 | FERNANDEZ\_MYC\_TARGETS |  | 64 | -0.42 | -1.16 | 0.243 | 0.395 | 1.000 | 1045 | tags=27%, list=18%, signal=32% |
| 492 | BAF57\_BT549\_DN |  | 116 | -0.40 | -1.15 | 0.208 | 0.405 | 1.000 | 650 | tags=16%, list=11%, signal=18% |
| 493 | ZHAN\_MULTIPLE\_MYELOMA\_VS\_NORMAL\_UP |  | 26 | -0.47 | -1.15 | 0.275 | 0.408 | 1.000 | 1721 | tags=46%, list=30%, signal=66% |
| 494 | IL1RPATHWAY |  | 16 | -0.51 | -1.15 | 0.307 | 0.408 | 1.000 | 1321 | tags=56%, list=23%, signal=73% |
| 495 | ASTIER\_FN\_DIFF |  | 21 | -0.49 | -1.15 | 0.295 | 0.412 | 1.000 | 1062 | tags=29%, list=19%, signal=35% |
| 496 | GAMMA-UV\_FIBRO\_UP |  | 17 | -0.50 | -1.14 | 0.293 | 0.420 | 1.000 | 630 | tags=24%, list=11%, signal=26% |
| 497 | REFRACTORY\_GASTRIC\_UP |  | 21 | -0.48 | -1.14 | 0.304 | 0.421 | 1.000 | 1053 | tags=33%, list=18%, signal=41% |
| 498 | HSA04720\_LONG\_TERM\_POTENTIATION |  | 26 | -0.46 | -1.14 | 0.305 | 0.420 | 1.000 | 2164 | tags=50%, list=38%, signal=80% |
| 499 | TPA\_RESIST\_MIDDLE\_DN |  | 47 | -0.43 | -1.14 | 0.256 | 0.424 | 1.000 | 1314 | tags=28%, list=23%, signal=36% |
| 500 | YU\_CMYC\_DN |  | 22 | -0.47 | -1.14 | 0.315 | 0.430 | 1.000 | 1464 | tags=55%, list=26%, signal=73% |
| 501 | HSA00510\_N\_GLYCAN\_BIOSYNTHESIS |  | 27 | -0.45 | -1.14 | 0.300 | 0.431 | 1.000 | 2110 | tags=56%, list=37%, signal=88% |
| 502 | RACCYCDPATHWAY |  | 15 | -0.51 | -1.14 | 0.316 | 0.431 | 1.000 | 1871 | tags=60%, list=33%, signal=89% |
| 503 | IGF\_VS\_PDGF\_DN |  | 20 | -0.48 | -1.13 | 0.335 | 0.436 | 1.000 | 589 | tags=35%, list=10%, signal=39% |
| 504 | HESS\_HOXAANMEIS1\_UP |  | 32 | -0.44 | -1.13 | 0.318 | 0.437 | 1.000 | 379 | tags=16%, list=7%, signal=17% |
| 505 | KENNY\_WNT\_UP |  | 23 | -0.46 | -1.13 | 0.336 | 0.443 | 1.000 | 432 | tags=22%, list=8%, signal=23% |
| 506 | UVB\_NHEK3\_C2 |  | 25 | -0.46 | -1.13 | 0.319 | 0.444 | 1.000 | 1892 | tags=44%, list=33%, signal=65% |
| 507 | SHEPARD\_BMYB\_MORPHOLINO\_UP |  | 61 | -0.41 | -1.13 | 0.298 | 0.444 | 1.000 | 1431 | tags=34%, list=25%, signal=45% |
| 508 | HESS\_HOXAANMEIS1\_DN |  | 32 | -0.44 | -1.12 | 0.312 | 0.450 | 1.000 | 379 | tags=16%, list=7%, signal=17% |
| 509 | UVC\_HIGH\_ALL\_DN |  | 141 | -0.39 | -1.12 | 0.235 | 0.451 | 1.000 | 1091 | tags=22%, list=19%, signal=26% |
| 510 | DORSAM\_HOXA9\_UP |  | 18 | -0.49 | -1.12 | 0.329 | 0.462 | 1.000 | 887 | tags=28%, list=15%, signal=33% |
| 511 | HSC\_STHSC\_ADULT |  | 18 | -0.49 | -1.12 | 0.357 | 0.463 | 1.000 | 1201 | tags=28%, list=21%, signal=35% |
| 512 | MYC\_ONCOGENIC\_SIGNATURE |  | 71 | -0.40 | -1.11 | 0.314 | 0.466 | 1.000 | 1404 | tags=37%, list=25%, signal=48% |
| 513 | SIG\_INSULIN\_RECEPTOR\_PATHWAY\_IN\_CARDIAC\_MYOCYTES |  | 23 | -0.46 | -1.11 | 0.328 | 0.470 | 1.000 | 1926 | tags=48%, list=34%, signal=72% |
| 514 | HPV31\_DN |  | 17 | -0.48 | -1.11 | 0.347 | 0.471 | 1.000 | 274 | tags=18%, list=5%, signal=18% |
| 515 | KNUDSEN\_PMNS\_DN |  | 107 | -0.39 | -1.11 | 0.295 | 0.476 | 1.000 | 1917 | tags=40%, list=33%, signal=59% |
| 516 | ERKPATHWAY |  | 15 | -0.50 | -1.11 | 0.364 | 0.478 | 1.000 | 1926 | tags=60%, list=34%, signal=90% |
| 517 | HSA04012\_ERBB\_SIGNALING\_PATHWAY |  | 40 | -0.42 | -1.10 | 0.337 | 0.485 | 1.000 | 1345 | tags=43%, list=23%, signal=55% |
| 518 | KENNY\_WNT\_DN |  | 23 | -0.46 | -1.10 | 0.346 | 0.485 | 1.000 | 1452 | tags=35%, list=25%, signal=46% |
| 519 | PENG\_GLUTAMINE\_UP |  | 115 | -0.39 | -1.10 | 0.286 | 0.484 | 1.000 | 1294 | tags=31%, list=23%, signal=40% |
| 520 | HSA00860\_PORPHYRIN\_AND\_CHLOROPHYLL\_METABOLISM |  | 17 | -0.49 | -1.10 | 0.348 | 0.484 | 1.000 | 1356 | tags=29%, list=24%, signal=38% |
| 521 | FALT\_BCLL\_UP |  | 19 | -0.47 | -1.10 | 0.357 | 0.489 | 1.000 | 1367 | tags=42%, list=24%, signal=55% |
| 522 | HIVNEFPATHWAY |  | 29 | -0.43 | -1.10 | 0.358 | 0.491 | 1.000 | 1476 | tags=31%, list=26%, signal=42% |
| 523 | UVB\_NHEK1\_UP |  | 52 | -0.41 | -1.10 | 0.336 | 0.491 | 1.000 | 1414 | tags=37%, list=25%, signal=48% |
| 524 | IFN\_BETA\_GLIOMA\_DN |  | 21 | -0.46 | -1.10 | 0.371 | 0.490 | 1.000 | 538 | tags=19%, list=9%, signal=21% |
| 525 | SANSOM\_APC\_LOSS4\_UP |  | 39 | -0.42 | -1.10 | 0.343 | 0.490 | 1.000 | 754 | tags=21%, list=13%, signal=23% |
| 526 | GHPATHWAY |  | 17 | -0.47 | -1.10 | 0.364 | 0.492 | 1.000 | 2164 | tags=65%, list=38%, signal=104% |
| 527 | HDACI\_COLON\_BUT24HRS\_DN |  | 42 | -0.42 | -1.09 | 0.343 | 0.493 | 1.000 | 570 | tags=14%, list=10%, signal=16% |
| 528 | CMV\_HCMV\_TIMECOURSE\_24HRS\_UP |  | 31 | -0.43 | -1.09 | 0.356 | 0.492 | 1.000 | 1220 | tags=35%, list=21%, signal=45% |
| 529 | HDACI\_COLON\_CLUSTER9 |  | 25 | -0.44 | -1.09 | 0.367 | 0.491 | 1.000 | 1236 | tags=32%, list=22%, signal=41% |
| 530 | LEE\_ACOX1\_DN |  | 17 | -0.48 | -1.09 | 0.346 | 0.493 | 1.000 | 618 | tags=29%, list=11%, signal=33% |
| 531 | HDACI\_COLON\_SUL16HRS\_DN |  | 22 | -0.46 | -1.09 | 0.368 | 0.494 | 1.000 | 668 | tags=18%, list=12%, signal=21% |
| 532 | TCELL\_ANERGIC\_UP |  | 28 | -0.44 | -1.09 | 0.370 | 0.495 | 1.000 | 1809 | tags=64%, list=32%, signal=94% |
| 533 | UVC\_HIGH\_D7\_DN |  | 17 | -0.48 | -1.09 | 0.372 | 0.501 | 1.000 | 724 | tags=24%, list=13%, signal=27% |
| 534 | FSH\_OVARY\_MCV152\_DN |  | 21 | -0.46 | -1.09 | 0.385 | 0.501 | 1.000 | 1179 | tags=29%, list=21%, signal=36% |
| 535 | PPARAPATHWAY |  | 27 | -0.44 | -1.09 | 0.375 | 0.500 | 1.000 | 1973 | tags=56%, list=34%, signal=84% |
| 536 | FLOTHO\_CASP8AP2\_MRD\_DIFF |  | 26 | -0.45 | -1.09 | 0.372 | 0.500 | 1.000 | 1859 | tags=54%, list=32%, signal=79% |
| 537 | UVC\_XPCS\_ALL\_DN |  | 248 | -0.37 | -1.09 | 0.272 | 0.499 | 1.000 | 1231 | tags=23%, list=22%, signal=28% |
| 538 | BYSTROM\_IL5\_DN |  | 39 | -0.42 | -1.09 | 0.349 | 0.498 | 1.000 | 1785 | tags=49%, list=31%, signal=70% |
| 539 | PGC |  | 156 | -0.38 | -1.09 | 0.288 | 0.499 | 1.000 | 1089 | tags=21%, list=19%, signal=25% |
| 540 | BLEO\_HUMAN\_LYMPH\_HIGH\_24HRS\_UP |  | 46 | -0.41 | -1.09 | 0.355 | 0.500 | 1.000 | 1917 | tags=50%, list=33%, signal=75% |
| 541 | ELECTRON\_TRANSPORTER\_ACTIVITY |  | 35 | -0.42 | -1.08 | 0.373 | 0.501 | 1.000 | 1267 | tags=37%, list=22%, signal=47% |
| 542 | HSA04150\_MTOR\_SIGNALING\_PATHWAY |  | 23 | -0.45 | -1.08 | 0.372 | 0.501 | 1.000 | 1185 | tags=39%, list=21%, signal=49% |
| 543 | FLECHNER\_KIDNEY\_TRANSPLANT\_WELL\_PBL\_DN |  | 21 | -0.46 | -1.08 | 0.367 | 0.502 | 1.000 | 759 | tags=19%, list=13%, signal=22% |
| 544 | COCAINE\_BRAIN\_5D\_UP |  | 25 | -0.44 | -1.08 | 0.395 | 0.502 | 1.000 | 1277 | tags=36%, list=22%, signal=46% |
| 545 | ROSS\_MLL\_FUSION |  | 33 | -0.43 | -1.08 | 0.383 | 0.506 | 1.000 | 1721 | tags=52%, list=30%, signal=73% |
| 546 | STRESS\_ARSENIC\_SPECIFIC\_UP |  | 63 | -0.39 | -1.08 | 0.378 | 0.514 | 1.000 | 1479 | tags=35%, list=26%, signal=47% |
| 547 | UVC\_XPCS\_8HR\_DN |  | 215 | -0.37 | -1.07 | 0.305 | 0.522 | 1.000 | 1231 | tags=22%, list=22%, signal=27% |
| 548 | UVC\_TTD\_4HR\_UP |  | 22 | -0.44 | -1.07 | 0.410 | 0.534 | 1.000 | 1422 | tags=41%, list=25%, signal=54% |
| 549 | HSA01510\_NEURODEGENERATIVE\_DISEASES |  | 16 | -0.47 | -1.06 | 0.404 | 0.536 | 1.000 | 2049 | tags=69%, list=36%, signal=107% |
| 550 | ZHAN\_MMPC\_SIM |  | 20 | -0.45 | -1.06 | 0.410 | 0.535 | 1.000 | 1414 | tags=40%, list=25%, signal=53% |
| 551 | CHESLER\_HIGHEST\_FOLD\_RANGE\_GENES |  | 22 | -0.44 | -1.06 | 0.413 | 0.534 | 1.000 | 2007 | tags=45%, list=35%, signal=70% |
| 552 | GH\_GHRHR\_KO\_24HRS\_DN |  | 82 | -0.38 | -1.06 | 0.382 | 0.533 | 1.000 | 735 | tags=11%, list=13%, signal=12% |
| 553 | CMV\_HCMV\_TIMECOURSE\_14HRS\_UP |  | 21 | -0.44 | -1.06 | 0.393 | 0.534 | 1.000 | 955 | tags=24%, list=17%, signal=28% |
| 554 | HSA04310\_WNT\_SIGNALING\_PATHWAY |  | 50 | -0.40 | -1.06 | 0.389 | 0.534 | 1.000 | 1332 | tags=24%, list=23%, signal=31% |
| 555 | CELL\_MOTILITY |  | 45 | -0.40 | -1.06 | 0.399 | 0.536 | 1.000 | 1222 | tags=36%, list=21%, signal=45% |
| 556 | ST\_JNK\_MAPK\_PATHWAY |  | 17 | -0.46 | -1.06 | 0.410 | 0.537 | 1.000 | 1243 | tags=35%, list=22%, signal=45% |
| 557 | GLYCOGEN\_METABOLISM |  | 18 | -0.46 | -1.06 | 0.412 | 0.539 | 1.000 | 2485 | tags=56%, list=43%, signal=98% |
| 558 | UVC\_XPCS\_8HR\_UP |  | 23 | -0.44 | -1.06 | 0.414 | 0.541 | 1.000 | 1179 | tags=35%, list=21%, signal=44% |
| 559 | STEFFEN\_AML\_PML\_PLZF\_TRGT |  | 16 | -0.47 | -1.06 | 0.419 | 0.540 | 1.000 | 1421 | tags=50%, list=25%, signal=66% |
| 560 | SIG\_CHEMOTAXIS |  | 21 | -0.44 | -1.06 | 0.405 | 0.543 | 1.000 | 1501 | tags=43%, list=26%, signal=58% |
| 561 | HDACI\_COLON\_BUT2HRS\_UP |  | 24 | -0.43 | -1.05 | 0.424 | 0.547 | 1.000 | 1381 | tags=38%, list=24%, signal=49% |
| 562 | STEMCELL\_COMMON\_UP |  | 103 | -0.37 | -1.05 | 0.410 | 0.552 | 1.000 | 1672 | tags=33%, list=29%, signal=46% |
| 563 | HSA04210\_APOPTOSIS |  | 38 | -0.40 | -1.05 | 0.407 | 0.555 | 1.000 | 1476 | tags=32%, list=26%, signal=42% |
| 564 | CMV\_HCMV\_TIMECOURSE\_ALL\_UP |  | 209 | -0.36 | -1.05 | 0.369 | 0.557 | 1.000 | 1191 | tags=23%, list=21%, signal=29% |
| 565 | HDACI\_COLON\_CUR48HRS\_UP |  | 26 | -0.43 | -1.04 | 0.446 | 0.563 | 1.000 | 1367 | tags=38%, list=24%, signal=50% |
| 566 | SMITH\_HTERT\_DN |  | 26 | -0.42 | -1.04 | 0.433 | 0.566 | 1.000 | 893 | tags=23%, list=16%, signal=27% |
| 567 | GAMMA-UV\_FIBRO\_DN |  | 20 | -0.44 | -1.04 | 0.436 | 0.569 | 1.000 | 1305 | tags=30%, list=23%, signal=39% |
| 568 | NFATPATHWAY |  | 18 | -0.45 | -1.03 | 0.461 | 0.580 | 1.000 | 2220 | tags=61%, list=39%, signal=100% |
| 569 | UVC\_XPCS\_ALL\_UP |  | 25 | -0.43 | -1.03 | 0.445 | 0.579 | 1.000 | 1179 | tags=32%, list=21%, signal=40% |
| 570 | IDX\_TSA\_UP\_CLUSTER6 |  | 76 | -0.37 | -1.03 | 0.437 | 0.586 | 1.000 | 1578 | tags=30%, list=28%, signal=41% |
| 571 | SRC\_ONCOGENIC\_SIGNATURE |  | 28 | -0.41 | -1.03 | 0.474 | 0.589 | 1.000 | 1279 | tags=29%, list=22%, signal=37% |
| 572 | WERNERONLY\_FIBRO\_DN |  | 24 | -0.43 | -1.02 | 0.468 | 0.600 | 1.000 | 1157 | tags=25%, list=20%, signal=31% |
| 573 | AGUIRRE\_PANCREAS\_CHR19 |  | 27 | -0.41 | -1.02 | 0.469 | 0.601 | 1.000 | 846 | tags=26%, list=15%, signal=30% |
| 574 | WERNER\_FIBRO\_DN |  | 72 | -0.37 | -1.02 | 0.487 | 0.602 | 1.000 | 1157 | tags=18%, list=20%, signal=22% |
| 575 | CMV\_24HRS\_UP |  | 31 | -0.40 | -1.02 | 0.469 | 0.603 | 1.000 | 500 | tags=16%, list=9%, signal=18% |
| 576 | GPCRS\_CLASS\_A\_RHODOPSIN\_LIKE |  | 18 | -0.44 | -1.02 | 0.453 | 0.610 | 1.000 | 601 | tags=33%, list=10%, signal=37% |
| 577 | HSC\_STHSC\_FETAL |  | 15 | -0.45 | -1.02 | 0.474 | 0.610 | 1.000 | 1201 | tags=27%, list=21%, signal=34% |
| 578 | UVC\_TTD\_ALL\_UP |  | 24 | -0.41 | -1.01 | 0.481 | 0.621 | 1.000 | 1422 | tags=42%, list=25%, signal=55% |
| 579 | FLECHNER\_KIDNEY\_TRANSPLANT\_WELL\_PBL\_UP |  | 69 | -0.37 | -1.00 | 0.498 | 0.636 | 1.000 | 1747 | tags=41%, list=31%, signal=58% |
| 580 | OLDWERNER\_FIBRO\_DN |  | 49 | -0.37 | -1.00 | 0.509 | 0.645 | 1.000 | 915 | tags=14%, list=16%, signal=17% |
| 581 | DEATHPATHWAY |  | 17 | -0.44 | -1.00 | 0.490 | 0.645 | 1.000 | 1476 | tags=35%, list=26%, signal=47% |
| 582 | HSC\_STHSC\_SHARED |  | 15 | -0.45 | -0.99 | 0.512 | 0.649 | 1.000 | 1201 | tags=27%, list=21%, signal=34% |
| 583 | DRUG\_RESISTANCE\_AND\_METABOLISM |  | 38 | -0.38 | -0.99 | 0.536 | 0.654 | 1.000 | 1171 | tags=32%, list=20%, signal=39% |
| 584 | VANTVEER\_BREAST\_OUTCOME\_GOOD\_VS\_POOR\_DN |  | 33 | -0.39 | -0.99 | 0.532 | 0.659 | 1.000 | 549 | tags=18%, list=10%, signal=20% |
| 585 | LEE\_E2F1\_DN |  | 21 | -0.40 | -0.98 | 0.525 | 0.679 | 1.000 | 559 | tags=24%, list=10%, signal=26% |
| 586 | HIPPOCAMPUS\_DEVELOPMENT\_POSTNATAL |  | 19 | -0.41 | -0.98 | 0.525 | 0.680 | 1.000 | 939 | tags=26%, list=16%, signal=31% |
| 587 | PENG\_RAPAMYCIN\_UP |  | 65 | -0.35 | -0.98 | 0.565 | 0.679 | 1.000 | 1183 | tags=28%, list=21%, signal=35% |
| 588 | LEE\_MYC\_UP |  | 23 | -0.40 | -0.97 | 0.550 | 0.691 | 1.000 | 817 | tags=22%, list=14%, signal=25% |
| 589 | SANSOM\_APC\_LOSS5\_UP |  | 24 | -0.40 | -0.97 | 0.534 | 0.691 | 1.000 | 572 | tags=17%, list=10%, signal=18% |
| 590 | YAGI\_AML\_PROG\_ASSOC |  | 59 | -0.35 | -0.97 | 0.566 | 0.697 | 1.000 | 424 | tags=12%, list=7%, signal=13% |
| 591 | TOLLPATHWAY |  | 18 | -0.41 | -0.97 | 0.532 | 0.697 | 1.000 | 1327 | tags=50%, list=23%, signal=65% |
| 592 | FASPATHWAY |  | 20 | -0.41 | -0.96 | 0.543 | 0.700 | 1.000 | 1476 | tags=45%, list=26%, signal=60% |
| 593 | YAGI\_AML\_PROGNOSIS |  | 18 | -0.41 | -0.96 | 0.545 | 0.705 | 1.000 | 1415 | tags=33%, list=25%, signal=44% |
| 594 | ZHAN\_MM\_MOLECULAR\_CLASSI\_DN |  | 16 | -0.42 | -0.96 | 0.563 | 0.708 | 1.000 | 939 | tags=25%, list=16%, signal=30% |
| 595 | AGUIRRE\_PANCREAS\_CHR8 |  | 16 | -0.41 | -0.95 | 0.558 | 0.719 | 1.000 | 406 | tags=13%, list=7%, signal=13% |
| 596 | CALRES\_RHESUS\_DN |  | 27 | -0.39 | -0.95 | 0.565 | 0.721 | 1.000 | 1578 | tags=26%, list=28%, signal=36% |
| 597 | CIS\_XPC\_UP |  | 53 | -0.36 | -0.95 | 0.590 | 0.720 | 1.000 | 715 | tags=17%, list=12%, signal=19% |
| 598 | CMV\_HCMV\_TIMECOURSE\_6HRS\_DN |  | 26 | -0.39 | -0.95 | 0.562 | 0.721 | 1.000 | 647 | tags=19%, list=11%, signal=22% |
| 599 | HSA04910\_INSULIN\_SIGNALING\_PATHWAY |  | 55 | -0.35 | -0.94 | 0.580 | 0.728 | 1.000 | 1243 | tags=25%, list=22%, signal=32% |
| 600 | ST\_ERK1\_ERK2\_MAPK\_PATHWAY |  | 15 | -0.42 | -0.94 | 0.570 | 0.730 | 1.000 | 2628 | tags=73%, list=46%, signal=135% |
| 601 | CMV\_ALL\_UP |  | 41 | -0.36 | -0.94 | 0.587 | 0.729 | 1.000 | 1158 | tags=27%, list=20%, signal=33% |
| 602 | BCNU\_GLIOMA\_MGMT\_48HRS\_DN |  | 32 | -0.37 | -0.94 | 0.593 | 0.733 | 1.000 | 1115 | tags=28%, list=19%, signal=35% |
| 603 | BRCA\_BRCA1\_POS |  | 54 | -0.35 | -0.94 | 0.613 | 0.736 | 1.000 | 488 | tags=11%, list=9%, signal=12% |
| 604 | REOVIRUS\_HEK293\_UP |  | 116 | -0.33 | -0.93 | 0.638 | 0.741 | 1.000 | 1191 | tags=22%, list=21%, signal=28% |
| 605 | HDACI\_COLON\_CUR24HRS\_UP |  | 20 | -0.39 | -0.93 | 0.583 | 0.744 | 1.000 | 1519 | tags=40%, list=27%, signal=54% |
| 606 | LIZUKA\_G1\_SM\_G2 |  | 17 | -0.41 | -0.93 | 0.594 | 0.747 | 1.000 | 1970 | tags=65%, list=34%, signal=98% |
| 607 | GAY\_YY1\_DN |  | 97 | -0.33 | -0.93 | 0.649 | 0.750 | 1.000 | 764 | tags=19%, list=13%, signal=21% |
| 608 | LEE\_TCELLS8\_UP |  | 78 | -0.33 | -0.93 | 0.643 | 0.749 | 1.000 | 1021 | tags=18%, list=18%, signal=22% |
| 609 | ST\_B\_CELL\_ANTIGEN\_RECEPTOR |  | 27 | -0.37 | -0.92 | 0.600 | 0.758 | 1.000 | 1321 | tags=41%, list=23%, signal=53% |
| 610 | HDACI\_COLON\_BUT\_DN |  | 114 | -0.32 | -0.92 | 0.678 | 0.759 | 1.000 | 1450 | tags=25%, list=25%, signal=32% |
| 611 | BRCA2\_BRCA1\_UP |  | 24 | -0.38 | -0.92 | 0.624 | 0.758 | 1.000 | 1981 | tags=50%, list=35%, signal=76% |
| 612 | LEE\_TCELLS1\_UP |  | 78 | -0.33 | -0.92 | 0.649 | 0.758 | 1.000 | 1021 | tags=18%, list=18%, signal=22% |
| 613 | HSA04130\_SNARE\_INTERACTIONS\_IN\_VESICULAR\_TRANSPORT |  | 15 | -0.40 | -0.92 | 0.620 | 0.759 | 1.000 | 1961 | tags=47%, list=34%, signal=71% |
| 614 | LEE\_TCELLS10\_UP |  | 78 | -0.33 | -0.92 | 0.663 | 0.759 | 1.000 | 1021 | tags=18%, list=18%, signal=22% |
| 615 | FETAL\_LIVER\_ENRICHED\_TRANSCRIPTION\_FACTORS |  | 36 | -0.36 | -0.92 | 0.626 | 0.758 | 1.000 | 1258 | tags=19%, list=22%, signal=25% |
| 616 | UVC\_HIGH\_D4\_DN |  | 29 | -0.37 | -0.92 | 0.625 | 0.760 | 1.000 | 1706 | tags=31%, list=30%, signal=44% |
| 617 | UV-CMV\_UNIQUE\_HCMV\_6HRS\_UP |  | 32 | -0.36 | -0.91 | 0.622 | 0.760 | 1.000 | 813 | tags=25%, list=14%, signal=29% |
| 618 | HUMAN\_CD34\_ENRICHED\_TRANSCRIPTION\_FACTORS |  | 73 | -0.33 | -0.91 | 0.662 | 0.760 | 1.000 | 1609 | tags=34%, list=28%, signal=47% |
| 619 | IDX\_TSA\_UP\_CLUSTER5 |  | 44 | -0.34 | -0.91 | 0.637 | 0.762 | 1.000 | 1344 | tags=23%, list=23%, signal=29% |
| 620 | AGUIRRE\_PANCREAS\_CHR7 |  | 17 | -0.40 | -0.91 | 0.611 | 0.762 | 1.000 | 1047 | tags=24%, list=18%, signal=29% |
| 621 | PEART\_HISTONE\_DN |  | 39 | -0.35 | -0.91 | 0.634 | 0.761 | 1.000 | 419 | tags=13%, list=7%, signal=14% |
| 622 | DSRNA\_UP |  | 16 | -0.40 | -0.91 | 0.620 | 0.760 | 1.000 | 1404 | tags=38%, list=25%, signal=50% |
| 623 | BYSTRYKH\_HSC\_BRAIN\_TRANS\_GLOCUS |  | 75 | -0.33 | -0.91 | 0.663 | 0.759 | 1.000 | 919 | tags=13%, list=16%, signal=16% |
| 624 | P38MAPKPATHWAY |  | 22 | -0.38 | -0.91 | 0.642 | 0.765 | 1.000 | 1243 | tags=41%, list=22%, signal=52% |
| 625 | GLUCONEOGENESIS |  | 15 | -0.40 | -0.91 | 0.604 | 0.765 | 1.000 | 1861 | tags=47%, list=33%, signal=69% |
| 626 | HSA00071\_FATTY\_ACID\_METABOLISM |  | 24 | -0.37 | -0.90 | 0.621 | 0.767 | 1.000 | 521 | tags=13%, list=9%, signal=14% |
| 627 | SHEPARD\_BMYB\_MORPHOLINO\_DN |  | 61 | -0.33 | -0.90 | 0.670 | 0.768 | 1.000 | 1118 | tags=28%, list=20%, signal=34% |
| 628 | BHATTACHARYA\_ESC\_UP |  | 17 | -0.39 | -0.90 | 0.624 | 0.769 | 1.000 | 1316 | tags=35%, list=23%, signal=46% |
| 629 | APOPTOSIS\_KEGG |  | 19 | -0.38 | -0.90 | 0.627 | 0.771 | 1.000 | 1529 | tags=32%, list=27%, signal=43% |
| 630 | HOFFMANN\_BIVSBII\_BI\_TABLE2 |  | 98 | -0.31 | -0.90 | 0.715 | 0.778 | 1.000 | 984 | tags=21%, list=17%, signal=25% |
| 631 | MENSE\_HYPOXIA\_TRANSPORTER\_GENES |  | 20 | -0.38 | -0.89 | 0.635 | 0.781 | 1.000 | 109 | tags=10%, list=2%, signal=10% |
| 632 | ELONGINA\_KO\_DN |  | 65 | -0.32 | -0.89 | 0.679 | 0.784 | 1.000 | 893 | tags=18%, list=16%, signal=22% |
| 633 | GLYCOLYSIS |  | 15 | -0.40 | -0.89 | 0.638 | 0.785 | 1.000 | 1861 | tags=47%, list=33%, signal=69% |
| 634 | FMLPPATHWAY |  | 19 | -0.38 | -0.89 | 0.631 | 0.784 | 1.000 | 1871 | tags=63%, list=33%, signal=94% |
| 635 | STRESS\_TPA\_SPECIFIC\_UP |  | 19 | -0.38 | -0.89 | 0.647 | 0.784 | 1.000 | 1051 | tags=26%, list=18%, signal=32% |
| 636 | LU\_IL4BCELL |  | 27 | -0.36 | -0.88 | 0.662 | 0.788 | 1.000 | 1209 | tags=30%, list=21%, signal=37% |
| 637 | AGUIRRE\_PANCREAS\_CHR22 |  | 21 | -0.37 | -0.88 | 0.643 | 0.793 | 1.000 | 1449 | tags=33%, list=25%, signal=44% |
| 638 | CHESLER\_BRAIN\_CIS\_GENES |  | 31 | -0.35 | -0.88 | 0.667 | 0.792 | 1.000 | 1271 | tags=23%, list=22%, signal=29% |
| 639 | PROPANOATE\_METABOLISM |  | 19 | -0.37 | -0.88 | 0.650 | 0.792 | 1.000 | 306 | tags=11%, list=5%, signal=11% |
| 640 | HDACI\_COLON\_BUT48HRS\_DN |  | 46 | -0.33 | -0.88 | 0.683 | 0.796 | 1.000 | 1442 | tags=30%, list=25%, signal=40% |
| 641 | HSA00564\_GLYCEROPHOSPHOLIPID\_METABOLISM |  | 25 | -0.36 | -0.88 | 0.670 | 0.795 | 1.000 | 1699 | tags=40%, list=30%, signal=57% |
| 642 | CALRES\_MOUSE\_DN |  | 18 | -0.38 | -0.88 | 0.659 | 0.795 | 1.000 | 587 | tags=11%, list=10%, signal=12% |
| 643 | HSC\_INTERMEDIATEPROGENITORS\_FETAL |  | 64 | -0.32 | -0.88 | 0.692 | 0.794 | 1.000 | 861 | tags=19%, list=15%, signal=22% |
| 644 | CMV-UV\_HCMV\_6HRS\_UP |  | 38 | -0.34 | -0.87 | 0.699 | 0.795 | 1.000 | 1372 | tags=37%, list=24%, signal=48% |
| 645 | UVB\_NHEK3\_C1 |  | 19 | -0.37 | -0.87 | 0.641 | 0.796 | 1.000 | 1090 | tags=26%, list=19%, signal=32% |
| 646 | CMV\_HCMV\_TIMECOURSE\_20HRS\_UP |  | 40 | -0.33 | -0.87 | 0.679 | 0.798 | 1.000 | 973 | tags=15%, list=17%, signal=18% |
| 647 | ZHAN\_MM\_CD1\_VS\_CD2\_DN |  | 21 | -0.36 | -0.87 | 0.664 | 0.800 | 1.000 | 943 | tags=29%, list=16%, signal=34% |
| 648 | MAPKPATHWAY |  | 46 | -0.33 | -0.86 | 0.699 | 0.808 | 1.000 | 1926 | tags=43%, list=34%, signal=65% |
| 649 | CHEN\_HOXA5\_TARGETS\_UP |  | 104 | -0.30 | -0.86 | 0.769 | 0.814 | 1.000 | 1426 | tags=22%, list=25%, signal=29% |
| 650 | SA\_B\_CELL\_RECEPTOR\_COMPLEXES |  | 18 | -0.37 | -0.86 | 0.677 | 0.812 | 1.000 | 1243 | tags=33%, list=22%, signal=42% |
| 651 | ALZHEIMERS\_DISEASE\_DN |  | 482 | -0.29 | -0.86 | 0.927 | 0.815 | 1.000 | 1339 | tags=19%, list=23%, signal=22% |
| 652 | H2O2\_CSBDIFF\_C1 |  | 15 | -0.39 | -0.85 | 0.690 | 0.817 | 1.000 | 1717 | tags=40%, list=30%, signal=57% |
| 653 | BASSO\_REGULATORY\_HUBS |  | 69 | -0.31 | -0.85 | 0.760 | 0.817 | 1.000 | 1113 | tags=19%, list=19%, signal=23% |
| 654 | IL2RBPATHWAY |  | 19 | -0.36 | -0.85 | 0.685 | 0.815 | 1.000 | 1926 | tags=47%, list=34%, signal=71% |
| 655 | PASSERINI\_APOPTOSIS |  | 18 | -0.37 | -0.85 | 0.657 | 0.816 | 1.000 | 804 | tags=22%, list=14%, signal=26% |
| 656 | CMV\_HCMV\_TIMECOURSE\_48HRS\_UP |  | 30 | -0.34 | -0.85 | 0.719 | 0.822 | 1.000 | 1108 | tags=30%, list=19%, signal=37% |
| 657 | MAPK\_CASCADE |  | 17 | -0.38 | -0.85 | 0.705 | 0.822 | 1.000 | 2015 | tags=71%, list=35%, signal=109% |
| 658 | HSC\_INTERMEDIATEPROGENITORS\_ADULT |  | 57 | -0.31 | -0.85 | 0.759 | 0.822 | 1.000 | 861 | tags=19%, list=15%, signal=22% |
| 659 | HSC\_LATEPROGENITORS\_ADULT |  | 193 | -0.29 | -0.84 | 0.847 | 0.822 | 1.000 | 1603 | tags=27%, list=28%, signal=36% |
| 660 | PYRUVATE\_METABOLISM |  | 15 | -0.37 | -0.84 | 0.711 | 0.829 | 1.000 | 363 | tags=13%, list=6%, signal=14% |
| 661 | OLDONLY\_FIBRO\_UP |  | 17 | -0.37 | -0.84 | 0.704 | 0.829 | 1.000 | 512 | tags=12%, list=9%, signal=13% |
| 662 | SANA\_TNFA\_ENDOTHELIAL\_UP |  | 26 | -0.34 | -0.84 | 0.719 | 0.828 | 1.000 | 1321 | tags=38%, list=23%, signal=50% |
| 663 | VHL\_RCC\_UP |  | 56 | -0.31 | -0.84 | 0.764 | 0.830 | 1.000 | 1875 | tags=39%, list=33%, signal=58% |
| 664 | PURINE\_METABOLISM |  | 50 | -0.31 | -0.83 | 0.740 | 0.830 | 1.000 | 1186 | tags=26%, list=21%, signal=33% |
| 665 | KLEIN\_PEL\_DN |  | 35 | -0.33 | -0.83 | 0.760 | 0.830 | 1.000 | 1043 | tags=26%, list=18%, signal=31% |
| 666 | AGEING\_KIDNEY\_SPECIFIC\_DN |  | 51 | -0.31 | -0.83 | 0.769 | 0.836 | 1.000 | 857 | tags=18%, list=15%, signal=21% |
| 667 | BREASTCA\_TWO\_CLASSES |  | 64 | -0.30 | -0.83 | 0.790 | 0.840 | 1.000 | 1604 | tags=27%, list=28%, signal=36% |
| 668 | TARTE\_BCELL |  | 21 | -0.35 | -0.82 | 0.717 | 0.839 | 1.000 | 1323 | tags=33%, list=23%, signal=43% |
| 669 | HSC\_INTERMEDIATEPROGENITORS\_SHARED |  | 52 | -0.31 | -0.82 | 0.764 | 0.843 | 1.000 | 1109 | tags=23%, list=19%, signal=28% |
| 670 | HSC\_LATEPROGENITORS\_SHARED |  | 189 | -0.28 | -0.82 | 0.903 | 0.843 | 1.000 | 1603 | tags=27%, list=28%, signal=36% |
| 671 | HSA00620\_PYRUVATE\_METABOLISM |  | 17 | -0.36 | -0.82 | 0.731 | 0.842 | 1.000 | 363 | tags=12%, list=6%, signal=13% |
| 672 | HDACI\_COLON\_BUT12HRS\_DN |  | 34 | -0.31 | -0.82 | 0.750 | 0.844 | 1.000 | 399 | tags=9%, list=7%, signal=9% |
| 673 | FETAL\_LIVER\_VS\_ADULT\_LIVER\_GNF2 |  | 16 | -0.35 | -0.81 | 0.744 | 0.848 | 1.000 | 1266 | tags=31%, list=22%, signal=40% |
| 674 | OLDAGE\_DN |  | 24 | -0.34 | -0.81 | 0.739 | 0.847 | 1.000 | 505 | tags=13%, list=9%, signal=14% |
| 675 | SIG\_PIP3\_SIGNALING\_IN\_B\_LYMPHOCYTES |  | 19 | -0.34 | -0.81 | 0.729 | 0.848 | 1.000 | 1020 | tags=26%, list=18%, signal=32% |
| 676 | GOLDRATH\_HP |  | 80 | -0.29 | -0.81 | 0.815 | 0.850 | 1.000 | 1374 | tags=23%, list=24%, signal=29% |
| 677 | BRCA\_PROGNOSIS\_NEG |  | 52 | -0.30 | -0.81 | 0.799 | 0.854 | 1.000 | 549 | tags=13%, list=10%, signal=15% |
| 678 | UVB\_NHEK3\_C7 |  | 25 | -0.33 | -0.80 | 0.775 | 0.858 | 1.000 | 580 | tags=16%, list=10%, signal=18% |
| 679 | HSC\_LATEPROGENITORS\_FETAL |  | 191 | -0.28 | -0.80 | 0.910 | 0.857 | 1.000 | 1603 | tags=27%, list=28%, signal=36% |
| 680 | HSA00020\_CITRATE\_CYCLE |  | 18 | -0.35 | -0.80 | 0.750 | 0.856 | 1.000 | 941 | tags=17%, list=16%, signal=20% |
| 681 | UVB\_SCC\_UP |  | 44 | -0.30 | -0.80 | 0.803 | 0.859 | 1.000 | 1399 | tags=25%, list=24%, signal=33% |
| 682 | APOPTOSIS\_GENMAPP |  | 20 | -0.34 | -0.79 | 0.770 | 0.862 | 1.000 | 1476 | tags=30%, list=26%, signal=40% |
| 683 | WANG\_MLL\_CBP\_VS\_GMP\_DN |  | 23 | -0.33 | -0.79 | 0.771 | 0.866 | 1.000 | 727 | tags=13%, list=13%, signal=15% |
| 684 | HSC\_EARLYPROGENITORS\_ADULT |  | 193 | -0.27 | -0.79 | 0.934 | 0.870 | 1.000 | 1441 | tags=22%, list=25%, signal=28% |
| 685 | HSA00252\_ALANINE\_AND\_ASPARTATE\_METABOLISM |  | 16 | -0.35 | -0.79 | 0.768 | 0.869 | 1.000 | 728 | tags=13%, list=13%, signal=14% |
| 686 | ALZHEIMERS\_INCIPIENT\_DN |  | 71 | -0.28 | -0.78 | 0.844 | 0.869 | 1.000 | 361 | tags=7%, list=6%, signal=7% |
| 687 | LYSINE\_DEGRADATION |  | 17 | -0.34 | -0.78 | 0.777 | 0.868 | 1.000 | 264 | tags=12%, list=5%, signal=12% |
| 688 | FERRANDO\_MLL\_T\_ALL\_DN |  | 46 | -0.29 | -0.78 | 0.834 | 0.874 | 1.000 | 899 | tags=15%, list=16%, signal=18% |
| 689 | LEE\_MYC\_TGFA\_DN |  | 20 | -0.33 | -0.78 | 0.777 | 0.876 | 1.000 | 694 | tags=25%, list=12%, signal=28% |
| 690 | HDACI\_COLON\_CLUSTER10 |  | 17 | -0.34 | -0.77 | 0.777 | 0.879 | 1.000 | 1492 | tags=24%, list=26%, signal=32% |
| 691 | RCC\_NL\_UP |  | 254 | -0.26 | -0.77 | 0.968 | 0.885 | 1.000 | 1477 | tags=22%, list=26%, signal=28% |
| 692 | MYC\_TARGETS |  | 18 | -0.32 | -0.76 | 0.806 | 0.886 | 1.000 | 758 | tags=17%, list=13%, signal=19% |
| 693 | HSC\_EARLYPROGENITORS\_SHARED |  | 192 | -0.26 | -0.76 | 0.942 | 0.888 | 1.000 | 1441 | tags=21%, list=25%, signal=28% |
| 694 | HSC\_EARLYPROGENITORS\_FETAL |  | 192 | -0.26 | -0.75 | 0.957 | 0.894 | 1.000 | 1441 | tags=21%, list=25%, signal=28% |
| 695 | LEE\_DENA\_DN |  | 20 | -0.32 | -0.74 | 0.815 | 0.907 | 1.000 | 817 | tags=20%, list=14%, signal=23% |
| 696 | ST\_FAS\_SIGNALING\_PATHWAY |  | 30 | -0.29 | -0.74 | 0.846 | 0.909 | 1.000 | 163 | tags=7%, list=3%, signal=7% |
| 697 | HYPOXIA\_RCC\_NOVHL\_UP |  | 28 | -0.29 | -0.74 | 0.836 | 0.911 | 1.000 | 2044 | tags=50%, list=36%, signal=77% |
| 698 | CALRES\_MOUSE\_NEOCORTEX\_DN |  | 32 | -0.29 | -0.73 | 0.845 | 0.910 | 1.000 | 865 | tags=22%, list=15%, signal=26% |
| 699 | ST\_P38\_MAPK\_PATHWAY |  | 17 | -0.32 | -0.73 | 0.815 | 0.912 | 1.000 | 1157 | tags=29%, list=20%, signal=37% |
| 700 | FLECHNER\_KIDNEY\_TRANSPLANT\_REJECTION\_PBL\_UP |  | 37 | -0.28 | -0.73 | 0.861 | 0.915 | 1.000 | 1598 | tags=30%, list=28%, signal=41% |
| 701 | HSA00230\_PURINE\_METABOLISM |  | 73 | -0.26 | -0.72 | 0.902 | 0.919 | 1.000 | 1189 | tags=23%, list=21%, signal=29% |
| 702 | VHL\_NORMAL\_UP |  | 189 | -0.25 | -0.72 | 0.971 | 0.921 | 1.000 | 1663 | tags=26%, list=29%, signal=36% |
| 703 | PRMT5\_KD\_UP |  | 95 | -0.25 | -0.72 | 0.924 | 0.921 | 1.000 | 620 | tags=11%, list=11%, signal=12% |
| 704 | OXIDATIVE\_PHOSPHORYLATION |  | 26 | -0.29 | -0.71 | 0.868 | 0.926 | 1.000 | 1245 | tags=19%, list=22%, signal=24% |
| 705 | HSA04662\_B\_CELL\_RECEPTOR\_SIGNALING\_PATHWAY |  | 39 | -0.27 | -0.70 | 0.886 | 0.931 | 1.000 | 1321 | tags=28%, list=23%, signal=36% |
| 706 | AGUIRRE\_PANCREAS\_CHR17 |  | 29 | -0.28 | -0.70 | 0.884 | 0.931 | 1.000 | 1230 | tags=21%, list=21%, signal=26% |
| 707 | FLECHNER\_KIDNEY\_TRANSPLANT\_REJECTION\_PBL\_DN |  | 24 | -0.29 | -0.70 | 0.885 | 0.934 | 1.000 | 1790 | tags=33%, list=31%, signal=48% |
| 708 | SIG\_BCR\_SIGNALING\_PATHWAY |  | 30 | -0.28 | -0.70 | 0.879 | 0.933 | 1.000 | 1266 | tags=33%, list=22%, signal=43% |
| 709 | UVB\_NHEK2\_DN |  | 39 | -0.27 | -0.69 | 0.902 | 0.940 | 1.000 | 1393 | tags=21%, list=24%, signal=27% |
| 710 | GLYCEROPHOSPHOLIPID\_METABOLISM |  | 22 | -0.29 | -0.69 | 0.894 | 0.939 | 1.000 | 1699 | tags=36%, list=30%, signal=52% |
| 711 | ZHAN\_MM\_CD1\_VS\_CD2\_UP |  | 27 | -0.28 | -0.69 | 0.880 | 0.939 | 1.000 | 1010 | tags=15%, list=18%, signal=18% |
| 712 | BREASTCA\_THREE\_CLASSES |  | 16 | -0.30 | -0.68 | 0.883 | 0.942 | 1.000 | 2195 | tags=50%, list=38%, signal=81% |
| 713 | INOS\_ALL\_UP |  | 30 | -0.27 | -0.68 | 0.887 | 0.942 | 1.000 | 766 | tags=13%, list=13%, signal=15% |
| 714 | VALINE\_LEUCINE\_AND\_ISOLEUCINE\_DEGRADATION |  | 25 | -0.27 | -0.67 | 0.913 | 0.946 | 1.000 | 306 | tags=8%, list=5%, signal=8% |
| 715 | AGED\_MOUSE\_HYPOTH\_UP |  | 23 | -0.28 | -0.67 | 0.893 | 0.946 | 1.000 | 1170 | tags=17%, list=20%, signal=22% |
| 716 | HSA00500\_STARCH\_AND\_SUCROSE\_METABOLISM |  | 26 | -0.27 | -0.66 | 0.900 | 0.955 | 1.000 | 1917 | tags=35%, list=33%, signal=52% |
| 717 | BCRPATHWAY |  | 23 | -0.27 | -0.65 | 0.911 | 0.962 | 1.000 | 1243 | tags=30%, list=22%, signal=39% |
| 718 | SANA\_IFNG\_ENDOTHELIAL\_DN |  | 27 | -0.26 | -0.64 | 0.914 | 0.964 | 1.000 | 1446 | tags=26%, list=25%, signal=35% |
| 719 | CERAMIDEPATHWAY |  | 15 | -0.28 | -0.62 | 0.920 | 0.974 | 1.000 | 1517 | tags=33%, list=27%, signal=45% |
| 720 | HDACI\_COLON\_BUT16HRS\_DN |  | 45 | -0.23 | -0.62 | 0.962 | 0.976 | 1.000 | 580 | tags=9%, list=10%, signal=10% |
| 721 | SHIPP\_DLBCL\_CURED\_DN |  | 18 | -0.27 | -0.62 | 0.937 | 0.974 | 1.000 | 1609 | tags=28%, list=28%, signal=39% |
| 722 | OXSTRESS\_RPE\_H2O2HNE\_DN |  | 17 | -0.27 | -0.61 | 0.929 | 0.975 | 1.000 | 1831 | tags=41%, list=32%, signal=60% |
| 723 | H2O2\_CSBDIFF\_C2 |  | 17 | -0.26 | -0.60 | 0.940 | 0.980 | 1.000 | 111 | tags=6%, list=2%, signal=6% |
| 724 | STRESS\_GENOTOXIC\_SPECIFIC\_DN |  | 17 | -0.26 | -0.59 | 0.931 | 0.983 | 1.000 | 904 | tags=18%, list=16%, signal=21% |
| 725 | PEART\_HISTONE\_UP |  | 28 | -0.23 | -0.58 | 0.961 | 0.987 | 1.000 | 1290 | tags=21%, list=23%, signal=28% |
| 726 | UVB\_NHEK1\_DN |  | 129 | -0.20 | -0.58 | 0.995 | 0.986 | 1.000 | 1417 | tags=19%, list=25%, signal=24% |
| 727 | UVB\_NHEK1\_C6 |  | 59 | -0.22 | -0.58 | 0.982 | 0.987 | 1.000 | 1034 | tags=12%, list=18%, signal=14% |
| 728 | BRENTANI\_DEATH |  | 28 | -0.22 | -0.56 | 0.968 | 0.993 | 1.000 | 1476 | tags=21%, list=26%, signal=29% |
| 729 | HSA00640\_PROPANOATE\_METABOLISM |  | 19 | -0.24 | -0.55 | 0.969 | 0.993 | 1.000 | 1398 | tags=21%, list=24%, signal=28% |
| 730 | IDX\_TSA\_DN\_CLUSTER6 |  | 15 | -0.25 | -0.55 | 0.960 | 0.993 | 1.000 | 1359 | tags=27%, list=24%, signal=35% |
| 731 | GENOTOXINS\_24HRS\_DISCR |  | 16 | -0.24 | -0.54 | 0.966 | 0.994 | 1.000 | 419 | tags=6%, list=7%, signal=7% |
| 732 | PENG\_RAPAMYCIN\_DN |  | 106 | -0.18 | -0.53 | 0.999 | 0.997 | 1.000 | 964 | tags=9%, list=17%, signal=11% |
| 733 | AGUIRRE\_PANCREAS\_CHR6 |  | 15 | -0.23 | -0.52 | 0.985 | 0.997 | 1.000 | 2124 | tags=53%, list=37%, signal=85% |
| 734 | MMS\_MOUSE\_LYMPH\_HIGH\_4HRS\_UP |  | 21 | -0.22 | -0.52 | 0.980 | 0.996 | 1.000 | 1878 | tags=48%, list=33%, signal=71% |
| 735 | BYSTRYKH\_HSC\_BRAIN\_CIS\_GLOCUS |  | 28 | -0.21 | -0.52 | 0.987 | 0.995 | 1.000 | 1900 | tags=29%, list=33%, signal=43% |
| 736 | BRCA1\_OVEREXP\_DN |  | 50 | -0.18 | -0.50 | 0.996 | 0.998 | 1.000 | 949 | tags=12%, list=17%, signal=14% |
| 737 | ST\_PHOSPHOINOSITIDE\_3\_KINASE\_PATHWAY |  | 17 | -0.22 | -0.49 | 0.988 | 0.997 | 1.000 | 1926 | tags=35%, list=34%, signal=53% |
| 738 | PENG\_GLUCOSE\_DN |  | 61 | -0.17 | -0.47 | 1.000 | 0.999 | 1.000 | 1981 | tags=33%, list=35%, signal=50% |
| 739 | HSA00010\_GLYCOLYSIS\_AND\_GLUCONEOGENESIS |  | 16 | -0.20 | -0.45 | 0.996 | 1.000 | 1.000 | 1861 | tags=38%, list=33%, signal=55% |
| 740 | AGUIRRE\_PANCREAS\_CHR12 |  | 25 | -0.16 | -0.40 | 0.999 | 1.000 | 1.000 | 1775 | tags=28%, list=31%, signal=40% |
| 741 | NOUZOVA\_CPG\_H4\_UP |  | 50 | -0.14 | -0.38 | 1.000 | 1.000 | 1.000 | 1853 | tags=26%, list=32%, signal=38% |
| 742 | HDACI\_COLON\_CLUSTER6 |  | 18 | -0.16 | -0.37 | 0.998 | 0.999 | 1.000 | 1755 | tags=28%, list=31%, signal=40% |
Table: Gene sets enriched in phenotype **NormalMicroarray (3 samples)**[plain text format]****

  
