# Supplementary material for: Comparative RNA-Seq and Microarray Analysis of Gene Expression Changes in B-Cell Lymphomas of Canis familiaris
Source: PLoS One. 2013 Apr 4;8(4):e61088. doi: 10.1371/journal.pone.0061088 (PMC3617154; doi:10.1371/journal.pone.0061088)
Supplement: Data File S2 — GSEA Results Files. (ZIP) [file pone.0061088.s005.zip › Array/index-array-v2.html]

Index for xtools.gsea.Gsea my\_analysis.Gsea.1334779850806

### GSEA Report for Dataset master-all-probes

#### Enrichment in phenotype: **LymphomaMicroarray (4 samples)**

- 110 / 852 gene sets are upregulated in phenotype **LymphomaMicroarray**- 58 gene sets are significant at FDR < 25%- 37 gene sets are significantly enriched at nominal pvalue < 1%- 45 gene sets are significantly enriched at nominal pvalue < 5%- Snapshot of enrichment results- Detailed enrichment results in html format- Detailed enrichment results in excel format (tab delimited text)- Guide to interpret results

#### Enrichment in phenotype: **NormalMicroarray (3 samples)**

- 742 / 852 gene sets are upregulated in phenotype **NormalMicroarray**- 426 gene sets are significantly enriched at FDR < 25%- 209 gene sets are significantly enriched at nominal pvalue < 1%- 315 gene sets are significantly enriched at nominal pvalue < 5%- Snapshot of enrichment results- Detailed enrichment results in html format- Detailed enrichment results in excel format (tab delimited text)- Guide to interpret results

#### Dataset details

- The dataset has 5724 features (genes)- No probe set => gene symbol collapsing was requested, so all 5724 features were used

#### Gene set details

- Gene set size filters (min=15, max=500) resulted in filtering out 1040 / 1892 gene sets- The remaining 852 gene sets were used in the analysis- List of gene sets used and their sizes (restricted to features in the specified dataset)

#### Gene markers for the **LymphomaMicroarray** *versus* **NormalMicroarray** comparison

- The dataset has 5724 features (genes)- # of markers for phenotype **LymphomaMicroarray**: 2642 (46.2% ) with correlation area 30.9%- # of markers for phenotype **NormalMicroarray**: 3082 (53.8% ) with correlation area 69.1%- Detailed rank ordered gene list for all features in the dataset- Heat map and gene list correlation  profile for all features in the dataset

#### Global statistics and plots

- Plot of p-values *vs.* NES- Global ES histogram

#### Other

- Parameters used for this analysis

#### Comments

- There were duplicate row identifiers in the specified dataset. One id was arbitarilly choosen. Details are below
  Generally, this is OK, but if you want to avoid this automagic, edit your dataset so that all row ids are unique
  # of row ids in original dataset: 5733
  # of row UNIQUE ids in original dataset: 5724
  # The duplicates were
  NA
  NA
  NA
  NA
  NA
  NA
  NA
  NA
  NA

---

Report: my\_analysis.Gsea.1334779850806.rpt   by user: Marie.Mooney

xtools.gsea.Gsea [Wed, Apr 18, '12 4 PM 10]

Website: www.broadinstitute.org/GSEA
Questions & Suggestions: Email
