# Supplementary material for: Comparative RNA-Seq and Microarray Analysis of Gene Expression Changes in B-Cell Lymphomas of Canis familiaris
Source: PLoS One. 2013 Apr 4;8(4):e61088. doi: 10.1371/journal.pone.0061088 (PMC3617154; doi:10.1371/journal.pone.0061088)
Supplement: Data File S2 — GSEA Results Files. (ZIP) [file pone.0061088.s005.zip › Array/index-array-v3.html]

Index for xtools.gsea.Gsea my\_analysis.Gsea.1334936519408

### GSEA Report for Dataset master-all-probes

#### Enrichment in phenotype: **LymphomaArray (4 samples)**

- 306 / 1605 gene sets are upregulated in phenotype **LymphomaArray**- 215 gene sets are significant at FDR < 25%- 129 gene sets are significantly enriched at nominal pvalue < 1%- 160 gene sets are significantly enriched at nominal pvalue < 5%- Snapshot of enrichment results- Detailed enrichment results in html format- Detailed enrichment results in excel format (tab delimited text)- Guide to interpret results

#### Enrichment in phenotype: **Normal (3 samples)**

- 1299 / 1605 gene sets are upregulated in phenotype **Normal**- 776 gene sets are significantly enriched at FDR < 25%- 425 gene sets are significantly enriched at nominal pvalue < 1%- 608 gene sets are significantly enriched at nominal pvalue < 5%- Snapshot of enrichment results- Detailed enrichment results in html format- Detailed enrichment results in excel format (tab delimited text)- Guide to interpret results

#### Dataset details

- The dataset has 5723 features (genes)- No probe set => gene symbol collapsing was requested, so all 5723 features were used

#### Gene set details

- Gene set size filters (min=15, max=500) resulted in filtering out 1667 / 3272 gene sets- The remaining 1605 gene sets were used in the analysis- List of gene sets used and their sizes (restricted to features in the specified dataset)

#### Gene markers for the **LymphomaArray** *versus* **Normal** comparison

- The dataset has 5723 features (genes)- # of markers for phenotype **LymphomaArray**: 2641 (46.1% ) with correlation area 30.9%- # of markers for phenotype **Normal**: 3082 (53.9% ) with correlation area 69.1%- Detailed rank ordered gene list for all features in the dataset- Heat map and gene list correlation  profile for all features in the dataset

#### Global statistics and plots

- Plot of p-values *vs.* NES- Global ES histogram

#### Other

- Parameters used for this analysis

---

Report: my\_analysis.Gsea.1334936519408.rpt   by user: Marie.Mooney

xtools.gsea.Gsea [Fri, Apr 20, '12 11 AM 41]

Website: www.broadinstitute.org/GSEA
Questions & Suggestions: Email
