# Supplementary material for: Comparative RNA-Seq and Microarray Analysis of Gene Expression Changes in B-Cell Lymphomas of Canis familiaris
Source: PLoS One. 2013 Apr 4;8(4):e61088. doi: 10.1371/journal.pone.0061088 (PMC3617154; doi:10.1371/journal.pone.0061088)
Supplement: Data File S3 — GeneGo Reports. (ZIP) [file pone.0061088.s006.zip › Report-UP-diff-chip-t.zip!/Summary_1.rtf]

Compare Experiments Workflow 1.0 Data Analysis Report
Server:		portal.genego.com
Date:		2012‑04‑09
Name:		Van Andel ‑ Craig Webb
Login:		vanandel1
Experiments

 1.	 	 topology‑UP‑diff‑t‑chip	
 2.	 	 up‑diff‑chip‑t	

Figure 1. The experiments uploaded for comparative analysis

Table of content:

	Experiments comparison
	Enrichment analysis
	Pathway Maps
	Top maps (sorted by common)
	1. Map : Development_GM‑CSF signaling
	2. Map : Signal transduction_AKT signaling
	3. Map : Immune response_Antigen presentation by MHC class I
	4. Map : Immune response_BCR pathway
	5. Map : DNA damage_ATM/ATR regulation of G1/S checkpoint
	GO Processes
	Process Networks
	Diseases (by Biomarkers)
	Most relevant networks
	Top scored networks for common
	Network 1
	Network 2
	Top scored networks for Unique for topology‑UP‑diff‑t‑chip
	Network 1
	Network 2
	Network 3
	Top scored networks for Unique for up‑diff‑chip‑t
	Network 1
	Network 2
	Network 3


Experiments comparison	(TOC)


Figure 2. The gene content is aligned between all uploaded experiments. The intersection set of experiments is defined as 'common' and marked as a blue/white striped bar. The unique genes for the experiments are marked as colored bars. The genes from the 'similar' set are present in all but one (any) file. The parameters for comparison are set as above.

Enrichment analysis	(TOC)
	Enrichment analysis consists of matching gene IDs of possible targets for the "common", "similar" and "unique" sets with gene IDs in functional ontologies in MetaCore. The probability of a random intersection between a set of IDs the size of target list with ontology entities is estimated in p‑value of hypergeometric intersection. The lower p‑value means higher relevance of the entity to the dataset, which shows in higher rating for the entity.
 Ontologies available for EA in Compare Experiments Workflow: 

Pathway Maps	(TOC)
Canonical pathway maps represent a set of signaling and metabolic maps covering human in a comprehensive way. All maps are created by Thomson Reuters scientists by a high‑quality manual curation process based on published peer‑reviewed literature. Experimental data is visualized on the maps as blue (for downregulation) and red (upregulation) histograms. The height of the histogram corresponds to the relative expression value for a particular gene/protein. 


Figure 3. Pathway Maps. Sorting is done for the 'common' set.

Top maps (sorted by common)
1. Map : Development_GM‑CSF signaling	(TOC)


Figure 4. The top scored map (map with the the lowest p‑value) based on the enrichment distribution sorted by 'common' set. Experimental data from all files is linked to and visualized on the maps as thermometer‑like figures. Up‑ward thermometers have red color and indicate up‑regulated signals and down‑ward (blue) ones indicate down‑regulated expression levels of the genes.

2. Map : Signal transduction_AKT signaling	(TOC)


Figure 5. The second scored map (map with the second lowest p‑value) based on the enrichment distribution sorted by 'common' set. Experimental data from all files is linked to and visualized on the maps as thermometer‑like figures. Up‑ward thermometers have red color and indicate up‑regulated signals and down‑ward (blue) ones indicate down‑regulated expression levels of the genes.

3. Map : Immune response_Antigen presentation by MHC class I	(TOC)


Figure 6. The third scored map (map with the third lowest p‑value) based on the enrichment distribution sorted by 'common' set. Experimental data from all files is linked to and visualized on the maps as thermometer‑like figures. Up‑ward thermometers have red color and indicate up‑regulated signals and down‑ward (blue) ones indicate down‑regulated expression levels of the genes.

4. Map : Immune response_BCR pathway	(TOC)


Figure 7. The fourth scored map (map with the fourth lowest p‑value) based on the enrichment distribution sorted by 'common' set. Experimental data from all files is linked to and visualized on the maps as thermometer‑like figures. Up‑ward thermometers have red color and indicate up‑regulated signals and down‑ward (blue) ones indicate down‑regulated expression levels of the genes.

5. Map : DNA damage_ATM/ATR regulation of G1/S checkpoint	(TOC)


Figure 8. The fifth scored map (map with the fifth lowest p‑value) based on the enrichment distribution sorted by 'common' set. Experimental data from all files is linked to and visualized on the maps as thermometer‑like figures. Up‑ward thermometers have red color and indicate up‑regulated signals and down‑ward (blue) ones indicate down‑regulated expression levels of the genes.

GO Processes	(TOC)
These are Gene Ontology (GO) cellular processes. As most GO processes have no gene/protein content, the "empty terms" are excluded from p‑value calculations. 


Figure 9. GO Processes. Sorting is done for the 'common' set.

Process Networks	(TOC)
The content of these cellular and molecular processes is defined and annotated by Thomson Reuters scientists. Each process represents a pre‑set network of protein interactions characteristic for the process. 


Figure 10. Process Networks. Sorting is done for the 'common' set.

Diseases (by Biomarkers)	(TOC)
Disease folders are organized into a hierarchical tree. Gene content may very greatly between such complex diseases as cancers and some Mendelian diseases. Also, coverage of different diseases in literature is skewed. These two factors may affect p‑value prioritization for diseases. 


Figure 11. Diseases (by Biomarkers). Sorting is done for the 'common' set.

Most relevant networks	(TOC)
The gene content of the uploaded files is used as the input list for generation of biological networks using Analyze Networks (AN) algorithm with default settings. This is a variant of the shortest paths algorithm with main parameters of 1. relative enrichment with the uploaded data, and 2. relative saturation of networks with canonical pathways. These networks are built on the fly and unique for the uploaded data. In this workflow the networks are prioritized based on the number of fragments of canonical pathways on the network.

No	Processes	Size	Target	Pathways	zScore	gScore	
common	
1	response to wounding (64.3%), regulation of multicellular organismal development (64.3%), response to stress (83.3%), positive regulation of developmental process (54.8%), immune system process (69.0%)	43	5	0	  19.93	  19.93	
2	serotonin receptor signaling pathway (41.7%), cell surface receptor linked signaling pathway (87.5%), second‑messenger‑mediated signaling (47.9%), G‑protein signaling, coupled to cyclic nucleotide second messenger (37.5%), G‑protein coupled receptor signaling pathway (64.6%)	50	2	0	   7.13	   7.13	
similar	
Unique for topology‑UP‑diff‑t‑chip	
1	enzyme linked receptor protein signaling pathway (50.0%), positive regulation of cellular process (79.5%), positive regulation of biological process (81.8%), nerve growth factor receptor signaling pathway (34.1%), transmembrane receptor protein tyrosine kinase signaling pathway (43.2%)	54	43	4	  59.68	  64.68	
2	positive regulation of cellular metabolic process (74.5%), positive regulation of macromolecule biosynthetic process (66.0%), positive regulation of metabolic process (74.5%), positive regulation of cellular biosynthetic process (66.0%), positive regulation of macromolecule metabolic process (70.2%)	50	45	0	  64.65	  64.65	
3	positive regulation of cellular metabolic process (57.5%), positive regulation of metabolic process (57.5%), positive regulation of cellular biosynthetic process (50.0%), positive regulation of biosynthetic process (50.0%), regulation of cell cycle (40.0%)	50	27	17	  41.63	  62.88	
Unique for up‑diff‑chip‑t	
1	transcription initiation, DNA‑dependent (16.7%), histone deubiquitination (10.4%), transcription initiation from RNA polymerase II promoter (14.6%), interphase of mitotic cell cycle (22.9%), transcription from RNA polymerase II promoter (22.9%)	50	50	0	  32.59	  32.59	
2	mitochondrial electron transport, NADH to ubiquinone (34.0%), respiratory electron transport chain (40.0%), ATP synthesis coupled electron transport (34.0%), mitochondrial ATP synthesis coupled electron transport (34.0%), oxidative phosphorylation (34.0%)	50	50	0	  31.93	  31.93	
3	mitotic cell cycle (28.0%), interphase of mitotic cell cycle (22.0%), interphase (22.0%), cell cycle phase (28.0%), cell cycle process (30.0%)	50	48	0	  31.22	  31.22	


Top scored networks for common
1.	(TOC)


Figure 12.  The top scored (by the number of pathways) AN network from common. Thick cyan lines indicate the fragments of canonical pathways. Up‑regulated genes are marked with red circles; down‑regulated with blue circles. The 'checkerboard' color indicates mixed expression for the gene between files or between multiple tags for the same gene.

2.	(TOC)


Figure 13.  The second scored (by the number of pathways) AN network from common. Thick cyan lines indicate the fragments of canonical pathways. Up‑regulated genes are marked with red circles; down‑regulated with blue circles. The 'checkerboard' color indicates mixed expression for the gene between files or between multiple tags for the same gene.

Top scored networks for Unique for topology‑UP‑diff‑t‑chip
1.	(TOC)


Figure 14.  The top scored (by the number of pathways) AN network from Unique for topology‑UP‑diff‑t‑chip. Thick cyan lines indicate the fragments of canonical pathways. Up‑regulated genes are marked with red circles; down‑regulated with blue circles. The 'checkerboard' color indicates mixed expression for the gene between files or between multiple tags for the same gene.

2.	(TOC)


Figure 15.  The second scored (by the number of pathways) AN network from Unique for topology‑UP‑diff‑t‑chip. Thick cyan lines indicate the fragments of canonical pathways. Up‑regulated genes are marked with red circles; down‑regulated with blue circles. The 'checkerboard' color indicates mixed expression for the gene between files or between multiple tags for the same gene.

3.	(TOC)


Figure 16.  The third scored (by the number of pathways) AN network from Unique for topology‑UP‑diff‑t‑chip. Thick cyan lines indicate the fragments of canonical pathways. Up‑regulated genes are marked with red circles; down‑regulated with blue circles. The 'checkerboard' color indicates mixed expression for the gene between files or between multiple tags for the same gene.

Top scored networks for Unique for up‑diff‑chip‑t
1.	(TOC)


Figure 17.  The top scored (by the number of pathways) AN network from Unique for up‑diff‑chip‑t. Thick cyan lines indicate the fragments of canonical pathways. Up‑regulated genes are marked with red circles; down‑regulated with blue circles. The 'checkerboard' color indicates mixed expression for the gene between files or between multiple tags for the same gene.

2.	(TOC)


Figure 18.  The second scored (by the number of pathways) AN network from Unique for up‑diff‑chip‑t. Thick cyan lines indicate the fragments of canonical pathways. Up‑regulated genes are marked with red circles; down‑regulated with blue circles. The 'checkerboard' color indicates mixed expression for the gene between files or between multiple tags for the same gene.

3.	(TOC)


Figure 19.  The third scored (by the number of pathways) AN network from Unique for up‑diff‑chip‑t. Thick cyan lines indicate the fragments of canonical pathways. Up‑regulated genes are marked with red circles; down‑regulated with blue circles. The 'checkerboard' color indicates mixed expression for the gene between files or between multiple tags for the same gene.
